# Regional absorption of talinolol mediated by intestinal transporters: insights from PBPK modeling analysis

**DOI:** 10.3389/fphar.2026.1726481

**Published:** 2026-02-11

**Authors:** Kazuya Ishida, Xiaomin Liang, Fulden Buyukozturk, Jia Hao, Christine Wan, Yurong Lai

**Affiliations:** 1 Drug Metabolism, Gilead Sciences, Inc., Foster City, CA, United States; 2 MathWorks, Natick, MA, United States

**Keywords:** drug transporter, intestinal absorption, P-glycoprotein, physiologically-based pharmacokinetic model, talinolol

## Abstract

Intestinal transporters play a pivotal role in oral absorption dynamics to shape the plasma or blood concentration-time curves of drugs, influencing interindividual pharmacokinetic (PK) variability and drug-drug or drug-food interactions. Plasma concentration-time profiles of several drugs, such as talinolol, bedaquiline, irbesartan, and amisulpride, exhibit dose-dependent dual or multiple absorption peaks, yet the mechanism underlying these phenomena remains elusive. It is hypothesized that the regional expression and interplay of intestinal transporters contribute to the observed dual peaks. To elucidate the mechanisms underlying these unique absorption phenomena, the concentration- and pH-dependent transport of talinolol, a substrate of intestinal transporter P-glycoprotein (P-gp) and organic anion transporting polypeptide (OATP) 2B1, was examined using Caco-2 cells, along with the development of a comprehensive physiologically-based pharmacokinetic (PBPK) model that includes a multi-layer gut wall within an advanced dissolution, absorption, and metabolism (M-ADAM) model built in SimBiology^®^ (MathWorks). The basolateral to apical permeability of talinolol in Caco-2 cells decreased as the extracellular pH decreased at the donor side, but the apical to basolateral permeability did not significantly change, indicating that the transport of talinolol by P-gp is pH-dependent. However, when incorporating Caco-2 permeability data and regional differences in P-gp expression, the PBPK model could not reproduce the plasma concentration profiles of talinolol reported in the literature. Increasing the active uptake on the apical membrane of enterocytes improved the curve fit but did not capture the dual peak profiles. Additionally, adjusting regional transporter activity—specifically lowering apical uptake and/or basolateral efflux transport in the lower jejunum and increasing them in the lower ileum—was key to describing the dual absorption peaks of talinolol. These findings indicate that regional differences in intestinal uptake and efflux influence the complex absorption profiles of talinolol and highlight the need for further investigation into additional transporter roles in basolateral transport in the enterocytes.

## 1 Introduction

Intestinal drug absorption is determined by multiple factors including dissolution of the compound, metabolism in the enterocytes, permeability, and active transport across the brush border and basolateral membrane. The compound dissolution is affected by multiple factors such as pH, bile salt concentration, and intestinal fluid volume (Mudie et al., 2010). Once the compound is dissolved, it penetrates the plasma membrane of enterocytes by passive diffusion and/or uptake transporters or is absorbed via a paracellular route (Barthe et al., 1999). Among the transporters expressed in the intestine, P-glycoprotein (P-gp), one of the most studied transporters, is found on the apical membrane of enterocytes (Murakami and Takano, 2008). Other efflux transporters, such as breast cancer resistance protein (BCRP), can act similarly to P-gp. They pump xenobiotics out into the intestinal lumen and act as a barrier in the intestine. In the enterocytes, drugs can be metabolized by drug-metabolizing enzymes such as cytochrome P450s (CYPs) and uridine diphosphate-glucuronosyltransferases (UGTs). Further, to transfer the compound from the enterocyte to the bloodstream (via the portal vein), it must pass through the basolateral membrane of the enterocytes, where several drug transporters, including multidrug resistance-associated protein (MRP) 3, are present (Estudante et al., 2013). Passive diffusion, driven by the concentration gradient of the compound, is considered a mechanism for the movement of the compound from enterocytes to the bloodstream (also known as sink condition). However, the role of basolateral transporters in the intestinal absorption of compounds is less studied compared to transporters on the apical membrane of enterocytes.

Drug-drug interactions (DDIs) associated with intestinal transporters are well documented. Clinical evidence shows that inhibition or induction of the transporters in the intestine, including P-gp, BCRP, and organic anion transporting polypeptide (OATP) 2B1, can cause significant changes in the plasma profile for their substrates (Rengelshausen et al., 2003; Yamasaki et al., 2008; Keskitalo et al., 2009; Imanaga et al., 2011; Hartter et al., 2013; Shirasaka et al., 2013b). For example, digoxin, a well-known P-gp clinical probe substrate (Shi et al., 2011), used to treat congestive heart failure, had significantly increased serum exposure when taken orally with clarithromycin, an antibiotic that strongly inhibits P-gp (Rengelshausen et al., 2003). Another P-gp substrate, dabigatran etexilate, is a prodrug of dabigatran, which is used as an anticoagulant. Plasma concentrations of dabigatran increase when dabigatran etexilate is administered with verapamil, a strong P-gp inhibitor used as an antihypertensive (Hartter et al., 2013). Similar to P-gp, BCRP, an efflux transporter expressed on the apical membrane of the enterocytes, pumps the xenobiotics back to the intestinal lumen (Murakami and Takano, 2008). The change of BCRP functionality by genetic polymorphism, e. g, lower expression by mutant allele, change the plasma exposure of the drugs (Yamasaki et al., 2008; Keskitalo et al., 2009). For example, rosuvastatin, an HMG-CoA reductase inhibitor, is commonly used as a clinical BCRP substrate. Its exposure increased about 2.4-fold in subjects carrying the 421A/A variant, a less functional homozygous mutant of BCRP, compared to those with the wild-type homozygote (421 C/C) (Keskitalo et al., 2009). Sulfasalazine, used to treat inflammatory bowel disease, is also a well-known BCRP substrate, and its exposure in subjects with homozygous mutant BCRP was approximately 3.5 times higher than in those with the wild-type homozygote (Yamasaki et al., 2008). As a result, the International Council for Harmonization (ICH) published guidelines to recommend assessing the P-gp and BCRP DDIs during drug development (Zamek-Gliszczynski et al., 2018). On the other hand, while it is not in the ICH guidance, OATP2B1 is expressed in the enterocytes and reportedly affects the absorption of its substrate drugs (Shirasaka et al., 2013b). For example, the absorption of fexofenadine, an OATP2B1 substrate, is reduced in subjects carrying the OATP2B1 c.1457C>T allele and is also decreased when co-administered with fruit juice due to inhibition of OATP2B1 in the intestine (Imanaga et al., 2011). OATP2B1 is traditionally considered to be expressed on the apical side; however, its localization in the intestine has recently been reported on the basolateral membrane of enterocytes (Keiser et al., 2017; Zamek-Gliszczynski et al., 2018).

The serum concentration of talinolol, a β1-adrenergic receptor antagonist used to treat hypertension, showed two distinct peaks during the absorption phase, with the first and second peaks appearing at 1 and 4 h after oral administration of a hard capsule in healthy young volunteers (Weitschies et al., 2005). The phenomenon was observed in a separate clinical study reported previously by Westphal et al. (2000a). Similarly, several drugs, such as bedaquiline, irbesartan, and amisulpride, exhibit dual or multiple peaks during the absorption phase (Westphal et al., 2000a; Weitschies et al., 2005; McLeay et al., 2014; Karatza and Karalis, 2020; Kousovista et al., 2023). Talinolol is a substrate for both OATP2B1 (intestinal uptake) and P-gp (intestinal efflux), and its bioavailability is influenced by the interplay of transporters and their regional expression in the gastrointestinal (GI) tract. DDIs may occur with inhibitors of these transporters, but clinical outcomes depend on which is mainly affected: uptake or efflux. Some potent inhibitors can lower talinolol absorption by selectively blocking OATP2B1 rather than P-gp efflux. On the other hand, P-gp inhibitors like verapamil or surfactants (D-α-tocopheryl polyethylene glucol succinate (TPGS)) can increase talinolol’s absorption by blocking P-gp efflux (Bogman et al., 2005). Conversely, verapamil can surprisingly decrease talinolol’s bioavailability in some cases (Schwarz et al., 1999), likely because it mainly inhibits OATP2B1-mediated uptake rather than P-gp-mediated efflux, resulting in reduced overall absorption (Lee et al., 2011). Efforts have been made to develop pharmacokinetic (PK) models that describe these dual absorption peaks. For example, Weitschies et al. created a three-compartment PK model to determine whether the talinolol double-peak is related to food absorption processes (Weitschies et al., 2005). Through the model analysis, the authors demonstrated that adding a presystemic storage compartment to the three-compartment systemic PK model effectively captures the double absorption peaks, reflecting the complex interactions of uptake and efflux transport processes along the GI tract (Weitschies et al., 2005). However, the virtual presystemic compartment in the model is not considered physiologically relevant. The rapid dissolution of talinolol at pH 1.2 and 6.8 also excludes the possibility of dissolution-related dual-peak absorption (Kazi et al., 2019). Similarly, in a population PK model of bedaquiline, an antituberculosis medication, was developed to explain the dual absorption peaks seen in Phase I and II studies in healthy subjects and tuberculosis patients (McLeay et al., 2014). The second peak of bedaquiline absorption occurred at 5 h after dosing, which was 1 h after lunch, indicating the impact of food intake on absorption. In order to capture these absorption peaks of bedaquiline, two zero-order absorption rates with separate lag times were incorporated into a 4-compartment disposition model (McLeay et al., 2014). Irbesartan, an angiotensin II type 1 receptor antagonist, showed dual absorption peaks in plasma concentration-time profiles of healthy male volunteers (Karatza and Karalis, 2020). A two-compartment population PK model with a constant delay in the first-order absorption rate constant was developed to capture the dual peaks of the drug absorption (Karatza and Karalis, 2020). In addition, Kousovista et al. (2023) used the first-order absorption followed by a first-order absorption with lag time in a population pharmacokinetic model to describe the dual absorption peaks in the plasma concentration-time profiles of amisulpride, a dopamine D_2_ receptor antagonist used for neuropsychiatric treatment (Kousovista et al., 2023). While these aforementioned modeling approaches can effectively describe the dual peaks of the drugs in plasma, they are empirical, and potential mechanisms underlying the absorption peaks, such as transporter regional expression along the GI track and interplay on apical and basal membranes of enterocytes, remain unanalyzed.

**FIGURE 1:**
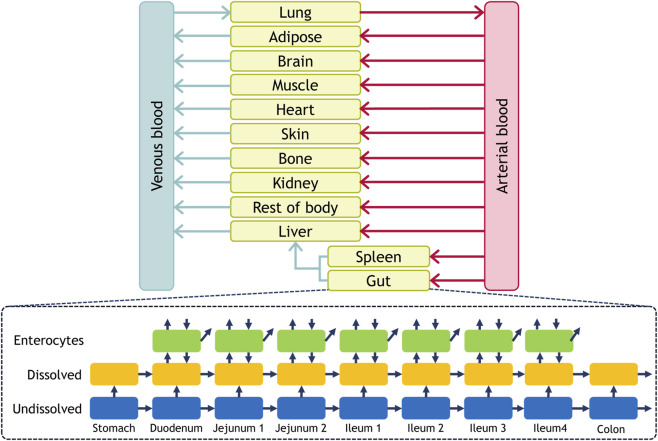
Model structure of the Full PBPK with M-ADAM Model. The full PBPK model was built in SimBiology version R2025a (MathWorks).

In the present study, we hypothesized that the mechanism of dual peaks in plasma is due to transporter activity and/or expression difference between intestinal segments, such as P-gp abundance difference between segments and their interplays (Drozdzik et al., 2019). Using talinolol as a model drug, which is a P-gp substrate and has dual absorption peaks in its PK curves, a full physiologically-based pharmacokinetic (PBPK) model that includes a multi-layer gut wall within an advanced dissolution, absorption, and metabolism (M-ADAM) model (the schematic are shown in Figure 1) was utilized to explain transporter interplays along the GI tract for the dual absorption peaks of talinolol.

## 2 Materials and methods

### 2.1 Materials

All chemicals used in the experiments were purchased from Sigma-Aldrich, Inc. (Atlanta, GA). Pre-plated Caco-2 cells were purchased from Sigma-Aldrich and Discovery Life Sciences (Huntsville, AL), respectively.

### 2.2 Transcellular permeability study of talinolol and digoxin in Caco-2 cells

#### 2.2.1 Assay conditions for bidirectional permeability assessment

Caco-2 cells monolayers were grown to confluence on collagen-coated, microporous, polycarbonate membranes in 24-well transwell plates for 21 days. The permeability assay buffer in donor wells was Hanks’ balanced salt solution (HBSS) containing 15 mM glucose, 10 mM HEPES and 0.1% BSA at pH 5.0-7.4. The receiver wells used HBSS buffer containing 15 mM glucose and 10 mM HEPES supplemented with 1% BSA at pH 7.4. After an initial equilibration with the HBSS buffers, TEER values were taken to test the integrity of cell monolayers. The experiment was started by adding buffers containing test compounds, 200 μL and 1,000 µL in the apical and basolateral chambers, respectively. At 0-, 60-, and 120-min post-dose, 10 µL was sampled from the donor compartment and diluted in 190 µL of 20% methanol. At 60- and 120-min post-dose, 100 µL samples were taken from the receiver compartments and were immediately diluted in 100 µL of 20% methanol. The removed buffer was replaced with fresh buffers, and a correction was applied to all calculations for the removed material. Each compound was tested in 2 separate replicate wells for each condition. All samples were extracted with 400 µL 100% acetonitrile with an internal standard (100 nM labetalol) to precipitate protein. Compounds were dosed (0.3–300 μM for talinolol and 0.3–100 μM for digoxin, final dimethyl sulfoxide concentration: 0.2% (v/v)) on the apical or basolateral side to determine the permeability of both directions. To test for non-specific binding and compound instability, the total amount of the drug was quantified at the end of the experiment and compared to the material present in the original dosing solution as a percent recovery. To evaluate P-gp-mediated efflux of the compounds, the permeability assay was also conducted in the presence of 30 μM elacridar, a potent P-gp inhibitor. The inhibitor was added to both the donor and receiver sides. After permeability assay, the permeability of lucifer yellow (concentration) was conducted to confirm the membrane integrity (data not shown). Samples were analyzed by LC-MS/MS (see below). The buffer samples were stored at −80 °C until analysis.

The apparent permeability (P_app_) was calculated as follows:

$$P_{app}=\frac{dR}{dt}\cdot \frac{V_{r}}{\left(A\cdot D_{0}\right)}$$ (1)

where **dR/dt** is the slope of the cumulative concentration in the receiver compartment versus time based on receiver concentrations measured at 60 and 120 min. **V**_**r**_ is the receiver compartment volumes. **A** is the area of the cell monolayer (0.33 cm^2^), and **D**_**0**_ is the measured donor concentration at the beginning of experiments.

#### 2.2.2 Membrane transport kinetics estimation

**FIGURE 2:**
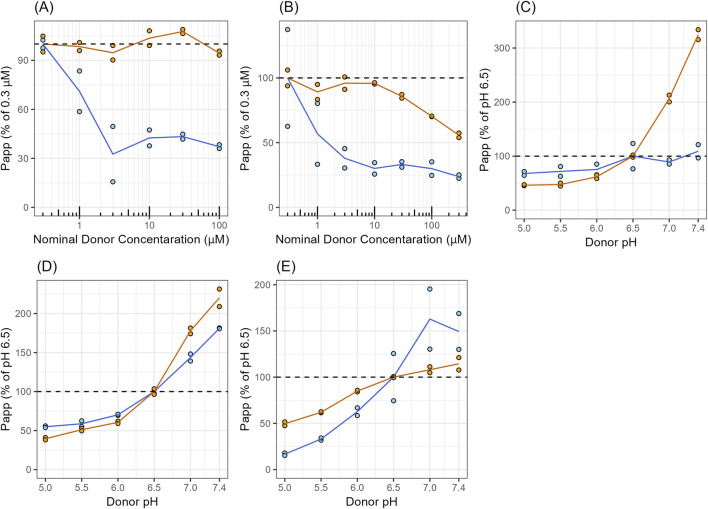
Concentration- and pH-Dependent Permeability of Digoxin and Talinolol in Caco-2 Cells **(A)** Concentration-dependent P_app_ of digoxin. **(B,C)** concentration- and pH-dependent P_app_ of talinolol in Caco-2, respectively. **(D,E)** pH-dependent P_app_ of propranolol and vinblastine, respectively. The closed circles represent observed P_app_ in duplicates. The solid lines represent the mean value from duplicates. Blue and orange represent P_app,AB_ and P_app,BA_, respectively.

A 3-compartment model (apical, cell, and basolateral compartments) using SimBiology (version R2025a, MathWorks) was developed to estimate the membrane transport kinetics of compounds in Caco-2 cells. The diagram of this analysis is shown in Figure 2, and the model report file generated by SimBiology is included in the Supplementary Material. In brief, the 3-compartment model analysis (apical, cell, and basolateral compartments) was employed to estimate membrane transport clearances. The following differential equations were used to estimate membrane transport clearances and transport kinetic parameters:

dXAdt=−6.5pHAαAC·CLAC+funionizedA·CLdiff·XAVA+pHA6.5αCA·VmaxCAKmCA+XCVC+funionizedC·fucell·CLdiff·XCVC (2)

dXBdt=−pHB6.5αBC·CLBC+funionizedB·CLdiff·XBVB+pHB6.5αCB·VmaxCBKmCB+XBVB+funionizedC·fucell·CLdiff·XCVC (3)

dXCdt=6.5pHAαAC·CLAC+funionizedA·CLdiff·XAVA−pHA6.5αCA·VmaxCAKmCA+XCVC+funionizedC·fucell·CLdiff·XCVC+pHB6.5αBC·CLBC+funionizedB·CLdiff·XBVB+pHB6.5αCB·VmaxCBKmCB+XBVB+funionizedC·fucell·CLdiff·XCVC (4)

where **X**_**A**_, **X**_**B**_, and **X**_**C**_ are the amount of the compound in the apical chamber, the basolateral chamber, and the cells at time t, respectively. **pH**_**A**_ and **pH**_**B**_ are the pH in the apical and basolateral chambers, respectively. $f_{unionized}^{A}$ , $f_{unionized}^{C}$ , and $f_{unionized}^{B}$ are the unionized fraction of the compound at apical, cell and basolateral compartment, respectively, calculated by Henderson-Hasselbalch equation. **V**_**A**_ and **V**_**B**_ are the volumes of the apical chamber (200 µL), the basolateral chamber (1,000 µL), respectively. **V**_**C**_ is the volume of the cells (0.908 μL, (Sun and Pang, 2008), assuming the cellular volume was proportionally increased with surface area). **CL**_**AC**_ and **CL**_**BC**_ are the uptake clearance on the apical and basolateral membrane, respectively. **CL**_**diff**_ represents passive diffusion, and only the unionized compound was assumed to penetrate the plasma membrane of Caco-2 cells. Additionally, pH-dependent change of the membrane transport activity was evaluated. The exponents of the pH ratio (**α** values in Equations 2–4) were estimated simultaneously.

### 2.3 Liquid chromatography-tandem mass spectroscopy (LC-MS/MS) analysis

200 μL of Acetonitrile:Water (70: 30) containing internal standard, labetalol, was added to the wells for compound extraction. The samples were shaken on the shaker for 20 min. The entirety of the extraction buffer was then transferred to a new 96-well plate and centrifuged at 3,000 rpm for 10 min at 4 °C. Standard curves for quantitation were prepared in blank cell or vesicle pellets that were treated similarly to extracted samples. 150 μL aliquot was transferred into 96 deep-well plates and then completely dried. The samples were reconstituted in 200 µL buffer containing 20% acetonitrile and 80% water with 0.1% formic acid. The samples were vortexed and centrifuged at 3,000 rpm at 4 °C for 20 min before LC-MS/MS analysis.

All the samples were analyzed on a Sciex Qtrap 6,500 or 7500 LC-MS/MS (Redwood City, CA) coupled with a Shimadzu Nexera-X2 ultra-high-performance liquid chromatograph and a Shimazu HPLC Autosampler SIL-40 (Shimadzu Corporation, Kyoto, Japan). Ten microliters of the sample were injected onto a Waters Acquity UPLC BEH C18 column (1.7 mm, 2.1 × 50 mm) (Milford, MA) and eluted by gradient mobile phases. The column temperature was set at 40 °C. The LC-MS/MS conditions for each compound are summarized in Supplementary Table S1. Additionally, a representative chromatogram of analytes and internal standard are shown in Supplementary Figure S1.

### 2.4 PBPK model analysis

A comprehensive PBPK model with M-ADAM functions was developed using SimBiology (version R2025a, MathWorks) to explain the influence of gut transporters on the PK oscillations during talinolol absorption. The schematic diagram of the PBPK model is shown in Figure 1, and the detailed model structure along with the differential equations are provided in the model report files included in the Supplementary Material. The observed human serum or plasma concentration profiles of digoxin, clarithromycin, and talinolol were obtained from literature (Kramer et al., 1979; Oosterhuis et al., 1991; Greiner et al., 1999; Ragueneau et al., 1999; Schwarz et al., 2000; Westphal et al., 2000a; Westphal et al., 2000b; Becquemont et al., 2001; Rengelshausen et al., 2003; Verstuyft et al., 2003; Weitschies et al., 2005; Gurley et al., 2006; Gurley et al., 2007; Schwarz et al., 2007; Gurley et al., 2008; Eckermann et al., 2012). To assess the effect of gut P-gp on absorption, literature DDI data between digoxin (the object) and clarithromycin (the precipitant) were used to validate the model. The physicochemical properties of the compounds were obtained from literature, and the input parameters for digoxin and clarithromycin are listed in Supplementary Table S2. The passive diffusion clearance and transporter-mediated clearance of digoxin were estimated from Caco-2 permeability assays. The kinetic parameters of the efflux transporter on the basolateral membrane of enterocytes were optimized to capture the plasma or serum concentration profile of digoxin after oral administration (see Figure 4B). The distribution, metabolism, and elimination data of the compounds were obtained from literature (see Supplementary Table S2). The passive diffusion of clarithromycin in the gut was estimated by fitting to capture the plasma or serum concentration profiles of clarithromycin after oral administration (see Supplementary Figure S2). All other parameters for clarithromycin were obtained from literature (see Supplementary Table S2).

**FIGURE 3:**
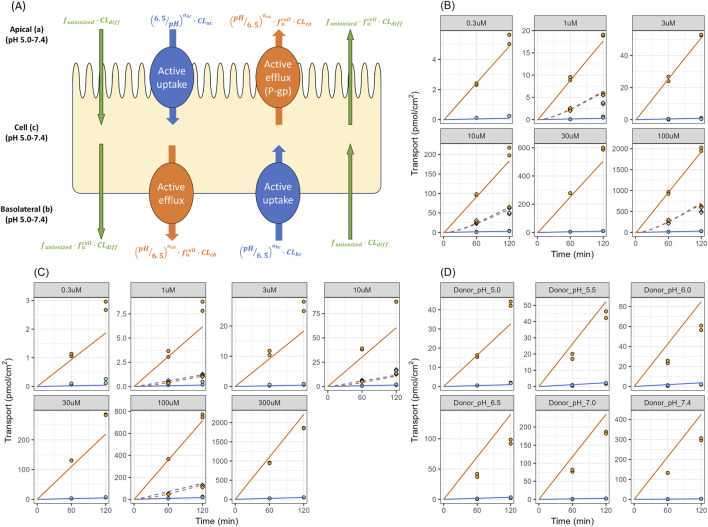
Membrane Transport of Talinolol and Digoxin in Caco-2 Cells **(A)** Schematic of membrane transport clearance estimation in Caco-2 cells. **(B)** Transcellular transport of digoxin (donor pH 6.5, receiver pH 7.4) at various concentrations in Caco-2 cells. **(C)** Transcellular transport of talinolol (donor pH 6.5 and receiver pH 7.4) at various concentrations in Caco-2 cells. **(D)** Transcellular transport of 10 μM talinolol at different donor pH in Caco-2 cells. The blue and orange solid lines in panels **(B–D)** represent the predicted transport of the drug in the absence of P-gp inhibitor (30 μM elacridar) in apical to basolateral and basolateral to apical direction, respectively. The blue and orange dashed lines in these panels represent the predicted transport of the drug in the presence of a P-gp inhibitor in the apical to basolateral and basolateral to apical direction, respectively. The closed circles represent the individual observed data in duplicates.

After validating gut P-gp function in the SimBiology model using DDI data between digoxin and clarithromycin, the talinolol model profile was created within the same model structure as the digoxin model (see the model report file in the Supplementary Material). Similar to the digoxin model, the passive diffusion and transporter-mediated kinetics were estimated from the Caco-2 permeability assay. The physicochemical properties, distribution, metabolism, and elimination data were obtained from literature, and input parameters are listed in Supplementary Table S2. The detailed model information is listed in the model report files, which are included in the Supplementary Material. The active uptake of talinolol on the apical membrane of Caco-2 cells was negligible (see Figure 3; Table 1), and the simulated plasma concentration profile of talinolol without active apical uptake did not capture the observed data (see Figure 5B). Therefore, the active uptake clearance of talinolol on the apical membrane of the enterocytes was estimated by fitting.

**TABLE 1:** Membrane transport clearance of digoxin and talinolol in Caco-2 cells.

Parameter	Estimate (mean ± SE)	
Compound	Digoxin	Talinolol
CL_diff_ (μL/min/cm^2^)	0.162 ± 0.000323	9.44 ± 0.164
CL_AC_ at pH 6.5 (μL/min/cm^2^)	0 (fixed)	0 (fixed)
CL_BC_ at pH 6.5 (μL/min/cm^2^)	0 (fixed)	0.0391 ± 0.000844
Vmax_CA_ at pH 6.5 (pmol/min/cm^2^)	22.1 ± 0.232	11.4 ± 0.0398
Km_CA_ (μM)	2.43 ± 0.00279	0.567 ± 0.0106
Vmax_CB_ at pH 6.5 (pmol/min/cm2)	0.00305 ± 0.000654	8.10 ± 0.205
Km_CB_ (μM)	658 ± 100	48.2 ± 1.34
α_AC_	NA	NA
α_CA_	NA	19.3 ± 0.0261
α_BC_	NA	4.65 ± 0.328
α_CB_	NA	20.1 ± 0.240
CL_BC_ at pH 7.4 (μL/min/cm^2^)^a^	NA	0.0715
Fold-difference of Vmax_CA_ at lower intestine (pH ∼7.0) compared to upper intestine (pH ∼6.5)^b^	NA	4.18
Vmax_CB_ at pH 7.4 (pmol/min/cm^2^)^c^	NA	110

NA, not applicable, SE, standard error.

^a^ CL_BC_ at pH 7.4 was calculated by ${CL}_{BC}^{pH\;7.4}={CL}_{BC}^{pH\;6.5}\times {\left(7.4/6.5\right)}^{\alpha_{BC}}$ .

^b^ Fold-difference of Vmax_CA_ was calculated by ${\left(7.0/6.5\right)}^{\alpha_{CA}}$ .

^c^ Vmax_CB_ at pH 7.4 was calculated by ${Vmax}_{CB}^{pH\;7.4}={Vmax}_{CB}^{pH\;6.5}\times {\left(7.4/6.5\right)}^{\alpha_{CB}}$ .

The sensitivity analysis of intestinal transporter function was also conducted to identify potential mechanisms attributed to the dual absorption peaks observed in talinolol plasma profiles in humans. Since the efflux of talinolol in the gut is mainly mediated by P-gp (Kosa et al., 2018), the efflux clearance of the drug on the apical membrane of the enterocytes was fixed.

## 3 Results

### 3.1 pH- and concentration-dependent permeability of digoxin and talinolol in Caco-2 cells

The P_app_ in the apical to basolateral direction (P_app,AB_, calculated using Equation 1) of digoxin at 0.3 μM (0.37 × 10^−6^ cm/s) was significantly lower than P_app_ in the basolateral to apical direction (P_app,BA_, calculated using Equation 1) of the drug (8.60 × 10^−6^ cm/s) (Figure 2A), resulting in an efflux ratio of 23.2. In addition, P_app_ of digoxin, especially P_app,AB_, decreased with increasing the donor concentration of the drug (Figure 2A), demonstrating concentration-dependent or saturated efflux. Similar to digoxin, P_app,AB_ of talinolol at 0.3 μM (0.25 × 10^−6^ cm/s) was also significantly lower than P_app,BA_ of the drug (3.81 × 10^−6^ cm/s) (Figure 2B), resulting in an efflux ratio of 15.2. Both P_app,AB_ and P_app,BA_ of talinolol decreased with increasing donor drug concentration (Figure 2B). Talinolol remains stable in the buffer at various pH levels (Sinha and Ghai, 2011). The P_app_ of talinolol was increased by increasing donor pH; the magnitude of P_app,BA_ increase was greater than that of P_app,AB_ (Figure 2C), suggesting that talinolol transport in Caco-2 cells, including basolateral uptake and apical efflux, is affected by extracellular pH. P_app_ of propranolol, a passive diffusion marker (not active transport), was also increased with increasing donor pH; however, the magnitude of the increase was the same between P_app,AB_ and P_app,BA_ (Figure 2D). The P_app_ of vinblastine, a P-gp substrate, was also affected by donor pH, and the magnitude of P_app,BA_ increase was less than that of P_app,AB_ (Figure 2E).

To estimate the passive diffusion, uptake, and efflux transport kinetics of talinolol through either the apical or basolateral membrane in Caco-2 cells simultaneously, a 3-compartment model was used. As shown in Table 1, the Michaelis-Menten constant (K_m_) and the maximum velocity (V_max_) of P-gp-mediated digoxin efflux on the apical membrane (K_mCA_ and V_maxCA_, respectively) were estimated to be 2.43 μM and 22.1 pmol/min/cm^2^, respectively (Table 1; Figure 3B). The K_mCA_ and V_maxCA_ of talinolol were estimated to be 0.567 μM and 11.4 pmol/min/cm^2^, respectively (Table 1; Figures 3C,D). No active uptake processes on the apical membrane were modeled for both talinolol and digoxin in Caco-2 cells. Compared to the apical efflux, the affinity of the basolateral efflux of digoxin was lower, with a K_mCB_ of 658 μM and a V_maxCB_ of 0.00305 pmol/min/cm^2^. The model fitting yielded a smaller active uptake on the basolateral membrane of Caco-2 cells for talinolol, with uptake clearance (CL_BC_) of 0.0391 μL/min/cm^2^. Incorporation of the basolateral efflux kinetics fitted better to the experimental data, and the estimated K_mCB_ and V_maxCB_ of talinolol were 48.2 μM and 8.10 pmol/min/cm^2^, respectively. pH-dependent talinolol transport in Caco-2 cells was further investigated. Changes in extracellular pH in Caco-2 incubations showed an impact on talinolol transport across Caco-2 cells (Figure 3D). The apical efflux modeled by the 3-compartment model increased by 4.18-fold at pH 7.0 compared to pH 6.5 (Table 1). To incorporate transporter kinetics values on the basolateral membrane, the CL_BC_ and V_maxCB_ at pH 7.4 (pH in whole blood) were calculated using α values (Table 1).

### 3.2 PBPK modeling and sensitivity analysis

**FIGURE 4:**
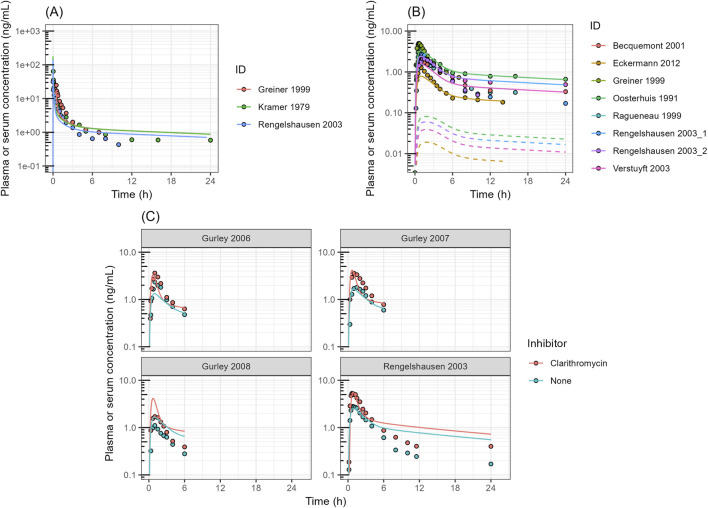
Simulated Versus Observed Serum or Plasma Profiles of Digoxin in Human **(A)** Digoxin plasma or serum concentration after intravenous injection. **(B)** Digoxin plasma or serum concentration after oral administration. **(C)** Effect of clarithromycin on the digoxin plasma or serum profiles in humans. Closed circles represent the observed data obtained from literature; The dashed lines represent the simulated digoxin concentration without any refinement. The solid lines represent the predicted digoxin profile with estimating V_maxCB_ of the drug. The V_maxCB_ was estimated to be 6,470 ± 1940 pmol/min/cm^2^ (mean ± SE).

A full PBPK with M-ADAM model was built in SimBiology version R2025a. To validate intestinal P-gp function in this model, digoxin plasma or serum profiles in healthy volunteers were simulated (Figure 4). The input parameters of digoxin are listed in Supplementary Table S2. The absorption kinetics parameters, including passive permeability and transporter kinetics, were estimated from the results of the Caco-2 permeability assay (Table 1), and incorporated into the model. The simulated plasma or serum profiles of digoxin after intravenous (IV) administration reasonably captured the observed data reported in the literature (Figure 4A). However, the plasma or serum profile of the drug after oral administration was underestimated (Figure 4B). Since uptake transporters expressed in the intestine do not transport digoxin, including OATP2B1 and OCT1 (Kimoto et al., 2011; Taub et al., 2011), the active uptake clearance of digoxin was kept as 0. To capture the serum or plasma profiles of digoxin, the *in vivo* V_maxCB_ of the drug was estimated by fitting, and the V_maxCB_ was estimated to be 6,470 pmol/min/cm^2^. To validate this refined digoxin model, the DDI data between digoxin and P-gp inhibitor clarithromycin were simulated. The clarithromycin PK profiles were also modeled in a PBPK model developed separately (Supplementary Figure S2). As shown in Figure 4C and Table 2, the plasma or serum profiles of digoxin and its AUC and C_max_ change by clarithromycin were accurately captured, which confirmed an adequate role of P-gp in digoxin absorption and validated the model structure.

**TABLE 2:** Observed and predicted AUC and C_max_ ratio of digoxin in the absence and presence of clarithromycin.

Group		AUC_0–3h_ (ng/mL·h)			C_max_ (ng/mL)		
Treatment		None	+CLA	Ratio	None	+CLA	Ratio
Gurley et al. (2006)	Obs	4.5	6.3	1.5	2.9	4.3	1.5
	Pred	3.0	5.0	1.7	1.3	3.3	2.5
Gurley et al. (2007)	Obs	4.0	7.3	1.8	2.1	4.1	2.0
	Pred	3.8	6.3	1.7	1.7	4.1	2.4
Gurley et al. (2008)	Obs	2.2	3.7	1.7	1.2	2.1	1.8
	Pred	3.7	6.3	1.7	1.7	4.1	2.4
Rengelshausen et al. (2003)	Obs	6.1	11	1.8	3.6	6.6	1.8
	Pred	5.7	8.6	1.5	2.6	5.6	2.2

+CLA, with clarithromycin; Obs, observed, Pred: predicted.

Observed AUC_0–3h_ and C_max_ are the mean values obtained from literature. Predicted AUC_0–3h_ and C_max_ were calculated with simulated digoxin profile (Figure 4C).

**FIGURE 5:**
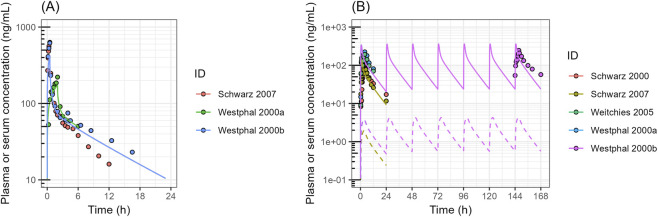
Simulated versus the Observed Plasma Concentration of Talinolol in Human **(A)** Plasma concentration of talinolol after intravenous administration. **(B)** Plasma concentration of talinolol after oral administration. Closed circles represent the observed data obtained from the literature. The dashed lines represent the simulated talinolol plasma profile without any refinement. The solid lines represent the predicted talinolol plasma profile, with the estimated active uptake of the drug at the apical membrane of enterocytes. The clearance was estimated to be 0.838 ± 0.112 μL/min/cm^2^ (mean ± SE).

The above PBPK model, validated with digoxin data, was used to simulate talinolol plasma profiles observed in healthy volunteers (Figure 5), with the input parameters listed in Supplementary Table S2. Similar to the digoxin model, the model incorporated talinolol-specific parameters, including passive diffusion, active uptake on the basolateral membrane, and kinetic parameters of transporters that were estimated from the Caco-2 permeability assay using the 3-compartmental model (Table 1). While the simulated plasma profiles of talinolol reasonably overlapped the observed data following IV administration (Figure 5A), the simulated profiles after oral administration were significantly underpredicted (Figure 5B). The apical uptake kinetics of talinolol in the intestine were added to the model at 0.838 μL/min/cm^2^ to align the predicted plasma profile with the observed data (Figure 5B). However, even after including the estimated apical uptake, the model could not reproduce the dual peaks of talinolol in plasma (Figure 5B).

**FIGURE 6:**
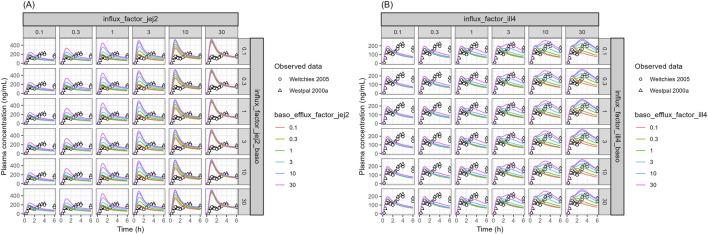
Sensitivity Analysis of Intestinal Transport for Talinolol PK **(A)** Effect of transport activity in the jejunum II segment. **(B)** Effect of transport activity in the ileum IV segment. influx_factor_jej2: SF of CL_AC_ in jejunum II segment, influx_factor_jej2_baso: SF of CL_BC_ in jejunum II segment, baso_efflux_factor_jej2: SF of CL_CB_ in jejunum II segment, influx_factor_ill4: SF of CL_AC_ in ileum IV segment, influx_factor_ill4_baso: SF of CL_BC_ in ileum IV segment, baso_efflux_factor_ill4: SF of CL_CB_ in ileum IV segment, SF: scaling factor. The open circles and triangles represent the observed data obtained from literature. The solid lines represent simulated talinolol plasma profiles.

**FIGURE 7:**
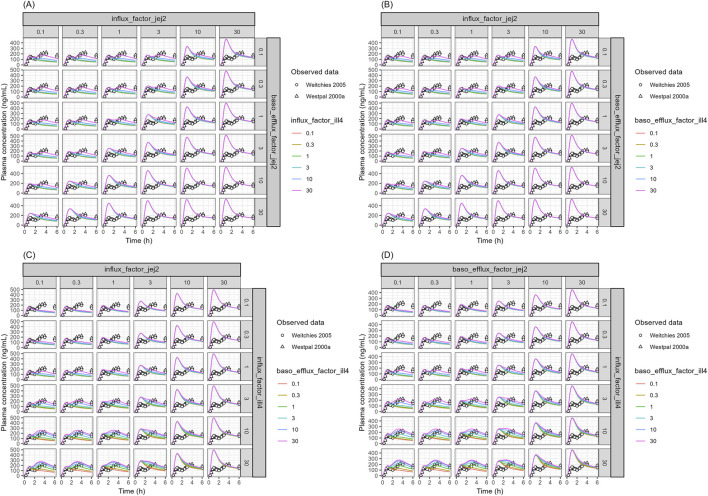
Sensitivity Analysis of CL_AC_ and CL_CB_ in the Jejunum II and the Ileum IV segments for Talinolol PK **(A)** CL_AC_ in the jejunum II, CL_CB_ in the jejunum II, and the CL_AC_ in the ileum IV. **(B)** CL_AC_ in the jejunum II, CL_CB_ in the jejunum II, and CL_CB_ in the ileum IV. **(C)** CL_AC_ in the jejunum II, CL_AC_ in the ileum IV, and CL_CB_ in the ileum IV. **(D)** CL_CB_ in the jejunum II, CL_AC_ in the ileum IV, and CL_CB_ in the ileum IV. influx_factor_jej2: SF of CL_AC_ in the jejunum II segment, baso_efflux_factor_jej2: SF of CL_CB_ in the jejunum II segment, influx_factor_ill4: SF of CL_AC_ in the ileum IV segment, baso_efflux_factor_ill4: SF of CL_CB_ in the ileum IV segment, SF: scaling factor. The open circles and triangles represent the observed data obtained from literature. The solid lines represent simulated talinolol plasma profiles.

The sensitivity analysis was further conducted to elucidate the intestinal transporters attributed to the dual peaks of oral absorption of talinolol. Since the model was developed and validated using digoxin data, where the P-gp-mediated efflux of digoxin was well-estimated using the parameters obtained from the Caco-2 permeability assay (Figure 3), intestinal P-gp acts as the primary efflux transporter for talinolol (Kosa et al., 2018). Therefore, its role in intestinal absorption was not included in the sensitivity analysis. Instead, the pH-dependent apical efflux due to the changes of luminal pH at the distal small intestine, which is higher than that in the proximal intestine, was incorporated into the talinolol PBPK model (the apical efflux transport activity in the distal intestine is about 4-fold higher than that in the proximal intestine). Further sensitivity analysis was conducted focusing on transport activities such as uptake on the apical membrane, and uptake and efflux on the basolateral membrane in a specific intestinal segment. Changing transporter activities in jejunum II or ileum IV only captures one peak of talinolol and not both simultaneously (Figure 6; Supplementary Figure S3); therefore, the second sensitivity analysis involved combining transporter activity changes simultaneously in both segments (Figure 7). The model showed that apical uptake and basolateral efflux in the distal jejunum (jejunum II in the PBPK model) mainly contributed to the first plasma peak of talinolol (Figure 6A). Conversely, apical uptake and basolateral efflux in the distal ileum (ileum IV in the PBPK model) were key factors for the second peak of talinolol absorption (Figure 6B). To replicate the dual peaks of talinolol in plasma, decreasing apical uptake, basolateral efflux, or both in jejunum II, along with increasing apical uptake, basolateral efflux, or both in ileum IV, was necessary (Figure 7). Transporter activities in other segments did not influence the dual peaks of talinolol in plasma (Supplementary Figure S3).

### 3.3 Talinolol-rifampicin DDI simulation

To validate the Talinolol PBPK model, P-gp-mediated DDI simulation (induction by rifampicin) was conducted. To optimize the magnitude of P-gp induction in the gut, serum concentration profile of digoxin with coadministration of rifampicin was used (Greiner et al., 1999). In order to match digoxin AUC_0–144h_ ratio, the abundance of P-gp in all intestinal segments (from duodenum to ileum IV) required to be increased by 1.7-fold (Supplementary Figure S4). After applying P-gp abundance increase and transporter activities changes shown by sensitivity analysis (Figure 7), the AUC_0–24h_ ratio of talinolol was captured well (Supplementary Figure S4).

## 4 Discussion

Only unionized compounds can passively cross the plasma membrane of enterocytes (Gaohua et al., 2021). Therefore, basic compounds like talinolol may undergo less passive diffusion compared to neutral or acidic compounds because of a lower unionized fraction in the intestinal lumen, where the pH is slightly below the physiological pH of 7.4. Additionally, various transporters are present in the intestine, including those involved in apical uptake and basolateral efflux, which can significantly influence drug absorption. Therefore, pH-dependent passive permeation and active efflux and uptake activities can affect drug absorption, both in bioavailability and absorption dynamics. In addition, the spatial distribution of uptake and efflux transporters along the GI tract—from the duodenum through the jejunum and ileum to the colon—constitutes a fundamental determinant of oral drug absorption. OATP2B1 exhibits relatively uniform mRNA and protein expression throughout the small intestine, whereas P-gp displays a pronounced proximal-to-distal gradient, with expression increasing markedly toward the lower intestine (Drozdzik et al., 2014). The region/localization-dependent interplay of these transport systems governs absorption dynamics, and site-specific intestinal infusion studies provide a mechanistic framework for investigating regional absorption processes. With this in mind, Mallol et al. developed a PBPK model to investigate the influence of transporters, including OATP2B1 and P-gp, on the talinolol PK (Mallol et al., 2023). The model consists of an active intestinal absorption process mediated by OATP2B1 and an active efflux mediated by P-gp, modeled through irreversible first-order Michaelis-Menten and irreversible mass-action kinetics, respectively. While the model fairly captured the overall systemic PK changes by gene polymorphisms and DDIs with P-gp inhibitors, it failed to describe the absorption dynamics of talinolol observed in humans (Mallol et al., 2023).

Talinolol showed two distinct peaks during the absorption phase after oral administration of a hard capsule in healthy young volunteers (Weitschies et al., 2005), and demonstrated linear pharmacokinetics at the dose levels from 50 to 100 mg (Figure 5). To clarify the mechanisms underlying the dual-peaks phenomenon observed in talinolol PK curves, we first conducted *in vitro* Caco-2 studies. Asymmetrical transport of talinolol between the apical to basolateral and basolateral to apical directions was observed, indicating transporter-mediated efflux in Caco-2 cells. The efflux was saturable in both apical and basolateral transport of talinolol (Figure 3). A three-compartment model was used to fit the results from Caco-2 experiments, aiming to deconvolute the membrane transport kinetics of talinolol, including efflux and uptake at both the apical and basal membranes. The passive permeability of talinolol was estimated to be 9.44 μL/min/cm^2^ using the Caco-2 permeability assay with different donor pH (Figure 3). At pH in the upper intestine (around pH 6.5), the unionized fraction of the drug calculated by the Henderson-Hasselbalch equation is 0.00112; therefore, the passive diffusion clearance of the unionized drug was 0.0106 μL/min/cm^2^. Talinolol transport across Caco-2 monolayers was also pH-dependent, demonstrating that P-gp-mediated apical efflux was increased 4.17-fold at pH 7.0 compared with pH 6.5. The P_app,BA_ was significantly increased by raising the donor pH, while the P_app,AB_ of talinolol was only slightly affected by changes in donor pH (Figure 2C). As a comparison, P_app_ of propranolol, which is passively transported across Caco-2 cells, was also increased with increasing donor pH, and the magnitude of the increase was the same between P_app,AB_ and P_app,BA_, suggesting that the changes were due to the increase of the unionized fraction of the drug (Figure 2D). P_app_ of vinblastine, a positive control of P-gp, was also pH-dependent, and the magnitude of this increase in P_app,AB_ was greater than that in P_app,BA_. These results indicated that P-gp has relatively lower activity at acidic pH, consistent with the literature reports (Varma et al., 2005; Mitra et al., 2011). For example, the permeability of quinidine, a P-gp substrate, increased by 3.6-fold when the luminal pH was changed from 4.5 to 7.4 (Varma et al., 2005), and inhibition of P-gp-mediated quinidine efflux by verapamil showed that passive permeation of quinidine decreased from 68% to 35% as pH increased from 4.5 to 7.4 (Varma et al., 2005). The efflux ratio of colchicine, a nonionizable P-gp substrate, was significantly decreased with decreasing extracellular pH in both Caco-2 and MDR1-MDCK cells (Mitra et al., 2011). A similar trend was also observed in other non-ionizable P-gp substrates such as digoxin, dexamethasone, paclitaxel, and etoposide (Mitra et al., 2011). It is worth noting that digoxin efflux was not changed between pH 5.0 and 7.4, in Caco-2 cell experiments, whereas that of colchicine was significantly changed (Mitra et al., 2011), suggesting the pH effect on P-gp activity could be substrate-dependent.

Drug-drug interactions (DDIs) have potential consequences in clinical applications. GI transporters mediate drug absorption; therefore, in theory, inhibition or induction of transporter activity can alter drug exposure (Elmeliegy et al., 2020). For example, talinolol plasma exposure was decreased by grapefruit juice (Schwarz et al., 2005), and the components of grapefruit juice inhibit OATP2B1-mediated uptake (Shirasaka et al., 2013a). Apricot extraction can increase the intestine absorption of talinolol through inhibiting P-gp efflux (Deferme et al., 2002). On the other hand, oral bioavailability of talinolol were reduced by repeating doses of St John’s wort or rifampin to induce P-gp expression in the intestine (Schwarz et al., 2007; Elmeliegy et al., 2020). These results suggest that OATP2B1, in concert with the efflux transporter P-gp, plays a key role in the intestinal absorption of talinolol. Sensitivity analysis of digoxin-rifampicin DDI revealed that P-gp expression increased about 1.7-fold along the GI tract (Supplementary Figure S4). After incorporating the P-gp induction, the model can predict the talinolol-rifampicin DDI, which clearly shows the role of P-gp in talinolol absorption. Interestingly, the apical uptake of talinolol in Caco-2 cells was found to be negligible in our three-compartment transporter model analysis (Figure 3). Traditionally, OATP2B1 was thought to be an apical transporter in the small intestine, based on limited immunohistochemical analysis of human tissue and Caco-2 cells (Kobayashi et al., 2003; Sai et al., 2006). This was believed to explain the intestinal absorption of OATP2B1 substrates like talinolol in the PBPK model by Mallol et al. (Mallol et al., 2023). In contrast, using immunohistochemical staining, it was found that OATP2B1 is actually localized to the basolateral membrane, rather than the apical membrane, which is the opposite domain of the apical membrane efflux transporter P-gp (Mooij et al., 2016; Keiser et al., 2017). Negligible apical uptake of talinolol in Caco-2 cells supports the later findings that OATP2B1 is mainly localized to the basolateral membrane. In addition, talinolol absorption is inhibited when taking grapefruit juice (Schwarz et al., 2005), suggesting that OATP2B1 may have a role in its absorption, and contribute to the talinolol GI absorption and the association of dual-peaks phenomena.

Abundance of transporter proteins in the GI tract can differ along with the intestinal segments, which can also affect drug absorption dynamics, leading to dual-peak curves in PK profiles. For instance, P-gp abundance differed between intestinal segments in both humans and animals (Drozdzik et al., 2019; Mai et al., 2021). Drozdzik et al. reported that P-gp abundance (lowest versus highest) is about 9.2-fold (duodenum versus ileum) (Drozdzik et al., 2019). Additionally, other transporters, such as peptide transporter 1 (PEPT1) and BCRP, showed differences in abundance between segments (lowest versus highest ratio: 34 (ileum versus colon) and 5.9 (ileum versus colon) for PEPT1 and BCRP, respectively) (Drozdzik et al., 2019).

The PBPK model, after incorporating passive diffusion and active transport estimated from Caco-2 cells data, the P-gp abundance in different intestinal segments, and the pH-dependent P-gp efflux, did not accurately capture talinolol plasma or serum profiles in healthy volunteers. The basolateral uptake and efflux of talinolol in Caco-2 cells were also pH-dependent; however, the *in vivo* physiological pH on the basolateral membrane of enterocytes is generally considered less fluctuating, consistent with a blood pH of about 7.4. Therefore, the pH-dependent uptake and efflux on the basolateral membrane of the enterocytes were not included in the PBPK model. Unfortunately, the model with the incorporation of segmental expression of P-gp still did not capture the observed bi-peak curves.

One of the reasons not to capture talinolol plasma PK profiles is the active uptake on the apical membrane of the enterocytes. The active uptake clearance on the apical membrane of Caco-2 cells was negligible; therefore, the active uptake on the apical membrane of the enterocytes was not incorporated into the base model. Schwarz et al. assessed the effect of grapefruit juice on the talinolol exposure in healthy volunteers (Schwarz et al., 2005) and showed that talinolol exposure was decreased by both single and multiple intakes of grapefruit juice. The reduction of the plasma talinolol exposure was similar between single and multiple grapefruit juice intakes, with no induction of P-gp expression (Schwarz et al., 2005). These results indicated that the active uptake on the apical membrane of the enterocytes is inhibited by grapefruit juice. When the apical uptake of talinolol was incorporated through model fitting, simulated plasma PK profiles of talinolol were closer to the observed data (Figure 5B); however, the dual peaks of the drug were still not captured. Sensitivity analyses were conducted to assess the contributions of transporters and/or intestinal segments to the dual peaks of talinolol. The modeling analysis showed that reducing the apical uptake and/or basal efflux in jejunum II (lower jejunum), along with increasing apical uptake and/or basal efflux in ileum IV (lower ileum), appears to be the key factor in capturing the dual peaks of talinolol in plasma (Figure 7). Adding mechanisms for P-gp efflux, OATP2B1 uptake, and pH-dependent transport did not fully explain the absorption process of talinolol, which suggests that apical uptake and basolateral efflux, likely involving unknown transporters, could play a role in talinolol absorption dynamics. Limitation applies to the current modeling analysis, because these parameters were derived empirically rather than from direct experimental measurement. Caution should be exercised when extrapolating these findings to physiological context. Our modeling analysis highlights the need for further investigation into the mechanisms behind the talinolol absorption phenomenon.

In conclusion, the findings in the present study suggest that regional differences in the extent of absorption in the intestine contribute to the complex absorption profile of talinolol. The interplay of uptake and efflux transporters in the enterocytes is one factor contributing to these absorption dynamics and is attributed to the dual peaks in talinolol PK profiles. The analysis warrants further investigation of the mechanisms underlying the absorption dynamics of talinolol.

## Data Availability

The original contributions presented in the study are included in the article/Supplementary Material, further inquiries can be directed to the corresponding author.
