# Supplementary material for: Regional absorption of talinolol mediated by intestinal transporters: insights from PBPK modeling analysis
Source: Front Pharmacol. 2026 Feb 11;17:1726481. doi: 10.3389/fphar.2026.1726481 (PMC12932482; doi:10.3389/fphar.2026.1726481)
Supplement: Supplementary file 1 [file DataSheet1.pdf]

Regional Absorption of Talinolol Mediated by Intestinal Transporters: Insights from PBPK Modeling analysis

Kazuya Ishida, Xiaomin Liang, Fulden Buyukozturk, Jia Hao, Christine Wan, Yurong Lai

Supplementary Material

Supplemental Table 1 LC-MS/MS Conditions

LC Method

Compound	Condition	Value
Digoxin	Flow rate	0.6 mL/min
	Mobile phase	A: 0.1% formic acid in water with 10 mM ammonium acetate
		B: Acetonitrile
	Gradient (B concentration, %)	0.2 min 15% B, 1.2 min 95% B, 2.15 min 10% B
Talinolol	Flow rate	0.8 mL/min
	Mobile phase	A: Water with 0.1% formic acid
		B: Acetonitrile with 0.1% formic acid
	Gradient (B concentration, %)	0.4 min 5% B, 1.4 min 50% B, 1.6 min 95% B, 2.2 min 5% B
Vinblastine Propranolol	Flow rate	0.4 mL/min
	Mobile phase	A: Water with 0.1% formic acid
		B: Acetonitrile with 0.1% formic acid
	Gradient (B concentration, %)	0.2 min 10% B, 1.2 min 99% B, 1.55 min 10% B

Mass Transitions for Compounds

Compound	ESI Mode	m/z	
Digoxin	Positive	798.3	651.3
Talinolol	Positive	364.3	100.3
Propranolol	Positive	260.2	183.2
Vinblastine	Positive	811.4	355.0
Labetalol (IS)	Positive	329.2	162.1

IS: internal standard

Supplemantal Table 2 Input Parameters of Digoxin, Clarithromycin, and Talinolol PBPK With M-ADAM Model

Compound	Digoxin		Clarithromycin		Talinolol	
Parameter	Value	Source	Value	Source	Value	Source
Physicochemical properties						
Molecular Weight (g/mol)	780.94	(Neuhoff et al., 2003)	747.97	(McFarland et al., 1997)	363.494	(Tubic et al., 2006)
Log P	1.26	Simcyp[a]	3.16	(McFarland et al., 1997)	3.466	(Tubic et al., 2006)
Compound Type	Neutral		Monotropic base		Monotropic base	
pKa	NA		8.99	(McFarland et al., 1997)	9.43	(Tubic et al., 2006)
B/P ratio	1.07	Simcyp[a]	1	Simcyp[a]	0.94	(Yamazaki et al., 2019)
fu_{plasma}	0.71	Simcyp[a]	0.18	(Yago et al., 1996)	0.45	(Yamazaki et al., 2019)
fu_{liver}	0.4263	Simcyp[b]	0.434	Simcyp[b]	0.0772	Simcyp[b]
Formulation						
Aqueous solubility at pH 7.4 (mg/mL)	NA[d]		NA[d]		1.234	(Tubic et al., 2006)
Particle size (μm)	NA[d]		NA[d]		25	(Tubic et al., 2006)
Particle density (g/mL)	NA[d]		NA[d]		1.2	(Tubic et al., 2006)
Molecular diffusion coefficient (cm^2/s)	NA[d]		NA[d]		6.53×10^{-6}	(Tubic et al., 2006)
Diffusion layer thickness (cm)	NA[d]		NA[d]		0.003	(Tubic et al., 2006)
Absorption						
CL_{AC} (μL/min/cm^2)	0	Estimated from Caco-2 assay (see Table 1)	NA		0.838	Optimized
CL_{BC} (μL/min/cm^2)	0		NA		0.0715	Estimated from Caco-2 assay (see Table 1)
Km_{CA} (μM)	2.43		NA		0.567	
$Vmax_{CA}$ (pmol/min/cm^2)	22.1		NA		11.4	
Fold-difference of $Vmax_{CA}$ compared to upper intestine	NA		NA		4.18[e]	
Km_{CB} (μM)	658		NA		48.2	
$Vmax_{CB}$ (pmol/min/cm^2)	6470	Optimized	NA		110	
CL_{diff} (μL/min/cm^2)	0.162	Estimated from Caco-2 assay (see Table 1)	103	Optimized	9.44	
Distribution						
Model	Full PBPK		Full PBPK		Full PBPK	

Tissue-plasma partition coefficient (Kp) prediction method	Optimized by Simcyp[c]	Simcyp[a]	Rodgers and Rowland	Simcyp[b]	Rodgers and Rowland	Simcyp[b]
Kp scalar	1	Simcyp[a]	0.371	Optimized[f]	1	Assumed[g]
Elimination						
CL_R (L/h)	9.66	Simcyp[a]	7.2	(Rodvold, 1999)	18.66	(Schwarz et al., 2007)
HLM CL_{int} (mL/min/mg protein)	NA		0.0193	(Yamamoto et al., 2005)	NA	
$CL_{int,bile}$ (μL/min/10^6 cells)	NA		NA		1.8	(Yamazaki et al., 2019)
DDI						
Ki for P-gp (μM)	NA		4	(Eberl et al., 2007)	NA	

NA: not applicable, FaSSIF: fasted state simulated intestinal fluid solution, HLM: human liver microsomes CL_{int}: intrinsic clearance, CLR: renal clearance, $CL_{int,bile}$: intrinsic biliary clearance.

[a]Simcyp library file (version 23).

[b]The value was calculated using physicochemical property data with Simcyp Simulator (version 23).

[c]Solubility in FaSSIF.

[d]The dissolution rate constant of the drug in the ith compartment (K_d^i) was calculated using the equation reported by Agoram et al. (Agoram et al., 2001), $\boldsymbol{K_d^i = 3\gamma \cdot \frac{Sol - C_L^i}{\rho r T}}$, where $\boldsymbol{\gamma}$ is molecular diffusion coefficient, Sol is the aqueous solubility at pH in the ith compartment, ($\boldsymbol{C_L^i}$) is the luminal concentration of the drug in ith compartment, $\boldsymbol{\rho}$ is a particle density of the drug, $\mathbf{r}$ is the effective drug particle radius, and $\mathbf{T}$ is the diffusion layer thickness. The particle size and particle density of digoxin and clarithromycin are not available. Therefore, in the current PBPK model, digoxin and clarithromycin was administered as a solution. The solution dosing of digoxin was used for previously published PBPK model (Yamazaki et al., 2019).

[e]Fold difference of $Vmax_{CA}$ was calculated by α_{CA} estimated from Caco-2 cells (see Table 1).

[c]Digoxin Kp values in adipose, muscle, and heart were optimized by Simcyp, and other tissue's Kp values were predicted using Rodgers and Rowland method.

[f]Kp scalar of clarithromycin was adjusted to capture the observed volume of distribution at steady state (1.75 L/kg, (Chu et al., 1992).

[g]The predicted volume of distribution at steady state of talinolol based on physicochemical properties of the drug (2.96 L/kg) was similar to reported values (2.4-3.3 L/kg, (Schwarz et al., 2007; Trausch et al., 1995). Therefore, Kp scalar of talinolol was assumed to be 1.

Supplemental Figure 1 Representative Chromatogram of Analytes and Internal Standard

Digoxin

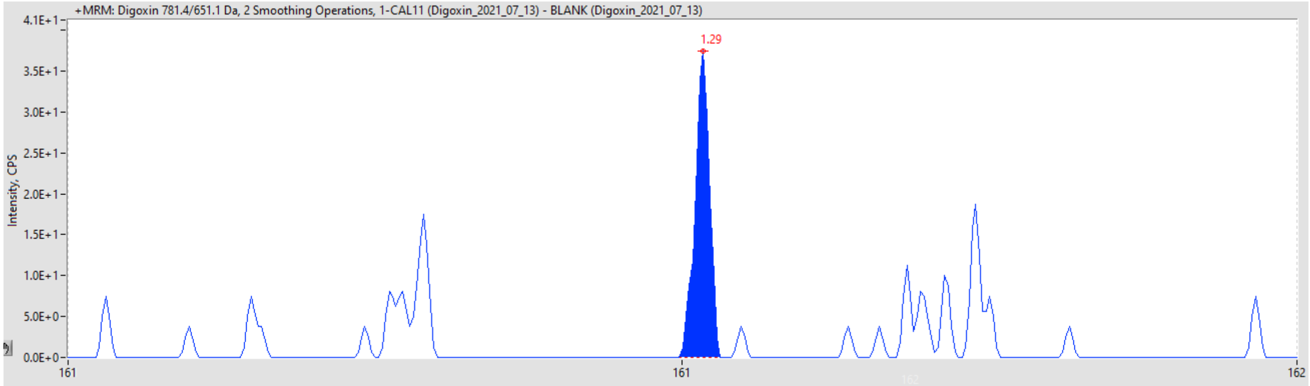

Talinolol

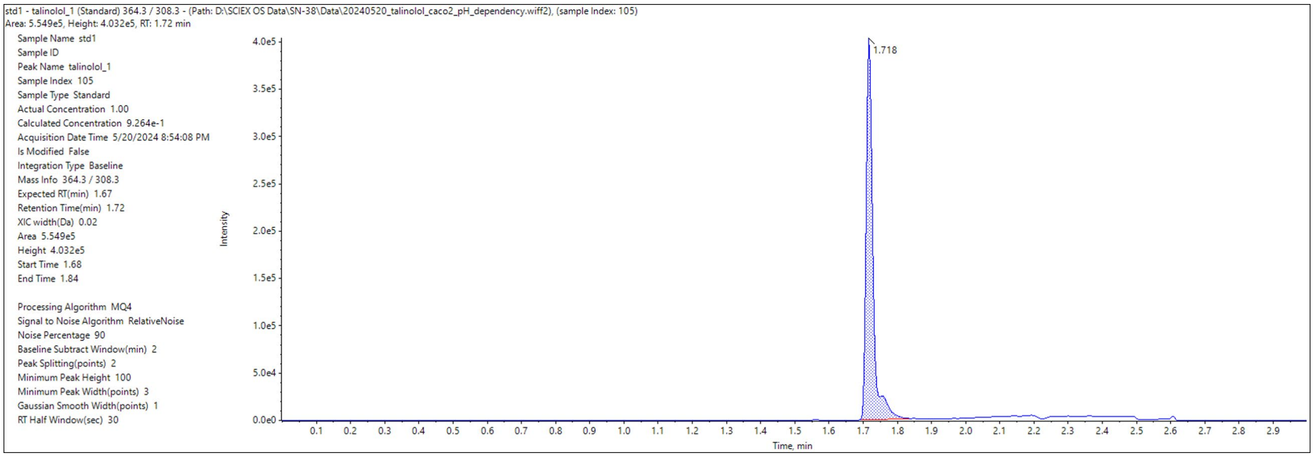

Propranolol

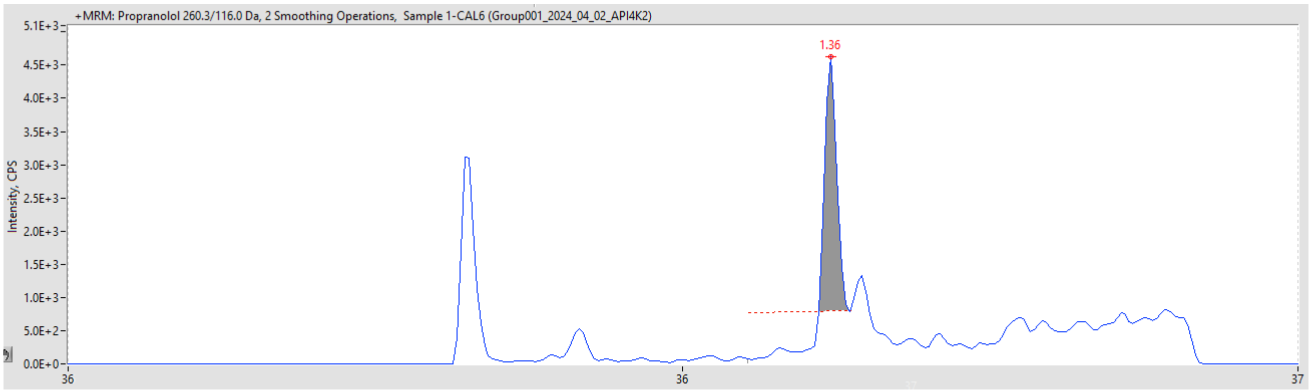

Vinblastine

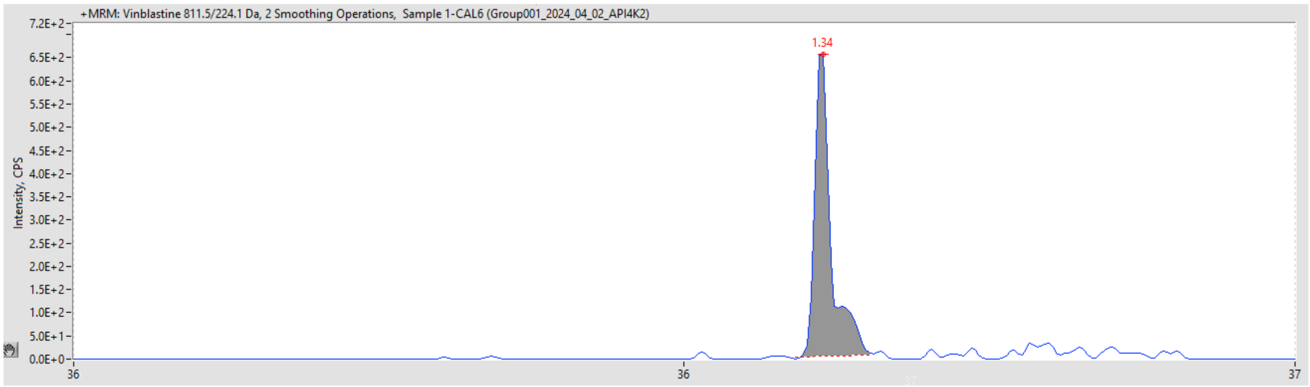

Labetalol (IS)

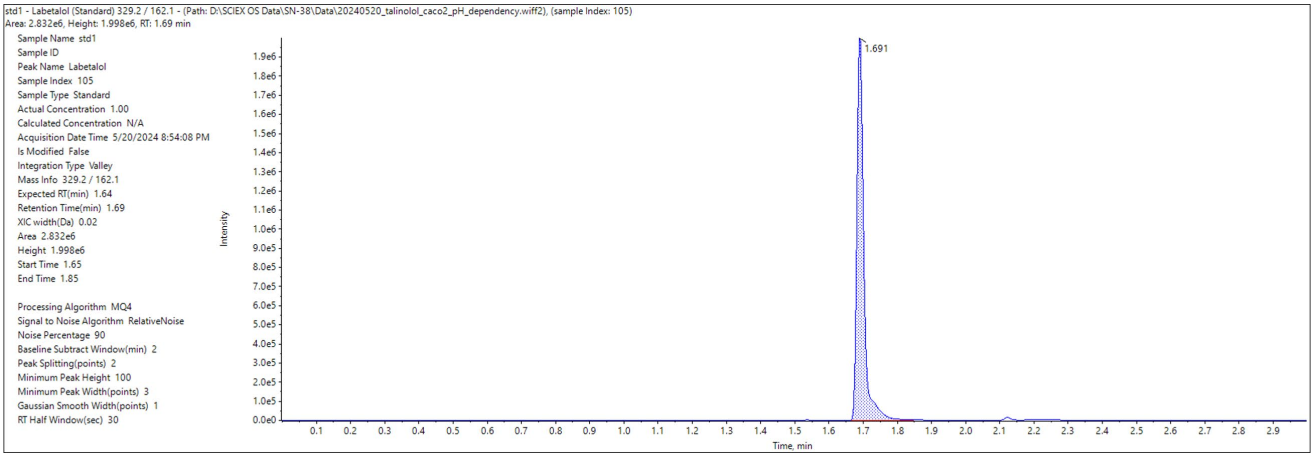

Supplemental Figure 2 Simulated Versus Observed Serum or Plasma Profiles of Clarithromycin in Human

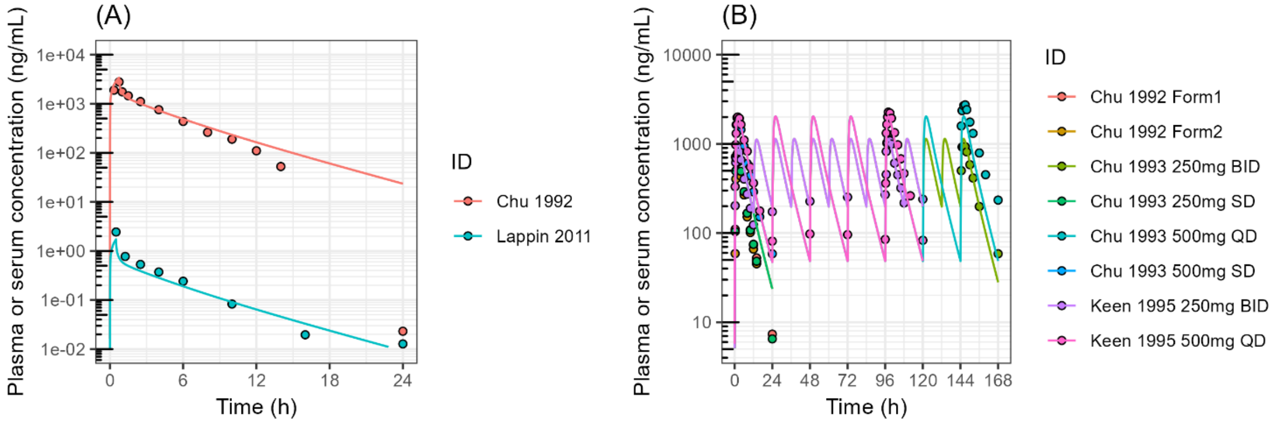

(A) Clarithromycin serum or plasma concentration after intravenous injection. The simulated curves accurately captured observed data. (B) Clarithromycin serum or plasma concentration after oral administration. The CL_{diff} was estimated to be 103 ± 26.3 μL/min/cm^2 (mean ± standard error). Closed circles represent the observed data obtained from the literature. The solid lines represent the predicted clarithromycin serum or plasma profile with the estimated CL_{diff} of the drug in the gut.

Supplemental Figure 3 Sensitivity Analysis of Intestinal Transport for Talinolol PK

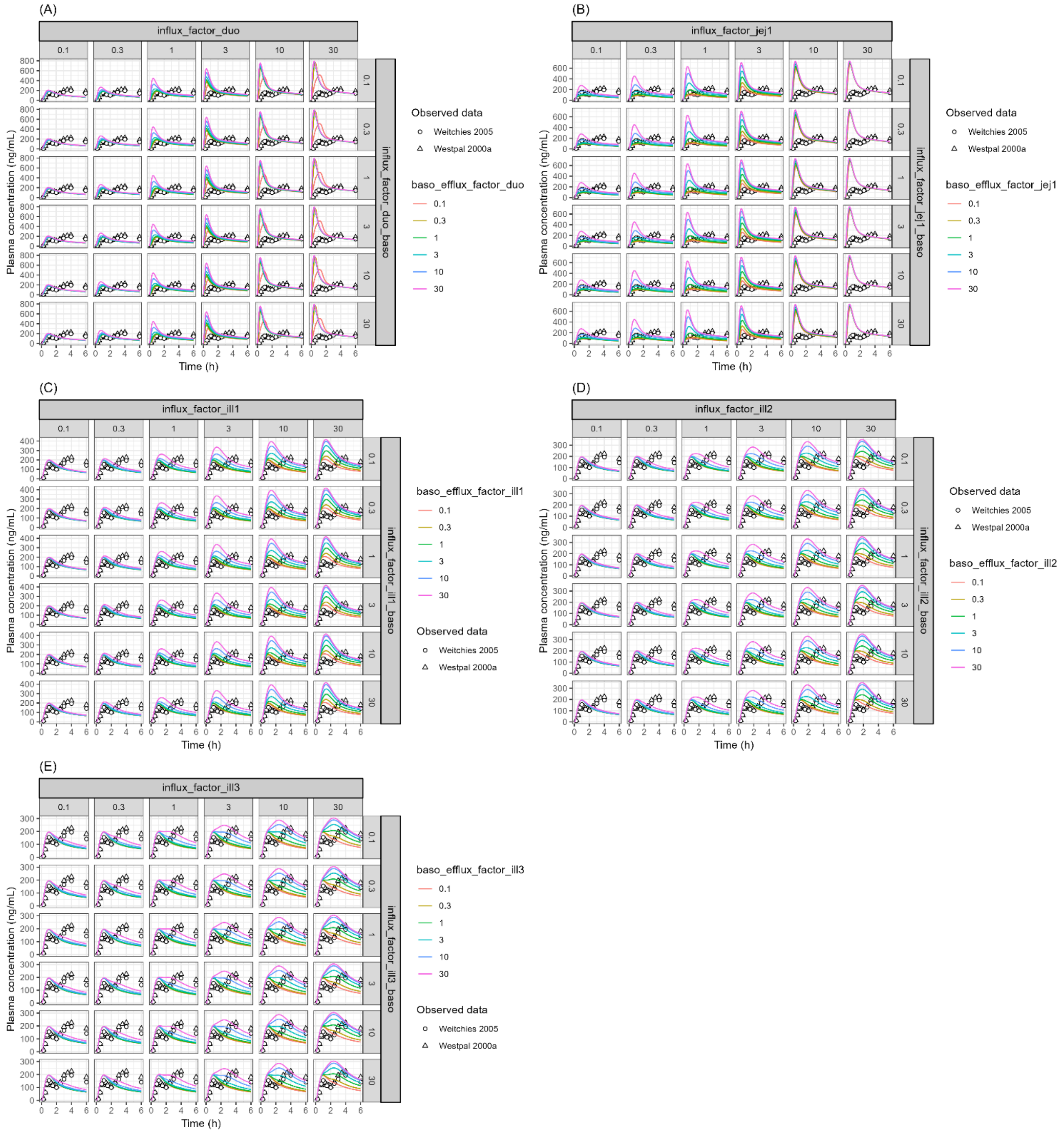

(A): Effect of transport activity in the duodenum segment. (B) Effect of transport activity in the jejunum I segment. (C): Effect of transport activity in the ileum I segment. (D): Effect of transport activity in the ileum II segment. (E): Effect of transport activity in the ileum III segment . influx_factor_segment: SF of CL_{AC} in the segment, influx_factor_segment_baso: SF of CL_{BC} in the segment, baso_efflux_factor_segment: SF of CL_{CB} in the segment, duo: duodenum, jej1: jejunum I,

ill1: ileum I, ill2 ileum II, ill3: ileum III. The open circles and triangles represent the observed data obtained from literature. The solid lines represent simulated talinolol plasma profiles.

Supplemental Figure 4 Simulated Versus Observed Serum or Plasma Profiles of Digoxin and Talinolol with Coadministration of Rifampicin in Human

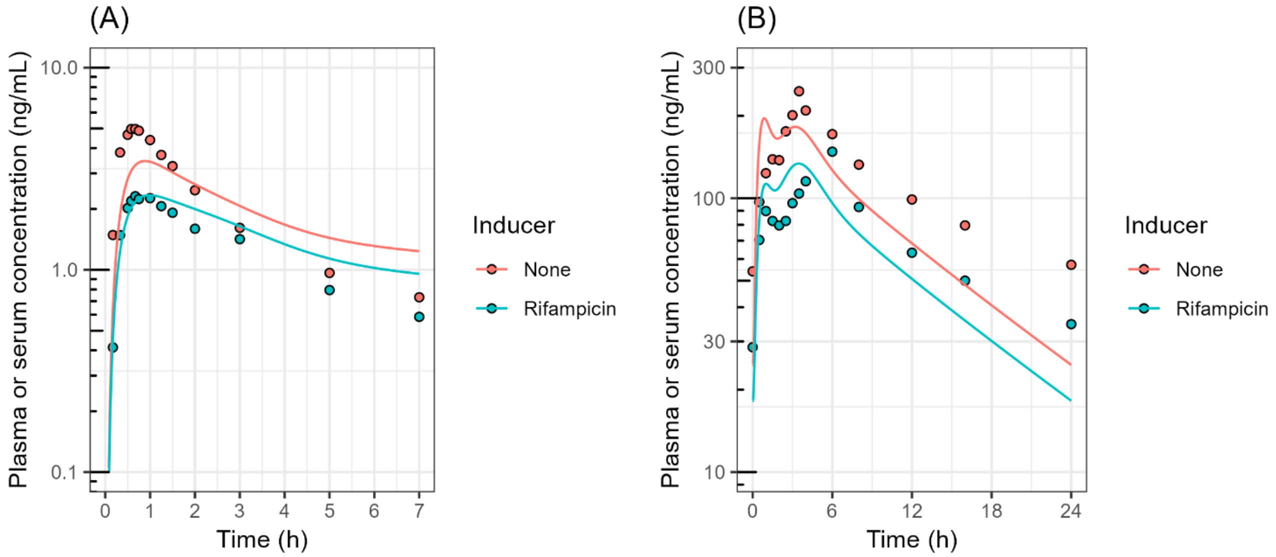

(C)

Group			AUC (ng/mL·h)		
Compound	Treatment		None	+ Rifampicin	Ratio
Digoxin	Greiner 1999	Obs	54.8	38.2	1.43
		Sim	58.1	44.3	1.31
Talinolol	Westphal 2000b	Obs	873	565	1.55
		Sim	2025	1456	1.39

(A): Digoxin serum concentration after oral administration. (B) Talinolol plasma concentration after oral administration. (C): Simulated and observed AUC of digoxin and talinolol. Closed circles represent the observed data obtained from literature. Solid lines represent simulated plasma or serum concentration profile of the drug. When plasma or serum concentration of the drug with coadministration of rifampicin was simulated, P-gp abundance was increased 1.7-fold in all intestinal segments compared with that without rifampicin treatment. The time window of AUC calculation is 0-144 hr and 0-24 for digoxin and talinolol, respectively.

References

Agoram, B., Woltosz, W. S., & Bolger, M. B. (2001). Predicting the impact of physiological and biochemical processes on oral drug bioavailability. *Adv Drug Deliv Rev, 50*, S41-67. doi:10.1016/s0169-409x(01)00179-x

Chu, S. Y., Deaton, R., & Cavanaugh, J. (1992). Absolute bioavailability of clarithromycin after oral administration in humans. *Antimicrob Agents Chemother, 36*(5), 1147-1150. doi:10.1128/AAC.36.5.1147

Eberl, S., Renner, B., Neubert, A., Reisig, M., Bachmakov, I., König, I., … Fromm, M. F. (2007). Role of p-glycoprotein inhibition for drug interactions: evidence from in vitro and pharmacoepidemiological studies. *Clin Pharmacokinet, 46*(12), 1039-1049. doi:10.2165/00003088-200746120-00004

McFarland, J. W., Berger, C. M., Froshauer, S. A., Hayashi, S. F., Hecker, S. J., Jaynes, B. H., … Vu, C. B. (1997). Quantitative structure-activity relationships among macrolide antibacterial agents: in vitro and in vivo potency against Pasteurella multocida. *J Med Chem, 40*(9), 1340-1346. doi:10.1021/jm960436i

Neuhoff, S., Ungell, A., Zamora, I., & Artursson, P. (2003). pH-dependent bidirectional transport of weakly basic drugs across Caco-2 monolayers: implications for drug-drug interactions. *Pharm Res, 20*(8), 1141-1148. doi:10.1023/a:1025032511040

Rodvold, K. A. (1999). Clinical pharmacokinetics of clarithromycin. *Clin Pharmacokinet, 37*(5), 385-398. doi:10.2165/00003088-199937050-00003

Schwarz, U. I., Hanso, H., Oertel, R., Miehlke, S., Kuhlisch, E., Glaeser, H., … Kirch, W. (2007). Induction of intestinal P-glycoprotein by St John's wort reduces the oral bioavailability of talinolol. *Clin Pharmacol Ther, 81*(5), 669-678. doi:10.1038/sj.clpt.6100191

Trausch, B., Oertel, R., Richter, K., & Gramatte, T. (1995). Disposition and bioavailability of the beta 1-adrenoceptor antagonist talinolol in man. *Biopharm Drug Dispos, 16*(5), 403-414. doi:10.1002/bdd.2510160505

Tubic, M., Wagner, D., Spahn-Langguth, H., Bolger, M. B., & Langguth, P. (2006). In silico modeling of non-linear drug absorption for the P-gp substrate talinolol and of consequences for the resulting pharmacodynamic effect. *Pharm Res, 23*(8), 1712-1720. doi:10.1007/s11095-006-9020-7

Yago, K., Kuroyama, M., Motohashi, S., & Kumano, K. (1996). [Protein binding of clarithromycin in patients with chronic renal failure]. *Jpn J Antibiot, 49*(3), 256-263.

Yamamoto, T., Itoga, H., Kohno, Y., Nagata, K., & Yamazoe, Y. (2005). Prediction of oral clearance from in vitro metabolic data using recombinant CYPs: comparison among well-stirred, parallel-tube, distributed and dispersion models. *Xenobiotica, 35*(6), 627-646. doi:10.1080/00498250500159371

Yamazaki, S., Costales, C., Lazzaro, S., Eatemadpour, S., Kimoto, E., & Varma, M. V. (2019). Physiologically-Based Pharmacokinetic Modeling Approach to Predict Rifampin-Mediated Intestinal P-Glycoprotein Induction. *CPT Pharmacometrics Syst Pharmacol, 8*(9), 634-642. doi:10.1002/psp4.12458

Model: Caco2_clearance_estimation_v3

Quantities

	Quantity Name	Type	Scope	Value	Initial Value	Units
1	Caco2	compartment	Caco2_clearance_estimation_v3	1	1	microliter
2	Apical_AB	compartment	A_to_B	200	200	microliter
3	compound	species	Apical_AB	0	0	picomole
4	Cell_AB	compartment	A_to_B	0.908	0.908	microliter
5	compound	species	Cell_AB	0	0	picomole
6	Basolateral_AB	compartment	A_to_B	1000	1000	microliter
7	compound	species	Basolateral_AB	0	0	picomole
8	A_to_B	compartment	Caco2	1	1	microliter
9	B_to_A	compartment	Caco2	1	1	microliter
10	Basolateral_BA	compartment	B_to_A	1000	1000	microliter
11	compound	species	Basolateral_BA	0	0	picomole
12	Apical_BA	compartment	B_to_A	200	200	microliter
13	compound	species	Apical_BA	0	0	picomole
14	Cell_BA	compartment	B_to_A	0.908	0.908	microliter
15	compound	species	Cell_BA	0	0	picomole
16	k_ac_a	parameter	Caco2_clearance_estimation_v3	1	0	1/minute
17	k_cb_a	parameter	Caco2_clearance_estimation_v3	1	0.085022	1/minute
18	k_bc_a	parameter	Caco2_clearance_estimation_v3	1	0	1/minute
19	k_ca_a	parameter	Caco2_clearance_estimation_v3	1	0.085022	1/minute
20	CLac	parameter	Caco2_clearance_estimation_v3	0	0	microliter/minute

	Quantity Name	Type	Scope	Value	Initial Value	Units
21	CLca	parameter	Caco2_clearance_estimation_v3	0	0	microliter/minute
22	CLbc	parameter	Caco2_clearance_estimation_v3	0	0	microliter/minute
23	CLcb	parameter	Caco2_clearance_estimation_v3	0	0	microliter/minute
24	CLdiff	parameter	Caco2_clearance_estimation_v3	0	0	microliter/minute
25	Fraction_A	parameter	Caco2_clearance_estimation_v3	1	1	dimensionless
26	pH_A	parameter	Caco2_clearance_estimation_v3	1	1	dimensionless
27	alpha_ac	parameter	Caco2_clearance_estimation_v3	0	0	dimensionless
28	alpha_ca	parameter	Caco2_clearance_estimation_v3	0	0	dimensionless
29	alpha_bc	parameter	Caco2_clearance_estimation_v3	0	0	dimensionless
30	alpha_cb	parameter	Caco2_clearance_estimation_v3	0	0	dimensionless
31	pH_B	parameter	Caco2_clearance_estimation_v3	1	1	dimensionless
32	Fraction_B	parameter	Caco2_clearance_estimation_v3	1	1	dimensionless
33	fu_cell	parameter	Caco2_clearance_estimation_v3	0.0772	0.0772	dimensionless
34	Fraction_C	parameter	Caco2_clearance_estimation_v3	1	1	dimensionless
35	beta_ac	parameter	Caco2_clearance_estimation_v3	1	1	dimensionless
36	beta_ca	parameter	Caco2_clearance_estimation_v3	1	1	dimensionless

	Quantity Name	Type	Scope	Value	Initial Value	Units
37	beta_bc	parameter	Caco2_clearance_estimation_v3	1	1	dimensionless
38	beta_cb	parameter	Caco2_clearance_estimation_v3	1	1	dimensionless
39	Vmax_ac	parameter	Caco2_clearance_estimation_v3	1	1	picomole/minute
40	Vmax_ca	parameter	Caco2_clearance_estimation_v3	1	1	picomole/minute
41	Km_ac	parameter	Caco2_clearance_estimation_v3	1	1	micromole/liter
42	Km_ca	parameter	Caco2_clearance_estimation_v3	1	1	micromole/liter
43	Linear_ac	parameter	Caco2_clearance_estimation_v3	1	1	dimensionless
44	Linear_ca	parameter	Caco2_clearance_estimation_v3	0	0	dimensionless
45	k_ac_b	parameter	Caco2_clearance_estimation_v3	1	0	1/minute
46	k_cb_b	parameter	Caco2_clearance_estimation_v3	1	0.085022	1/minute
47	k_bc_b	parameter	Caco2_clearance_estimation_v3	1	0	1/minute
48	k_ca_b	parameter	Caco2_clearance_estimation_v3	1	0.085022	1/minute
49	Km_bc	parameter	Caco2_clearance_estimation_v3	1	1	micromole/liter
50	Vmax_bc	parameter	Caco2_clearance_estimation_v3	1	1	picomole/minute
51	Linear_bc	parameter	Caco2_clearance_estimation_v3	1	1	dimensionless
52	Km_cb	parameter	Caco2_clearance_estimation_v3	1	1	micromole/liter

	Quantity Name	Type	Scope	Value	Initial Value	Units
53	Vmax_cb	parameter	Caco2_clearance_estimation_v3	1	1	picomole/minute
54	Linear_cb	parameter	Caco2_clearance_estimation_v3	0	0	dimensionless

Initial Assignments

	Initial Assignments	Initial Value
1	k_ac_a = beta_ac*(6.5/pH_A)^alpha_ac*(Linear_ac*CLac+(1-Linear_ac)*(Vmax_ac/(Km_ac+Apical_AB.compound/Apical_AB)))/Apical_AB+Fraction_A*CLdiff/Apical_AB	0
2	k_ca_a = beta_ca*(pH_A/6.5)^alpha_ca*fu_cell*(Linear_ca*CLca+(1-Linear_ca)*(Vmax_ca/(Km_ca+Cell_AB.compound/Cell_AB)))/Cell_AB+(Fraction_C*CLdiff*fu_cell)/Cell_AB	0.085022
3	k_bc_a = beta_bc*(pH_B/6.5)^alpha_bc*(Linear_bc*CLbc+(1-Linear_bc)*(Vmax_bc/(Km_bc+Basolateral_AB.compound/Basolateral_AB)))/Basolateral_AB+Fraction_B*CLdiff/Basolateral_AB	0
4	k_cb_a = beta_cb*(pH_B/6.5)^alpha_cb*fu_cell*(Linear_cb*CLcb+(1-Linear_cb)*(Vmax_cb/(Km_cb+Cell_AB.compound/Cell_AB)))/Cell_AB+(Fraction_C*CLdiff*fu_cell)/Cell_AB	0.085022
5	k_ac_b = beta_ac*(6.5/pH_A)^alpha_ac*(Linear_ac*CLac+(1-Linear_ac)*(Vmax_ac/(Km_ac+Apical_BA.compound/Apical_BA)))/Apical_BA+Fraction_A*CLdiff/Apical_BA	0
6	k_ca_b = beta_ca*(pH_A/6.5)^alpha_ca*fu_cell*(Linear_ca*CLca+(1-Linear_ca)*(Vmax_ca/(Km_ca+Cell_BA.compound/Cell_BA)))/Cell_BA+(Fraction_C*CLdiff*fu_cell)/Cell_BA'	0.085022
7	k_bc_b = beta_bc*(pH_B/6.5)^alpha_bc*(Linear_bc*CLbc+(1-Linear_bc)*(Vmax_bc/(Km_bc+Basolateral_BA.compound/Basolateral_BA)))/Basolateral_BA+Fraction_B*CLdiff/Basolateral_BA	0
8	k_cb_b = beta_cb*(pH_B/6.5)^alpha_cb*fu_cell*(Linear_cb*CLcb+(1-Linear_cb)*(Vmax_cb/(Km_cb+Cell_BA.compound/Cell_BA)))/Cell_BA+(Fraction_C*CLdiff*fu_cell)/Cell_BA	0.085022

Reactions

	Reactions
1	Apical_AB.compound -> Cell_AB.compound
	*k_ac_a*Apical_AB.compound*

	Reactions
2	Cell_AB.compound -> Basolateral_AB.compound
	*k_cb_a*Cell_AB.compound*
3	Basolateral_AB.compound -> Cell_AB.compound
	*k_bc_a*Basolateral_AB.compound*
4	Cell_AB.compound -> Apical_AB.compound
	*k_ca_a*Cell_AB.compound*
5	Apical_BA.compound -> Cell_BA.compound
	*k_ac_b*Apical_BA.compound*
6	Cell_BA.compound -> Basolateral_BA.compound
	*k_cb_b*Cell_BA.compound*
7	Basolateral_BA.compound -> Cell_BA.compound
	*k_bc_b*Basolateral_BA.compound*
8	Cell_BA.compound -> Apical_BA.compound
	*k_ca_b*Cell_BA.compound*

Model Equations

ODEs

	ODEs
1	d(Basolateral_AB.compound)/dt = (k_cb_a*Cell_AB.compound) - (k_bc_a*Basolateral_AB.compound)
2	d(Apical_AB.compound)/dt = -(k_ac_a*Apical_AB.compound) + (k_ca_a*Cell_AB.compound)
3	d(Cell_AB.compound)/dt = (k_ac_a*Apical_AB.compound) - (k_cb_a*Cell_AB.compound) + (k_bc_a*Basolateral_AB.compound) - (k_ca_a*Cell_AB.compound)
4	d(Basolateral_BA.compound)/dt = (k_cb_b*Cell_BA.compound) - (k_bc_b*Basolateral_BA.compound)
5	d(Apical_BA.compound)/dt = -(k_ac_b*Apical_BA.compound) + (k_ca_b*Cell_BA.compound)
6	d(Cell_BA.compound)/dt = (k_ac_b*Apical_BA.compound) - (k_cb_b*Cell_BA.compound) + (k_bc_b*Basolateral_BA.compound) - (k_ca_b*Cell_BA.compound)

Program Setup

Data Step

Data Map

Classification	Value
group	ID
independent	Time
response	B_AMT_AB ~ Basolateral_AB.compound
response	A_AMT_BA ~ Apical_BA.compound
dose from data	Dose_AB -> Apical_AB.compound
	Bolus
dose from data	Dose_BA -> Basolateral_BA.compound

Classification	Value
	Bolus
variant from data	pH_A ~ pH_A
	UnitConversion: auto
variant from data	pH_B ~ pH_B
	UnitConversion: auto
variant from data	Fraction_A ~ Fraction_A
	UnitConversion: auto
variant from data	Fraction_B ~ Fraction_B
	UnitConversion: auto
variant from data	Fraction_C ~ Fraction_C
	UnitConversion: auto
variant from data	beta_ac ~ beta_ac
	UnitConversion: auto
variant from data	beta_ca ~ beta_ca
	UnitConversion: auto
variant from data	beta_bc ~ beta_bc
	UnitConversion: auto
variant from data	beta_cb ~ beta_cb
	UnitConversion: auto

Variant and Dose Setup Step

Variant and Dose Setup

Group	Variants1	Variants2	Variants3	Variants4	Variants5	Variants6	Variants7	Variants8	Variants9	Variants10	Doses1	Doses2	Doses3
	Group Specific	Data	Data	Data	Data	Data	Data	Data	Data	Data	Group Specific	Data	Data
1		pH_A	pH_B	Fraction_A	Fraction_B	Fraction_C	beta_ac	beta_ca	beta_bc	beta_cb		Dose_AB	Dose_BA
2		pH_A	pH_B	Fraction_A	Fraction_B	Fraction_C	beta_ac	beta_ca	beta_bc	beta_cb		Dose_AB	Dose_BA
3		pH_A	pH_B	Fraction_A	Fraction_B	Fraction_C	beta_ac	beta_ca	beta_bc	beta_cb		Dose_AB	Dose_BA
4		pH_A	pH_B	Fraction_A	Fraction_B	Fraction_C	beta_ac	beta_ca	beta_bc	beta_cb		Dose_AB	Dose_BA
5		pH_A	pH_B	Fraction_A	Fraction_B	Fraction_C	beta_ac	beta_ca	beta_bc	beta_cb		Dose_AB	Dose_BA
6		pH_A	pH_B	Fraction_A	Fraction_B	Fraction_C	beta_ac	beta_ca	beta_bc	beta_cb		Dose_AB	Dose_BA
7		pH_A	pH_B	Fraction_A	Fraction_B	Fraction_C	beta_ac	beta_ca	beta_bc	beta_cb		Dose_AB	Dose_BA

Group	Variants1	Variants2	Variants3	Variants4	Variants5	Variants6	Variants7	Variants8	Variants9	Variants10	Doses1	Doses2	Doses3
8		pH_A	pH_B	Fraction_A	Fraction_B	Fraction_C	beta_ac	beta_ca	beta_bc	beta_cb		Dose_AB	Dose_BA
9		pH_A	pH_B	Fraction_A	Fraction_B	Fraction_C	beta_ac	beta_ca	beta_bc	beta_cb		Dose_AB	Dose_BA
10		pH_A	pH_B	Fraction_A	Fraction_B	Fraction_C	beta_ac	beta_ca	beta_bc	beta_cb		Dose_AB	Dose_BA
11		pH_A	pH_B	Fraction_A	Fraction_B	Fraction_C	beta_ac	beta_ca	beta_bc	beta_cb		Dose_AB	Dose_BA
12		pH_A	pH_B	Fraction_A	Fraction_B	Fraction_C	beta_ac	beta_ca	beta_bc	beta_cb		Dose_AB	Dose_BA
13		pH_A	pH_B	Fraction_A	Fraction_B	Fraction_C	beta_ac	beta_ca	beta_bc	beta_cb		Dose_AB	Dose_BA
14		pH_A	pH_B	Fraction_A	Fraction_B	Fraction_C	beta_ac	beta_ca	beta_bc	beta_cb		Dose_AB	Dose_BA
15		pH_A	pH_B	Fraction_A	Fraction_B	Fraction_C	beta_ac	beta_ca	beta_bc	beta_cb		Dose_AB	Dose_BA
16		pH_A	pH_B	Fraction_A	Fraction_B	Fraction_C	beta_ac	beta_ca	beta_bc	beta_cb		Dose_AB	Dose_BA
17		pH_A	pH_B	Fraction_A	Fraction_B	Fraction_C	beta_ac	beta_ca	beta_bc	beta_cb		Dose_AB	Dose_BA
18		pH_A	pH_B	Fraction_A	Fraction_B	Fraction_C	beta_ac	beta_ca	beta_bc	beta_cb		Dose_AB	Dose_BA
19		pH_A	pH_B	Fraction_A	Fraction_B	Fraction_C	beta_ac	beta_ca	beta_bc	beta_cb		Dose_AB	Dose_BA
20		pH_A	pH_B	Fraction_A	Fraction_B	Fraction_C	beta_ac	beta_ca	beta_bc	beta_cb		Dose_AB	Dose_BA
21		pH_A	pH_B	Fraction_A	Fraction_B	Fraction_C	beta_ac	beta_ca	beta_bc	beta_cb		Dose_AB	Dose_BA
22		pH_A	pH_B	Fraction_A	Fraction_B	Fraction_C	beta_ac	beta_ca	beta_bc	beta_cb		Dose_AB	Dose_BA
23		pH_A	pH_B	Fraction_A	Fraction_B	Fraction_C	beta_ac	beta_ca	beta_bc	beta_cb		Dose_AB	Dose_BA
24		pH_A	pH_B	Fraction_A	Fraction_B	Fraction_C	beta_ac	beta_ca	beta_bc	beta_cb		Dose_AB	Dose_BA
25		pH_A	pH_B	Fraction_A	Fraction_B	Fraction_C	beta_ac	beta_ca	beta_bc	beta_cb		Dose_AB	Dose_BA
26		pH_A	pH_B	Fraction_A	Fraction_B	Fraction_C	beta_ac	beta_ca	beta_bc	beta_cb		Dose_AB	Dose_BA
27		pH_A	pH_B	Fraction_A	Fraction_B	Fraction_C	beta_ac	beta_ca	beta_bc	beta_cb		Dose_AB	Dose_BA
28		pH_A	pH_B	Fraction_A	Fraction_B	Fraction_C	beta_ac	beta_ca	beta_bc	beta_cb		Dose_AB	Dose_BA
29		pH_A	pH_B	Fraction_A	Fraction_B	Fraction_C	beta_ac	beta_ca	beta_bc	beta_cb		Dose_AB	Dose_BA

Group	Variants1	Variants2	Variants3	Variants4	Variants5	Variants6	Variants7	Variants8	Variants9	Variants10	Doses1	Doses2	Doses3
30		pH_A	pH_B	Fraction_A	Fraction_B	Fraction_C	beta_ac	beta_ca	beta_bc	beta_cb		Dose_AB	Dose_BA
31		pH_A	pH_B	Fraction_A	Fraction_B	Fraction_C	beta_ac	beta_ca	beta_bc	beta_cb		Dose_AB	Dose_BA
32		pH_A	pH_B	Fraction_A	Fraction_B	Fraction_C	beta_ac	beta_ca	beta_bc	beta_cb		Dose_AB	Dose_BA
33		pH_A	pH_B	Fraction_A	Fraction_B	Fraction_C	beta_ac	beta_ca	beta_bc	beta_cb		Dose_AB	Dose_BA
34		pH_A	pH_B	Fraction_A	Fraction_B	Fraction_C	beta_ac	beta_ca	beta_bc	beta_cb		Dose_AB	Dose_BA
35		pH_A	pH_B	Fraction_A	Fraction_B	Fraction_C	beta_ac	beta_ca	beta_bc	beta_cb		Dose_AB	Dose_BA
36		pH_A	pH_B	Fraction_A	Fraction_B	Fraction_C	beta_ac	beta_ca	beta_bc	beta_cb		Dose_AB	Dose_BA
37		pH_A	pH_B	Fraction_A	Fraction_B	Fraction_C	beta_ac	beta_ca	beta_bc	beta_cb		Dose_AB	Dose_BA
38		pH_A	pH_B	Fraction_A	Fraction_B	Fraction_C	beta_ac	beta_ca	beta_bc	beta_cb		Dose_AB	Dose_BA
39		pH_A	pH_B	Fraction_A	Fraction_B	Fraction_C	beta_ac	beta_ca	beta_bc	beta_cb		Dose_AB	Dose_BA
40		pH_A	pH_B	Fraction_A	Fraction_B	Fraction_C	beta_ac	beta_ca	beta_bc	beta_cb		Dose_AB	Dose_BA
41		pH_A	pH_B	Fraction_A	Fraction_B	Fraction_C	beta_ac	beta_ca	beta_bc	beta_cb		Dose_AB	Dose_BA
42		pH_A	pH_B	Fraction_A	Fraction_B	Fraction_C	beta_ac	beta_ca	beta_bc	beta_cb		Dose_AB	Dose_BA
43		pH_A	pH_B	Fraction_A	Fraction_B	Fraction_C	beta_ac	beta_ca	beta_bc	beta_cb		Dose_AB	Dose_BA
44		pH_A	pH_B	Fraction_A	Fraction_B	Fraction_C	beta_ac	beta_ca	beta_bc	beta_cb		Dose_AB	Dose_BA
45		pH_A	pH_B	Fraction_A	Fraction_B	Fraction_C	beta_ac	beta_ca	beta_bc	beta_cb		Dose_AB	Dose_BA
46		pH_A	pH_B	Fraction_A	Fraction_B	Fraction_C	beta_ac	beta_ca	beta_bc	beta_cb		Dose_AB	Dose_BA
47		pH_A	pH_B	Fraction_A	Fraction_B	Fraction_C	beta_ac	beta_ca	beta_bc	beta_cb		Dose_AB	Dose_BA
48		pH_A	pH_B	Fraction_A	Fraction_B	Fraction_C	beta_ac	beta_ca	beta_bc	beta_cb		Dose_AB	Dose_BA
49		pH_A	pH_B	Fraction_A	Fraction_B	Fraction_C	beta_ac	beta_ca	beta_bc	beta_cb		Dose_AB	Dose_BA
50		pH_A	pH_B	Fraction_A	Fraction_B	Fraction_C	beta_ac	beta_ca	beta_bc	beta_cb		Dose_AB	Dose_BA
51		pH_A	pH_B	Fraction_A	Fraction_B	Fraction_C	beta_ac	beta_ca	beta_bc	beta_cb		Dose_AB	Dose_BA

Group	Variants1	Variants2	Variants3	Variants4	Variants5	Variants6	Variants7	Variants8	Variants9	Variants10	Doses1	Doses2	Doses3
52		pH_A	pH_B	Fraction_A	Fraction_B	Fraction_C	beta_ac	beta_ca	beta_bc	beta_cb		Dose_AB	Dose_BA
53		pH_A	pH_B	Fraction_A	Fraction_B	Fraction_C	beta_ac	beta_ca	beta_bc	beta_cb		Dose_AB	Dose_BA
54		pH_A	pH_B	Fraction_A	Fraction_B	Fraction_C	beta_ac	beta_ca	beta_bc	beta_cb		Dose_AB	Dose_BA
55		pH_A	pH_B	Fraction_A	Fraction_B	Fraction_C	beta_ac	beta_ca	beta_bc	beta_cb		Dose_AB	Dose_BA
56		pH_A	pH_B	Fraction_A	Fraction_B	Fraction_C	beta_ac	beta_ca	beta_bc	beta_cb		Dose_AB	Dose_BA
57		pH_A	pH_B	Fraction_A	Fraction_B	Fraction_C	beta_ac	beta_ca	beta_bc	beta_cb		Dose_AB	Dose_BA
58		pH_A	pH_B	Fraction_A	Fraction_B	Fraction_C	beta_ac	beta_ca	beta_bc	beta_cb		Dose_AB	Dose_BA
59		pH_A	pH_B	Fraction_A	Fraction_B	Fraction_C	beta_ac	beta_ca	beta_bc	beta_cb		Dose_AB	Dose_BA
60		pH_A	pH_B	Fraction_A	Fraction_B	Fraction_C	beta_ac	beta_ca	beta_bc	beta_cb		Dose_AB	Dose_BA
61		pH_A	pH_B	Fraction_A	Fraction_B	Fraction_C	beta_ac	beta_ca	beta_bc	beta_cb		Dose_AB	Dose_BA
62		pH_A	pH_B	Fraction_A	Fraction_B	Fraction_C	beta_ac	beta_ca	beta_bc	beta_cb		Dose_AB	Dose_BA
63		pH_A	pH_B	Fraction_A	Fraction_B	Fraction_C	beta_ac	beta_ca	beta_bc	beta_cb		Dose_AB	Dose_BA
64		pH_A	pH_B	Fraction_A	Fraction_B	Fraction_C	beta_ac	beta_ca	beta_bc	beta_cb		Dose_AB	Dose_BA

Fit Step

Estimated Parameters (Pooled Fit)

Name	Transformation	Initial Untransformed Value	Untransformed Bounds
CLbc	log	0.03	[0.003 0.3]
CLdiff	log	10	[1 100]
Vmax_ca	log	10	[1 100]
Km_ca	log	3	[0.3 30]
alpha_ca	none	10	[1 100]
alpha_bc	none	4	[0.4 40]
alpha_cb	none	5	[0.5 50]
Vmax_cb	log	3	[0.3 30]
Km_cb	log	10	[1 100]

Error Model

Use one common error model for all responses: combined

Algorithm Settings

Property	Value
EstimationFcn	scattersearch
MaxIterations	400
FunctionTolerance	1e-08
MaxStallIterations	50
MaxTime	Inf
NumInitialPoints	1800
NumTrialPoints	auto
XTolerance	1e-06
LocalSolver	lsqnonlin

Local Solver Settings

Property	Value
StepTolerance	1e-08
FunctionTolerance	1e-08
OptimalityTolerance	1e-06
MaxIterations	400

Confidence Interval Step

Parameter Confidence Interval Options

Property	Value
Confidence Level (%)	95
Type	gaussian
UseParallel	true

Prediction Confidence Interval Options

Property	Value
Confidence Level (%)	95
Type	gaussian
UseParallel	true

Program Results

Fit Step

Pooled Parameter Estimates

Name	Estimate	StandardError
CLbc	0.039138	0.00084428
CLdiff	9.4396	0.16351
Vmax_ca	11.3519	0.039783

Name	Estimate	StandardError
Km_ca	0.56739	0.0010584
alpha_ca	19.305	0.02609
alpha_bc	4.6508	0.32795
alpha_cb	20.1224	0.24034
Vmax_cb	8.0993	0.2047
Km_cb	48.1729	1.3372

Statistics

Name	Value
AIC	674.6473
BIC	700.3156
LogLikelihood	-328.3236
DFE	119
MSE	3303.2651
SSE	393088.546

Pooled Beta

Name	Estimate	StandardError
log(CLbc)	-3.2406	0.021572
log(CLdiff)	2.2449	0.017322
log(Vmax_ca)	2.4294	0.0035045
log(Km_ca)	-0.56672	0.0018654
alpha_ca	19.305	0.02609
alpha_bc	4.6508	0.32795
alpha_cb	20.1224	0.24034
log(Vmax_cb)	2.0918	0.025274
log(Km_cb)	3.8748	0.027758

Residuals

ID	Time	B_AMT_AB	A_AMT_BA
1	0	NaN	NaN
1	60	-0.055815	NaN
1	120	-1.0167	NaN
2	0	NaN	NaN
2	60	-0.10862	NaN
2	120	-0.7887	NaN
3	0	NaN	NaN
3	60	NaN	0.85126
3	120	NaN	-9.6716

ID	Time	B_AMT_AB	A_AMT_BA
4	0	NaN	NaN
4	60	NaN	0.1523
4	120	NaN	-11.369
5	0	NaN	NaN
5	60	0.64761	NaN
5	120	0.6152	NaN
6	0	NaN	NaN
6	60	0.18497	NaN
6	120	0.15438	NaN
7	0	NaN	NaN
7	60	NaN	9.4902
7	120	NaN	10.6177
8	0	NaN	NaN
8	60	NaN	5.8577
8	120	NaN	5.4156
9	0	NaN	NaN
9	60	1.3518	NaN
9	120	1.9076	NaN
10	0	NaN	NaN
10	60	0.48493	NaN
10	120	1.2817	NaN
11	0	NaN	NaN
11	60	NaN	19.7541
11	120	NaN	29.5891
12	0	NaN	NaN
12	60	NaN	16.1284
12	120	NaN	22.8187
13	0	NaN	NaN
13	60	1.1516	NaN
13	120	1.4022	NaN
14	0	NaN	NaN
14	60	0.33284	NaN
14	120	0.12059	NaN
15	0	NaN	NaN
15	60	NaN	31.994
15	120	NaN	45.7697
16	0	NaN	NaN
16	60	NaN	29.1128

ID	Time	B_AMT_AB	A_AMT_BA
16	120	NaN	43.5957
17	0	NaN	NaN
17	60	0.71168	NaN
17	120	0.52927	NaN
18	0	NaN	NaN
18	60	0.010174	NaN
18	120	0.31701	NaN
19	0	NaN	NaN
19	60	NaN	38.9592
19	120	NaN	43.6018
20	0	NaN	NaN
20	60	NaN	37.6735
20	120	NaN	56.3002
21	0	NaN	NaN
21	60	0.31137	NaN
21	120	-0.12408	NaN
22	0	NaN	NaN
22	60	0.12213	NaN
22	120	-0.77429	NaN
23	0	NaN	NaN
23	60	NaN	83.3516
23	120	NaN	133.1984
24	0	NaN	NaN
24	60	NaN	78.1422
24	120	NaN	113.2518
25	0	NaN	NaN
25	60	-0.045264	NaN
25	120	-0.071778	NaN
26	0	NaN	NaN
26	60	-0.081252	NaN
26	120	-0.21353	NaN
27	0	NaN	NaN
27	60	NaN	-0.11286
27	120	NaN	-0.78533
28	0	NaN	NaN
28	60	NaN	-0.21347
28	120	NaN	-1.1103
29	0	NaN	NaN

ID	Time	B_AMT_AB	A_AMT_BA
29	60	-0.092113	NaN
29	120	-0.056261	NaN
30	0	NaN	NaN
30	60	-0.073775	NaN
30	120	-0.3442	NaN
31	0	NaN	NaN
31	60	NaN	0.058158
31	120	NaN	-1.6063
32	0	NaN	NaN
32	60	NaN	-0.60105
32	120	NaN	-2.6494
33	0	NaN	NaN
33	60	-0.049894	NaN
33	120	-0.10226	NaN
34	0	NaN	NaN
34	60	0.13463	NaN
34	120	0.044041	NaN
35	0	NaN	NaN
35	60	NaN	-0.011303
35	120	NaN	-0.016008
36	0	NaN	NaN
36	60	NaN	0.054462
36	120	NaN	0.097749
37	0	NaN	NaN
37	60	-0.048713	NaN
37	120	-0.12203	NaN
38	0	NaN	NaN
38	60	-0.34879	NaN
38	120	-0.39816	NaN
39	0	NaN	NaN
39	60	NaN	-1.3056
39	120	NaN	-6.8101
40	0	NaN	NaN
40	60	NaN	-2.5515
40	120	NaN	-9.5912
41	0	NaN	NaN
41	60	0.045849	NaN
41	120	-0.12097	NaN

ID	Time	B_AMT_AB	A_AMT_BA
42	0	NaN	NaN
42	60	0.039746	NaN
42	120	-0.64663	NaN
43	0	NaN	NaN
43	60	NaN	-7.6989
43	120	NaN	-26.5954
44	0	NaN	NaN
44	60	NaN	-9.1049
44	120	NaN	-27.1938
45	0	NaN	NaN
45	60	-0.94333	NaN
45	120	-5.8674	NaN
46	0	NaN	NaN
46	60	-0.23353	NaN
46	120	-4.329	NaN
47	0	NaN	NaN
47	60	NaN	-1.003
47	120	NaN	-1.8384
48	0	NaN	NaN
48	60	NaN	-1.0415
48	120	NaN	-2.6408
49	0	NaN	NaN
49	60	-0.33892	NaN
49	120	-1.5189	NaN
50	0	NaN	NaN
50	60	-0.95915	NaN
50	120	-2.4328	NaN
51	0	NaN	NaN
51	60	NaN	-22.3288
51	120	NaN	-68.3209
52	0	NaN	NaN
52	60	NaN	-18.8586
52	120	NaN	-60.6901
53	0	NaN	NaN
53	60	-1.763	NaN
53	120	-7.8187	NaN
54	0	NaN	NaN
54	60	0.39148	NaN

ID	Time	B_AMT_AB	A_AMT_BA
54	120	-0.72748	NaN
55	0	NaN	NaN
55	60	NaN	-1.9382
55	120	NaN	-42.2629
56	0	NaN	NaN
56	60	NaN	-16.5043
56	120	NaN	-45.5297
57	0	NaN	NaN
57	60	21.6505	NaN
57	120	18.5042	NaN
58	0	NaN	NaN
58	60	22.967	NaN
58	120	18.1951	NaN
59	0	NaN	NaN
59	60	NaN	-3.7591
59	120	NaN	2.1039
60	0	NaN	NaN
60	60	NaN	3.4373
60	120	NaN	23.8853
61	0	NaN	NaN
61	60	2.8635	NaN
61	120	2.6716	NaN
62	0	NaN	NaN
62	60	2.2399	NaN
62	120	-3.06	NaN
63	0	NaN	NaN
63	60	NaN	188.9139
63	120	NaN	428.1855
64	0	NaN	NaN
64	60	NaN	125.6488
64	120	NaN	284.0637

Covariance Matrix

Name	CLbc	CLdiff	Vmax_ca	Km_ca	alpha_ca	alpha_bc	alpha_cb	Vmax_cb	Km_cb
CLbc	7.128e-07	1.9104e-05	1.9571e-05	8.0656e-08	6.498e-06	1.0318e-05	7.9483e-05	-7.2116e-05	-0.00037235
CLdiff	1.9104e-05	0.026737	-0.00088111	-6.2976e-05	0.0015034	-0.0031838	-0.0070637	0.0043178	-0.19222

Name	CLbc	CLdiff	Vmax_ca	Km_ca	alpha_ca	alpha_bc	alpha_cb	Vmax_cb	Km_cb
Vmax_ca	1.9571e-05	-0.00088111	0.0015827	-2.0951e-06	0.00024287	-0.0001886	-0.00030773	4.1825e-05	-0.0020247
Km_ca	8.0656e-08	-6.2976e-05	-2.0951e-06	1.1202e-06	-1.8105e-05	1.7064e-05	0.00010447	-7.2672e-05	-7.1895e-05
alpha_ca	6.498e-06	0.0015034	0.00024287	-1.8105e-05	0.0006807	0.00018622	0.00064436	-0.0011343	-0.0047832
alpha_bc	1.0318e-05	-0.0031838	-0.0001886	1.7064e-05	0.00018622	0.10755	0.010955	-0.0094921	0.0091592
alpha_cb	7.9483e-05	-0.0070637	-0.00030773	0.00010447	0.00064436	0.010955	0.057764	-0.048468	-0.002887
Vmax_cb	-7.2116e-05	0.0043178	4.1825e-05	-7.2672e-05	-0.0011343	-0.0094921	-0.048468	0.041901	0.0053655
Km_cb	-0.00037235	-0.19222	-0.0020247	-7.1895e-05	-0.0047832	0.0091592	-0.002887	0.0053655	1.7881

Error Model

Response	ErrorModel	a	b
	combined	0.12489	0.27633

Confidence Interval Step

Parameter Confidence Interval Results

Group	Name	Estimate	ConfidenceInterval	Type	Alpha	Status
pooled	CLbc	0.039138	0.037467 0.04081	Gaussian	0.05	success
pooled	CLdiff	9.4396	9.1159 9.7634	Gaussian	0.05	success
pooled	Vmax_ca	11.3519	11.2731 11.4307	Gaussian	0.05	success
pooled	Km_ca	0.56739	0.56529 0.56948	Gaussian	0.05	success
pooled	alpha_ca	19.305	19.2534 19.3567	Gaussian	0.05	success
pooled	alpha_bc	4.6508	4.0014 5.3002	Gaussian	0.05	success
pooled	alpha_cb	20.1224	19.6465 20.5983	Gaussian	0.05	success
pooled	Vmax_cb	8.0993	7.694 8.5046	Gaussian	0.05	success
pooled	Km_cb	48.1729	45.5251 50.8207	Gaussian	0.05	success

Model: PBPK_MADAM

Quantities

	Quantity Name	Type	Scope	Value	Initial Value	Units
1	Main_compartment	compartment	PBPK_MADAM	1	1	liter
2	Bile_drug	species	Main_compartment	0	0	milligram
3	Venous	compartment	Main_compartment	1	3.598	liter
4	Venous_drug	species	Venous	0	0	milligram/liter
5	Lung	compartment	Main_compartment	1	0.56	liter
6	Lung_drug	species	Lung	0	0	milligram/liter
7	Kidney	compartment	Main_compartment	1	0.35	liter
8	Kidney_drug	species	Kidney	0	0	milligram/liter
9	Brain	compartment	Main_compartment	1	1.47	liter
10	Brain_drug	species	Brain	0	0	milligram/liter
11	Muscle	compartment	Main_compartment	1	29.12	liter
12	Muscle_drug	species	Muscle	0	0	milligram/liter
13	Adipose	compartment	Main_compartment	1	13.79	liter
14	Adipose_drug	species	Adipose	0	0	milligram/liter
15	Heart	compartment	Main_compartment	1	0.35	liter
16	Heart_drug	species	Heart	0	0	milligram/liter
17	Skin	compartment	Main_compartment	1	2.87	liter
18	Skin_drug	species	Skin	0	0	milligram/liter
19	Bone	compartment	Main_compartment	1	11.06	liter
20	Bone_drug	species	Bone	0	0	milligram/liter
21	Rest	compartment	Main_compartment	1	7	liter
22	Rest_drug	species	Rest	0	0	milligram/liter
23	Artery	compartment	Main_compartment	1	1.799	liter
24	Artery_drug	species	Artery	0	0	milligram/liter
25	Gut	compartment	Main_compartment	1	1.26	liter
26	Gut_drug	species	Gut	0	0	milligram/liter
27	Spleen	compartment	Main_compartment	1	0.21	liter
28	Spleen_drug	species	Spleen	0	0	milligram/liter

	Quantity Name	Type	Scope	Value	Initial Value	Units
29	Liver_EC_S1	compartment	Main_compartment	1	0.0686	liter
30	Liver_EC_S1_drug	species	Liver_EC_S1	0	0	milligram/liter
31	Liver_EC_S2	compartment	Main_compartment	1	0.0686	liter
32	Liver_EC_S2_drug	species	Liver_EC_S2	0	0	milligram/liter
33	Liver_EC_S3	compartment	Main_compartment	1	0.0686	liter
34	Liver_EC_S3_drug	species	Liver_EC_S3	0	0	milligram/liter
35	Liver_EC_S4	compartment	Main_compartment	1	0.0686	liter
36	Liver_EC_S4_drug	species	Liver_EC_S4	0	0	milligram/liter
37	Liver_EC_S5	compartment	Main_compartment	1	0.0686	liter
38	Liver_EC_S5_drug	species	Liver_EC_S5	0	0	milligram/liter
39	Liver_IC_S5	compartment	Main_compartment	1	0.252	liter
40	Liver_IC_S5_drug	species	Liver_IC_S5	0	0	milligram/liter
41	Liver_IC_S4	compartment	Main_compartment	1	0.252	liter
42	Liver_IC_S4_drug	species	Liver_IC_S4	0	0	milligram/liter
43	Liver_IC_S3	compartment	Main_compartment	1	0.252	liter
44	Liver_IC_S3_drug	species	Liver_IC_S3	0	0	milligram/liter
45	Liver_IC_S1	compartment	Main_compartment	1	0.252	liter
46	Liver_IC_S1_drug	species	Liver_IC_S1	0	0	milligram/liter
47	Liver_IC_S2	compartment	Main_compartment	1	0.252	liter
48	Liver_IC_S2_drug	species	Liver_IC_S2	0	0	milligram/liter
49	Metabolites	compartment	Main_compartment	1	1	liter
50	Metabolites_drug	species	Metabolites	0	0	milligram
51	Testes	compartment	Main_compartment	1	0.07	liter
52	Testes_drug	species	Testes	0	0	milligram/liter
53	Blood_total	compartment	Main_compartment	1	1	liter

	Quantity Name	Type	Scope	Value	Initial Value	Units
54	Blood_total_drug	species	Blood_total	0	0	milligram/liter
55	Plasma_total	compartment	Main_compartment	1	1	liter
56	Plasma_total_drug	species	Plasma_total	0	0	nanogram/milliliter
57	Plasma_free_uM	species	Plasma_total	0	0	micromole/liter
58	Plasma_total_uM	species	Plasma_total	0	0	micromole/liter
59	Portal	compartment	Main_compartment	1	1	liter
60	Portal_drug	species	Portal	0	0	milligram/liter
61	Mass_Balance	compartment	Main_compartment	1	1	liter
62	Amount_body	species	Mass_Balance	0	0	milligram
63	Amount_total	species	Mass_Balance	0	0	milligram
64	Urine	compartment	Main_compartment	1	1	liter
65	Urine_drug	species	Urine	0	0	milligram
66	Liver_total	compartment	Main_compartment	1	1	liter
67	Liver_tissue_total	species	Liver_total	0	0	milligram/liter
68	Liver_blood_total	species	Liver_total	0	0	milligram/liter
69	Liver_blood_free	species	Liver_total	0	0	milligram/liter
70	Liver_tissue_free	species	Liver_total	0	0	milligram/liter
71	Liver_tissue_total_uM	species	Liver_total	0	0	micromole/liter
72	convert_to_nmole_per_kg	compartment	Main_compartment	1	1	liter
73	Venous_nmole	species	convert_to_nmole_per_kg	0	0	nanomole
74	Artery_nmole	species	convert_to_nmole_per_kg	0	0	nanomole
75	Bone_nmole	species	convert_to_nmole_per_kg	0	0	nanomole
76	Adipose_nmole	species	convert_to_nmole_per_kg	0	0	nanomole
77	Muscle_nmole	species	convert_to_nmole_per_kg	0	0	nanomole
78	Urine_nmole	species	convert_to_nmole_per_kg	0	0	nanomole

	Quantity Name	Type	Scope	Value	Initial Value	Units
79	Liver_EC1_nmole	species	convert_to_nmole_per_kg	0	0	nanomole
80	Liver_IC1_nmole	species	convert_to_nmole_per_kg	0	0	nanomole
81	Bile_nmole	species	convert_to_nmole_per_kg	0	0	nanomole
82	Kidney_nmole	species	convert_to_nmole_per_kg	0	0	nanomole
83	Lung_nmole	species	convert_to_nmole_per_kg	0	0	nanomole
84	Metabolites_nmole	species	convert_to_nmole_per_kg	0	0	nanomole
85	Liver_IC2_nmole	species	convert_to_nmole_per_kg	0	0	nanomole
86	Liver_EC2_nmole	species	convert_to_nmole_per_kg	0	0	nanomole
87	Liver_EC3_nmole	species	convert_to_nmole_per_kg	0	0	nanomole
88	Liver_IC3_nmole	species	convert_to_nmole_per_kg	0	0	nanomole
89	Liver_IC4_nmole	species	convert_to_nmole_per_kg	0	0	nanomole
90	Liver_EC4_nmole	species	convert_to_nmole_per_kg	0	0	nanomole
91	Liver_EC5_nmole	species	convert_to_nmole_per_kg	0	0	nanomole
92	Liver_IC5_nmole	species	convert_to_nmole_per_kg	0	0	nanomole
93	Gut_nmole	species	convert_to_nmole_per_kg	0	0	nanomole
94	Spleen_nmole	species	convert_to_nmole_per_kg	0	0	nanomole
95	Skin_nmole	species	convert_to_nmole_per_kg	0	0	nanomole
96	Brain_nmole	species	convert_to_nmole_per_kg	0	0	nanomole
97	Rest_nmole	species	convert_to_nmole_per_kg	0	0	nanomole
98	Heart_nmole	species	convert_to_nmole_per_kg	0	0	nanomole
99	X_CECUM_DISS_nmole	species	convert_to_nmole_per_kg	0	0	nanomole
100	Main_compartment_1	compartment	PBPK_MADAM	1	1	liter
101	Bile_drug_1	species	Main_compartment_1	0	0	milligram
102	Venous_1	compartment	Main_compartment_1	1	3.598	liter
103	Venous_drug_1	species	Venous_1	0	0	milligram/liter

	Quantity Name	Type	Scope	Value	Initial Value	Units
104	Lung_1	compartment	Main_compartment_1	1	0.56	liter
105	Lung_drug_1	species	Lung_1	0	0	milligram/liter
106	Kidney_1	compartment	Main_compartment_1	1	0.35	liter
107	Kidney_drug_1	species	Kidney_1	0	0	milligram/liter
108	Brain_1	compartment	Main_compartment_1	1	1.47	liter
109	Brain_drug_1	species	Brain_1	0	0	milligram/liter
110	Muscle_1	compartment	Main_compartment_1	1	29.12	liter
111	Muscle_drug_1	species	Muscle_1	0	0	milligram/liter
112	Adipose_1	compartment	Main_compartment_1	1	13.79	liter
113	Adipose_drug_1	species	Adipose_1	0	0	milligram/liter
114	Heart_1	compartment	Main_compartment_1	1	0.35	liter
115	Heart_drug_1	species	Heart_1	0	0	milligram/liter
116	Skin_1	compartment	Main_compartment_1	1	2.87	liter
117	Skin_drug_1	species	Skin_1	0	0	milligram/liter
118	Bone_1	compartment	Main_compartment_1	1	11.06	liter
119	Bone_drug_1	species	Bone_1	0	0	milligram/liter
120	Rest_1	compartment	Main_compartment_1	1	7	liter
121	Rest_drug_1	species	Rest_1	0	0	milligram/liter
122	Artery_1	compartment	Main_compartment_1	1	1.799	liter
123	Artery_drug_1	species	Artery_1	0	0	milligram/liter
124	Gut_1	compartment	Main_compartment_1	1	1.26	liter
125	Gut_drug_1	species	Gut_1	0	0	milligram/liter
126	Spleen_1	compartment	Main_compartment_1	1	0.21	liter
127	Spleen_drug_1	species	Spleen_1	0	0	milligram/liter
128	Liver_EC_S1_1	compartment	Main_compartment_1	1	0.0686	liter
129	Liver_EC_S1_drug_1	species	Liver_EC_S1_1	0	0	milligram/liter
130	Liver_EC_S2_1	compartment	Main_compartment_1	1	0.0686	liter

	Quantity Name	Type	Scope	Value	Initial Value	Units
131	Liver_EC_S2_drug_1	species	Liver_EC_S2_1	0	0	milligram/liter
132	Liver_EC_S3_1	compartment	Main_compartment_1	1	0.0686	liter
133	Liver_EC_S3_drug_1	species	Liver_EC_S3_1	0	0	milligram/liter
134	Liver_EC_S4_1	compartment	Main_compartment_1	1	0.0686	liter
135	Liver_EC_S4_drug_1	species	Liver_EC_S4_1	0	0	milligram/liter
136	Liver_EC_S5_1	compartment	Main_compartment_1	1	0.0686	liter
137	Liver_EC_S5_drug_1	species	Liver_EC_S5_1	0	0	milligram/liter
138	Liver_IC_S5_1	compartment	Main_compartment_1	1	0.252	liter
139	Liver_IC_S5_drug_1	species	Liver_IC_S5_1	0	0	milligram/liter
140	Liver_IC_S3_1	compartment	Main_compartment_1	1	0.252	liter
141	Liver_IC_S3_drug_1	species	Liver_IC_S3_1	0	0	milligram/liter
142	Liver_IC_S1_1	compartment	Main_compartment_1	1	0.252	liter
143	Liver_IC_S1_drug_1	species	Liver_IC_S1_1	0	0	milligram/liter
144	Liver_IC_S2_1	compartment	Main_compartment_1	1	0.252	liter
145	Liver_IC_S2_drug_1	species	Liver_IC_S2_1	0	0	milligram/liter
146	Metabolites_1	compartment	Main_compartment_1	1	1	liter
147	Metabolites_drug_1	species	Metabolites_1	0	0	milligram
148	Testes_1	compartment	Main_compartment_1	1	0.07	liter
149	Testes_drug_1	species	Testes_1	0	0	milligram/liter
150	Blood_total_1	compartment	Main_compartment_1	1	1	liter
151	Blood_total_drug_1	species	Blood_total_1	0	0	milligram/liter
152	Plasma_total_1	compartment	Main_compartment_1	1	1	liter
153	Plasma_total_drug_1	species	Plasma_total_1	0	0	nanogram/milliliter
154	Plasma_total_uM_1	species	Plasma_total_1	0	0	micromole/liter
155	Plasma_free_uM_1	species	Plasma_total_1	0	0	micromole/liter

	Quantity Name	Type	Scope	Value	Initial Value	Units
156	Portal_1	compartment	Main_compartment_1	1	1	liter
157	Portal_drug_1	species	Portal_1	0	0	milligram/liter
158	Portal_plasma_drug_1	species	Portal_1	0	0	milligram/liter
159	Portal_plasma_drug	species	Portal_1	0	0	milligram/liter
160	Mass_Balance_1	compartment	Main_compartment_1	1	1	liter
161	Amount_body_1	species	Mass_Balance_1	0	0	milligram
162	Amount_total_1	species	Mass_Balance_1	0	0	milligram
163	Urine_1	compartment	Main_compartment_1	1	1	liter
164	Urine_drug_1	species	Urine_1	0	0	milligram
165	Liver_IC_S4_1	compartment	Main_compartment_1	1	0.252	liter
166	Liver_IC_S4_drug_1	species	Liver_IC_S4_1	0	0	milligram/liter
167	Liver_total_1	compartment	Main_compartment_1	1	1	liter
168	Liver_tissue_free_uM_1	species	Liver_total_1	0	0	micromole/liter
169	Liver_tissue_total_1	species	Liver_total_1	0	0	milligram/liter
170	Liver_blood_free_1	species	Liver_total_1	0	0	milligram/liter
171	Liver_tissue_total_uM_1	species	Liver_total_1	0	0	micromole/liter
172	Liver_blood_total_1	species	Liver_total_1	0	0	milligram/liter
173	convert_to_nmole_per_kg_1	compartment	Main_compartment_1	1	1	liter
174	Venous_nmole_1	species	convert_to_nmole_per_kg_1	0	0	nanomole
175	Artery_nmole_1	species	convert_to_nmole_per_kg_1	0	0	nanomole
176	Bone_nmole_1	species	convert_to_nmole_per_kg_1	0	0	nanomole
177	Adipose_nmole_1	species	convert_to_nmole_per_kg_1	0	0	nanomole
178	Muscle_nmole_1	species	convert_to_nmole_per_kg_1	0	0	nanomole

	Quantity Name	Type	Scope	Value	Initial Value	Units
179	Urine_nmole_1	species	convert_to_nmole_per_kg_1	0	0	nanomole
180	Liver_EC1_nmole_1	species	convert_to_nmole_per_kg_1	0	0	nanomole
181	Liver_IC1_nmole_1	species	convert_to_nmole_per_kg_1	0	0	nanomole
182	Bile_nmole_1	species	convert_to_nmole_per_kg_1	0	0	nanomole
183	Kidney_nmole_1	species	convert_to_nmole_per_kg_1	0	0	nanomole
184	Lung_nmole_1	species	convert_to_nmole_per_kg_1	0	0	nanomole
185	Metabolites_nmole_1	species	convert_to_nmole_per_kg_1	0	0	nanomole
186	Liver_IC2_nmole_1	species	convert_to_nmole_per_kg_1	0	0	nanomole
187	Liver_EC2_nmole_1	species	convert_to_nmole_per_kg_1	0	0	nanomole
188	Liver_EC3_nmole_1	species	convert_to_nmole_per_kg_1	0	0	nanomole
189	Liver_IC3_nmole_1	species	convert_to_nmole_per_kg_1	0	0	nanomole
190	Liver_IC4_nmole_1	species	convert_to_nmole_per_kg_1	0	0	nanomole
191	Liver_EC4_nmole_1	species	convert_to_nmole_per_kg_1	0	0	nanomole
192	Liver_EC5_nmole_1	species	convert_to_nmole_per_kg_1	0	0	nanomole
193	Liver_IC5_nmole_1	species	convert_to_nmole_per_kg_1	0	0	nanomole
194	Gut_nmole_1	species	convert_to_nmole_per_kg_1	0	0	nanomole
195	Spleen_nmole_1	species	convert_to_nmole_per_kg_1	0	0	nanomole
196	Skin_nmole_1	species	convert_to_nmole_per_kg_1	0	0	nanomole

	Quantity Name	Type	Scope	Value	Initial Value	Units
197	Brain_nmole_1	species	convert_to_nmole_per_kg_1	0	0	nanomole
198	Rest_nmole_1	species	convert_to_nmole_per_kg_1	0	0	nanomole
199	Heart_nmole_1	species	convert_to_nmole_per_kg_1	0	0	nanomole
200	Gut_Lumen	compartment	Main_compartment	1	1	liter
201	Gut_Lumen_drug	species	Gut_Lumen	0	0	milligram
202	Gut_Lumen_1	compartment	Main_compartment_1	1	1	liter
203	Gut_Lumen_drug_1	species	Gut_Lumen_1	0	0	milligram
204	STOMACH	compartment	Main_compartment	0.147	0.05	liter
205	X_STOMACH_SOLID	species	STOMACH	0	0	microgram
206	X_STOMACH_DISS	species	STOMACH	0	0	microgram
207	VDUO	compartment	Main_compartment	1	0.03435	liter
208	X_DUO_SOLID	species	VDUO	0	0	microgram
209	X_DUO_DISS	species	VDUO	0	0	microgram
210	VJEJ1	compartment	Main_compartment	1	0.0211	liter
211	X_JEJ1_SOLID	species	VJEJ1	0	0	microgram
212	X_JEJ1_DISS	species	VJEJ1	0	0	microgram
213	MDUO	compartment	Main_compartment	1	0.037454	liter
214	MEM_DUO	species	MDUO	0	0	microgram
215	MJEJ1	compartment	Main_compartment	1	0.073785	liter
216	MEM_JEJ1	species	MJEJ1	0	0	microgram
217	VJEJ2	compartment	Main_compartment	1	0.0211	liter
218	X_JEJ2_SOLID	species	VJEJ2	0	0	microgram
219	X_JEJ2_DISS	species	VJEJ2	0	0	microgram
220	MJEJ2	compartment	Main_compartment	1	0.051687	liter
221	MEM_JEJ2	species	MJEJ2	0	0	microgram

	Quantity Name	Type	Scope	Value	Initial Value	Units
222	VILL1	compartment	Main_compartment	1	0.0126	liter
223	X_ILL1_SOLID	species	VILL1	0	0	microgram
224	X_ILL1_DISS	species	VILL1	0	0	microgram
225	MILL1	compartment	Main_compartment	1	0.0412	liter
226	MEM_ILL1	species	MILL1	0	0	microgram
227	VILL2	compartment	Main_compartment	1	0.0126	liter
228	X_ILL2_SOLID	species	VILL2	0	0	microgram
229	X_ILL2_DISS	species	VILL2	0	0	microgram
230	MILL2	compartment	Main_compartment	1	0.0412	liter
231	MEM_ILL2	species	MILL2	0	0	microgram
232	VILL3	compartment	Main_compartment	1	0.0126	liter
233	X_ILL3_SOLID	species	VILL3	0	0	microgram
234	X_ILL3_DISS	species	VILL3	0	0	microgram
235	MILL3	compartment	Main_compartment	1	0.04045	liter
236	MEM_ILL3	species	MILL3	0	0	microgram
237	VILL4	compartment	Main_compartment	1	0.0126	liter
238	X_ILL4_SOLID	species	VILL4	0	0	microgram
239	X_ILL4_DISS	species	VILL4	0	0	microgram
240	MILL4	compartment	Main_compartment	1	0.038952	liter
241	MEM_ILL4	species	MILL4	0	0	microgram
242	Colon	compartment	Main_compartment	1	1	liter
243	X_CECUM_SOLID	species	Colon	0	0	microgram
244	X_CECUM_DISS	species	Colon	0	0	microgram
245	VillousDUO	compartment	Main_compartment	1	0.0057016	liter
246	Villous_DUO	species	VillousDUO	0	0	microgram
247	VillousJEJ1	compartment	Main_compartment	1	0.011232	liter
248	Villous_JEJ1	species	VillousJEJ1	0	0	microgram
249	VillousJEJ2	compartment	Main_compartment	1	0.0078682	liter
250	Villous_JEJ2	species	VillousJEJ2	0	0	microgram

	Quantity Name	Type	Scope	Value	Initial Value	Units
251	VillousILL1	compartment	Main_compartment	1	0.0062718	liter
252	Villous_ILL1	species	VillousILL1	0	0	microgram
253	VillousILL2	compartment	Main_compartment	1	0.0062718	liter
254	Villous_ILL2	species	VillousILL2	0	0	microgram
255	VillousILL3	compartment	Main_compartment	1	0.0061578	liter
256	Villous_ILL3	species	VillousILL3	0	0	microgram
257	VillousILL4	compartment	Main_compartment	1	0.0059297	liter
258	Villous_ILL4	species	VillousILL4	0	0	microgram
259	Liver	compartment	Main_compartment	1	1.603	liter
260	Liver_drug	species	Liver	0	0	milligram/liter
261	Serosa	compartment	Main_compartment	1	0.14284	liter
262	Serosa_drug	species	Serosa	0	0	milligram/liter
263	STOMACH_1	compartment	Main_compartment_1	0.147	0.05	liter
264	X_STOMACH_SOLID_1	species	STOMACH_1	0	0	microgram
265	X_STOMACH_DISS_1	species	STOMACH_1	0	0	microgram
266	VDUO_1	compartment	Main_compartment_1	1	0.03435	liter
267	X_DUO_SOLID_1	species	VDUO_1	0	0	microgram
268	X_DUO_DISS_1	species	VDUO_1	0	0	microgram
269	VJEJ1_1	compartment	Main_compartment_1	1	0.0211	liter
270	X_JEJ1_SOLID_1	species	VJEJ1_1	0	0	microgram
271	X_JEJ1_DISS_1	species	VJEJ1_1	0	0	microgram
272	MDUO_1	compartment	Main_compartment_1	1	0.037454	liter
273	MEM_DUO_1	species	MDUO_1	0	0	microgram
274	MJEJ1_1	compartment	Main_compartment_1	1	0.073785	liter
275	MEM_JEJ1_1	species	MJEJ1_1	0	0	microgram
276	VJEJ2_1	compartment	Main_compartment_1	1	0.0211	liter
277	X_JEJ2_SOLID_1	species	VJEJ2_1	0	0	microgram
278	X_JEJ2_DISS_1	species	VJEJ2_1	0	0	microgram

	Quantity Name	Type	Scope	Value	Initial Value	Units
279	MJEJ2_1	compartment	Main_compartment_1	1	0.051687	liter
280	MEM_JEJ2_1	species	MJEJ2_1	0	0	microgram
281	VILL1_1	compartment	Main_compartment_1	1	0.0126	liter
282	X_ILL1_SOLID_1	species	VILL1_1	0	0	microgram
283	X_ILL1_DISS_1	species	VILL1_1	0	0	microgram
284	MILL1_1	compartment	Main_compartment_1	1	0.0412	liter
285	MEM_ILL1_1	species	MILL1_1	0	0	microgram
286	VILL2_1	compartment	Main_compartment_1	1	0.0126	liter
287	X_ILL2_SOLID_1	species	VILL2_1	0	0	microgram
288	X_ILL2_DISS_1	species	VILL2_1	0	0	microgram
289	MILL2_1	compartment	Main_compartment_1	1	0.0412	liter
290	MEM_ILL2_1	species	MILL2_1	0	0	microgram
291	VILL3_1	compartment	Main_compartment_1	1	0.0126	liter
292	X_ILL3_SOLID_1	species	VILL3_1	0	0	microgram
293	X_ILL3_DISS_1	species	VILL3_1	0	0	microgram
294	MILL3_1	compartment	Main_compartment_1	1	0.04045	liter
295	MEM_ILL3_1	species	MILL3_1	0	0	microgram
296	VILL4_1	compartment	Main_compartment_1	1	0.0126	liter
297	X_ILL4_SOLID_1	species	VILL4_1	0	0	microgram
298	X_ILL4_DISS_1	species	VILL4_1	0	0	microgram
299	MILL4_1	compartment	Main_compartment_1	1	0.038952	liter
300	MEM_ILL4_1	species	MILL4_1	0	0	microgram
301	Colon_1	compartment	Main_compartment_1	1	1	liter
302	X_CECUM_SOLID_1	species	Colon_1	0	0	microgram
303	X_CECUM_DISS_1	species	Colon_1	0	0	microgram
304	VillousDUO_1	compartment	Main_compartment_1	1	0.0057016	liter
305	Villous_DUO_1	species	VillousDUO_1	0	0	microgram

	Quantity Name	Type	Scope	Value	Initial Value	Units
306	VillousJEJ2_1	compartment	Main_compartment_1	1	0.0078682	liter
307	Villous_JEJ2_1	species	VillousJEJ2_1	0	0	microgram
308	VillousILL1_1	compartment	Main_compartment_1	1	0.0062718	liter
309	Villous_ILL1_1	species	VillousILL1_1	0	0	microgram
310	VillousILL2_1	compartment	Main_compartment_1	1	0.0062718	liter
311	Villous_ILL2_1	species	VillousILL2_1	0	0	microgram
312	VillousILL3_1	compartment	Main_compartment_1	1	0.0061578	liter
313	Villous_ILL3_1	species	VillousILL3_1	0	0	microgram
314	VillousILL4_1	compartment	Main_compartment_1	1	0.0059297	liter
315	Villous_ILL4_1	species	VillousILL4_1	0	0	microgram
316	VillousJEJ1_1	compartment	Main_compartment_1	1	0.011232	liter
317	Villous_JEJ1_1	species	VillousJEJ1_1	0	0	microgram
318	Liver_1	compartment	Main_compartment_1	1	1.603	liter
319	Liver_drug_1	species	Liver_1	0	0	milligram/liter
320	Serosa_1	compartment	Main_compartment_1	1	0.14284	liter
321	Serosa_drug_1	species	Serosa_1	0	0	milligram/liter
322	Qlung	parameter	PBPK_MADAM	1	336	liter/hour
323	k_lung_artery	parameter	PBPK_MADAM	1	518.5185	liter/hour
324	k_venous_lung	parameter	PBPK_MADAM	1	336	liter/hour
325	Qkidney	parameter	PBPK_MADAM	1	63	liter/hour
326	k_artery_kidney	parameter	PBPK_MADAM	1	63	liter/hour
327	Qbrain	parameter	PBPK_MADAM	1	42	liter/hour
328	k_artery_brain	parameter	PBPK_MADAM	1	42	liter/hour
329	Qmuscle	parameter	PBPK_MADAM	1	58.8	liter/hour
330	k_artery_muscle	parameter	PBPK_MADAM	1	58.8	liter/hour

	Quantity Name	Type	Scope	Value	Initial Value	Units
331	Qadipose	parameter	PBPK_MADAM	1	16.8	liter/hour
332	k_artery_adipos	parameter	PBPK_MADAM	1	16.8	liter/hour
333	Qskin	parameter	PBPK_MADAM	1	16.8	liter/hour
334	k_artery_skin	parameter	PBPK_MADAM	1	16.8	liter/hour
335	Qbone	parameter	PBPK_MADAM	1	16.8	liter/hour
336	k_artery_bone	parameter	PBPK_MADAM	1	16.8	liter/hour
337	Qrest	parameter	PBPK_MADAM	1	4.2	liter/hour
338	k_artery_rest	parameter	PBPK_MADAM	1	4.2	liter/hour
339	k_kidney_venous	parameter	PBPK_MADAM	1	72.7679	liter/hour
340	k_brain_venous	parameter	PBPK_MADAM	1	30.7322	liter/hour
341	k_muscle_venous	parameter	PBPK_MADAM	1	3.9421	liter/hour
342	k_adipos_venous	parameter	PBPK_MADAM	1	22.9954	liter/hour
343	Qheart	parameter	PBPK_MADAM	1	12.6	liter/hour
344	k_heart_venous	parameter	PBPK_MADAM	1	0.22189	liter/hour
345	k_skin_venous	parameter	PBPK_MADAM	1	18.9708	liter/hour
346	k_bone_venous	parameter	PBPK_MADAM	1	13.9966	liter/hour
347	k_rest_venous	parameter	PBPK_MADAM	1	5.7489	liter/hour
348	Q_artery_spleen	parameter	PBPK_MADAM	1	8.4	liter/hour
349	k_artery_spleen	parameter	PBPK_MADAM	1	8.4	liter/hour
350	Q_artery_gut	parameter	PBPK_MADAM	1	71.4	liter/hour
351	k_artery_gut	parameter	PBPK_MADAM	1	71.4	liter/hour
352	Q_artery_liver	parameter	PBPK_MADAM	1	4.2	liter/hour
353	Q_spleen_liver	parameter	PBPK_MADAM	1	8.4	liter/hour
354	k_spleen_liver	parameter	PBPK_MADAM	1	9.425	liter/hour
355	Q_gut_liver	parameter	PBPK_MADAM	1	71.4	liter/hour

	Quantity Name	Type	Scope	Value	Initial Value	Units
356	k_gut_liver	parameter	PBPK_MADAM	1	61.4428	liter/hour
357	k_Liver_EC_S1_Liver_IC_S1	parameter	PBPK_MADAM	1	1894.1175	liter/hour
358	k_Liver_IC_S1_Liver_EC_S1	parameter	PBPK_MADAM	0	4.029	liter/hour
359	k_Liver_EC_S2_Liver_IC_S2	parameter	PBPK_MADAM	1	1894.1175	liter/hour
360	k_Liver_IC_S2_Liver_EC_S2	parameter	PBPK_MADAM	0	4.029	liter/hour
361	k_Liver_IC_S4_Liver_EC_S4	parameter	PBPK_MADAM	0	4.029	liter/hour
362	k_Liver_EC_S4_Liver_IC_S4	parameter	PBPK_MADAM	1	1894.1175	liter/hour
363	k_Liver_IC_S5_Liver_EC_S5	parameter	PBPK_MADAM	0	4.029	liter/hour
364	k_Liver_EC_S5_Liver_IC_S5	parameter	PBPK_MADAM	1	1894.1175	liter/hour
365	k_Liver_EC_S3_Liver_IC_S3	parameter	PBPK_MADAM	1	1894.1175	liter/hour
366	Q_li	parameter	PBPK_MADAM	1	84	liter/hour
367	k_Liver_EC_S1_Liver_EC_S2	parameter	PBPK_MADAM	1	84	liter/hour
368	k_Liver_EC_S2_Liver_EC_S3	parameter	PBPK_MADAM	1	84	liter/hour
369	k_Liver_EC_S3_Liver_EC_S4	parameter	PBPK_MADAM	1	84	liter/hour
370	k_Liver_EC_S4_Liver_EC_S5	parameter	PBPK_MADAM	1	84	liter/hour
371	k_Liver_IC_S5_Bile	parameter	PBPK_MADAM	1	0	liter/hour
372	k_Liver_IC_S4_Bile	parameter	PBPK_MADAM	1	0	liter/hour
373	k_Liver_IC_S3_Bile	parameter	PBPK_MADAM	1	0	liter/hour
374	k_Liver_IC_S2_Bile	parameter	PBPK_MADAM	1	0	liter/hour
375	k_Liver_IC_S1_Bile	parameter	PBPK_MADAM	1	0	liter/hour

	Quantity Name	Type	Scope	Value	Initial Value	Units
376	k_Liver_IC_S1_Metabolites	parameter	PBPK_MADAM	1	0.3237	liter/hour
377	k_Liver_IC_S2_Metabolites	parameter	PBPK_MADAM	1	0.3237	liter/hour
378	k_Liver_IC_S3_Metabolites	parameter	PBPK_MADAM	1	0.3237	liter/hour
379	k_Liver_IC_S4_Metabolites	parameter	PBPK_MADAM	1	0.3237	liter/hour
380	k_Liver_IC_S5_Metabolites	parameter	PBPK_MADAM	1	0.3237	liter/hour
381	drug_fB	parameter	PBPK_MADAM	1	0.66355	dimensionless
382	drug_fuLiver	parameter	PBPK_MADAM	0.018	0.42635	dimensionless
383	Kp_kidney	parameter	PBPK_MADAM	0.134	0.92637	dimensionless
384	drug_BRP	parameter	PBPK_MADAM	0.65	1.07	dimensionless
385	Kp_heart	parameter	PBPK_MADAM	0.16	60.76	dimensionless
386	Kp_gut	parameter	PBPK_MADAM	0.165	1.2434	dimensionless
387	Kp_brain	parameter	PBPK_MADAM	0.057	1.4623	dimensionless
388	Kp_bone	parameter	PBPK_MADAM	0.108	1.2843	dimensionless
389	Kp_adipose	parameter	PBPK_MADAM	0.047	0.78172	dimensionless
390	Kp_muscle	parameter	PBPK_MADAM	0.038	15.96	dimensionless
391	Kp_rest	parameter	PBPK_MADAM	0.12	0.78172	dimensionless
392	Kp_lung	parameter	PBPK_MADAM	0.21	0.69336	dimensionless
393	drug_CLrenal	parameter	PBPK_MADAM	0	9.66	liter/hour
394	drug_fuplasma	parameter	PBPK_MADAM	0.0382	0.71	dimensionless
395	Kp_spleen	parameter	PBPK_MADAM	0.1	0.95363	dimensionless
396	k_Liver_Venous	parameter	PBPK_MADAM	1	79.4828	liter/hour
397	k_artery_heart	parameter	PBPK_MADAM	1	12.6	liter/hour
398	k_Liver_IC_S3_Liver__EC_S3	parameter	PBPK_MADAM	0	4.029	liter/hour
399	k_artery_liver	parameter	PBPK_MADAM	1	4.2	liter/hour

	Quantity Name	Type	Scope	Value	Initial Value	Units
400	drug_PSinfg	parameter	PBPK_MADAM	471	471	microliter/minute
401	phys_HPGL	parameter	PBPK_MADAM	122	125	1/gram
402	switch_SFinf	parameter	PBPK_MADAM	3.196	3.196	dimensionless
403	Specific_volume	parameter	PBPK_MADAM	1	1	milliliter/gram
404	drug_PSbileg	parameter	PBPK_MADAM	2.5	0	microliter/minute
405	switch_SFbile	parameter	PBPK_MADAM	0.23114	1	dimensionless
406	drug_PSdifg	parameter	PBPK_MADAM	5	5	microliter/minute
407	switch_SFdiff	parameter	PBPK_MADAM	1.5621	1	dimensionless
408	drug_CLmetg	parameter	PBPK_MADAM	19	0.37	microliter/minute
409	drug_HLM_CLint	parameter	PBPK_MADAM	0	0	milliliter/minute/milligram
410	phys_MPGL	parameter	PBPK_MADAM	45	45	milligram/gram
411	drug_fumic	parameter	PBPK_MADAM	1	0.92106	dimensionless
412	drug_funic	parameter	PBPK_MADAM	1	0.92106	dimensionless
413	Kp_skin	parameter	PBPK_MADAM	0.28	0.94756	dimensionless
414	phys_BW	parameter	PBPK_MADAM	1	70	kilogram
415	switch_SFrenal	parameter	PBPK_MADAM	1	1	dimensionless
416	k_venous_urine_CLR	parameter	PBPK_MADAM	1	9.66	liter/hour
417	Qtestes	parameter	PBPK_MADAM	1	0	liter/hour
418	k_artery_testes	parameter	PBPK_MADAM	1	0	liter/hour
419	Kp_testes	parameter	PBPK_MADAM	1	1	dimensionless
420	k_testes_venous	parameter	PBPK_MADAM	1	0	liter/hour
421	k_Liver_IC_S2_Liver_EC_S2_efflux	parameter	PBPK_MADAM	1	0	liter/hour
422	k_Liver_IC_S3_Liver_EC_S3_efflux	parameter	PBPK_MADAM	1	0	liter/hour

	Quantity Name	Type	Scope	Value	Initial Value	Units
423	k_Liver_IC_S4_Liver_EC_S4_efflux	parameter	PBPK_MADAM	1	0	liter/hour
424	k_Liver_IC_S5_Liver_EC_S5_efflux	parameter	PBPK_MADAM	1	0	liter/hour
425	k_Liver_IC_S1_Liver_EC_S1_efflux	parameter	PBPK_MADAM	1	0	liter/hour
426	drug_fa	parameter	PBPK_MADAM	1	1	dimensionless
427	drug_dose_rate_IV	parameter	PBPK_MADAM	1	1	milligram/hour
428	phys_Normalized_weight_adipose	parameter	PBPK_MADAM	92	197	gram/kilogram
429	phys_Normalized_weight_lung	parameter	PBPK_MADAM	5	8	gram/kilogram
430	phys_Normalized_weight_kidney	parameter	PBPK_MADAM	4	5	gram/kilogram
431	phys_Normalized_weight_brain	parameter	PBPK_MADAM	21	21	gram/kilogram
432	phys_Normalized_weight_muscle	parameter	PBPK_MADAM	409	416	gram/kilogram
433	phys_Normalized_weight_heart	parameter	PBPK_MADAM	4	5	gram/kilogram
434	phys_Normalized_weight_skin	parameter	PBPK_MADAM	85	41	gram/kilogram
435	phys_Normalized_weight_bone	parameter	PBPK_MADAM	203	158	gram/kilogram
436	phys_Normalized_weight_remainder	parameter	PBPK_MADAM	100	100	gram/kilogram
437	phys_Normalized_weight_spleen	parameter	PBPK_MADAM	2	3	gram/kilogram
438	phys_Normalized_weight_gut	parameter	PBPK_MADAM	47	18	gram/kilogram
439	phys_Normalized_weight_liver_blood	parameter	PBPK_MADAM	5	4.9	gram/kilogram
440	phys_Normalized_weight_liver_tissue	parameter	PBPK_MADAM	20	18	gram/kilogram

	Quantity Name	Type	Scope	Value	Initial Value	Units
441	phys_Normalized_weight_artery	parameter	PBPK_MADAM	22.4	25.7	gram/kilogram
442	phys_Normalized_weight_venous	parameter	PBPK_MADAM	45.2	51.4	gram/kilogram
443	phys_Normalized_weight_testes	parameter	PBPK_MADAM	1	1	gram/kilogram
444	phys_Normalized_Q_adipose	parameter	PBPK_MADAM	15	4	milliliter/minute/kilogram
445	phys_Normalized_Q_lung	parameter	PBPK_MADAM	200	80	milliliter/minute/kilogram
446	phys_Normalized_Q_brain	parameter	PBPK_MADAM	11	10	milliliter/minute/kilogram
447	phys_Normalized_Q_muscle	parameter	PBPK_MADAM	65	14	milliliter/minute/kilogram
448	phys_Normalized_Q_heart	parameter	PBPK_MADAM	12	3	milliliter/minute/kilogram
449	phys_Normalized_Q_bone	parameter	PBPK_MADAM	19	4	milliliter/minute/kilogram
450	phys_Normalized_Q_remainder	parameter	PBPK_MADAM	1	1	milliliter/minute/kilogram
451	phys_Normalized_Q_gut	parameter	PBPK_MADAM	17	17	milliliter/minute/kilogram
452	phys_Normalized_Q_spleen	parameter	PBPK_MADAM	2	2	milliliter/minute/kilogram
453	phys_Normalized_Q_liver	parameter	PBPK_MADAM	26	20	milliliter/minute/kilogram
454	phys_Normalized_Q_kidney	parameter	PBPK_MADAM	25	15	milliliter/minute/kilogram
455	phys_Normalized_Q_skin	parameter	PBPK_MADAM	20	4	milliliter/minute/kilogram
456	phys_Normalized_Q_testes	parameter	PBPK_MADAM	0	0	milliliter/minute/kilogram
457	switch_SFmet	parameter	PBPK_MADAM	0.32509	1	dimensionless
458	drug_Km_uptake	parameter	PBPK_MADAM	1	1	micromole/liter
459	switch_Vmax_uptake	parameter	PBPK_MADAM	0	0	micromole/kilogram/hour
460	drug_molar_mass	parameter	PBPK_MADAM	712800	780940	milligram/mole
461	drug_Km_met	parameter	PBPK_MADAM	1	1	micromole/liter

	Quantity Name	Type	Scope	Value	Initial Value	Units
462	switch_Vmax_met	parameter	PBPK_MADAM	0	0	micromole/hour/kilogram
463	drug_Kp_adipose_raw	parameter	PBPK_MADAM	0.45	0.78172	dimensionless
464	switch_SFKp	parameter	PBPK_MADAM	0.51837	1	dimensionless
465	drug_Kp_bone_raw	parameter	PBPK_MADAM	0.6	1.2843	dimensionless
466	drug_Kp_brain_raw	parameter	PBPK_MADAM	0.32	1.4623	dimensionless
467	drug_Kp_gut_raw	parameter	PBPK_MADAM	0.47	1.2434	dimensionless
468	drug_Kp_heart_raw	parameter	PBPK_MADAM	0.2	60.76	dimensionless
469	drug_Kp_kidney_raw	parameter	PBPK_MADAM	0.26	0.92637	dimensionless
470	drug_Kp_lung_raw	parameter	PBPK_MADAM	0.4	0.69336	dimensionless
471	drug_Kp_muscle_raw	parameter	PBPK_MADAM	0.05	15.96	dimensionless
472	drug_Kp_rest_raw	parameter	PBPK_MADAM	0.0065	0.78172	dimensionless
473	drug_Kp_skin_raw	parameter	PBPK_MADAM	0.37	0.94756	dimensionless
474	drug_Kp_spleen_raw	parameter	PBPK_MADAM	0.35	0.95363	dimensionless
475	drug_Kp_testes_raw	parameter	PBPK_MADAM	1	1	dimensionless
476	k_transit	parameter	PBPK_MADAM	0	0	1/(hour)
477	drug_t_lag	parameter	PBPK_MADAM	1	1	hour
478	drug_k_oral	parameter	PBPK_MADAM	3.381	3.381	1/(hour)
479	switch_SFeff	parameter	PBPK_MADAM	0	0	dimensionless
480	nanomole_per_mole	parameter	PBPK_MADAM	1000000000	1000000000	nanomole/mole
481	kilogram	parameter	PBPK_MADAM	1	1	kilogram
482	k_venous_urine_GFR	parameter	PBPK_MADAM	1	0	liter/hour
483	drug_GFR	parameter	PBPK_MADAM	1	0	milliliter/minute
484	drug_CLeffluxHep	parameter	PBPK_MADAM	0	0	microliter/minute
485	drug_FR	parameter	PBPK_MADAM	0	0	dimensionless
486	switch_slow_dist_Kp	parameter	PBPK_MADAM	1	1	dimensionless

	Quantity Name	Type	Scope	Value	Initial Value	Units
487	Qlung_1	parameter	PBPK_MADAM	1	336	liter/hour
488	k_lung_artery_1	parameter	PBPK_MADAM	1	1053.3017	liter/hour
489	k_venous_lung_1	parameter	PBPK_MADAM	1	336	liter/hour
490	Qkidney_1	parameter	PBPK_MADAM	1	63	liter/hour
491	k_artery_kidney_1	parameter	PBPK_MADAM	1	63	liter/hour
492	Qbrain_1	parameter	PBPK_MADAM	1	42	liter/hour
493	k_artery_brain_1	parameter	PBPK_MADAM	1	42	liter/hour
494	Qmuscle_1	parameter	PBPK_MADAM	1	58.8	liter/hour
495	k_artery_muscle_1	parameter	PBPK_MADAM	1	58.8	liter/hour
496	Qadipose_1	parameter	PBPK_MADAM	1	16.8	liter/hour
497	k_artery_adipos_1	parameter	PBPK_MADAM	1	16.8	liter/hour
498	Qskin_1	parameter	PBPK_MADAM	1	16.8	liter/hour
499	k_artery_skin_1	parameter	PBPK_MADAM	1	16.8	liter/hour
500	Qbone_1	parameter	PBPK_MADAM	1	16.8	liter/hour
501	k_artery_bone_1	parameter	PBPK_MADAM	1	16.8	liter/hour
502	Qrest_1	parameter	PBPK_MADAM	1	4.2	liter/hour
503	k_artery_rest_1	parameter	PBPK_MADAM	1	4.2	liter/hour
504	k_kidney_venous_1	parameter	PBPK_MADAM	1	303.837	liter/hour
505	k_brain_venous_1	parameter	PBPK_MADAM	1	164.5784	liter/hour
506	k_muscle_venous_1	parameter	PBPK_MADAM	1	1474.6224	liter/hour
507	k_adipos_venous_1	parameter	PBPK_MADAM	1	46.8134	liter/hour
508	Qheart_1	parameter	PBPK_MADAM	1	12.6	liter/hour
509	k_heart_venous_1	parameter	PBPK_MADAM	1	78.9976	liter/hour
510	k_skin_venous_1	parameter	PBPK_MADAM	1	56.9352	liter/hour
511	k_bone_venous_1	parameter	PBPK_MADAM	1	35.1101	liter/hour

	Quantity Name	Type	Scope	Value	Initial Value	Units
512	k_rest_venous_1	parameter	PBPK_MADAM	1	420	liter/hour
513	Q_artery_spleen_1	parameter	PBPK_MADAM	1	8.4	liter/hour
514	k_artery_spleen_1	parameter	PBPK_MADAM	1	8.4	liter/hour
515	Q_artery_gut_1	parameter	PBPK_MADAM	1	71.4	liter/hour
516	k_artery_gut_1	parameter	PBPK_MADAM	1	71.4	liter/hour
517	Q_artery_liver_1	parameter	PBPK_MADAM	1	4.2	liter/hour
518	Q_spleen_liver_1	parameter	PBPK_MADAM	1	8.4	liter/hour
519	k_spleen_liver_1	parameter	PBPK_MADAM	1	30.0943	liter/hour
520	Q_gut_liver_1	parameter	PBPK_MADAM	1	71.4	liter/hour
521	k_gut_liver_1	parameter	PBPK_MADAM	1	190.4907	liter/hour
522	k_Liver_EC_S1_Liver_IC_S1_1	parameter	PBPK_MADAM	1	168.0698	liter/hour
523	k_Liver_IC_S1_Liver_EC_S1_1	parameter	PBPK_MADAM	0	0.26572	liter/hour
524	k_Liver_EC_S2_Liver_IC_S2_1	parameter	PBPK_MADAM	1	168.0698	liter/hour
525	k_Liver_IC_S2_Liver_EC_S2_1	parameter	PBPK_MADAM	0	0.26572	liter/hour
526	k_Liver_IC_S4_Liver_EC_S4_1	parameter	PBPK_MADAM	0	0.26572	liter/hour
527	k_Liver_EC_S4_Liver_IC_S4_1	parameter	PBPK_MADAM	1	168.0698	liter/hour
528	k_Liver_IC_S5_Liver_EC_S5_1	parameter	PBPK_MADAM	0	0.26572	liter/hour
529	k_Liver_EC_S5_Liver_IC_S5_1	parameter	PBPK_MADAM	1	168.0698	liter/hour
530	k_Liver_EC_S3_Liver_IC_S3_1	parameter	PBPK_MADAM	1	168.0698	liter/hour
531	Q_li_1	parameter	PBPK_MADAM	1	84	liter/hour
532	k_Liver_EC_S1_Liver_EC_S2_1	parameter	PBPK_MADAM	1	84	liter/hour

	Quantity Name	Type	Scope	Value	Initial Value	Units
533	k_Liver_EC_S2_Liver_EC_S3_1	parameter	PBPK_MADAM	1	84	liter/hour
534	k_Liver_EC_S3_Liver_EC_S4_1	parameter	PBPK_MADAM	1	84	liter/hour
535	k_Liver_EC_S4_Liver_EC_S5_1	parameter	PBPK_MADAM	1	84	liter/hour
536	k_Liver_IC_S5_Bile_1	parameter	PBPK_MADAM	1	0.019658	liter/hour
537	k_Liver_IC_S4_Bile_1	parameter	PBPK_MADAM	1	0.019658	liter/hour
538	k_Liver_IC_S3_Bile_1	parameter	PBPK_MADAM	1	0.019658	liter/hour
539	k_Liver_IC_S2_Bile_1	parameter	PBPK_MADAM	1	0.019658	liter/hour
540	k_Liver_IC_S1_Bile_1	parameter	PBPK_MADAM	1	0.019658	liter/hour
541	k_Liver_IC_S1_Metabolites_1	parameter	PBPK_MADAM	1	0.21013	liter/hour
542	k_Liver_IC_S2_Metabolites_1	parameter	PBPK_MADAM	1	0.21013	liter/hour
543	k_Liver_IC_S3_Metabolites_1	parameter	PBPK_MADAM	1	0.21013	liter/hour
544	k_Liver_IC_S4_Metabolites_1	parameter	PBPK_MADAM	1	0.21013	liter/hour
545	k_Liver_IC_S5_Metabolites_1	parameter	PBPK_MADAM	1	0.21013	liter/hour
546	drug_fB_1	parameter	PBPK_MADAM	1	0.058769	dimensionless
547	drug_fuLiver_1	parameter	PBPK_MADAM	0.018	0.018	dimensionless
548	Kp_kidney_1	parameter	PBPK_MADAM	0.134	0.13478	dimensionless
549	drug_BRP_1	parameter	PBPK_MADAM	0.65	0.65	dimensionless
550	Kp_heart_1	parameter	PBPK_MADAM	0.16	0.10367	dimensionless
551	Kp_gut_1	parameter	PBPK_MADAM	0.165	0.24363	dimensionless
552	Kp_brain_1	parameter	PBPK_MADAM	0.057	0.16588	dimensionless
553	Kp_bone_1	parameter	PBPK_MADAM	0.108	0.31102	dimensionless

	Quantity Name	Type	Scope	Value	Initial Value	Units
554	Kp_adipose_1	parameter	PBPK_MADAM	0.047	0.23327	dimensionless
555	Kp_muscle_1	parameter	PBPK_MADAM	0.038	0.025919	dimensionless
556	Kp_rest_1	parameter	PBPK_MADAM	0.12	0.0065	dimensionless
557	Kp_lung_1	parameter	PBPK_MADAM	0.21	0.20735	dimensionless
558	drug_CLrenal_1	parameter	PBPK_MADAM	0	0	liter/hour
559	drug_fuplasma_1	parameter	PBPK_MADAM	0.0382	0.0382	dimensionless
560	Kp_spleen_1	parameter	PBPK_MADAM	0.1	0.18143	dimensionless
561	k_Liver_EC_S5_Venous_1	parameter	PBPK_MADAM	1	84	liter/hour
562	k_artery_heart_1	parameter	PBPK_MADAM	1	12.6	liter/hour
563	k_Liver_IC_S3_Liver__EC_S3_1	parameter	PBPK_MADAM	0	0.26572	liter/hour
564	k_artery_liver_1	parameter	PBPK_MADAM	1	4.2	liter/hour
565	drug_PSinfg_1	parameter	PBPK_MADAM	471	471	microliter/minute
566	phys_HPGL_1	parameter	PBPK_MADAM	122	122	1/gram
567	switch_SFinf_1	parameter	PBPK_MADAM	3.196	3.196	dimensionless
568	drug_PSbileg_1	parameter	PBPK_MADAM	2.5	2.5	microliter/minute
569	switch_SFbile_1	parameter	PBPK_MADAM	0.23114	0.23114	dimensionless
570	drug_PSdifg_1	parameter	PBPK_MADAM	5	5	microliter/minute
571	switch_SFdiff_1	parameter	PBPK_MADAM	1.5621	1.5621	dimensionless
572	drug_CLmetg_1	parameter	PBPK_MADAM	19	19	microliter/minute
573	drug_HLM_CLint_1	parameter	PBPK_MADAM	0	0	milliliter/minute/milligram
574	drug_fumic_1	parameter	PBPK_MADAM	1	1	dimensionless
575	drug_funic_1	parameter	PBPK_MADAM	1	1	dimensionless
576	Kp_skin_1	parameter	PBPK_MADAM	0.28	0.1918	dimensionless
577	switch_SFrenal_1	parameter	PBPK_MADAM	1	1	dimensionless

	Quantity Name	Type	Scope	Value	Initial Value	Units
578	k_venous_urine_CLR_1	parameter	PBPK_MADAM	1	0	liter/hour
579	Qtestes_1	parameter	PBPK_MADAM	1	0	liter/hour
580	k_artery_testes_1	parameter	PBPK_MADAM	1	0	liter/hour
581	Kp_testes_1	parameter	PBPK_MADAM	1	0.51837	dimensionless
582	k_testes_venous_1	parameter	PBPK_MADAM	1	0	liter/hour
583	k_Liver_IC_S2_Liver_EC_S2_efflux_1	parameter	PBPK_MADAM	1	0	liter/hour
584	k_Liver_IC_S3_Liver_EC_S3_efflux_1	parameter	PBPK_MADAM	1	0	liter/hour
585	k_Liver_IC_S4_Liver_EC_S4_efflux_1	parameter	PBPK_MADAM	1	0	liter/hour
586	k_Liver_IC_S5_Liver_EC_S5_efflux_1	parameter	PBPK_MADAM	1	0	liter/hour
587	k_Liver_IC_S1_Liver_EC_S1_efflux_1	parameter	PBPK_MADAM	1	0	liter/hour
588	drug_fa_1	parameter	PBPK_MADAM	1	1	dimensionless
589	drug_dose_rate_IV_1	parameter	PBPK_MADAM	1	1	milligram/hour
590	switch_SFmet_1	parameter	PBPK_MADAM	0.32509	0.32509	dimensionless
591	drug_Km_uptake_1	parameter	PBPK_MADAM	1	1	micromole/liter
592	switch_Vmax_uptake_1	parameter	PBPK_MADAM	0	0	micromole/kilogram/hour
593	drug_molar_mass_1	parameter	PBPK_MADAM	712800	712800	milligram/mole
594	drug_Km_met_1	parameter	PBPK_MADAM	1	1	micromole/liter
595	switch_Vmax_met_1	parameter	PBPK_MADAM	0	0	micromole/hour/kilogram
596	drug_Kp_adipose_raw_1	parameter	PBPK_MADAM	0.45	0.45	dimensionless
597	switch_SFKp_1	parameter	PBPK_MADAM	0.51837	0.51837	dimensionless
598	drug_Kp_bone_raw_1	parameter	PBPK_MADAM	0.6	0.6	dimensionless
599	drug_Kp_brain_raw_1	parameter	PBPK_MADAM	0.32	0.32	dimensionless
600	drug_Kp_gut_raw_1	parameter	PBPK_MADAM	0.47	0.47	dimensionless

	Quantity Name	Type	Scope	Value	Initial Value	Units
601	drug_Kp_heart_raw_1	parameter	PBPK_MADAM	0.2	0.2	dimensionless
602	drug_Kp_kidney_raw_1	parameter	PBPK_MADAM	0.26	0.26	dimensionless
603	drug_Kp_lung_raw_1	parameter	PBPK_MADAM	0.4	0.4	dimensionless
604	drug_Kp_muscle_raw_1	parameter	PBPK_MADAM	0.05	0.05	dimensionless
605	drug_Kp_rest_raw_1	parameter	PBPK_MADAM	0.0065	0.0065	dimensionless
606	drug_Kp_skin_raw_1	parameter	PBPK_MADAM	0.37	0.37	dimensionless
607	drug_Kp_spleen_raw_1	parameter	PBPK_MADAM	0.35	0.35	dimensionless
608	drug_Kp_testes_raw_1	parameter	PBPK_MADAM	1	1	dimensionless
609	k_transit_1	parameter	PBPK_MADAM	0	0	1/(hour)
610	drug_k_oral_1	parameter	PBPK_MADAM	10	10	1/(hour)
611	switch_SFeff_1	parameter	PBPK_MADAM	0	0	dimensionless
612	k_venous_urine_GFR_1	parameter	PBPK_MADAM	1	0	liter/hour
613	drug_GFR_1	parameter	PBPK_MADAM	1	0	milliliter/minute
614	drug_CLeffluxHep_1	parameter	PBPK_MADAM	0	0	microliter/minute
615	drug_FR_1	parameter	PBPK_MADAM	0	0	dimensionless
616	switch_slow_dist_Kp_1	parameter	PBPK_MADAM	1	1	dimensionless
617	drug_uptake_Ki	parameter	PBPK_MADAM	0.226	0.226	micromole/liter
618	drug_dose_amount_IV	parameter	PBPK_MADAM	0	1	milligram
619	drug_dose_amount_PO_1	parameter	PBPK_MADAM	0	0	milligram
620	drug_dose_amount_IV_1	parameter	PBPK_MADAM	0	0	milligram
621	drug_dose_amount_PO	parameter	PBPK_MADAM	0	0	milligram
622	uptake_inhib_S1	parameter	PBPK_MADAM	1	1	dimensionless
623	uptake_inhib_S2	parameter	PBPK_MADAM	1	1	dimensionless
624	uptake_inhib_S3	parameter	PBPK_MADAM	1	1	dimensionless
625	uptake_inhib_S4	parameter	PBPK_MADAM	1	1	dimensionless

	Quantity Name	Type	Scope	Value	Initial Value	Units
626	uptake_inhib_S5	parameter	PBPK_MADAM	1	1	dimensionless
627	switch_biliary_inhib_1	parameter	PBPK_MADAM	0	0	dimensionless
628	switch_met_inhib_1	parameter	PBPK_MADAM	0	0	dimensionless
629	biliary_inhib_S1	parameter	PBPK_MADAM	1	1	dimensionless
630	biliary_inhib_S2	parameter	PBPK_MADAM	1	1	dimensionless
631	biliary_inhib_S3	parameter	PBPK_MADAM	1	1	dimensionless
632	biliary_inhib_S4	parameter	PBPK_MADAM	1	1	dimensionless
633	biliary_inhib_S5	parameter	PBPK_MADAM	1	1	dimensionless
634	switch_uptake_inhib_1	parameter	PBPK_MADAM	0	0	dimensionless
635	met_inhib_S1	parameter	PBPK_MADAM	1	1	dimensionless
636	met_inhib_S2	parameter	PBPK_MADAM	1	1	dimensionless
637	met_inhib_S3	parameter	PBPK_MADAM	1	1	dimensionless
638	met_inhib_S4	parameter	PBPK_MADAM	1	1	dimensionless
639	met_inhib_S5	parameter	PBPK_MADAM	1	1	dimensionless
640	drug_biliary_Ki	parameter	PBPK_MADAM	0.226	0.226	micromole/liter
641	drug_met_Ki	parameter	PBPK_MADAM	0.226	0.226	micromole/liter
642	drug_dose_IV_start_1	parameter	PBPK_MADAM	3	3	hour
643	drug_dose_IV_start	parameter	PBPK_MADAM	3	3	hour
644	Kpuu_Liver_1	parameter	PBPK_MADAM	1	NaN	dimensionless
645	drug_k_bile_deg	parameter	PBPK_MADAM	0	0	1/hour
646	drug_k_bile_deg_1	parameter	PBPK_MADAM	0	0	1/hour
647	TSTOMACH	parameter	PBPK_MADAM	15	16.2	minute
648	TDUO	parameter	PBPK_MADAM	15.6	9.384	minute
649	TJEJ1	parameter	PBPK_MADAM	56.4	35.292	minute
650	TJEJ2	parameter	PBPK_MADAM	42	35.292	minute

	Quantity Name	Type	Scope	Value	Initial Value	Units
651	TILL1	parameter	PBPK_MADAM	34.8	31.008	minute
652	TILL2	parameter	PBPK_MADAM	25.2	31.008	minute
653	TILL3	parameter	PBPK_MADAM	17.4	31.008	minute
654	TILL4	parameter	PBPK_MADAM	261	31.008	minute
655	QMUC	parameter	PBPK_MADAM	142.8571	170	milliliter/minute
656	SOLIF_STOMACH	parameter	PBPK_MADAM	1476.373	64	milligram/liter
657	SOLIF_DUO	parameter	PBPK_MADAM	1476.373	64	milligram/liter
658	SOLIF_JEJ1	parameter	PBPK_MADAM	1476.373	64	milligram/liter
659	SOLIF_JEJ2	parameter	PBPK_MADAM	1476.373	64	milligram/liter
660	SOLIF_ILL1	parameter	PBPK_MADAM	1476.373	64	milligram/liter
661	SOLIF_ILL2	parameter	PBPK_MADAM	1476.373	64	milligram/liter
662	SOLIF_ILL3	parameter	PBPK_MADAM	1476.373	64	milligram/liter
663	SOLIF_ILL4	parameter	PBPK_MADAM	1476.373	64	milligram/liter
664	DIFF	parameter	PBPK_MADAM	0.010286	2.7771	centimeter^3/minute
665	NI_DUO	parameter	PBPK_MADAM	1	1	dimensionless
666	NI_JEJ1	parameter	PBPK_MADAM	1	1	dimensionless
667	NI_JEJ2	parameter	PBPK_MADAM	1	1	dimensionless
668	NI_ILL1	parameter	PBPK_MADAM	1	1	dimensionless
669	NI_ILL2	parameter	PBPK_MADAM	1	1	dimensionless
670	NI_ILL3	parameter	PBPK_MADAM	1	1	dimensionless
671	NI_ILL4	parameter	PBPK_MADAM	1	1	dimensionless
672	KD	parameter	PBPK_MADAM	0.0002	1.5385e-05	liter/milligram/minute
673	fu_mem	parameter	PBPK_MADAM	1	0.42635	dimensionless
674	CLINT_efflux_DUO	parameter	PBPK_MADAM	1	0	milliliter/minute
675	CLINT_influx_DUO	parameter	PBPK_MADAM	1	0	milliliter/minute

	Quantity Name	Type	Scope	Value	Initial Value	Units
676	CLINT_metabolic_DUO	parameter	PBPK_MADAM	1	1	milliliter/minute
677	CLINT_efflux_JEJ1	parameter	PBPK_MADAM	1	0	milliliter/minute
678	CLINT_influx_JEJ1	parameter	PBPK_MADAM	1	0	milliliter/minute
679	CLINT_metabolic_JEJ1	parameter	PBPK_MADAM	1	1	milliliter/minute
680	CLINT_efflux_JEJ2	parameter	PBPK_MADAM	1	0	milliliter/minute
681	CLINT_influx_JEJ2	parameter	PBPK_MADAM	1	0	milliliter/minute
682	CLINT_metabolic_JEJ2	parameter	PBPK_MADAM	1	1	milliliter/minute
683	CLINT_efflux_ILL1	parameter	PBPK_MADAM	1	0	milliliter/minute
684	CLINT_influx_ILL1	parameter	PBPK_MADAM	1	0	milliliter/minute
685	CLINT_metabolic_ILL1	parameter	PBPK_MADAM	1	1	milliliter/minute
686	CLINT_efflux_ILL2	parameter	PBPK_MADAM	1	0	milliliter/minute
687	CLINT_influx_ILL2	parameter	PBPK_MADAM	1	0	milliliter/minute
688	CLINT_metabolic_ILL2	parameter	PBPK_MADAM	1	1	milliliter/minute
689	CLINT_efflux_ILL3	parameter	PBPK_MADAM	1	0	milliliter/minute
690	CLINT_influx_ILL3	parameter	PBPK_MADAM	1	0	milliliter/minute
691	CLINT_metabolic_ILL3	parameter	PBPK_MADAM	1	1	milliliter/minute
692	CLINT_efflux_ILL4	parameter	PBPK_MADAM	1	0	milliliter/minute
693	CLINT_influx_ILL4	parameter	PBPK_MADAM	1	0	milliliter/minute
694	CLINT_metabolic_ILL4	parameter	PBPK_MADAM	1	1	milliliter/minute
695	BW_average	parameter	PBPK_MADAM	70	70	kilogram
696	liter_to_milliliter	parameter	PBPK_MADAM	1000	1000	milliliter/liter
697	phys_Normalized_weight_stomach	parameter	PBPK_MADAM	2.1	2.1	gram/kilogram
698	V_LUM_TOT	parameter	PBPK_MADAM	6543.215	126.95	milliliter
699	V_ONECOMP	parameter	PBPK_MADAM	934.745	18.1357	milliliter

	Quantity Name	Type	Scope	Value	Initial Value	Units
700	VGut	parameter	PBPK_MADAM	50	0.517	liter
701	V_MEM	parameter	PBPK_MADAM	7142.8571	73.857	milliliter
702	HHINT	parameter	PBPK_MADAM	1	1	dimensionless
703	HHSTOMACH	parameter	PBPK_MADAM	1	1	dimensionless
704	HHDUO	parameter	PBPK_MADAM	1	1	dimensionless
705	HHJEJ1	parameter	PBPK_MADAM	1	1	dimensionless
706	HHJEJ2	parameter	PBPK_MADAM	1	1	dimensionless
707	HHILL1	parameter	PBPK_MADAM	1	1	dimensionless
708	HHILL2	parameter	PBPK_MADAM	1	1	dimensionless
709	HHILL3	parameter	PBPK_MADAM	1	1	dimensionless
710	HHILL4	parameter	PBPK_MADAM	1	1	dimensionless
711	LOGP	parameter	PBPK_MADAM	1	1.26	dimensionless
712	MW	parameter	PBPK_MADAM	1	780940	microgram/micromole
713	CACO2AB	parameter	PBPK_MADAM	1e-06	1e-06	centimeter/second
714	CACO2BA	parameter	PBPK_MADAM	1e-06	1e-06	centimeter/second
715	HPeff_exp	parameter	PBPK_MADAM	0.0001	0.0001	centimeter/second
716	SOLWATER	parameter	PBPK_MADAM	1	1	milligram/liter
717	REFPHSOL	parameter	PBPK_MADAM	7.4	6.5	dimensionless
718	PSIZE	parameter	PBPK_MADAM	0.0005	0.0065	centimeter
719	PDENSITY	parameter	PBPK_MADAM	1000000	1000000	microgram/milliliter
720	DLT	parameter	PBPK_MADAM	0.003	0.003	centimeter
721	DIFFCOEFF	parameter	PBPK_MADAM	0.0001	0.0001	centimeter^2/minute
722	LL	parameter	PBPK_MADAM	680	680	centimeter
723	LR	parameter	PBPK_MADAM	1.75	1.75	centimeter
724	phys_ESA	parameter	PBPK_MADAM	120000	120000	centimeter^2

	Quantity Name	Type	Scope	Value	Initial Value	Units
725	LOGSR	parameter	PBPK_MADAM	3.02	3.215	dimensionless
726	SOLBILE	parameter	PBPK_MADAM	368.8433	368.8433	milligram/liter
727	NATC	parameter	PBPK_MADAM	4	4	nanomole/liter
728	SOLFASSIF	parameter	PBPK_MADAM	1	64	milligram/liter
729	SOLINT	parameter	PBPK_MADAM	1476.373	64	milligram/liter
730	HPeff_est	parameter	PBPK_MADAM	0.0001	2.7e-06	centimeter/second
731	numIntestinalCompartments	parameter	PBPK_MADAM	7	7	dimensionless
732	second_per_minute	parameter	PBPK_MADAM	60	60	second/minute
733	minute_per_hour	parameter	PBPK_MADAM	60	60	minute/hour
734	milligram_per_microgram	parameter	PBPK_MADAM	0.001	0.001	milligram/microgram
735	pHStomach	parameter	PBPK_MADAM	1	1.5	dimensionless
736	pHDuo	parameter	PBPK_MADAM	1	6.4	dimensionless
737	pHJej1	parameter	PBPK_MADAM	1	6.5	dimensionless
738	pHJej2	parameter	PBPK_MADAM	1	6.6	dimensionless
739	pHIll1	parameter	PBPK_MADAM	1	6.8	dimensionless
740	pHIll2	parameter	PBPK_MADAM	1	7	dimensionless
741	pHIll3	parameter	PBPK_MADAM	1	7.7	dimensionless
742	pHIll4	parameter	PBPK_MADAM	1	7.3	dimensionless
743	fu_blood	parameter	PBPK_MADAM	1	0.66355	dimensionless
744	drug_Km_influx	parameter	PBPK_MADAM	1	1	micromole/liter
745	drug_Km_efflux	parameter	PBPK_MADAM	1	2.43	micromole/liter
746	Gut_EC_fraction	parameter	PBPK_MADAM	0.3719	0.3719	dimensionless
747	Gut_IC_fraction	parameter	PBPK_MADAM	0.6281	0.6281	dimensionless
748	influx_factor_duo	parameter	PBPK_MADAM	1	1	dimensionless

	Quantity Name	Type	Scope	Value	Initial Value	Units
749	influx_factor_jej1	parameter	PBPK_MADAM	1	1	dimensionless
750	influx_factor_jej2	parameter	PBPK_MADAM	1	1	dimensionless
751	influx_factor_ill1	parameter	PBPK_MADAM	1	1	dimensionless
752	influx_factor_ill2	parameter	PBPK_MADAM	1	1	dimensionless
753	influx_factor_ill3	parameter	PBPK_MADAM	1	1	dimensionless
754	influx_factor_ill4	parameter	PBPK_MADAM	1	1	dimensionless
755	efflux_factor_duo	parameter	PBPK_MADAM	1	0.23	dimensionless
756	efflux_factor_jej1	parameter	PBPK_MADAM	1	1	dimensionless
757	efflux_factor_jej2	parameter	PBPK_MADAM	1	1.44	dimensionless
758	efflux_factor_ill1	parameter	PBPK_MADAM	1	2.14	dimensionless
759	efflux_factor_ill2	parameter	PBPK_MADAM	1	2.14	dimensionless
760	efflux_factor_ill3	parameter	PBPK_MADAM	1	2.14	dimensionless
761	efflux_factor_ill4	parameter	PBPK_MADAM	1	2.14	dimensionless
762	switchVmax_influx	parameter	PBPK_MADAM	0	0	micromole/minute/centimeter^2
763	switchVmax_efflux	parameter	PBPK_MADAM	0	2.207e-05	micromole/minute/centimeter^2
764	Qmuc_DUO	parameter	PBPK_MADAM	1	26.9235	milliliter/minute
765	Qmuc_JEJ1	parameter	PBPK_MADAM	1	74.0397	milliliter/minute
766	Qmuc_JEJ2	parameter	PBPK_MADAM	1	74.0397	milliliter/minute
767	Qmuc_ILL1	parameter	PBPK_MADAM	1	32.7365	milliliter/minute
768	Qmuc_ILL2	parameter	PBPK_MADAM	1	32.7365	milliliter/minute
769	Qmuc_ILL3	parameter	PBPK_MADAM	1	32.7365	milliliter/minute
770	Qmuc_ILL4	parameter	PBPK_MADAM	1	32.7365	milliliter/minute
771	volumeRatio_DUO	parameter	PBPK_MADAM	0.1429	0.11534	dimensionless
772	volumeRatio_JEJ1	parameter	PBPK_MADAM	0.1429	0.22722	dimensionless
773	volumeRatio_JEJ2	parameter	PBPK_MADAM	0.1429	0.15917	dimensionless

	Quantity Name	Type	Scope	Value	Initial Value	Units
774	volumeRatio_ILL1	parameter	PBPK_MADAM	0.1429	0.12687	dimensionless
775	volumeRatio_ILL2	parameter	PBPK_MADAM	0.1429	0.12687	dimensionless
776	volumeRatio_ILL3	parameter	PBPK_MADAM	0.1429	0.12457	dimensionless
777	volumeRatio_ILL4	parameter	PBPK_MADAM	0.1429	0.11995	dimensionless
778	flowRatio_DUO	parameter	PBPK_MADAM	0.1429	0.088	dimensionless
779	flowRatio_JEJ1	parameter	PBPK_MADAM	0.1429	0.242	dimensionless
780	flowRatio_JEJ2	parameter	PBPK_MADAM	0.1429	0.242	dimensionless
781	flowRatio_ILL1	parameter	PBPK_MADAM	0.1429	0.107	dimensionless
782	flowRatio_ILL2	parameter	PBPK_MADAM	0.1429	0.107	dimensionless
783	flowRatio_ILL3	parameter	PBPK_MADAM	0.1429	0.107	dimensionless
784	flowRatio_ILL4	parameter	PBPK_MADAM	0.1429	0.107	dimensionless
785	switch_SFinflux	parameter	PBPK_MADAM	1	1	dimensionless
786	switch_SFefflux	parameter	PBPK_MADAM	1	1	dimensionless
787	switch_SFgutmet	parameter	PBPK_MADAM	1	1	dimensionless
788	switch_SFdiffapi	parameter	PBPK_MADAM	1	1	dimensionless
789	switch_SFdiffbaso	parameter	PBPK_MADAM	1	1	dimensionless
790	zero	parameter	PBPK_MADAM	0	0	micromole/minute/centimeter^2
791	CLINT_metabolism	parameter	PBPK_MADAM	1	0	milliliter/minute
792	metabolism_factor_duo	parameter	PBPK_MADAM	1	1	dimensionless
793	metabolism_factor_jej1	parameter	PBPK_MADAM	1	1	dimensionless
794	metabolism_factor_jej2	parameter	PBPK_MADAM	1	1	dimensionless
795	metabolism_factor_ill1	parameter	PBPK_MADAM	1	1	dimensionless
796	metabolism_factor_ill2	parameter	PBPK_MADAM	1	1	dimensionless
797	metabolism_factor_ill3	parameter	PBPK_MADAM	1	1	dimensionless
798	metabolism_factor_ill4	parameter	PBPK_MADAM	1	1	dimensionless

	Quantity Name	Type	Scope	Value	Initial Value	Units
799	k_liver_metabolites	parameter	PBPK_MADAM	1	1.6185	liter/hour
800	k_liver_bile	parameter	PBPK_MADAM	1	0	liter/hour
801	Kp_liver	parameter	PBPK_MADAM	1	1.1308	dimensionless
802	drug_Kp_liver_raw	parameter	PBPK_MADAM	1.1	1.1308	dimensionless
803	phys_Normalized_Q_villi	parameter	PBPK_MADAM	4.8	4.8	milliliter/minute/kilogram
804	phys_Normalized_weight_enterocyte	parameter	PBPK_MADAM	7.3857	7.3857	gram/kilogram
805	LumenTotal	parameter	PBPK_MADAM	126.95	126.95	milliliter
806	lumenvolumeRatio_DUO	parameter	PBPK_MADAM	1	0.27058	dimensionless
807	lumenvolumeRatio_JEJ1	parameter	PBPK_MADAM	1	0.16621	dimensionless
808	lumenvolumeRatio_JEJ2	parameter	PBPK_MADAM	1	0.16621	dimensionless
809	lumenvolumeRatio_ILL1	parameter	PBPK_MADAM	1	0.099252	dimensionless
810	lumenvolumeRatio_ILL2	parameter	PBPK_MADAM	1	0.099252	dimensionless
811	lumenvolumeRatio_ILL3	parameter	PBPK_MADAM	1	0.099252	dimensionless
812	lumenvolumeRatio_ILL4	parameter	PBPK_MADAM	1	0.099252	dimensionless
813	Q_villi	parameter	PBPK_MADAM	1	336	milliliter/minute
814	StomachLumenTotal	parameter	PBPK_MADAM	50	50	milliliter
815	phys_ESA_baso	parameter	PBPK_MADAM	1	6703	centimeter^2
816	DIFF_duo	parameter	PBPK_MADAM	1	2.2356	centimeter^3/minute
817	DIFF_jej1	parameter	PBPK_MADAM	1	4.4129	centimeter^3/minute
818	DIFF_jej2	parameter	PBPK_MADAM	1	3.091	centimeter^3/minute
819	DIFF_ill1	parameter	PBPK_MADAM	1	2.4689	centimeter^3/minute
820	DIFF_ill2	parameter	PBPK_MADAM	1	2.4689	centimeter^3/minute
821	DIFF_ill3	parameter	PBPK_MADAM	1	2.43	centimeter^3/minute
822	DIFF_ill4	parameter	PBPK_MADAM	1	2.3328	centimeter^3/minute

	Quantity Name	Type	Scope	Value	Initial Value	Units
823	surfaceRatio_DUO	parameter	PBPK_MADAM	1	0.115	dimensionless
824	surfaceRatio_JEJ1	parameter	PBPK_MADAM	1	0.227	dimensionless
825	surfaceRatio_JEJ2	parameter	PBPK_MADAM	1	0.159	dimensionless
826	surfaceRatio_ILL1	parameter	PBPK_MADAM	1	0.127	dimensionless
827	surfaceRatio_ILL2	parameter	PBPK_MADAM	1	0.127	dimensionless
828	surfaceRatio_ILL3	parameter	PBPK_MADAM	1	0.125	dimensionless
829	surfaceRatio_ILL4	parameter	PBPK_MADAM	1	0.12	dimensionless
830	DIFF_BASO_duo	parameter	PBPK_MADAM	1	0.057118	milliliter/minute
831	DIFF_BASO_jej1	parameter	PBPK_MADAM	1	0.22	milliliter/minute
832	DIFF_BASO_jej2	parameter	PBPK_MADAM	1	0.22	milliliter/minute
833	DIFF_BASO_ill1	parameter	PBPK_MADAM	1	0.14725	milliliter/minute
834	DIFF_BASO_ill2	parameter	PBPK_MADAM	1	0.14725	milliliter/minute
835	DIFF_BASO_ill3	parameter	PBPK_MADAM	1	0.14725	milliliter/minute
836	DIFF_BASO_ill4	parameter	PBPK_MADAM	1	0.14725	milliliter/minute
837	basoSurfaceRatio_DUO	parameter	PBPK_MADAM	1	0.0526	dimensionless
838	basoSurfaceRatio_JEJ1	parameter	PBPK_MADAM	1	0.2026	dimensionless
839	basoSurfaceRatio_JEJ2	parameter	PBPK_MADAM	1	0.2026	dimensionless
840	basoSurfaceRatio_ILL1	parameter	PBPK_MADAM	1	0.1356	dimensionless
841	basoSurfaceRatio_ILL2	parameter	PBPK_MADAM	1	0.1356	dimensionless
842	basoSurfaceRatio_ILL3	parameter	PBPK_MADAM	1	0.1356	dimensionless
843	basoSurfaceRatio_ILL4	parameter	PBPK_MADAM	1	0.1356	dimensionless
844	drug_fQ	parameter	PBPK_MADAM	1	0.2571	dimensionless
845	k_artery_serosa	parameter	PBPK_MADAM	1	53.0431	liter/hour
846	k_serosa_liver	parameter	PBPK_MADAM	1	45.6459	liter/hour
847	Kp_serosa	parameter	PBPK_MADAM	1	1.2434	dimensionless

	Quantity Name	Type	Scope	Value	Initial Value	Units
848	drug_Kp_serosa_raw	parameter	PBPK_MADAM	1	1.2434	dimensionless
849	pKA	parameter	PBPK_MADAM	9.3	0	dimensionless
850	CL_inf_api	parameter	PBPK_MADAM	1	0	microliter/minute/centimeter^2
851	CL_eff	parameter	PBPK_MADAM	1	0	microliter/minute/centimeter^2
852	CLINT_influx_baso_DUO	parameter	PBPK_MADAM	1	0	milliliter/minute
853	CLINT_influx_baso_JEJ1	parameter	PBPK_MADAM	1	0	milliliter/minute
854	CLINT_influx_baso_JEJ2	parameter	PBPK_MADAM	1	0	milliliter/minute
855	CLINT_influx_baso_ILL1	parameter	PBPK_MADAM	1	0	milliliter/minute
856	CLINT_influx_baso_ILL2	parameter	PBPK_MADAM	1	0	milliliter/minute
857	CLINT_influx_baso_ILL3	parameter	PBPK_MADAM	1	0	milliliter/minute
858	CLINT_influx_baso_ILL4	parameter	PBPK_MADAM	1	0	milliliter/minute
859	diff_api	parameter	PBPK_MADAM	1	0.162	microliter/minute/centimeter^2
860	HPeff_est_baso	parameter	PBPK_MADAM	1	2.7e-06	centimeter/second
861	diff_baso	parameter	PBPK_MADAM	1	0.162	microliter/minute/centimeter^2
862	CL_inf_baso	parameter	PBPK_MADAM	1	0	microliter/minute/centimeter^2
863	influx_factor_duo_baso	parameter	PBPK_MADAM	1	1	dimensionless
864	influx_factor_jej1_baso	parameter	PBPK_MADAM	1	1	dimensionless
865	influx_factor_jej2_baso	parameter	PBPK_MADAM	1	1	dimensionless
866	influx_factor_ill1_baso	parameter	PBPK_MADAM	1	1	dimensionless
867	influx_factor_ill2_baso	parameter	PBPK_MADAM	1	1	dimensionless
868	influx_factor_ill3_baso	parameter	PBPK_MADAM	1	1	dimensionless
869	influx_factor_ill4_baso	parameter	PBPK_MADAM	1	1	dimensionless
870	k_Liver_EC_S5_Venous	parameter	PBPK_MADAM	1	84	liter/hour
871	switch_liverFlag	parameter	PBPK_MADAM	1	1	dimensionless
872	phys_Normalized_ESA	parameter	PBPK_MADAM	1	1714.2857	centimeter^2/kilogram

	Quantity Name	Type	Scope	Value	Initial Value	Units
873	phys_Normalized_ESA_baso	parameter	PBPK_MADAM	1	95.7571	centimeter^2/kilogram
874	drug_inputFlag	parameter	PBPK_MADAM	1	6	dimensionless
875	drug_pKABase1	parameter	PBPK_MADAM	1	1	dimensionless
876	drug_pKABase2	parameter	PBPK_MADAM	1	1	dimensionless
877	drug_pKAAcid1	parameter	PBPK_MADAM	1	1	dimensionless
878	drug_pKAAcid2	parameter	PBPK_MADAM	1	1	dimensionless
879	SOLIF_STOMACH_1	parameter	PBPK_MADAM	1476.373	1.3162	milligram/liter
880	SOLIF_DUO_1	parameter	PBPK_MADAM	1476.373	1	milligram/liter
881	SOLIF_JEJ1_1	parameter	PBPK_MADAM	1476.373	1	milligram/liter
882	SOLIF_JEJ2_1	parameter	PBPK_MADAM	1476.373	1	milligram/liter
883	SOLIF_ILL1_1	parameter	PBPK_MADAM	1476.373	1	milligram/liter
884	SOLIF_ILL2_1	parameter	PBPK_MADAM	1476.373	1	milligram/liter
885	SOLIF_ILL3_1	parameter	PBPK_MADAM	1476.373	1	milligram/liter
886	SOLIF_ILL4_1	parameter	PBPK_MADAM	1476.373	1	milligram/liter
887	NI_DUO_1	parameter	PBPK_MADAM	1	1	dimensionless
888	NI_JEJ1_1	parameter	PBPK_MADAM	1	1	dimensionless
889	NI_JEJ2_1	parameter	PBPK_MADAM	1	1	dimensionless
890	NI_ILL1_1	parameter	PBPK_MADAM	1	1	dimensionless
891	NI_ILL2_1	parameter	PBPK_MADAM	1	1	dimensionless
892	NI_ILL3_1	parameter	PBPK_MADAM	1	1	dimensionless
893	NI_ILL4_1	parameter	PBPK_MADAM	1	1	dimensionless
894	KD_1	parameter	PBPK_MADAM	0.0002	0.0002	liter/milligram/minute
895	fu_mem_1	parameter	PBPK_MADAM	1	1	dimensionless
896	CLINT_efflux_DUO_1	parameter	PBPK_MADAM	1	13.8	milliliter/minute

	Quantity Name	Type	Scope	Value	Initial Value	Units
897	CLINT_influx_DUO_1	parameter	PBPK_MADAM	1	13.8	milliliter/minute
898	CLINT_efflux_JEJ1_1	parameter	PBPK_MADAM	1	27.24	milliliter/minute
899	CLINT_influx_JEJ1_1	parameter	PBPK_MADAM	1	27.24	milliliter/minute
900	CLINT_efflux_JEJ2_1	parameter	PBPK_MADAM	1	19.08	milliliter/minute
901	CLINT_influx_JEJ2_1	parameter	PBPK_MADAM	1	19.08	milliliter/minute
902	CLINT_efflux_ILL1_1	parameter	PBPK_MADAM	1	15.24	milliliter/minute
903	CLINT_influx_ILL1_1	parameter	PBPK_MADAM	1	15.24	milliliter/minute
904	CLINT_efflux_ILL2_1	parameter	PBPK_MADAM	1	15.24	milliliter/minute
905	CLINT_influx_ILL2_1	parameter	PBPK_MADAM	1	15.24	milliliter/minute
906	CLINT_efflux_ILL3_1	parameter	PBPK_MADAM	1	15	milliliter/minute
907	CLINT_influx_ILL3_1	parameter	PBPK_MADAM	1	15	milliliter/minute
908	CLINT_efflux_ILL4_1	parameter	PBPK_MADAM	1	14.4	milliliter/minute
909	CLINT_influx_ILL4_1	parameter	PBPK_MADAM	1	14.4	milliliter/minute
910	fu_blood_1	parameter	PBPK_MADAM	1	1	dimensionless
911	drug_Km_influx_1	parameter	PBPK_MADAM	1	1	micromole/liter
912	drug_Km_efflux_1	parameter	PBPK_MADAM	1	1	micromole/liter
913	switchVmax_influx_1	parameter	PBPK_MADAM	0	0	micromole/minute/centimeter^2
914	switchVmax_efflux_1	parameter	PBPK_MADAM	0	0	micromole/minute/centimeter^2
915	switch_SFinflux_1	parameter	PBPK_MADAM	1	1	dimensionless
916	switch_SFefflux_1	parameter	PBPK_MADAM	1	1	dimensionless
917	switch_SFgutmet_1	parameter	PBPK_MADAM	1	1	dimensionless
918	switch_SFdiffapi_1	parameter	PBPK_MADAM	1	1	dimensionless
919	switch_SFdiffbaso_1	parameter	PBPK_MADAM	1	1	dimensionless
920	zero_1	parameter	PBPK_MADAM	0	0	micromole/minute/centimeter^2
921	CLINT_metabolism_1	parameter	PBPK_MADAM	1	1	milliliter/minute

	Quantity Name	Type	Scope	Value	Initial Value	Units
922	metabolism_factor_duo_1	parameter	PBPK_MADAM	1	1	dimensionless
923	metabolism_factor_jej1_1	parameter	PBPK_MADAM	1	1	dimensionless
924	metabolism_factor_jej2_1	parameter	PBPK_MADAM	1	1	dimensionless
925	metabolism_factor_ill1_1	parameter	PBPK_MADAM	1	1	dimensionless
926	metabolism_factor_ill2_1	parameter	PBPK_MADAM	1	1	dimensionless
927	metabolism_factor_ill3_1	parameter	PBPK_MADAM	1	1	dimensionless
928	metabolism_factor_ill4_1	parameter	PBPK_MADAM	1	1	dimensionless
929	DIFF_duo_1	parameter	PBPK_MADAM	1	13.8	centimeter^3/minute
930	DIFF_jej1_1	parameter	PBPK_MADAM	1	27.24	centimeter^3/minute
931	DIFF_jej2_1	parameter	PBPK_MADAM	1	19.08	centimeter^3/minute
932	DIFF_ill1_1	parameter	PBPK_MADAM	1	15.24	centimeter^3/minute
933	DIFF_ill2_1	parameter	PBPK_MADAM	1	15.24	centimeter^3/minute
934	DIFF_ill3_1	parameter	PBPK_MADAM	1	15	centimeter^3/minute
935	DIFF_ill4_1	parameter	PBPK_MADAM	1	14.4	centimeter^3/minute
936	DIFF_BASO_duo_1	parameter	PBPK_MADAM	1	0.35258	milliliter/minute
937	DIFF_BASO_jej1_1	parameter	PBPK_MADAM	1	1.358	milliliter/minute
938	DIFF_BASO_jej2_1	parameter	PBPK_MADAM	1	1.358	milliliter/minute
939	DIFF_BASO_ill1_1	parameter	PBPK_MADAM	1	0.90893	milliliter/minute
940	DIFF_BASO_ill2_1	parameter	PBPK_MADAM	1	0.90893	milliliter/minute
941	DIFF_BASO_ill3_1	parameter	PBPK_MADAM	1	0.90893	milliliter/minute
942	DIFF_BASO_ill4_1	parameter	PBPK_MADAM	1	0.90893	milliliter/minute
943	CLINT_influx_baso_DUO_1	parameter	PBPK_MADAM	1	0.35258	milliliter/minute
944	CLINT_influx_baso_JEJ1_1	parameter	PBPK_MADAM	1	1.358	milliliter/minute

	Quantity Name	Type	Scope	Value	Initial Value	Units
945	CLINT_influx_baso_JEJ2_1	parameter	PBPK_MADAM	1	1.358	milliliter/minute
946	CLINT_influx_baso_ILL1_1	parameter	PBPK_MADAM	1	0.90893	milliliter/minute
947	CLINT_influx_baso_ILL2_1	parameter	PBPK_MADAM	1	0.90893	milliliter/minute
948	CLINT_influx_baso_ILL3_1	parameter	PBPK_MADAM	1	0.90893	milliliter/minute
949	CLINT_influx_baso_ILL4_1	parameter	PBPK_MADAM	1	0	milliliter/minute
950	Qmuc_DUO_1	parameter	PBPK_MADAM	1	26.9235	milliliter/minute
951	Qmuc_JEJ1_1	parameter	PBPK_MADAM	1	74.0397	milliliter/minute
952	Qmuc_JEJ2_1	parameter	PBPK_MADAM	1	74.0397	milliliter/minute
953	Qmuc_ILL1_1	parameter	PBPK_MADAM	1	32.7365	milliliter/minute
954	Qmuc_ILL2_1	parameter	PBPK_MADAM	1	32.7365	milliliter/minute
955	Qmuc_ILL3_1	parameter	PBPK_MADAM	1	32.7365	milliliter/minute
956	Qmuc_ILL4_1	parameter	PBPK_MADAM	1	32.7365	milliliter/minute
957	switch_liverFlag_1	parameter	PBPK_MADAM	1	1	dimensionless
958	k_Liver_Venous_1	parameter	PBPK_MADAM	1	105.3302	liter/hour
959	k_artery_serosa_1	parameter	PBPK_MADAM	1	53.0431	liter/hour
960	k_serosa_liver_1	parameter	PBPK_MADAM	1	66.5123	liter/hour
961	k_liver_metabolites_1	parameter	PBPK_MADAM	1	1.0507	liter/hour
962	k_liver_bile_1	parameter	PBPK_MADAM	1	0.098292	liter/hour
963	Kp_serosa_1	parameter	PBPK_MADAM	1	0.51837	dimensionless
964	drug_Kp_serosa_raw_1	parameter	PBPK_MADAM	1	1	dimensionless
965	HPeff_est_baso_1	parameter	PBPK_MADAM	1	1.6667e-05	centimeter/second
966	diff_baso_1	parameter	PBPK_MADAM	1	1	microliter/minute/centimeter^2
967	CL_inf_baso_1	parameter	PBPK_MADAM	1	1	microliter/minute/centimeter^2

	Quantity Name	Type	Scope	Value	Initial Value	Units
968	CL_inf_api_1	parameter	PBPK_MADAM	1	1	microliter/minute/centimeter^2
969	HPeff_est_1	parameter	PBPK_MADAM	0.0001	1.6667e-05	centimeter/second
970	diff_api_1	parameter	PBPK_MADAM	1	1	microliter/minute/centimeter^2
971	CL_eff_1	parameter	PBPK_MADAM	1	1	microliter/minute/centimeter^2
972	HHSTOMACH_1	parameter	PBPK_MADAM	1	1.3162	dimensionless
973	SOLINT_1	parameter	PBPK_MADAM	1476.373	1	milligram/liter
974	HHDUO_1	parameter	PBPK_MADAM	1	1	dimensionless
975	HHJEJ1_1	parameter	PBPK_MADAM	1	1	dimensionless
976	HHJEJ2_1	parameter	PBPK_MADAM	1	1	dimensionless
977	HHILL1_1	parameter	PBPK_MADAM	1	1	dimensionless
978	HHILL2_1	parameter	PBPK_MADAM	1	1	dimensionless
979	HHILL3_1	parameter	PBPK_MADAM	1	1	dimensionless
980	HHILL4_1	parameter	PBPK_MADAM	1	1	dimensionless
981	HHINT_1	parameter	PBPK_MADAM	1	1	dimensionless
982	SOLFASSIF_1	parameter	PBPK_MADAM	1	1	milligram/liter
983	REFPHSOL_1	parameter	PBPK_MADAM	7.4	7.4	dimensionless
984	drug_inputFlag_1	parameter	PBPK_MADAM	1	1	dimensionless
985	drug_pKABase1_1	parameter	PBPK_MADAM	1	1	dimensionless
986	drug_pKABase2_1	parameter	PBPK_MADAM	1	1	dimensionless
987	drug_pKAAcid1_1	parameter	PBPK_MADAM	1	1	dimensionless
988	drug_pKAAcid2_1	parameter	PBPK_MADAM	1	1	dimensionless
989	PSIZE_1	parameter	PBPK_MADAM	0.0005	0.0005	centimeter
990	PDENSITY_1	parameter	PBPK_MADAM	1000000	1000000	microgram/milliliter
991	DLT_1	parameter	PBPK_MADAM	0.003	0.003	centimeter
992	DIFFCOEFF_1	parameter	PBPK_MADAM	0.0001	0.0001	centimeter^2/minute

	Quantity Name	Type	Scope	Value	Initial Value	Units
993	efflux_inhib_duo	parameter	PBPK_MADAM	1	1	dimensionless
994	switch_efflux_inhib_1	parameter	PBPK_MADAM	1	1	dimensionless
995	drug_efflux_Ki	parameter	PBPK_MADAM	1	1	micromole/liter
996	efflux_inhib_jej1	parameter	PBPK_MADAM	1	1	dimensionless
997	efflux_inhib_jej2	parameter	PBPK_MADAM	1	1	dimensionless
998	efflux_inhib_ill1	parameter	PBPK_MADAM	1	1	dimensionless
999	efflux_inhib_ill2	parameter	PBPK_MADAM	1	1	dimensionless
1000	efflux_inhib_ill3	parameter	PBPK_MADAM	1	1	dimensionless
1001	efflux_inhib_ill4	parameter	PBPK_MADAM	1	1	dimensionless
1002	Kp_liver_1	parameter	PBPK_MADAM	1	0.51837	dimensionless
1003	drug_Kp_liver_raw_1	parameter	PBPK_MADAM	1	1	dimensionless
1004	LOGP_1	parameter	PBPK_MADAM	1	1	dimensionless
1005	LOGSR_1	parameter	PBPK_MADAM	1	3.02	dimensionless
1006	MW_1	parameter	PBPK_MADAM	1	1	microgram/micromole
1007	pKA_1	parameter	PBPK_MADAM	1	1	dimensionless
1008	CLINT_efflux_baso_DUO	parameter	PBPK_MADAM	1	0	milliliter/minute
1009	CLINT_efflux_baso_JEJ1	parameter	PBPK_MADAM	1	0	milliliter/minute
1010	CLINT_efflux_baso_JEJ2	parameter	PBPK_MADAM	1	0	milliliter/minute
1011	CLINT_efflux_baso_ILL1	parameter	PBPK_MADAM	1	0	milliliter/minute
1012	CLINT_efflux_baso_ILL2	parameter	PBPK_MADAM	1	0	milliliter/minute
1013	CLINT_efflux_baso_ILL3	parameter	PBPK_MADAM	1	0	milliliter/minute
1014	CLINT_efflux_baso_ILL4	parameter	PBPK_MADAM	1	0	milliliter/minute
1015	CL_eff_baso	parameter	PBPK_MADAM	0	0	microliter/minute/centimeter^2
1016	switch_SFefflux_baso	parameter	PBPK_MADAM	1	1	dimensionless
1017	baso_efflux_factor_duo	parameter	PBPK_MADAM	1	1	dimensionless

	Quantity Name	Type	Scope	Value	Initial Value	Units
1018	baso_efflux_factor_jej1	parameter	PBPK_MADAM	1	1	dimensionless
1019	baso_efflux_factor_jej2	parameter	PBPK_MADAM	1	1	dimensionless
1020	baso_efflux_factor_ill1	parameter	PBPK_MADAM	1	1	dimensionless
1021	baso_efflux_factor_ill2	parameter	PBPK_MADAM	1	1	dimensionless
1022	baso_efflux_factor_ill3	parameter	PBPK_MADAM	1	1	dimensionless
1023	baso_efflux_factor_ill4	parameter	PBPK_MADAM	1	1	dimensionless
1024	switchVmax_efflux_baso	parameter	PBPK_MADAM	0.00016	3.049e-09	micromole/minute/centimeter^2
1025	drug_Km_efflux_baso	parameter	PBPK_MADAM	89	658	micromole/liter
1026	switchVmax_efflux_baso_1	parameter	PBPK_MADAM	0	0	micromole/minute/centimeter^2
1027	CLINT_efflux_baso_DUO_1	parameter	PBPK_MADAM	1	0	milliliter/minute
1028	switch_SFefflux_baso_1	parameter	PBPK_MADAM	1	1	dimensionless
1029	drug_Km_efflux_baso_1	parameter	PBPK_MADAM	1	1	micromole/liter
1030	CLINT_efflux_baso_JEJ1_1	parameter	PBPK_MADAM	1	0	milliliter/minute
1031	CLINT_efflux_baso_JEJ2_1	parameter	PBPK_MADAM	1	0	milliliter/minute
1032	CLINT_efflux_baso_ILL1_1	parameter	PBPK_MADAM	1	0	milliliter/minute
1033	CLINT_efflux_baso_ILL2_1	parameter	PBPK_MADAM	1	0	milliliter/minute
1034	CLINT_efflux_baso_ILL3_1	parameter	PBPK_MADAM	1	0	milliliter/minute
1035	CLINT_efflux_baso_ILL4_1	parameter	PBPK_MADAM	1	0	milliliter/minute
1036	CL_eff_baso_1	parameter	PBPK_MADAM	0	0	microliter/minute/centimeter^2

Initial Assignments

	Initial Assignments	Initial Value
1	k_Liver_IC_S5_Bile = drug_PSbileg*switch_SFbile*phys_HPGL*(phys_BW*phys_Normalized_weight_liver_tissue*Specific_volume)/Specific_volume/5*drug_fuLiver	0
2	k_Liver_IC_S4_Bile = drug_PSbileg*switch_SFbile*phys_HPGL*(phys_BW*phys_Normalized_weight_liver_tissue*Specific_volume)/Specific_volume/5*drug_fuLiver	0
3	k_Liver_IC_S3_Bile = drug_PSbileg*switch_SFbile*phys_HPGL*(phys_BW*phys_Normalized_weight_liver_tissue*Specific_volume)/Specific_volume/5*drug_fuLiver	0
4	k_Liver_IC_S2_Bile = drug_PSbileg*switch_SFbile*phys_HPGL*(phys_BW*phys_Normalized_weight_liver_tissue*Specific_volume)/Specific_volume/5*drug_fuLiver	0
5	k_Liver_IC_S1_Bile = drug_PSbileg*switch_SFbile*phys_HPGL*(phys_BW*phys_Normalized_weight_liver_tissue*Specific_volume)/Specific_volume/5*drug_fuLiver	0
6	k_Liver_EC_S4_Liver_EC_S5 = Q_li	84
7	k_Liver_EC_S3_Liver_EC_S4 = Q_li	84
8	k_Liver_EC_S2_Liver_EC_S3 = Q_li	84
9	k_Liver_EC_S1_Liver_EC_S2 = Q_li	84
10	k_Liver_IC_S5_Liver_EC_S5 = drug_PSdifg*switch_SFdiff*phys_HPGL*(phys_BW*phys_Normalized_weight_liver_tissue*Specific_volume)/Specific_volume/5*drug_fuLiver	4.029
11	k_Liver_IC_S4_Liver_EC_S4 = drug_PSdifg*switch_SFdiff*phys_HPGL*(phys_BW*phys_Normalized_weight_liver_tissue*Specific_volume)/Specific_volume/5*drug_fuLiver	4.029
12	k_Liver_IC_S2_Liver_EC_S2 = drug_PSdifg*switch_SFdiff*phys_HPGL*(phys_BW*phys_Normalized_weight_liver_tissue*Specific_volume)/Specific_volume/5*drug_fuLiver	4.029
13	k_Liver_IC_S1_Liver_EC_S1 = drug_PSdifg*switch_SFdiff*phys_HPGL*(phys_BW*phys_Normalized_weight_liver_tissue*Specific_volume)/Specific_volume/5*drug_fuLiver	4.029
14	k_rest_venous = Qrest/Kp_rest*drug_BRP	5.7489
15	k_artery_spleen = Q_artery_spleen	8.4
16	k_artery_gut = Q_artery_gut	71.4
17	k_gut_liver = Q_gut_liver/Kp_gut*drug_BRP	61.4428
18	k_spleen_liver = Q_spleen_liver/Kp_spleen*drug_BRP	9.425
19	k_artery_rest = Qrest	4.2
20	k_kidney_venous = Qkidney/Kp_kidney*drug_BRP	72.7679
21	k_muscle_venous = Qmuscle/Kp_muscle*drug_BRP	3.9421
22	k_brain_venous = Qbrain/Kp_brain*drug_BRP	30.7322
23	k_skin_venous = Qskin/Kp_skin*drug_BRP	18.9708
24	k_bone_venous = Qbone/Kp_bone*drug_BRP	13.9966

	Initial Assignments	Initial Value
25	k_heart_venous = Qheart/Kp_heart*drug_BRP	0.22189
26	k_adipos_venous = Qadipose/Kp_adipose*drug_BRP	22.9954
27	k_artery_muscle = Qmuscle	58.8
28	k_artery_adipos = Qadipose	16.8
29	k_artery_bone = Qbone	16.8
30	k_artery_skin = Qskin	16.8
31	k_artery_kidney = Qkidney	63
32	k_artery_brain = Qbrain	42
33	k_venous_lung = Qlung	336
34	k_lung_artery = Qlung/Kp_lung*drug_BRP	518.5185
35	k_artery_heart = Qheart	12.6
36	k_Liver_Venous = Q_li/Kp_liver*drug_BRP	79.4828
37	k_Liver_IC_S3_Liver__EC_S3 = drug_PSdifg*switch_SFdiff*phys_HPGL*(phys_BW*phys_Normalized_weight_liver_tissue*Specific_volume)/Specific_volume/5*drug_fuLiver	4.029
38	k_artery_liver = Q_artery_liver	4.2
39	Venous = phys_BW*phys_Normalized_weight_venous*Specific_volume	3.598
40	Lung = phys_BW*phys_Normalized_weight_lung*Specific_volume	0.56
41	Kidney = phys_BW*phys_Normalized_weight_kidney*Specific_volume	0.35
42	Brain = phys_BW*phys_Normalized_weight_brain*Specific_volume	1.47
43	Muscle = phys_BW*phys_Normalized_weight_muscle*Specific_volume	29.12
44	Adipose = phys_BW*phys_Normalized_weight_adipose*Specific_volume	13.79
45	Heart = phys_BW*phys_Normalized_weight_heart*Specific_volume	0.35
46	Skin = phys_BW*phys_Normalized_weight_skin*Specific_volume	2.87
47	Bone = phys_BW*phys_Normalized_weight_bone*Specific_volume	11.06
48	Rest = phys_BW*phys_Normalized_weight_remainder*Specific_volume	7
49	Artery = phys_BW*phys_Normalized_weight_artery*Specific_volume	1.799
50	Spleen = phys_BW*phys_Normalized_weight_spleen*Specific_volu	0.21

	Initial Assignments	Initial Value
	me	
51	Gut = phys_BW*phys_Normalized_weight_gut*Specific_volume	1.26
52	Liver_EC_S1 = phys_BW*phys_Normalized_weight_liver_blood*Specific_volume/5	0.0686
53	Liver_EC_S2 = phys_BW*phys_Normalized_weight_liver_blood*Specific_volume/5	0.0686
54	Liver_EC_S3 = phys_BW*phys_Normalized_weight_liver_blood*Specific_volume/5	0.0686
55	Liver_EC_S4 = phys_BW*phys_Normalized_weight_liver_blood*Specific_volume/5	0.0686
56	Liver_EC_S5 = phys_BW*phys_Normalized_weight_liver_blood*Specific_volume/5	0.0686
57	Liver_IC_S5 = phys_BW*phys_Normalized_weight_liver_tissue*Specific_volume/5	0.252
58	Liver_IC_S4 = phys_BW*phys_Normalized_weight_liver_tissue*Specific_volume/5	0.252
59	Liver_IC_S3 = phys_BW*phys_Normalized_weight_liver_tissue*Specific_volume/5	0.252
60	Liver_IC_S2 = phys_BW*phys_Normalized_weight_liver_tissue*Specific_volume/5	0.252
61	Liver_IC_S1 = phys_BW*phys_Normalized_weight_liver_tissue*Specific_volume/5	0.252
62	Q_artery_gut = phys_BW*phys_Normalized_Q_gut	71.4
63	Q_artery_liver = Q_li-Q_artery_gut-Q_artery_spleen	4.2
64	Q_artery_spleen = phys_BW*phys_Normalized_Q_spleen	8.4
65	Q_gut_liver = phys_BW*phys_Normalized_Q_gut	71.4
66	Q_spleen_liver = phys_BW*phys_Normalized_Q_spleen	8.4
67	Qadipose = phys_BW*phys_Normalized_Q_adipose	16.8
68	Qbone = phys_BW*phys_Normalized_Q_bone	16.8
69	Qbrain = phys_BW*phys_Normalized_Q_brain	42
70	Qheart = phys_BW*phys_Normalized_Q_heart	12.6
71	Qkidney = phys_BW*phys_Normalized_Q_kidney	63
72	Q_li = phys_BW*phys_Normalized_Q_liver	84
73	Qlung = phys_BW*phys_Normalized_Q_lung	336
74	Qmuscle = phys_BW*phys_Normalized_Q_muscle	58.8
75	Qrest = phys_BW*phys_Normalized_Q_remainder	4.2
76	Qskin = phys_BW*phys_Normalized_Q_skin	16.8
77	drug_fB = drug_fuplasma/drug_BRP	0.66355

	Initial Assignments	Initial Value
78	k_venous_urine_CLR = drug_CLrenal*switch_SFrenal	9.66
79	k_artery_testes = Qtestes	0
80	k_testes_venous = Qtestes/Kp_testes*drug_BRP	0
81	Qtestes = phys_BW*phys_Normalized_Q_testes	0
82	k_Liver_IC_S2_Liver_EC_S2_efflux = switch_SFeff*drug_CLeffluxHep*phys_HPGL*(phys_BW*phys_Normalized_weight_liver_tissue*Specific_volume)/Specific_volume/5*drug_fuLiver	0
83	k_Liver_IC_S3_Liver_EC_S3_efflux = switch_SFeff*drug_CLeffluxHep*phys_HPGL*(phys_BW*phys_Normalized_weight_liver_tissue*Specific_volume)/Specific_volume/5*drug_fuLiver	0
84	k_Liver_IC_S4_Liver_EC_S4_efflux = switch_SFeff*drug_CLeffluxHep*phys_HPGL*(phys_BW*phys_Normalized_weight_liver_tissue*Specific_volume)/Specific_volume/5*drug_fuLiver	0
85	k_Liver_IC_S5_Liver_EC_S5_efflux = switch_SFeff*drug_CLeffluxHep*phys_HPGL*(phys_BW*phys_Normalized_weight_liver_tissue*Specific_volume)/Specific_volume/5*drug_fuLiver	0
86	k_Liver_IC_S1_Liver_EC_S1_efflux = switch_SFeff*drug_CLeffluxHep*phys_HPGL*(phys_BW*phys_Normalized_weight_liver_tissue*Specific_volume)/Specific_volume/5*drug_fuLiver	0
87	Testes = phys_BW*phys_Normalized_weight_testes*Specific_volume	0.07
88	Kp_adipose = drug_Kp_adipose_raw*switch_SFKp	0.78172
89	Kp_bone = drug_Kp_bone_raw*switch_SFKp	1.2843
90	Kp_brain = drug_Kp_brain_raw*switch_SFKp	1.4623
91	Kp_gut = drug_Kp_gut_raw*switch_SFKp	1.2434
92	Kp_heart = drug_Kp_heart_raw*switch_SFKp	60.76
93	Kp_kidney = drug_Kp_kidney_raw*switch_SFKp	0.92637
94	Kp_lung = drug_Kp_lung_raw*switch_SFKp	0.69336
95	Kp_muscle = drug_Kp_muscle_raw*switch_SFKp	15.96
96	Kp_skin = drug_Kp_skin_raw*switch_SFKp	0.94756
97	Kp_spleen = drug_Kp_spleen_raw*switch_SFKp	0.95363
98	Kp_testes = drug_Kp_testes_raw*switch_SFKp	1
99	k_venous_urine_GFR = drug_GFR*drug_fuplasma*switch_SFrenal	0
100	drug_GFR = drug_FR*Qkidney	0
101	Kp_rest = switch_slow_dist_Kp*drug_Kp_rest_raw+(1-switch_slow_dist_Kp)*drug_Kp_rest_raw*switch_SFKp	0.78172
102	k_Liver_EC_S4_Liver_EC_S5_1 = Q_li_1	84
103	k_Liver_EC_S3_Liver_EC_S4_1 = Q_li_1	84
104	k_Liver_EC_S2_Liver_EC_S3_1 = Q_li_1	84
105	k_Liver_EC_S1_Liver_EC_S2_1 = Q_li_1	84
106	k_Liver_IC_S5_Liver_EC_S5_1 = drug_PSdifg_1*switch_SFdiff_1*phys_HPGL*	0.26572

	Initial Assignments	Initial Value
	(phys_BW*phys_Normalized_weight_liver_tissue*Specific_volume)/Specific_volume/5*drug_fuLiver_1	
107	k_Liver_IC_S4_Liver_EC_S4_1 = drug_PSdifg_1*switch_SFdiff_1*phys_HPGL*(phys_BW*phys_Normalized_weight_liver_tissue*Specific_volume)/Specific_volume/5*drug_fuLiver_1	0.26572
108	k_Liver_IC_S2_Liver_EC_S2_1 = drug_PSdifg_1*switch_SFdiff_1*phys_HPGL*(phys_BW*phys_Normalized_weight_liver_tissue*Specific_volume)/Specific_volume/5*drug_fuLiver_1	0.26572
109	k_Liver_IC_S1_Liver_EC_S1_1 = drug_PSdifg_1*switch_SFdiff_1*phys_HPGL*(phys_BW*phys_Normalized_weight_liver_tissue*Specific_volume)/Specific_volume/5*drug_fuLiver_1	0.26572
110	k_rest_venous_1 = Qrest_1/Kp_rest_1*drug_BRP_1	420
111	k_artery_spleen_1 = Q_artery_spleen_1	8.4
112	k_artery_gut_1 = Q_artery_gut_1	71.4
113	k_gut_liver_1 = Q_gut_liver_1/Kp_gut_1*drug_BRP_1	190.4907
114	k_spleen_liver_1 = Q_spleen_liver_1/Kp_spleen_1*drug_BRP_1	30.0943
115	k_artery_rest_1 = Qrest_1	4.2
116	k_kidney_venous_1 = Qkidney_1/Kp_kidney_1*drug_BRP_1	303.837
117	k_muscle_venous_1 = Qmuscle_1/Kp_muscle_1*drug_BRP_1	1474.6224
118	k_brain_venous_1 = Qbrain_1/Kp_brain_1*drug_BRP_1	164.5784
119	k_skin_venous_1 = Qskin_1/Kp_skin_1*drug_BRP_1	56.9352
120	k_bone_venous_1 = Qbone_1/Kp_bone_1*drug_BRP_1	35.1101
121	k_heart_venous_1 = Qheart_1/Kp_heart_1*drug_BRP_1	78.9976
122	k_adipos_venous_1 = Qadipose_1/Kp_adipose_1*drug_BRP_1	46.8134
123	k_artery_muscle_1 = Qmuscle_1	58.8
124	k_artery_adipos_1 = Qadipose_1	16.8
125	k_artery_bone_1 = Qbone_1	16.8
126	k_artery_skin_1 = Qskin_1	16.8
127	k_artery_kidney_1 = Qkidney_1	63
128	k_artery_brain_1 = Qbrain_1	42
129	k_venous_lung_1 = Qlung_1	336
130	k_lung_artery_1 = Qlung_1/Kp_lung_1*drug_BRP_1	1053.3017
131	k_artery_heart_1 = Qheart_1	12.6
132	k_Liver_EC_S5_Venous_1 = Q_li_1	84
133	k_Liver_IC_S3_Liver__EC_S3_1 = drug_PSdifg_1*switch_SFdiff_1*phys_HPGL*(phys_BW*phys_Normalized_weight_liver_tissue*Specific_volume)/Specific_volume/5*drug_fuLiver_1	0.26572
134	k_artery_liver_1 = Q_artery_liver_1	4.2
135	Venous_1 = phys_BW*phys_Normalized_weight_venous*Specific_volu	3.598

	Initial Assignments	Initial Value
	me	
136	Lung_1 = phys_BW*phys_Normalized_weight_lung*Specific_volume	0.56
137	Kidney_1 = phys_BW*phys_Normalized_weight_kidney*Specific_volume	0.35
138	Brain_1 = phys_BW*phys_Normalized_weight_brain*Specific_volume	1.47
139	Muscle_1 = phys_BW*phys_Normalized_weight_muscle*Specific_volume	29.12
140	Adipose_1 = phys_BW*phys_Normalized_weight_adipose*Specific_volume	13.79
141	Heart_1 = phys_BW*phys_Normalized_weight_heart*Specific_volume	0.35
142	Skin_1 = phys_BW*phys_Normalized_weight_skin*Specific_volume	2.87
143	Bone_1 = phys_BW*phys_Normalized_weight_bone*Specific_volume	11.06
144	Rest_1 = phys_BW*phys_Normalized_weight_remainder*Specific_volume	7
145	Artery_1 = phys_BW*phys_Normalized_weight_artery*Specific_volume	1.799
146	Spleen_1 = phys_BW*phys_Normalized_weight_spleen*Specific_volume	0.21
147	Gut_1 = phys_BW*phys_Normalized_weight_gut*Specific_volume	1.26
148	Liver_EC_S1_1 = phys_BW*phys_Normalized_weight_liver_blood*Specific_volume/5	0.0686
149	Liver_EC_S2_1 = phys_BW*phys_Normalized_weight_liver_blood*Specific_volume/5	0.0686
150	Liver_EC_S3_1 = phys_BW*phys_Normalized_weight_liver_blood*Specific_volume/5	0.0686
151	Liver_EC_S4_1 = phys_BW*phys_Normalized_weight_liver_blood*Specific_volume/5	0.0686
152	Liver_EC_S5_1 = phys_BW*phys_Normalized_weight_liver_blood*Specific_volume/5	0.0686
153	Liver_IC_S5_1 = phys_BW*phys_Normalized_weight_liver_tissue*Specific_volume/5	0.252
154	Liver_IC_S4_1 = phys_BW*phys_Normalized_weight_liver_tissue*Specific_	0.252

	Initial Assignments	Initial Value
	volume/5	
155	Liver_IC_S3_1 = phys_BW*phys_Normalized_weight_liver_tissue*Specific_volume/5	0.252
156	Liver_IC_S2_1 = phys_BW*phys_Normalized_weight_liver_tissue*Specific_volume/5	0.252
157	Liver_IC_S1_1 = phys_BW*phys_Normalized_weight_liver_tissue*Specific_volume/5	0.252
158	Q_artery_gut_1 = phys_BW*phys_Normalized_Q_gut	71.4
159	Q_artery_liver_1 = Q_li-Q_artery_gut-Q_artery_spleen	4.2
160	Q_artery_spleen_1 = phys_BW*phys_Normalized_Q_spleen	8.4
161	Q_gut_liver_1 = phys_BW*phys_Normalized_Q_gut	71.4
162	Q_spleen_liver_1 = phys_BW*phys_Normalized_Q_spleen	8.4
163	Qadipose_1 = phys_BW*phys_Normalized_Q_adipose	16.8
164	Qbone_1 = phys_BW*phys_Normalized_Q_bone	16.8
165	Qbrain_1 = phys_BW*phys_Normalized_Q_brain	42
166	Qheart_1 = phys_BW*phys_Normalized_Q_heart	12.6
167	Qkidney_1 = phys_BW*phys_Normalized_Q_kidney	63
168	Q_li_1 = phys_BW*phys_Normalized_Q_liver	84
169	Qlung_1 = phys_BW*phys_Normalized_Q_lung	336
170	Qmuscle_1 = phys_BW*phys_Normalized_Q_muscle	58.8
171	Qrest_1 = phys_BW*phys_Normalized_Q_remainder	4.2
172	Qskin_1 = phys_BW*phys_Normalized_Q_skin	16.8
173	drug_fB_1 = drug_fuplasma_1/drug_BRP_1	0.058769
174	k_venous_urine_CLR_1 = drug_CLrenal_1*switch_SFrenal_1	0
175	k_artery_testes_1 = Qtestes_1	0
176	k_testes_venous_1 = Qtestes_1/Kp_testes*drug_BRP_1	0
177	Qtestes_1 = phys_BW*phys_Normalized_Q_testes	0
178	k_Liver_IC_S2_Liver_EC_S2_efflux_1 = switch_SFeff_1*drug_CLeffluxHep_1*phys_HPGL*(phys_BW*phys_Normalized_weight_liver_tissue*Specific_volume)/Specific_volume/5*drug_fuLiver_1	0
179	k_Liver_IC_S3_Liver_EC_S3_efflux_1 = switch_SFeff_1*drug_CLeffluxHep_1*phys_HPGL*(phys_BW*phys_Normalized_weight_liver_tissue*Specific_volume)/Specific_volume/5*drug_fuLiver_1	0
180	k_Liver_IC_S4_Liver_EC_S4_efflux_1 = switch_SFeff_1*drug_CLeffluxHep_1*phys_HPGL*(phys_BW*phys_Normalized_weight_liver_tissue*Specific_volume)/Specific_volume/5*drug_fuLiver_1	0
181	k_Liver_IC_S5_Liver_EC_S5_efflux_1 = switch_SFeff_1*drug_CLeffluxHep_1*phys_HPGL*(phys_BW*phys_Normalized_weight_liver_tissue*Specific_volume)/Specific_volume/5*drug_fuLiver_1	0

	Initial Assignments	Initial Value
182	k_Liver_IC_S1_Liver_EC_S1_efflux_1 = switch_SFeff_1*drug_CLeffluxHep_1*phys_HPGL*(phys_BW*phys_Normalized_weight_liver_tissue*Specific_volume)/Specific_volume/5*drug_fuLiver_1	0
183	Testes_1 = phys_BW*phys_Normalized_weight_testes*Specific_volume	0.07
184	Kp_adipose_1 = drug_Kp_adipose_raw_1*switch_SFKp_1	0.23327
185	Kp_bone_1 = drug_Kp_bone_raw_1*switch_SFKp_1	0.31102
186	Kp_brain_1 = drug_Kp_brain_raw_1*switch_SFKp_1	0.16588
187	Kp_gut_1 = drug_Kp_gut_raw_1*switch_SFKp_1	0.24363
188	Kp_heart_1 = drug_Kp_heart_raw_1*switch_SFKp_1	0.10367
189	Kp_kidney_1 = drug_Kp_kidney_raw_1*switch_SFKp_1	0.13478
190	Kp_lung_1 = drug_Kp_lung_raw_1*switch_SFKp_1	0.20735
191	Kp_muscle_1 = drug_Kp_muscle_raw_1*switch_SFKp_1	0.025919
192	Kp_skin_1 = drug_Kp_skin_raw_1*switch_SFKp_1	0.1918
193	Kp_spleen_1 = drug_Kp_spleen_raw_1*switch_SFKp_1	0.18143
194	Kp_testes_1 = drug_Kp_testes_raw_1*switch_SFKp_1	0.51837
195	k_venous_urine_GFR_1 = drug_GFR_1*drug_fuplasma_1*switch_SFrenal_1	0
196	drug_GFR_1 = drug_FR_1*Qkidney_1	0
197	Kp_rest_1 = switch_slow_dist_Kp_1*drug_Kp_rest_raw_1+(1-switch_slow_dist_Kp_1)*drug_Kp_rest_raw_1*switch_SFKp_1	0.0065
198	STOMACH = StomachLumenTotal/BW_average*phys_BW	0.05
199	QMUC = (Q_gut_liver/numIntestinalCompartments)*liter_to_milliliter/minute_per_hour	170
200	V_LUM_TOT = LumenTotal/BW_average*phys_BW	126.95
201	V_ONECOMP = V_LUM_TOT/numIntestinalCompartments	18.1357
202	V_MEM = (VGut/numIntestinalCompartments)*liter_to_milliliter	73.857
203	LOGSR = 0.75*LOGP+2.27	3.215
204	SOLINT = SOLFASSIF/HHINT	64
205	SOLIF_STOMACH = SOLINT*HHSTOMACH	64
206	SOLIF_DUO = SOLINT*HHDUO	64
207	SOLIF_JEJ1 = SOLINT*HHJEJ1	64
208	SOLIF_JEJ2 = SOLINT*HHJEJ2	64
209	SOLIF_ILL1 = SOLINT*HHILL1	64
210	SOLIF_ILL2 = SOLINT*HHILL2	64
211	SOLIF_ILL3 = SOLINT*HHILL3	64
212	SOLIF_ILL4 = SOLINT*HHILL4	64
213	HPeff_est = diff_api	2.7e-06
214	DIFF = second_per_minute*HPeff_est*phys_ESA/numIntestinalCo	2.7771

	Initial Assignments	Initial Value
	mpartments	
215	NI_DUO = 1/HHDUO	1
216	NI_JEJ1 = 1/HHJEJ1	1
217	NI_JEJ2 = 1/HHJEJ2	1
218	NI_ILL1 = 1/HHILL1	1
219	NI_ILL2 = 1/HHILL2	1
220	NI_ILL3 = 1/HHILL3	1
221	NI_ILL4 = 1/HHILL4	1
222	KD = 3*DIFFCOEFF/(PDENSITY*PSIZE*DLT)	1.5385e-05
223	VDUO = V_LUM_TOT*lumenvolumeRatio_DUO	0.03435
224	VJEJ1 = V_LUM_TOT*lumenvolumeRatio_JEJ1	0.0211
225	VJEJ2 = V_LUM_TOT*lumenvolumeRatio_JEJ2	0.0211
226	VILL1 = V_LUM_TOT*lumenvolumeRatio_ILL1	0.0126
227	VILL2 = V_LUM_TOT*lumenvolumeRatio_ILL2	0.0126
228	VILL3 = V_LUM_TOT*lumenvolumeRatio_ILL3	0.0126
229	VILL4 = V_LUM_TOT*lumenvolumeRatio_ILL4	0.0126
230	MDUO = VGut*volumeRatio_DUO*Gut_IC_fraction	0.037454
231	MJEJ1 = VGut*volumeRatio_JEJ1*Gut_IC_fraction	0.073785
232	MJEJ2 = VGut*volumeRatio_JEJ2*Gut_IC_fraction	0.051687
233	MILL1 = VGut*volumeRatio_ILL1*Gut_IC_fraction	0.0412
234	MILL2 = VGut*volumeRatio_ILL2*Gut_IC_fraction	0.0412
235	MILL3 = VGut*volumeRatio_ILL3*Gut_IC_fraction	0.04045
236	MILL4 = VGut*volumeRatio_ILL4*Gut_IC_fraction	0.038952
237	VGut = phys_BW*phys_Normalized_weight_enterocyte*Specific_volume	0.517
238	VillousDUO = VGut*volumeRatio_DUO*Gut_EC_fraction*drug_fQ	0.0057016
239	VillousJEJ1 = VGut*volumeRatio_JEJ1*Gut_EC_fraction*drug_fQ	0.011232
240	VillousJEJ2 = VGut*volumeRatio_JEJ2*Gut_EC_fraction*drug_fQ	0.0078682
241	VillousILL1 = VGut*volumeRatio_ILL1*Gut_EC_fraction*drug_fQ	0.0062718
242	VillousILL2 = VGut*volumeRatio_ILL2*Gut_EC_fraction*drug_fQ	0.0062718
243	VillousILL3 = VGut*volumeRatio_ILL3*Gut_EC_fraction*drug_fQ	0.0061578
244	VillousILL4 = VGut*volumeRatio_ILL4*Gut_EC_fraction*drug_fQ	0.0059297
245	Qmuc_DUO = drug_fQ*Q_artery_gut*flowRatio_DUO	26.9235
246	Qmuc_JEJ1 = drug_fQ*Q_artery_gut*flowRatio_JEJ1	74.0397
247	Qmuc_JEJ2 = drug_fQ*Q_artery_gut*flowRatio_JEJ2	74.0397
248	Qmuc_ILL1 = drug_fQ*Q_artery_gut*flowRatio_ILL1	32.7365
249	Qmuc_ILL2 = drug_fQ*Q_artery_gut*flowRatio_ILL2	32.7365

	Initial Assignments	Initial Value
250	Qmuc_ILL3 = drug_fQ*Q_artery_gut*flowRatio_ILL3	32.7365
251	Qmuc_ILL4 = drug_fQ*Q_artery_gut*flowRatio_ILL4	32.7365
252	Liver = phys_BW*(phys_Normalized_weight_liver_blood + phys_Normalized_weight_liver_tissue)*Specific_volume	1.603
253	Kp_liver = drug_Kp_liver_raw*switch_SFKp	1.1308
254	k_liver_bile = drug_PSbileg*switch_SFbile*phys_HPGL*(phys_BW*phys_Normalized_weight_liver_tissue*Specific_volume)/Specific_volume*drug_fuLiver	0
255	k_liver_metabolites = (switch_SFmet*((drug_HLM_CLint/drug_fumic*phys_MPGL)+(drug_CLmetg/drug_funic*phys_HPGL))*(phys_BW*phys_Normalized_weight_liver_tissue*Specific_volume)/Specific_volume)*drug_fuLiver	1.6185
256	Q_villi = phys_BW*phys_Normalized_Q_villi	336
257	DIFF_duo = HPeff_est*phys_Normalized_ESA*phys_BW*surfaceRatio_DUO	2.2356
258	DIFF_jej1 = HPeff_est*phys_Normalized_ESA*phys_BW*surfaceRatio_JEJ1	4.4129
259	DIFF_jej2 = HPeff_est*phys_Normalized_ESA*phys_BW*surfaceRatio_JEJ2	3.091
260	DIFF_ill1 = HPeff_est*phys_Normalized_ESA*phys_BW*surfaceRatio_ILL1	2.4689
261	DIFF_ill2 = HPeff_est*phys_Normalized_ESA*phys_BW*surfaceRatio_ILL2	2.4689
262	DIFF_ill3 = HPeff_est*phys_Normalized_ESA*phys_BW*surfaceRatio_ILL3	2.43
263	DIFF_ill4 = HPeff_est*phys_Normalized_ESA*phys_BW*surfaceRatio_ILL4	2.3328
264	DIFF_BASO_duo = HPeff_est_baso*phys_Normalized_ESA_baso*phys_BW*basoSurfaceRatio_DUO	0.057118
265	DIFF_BASO_jej1 = HPeff_est_baso*phys_Normalized_ESA_baso*phys_BW*basoSurfaceRatio_JEJ1	0.22
266	DIFF_BASO_jej2 = HPeff_est_baso*phys_Normalized_ESA_baso*phys_BW*basoSurfaceRatio_JEJ2	0.22
267	DIFF_BASO_ill1 = HPeff_est_baso*phys_Normalized_ESA_baso*phys_BW*basoSurfaceRatio_ILL1	0.14725
268	DIFF_BASO_ill2 = HPeff_est_baso*phys_Normalized_ESA_baso*phys_BW*basoSurfaceRatio_ILL2	0.14725
269	DIFF_BASO_ill3 = HPeff_est_baso*phys_Normalized_ESA_baso*phys_BW*basoSurfaceRatio_ILL3	0.14725

	Initial Assignments	Initial Value
270	DIFF_BASO_ill4 = HPeff_est_baso*phys_Normalized_ESA_baso*phys_BW*basoSurfaceRatio_ILL4	0.14725
271	k_artery_serosa = (1-drug_fQ)*Q_artery_gut	53.0431
272	k_serosa_liver = (1-drug_fQ)*Q_gut_liver/Kp_serosa*drug_BRP	45.6459
273	Kp_serosa = drug_Kp_serosa_raw*switch_SFKp	1.2434
274	HHINT = calculateHH(drug_inputFlag, REFPHSOL, drug_pKABase1, drug_pKABase2, drug_pKAAcid1, drug_pKAAcid2)	1
275	HHSTOMACH = calculateHH(drug_inputFlag, pHStomach, drug_pKABase1, drug_pKABase2, drug_pKAAcid1, drug_pKAAcid2)	1
276	HHDUO = calculateHH(drug_inputFlag, pHDuo, drug_pKABase1, drug_pKABase2, drug_pKAAcid1, drug_pKAAcid2)	1
277	HHJEJ1 = calculateHH(drug_inputFlag, pHJej1, drug_pKABase1, drug_pKABase2, drug_pKAAcid1, drug_pKAAcid2)	1
278	HHJEJ2 = calculateHH(drug_inputFlag, pHJej2, drug_pKABase1, drug_pKABase2, drug_pKAAcid1, drug_pKAAcid2)	1
279	HHILL1 = calculateHH(drug_inputFlag, pHIll1, drug_pKABase1, drug_pKABase2, drug_pKAAcid1, drug_pKAAcid2)	1
280	HHILL2 = calculateHH(drug_inputFlag, pHIll2, drug_pKABase1, drug_pKABase2, drug_pKAAcid1, drug_pKAAcid2)	1
281	HHILL3 = calculateHH(drug_inputFlag, pHIll3, drug_pKABase1, drug_pKABase2, drug_pKAAcid1, drug_pKAAcid2)	1
282	HHILL4 = calculateHH(drug_inputFlag, pHIll4, drug_pKABase1, drug_pKABase2, drug_pKAAcid1, drug_pKAAcid2)	1
283	CLINT_influx_DUO = CL_inf_api*phys_Normalized_ESA*phys_BW*surfaceRatio_DUO	0
284	CLINT_influx_JEJ1 = CL_inf_api*phys_Normalized_ESA*phys_BW*surfaceRatio_JEJ1	0
285	CLINT_influx_JEJ2 = CL_inf_api*phys_Normalized_ESA*phys_BW*surfaceRatio_JEJ2	0
286	CLINT_influx_ILL1 = CL_inf_api*phys_Normalized_ESA*phys_BW*surfaceRatio_ILL1	0
287	CLINT_influx_ILL2 = CL_inf_api*phys_Normalized_ESA*phys_BW*surfaceRatio_ILL2	0
288	CLINT_influx_ILL3 = CL_inf_api*phys_Normalized_ESA*phys_BW*surfaceRatio_ILL3	0
289	CLINT_influx_ILL4 = CL_inf_api*phys_Normalized_ESA*phys_BW*surfaceRatio	0

	Initial Assignments	Initial Value
	_ILL4	
290	CLINT_efflux_DUO = CL_eff*phys_Normalized_ESA*phys_BW*surfaceRatio_DUO	0
291	CLINT_efflux_JEJ1 = CL_eff*phys_Normalized_ESA*phys_BW*surfaceRatio_JEJ1	0
292	CLINT_efflux_JEJ2 = CL_eff*phys_Normalized_ESA*phys_BW*surfaceRatio_JEJ2	0
293	CLINT_efflux_ILL1 = CL_eff*phys_Normalized_ESA*phys_BW*surfaceRatio_ILL1	0
294	CLINT_efflux_ILL2 = CL_eff*phys_Normalized_ESA*phys_BW*surfaceRatio_ILL2	0
295	CLINT_efflux_ILL3 = CL_eff*phys_Normalized_ESA*phys_BW*surfaceRatio_ILL3	0
296	CLINT_efflux_ILL4 = CL_eff*phys_Normalized_ESA*phys_BW*surfaceRatio_ILL4	0
297	HPeff_est_baso = diff_baso	2.7e-06
298	CLINT_influx_baso_DUO = CL_inf_baso*phys_Normalized_ESA_baso*phys_BW*basoSurfaceRatio_DUO	0
299	CLINT_influx_baso_JEJ1 = CL_inf_baso*phys_Normalized_ESA_baso*phys_BW*basoSurfaceRatio_JEJ1	0
300	CLINT_influx_baso_JEJ2 = CL_inf_baso*phys_Normalized_ESA_baso*phys_BW*basoSurfaceRatio_JEJ2	0
301	CLINT_influx_baso_ILL1 = CL_inf_baso*phys_Normalized_ESA_baso*phys_BW*basoSurfaceRatio_ILL1	0
302	CLINT_influx_baso_ILL2 = CL_inf_baso*phys_Normalized_ESA_baso*phys_BW*basoSurfaceRatio_ILL2	0
303	CLINT_influx_baso_ILL3 = CL_inf_baso*phys_Normalized_ESA_baso*phys_BW*basoSurfaceRatio_ILL3	0
304	CLINT_influx_baso_ILL4 = CL_inf_baso*phys_Normalized_ESA_baso*phys_BW*basoSurfaceRatio_ILL4	0
305	Serosa = VGut*Gut_EC_fraction*(1-drug_fQ)	0.14284
306	diff_api = diff_baso	0.162
307	k_Liver_EC_S5_Venous = Q_li	84
308	phys_Normalized_ESA = phys_ESA/BW_average	1714.2857
309	phys_Normalized_ESA_baso = phys_ESA_baso/BW_average	95.7571
310	k_artery_serosa_1 = (1-drug_fQ)*Q_artery_gut_1	53.0431

	Initial Assignments	Initial Value
311	k_serosa_liver_1 = (1-drug_fQ)*Q_gut_liver_1/Kp_serosa_1*drug_BRP_1	66.5123
312	Kp_serosa_1 = drug_Kp_serosa_raw_1*switch_SFKp_1	0.51837
313	Qmuc_JEJ1_1 = drug_fQ*Q_artery_gut*flowRatio_JEJ1	74.0397
314	Qmuc_DUO_1 = drug_fQ*Q_artery_gut*flowRatio_DUO	26.9235
315	Qmuc_JEJ2_1 = drug_fQ*Q_artery_gut*flowRatio_JEJ2	74.0397
316	Qmuc_ILL1_1 = drug_fQ*Q_artery_gut*flowRatio_ILL1	32.7365
317	Qmuc_ILL2_1 = drug_fQ*Q_artery_gut*flowRatio_ILL2	32.7365
318	Qmuc_ILL3_1 = drug_fQ*Q_artery_gut*flowRatio_ILL3	32.7365
319	Qmuc_ILL4_1 = drug_fQ*Q_artery_gut*flowRatio_ILL4	32.7365
320	HPeff_est_baso_1 = diff_baso_1	1.6667e-05
321	DIFF_BASO_duo_1 = HPeff_est_baso_1*phys_Normalized_ESA_baso*phys_BW*basoSurfaceRatio_DUO	0.35258
322	DIFF_BASO_jej1_1 = HPeff_est_baso_1*phys_Normalized_ESA_baso*phys_BW*basoSurfaceRatio_JEJ1	1.358
323	DIFF_BASO_jej2_1 = HPeff_est_baso_1*phys_Normalized_ESA_baso*phys_BW*basoSurfaceRatio_JEJ2	1.358
324	DIFF_BASO_ill1_1 = HPeff_est_baso_1*phys_Normalized_ESA_baso*phys_BW*basoSurfaceRatio_ILL1	0.90893
325	DIFF_BASO_ill2_1 = HPeff_est_baso_1*phys_Normalized_ESA_baso*phys_BW*basoSurfaceRatio_ILL2	0.90893
326	DIFF_BASO_ill3_1 = HPeff_est_baso_1*phys_Normalized_ESA_baso*phys_BW*basoSurfaceRatio_ILL3	0.90893
327	DIFF_BASO_ill4_1 = HPeff_est_baso_1*phys_Normalized_ESA_baso*phys_BW*basoSurfaceRatio_ILL4	0.90893
328	CLINT_influx_baso_DUO_1 = CL_inf_baso_1*phys_Normalized_ESA_baso*phys_BW*basoSurfaceRatio_DUO	0.35258
329	CLINT_influx_baso_JEJ1_1 = CL_inf_baso_1*phys_Normalized_ESA_baso*phys_BW*basoSurfaceRatio_JEJ1	1.358
330	CLINT_influx_baso_JEJ2_1 = CL_inf_baso_1*phys_Normalized_ESA_baso*phys_BW*basoSurfaceRatio_JEJ2	1.358
331	CLINT_influx_baso_ILL1_1 = CL_inf_baso_1*phys_Normalized_ESA_baso*phys_BW*basoSurfaceRatio_ILL1	0.90893
332	CLINT_influx_baso_ILL2_1 = CL_inf_baso_1*phys_Normalized_ESA_baso*phys_BW*basoSurfaceRatio_ILL2	0.90893
333	CLINT_influx_baso_ILL3_1 = CL_inf_baso_1*phys_Normalized_ESA_baso*phys_BW*basoSurfaceRatio_ILL3	0.90893
334	CLINT_influx_baso_ILL4_1 = CL_inf_baso*phys_Normalized_ESA_baso*phys_BW*baso	0

	Initial Assignments	Initial Value
	SurfaceRatio_ILL4	
335	CLINT_influx_DUO_1 = CL_inf_api_1*phys_Normalized_ESA*phys_BW*surfaceRatio_DUO	13.8
336	CLINT_influx_JEJ1_1 = CL_inf_api_1*phys_Normalized_ESA*phys_BW*surfaceRatio_JEJ1	27.24
337	CLINT_influx_JEJ2_1 = CL_inf_api_1*phys_Normalized_ESA*phys_BW*surfaceRatio_JEJ2	19.08
338	CLINT_influx_ILL1_1 = CL_inf_api_1*phys_Normalized_ESA*phys_BW*surfaceRatio_ILL1	15.24
339	CLINT_influx_ILL2_1 = CL_inf_api_1*phys_Normalized_ESA*phys_BW*surfaceRatio_ILL2	15.24
340	CLINT_influx_ILL3_1 = CL_inf_api_1*phys_Normalized_ESA*phys_BW*surfaceRatio_ILL3	15
341	CLINT_influx_ILL4_1 = CL_inf_api_1*phys_Normalized_ESA*phys_BW*surfaceRatio_ILL4	14.4
342	HPeff_est_1 = diff_api_1	1.6667e-05
343	DIFF_duo_1 = HPeff_est_1*phys_Normalized_ESA*phys_BW*surfaceRatio_DUO	13.8
344	DIFF_jej1_1 = HPeff_est_1*phys_Normalized_ESA*phys_BW*surfaceRatio_JEJ1	27.24
345	DIFF_jej2_1 = HPeff_est_1*phys_Normalized_ESA*phys_BW*surfaceRatio_JEJ2	19.08
346	DIFF_ill1_1 = HPeff_est_1*phys_Normalized_ESA*phys_BW*surfaceRatio_ILL1	15.24
347	DIFF_ill2_1 = HPeff_est_1*phys_Normalized_ESA*phys_BW*surfaceRatio_ILL2	15.24
348	DIFF_ill3_1 = HPeff_est_1*phys_Normalized_ESA*phys_BW*surfaceRatio_ILL3	15
349	DIFF_ill4_1 = HPeff_est_1*phys_Normalized_ESA*phys_BW*surfaceRatio_ILL4	14.4
350	CLINT_efflux_DUO_1 = CL_eff_1*phys_Normalized_ESA*phys_BW*surfaceRatio_DUO	13.8
351	CLINT_efflux_JEJ1_1 = CL_eff_1*phys_Normalized_ESA*phys_BW*surfaceRatio_JEJ1	27.24
352	CLINT_efflux_JEJ2_1 = CL_eff_1*phys_Normalized_ESA*phys_BW*surfaceRatio_JEJ2	19.08

	Initial Assignments	Initial Value
353	CLINT_efflux_ILL1_1 = CL_eff_1*phys_Normalized_ESA*phys_BW*surfaceRatio_ILL1	15.24
354	CLINT_efflux_ILL2_1 = CL_eff_1*phys_Normalized_ESA*phys_BW*surfaceRatio_ILL2	15.24
355	CLINT_efflux_ILL3_1 = CL_eff_1*phys_Normalized_ESA*phys_BW*surfaceRatio_ILL3	15
356	CLINT_efflux_ILL4_1 = CL_eff_1*phys_Normalized_ESA*phys_BW*surfaceRatio_ILL4	14.4
357	SOLIF_STOMACH_1 = SOLINT_1*HHSTOMACH_1	1.3162
358	SOLIF_DUO_1 = SOLINT_1*HHDUO_1	1
359	SOLIF_JEJ1_1 = SOLINT_1*HHJEJ1_1	1
360	SOLIF_JEJ2_1 = SOLINT_1*HHJEJ2_1	1
361	SOLIF_ILL1_1 = SOLINT_1*HHILL1_1	1
362	SOLIF_ILL2_1 = SOLINT_1*HHILL2_1	1
363	SOLIF_ILL3_1 = SOLINT_1*HHILL3_1	1
364	SOLIF_ILL4_1 = SOLINT_1*HHILL4_1	1
365	SOLINT_1 = SOLFASSIF_1/HHINT_1	1
366	HHINT_1 = calculateHH(drug_inputFlag_1,REFPHSOL_1,drug_pKABase1_1,drug_pKABase2_1,drug_pKAAcid1_1,drug_pKAAcid2_1)	1
367	HHSTOMACH_1 = calculateHH(drug_inputFlag_1,pHStomach,drug_pKABase1_1,drug_pKABase2_1,drug_pKAAcid1_1,drug_pKAAcid2_1)	1.3162
368	HHDUO_1 = calculateHH(drug_inputFlag_1, pHDuo, drug_pKABase1_1, drug_pKABase2_1, drug_pKAAcid1_1, drug_pKAAcid2_1)	1
369	HHJEJ1_1 = calculateHH(drug_inputFlag_1, pHJej1, drug_pKABase1_1, drug_pKABase2_1, drug_pKAAcid1_1, drug_pKAAcid2_1)	1
370	HHJEJ2_1 = calculateHH(drug_inputFlag_1, pHJej2, drug_pKABase1_1, drug_pKABase2_1, drug_pKAAcid1_1, drug_pKAAcid2_1)	1
371	HHILL1_1 = calculateHH(drug_inputFlag_1, pHIll1, drug_pKABase1_1, drug_pKABase2_1, drug_pKAAcid1_1, drug_pKAAcid2_1)	1
372	HHILL2_1 = calculateHH(drug_inputFlag_1, pHIll2, drug_pKABase1_1, drug_pKABase2_1, drug_pKAAcid1_1, drug_pKAAcid2_1)	1
373	HHILL3_1 = calculateHH(drug_inputFlag_1, pHIll3, drug_pKABase1_1, drug_pKABase2_1, drug_pKAAcid1_1, drug_pKAAcid2_1)	1
374	HHILL4_1 = calculateHH(drug_inputFlag_1, pHIll4, drug_pKABase1_1, drug_pKABase2_1, drug_pKAAcid1_1, drug_pKAAcid2_1)	1
375	KD_1 = 3*DIFFCOEFF_1/(PDENSITY_1*PSIZE_1*DLT_1)	0.0002

	Initial Assignments	Initial Value
376	NI_DUO_1 = 1/HHDUO_1	1
377	NI_JEJ1_1 = 1/HHJEJ1_1	1
378	NI_JEJ2_1 = 1/HHJEJ2_1	1
379	NI_ILL1_1 = 1/HHILL1_1	1
380	NI_ILL2_1 = 1/HHILL2_1	1
381	NI_ILL3_1 = 1/HHILL3_1	1
382	NI_ILL4_1 = 1/HHILL4_1	1
383	Serosa_1 = VGut*Gut_EC_fraction*(1-drug_fQ)	0.14284
384	STOMACH_1 = StomachLumenTotal/BW_average*phys_BW	0.05
385	VDUO_1 = V_LUM_TOT*lumenvolumeRatio_DUO	0.03435
386	VJEJ1_1 = V_LUM_TOT*lumenvolumeRatio_JEJ1	0.0211
387	VJEJ2_1 = V_LUM_TOT*lumenvolumeRatio_JEJ2	0.0211
388	VILL1_1 = V_LUM_TOT*lumenvolumeRatio_ILL1	0.0126
389	VILL2_1 = V_LUM_TOT*lumenvolumeRatio_ILL2	0.0126
390	VILL3_1 = V_LUM_TOT*lumenvolumeRatio_ILL3	0.0126
391	VILL4_1 = V_LUM_TOT*lumenvolumeRatio_ILL4	0.0126
392	MDUO_1 = VGut*volumeRatio_DUO*Gut_IC_fraction	0.037454
393	MJEJ1_1 = VGut*volumeRatio_JEJ1*Gut_IC_fraction	0.073785
394	MJEJ2_1 = VGut*volumeRatio_JEJ2*Gut_IC_fraction	0.051687
395	MILL1_1 = VGut*volumeRatio_ILL1*Gut_IC_fraction	0.0412
396	MILL2_1 = VGut*volumeRatio_ILL2*Gut_IC_fraction	0.0412
397	MILL3_1 = VGut*volumeRatio_ILL3*Gut_IC_fraction	0.04045
398	MILL4_1 = VGut*volumeRatio_ILL4*Gut_IC_fraction	0.038952
399	VillousDUO_1 = VGut*volumeRatio_DUO*Gut_EC_fraction*drug_fQ	0.0057016
400	VillousJEJ1_1 = VGut*volumeRatio_JEJ1*Gut_EC_fraction*drug_fQ	0.011232
401	VillousJEJ2_1 = VGut*volumeRatio_JEJ2*Gut_EC_fraction*drug_fQ	0.0078682
402	VillousILL1_1 = VGut*volumeRatio_ILL1*Gut_EC_fraction*drug_fQ	0.0062718
403	VillousILL2_1 = VGut*volumeRatio_ILL2*Gut_EC_fraction*drug_fQ	0.0062718
404	VillousILL3_1 = VGut*volumeRatio_ILL3*Gut_EC_fraction*drug_fQ	0.0061578
405	VillousILL4_1 = VGut*volumeRatio_ILL4*Gut_EC_fraction*drug_fQ	0.0059297
406	Liver_1 = phys_BW*(phys_Normalized_weight_liver_blood + phys_Normalized_weight_liver_tissue)*Specific_volume	1.603
407	Kp_liver_1 = drug_Kp_liver_raw_1*switch_SFKp_1	0.51837
408	k_liver_metabolites_1 = (switch_SFmet_1*((drug_HLM_CLint_1/drug_fumic_1*phys_MPGL)+(drug_CLmetg_1/drug_funic_1*phys_HPGL))*(phys_BW*phys_Normalized_weight_liver_tissue*Specific_volume)/Specific_volume)*drug_fuLiver_1	1.0507

	Initial Assignments	Initial Value
409	k_Liver_Venous_1 = Q_li_1/Kp_liver_1*drug_BRP_1	105.3302
410	k_liver_bile_1 = drug_PSbileg_1*switch_SFbile_1*phys_HPGL*(phys_BW*phys_Normalized_weight_liver_tissue*Specific_volume)/Specific_volume*drug_fuLiver_1	0.098292
411	LOGSR_1 = 0.75*LOGP_1+2.27	3.02
412	CLINT_efflux_baso_DUO = CL_eff_baso*phys_Normalized_ESA_baso*phys_BW*basoSurfaceRatio_DUO	0
413	CLINT_efflux_baso_ILL1 = CL_eff_baso*phys_Normalized_ESA_baso*phys_BW*basoSurfaceRatio_ILL1	0
414	CLINT_efflux_baso_ILL2 = CL_eff_baso*phys_Normalized_ESA_baso*phys_BW*basoSurfaceRatio_ILL2	0
415	CLINT_efflux_baso_ILL3 = CL_eff_baso*phys_Normalized_ESA_baso*phys_BW*basoSurfaceRatio_ILL3	0
416	CLINT_efflux_baso_ILL4 = CL_eff_baso*phys_Normalized_ESA_baso*phys_BW*basoSurfaceRatio_ILL4	0
417	CLINT_efflux_baso_JEJ1 = CL_eff_baso*phys_Normalized_ESA_baso*phys_BW*basoSurfaceRatio_JEJ1	0
418	CLINT_efflux_baso_JEJ2 = CL_eff_baso*phys_Normalized_ESA_baso*phys_BW*basoSurfaceRatio_JEJ2	0
419	CLINT_efflux_baso_DUO_1 = CL_eff_baso_1*phys_Normalized_ESA_baso*phys_BW*basoSurfaceRatio_DUO	0
420	CLINT_efflux_baso_JEJ1_1 = CL_eff_baso_1*phys_Normalized_ESA_baso*phys_BW*basoSurfaceRatio_JEJ1	0
421	CLINT_efflux_baso_JEJ2_1 = CL_eff_baso_1*phys_Normalized_ESA_baso*phys_BW*basoSurfaceRatio_JEJ2	0
422	CLINT_efflux_baso_ILL1_1 = CL_eff_baso_1*phys_Normalized_ESA_baso*phys_BW*basoSurfaceRatio_ILL1	0
423	CLINT_efflux_baso_ILL2_1 = CL_eff_baso_1*phys_Normalized_ESA_baso*phys_BW*basoSurfaceRatio_ILL2	0
424	CLINT_efflux_baso_ILL3_1 = CL_eff_baso_1*phys_Normalized_ESA_baso*phys_BW*basoSurfaceRatio_ILL3	0
425	CLINT_efflux_baso_ILL4_1 = CL_eff_baso_1*phys_Normalized_ESA_baso*phys_BW*basoSurfaceRatio_ILL4	0
426	diff_api_1 = diff_baso_1	1

Repeated Assignments

	Repeated Assignments	Initial Value
1	k_Liver_IC_S5_Metabolites = (switch_Vmax_met*phys_BW/(drug_Km_met+drug_fuLiver*Liver_IC_S5.Liver_IC_S5_drug/drug_molar_mass)+switch_SFmet*((drug_HLM_CLint/drug_fumic*phys_MPGL)+(drug_CLmetg/drug_funic*phys_HPGL))*(phys_BW*phys_Normalized_weight_liver_tissue*Specific_volume)/Specific_volume)/5*drug_fuLiver	0.3237
2	k_Liver_IC_S4_Metabolites = (switch_Vmax_met*phys_BW/(drug_Km_met+drug_fuLiver*Liver_IC_S4.Liver_IC_S4_drug/drug_molar_mass)+switch_SFmet*((drug_HLM_CLint/drug_fumic*phys_MPGL)+(drug_CLmetg/drug_funic*phys_HPGL))*(phys_BW*phys_Normalized_weight_liver_tissue*Specific_volume)/Specific_volume)/5*drug_fuLiver	0.3237
3	k_Liver_IC_S3_Metabolites = (switch_Vmax_met*phys_BW/(drug_Km_met+drug_fuLiver*Liver_IC_S3.Liver_IC_S3_drug/drug_molar_mass)+switch_SFmet*((drug_HLM_CLint/drug_fumic*phys_MPGL)+(drug_CLmetg/drug_funic*phys_HPGL))*(phys_BW*phys_Normalized_weight_liver_tissue*Specific_volume)/Specific_volume)/5*drug_fuLiver	0.3237
4	k_Liver_IC_S2_Metabolites = (switch_Vmax_met*phys_BW/(drug_Km_met+drug_fuLiver*Liver_IC_S2.Liver_IC_S2_drug/drug_molar_mass)+switch_SFmet*((drug_HLM_CLint/drug_fumic*phys_MPGL)+(drug_CLmetg/drug_funic*phys_HPGL))*(phys_BW*phys_Normalized_weight_liver_tissue*Specific_volume)/Specific_volume)/5*drug_fuLiver	0.3237
5	k_Liver_IC_S1_Metabolites = (switch_Vmax_met*phys_BW/(drug_Km_met+drug_fuLiver*Liver_IC_S1.Liver_IC_S1_drug/drug_molar_mass)+switch_SFmet*((drug_HLM_CLint/drug_fumic*phys_MPGL)+(drug_CLmetg/drug_funic*phys_HPGL))*(phys_BW*phys_Normalized_weight_liver_tissue*Specific_volume)/Specific_volume)/5*drug_fuLiver	0.3237
6	k_Liver_EC_S5_Liver_IC_S5 = (drug_PSdifg*switch_SFdiff*phys_HPGL*(phys_BW*phys_Normalized_weight_liver_tissue*Specific_volume)/Specific_volume+switch_Vmax_uptake*phys_BW/(drug_Km_uptake+drug_fB*Liver_EC_S5.Liver_EC_S5_drug/drug_molar_mass)+drug_PSinfg*switch_SFinf*phys_HPGL*(phys_BW*phys_Normalized_weight_liver_tissue*Specific_volume)/Specific_volume)/5*drug_fB	1894.1175
7	k_Liver_EC_S4_Liver_IC_S4 = (drug_PSdifg*switch_SFdiff*phys_HPGL*(phys_BW*phys_Normalized_weight_liver_tissue*Specific_volume)/Specific_volume+switch_Vmax_uptake*phys_BW/(drug_Km_uptake+drug_fB*Liver_EC_S4.Liver_EC_S4_drug/drug_molar_mass)+drug_PSinfg*switch_SFinf*phys_HPGL*(phys_BW*phys_Normalized_weight_liver_tissue*Specific_volume)/Specific_volume)/5*drug_fB	1894.1175
8	k_Liver_EC_S3_Liver_IC_S3 = (drug_PSdifg*switch_SFdiff*phys_HPGL*(phys_BW*phys_Normalized_weight_liver_tissue*Specific_volume)/Specific_volume+switch_Vmax_uptake*phys_BW/(drug_Km_uptake+drug_fB*Liver_EC_S3.Liver_EC_S3_drug/drug_molar_mass)+drug_PSinfg*switch_SFinf*phys_HPGL*	1894.1175

	Repeated Assignments	Initial Value
	(phys_BW*phys_Normalized_weight_liver_tissue*Specific_volume)/Specific_volume)/5*drug_fB	
9	k_Liver_EC_S2_Liver_IC_S2 = (drug_PSdifg*switch_SFdiff*phys_HPGL*(phys_BW*phys_Normalized_weight_liver_tissue*Specific_volume)/Specific_volume+switch_Vmax_uptake*phys_BW/(drug_Km_uptake+drug_fB*Liver_EC_S2.Liver_EC_S2_drug/drug_molar_mass)+drug_PSinfg*switch_SFinf*phys_HPGL*(phys_BW*phys_Normalized_weight_liver_tissue*Specific_volume)/Specific_volume)/5*drug_fB	1894.1175
10	k_Liver_EC_S1_Liver_IC_S1 = (drug_PSdifg*switch_SFdiff*phys_HPGL*(phys_BW*phys_Normalized_weight_liver_tissue*Specific_volume)/Specific_volume+switch_Vmax_uptake*phys_BW/(drug_Km_uptake+drug_fB*Liver_EC_S1.Liver_EC_S1_drug/drug_molar_mass)+drug_PSinfg*switch_SFinf*phys_HPGL*(phys_BW*phys_Normalized_weight_liver_tissue*Specific_volume)/Specific_volume)/5*drug_fB	1894.1175
11	Blood_total.Blood_total_drug = (Artery.Artery_drug*Artery+Venous.Venous_drug*Venous)/(Artery+Venous)	0
12	Plasma_total.Plasma_total_drug = Blood_total.Blood_total_drug/drug_BRP	0
13	Portal.Portal_drug = (Q_gut_liver/numIntestinalCompartments*(VillousDUO.Villous_DUO/VillousDUO+VillousJEJ1.Villous_JEJ1/VillousJEJ1+VillousJEJ2.Villous_JEJ2/VillousJEJ2+VillousILL1.Villous_ILL1/VillousILL1+VillousILL2.Villous_ILL2/VillousILL2+VillousILL3.Villous_ILL3/VillousILL3+VillousILL4.Villous_ILL4/VillousILL4)+Q_spleen_liver*(Spleen.Spleen_drug/Kp_spleen*drug_BRP))/(Q_gut_liver+Q_spleen_liver)	0
14	Portal_1.Portal_plasma_drug = Portal.Portal_drug/drug_BRP	0
15	Mass_Balance.Amount_body = Venous.Venous_drug*Venous+Artery.Artery_drug*Artery+(1-switch_liverFlag)*(Liver_IC_S1.Liver_IC_S1_drug*Liver_IC_S1+Liver_IC_S2.Liver_IC_S2_drug*Liver_IC_S2+Liver_IC_S3.Liver_IC_S3_drug*Liver_IC_S3+Liver_IC_S4.Liver_IC_S4_drug*Liver_IC_S4+Liver_IC_S5.Liver_IC_S5_drug*Liver_IC_S5+Liver_EC_S1.Liver_EC_S1_drug*Liver_EC_S1+Liver_EC_S2.Liver_EC_S2_drug*Liver_EC_S2+Liver_EC_S3.Liver_EC_S3_drug*Liver_EC_S3+Liver_EC_S4.Liver_EC_S4_drug*Liver_EC_S4+Liver_EC_S5.Liver_EC_S5_drug*Liver_EC_S5)+switch_liverFlag*Liver.Liver_drug*Liver+Lung.Lung_drug*Lung+Adipose.Adipose_drug*Adipose+Heart.Heart_drug*Heart+Muscle.Muscle_drug*Muscle+Skin.Skin_drug*Skin+Kidney.Kidney_drug*Kidney+Bone.Bone_drug*Bone+Testes.Testes_drug*Testes+Rest.Rest_drug*Rest+Gut.Gut_drug*Gut+MDUO.MEM_DUO*milligram_per_microgram+MJEJ1.MEM_JEJ1*milligram_per_microgram+MJEJ2.MEM_JEJ2*milligram_per_microgram+MILL1.MEM_ILL1*milligram_per_microgram+MILL2.MEM_ILL2*milligram_per_microgram+MILL3.MEM_ILL3*milligram_per_microgram+MILL4.MEM_ILL4*milligram_per_microgram+Spleen.Spleen_drug*Spleen+Brain.Brain_drug*Brain+VillousDUO.Villous_DUO+VillousJEJ1.Villous_JEJ1+VillousJEJ2.Villous_JEJ2+VillousILL1.Villous_ILL1+VillousILL2.Villous_ILL2+VillousILL3.Villou	0

	Repeated Assignments	Initial Value
	s_ILL3+VillousILL4.Villous_ILL4+Serosa*Serosa.Serosa_drug	
16	Mass_Balance.Amount_total = Venous.Venous_drug*Venous+Artery.Artery_drug*Artery+(1-switch_liverFlag)*(Liver_IC_S1.Liver_IC_S1_drug*Liver_IC_S1+Liver_IC_S2.Liver_IC_S2_drug*Liver_IC_S2+Liver_IC_S3.Liver_IC_S3_drug*Liver_IC_S3+Liver_IC_S4.Liver_IC_S4_drug*Liver_IC_S4+Liver_IC_S5.Liver_IC_S5_drug*Liver_IC_S5+Liver_EC_S1.Liver_EC_S1_drug*Liver_EC_S1+Liver_EC_S2.Liver_EC_S2_drug*Liver_EC_S2+Liver_EC_S3.Liver_EC_S3_drug*Liver_EC_S3+Liver_EC_S4.Liver_EC_S4_drug*Liver_EC_S4+Liver_EC_S5.Liver_EC_S5_drug*Liver_EC_S5)+switch_liverFlag*Liver.Liver_drug*Liver+Lung.Lung_drug*Lung+Adipose.Adipose_drug*Adipose+Heart.Heart_drug*Heart+Muscle.Muscle_drug*Muscle+Skin.Skin_drug*Skin+Kidney.Kidney_drug*Kidney+Bone.Bone_drug*Bone+Testes.Testes_drug*Testes+Rest.Rest_drug*Rest+(MDUO.MEM_DUO+MJEJ1.MEM_JEJ1+MJEJ2.MEM_JEJ2+MILL1.MEM_ILL1+MILL2.MEM_ILL2+MILL3.MEM_ILL3+MILL4.MEM_ILL4)*milligram_per_microgram+Spleen.Spleen_drug*Spleen+Brain.Brain_drug*Brain+Main_compartment.Bile_drug+Urine.Urine_drug+Gut_Lumen.Gut_Lumen_drug+(STOMACH.X_STOMACH_DISS+VDUO.X_DUO_DISS+VJEJ1.X_JEJ1_DISS+VJEJ2.X_JEJ2_DISS+VILL1.X_ILL1_DISS+VILL2.X_ILL2_DISS+VILL3.X_ILL3_DISS+VILL4.X_ILL4_DISS+Colon.X_CECUM_DISS)*milligram_per_microgram+(STOMACH.X_STOMACH_SOLID+VDUO.X_DUO_SOLID+VJEJ1.X_JEJ1_SOLID+VJEJ2.X_JEJ2_SOLID+VILL1.X_ILL1_SOLID+VILL2.X_ILL2_SOLID+VILL3.X_ILL3_SOLID+VILL4.X_ILL4_SOLID+Colon.X_CECUM_SOLID)*milligram_per_microgram+VillousDUO.Villous_DUO+VillousJEJ1.Villous_JEJ1+VillousJEJ2.Villous_JEJ2+VillousILL1.Villous_ILL1+VillousILL2.Villous_ILL2+VillousILL3.Villous_ILL3+VillousILL4.Villous_ILL4+Serosa.Serosa_drug*Serosa	0
17	Plasma_total.Plasma_free_uM = Plasma_total.Plasma_total_drug*drug_fuplasma/drug_molar_mass	0
18	Liver_total.Liver_blood_total = (1-switch_liverFlag)*((Liver_EC_S1.Liver_EC_S1_drug+Liver_EC_S2.Liver_EC_S2_drug+Liver_EC_S3.Liver_EC_S3_drug+Liver_EC_S4.Liver_EC_S4_drug+Liver_EC_S5.Liver_EC_S5_drug)/5)	0
19	Liver_total.Liver_tissue_total = (1-switch_liverFlag)*((Liver_IC_S1.Liver_IC_S1_drug+Liver_IC_S2.Liver_IC_S2_drug+Liver_IC_S3.Liver_IC_S3_drug+Liver_IC_S4.Liver_IC_S4_drug+Liver_IC_S5.Liver_IC_S5_drug)/5)	0
20	Liver_total.Liver_blood_free = Liver_total.Liver_blood_total*drug_fuplasma/drug_BRP	0
21	Liver_total.Liver_tissue_free = Liver_total.Liver_tissue_total*drug_fuLiver	0
22	convert_to_nmole_per_kg.Adipose_nmole = Adipose.Adipose_drug*Adipose/drug_molar_mass*nanomole_per_mole*kilogram/phys_BW	0
23	convert_to_nmole_per_kg.Artery_nmole = Artery.Artery_drug*Artery/drug_molar_mass*nanomole_per_mole*kilogram/phys_BW	0
24	convert_to_nmole_per_kg.Bone_nmole = Bone.Bone_drug*Bone/drug_molar_mass*nanomole_per_	0

	Repeated Assignments	Initial Value
	mole*kilogram/phys_BW	
25	convert_to_nmole_per_kg.Liver_EC1_nmole = Liver_EC_S1.Liver_EC_S1_drug*Liver_EC_S1/drug_molar_mass*nanomole_per_mole*kilogram/phys_BW	0
26	convert_to_nmole_per_kg.Liver_IC1_nmole = Liver_IC_S1.Liver_IC_S1_drug*Liver_IC_S1/drug_molar_mass*nanomole_per_mole*kilogram/phys_BW	0
27	convert_to_nmole_per_kg.Muscle_nmole = Muscle.Muscle_drug*Muscle/drug_molar_mass*nanomole_per_mole*kilogram/phys_BW	0
28	convert_to_nmole_per_kg.Urine_nmole = Urine.Urine_drug/drug_molar_mass*nanomole_per_mole*kilogram/phys_BW	0
29	convert_to_nmole_per_kg.Venous_nmole = Venous.Venous_drug*Venous/drug_molar_mass*nanomole_per_mole*kilogram/phys_BW	0
30	convert_to_nmole_per_kg.Bile_nmole = Main_compartment.Bile_drug/drug_molar_mass*nanomole_per_mole*kilogram/phys_BW	0
31	Plasma_total.Plasma_total_uM = Plasma_total.Plasma_total_drug/drug_molar_mass	0
32	Liver_total.Liver_tissue_total_uM = Liver_total.Liver_tissue_total/drug_molar_mass	0
33	convert_to_nmole_per_kg.Kidney_nmole = Kidney.Kidney_drug*Kidney/drug_molar_mass*nanomole_per_mole*kilogram/phys_BW	0
34	convert_to_nmole_per_kg.Lung_nmole = Lung.Lung_drug*Lung/drug_molar_mass*nanomole_per_mole*kilogram/phys_BW	0
35	convert_to_nmole_per_kg.Metabolites_nmole = Metabolites.Metabolites_drug/drug_molar_mass*nanomole_per_mole*kilogram/phys_BW	0
36	convert_to_nmole_per_kg.Liver_IC2_nmole = Liver_IC_S2.Liver_IC_S2_drug*Liver_IC_S2/drug_molar_mass*nanomole_per_mole*kilogram/phys_BW	0
37	convert_to_nmole_per_kg.Liver_EC2_nmole = Liver_EC_S2.Liver_EC_S2_drug*Liver_EC_S2/drug_molar_mass*nanomole_per_mole*kilogram/phys_BW	0
38	convert_to_nmole_per_kg.Liver_EC3_nmole = Liver_EC_S3.Liver_EC_S3_drug*Liver_EC_S3/drug_molar_mass*nanomole_per_mole*kilogram/phys_BW	0
39	convert_to_nmole_per_kg.Liver_IC3_nmole = Liver_IC_S3.Liver_IC_S3_drug*Liver_IC_S3/drug_molar_mass*nanomole_per_mole*kilogram/phys_BW	0
40	convert_to_nmole_per_kg.Liver_IC4_nmole = Liver_IC_S4.Liver_IC_S4_drug*Liver_IC_S4/drug_molar_mass*nanomole_per_mole*kilogram/phys_BW	0
41	convert_to_nmole_per_kg.Liver_EC4_nmole = Liver_EC_S4.Liver_EC_S4_drug*Liver_EC_S4/drug_molar_mass*nanomole_per_mole*kilogram/phys_BW	0
42	convert_to_nmole_per_kg.Liver_EC5_nmole = Liver_EC_S5.Liver_EC_S5_drug*Liver_EC_S5/drug_molar_mass*nanomole_per_mole*kilogram/phys_BW	0

	Repeated Assignments	Initial Value
43	convert_to_nmole_per_kg.Liver_IC5_nmole = Liver_IC_S5.Liver_IC_S5_drug*Liver_IC_S5/drug_molar_mass*nanomole_per_mole*kilogram/phys_BW	0
44	convert_to_nmole_per_kg.Gut_nmole = Gut.Gut_drug*Gut/drug_molar_mass*nanomole_per_mole*kilogram/phys_BW	0
45	convert_to_nmole_per_kg.Spleen_nmole = Spleen.Spleen_drug*Spleen/drug_molar_mass*nanomole_per_mole*kilogram/phys_BW	0
46	convert_to_nmole_per_kg.Skin_nmole = Skin.Skin_drug*Skin/drug_molar_mass*nanomole_per_mole*kilogram/phys_BW	0
47	convert_to_nmole_per_kg.Brain_nmole = Brain.Brain_drug*Brain/drug_molar_mass*nanomole_per_mole*kilogram/phys_BW	0
48	convert_to_nmole_per_kg.Rest_nmole = Rest.Rest_drug*Rest/drug_molar_mass*nanomole_per_mole*kilogram/phys_BW	0
49	convert_to_nmole_per_kg.Heart_nmole = Heart.Heart_drug*Heart/drug_molar_mass*nanomole_per_mole*kilogram/phys_BW	0
50	k_Liver_IC_S5_Metabolites_1 = met_inhib_S5*(switch_Vmax_met_1*phys_BW/(drug_Km_met_1+drug_fuLiver_1*Liver_IC_S5_1.Liver_IC_S5_drug_1/drug_molar_mass_1)+switch_SFmet_1*((drug_HLM_CLint_1/drug_fumic_1*phys_MPGL)+(drug_CLmetg_1/drug_funic_1*phys_HPGL))*(phys_BW*phys_Normalized_weight_liver_tissue*Specific_volume)/Specific_volume)/5*drug_fuLiver_1	0.21013
51	k_Liver_IC_S4_Metabolites_1 = met_inhib_S4*(switch_Vmax_met_1*phys_BW/(drug_Km_met_1+drug_fuLiver_1*Liver_IC_S4_1.Liver_IC_S4_drug_1/drug_molar_mass_1)+switch_SFmet_1*((drug_HLM_CLint_1/drug_fumic_1*phys_MPGL)+(drug_CLmetg_1/drug_funic_1*phys_HPGL))*(phys_BW*phys_Normalized_weight_liver_tissue*Specific_volume)/Specific_volume)/5*drug_fuLiver_1	0.21013
52	k_Liver_IC_S3_Metabolites_1 = met_inhib_S3*(switch_Vmax_met_1*phys_BW/(drug_Km_met_1+drug_fuLiver_1*Liver_IC_S3_1.Liver_IC_S3_drug_1/drug_molar_mass_1)+switch_SFmet_1*((drug_HLM_CLint_1/drug_fumic_1*phys_MPGL)+(drug_CLmetg_1/drug_funic_1*phys_HPGL))*(phys_BW*phys_Normalized_weight_liver_tissue*Specific_volume)/Specific_volume)/5*drug_fuLiver_1	0.21013
53	k_Liver_IC_S2_Metabolites_1 = met_inhib_S2*(switch_Vmax_met_1*phys_BW/(drug_Km_met_1+drug_fuLiver_1*Liver_IC_S2_1.Liver_IC_S2_drug_1/drug_molar_mass_1)+switch_SFmet_1*((drug_HLM_CLint_1/drug_fumic_1*phys_MPGL)+(drug_CLmetg_1/drug_funic_1*phys_HPGL))*(phys_BW*phys_Normalized_weight_liver_tissue*Specific_volume)/Specific_volume)/5*drug_fuLiver_1	0.21013
54	k_Liver_IC_S1_Metabolites_1 = met_inhib_S1*(switch_Vmax_met_1*phys_BW/(drug_Km_met_1+drug_fuLiver_1*Liver_IC_S1_1.Liver_IC_S1_drug_1/drug_molar_mass_1)+switch_SFmet_1*((drug_HLM_CLint_1/drug_fumic_1*phys_MPGL)+	0.21013

	Repeated Assignments	Initial Value
	(drug_CLmetg_1/drug_funic_1*phys_HPGL))*(phys_BW*phys_Normalized_weight_liver_tissue*Specific_volume)/Specific_volume)/5*drug_fuLiver_1	
55	k_Liver_IC_S5_Bile_1 = biliary_inhib_S5*drug_PSbileg_1*switch_SFbile_1*phys_HPGL*(phys_BW*phys_Normalized_weight_liver_tissue*Specific_volume)/Specific_volume/5*drug_fuLiver_1	0.019658
56	k_Liver_IC_S4_Bile_1 = biliary_inhib_S4*drug_PSbileg_1*switch_SFbile_1*phys_HPGL*(phys_BW*phys_Normalized_weight_liver_tissue*Specific_volume)/Specific_volume/5*drug_fuLiver_1	0.019658
57	k_Liver_IC_S3_Bile_1 = biliary_inhib_S3*drug_PSbileg_1*switch_SFbile_1*phys_HPGL*(phys_BW*phys_Normalized_weight_liver_tissue*Specific_volume)/Specific_volume/5*drug_fuLiver_1	0.019658
58	k_Liver_IC_S2_Bile_1 = biliary_inhib_S2*drug_PSbileg_1*switch_SFbile_1*phys_HPGL*(phys_BW*phys_Normalized_weight_liver_tissue*Specific_volume)/Specific_volume/5*drug_fuLiver_1	0.019658
59	k_Liver_IC_S1_Bile_1 = biliary_inhib_S1*drug_PSbileg_1*switch_SFbile_1*phys_HPGL*(phys_BW*phys_Normalized_weight_liver_tissue*Specific_volume)/Specific_volume/5*drug_fuLiver_1	0.019658
60	k_Liver_EC_S5_Liver_IC_S5_1 = (drug_PSdifg_1*switch_SFdiff_1*phys_HPGL*(phys_BW*phys_Normalized_weight_liver_tissue*Specific_volume)/Specific_volume+uptake_inhib_S5*switch_Vmax_uptake_1*phys_BW/(drug_Km_uptake_1+drug_fB_1*Liver_EC_S5_1.Liver_EC_S5_drug_1/drug_molar_mass_1)+uptake_inhib_S5*drug_PSinfg_1*switch_SFinf_1*phys_HPGL*(phys_BW*phys_Normalized_weight_liver_tissue*Specific_volume)/Specific_volume)/5*drug_fB_1	168.0698
61	k_Liver_EC_S4_Liver_IC_S4_1 = (drug_PSdifg_1*switch_SFdiff_1*phys_HPGL*(phys_BW*phys_Normalized_weight_liver_tissue*Specific_volume)/Specific_volume+uptake_inhib_S4*switch_Vmax_uptake_1*phys_BW/(drug_Km_uptake_1+drug_fB_1*Liver_EC_S4_1.Liver_EC_S4_drug_1/drug_molar_mass_1)+uptake_inhib_S4*drug_PSinfg_1*switch_SFinf_1*phys_HPGL*(phys_BW*phys_Normalized_weight_liver_tissue*Specific_volume)/Specific_volume)/5*drug_fB_1	168.0698
62	k_Liver_EC_S3_Liver_IC_S3_1 = (drug_PSdifg_1*switch_SFdiff_1*phys_HPGL*(phys_BW*phys_Normalized_weight_liver_tissue*Specific_volume)/Specific_volume+uptake_inhib_S3*switch_Vmax_uptake_1*phys_BW/(drug_Km_uptake_1+drug_fB_1*Liver_EC_S3_1.Liver_EC_S3_drug_1/drug_molar_mass_1)+uptake_inhib_S3*drug_PSinfg_1*switch_SFinf_1*phys_HPGL*(phys_BW*phys_Normalized_weight_liver_tissue*Specific_volume)/Specific_volume)/5*drug_fB_1	168.0698
63	k_Liver_EC_S2_Liver_IC_S2_1 = (drug_PSdifg_1*switch_SFdiff_1*phys_HPGL*	168.0698

	Repeated Assignments	Initial Value
	(phys_BW*phys_Normalized_weight_liver_tissue*Specific_volume)/Specific_volume+uptake_inhib_S2*switch_Vmax_uptake_1*phys_BW/(drug_Km_uptake_1+drug_fB_1*Liver_EC_S2_1.Liver_EC_S2_drug_1/drug_molar_mass_1)+uptake_inhib_S2*drug_PSinfg_1*switch_SFinf_1*phys_HPGL*(phys_BW*phys_Normalized_weight_liver_tissue*Specific_volume)/Specific_volume)/5*drug_fB_1	
64	k_Liver_EC_S1_Liver_IC_S1_1 = (drug_PSdifg_1*switch_SFdiff_1*phys_HPGL*(phys_BW*phys_Normalized_weight_liver_tissue*Specific_volume)/Specific_volume+uptake_inhib_S1*switch_Vmax_uptake_1*phys_BW/(drug_Km_uptake_1+drug_fB_1*Liver_EC_S1_1.Liver_EC_S1_drug_1/drug_molar_mass_1)+uptake_inhib_S1*drug_PSinfg_1*switch_SFinf_1*phys_HPGL*(phys_BW*phys_Normalized_weight_liver_tissue*Specific_volume)/Specific_volume)/5*drug_fB_1	168.0698
65	Blood_total_1.Blood_total_drug_1 = (Artery_1.Artery_drug_1*Artery_1+Venous_1.Venous_drug_1*Venous_1)/(Artery_1+Venous_1)	0
66	Plasma_total_1.Plasma_total_drug_1 = Blood_total_1.Blood_total_drug_1/drug_BRP_1	0
67	Portal_1.Portal_drug_1 = (Q_gut_liver_1*(Gut_1.Gut_drug_1/Kp_gut_1*drug_BRP_1)+Q_spleen_liver_1*(Spleen_1.Spleen_drug_1/Kp_spleen_1*drug_BRP_1))/(Q_gut_liver_1+Q_spleen_liver_1)	0
68	Portal_1.Portal_plasma_drug_1 = Portal_1.Portal_drug_1/drug_BRP_1	0
69	Mass_Balance_1.Amount_body_1 = Venous_1.Venous_drug_1*Venous_1+Artery_1.Artery_drug_1*Artery_1+Liver_IC_S1_1.Liver_IC_S1_drug_1*Liver_IC_S1_1+Liver_IC_S2_1.Liver_IC_S2_drug_1*Liver_IC_S2_1+Liver_IC_S3_1.Liver_IC_S3_drug_1*Liver_IC_S3_1+Liver_IC_S4_1.Liver_IC_S4_drug_1*Liver_IC_S4_1+Liver_IC_S5_1.Liver_IC_S5_drug_1*Liver_IC_S5_1+Liver_EC_S1_1.Liver_EC_S1_drug_1*Liver_EC_S1_1+Liver_EC_S2_1.Liver_EC_S2_drug_1*Liver_EC_S2_1+Liver_EC_S3_1.Liver_EC_S3_drug_1*Liver_EC_S3_1+Liver_EC_S4_1.Liver_EC_S4_drug_1*Liver_EC_S4_1+Liver_EC_S5_1.Liver_EC_S5_drug_1*Liver_EC_S5_1+Lung_1.Lung_drug_1*Lung_1+Adipose_1.Adipose_drug_1*Adipose_1+Heart_1.Heart_drug_1*Heart_1+Muscle_1.Muscle_drug_1*Muscle_1+Skin_1.Skin_drug_1*Skin_1+Kidney_1.Kidney_drug_1*Kidney_1+Bone_1.Bone_drug_1*Bone_1+Testes_1.Testes_drug_1*Testes_1+Rest_1.Rest_drug_1*Rest_1+Gut_1.Gut_drug_1*Gut_1+Spleen_1.Spleen_drug_1*Spleen_1+Brain_1.Brain_drug_1*Brain_1	0
70	Mass_Balance_1.Amount_total_1 = Venous_1.Venous_drug_1*Venous_1+Artery_1.Artery_drug_1*Artery_1+Liver_IC_S1_1.Liver_IC_S1_drug_1*Liver_IC_S1_1+Liver_IC_S2_1.Liver_IC_S2_drug_1*Liver_IC_S2_1+Liver_IC_S3_1.Liver_IC_S3_drug_1*Liver_IC_S3_1+Liver_IC_S4_1.Liver_IC_S4_drug_1*Liver_IC_S4_1+Liver_IC_S5_1.Liver_IC_S5_drug_1*Liver_IC_S5_1+Liver_EC_S1_1.Liver_EC_S1_drug_1*Liver_EC_S1_1+Liver_EC_S2_1.Liver_EC_S2_drug_1*Liver_EC_S2_1+Liver_EC_S3_1.Liver_EC_S3_drug_1*Liver_EC_S3_1+Liver_EC_S4_1.Liver_EC_S4_drug_1*Liver_EC_S4_1+Liver_EC_S5_1.Liver_EC_S5_drug_1*Liver_EC_S5_1+Lung_1.Lung_drug_1*Lu	0

	Repeated Assignments	Initial Value
	ng_1+Adipose_1.Adipose_drug_1*Adipose_1+Heart_1.Heart_drug_1*Heart_1+Muscle_1.Muscle_drug_1*Muscle_1+Skin_1.Skin_drug_1*Skin_1+Kidney_1.Kidney_drug_1*Kidney_1+Bone_1.Bone_drug_1*Bone_1+Testes_1.Testes_drug_1*Testes_1+Rest_1.Rest_drug_1*Rest_1+Gut_1.Gut_drug_1*Gut_1+Spleen_1.Spleen_drug_1*Spleen_1+Brain_1.Brain_drug_1*Brain_1+Main_compartment_1.Bile_drug_1+Metabolites_1.Metabolites_drug_1+Urine_1.Urine_drug_1+Gut_Lumen_1.Gut_Lumen_drug_1	
71	Plasma_total_1.Plasma_free_uM_1 = Plasma_total_1.Plasma_total_drug_1*drug_fuplasma_1/drug_molar_mass_1	0
72	Liver_total_1.Liver_blood_total_1 = (Liver_EC_S1_1.Liver_EC_S1_drug_1+Liver_EC_S2_1.Liver_EC_S2_drug_1+Liver_EC_S3_1.Liver_EC_S3_drug_1+Liver_EC_S4_1.Liver_EC_S4_drug_1+Liver_EC_S5_1.Liver_EC_S5_drug_1)/5	0
73	Liver_total_1.Liver_tissue_total_1 = (Liver_IC_S1_1.Liver_IC_S1_drug_1+Liver_IC_S2_1.Liver_IC_S2_drug_1+Liver_IC_S3_1.Liver_IC_S3_drug_1+Liver_IC_S4_1.Liver_IC_S4_drug_1+Liver_IC_S5_1.Liver_IC_S5_drug_1)/5	0
74	Liver_total_1.Liver_blood_free_1 = Liver_total_1.Liver_blood_total_1*drug_fuplasma_1/drug_BRP_1	0
75	Liver_total_1.Liver_tissue_free_uM_1 = Liver_total_1.Liver_tissue_total_1*drug_fuLiver_1/drug_molar_mass_1	0
76	convert_to_nmole_per_kg_1.Adipose_nmole_1 = Adipose_1.Adipose_drug_1*Adipose_1/drug_molar_mass_1*nanomole_per_mole*kilogram/phys_BW	0
77	convert_to_nmole_per_kg_1.Artery_nmole_1 = Artery_1.Artery_drug_1*Artery_1/drug_molar_mass_1*nanomole_per_mole*kilogram/phys_BW	0
78	convert_to_nmole_per_kg_1.Bone_nmole_1 = Bone_1.Bone_drug_1*Bone_1/drug_molar_mass_1*nanomole_per_mole*kilogram/phys_BW	0
79	convert_to_nmole_per_kg_1.Liver_EC1_nmole_1 = Liver_EC_S1_1.Liver_EC_S1_drug_1*Liver_EC_S1_1/drug_molar_mass_1*nanomole_per_mole*kilogram/phys_BW	0
80	convert_to_nmole_per_kg_1.Liver_IC1_nmole_1 = Liver_IC_S1_1.Liver_IC_S1_drug_1*Liver_IC_S1_1/drug_molar_mass_1*nanomole_per_mole*kilogram/phys_BW	0
81	convert_to_nmole_per_kg_1.Muscle_nmole_1 = Muscle_1.Muscle_drug_1*Muscle_1/drug_molar_mass_1*nanomole_per_mole*kilogram/phys_BW	0
82	convert_to_nmole_per_kg_1.Urine_nmole_1 = Urine_1.Urine_drug_1/drug_molar_mass_1*nanomole_per_mole*kilogram/phys_BW	0
83	convert_to_nmole_per_kg_1.Venous_nmole_1 = Venous_1.Venous_drug_1*Venous_1/drug_molar_mass_1*nanomole_per_mole*kilogram/phys_BW	0
84	convert_to_nmole_per_kg_1.Bile_nmole_1 = Main_compartment_1.Bile_drug_1/drug_molar_mass_1*nanomole_per_mole*kilogram/phys_BW	0

	Repeated Assignments	Initial Value
85	Plasma_total_1.Plasma_total_uM_1 = Plasma_total_1.Plasma_total_drug_1/drug_molar_mass_1	0
86	Liver_total_1.Liver_tissue_total_uM_1 = Liver_total_1.Liver_tissue_total_1/drug_molar_mass_1	0
87	convert_to_nmole_per_kg_1.Kidney_nmole_1 = Kidney_1.Kidney_drug_1*Kidney_1/drug_molar_mass_1*nanomole_per_mole*kilogram/phys_BW	0
88	convert_to_nmole_per_kg_1.Lung_nmole_1 = Lung_1.Lung_drug_1*Lung_1/drug_molar_mass_1*nanomole_per_mole*kilogram/phys_BW	0
89	convert_to_nmole_per_kg_1.Metabolites_nmole_1 = Metabolites_1.Metabolites_drug_1/drug_molar_mass_1*nanomole_per_mole*kilogram/phys_BW	0
90	convert_to_nmole_per_kg_1.Liver_IC2_nmole_1 = Liver_IC_S2_1.Liver_IC_S2_drug_1*Liver_IC_S2_1/drug_molar_mass_1*nanomole_per_mole*kilogram/phys_BW	0
91	convert_to_nmole_per_kg_1.Liver_EC2_nmole_1 = Liver_EC_S2_1.Liver_EC_S2_drug_1*Liver_EC_S2_1/drug_molar_mass_1*nanomole_per_mole*kilogram/phys_BW	0
92	convert_to_nmole_per_kg_1.Liver_EC3_nmole_1 = Liver_EC_S3_1.Liver_EC_S3_drug_1*Liver_EC_S3_1/drug_molar_mass_1*nanomole_per_mole*kilogram/phys_BW	0
93	convert_to_nmole_per_kg_1.Liver_IC3_nmole_1 = Liver_IC_S3_1.Liver_IC_S3_drug_1*Liver_IC_S3_1/drug_molar_mass_1*nanomole_per_mole*kilogram/phys_BW	0
94	convert_to_nmole_per_kg_1.Liver_IC4_nmole_1 = Liver_IC_S4_1.Liver_IC_S4_drug_1*Liver_IC_S4_1/drug_molar_mass_1*nanomole_per_mole*kilogram/phys_BW	0
95	convert_to_nmole_per_kg_1.Liver_EC4_nmole_1 = Liver_EC_S4_1.Liver_EC_S4_drug_1*Liver_EC_S4_1/drug_molar_mass_1*nanomole_per_mole*kilogram/phys_BW	0
96	convert_to_nmole_per_kg_1.Liver_EC5_nmole_1 = Liver_EC_S5_1.Liver_EC_S5_drug_1*Liver_EC_S5_1/drug_molar_mass_1*nanomole_per_mole*kilogram/phys_BW	0
97	convert_to_nmole_per_kg_1.Liver_IC5_nmole_1 = Liver_IC_S5_1.Liver_IC_S5_drug_1*Liver_IC_S5_1/drug_molar_mass_1*nanomole_per_mole*kilogram/phys_BW	0
98	convert_to_nmole_per_kg_1.Gut_nmole_1 = Gut_1.Gut_drug_1*Gut_1/drug_molar_mass_1*nanomole_per_mole*kilogram/phys_BW	0
99	convert_to_nmole_per_kg_1.Spleen_nmole_1 = Spleen_1.Spleen_drug_1*Spleen_1/drug_molar_mass_1*nanomole_per_mole*kilogram/phys_BW	0
100	convert_to_nmole_per_kg_1.Skin_nmole_1 = Skin_1.Skin_drug_1*Skin_1/drug_molar_mass_1*nanomole_per_mole*kilogram/phys_BW	0
101	convert_to_nmole_per_kg_1.Brain_nmole_1 = Brain_1.Brain_drug_1*Brain_1/drug_molar_mass_1*nanomole_per_mole*kilogram/phys_BW	0
102	convert_to_nmole_per_kg_1.Rest_nmole_1 = Rest_1.Rest_drug_1*Rest_1/drug_molar_mass_1*nanomole_per_mole*kilogram/phys_BW	0
103	convert_to_nmole_per_kg_1.Heart_nmole_1 = Heart_1.Heart_drug_1*Heart_1/drug_molar_mass_1*nano	0

	Repeated Assignments	Initial Value
	mole_per_mole*kilogram/phys_BW	
104	uptake_inhib_S1 = (1-switch_uptake_inhib_1)+switch_uptake_inhib_1/(1+drug_fB*Liver_EC_S1.Liver_EC_S1_drug/(drug_uptake_Ki*drug_molar_mass))	1
105	uptake_inhib_S3 = (1-switch_uptake_inhib_1)+switch_uptake_inhib_1/(1+drug_fB*Liver_EC_S3.Liver_EC_S3_drug/(drug_uptake_Ki*drug_molar_mass))	1
106	uptake_inhib_S2 = (1-switch_uptake_inhib_1)+switch_uptake_inhib_1/(1+drug_fB*Liver_EC_S2.Liver_EC_S2_drug/(drug_uptake_Ki*drug_molar_mass))	1
107	uptake_inhib_S4 = (1-switch_uptake_inhib_1)+switch_uptake_inhib_1/(1+drug_fB*Liver_EC_S4.Liver_EC_S4_drug/(drug_uptake_Ki*drug_molar_mass))	1
108	uptake_inhib_S5 = (1-switch_uptake_inhib_1)+switch_uptake_inhib_1/(1+drug_fB*Liver_EC_S5.Liver_EC_S5_drug/(drug_uptake_Ki*drug_molar_mass))	1
109	biliary_inhib_S1 = (1-switch_biliary_inhib_1)+switch_biliary_inhib_1/(1+drug_fuLiver*Liver_IC_S1.Liver_IC_S1_drug/(drug_biliary_Ki*drug_molar_mass))	1
110	biliary_inhib_S2 = (1-switch_biliary_inhib_1)+switch_biliary_inhib_1/(1+drug_fuLiver*Liver_IC_S2.Liver_IC_S2_drug/(drug_biliary_Ki*drug_molar_mass))	1
111	biliary_inhib_S3 = (1-switch_biliary_inhib_1)+switch_biliary_inhib_1/(1+drug_fuLiver*Liver_IC_S3.Liver_IC_S3_drug/(drug_biliary_Ki*drug_molar_mass))	1
112	biliary_inhib_S4 = (1-switch_biliary_inhib_1)+switch_biliary_inhib_1/(1+drug_fuLiver*Liver_IC_S4.Liver_IC_S4_drug/(drug_biliary_Ki*drug_molar_mass))	1
113	biliary_inhib_S5 = (1-switch_biliary_inhib_1)+switch_biliary_inhib_1/(1+drug_fuLiver*Liver_IC_S5.Liver_IC_S5_drug/(drug_biliary_Ki*drug_molar_mass))	1
114	met_inhib_S1 = (1-switch_met_inhib_1)+switch_met_inhib_1/(1+drug_fuLiver*Liver_IC_S1.Liver_IC_S1_drug/(drug_met_Ki*drug_molar_mass))	1
115	met_inhib_S2 = (1-switch_met_inhib_1)+switch_met_inhib_1/(1+drug_fuLiver*Liver_IC_S2.Liver_IC_S2_drug/(drug_met_Ki*drug_molar_mass))	1
116	met_inhib_S3 = (1-switch_met_inhib_1)+switch_met_inhib_1/(1+drug_fuLiver*Liver_IC_S3.Liver_IC_S3_drug/(drug_met_Ki*drug_molar_mass))	1
117	met_inhib_S4 = (1-switch_met_inhib_1)+switch_met_inhib_1/(1+drug_fuLiver*Liver_IC_S4.Liver_IC_S4_drug/(drug_met_Ki*drug_molar_mass))	1

	Repeated Assignments	Initial Value
118	met_inhib_S5 = (1-switch_met_inhib_1)+switch_met_inhib_1/(1+drug_fuLiver*Liver_IC_S5.Liver_IC_S5_drug/(drug_met_Ki*drug_molar_mass))	1
119	Kpuu_Liver_1 = Liver_total_1.Liver_tissue_free_uM_1/Plasma_total_1.Plasma_free_uM_1	NaN
120	convert_to_nmole_per_kg.X_CECUM_DISS_nmole = Colon.X_CECUM_DISS/drug_molar_mass*nanomole_per_mole*kilogram/phys_BW	0
121	efflux_inhib_duo = (1-switch_efflux_inhib_1)+switch_efflux_inhib_1/(1+fu_mem*MDUO.MEM_DUO/VDUO/(drug_efflux_Ki*drug_molar_mass))	1
122	efflux_inhib_jej1 = (1-switch_efflux_inhib_1)+switch_efflux_inhib_1/(1+fu_mem*MJEJ1.MEM_JEJ1/VJEJ1/(drug_efflux_Ki*drug_molar_mass))	1
123	efflux_inhib_jej2 = (1-switch_efflux_inhib_1)+switch_efflux_inhib_1/(1+fu_mem*MJEJ2.MEM_JEJ2/VJEJ2/(drug_efflux_Ki*drug_molar_mass))	1
124	efflux_inhib_ill1 = (1-switch_efflux_inhib_1)+switch_efflux_inhib_1/(1+fu_mem*MILL1.MEM_ILL1/VILL1/(drug_efflux_Ki*drug_molar_mass))	1
125	efflux_inhib_ill2 = (1-switch_efflux_inhib_1)+switch_efflux_inhib_1/(1+fu_mem*MILL2.MEM_ILL2/VILL2/(drug_efflux_Ki*drug_molar_mass))	1
126	efflux_inhib_ill3 = (1-switch_efflux_inhib_1)+switch_efflux_inhib_1/(1+fu_mem*MILL3.MEM_ILL3/VILL3/(drug_efflux_Ki*drug_molar_mass))	1
127	efflux_inhib_ill4 = (1-switch_efflux_inhib_1)+switch_efflux_inhib_1/(1+fu_mem*MILL4.MEM_ILL4/VILL4/(drug_efflux_Ki*drug_molar_mass))	1

Reactions

	Reactions
1	Liver_IC_S5.Liver_IC_S5_drug -> Main_compartment.Bile_drug
	*(1-switch_liverFlag)*k_Liver_IC_S5_Bile*Liver_IC_S5.Liver_IC_S5_drug*
2	Liver_IC_S5.Liver_IC_S5_drug -> Metabolites.Metabolites_drug
	*(1-switch_liverFlag)*k_Liver_IC_S5_Metabolites*Liver_IC_S5.Liver_IC_S5_drug*
3	Liver_IC_S4.Liver_IC_S4_drug -> Metabolites.Metabolites_drug
	*(1-switch_liverFlag)*k_Liver_IC_S4_Metabolites*Liver_IC_S4.Liver_IC_S4_drug*
4	Liver_IC_S3.Liver_IC_S3_drug -> Metabolites.Metabolites_drug
	*(1-switch_liverFlag)*k_Liver_IC_S3_Metabolites*Liver_IC_S3.Liver_IC_S3_drug*
5	Liver_IC_S2.Liver_IC_S2_drug -> Metabolites.Metabolites_drug
	*(1-switch_liverFlag)*k_Liver_IC_S2_Metabolites*Liver_IC_S2.Liver_IC_S2_drug*
6	Liver_IC_S1.Liver_IC_S1_drug -> Metabolites.Metabolites_drug

	Reactions
	*(1-switch_liverFlag)*k_Liver_IC_S1_Metabolites*Liver_IC_S1.Liver_IC_S1_drug*
7	Liver_IC_S4.Liver_IC_S4_drug -> Main_compartment.Bile_drug
	*(1-switch_liverFlag)*k_Liver_IC_S4_Bile*Liver_IC_S4.Liver_IC_S4_drug*
8	Liver_IC_S3.Liver_IC_S3_drug -> Main_compartment.Bile_drug
	*(1-switch_liverFlag)*k_Liver_IC_S3_Bile*Liver_IC_S3.Liver_IC_S3_drug*
9	Liver_IC_S2.Liver_IC_S2_drug -> Main_compartment.Bile_drug
	*(1-switch_liverFlag)*k_Liver_IC_S2_Bile*Liver_IC_S2.Liver_IC_S2_drug*
10	Liver_IC_S1.Liver_IC_S1_drug -> Main_compartment.Bile_drug
	*(1-switch_liverFlag)*k_Liver_IC_S1_Bile*Liver_IC_S1.Liver_IC_S1_drug*
11	Liver_IC_S5.Liver_IC_S5_drug -> Liver_EC_S5.Liver_EC_S5_drug
	*k_Liver_IC_S5_Liver_EC_S5*Liver_IC_S5.Liver_IC_S5_drug*
12	Liver_EC_S5.Liver_EC_S5_drug -> Liver_IC_S5.Liver_IC_S5_drug
	*k_Liver_EC_S5_Liver_IC_S5*Liver_EC_S5.Liver_EC_S5_drug*
13	Liver_IC_S4.Liver_IC_S4_drug -> Liver_EC_S4.Liver_EC_S4_drug
	*k_Liver_IC_S4_Liver_EC_S4*Liver_IC_S4.Liver_IC_S4_drug*
14	Liver_EC_S4.Liver_EC_S4_drug -> Liver_IC_S4.Liver_IC_S4_drug
	*k_Liver_EC_S4_Liver_IC_S4*Liver_EC_S4.Liver_EC_S4_drug*
15	Liver_IC_S3.Liver_IC_S3_drug -> Liver_EC_S3.Liver_EC_S3_drug
	*k_Liver_IC_S3_Liver__EC_S3*Liver_IC_S3.Liver_IC_S3_drug*
16	Liver_EC_S3.Liver_EC_S3_drug -> Liver_IC_S3.Liver_IC_S3_drug
	*k_Liver_EC_S3_Liver_IC_S3*Liver_EC_S3.Liver_EC_S3_drug*
17	Liver_IC_S2.Liver_IC_S2_drug -> Liver_EC_S2.Liver_EC_S2_drug
	*k_Liver_IC_S2_Liver_EC_S2*Liver_IC_S2.Liver_IC_S2_drug*
18	Liver_EC_S2.Liver_EC_S2_drug -> Liver_IC_S2.Liver_IC_S2_drug
	*k_Liver_EC_S2_Liver_IC_S2*Liver_EC_S2.Liver_EC_S2_drug*
19	Liver_IC_S1.Liver_IC_S1_drug -> Liver_EC_S1.Liver_EC_S1_drug
	*k_Liver_IC_S1_Liver_EC_S1*Liver_IC_S1.Liver_IC_S1_drug*
20	Liver_EC_S1.Liver_EC_S1_drug -> Liver_IC_S1.Liver_IC_S1_drug
	*k_Liver_EC_S1_Liver_IC_S1*Liver_EC_S1.Liver_EC_S1_drug*
21	Liver.Liver_drug -> Venous.Venous_drug
	*switch_liverFlag*k_Liver_Venous*Liver.Liver_drug*
22	Liver_EC_S4.Liver_EC_S4_drug -> Liver_EC_S5.Liver_EC_S5_drug
	*k_Liver_EC_S4_Liver_EC_S5*Liver_EC_S4.Liver_EC_S4_drug*
23	Liver_EC_S3.Liver_EC_S3_drug -> Liver_EC_S4.Liver_EC_S4_drug
	*k_Liver_EC_S3_Liver_EC_S4*Liver_EC_S3.Liver_EC_S3_drug*
24	Liver_EC_S2.Liver_EC_S2_drug -> Liver_EC_S3.Liver_EC_S3_drug
	*k_Liver_EC_S2_Liver_EC_S3*Liver_EC_S2.Liver_EC_S2_drug*
25	Liver_EC_S1.Liver_EC_S1_drug -> Liver_EC_S2.Liver_EC_S2_drug
	*k_Liver_EC_S1_Liver_EC_S2*Liver_EC_S1.Liver_EC_S1_drug*
26	Artery.Artery_drug -> Liver.Liver_drug
	*switch_liverFlag*k_artery_liver*Artery.Artery_drug*

	**Reactions**
27	Artery.Artery_drug -> VillousILL1.Villous_ILL1
	*Qmuc_ILL1*Artery.Artery_drug*
28	Artery.Artery_drug -> Spleen.Spleen_drug
	*k_artery_spleen*Artery.Artery_drug*
29	Rest.Rest_drug -> Venous.Venous_drug
	*k_rest_venous*Rest.Rest_drug*
30	Bone.Bone_drug -> Venous.Venous_drug
	*k_bone_venous*Bone.Bone_drug*
31	Skin.Skin_drug -> Venous.Venous_drug
	*k_skin_venous*Skin.Skin_drug*
32	Heart.Heart_drug -> Venous.Venous_drug
	*k_heart_venous*Heart.Heart_drug*
33	Adipose.Adipose_drug -> Venous.Venous_drug
	*k_adipos_venous*Adipose.Adipose_drug*
34	Muscle.Muscle_drug -> Venous.Venous_drug
	*k_muscle_venous*Muscle.Muscle_drug*
35	Brain.Brain_drug -> Venous.Venous_drug
	*k_brain_venous*Brain.Brain_drug*
36	Kidney.Kidney_drug -> Venous.Venous_drug
	*k_kidney_venous*Kidney.Kidney_drug*
37	Artery.Artery_drug -> Rest.Rest_drug
	*k_artery_rest*Artery.Artery_drug*
38	Artery.Artery_drug -> Bone.Bone_drug
	*k_artery_bone*Artery.Artery_drug*
39	Artery.Artery_drug -> Skin.Skin_drug
	*k_artery_skin*Artery.Artery_drug*
40	Artery.Artery_drug -> Heart.Heart_drug
	*k_artery_heart*Artery.Artery_drug*
41	Artery.Artery_drug -> Adipose.Adipose_drug
	*k_artery_adipos*Artery.Artery_drug*
42	Artery.Artery_drug -> Muscle.Muscle_drug
	*k_artery_muscle*Artery.Artery_drug*
43	Artery.Artery_drug -> Brain.Brain_drug
	*k_artery_brain*Artery.Artery_drug*
44	Artery.Artery_drug -> Kidney.Kidney_drug
	*k_artery_kidney*Artery.Artery_drug*
45	Venous.Venous_drug -> Lung.Lung_drug
	*k_venous_lung*Venous.Venous_drug*
46	Lung.Lung_drug -> Artery.Artery_drug
	*k_lung_artery*Lung.Lung_drug*
47	Venous.Venous_drug -> Urine.Urine_drug

	Reactions
	*k_venous_urine_CLR*Venous.Venous_drug*
48	Artery.Artery_drug -> Testes.Testes_drug
	*k_artery_testes*Artery.Artery_drug*
49	Testes.Testes_drug -> Venous.Venous_drug
	*k_testes_venous*Testes.Testes_drug*
50	Liver_IC_S4.Liver_IC_S4_drug -> Liver_EC_S4.Liver_EC_S4_drug
	*k_Liver_IC_S4_Liver_EC_S4_efflux*Liver_IC_S4.Liver_IC_S4_drug*
51	Liver_IC_S3.Liver_IC_S3_drug -> Liver_EC_S3.Liver_EC_S3_drug
	*k_Liver_IC_S3_Liver_EC_S3_efflux*Liver_IC_S3.Liver_IC_S3_drug*
52	Liver_IC_S2.Liver_IC_S2_drug -> Liver_EC_S2.Liver_EC_S2_drug
	*k_Liver_IC_S2_Liver_EC_S2_efflux*Liver_IC_S2.Liver_IC_S2_drug*
53	Liver_IC_S5.Liver_IC_S5_drug -> Liver_EC_S5.Liver_EC_S5_drug
	*k_Liver_IC_S5_Liver_EC_S5_efflux*Liver_IC_S5.Liver_IC_S5_drug*
54	Liver_IC_S1.Liver_IC_S1_drug -> Liver_EC_S1.Liver_EC_S1_drug
	*k_Liver_IC_S1_Liver_EC_S1_efflux*Liver_IC_S1.Liver_IC_S1_drug*
55	Venous.Venous_drug -> Urine.Urine_drug
	*k_venous_urine_GFR*Venous.Venous_drug*
56	Liver_IC_S5_1.Liver_IC_S5_drug_1 -> Main_compartment_1.Bile_drug_1
	*(1-switch_liverFlag_1)*k_Liver_IC_S5_Bile_1*Liver_IC_S5_1.Liver_IC_S5_drug_1*
57	Liver_IC_S5_1.Liver_IC_S5_drug_1 -> Metabolites_1.Metabolites_drug_1
	*(1-switch_liverFlag_1)*k_Liver_IC_S5_Metabolites_1*Liver_IC_S5_1.Liver_IC_S5_drug_1*
58	Liver_IC_S4_1.Liver_IC_S4_drug_1 -> Metabolites_1.Metabolites_drug_1
	*(1-switch_liverFlag_1)*k_Liver_IC_S4_Metabolites_1*Liver_IC_S4_1.Liver_IC_S4_drug_1*
59	Liver_IC_S3_1.Liver_IC_S3_drug_1 -> Metabolites_1.Metabolites_drug_1
	*(1-switch_liverFlag_1)*k_Liver_IC_S3_Metabolites_1*Liver_IC_S3_1.Liver_IC_S3_drug_1*
60	Liver_IC_S2_1.Liver_IC_S2_drug_1 -> Metabolites_1.Metabolites_drug_1
	*(1-switch_liverFlag_1)*k_Liver_IC_S2_Metabolites_1*Liver_IC_S2_1.Liver_IC_S2_drug_1*
61	Liver_IC_S1_1.Liver_IC_S1_drug_1 -> Metabolites_1.Metabolites_drug_1
	*(1-switch_liverFlag_1)*k_Liver_IC_S1_Metabolites_1*Liver_IC_S1_1.Liver_IC_S1_drug_1*
62	Liver_IC_S4_1.Liver_IC_S4_drug_1 -> Main_compartment_1.Bile_drug_1
	*(1-switch_liverFlag_1)*k_Liver_IC_S4_Bile_1*Liver_IC_S4_1.Liver_IC_S4_drug_1*
63	Liver_IC_S3_1.Liver_IC_S3_drug_1 -> Main_compartment_1.Bile_drug_1
	*(1-switch_liverFlag_1)*k_Liver_IC_S3_Bile_1*Liver_IC_S3_1.Liver_IC_S3_drug_1*
64	Liver_IC_S2_1.Liver_IC_S2_drug_1 -> Main_compartment_1.Bile_drug_1
	*(1-switch_liverFlag_1)*k_Liver_IC_S2_Bile_1*Liver_IC_S2_1.Liver_IC_S2_drug_1*
65	Liver_IC_S1_1.Liver_IC_S1_drug_1 -> Main_compartment_1.Bile_drug_1
	*(1-switch_liverFlag_1)*k_Liver_IC_S1_Bile_1*Liver_IC_S1_1.Liver_IC_S1_drug_1*
66	Liver_IC_S5_1.Liver_IC_S5_drug_1 -> Liver_EC_S5_1.Liver_EC_S5_drug_1
	*k_Liver_IC_S5_Liver_EC_S5_1*Liver_IC_S5_1.Liver_IC_S5_drug_1*
67	Liver_EC_S5_1.Liver_EC_S5_drug_1 -> Liver_IC_S5_1.Liver_IC_S5_drug_1
	*k_Liver_EC_S5_Liver_IC_S5_1*Liver_EC_S5_1.Liver_EC_S5_drug_1*

	Reactions
68	Liver_IC_S4_1.Liver_IC_S4_drug_1 -> Liver_EC_S4_1.Liver_EC_S4_drug_1
	*k_Liver_IC_S4_Liver_EC_S4_1*Liver_IC_S4_1.Liver_IC_S4_drug_1*
69	Liver_EC_S4_1.Liver_EC_S4_drug_1 -> Liver_IC_S4_1.Liver_IC_S4_drug_1
	*k_Liver_EC_S4_Liver_IC_S4_1*Liver_EC_S4_1.Liver_EC_S4_drug_1*
70	Liver_IC_S3_1.Liver_IC_S3_drug_1 -> Liver_EC_S3_1.Liver_EC_S3_drug_1
	*k_Liver_IC_S3_Liver__EC_S3_1*Liver_IC_S3_1.Liver_IC_S3_drug_1*
71	Liver_EC_S3_1.Liver_EC_S3_drug_1 -> Liver_IC_S3_1.Liver_IC_S3_drug_1
	*k_Liver_EC_S3_Liver_IC_S3_1*Liver_EC_S3_1.Liver_EC_S3_drug_1*
72	Liver_IC_S2_1.Liver_IC_S2_drug_1 -> Liver_EC_S2_1.Liver_EC_S2_drug_1
	*k_Liver_IC_S2_Liver_EC_S2_1*Liver_IC_S2_1.Liver_IC_S2_drug_1*
73	Liver_EC_S2_1.Liver_EC_S2_drug_1 -> Liver_IC_S2_1.Liver_IC_S2_drug_1
	*k_Liver_EC_S2_Liver_IC_S2_1*Liver_EC_S2_1.Liver_EC_S2_drug_1*
74	Liver_IC_S1_1.Liver_IC_S1_drug_1 -> Liver_EC_S1_1.Liver_EC_S1_drug_1
	*k_Liver_IC_S1_Liver_EC_S1_1*Liver_IC_S1_1.Liver_IC_S1_drug_1*
75	Liver_EC_S1_1.Liver_EC_S1_drug_1 -> Liver_IC_S1_1.Liver_IC_S1_drug_1
	*k_Liver_EC_S1_Liver_IC_S1_1*Liver_EC_S1_1.Liver_EC_S1_drug_1*
76	Liver_EC_S5_1.Liver_EC_S5_drug_1 -> Venous_1.Venous_drug_1
	*(1-switch_liverFlag_1)*k_Liver_EC_S5_Venous_1*Liver_EC_S5_1.Liver_EC_S5_drug_1*
77	Liver_EC_S4_1.Liver_EC_S4_drug_1 -> Liver_EC_S5_1.Liver_EC_S5_drug_1
	*k_Liver_EC_S4_Liver_EC_S5_1*Liver_EC_S4_1.Liver_EC_S4_drug_1*
78	Liver_EC_S3_1.Liver_EC_S3_drug_1 -> Liver_EC_S4_1.Liver_EC_S4_drug_1
	*k_Liver_EC_S3_Liver_EC_S4_1*Liver_EC_S3_1.Liver_EC_S3_drug_1*
79	Liver_EC_S2_1.Liver_EC_S2_drug_1 -> Liver_EC_S3_1.Liver_EC_S3_drug_1
	*k_Liver_EC_S2_Liver_EC_S3_1*Liver_EC_S2_1.Liver_EC_S2_drug_1*
80	Liver_EC_S1_1.Liver_EC_S1_drug_1 -> Liver_EC_S2_1.Liver_EC_S2_drug_1
	*k_Liver_EC_S1_Liver_EC_S2_1*Liver_EC_S1_1.Liver_EC_S1_drug_1*
81	Gut_1.Gut_drug_1 -> Liver_EC_S1_1.Liver_EC_S1_drug_1
	*k_gut_liver_1*Gut_1.Gut_drug_1*
82	Spleen_1.Spleen_drug_1 -> Liver_EC_S1_1.Liver_EC_S1_drug_1
	*(1-switch_liverFlag_1)*k_spleen_liver_1*Spleen_1.Spleen_drug_1*
83	Artery_1.Artery_drug_1 -> Liver_EC_S1_1.Liver_EC_S1_drug_1
	*(1-switch_liverFlag_1)*k_artery_liver_1*Artery_1.Artery_drug_1*
84	Artery_1.Artery_drug_1 -> Gut_1.Gut_drug_1
	*k_artery_gut_1*Artery_1.Artery_drug_1*
85	Artery_1.Artery_drug_1 -> Spleen_1.Spleen_drug_1
	*k_artery_spleen_1*Artery_1.Artery_drug_1*
86	Rest_1.Rest_drug_1 -> Venous_1.Venous_drug_1
	*k_rest_venous_1*Rest_1.Rest_drug_1*
87	Bone_1.Bone_drug_1 -> Venous_1.Venous_drug_1
	*k_bone_venous_1*Bone_1.Bone_drug_1*
88	Skin_1.Skin_drug_1 -> Venous_1.Venous_drug_1

	Reactions
	*k_skin_venous_1*Skin_1.Skin_drug_1*
89	Heart_1.Heart_drug_1 -> Venous_1.Venous_drug_1
	*k_heart_venous_1*Heart_1.Heart_drug_1*
90	Adipose_1.Adipose_drug_1 -> Venous_1.Venous_drug_1
	*k_adipos_venous_1*Adipose_1.Adipose_drug_1*
91	Muscle_1.Muscle_drug_1 -> Venous_1.Venous_drug_1
	*k_muscle_venous_1*Muscle_1.Muscle_drug_1*
92	Brain_1.Brain_drug_1 -> Venous_1.Venous_drug_1
	*k_brain_venous_1*Brain_1.Brain_drug_1*
93	Kidney_1.Kidney_drug_1 -> Venous_1.Venous_drug_1
	*k_kidney_venous_1*Kidney_1.Kidney_drug_1*
94	Artery_1.Artery_drug_1 -> Rest_1.Rest_drug_1
	*k_artery_rest_1*Artery_1.Artery_drug_1*
95	Artery_1.Artery_drug_1 -> Bone_1.Bone_drug_1
	*k_artery_bone_1*Artery_1.Artery_drug_1*
96	Artery_1.Artery_drug_1 -> Skin_1.Skin_drug_1
	*k_artery_skin_1*Artery_1.Artery_drug_1*
97	Artery_1.Artery_drug_1 -> Heart_1.Heart_drug_1
	*k_artery_heart_1*Artery_1.Artery_drug_1*
98	Artery_1.Artery_drug_1 -> Adipose_1.Adipose_drug_1
	*k_artery_adipos_1*Artery_1.Artery_drug_1*
99	Artery_1.Artery_drug_1 -> Muscle_1.Muscle_drug_1
	*k_artery_muscle_1*Artery_1.Artery_drug_1*
100	Artery_1.Artery_drug_1 -> Brain_1.Brain_drug_1
	*k_artery_brain_1*Artery_1.Artery_drug_1*
101	Artery_1.Artery_drug_1 -> Kidney_1.Kidney_drug_1
	*k_artery_kidney_1*Artery_1.Artery_drug_1*
102	Venous_1.Venous_drug_1 -> Lung_1.Lung_drug_1
	*k_venous_lung_1*Venous_1.Venous_drug_1*
103	Lung_1.Lung_drug_1 -> Artery_1.Artery_drug_1
	*k_lung_artery_1*Lung_1.Lung_drug_1*
104	Venous_1.Venous_drug_1 -> Urine_1.Urine_drug_1
	*k_venous_urine_CLR_1*Venous_1.Venous_drug_1*
105	Artery_1.Artery_drug_1 -> Testes_1.Testes_drug_1
	*k_artery_testes_1*Artery_1.Artery_drug_1*
106	Testes_1.Testes_drug_1 -> Venous_1.Venous_drug_1
	*k_testes_venous_1*Testes_1.Testes_drug_1*
107	Venous_1.Venous_drug_1 -> Urine_1.Urine_drug_1
	*k_venous_urine_GFR_1*Venous_1.Venous_drug_1*
108	Liver_IC_S5_1.Liver_IC_S5_drug_1 -> Liver_EC_S5_1.Liver_EC_S5_drug_1
	*k_Liver_IC_S5_Liver_EC_S5_efflux_1*Liver_IC_S5_1.Liver_IC_S5_drug_1*

	**Reactions**
109	Liver_IC_S4_1.Liver_IC_S4_drug_1 -> Liver_EC_S4_1.Liver_EC_S4_drug_1
	*k_Liver_IC_S4_Liver_EC_S4_efflux_1*Liver_IC_S4_1.Liver_IC_S4_drug_1*
110	Liver_IC_S3_1.Liver_IC_S3_drug_1 -> Liver_EC_S3_1.Liver_EC_S3_drug_1
	*k_Liver_IC_S3_Liver_EC_S3_efflux_1*Liver_IC_S3_1.Liver_IC_S3_drug_1*
111	Liver_IC_S2_1.Liver_IC_S2_drug_1 -> Liver_EC_S2_1.Liver_EC_S2_drug_1
	*k_Liver_IC_S2_Liver_EC_S2_efflux_1*Liver_IC_S2_1.Liver_IC_S2_drug_1*
112	Liver_IC_S1_1.Liver_IC_S1_drug_1 -> Liver_EC_S1_1.Liver_EC_S1_drug_1
	*k_Liver_IC_S1_Liver_EC_S1_efflux_1*Liver_IC_S1_1.Liver_IC_S1_drug_1*
113	Main_compartment.Bile_drug -> VDUO.X_DUO_DISS
	*k_transit*Main_compartment.Bile_drug*
114	Gut_Lumen.Gut_Lumen_drug -> Gut.Gut_drug
	*drug_k_oral*drug_fa*Gut_Lumen.Gut_Lumen_drug*
115	Main_compartment.Bile_drug -> null
	*drug_k_bile_deg*Main_compartment.Bile_drug*
116	Gut_Lumen.Gut_Lumen_drug -> null
	drug_k_oral(1-drug_fa)*Gut_Lumen.Gut_Lumen_drug*
117	Gut.Gut_drug -> null
	*k_gut_liver*Gut.Gut_drug*
118	Spleen.Spleen_drug -> Liver.Liver_drug
	*switch_liverFlag*k_spleen_liver*Spleen.Spleen_drug*
119	Gut_Lumen_1.Gut_Lumen_drug_1 -> Gut_1.Gut_drug_1
	*drug_k_oral_1*drug_fa_1*Gut_Lumen_1.Gut_Lumen_drug_1*
120	Main_compartment_1.Bile_drug_1 -> Gut_Lumen_1.Gut_Lumen_drug_1
	*k_transit_1*Main_compartment_1.Bile_drug_1*
121	Main_compartment_1.Bile_drug_1 -> null
	*drug_k_bile_deg_1*Main_compartment_1.Bile_drug_1*
122	Gut_Lumen_1.Gut_Lumen_drug_1 -> null
	drug_k_oral_1(1-drug_fa_1)*Gut_Lumen_1.Gut_Lumen_drug_1*
123	STOMACH.X_STOMACH_SOLID -> VDUO.X_DUO_SOLID
	STOMACH.X_STOMACH_SOLID/TSTOMACH
124	STOMACH.X_STOMACH_DISS -> VDUO.X_DUO_DISS
	STOMACH.X_STOMACH_DISS/TSTOMACH
125	VDUO.X_DUO_SOLID -> VJEJ1.X_JEJ1_SOLID
	VDUO.X_DUO_SOLID/TDUO
126	VDUO.X_DUO_SOLID -> VDUO.X_DUO_DISS
	*KD*VDUO.X_DUO_SOLID*(SOLIF_DUO-VDUO.X_DUO_DISS/VDUO)*
127	VDUO.X_DUO_DISS -> VJEJ1.X_JEJ1_DISS
	VDUO.X_DUO_DISS/TDUO
128	VDUO.X_DUO_DISS -> MDUO.MEM_DUO
	*(DIFF_duo*NI_DUO*switch_SFdiffapi*VDUO.X_DUO_DISS)/VDUO*
129	MDUO.MEM_DUO -> VDUO.X_DUO_DISS

	Reactions
	((switchVmax_efflux==zero)*CLINT_efflux_DUO*efflux_factor_duo*switch_SFefflux+switchVmax_efflux*phys_Normalized_ESA*phys_BW*surfaceRatio_DUO*efflux_factor_duo*switch_SFefflux/(drug_Km_efflux+MDUO.MEM_DUO*fu_mem/MDUO/drug_molar_mass))*MDUO.MEM_DUO*fu_mem/MDUO
130	VDUO.X_DUO_DISS -> MDUO.MEM_DUO
	((switchVmax_influx==zero)*CLINT_influx_DUO*influx_factor_duo*switch_SFinflux+switchVmax_influx*phys_Normalized_ESA*phys_BW*surfaceRatio_DUO*influx_factor_duo/(drug_Km_influx+VDUO.X_DUO_DISS/VDUO/drug_molar_mass))*VDUO.X_DUO_DISS/VDUO
131	VillousDUO.Villous_DUO -> Liver.Liver_drug
	switch_liverFlag*VillousDUO.Villous_DUO*Qmuc_DUO/VillousDUO
132	MDUO.MEM_DUO -> null
	(CLINT_metabolism*metabolism_factor_duo*switch_SFgutmet*MDUO.MEM_DUO*fu_mem)/MDUO
133	VJEJ1.X_JEJ1_SOLID -> VJEJ2.X_JEJ2_SOLID
	VJEJ1.X_JEJ1_SOLID/TJEJ1
134	VJEJ1.X_JEJ1_SOLID -> VJEJ1.X_JEJ1_DISS
	KD*VJEJ1.X_JEJ1_SOLID*(SOLIF_JEJ1-VJEJ1.X_JEJ1_DISS/VJEJ1)
135	VJEJ1.X_JEJ1_DISS -> VJEJ2.X_JEJ2_DISS
	VJEJ1.X_JEJ1_DISS/TJEJ1
136	VJEJ1.X_JEJ1_DISS -> MJEJ1.MEM_JEJ1
	(DIFF_jej1*NI_JEJ1*switch_SFdiffapi*VJEJ1.X_JEJ1_DISS)/VJEJ1
137	MJEJ1.MEM_JEJ1 -> VJEJ1.X_JEJ1_DISS
	((switchVmax_efflux==zero)*CLINT_efflux_JEJ1*efflux_factor_jej1*switch_SFefflux+switchVmax_efflux*phys_Normalized_ESA*phys_BW*surfaceRatio_JEJ1*efflux_factor_jej1*switch_SFefflux/(drug_Km_efflux+MJEJ1.MEM_JEJ1*fu_mem/MJEJ1/drug_molar_mass))*MJEJ1.MEM_JEJ1*fu_mem/MJEJ1
138	VJEJ1.X_JEJ1_DISS -> MJEJ1.MEM_JEJ1
	((switchVmax_influx==zero)*CLINT_influx_JEJ1*influx_factor_jej1*switch_SFinflux+switchVmax_influx*phys_Normalized_ESA*phys_BW*surfaceRatio_JEJ1*influx_factor_jej1/(drug_Km_influx+VJEJ1.X_JEJ1_DISS/VJEJ1/drug_molar_mass))*VJEJ1.X_JEJ1_DISS/VJEJ1
139	VillousJEJ1.Villous_JEJ1 -> Liver.Liver_drug
	switch_liverFlag*VillousJEJ1.Villous_JEJ1*Qmuc_JEJ1/VillousJEJ1
140	MJEJ1.MEM_JEJ1 -> null
	(CLINT_metabolism*metabolism_factor_jej1*switch_SFgutmet*MJEJ1.MEM_JEJ1*fu_mem)/MJEJ1
141	VJEJ2.X_JEJ2_SOLID -> VILL1.X_ILL1_SOLID
	VJEJ2.X_JEJ2_SOLID/TJEJ2
142	VJEJ2.X_JEJ2_SOLID -> VJEJ2.X_JEJ2_DISS
	KD*VJEJ2.X_JEJ2_SOLID*(SOLIF_JEJ2-VJEJ2.X_JEJ2_DISS/VJEJ2)
143	VJEJ2.X_JEJ2_DISS -> VILL1.X_ILL1_DISS
	VJEJ2.X_JEJ2_DISS/TJEJ2
144	VJEJ2.X_JEJ2_DISS -> MJEJ2.MEM_JEJ2
	(DIFF_jej2*NI_JEJ2*switch_SFdiffapi*VJEJ2.X_JEJ2_DISS)/VJEJ2
145	MJEJ2.MEM_JEJ2 -> VJEJ2.X_JEJ2_DISS
	((switchVmax_efflux==zero)*CLINT_efflux_JEJ2*efflux_factor_jej2*switch_SFefflux+switchVmax_efflux*phys_Normalized_ESA*phys_BW*surfaceRatio_JEJ2*efflux_factor_jej2*switch_SFefflux/(drug_Km_efflux+MJEJ2.MEM_JEJ2*fu_mem/MJEJ2/drug_molar_mass))*MJEJ2.MEM_JEJ2*fu_mem/MJEJ2
146	VJEJ2.X_JEJ2_DISS -> MJEJ2.MEM_JEJ2

	Reactions
	*((switchVmax_influx==zero)*CLINT_influx_JEJ2*influx_factor_jej2*switch_SFinflux+switchVmax_influx*phys_Normalized_ESA*phys_BW*surfaceRatio_JEJ2*influx_factor_jej2/(drug_Km_influx+VJEJ2.X_JEJ2_DISS/VJEJ2/drug_molar_mass))*VJEJ2.X_JEJ2_DISS/VJEJ2*
147	VillousJEJ2.Villous_JEJ2 -> Liver.Liver_drug
	*switch_liverFlag*VillousJEJ2.Villous_JEJ2*Qmuc_JEJ2/VillousJEJ2*
148	MJEJ2.MEM_JEJ2 -> null
	*(CLINT_metabolism*metabolism_factor_jej2*switch_SFgutmet*MJEJ2.MEM_JEJ2*fu_mem)/MJEJ2*
149	VILL1.X_ILL1_SOLID -> VILL2.X_ILL2_SOLID
	VILL1.X_ILL1_SOLID/TILL1
150	VILL1.X_ILL1_SOLID -> VILL1.X_ILL1_DISS
	*KD*VILL1.X_ILL1_SOLID*(SOLIF_ILL1-VILL1.X_ILL1_DISS/VILL1)*
151	VILL1.X_ILL1_DISS -> VILL2.X_ILL2_DISS
	VILL1.X_ILL1_DISS/TILL1
152	VILL1.X_ILL1_DISS -> MILL1.MEM_ILL1
	*(DIFF_ill1*NI_ILL1*switch_SFdiffapi*VILL1.X_ILL1_DISS)/VILL1*
153	MILL1.MEM_ILL1 -> VILL1.X_ILL1_DISS
	*((switchVmax_efflux==zero)*CLINT_efflux_ILL1*efflux_factor_ill1*switch_SFefflux+switchVmax_efflux*phys_Normalized_ESA*phys_BW*surfaceRatio_ILL1*efflux_factor_ill1*switch_SFefflux/(drug_Km_efflux+MILL1.MEM_ILL1*fu_mem/MILL1/drug_molar_mass))*MILL1.MEM_ILL1*fu_mem/MILL1*
154	VILL1.X_ILL1_DISS -> MILL1.MEM_ILL1
	*((switchVmax_influx==zero)*CLINT_influx_ILL1*influx_factor_ill1*switch_SFinflux+switchVmax_influx*phys_Normalized_ESA*phys_BW*surfaceRatio_ILL1*influx_factor_ill1/(drug_Km_influx+VILL1.X_ILL1_DISS/VILL1/drug_molar_mass))*VILL1.X_ILL1_DISS/VILL1*
155	VillousILL1.Villous_ILL1 -> Liver.Liver_drug
	*switch_liverFlag*VillousILL1.Villous_ILL1*Qmuc_ILL1/VillousILL1*
156	MILL1.MEM_ILL1 -> null
	*(CLINT_metabolism*metabolism_factor_ill1*switch_SFgutmet*MILL1.MEM_ILL1*fu_mem)/MILL1*
157	VILL2.X_ILL2_SOLID -> VILL3.X_ILL3_SOLID
	VILL2.X_ILL2_SOLID/TILL2
158	VILL2.X_ILL2_SOLID -> VILL2.X_ILL2_DISS
	*KD*VILL2.X_ILL2_SOLID*(SOLIF_ILL2-VILL2.X_ILL2_DISS/VILL2)*
159	VILL2.X_ILL2_DISS -> VILL3.X_ILL3_DISS
	VILL2.X_ILL2_DISS/TILL2
160	VILL2.X_ILL2_DISS -> MILL2.MEM_ILL2
	*(DIFF_ill2*NI_ILL2*switch_SFdiffapi*VILL2.X_ILL2_DISS)/VILL2*
161	MILL2.MEM_ILL2 -> VILL2.X_ILL2_DISS
	*((switchVmax_efflux==zero)*CLINT_efflux_ILL2*efflux_factor_ill2*switch_SFefflux+switchVmax_efflux*phys_Normalized_ESA*phys_BW*surfaceRatio_ILL2*efflux_factor_ill2*switch_SFefflux/(drug_Km_efflux+MILL2.MEM_ILL2*fu_mem/MILL2/drug_molar_mass))*MILL2.MEM_ILL2*fu_mem/MILL2*
162	VILL2.X_ILL2_DISS -> MILL2.MEM_ILL2
	*((switchVmax_influx==zero)*CLINT_influx_ILL2*influx_factor_ill2*switch_SFinflux+switchVmax_influx*phys_Normalized_ESA*phys_BW*surfaceRatio_ILL2*influx_factor_ill2/(drug_Km_influx+VILL2.X_ILL2_DISS/VILL2/drug_molar_mass))*VILL2.X_ILL2_DISS/VILL2*
163	VillousILL2.Villous_ILL2 -> Liver.Liver_drug
	*switch_liverFlag*VillousILL2.Villous_ILL2*Qmuc_ILL2/VillousILL2*

	Reactions
164	MILL2.MEM_ILL2 -> null
	*(CLINT_metabolism*metabolism_factor_ill2*switch_SFgutmet*MILL2.MEM_ILL2*fu_mem)/MILL2*
165	VILL3.X_ILL3_SOLID -> VILL4.X_ILL4_SOLID
	VILL3.X_ILL3_SOLID/TILL3
166	VILL3.X_ILL3_SOLID -> VILL3.X_ILL3_DISS
	*KD*VILL3.X_ILL3_SOLID*(SOLIF_ILL3-VILL3.X_ILL3_DISS/VILL3)*
167	VILL3.X_ILL3_DISS -> VILL4.X_ILL4_DISS
	VILL3.X_ILL3_DISS/TILL3
168	VILL3.X_ILL3_DISS -> MILL3.MEM_ILL3
	*(DIFF_ill3*NI_ILL3*switch_SFdiffapi*VILL3.X_ILL3_DISS)/VILL3*
169	MILL3.MEM_ILL3 -> VILL3.X_ILL3_DISS
	*((switchVmax_efflux==zero)*CLINT_efflux_ILL3*efflux_factor_ill3*switch_SFefflux+switchVmax_efflux*phys_Normalized_ESA*phys_BW*surfaceRatio_ILL3*efflux_factor_ill3*switch_SFefflux/(drug_Km_efflux+MILL3.MEM_ILL3*fu_mem/MILL3/drug_molar_mass))*MILL3.MEM_ILL3*fu_mem/MILL3*
170	VILL3.X_ILL3_DISS -> MILL3.MEM_ILL3
	*((switchVmax_influx==zero)*CLINT_influx_ILL3*influx_factor_ill3*switch_SFinflux+switchVmax_influx*phys_Normalized_ESA*phys_BW*surfaceRatio_ILL3*influx_factor_ill3/(drug_Km_influx+VILL3.X_ILL3_DISS/VILL3/drug_molar_mass))*VILL3.X_ILL3_DISS/VILL3*
171	VillousILL3.Villous_ILL3 -> Liver.Liver_drug
	*switch_liverFlag*VillousILL3.Villous_ILL3*Qmuc_ILL3/VillousILL3*
172	MILL3.MEM_ILL3 -> null
	*(CLINT_metabolism*metabolism_factor_ill3*switch_SFgutmet*MILL3.MEM_ILL3*fu_mem)/MILL3*
173	VILL4.X_ILL4_SOLID -> Colon.X_CECUM_SOLID
	VILL4.X_ILL4_SOLID/TILL4
174	VILL4.X_ILL4_SOLID -> VILL4.X_ILL4_DISS
	*KD*VILL4.X_ILL4_SOLID*(SOLIF_ILL4-VILL4.X_ILL4_DISS/VILL4)*
175	VILL4.X_ILL4_DISS -> Colon.X_CECUM_DISS
	VILL4.X_ILL4_DISS/TILL4
176	VILL4.X_ILL4_DISS -> MILL4.MEM_ILL4
	*(DIFF_ill4*NI_ILL4*switch_SFdiffapi*VILL4.X_ILL4_DISS)/VILL4*
177	MILL4.MEM_ILL4 -> VILL4.X_ILL4_DISS
	*((switchVmax_efflux==zero)*CLINT_efflux_ILL4*efflux_factor_ill4*switch_SFefflux+switchVmax_efflux*phys_Normalized_ESA*phys_BW*surfaceRatio_ILL4*efflux_factor_ill4*switch_SFefflux/(drug_Km_efflux+MILL4.MEM_ILL4*fu_mem/MILL4/drug_molar_mass))*MILL4.MEM_ILL4*fu_mem/MILL4*
178	VILL4.X_ILL4_DISS -> MILL4.MEM_ILL4
	*((switchVmax_influx==zero)*CLINT_influx_ILL4*influx_factor_ill4*switch_SFinflux+switchVmax_influx*phys_Normalized_ESA*phys_BW*surfaceRatio_ILL4*influx_factor_ill4/(drug_Km_influx+VILL4.X_ILL4_DISS/VILL4/drug_molar_mass))*VILL4.X_ILL4_DISS/VILL4*
179	VillousILL4.Villous_ILL4 -> Liver.Liver_drug
	*switch_liverFlag*VillousILL4.Villous_ILL4*Qmuc_ILL4/VillousILL4*
180	MILL4.MEM_ILL4 -> null
	*(CLINT_metabolism*metabolism_factor_ill4*switch_SFgutmet*MILL4.MEM_ILL4*fu_mem)/MILL4*
181	Artery.Artery_drug -> VillousDUO.Villous_DUO
	*Qmuc_DUO*Artery.Artery_drug*

	Reactions
182	Artery.Artery_drug -> VillousJEJ1.Villous_JEJ1
	*Qmuc_JEJ1*Artery.Artery_drug*
183	Artery.Artery_drug -> VillousJEJ2.Villous_JEJ2
	*Qmuc_JEJ2*Artery.Artery_drug*
184	Artery.Artery_drug -> VillousILL2.Villous_ILL2
	*Qmuc_ILL2*Artery.Artery_drug*
185	Artery.Artery_drug -> VillousILL3.Villous_ILL3
	*Qmuc_ILL3*Artery.Artery_drug*
186	Artery.Artery_drug -> VillousILL4.Villous_ILL4
	*Qmuc_ILL4*Artery.Artery_drug*
187	STOMACH.X_STOMACH_SOLID -> STOMACH.X_STOMACH_DISS
	*KD*STOMACH.X_STOMACH_SOLID*(SOLIF_STOMACH-STOMACH.X_STOMACH_DISS/STOMACH)*
188	MDUO.MEM_DUO -> VillousDUO.Villous_DUO
	*DIFF_BASO_duo*switch_SFdiffbaso*MDUO.MEM_DUO*fu_mem/MDUO*
189	VillousDUO.Villous_DUO -> MDUO.MEM_DUO
	*DIFF_BASO_duo*switch_SFdiffbaso*VillousDUO.Villous_DUO*fu_blood/VillousDUO*
190	MDUO.MEM_DUO -> VDUO.X_DUO_DISS
	*DIFF_duo*switch_SFdiffapi*MDUO.MEM_DUO*fu_mem/MDUO*
191	MJEJ1.MEM_JEJ1 -> VillousJEJ1.Villous_JEJ1
	*DIFF_BASO_jej1*switch_SFdiffbaso*MJEJ1.MEM_JEJ1*fu_mem/MJEJ1*
192	VillousJEJ1.Villous_JEJ1 -> MJEJ1.MEM_JEJ1
	*DIFF_BASO_jej1*switch_SFdiffbaso*VillousJEJ1.Villous_JEJ1*fu_blood/VillousJEJ1*
193	MJEJ1.MEM_JEJ1 -> VJEJ1.X_JEJ1_DISS
	*DIFF_jej1*switch_SFdiffapi*MJEJ1.MEM_JEJ1*fu_mem/MJEJ1*
194	MJEJ2.MEM_JEJ2 -> VillousJEJ2.Villous_JEJ2
	*DIFF_BASO_jej2*switch_SFdiffbaso*MJEJ2.MEM_JEJ2*fu_mem/MJEJ2*
195	VillousJEJ2.Villous_JEJ2 -> MJEJ2.MEM_JEJ2
	*DIFF_BASO_jej2*switch_SFdiffbaso*VillousJEJ2.Villous_JEJ2*fu_blood/VillousJEJ2*
196	MJEJ2.MEM_JEJ2 -> VJEJ2.X_JEJ2_DISS
	*DIFF_jej2*switch_SFdiffapi*MJEJ2.MEM_JEJ2*fu_mem/MJEJ2*
197	MILL1.MEM_ILL1 -> VillousILL1.Villous_ILL1
	*DIFF_BASO_ill1*switch_SFdiffbaso*MILL1.MEM_ILL1*fu_mem/MILL1*
198	VillousILL1.Villous_ILL1 -> MILL1.MEM_ILL1
	*DIFF_BASO_ill1*switch_SFdiffbaso*VillousILL1.Villous_ILL1*fu_blood/VillousILL1*
199	MILL1.MEM_ILL1 -> VILL1.X_ILL1_DISS
	*DIFF_ill1*switch_SFdiffapi*MILL1.MEM_ILL1*fu_mem/MILL1*
200	VillousILL2.Villous_ILL2 -> MILL2.MEM_ILL2
	*DIFF_BASO_ill2*switch_SFdiffbaso*VillousILL2.Villous_ILL2*fu_blood/VillousILL2*
201	MILL2.MEM_ILL2 -> VillousILL2.Villous_ILL2
	*DIFF_BASO_ill2*switch_SFdiffbaso*MILL2.MEM_ILL2*fu_mem/MILL2*
202	MILL2.MEM_ILL2 -> VILL2.X_ILL2_DISS

	Reactions
	*DIFF_ill2*switch_SFdiffapi*MILL2.MEM_ILL2*fu_mem/MILL2*
203	VillousILL3.Villous_ILL3 -> MILL3.MEM_ILL3
	*DIFF_BASO_ill3*switch_SFdiffbaso*VillousILL3.Villous_ILL3*fu_blood/VillousILL3*
204	MILL3.MEM_ILL3 -> VillousILL3.Villous_ILL3
	*DIFF_BASO_ill3*switch_SFdiffbaso*MILL3.MEM_ILL3*fu_mem/MILL3*
205	MILL3.MEM_ILL3 -> VILL3.X_ILL3_DISS
	*DIFF_ill3*switch_SFdiffapi*MILL3.MEM_ILL3*fu_mem/MILL3*
206	MILL4.MEM_ILL4 -> VILL4.X_ILL4_DISS
	*DIFF_ill4*switch_SFdiffapi*MILL4.MEM_ILL4*fu_mem/MILL4*
207	VillousILL4.Villous_ILL4 -> MILL4.MEM_ILL4
	*DIFF_BASO_ill4*switch_SFdiffbaso*VillousILL4.Villous_ILL4*fu_blood/VillousILL4*
208	MILL4.MEM_ILL4 -> VillousILL4.Villous_ILL4
	*DIFF_BASO_ill4*switch_SFdiffbaso*MILL4.MEM_ILL4*fu_mem/MILL4*
209	Liver.Liver_drug -> Main_compartment.Bile_drug
	*switch_liverFlag*k_liver_bile*Liver.Liver_drug*
210	Liver.Liver_drug -> Metabolites.Metabolites_drug
	*switch_liverFlag*k_liver_metabolites*Liver.Liver_drug*
211	Artery.Artery_drug -> Serosa.Serosa_drug
	*k_artery_serosa*Artery.Artery_drug*
212	Serosa.Serosa_drug -> Liver.Liver_drug
	*switch_liverFlag*k_serosa_liver*Serosa.Serosa_drug*
213	VillousDUO.Villous_DUO -> MDUO.MEM_DUO
	*CLINT_influx_baso_DUO*influx_factor_duo_baso*switch_SFinflux*VillousDUO.Villous_DUO/VillousDUO*
214	VillousJEJ1.Villous_JEJ1 -> MJEJ1.MEM_JEJ1
	*CLINT_influx_baso_JEJ1*influx_factor_jej1_baso*switch_SFinflux*VillousJEJ1.Villous_JEJ1/VillousJEJ1*
215	VillousJEJ2.Villous_JEJ2 -> MJEJ2.MEM_JEJ2
	*CLINT_influx_baso_JEJ2*influx_factor_jej2_baso*switch_SFinflux*VillousJEJ2.Villous_JEJ2/VillousJEJ2*
216	VillousILL1.Villous_ILL1 -> MILL1.MEM_ILL1
	*CLINT_influx_baso_ILL1*influx_factor_ill1_baso*switch_SFinflux*VillousILL1.Villous_ILL1/VillousILL1*
217	VillousILL2.Villous_ILL2 -> MILL2.MEM_ILL2
	*CLINT_influx_baso_ILL2*influx_factor_ill2_baso*switch_SFinflux*VillousILL2.Villous_ILL2/VillousILL2*
218	VillousILL3.Villous_ILL3 -> MILL3.MEM_ILL3
	*CLINT_influx_baso_ILL3*influx_factor_ill3_baso*switch_SFinflux*VillousILL3.Villous_ILL3/VillousILL3*
219	VillousILL4.Villous_ILL4 -> MILL4.MEM_ILL4
	*CLINT_influx_baso_ILL4*influx_factor_ill4_baso*switch_SFinflux*VillousILL4.Villous_ILL4/VillousILL4*
220	Spleen.Spleen_drug -> Liver_EC_S1.Liver_EC_S1_drug
	*(1-switch_liverFlag)*k_spleen_liver*Spleen.Spleen_drug*
221	Artery.Artery_drug -> Liver_EC_S1.Liver_EC_S1_drug
	*(1-switch_liverFlag)*k_artery_liver*Artery.Artery_drug*
222	VillousDUO.Villous_DUO -> Liver_EC_S1.Liver_EC_S1_drug
	*(1-switch_liverFlag)*VillousDUO.Villous_DUO*Qmuc_DUO/VillousDUO*

	Reactions
223	VillousJEJ1.Villous_JEJ1 -> Liver_EC_S1.Liver_EC_S1_drug
	*(1-switch_liverFlag)*VillousJEJ1.Villous_JEJ1*Qmuc_JEJ1/VillousJEJ1*
224	VillousJEJ2.Villous_JEJ2 -> Liver_EC_S1.Liver_EC_S1_drug
	*(1-switch_liverFlag)*VillousJEJ2.Villous_JEJ2*Qmuc_JEJ2/VillousJEJ2*
225	VillousILL1.Villous_ILL1 -> Liver_EC_S1.Liver_EC_S1_drug
	*(1-switch_liverFlag)*VillousILL1.Villous_ILL1*Qmuc_ILL1/VillousILL1*
226	VillousILL2.Villous_ILL2 -> Liver_EC_S1.Liver_EC_S1_drug
	*(1-switch_liverFlag)*VillousILL2.Villous_ILL2*Qmuc_ILL2/VillousILL2*
227	VillousILL3.Villous_ILL3 -> Liver_EC_S1.Liver_EC_S1_drug
	*(1-switch_liverFlag)*VillousILL3.Villous_ILL3*Qmuc_ILL3/VillousILL3*
228	VillousILL4.Villous_ILL4 -> Liver_EC_S1.Liver_EC_S1_drug
	*(1-switch_liverFlag)*VillousILL4.Villous_ILL4*Qmuc_ILL4/VillousILL4*
229	Serosa.Serosa_drug -> Liver_EC_S1.Liver_EC_S1_drug
	*(1-switch_liverFlag)*k_serosa_liver*Serosa.Serosa_drug*
230	Liver_EC_S5.Liver_EC_S5_drug -> Venous.Venous_drug
	*(1-switch_liverFlag)*k_Liver_EC_S5_Venous*Liver_EC_S5.Liver_EC_S5_drug*
231	STOMACH_1.X_STOMACH_SOLID_1 -> VDUO_1.X_DUO_SOLID_1
	STOMACH_1.X_STOMACH_SOLID_1/TSTOMACH
232	STOMACH_1.X_STOMACH_DISS_1 -> VDUO_1.X_DUO_DISS_1
	STOMACH_1.X_STOMACH_DISS_1/TSTOMACH
233	VDUO_1.X_DUO_SOLID_1 -> VJEJ1_1.X_JEJ1_SOLID_1
	VDUO_1.X_DUO_SOLID_1/TDUO
234	VDUO_1.X_DUO_SOLID_1 -> VDUO_1.X_DUO_DISS_1
	*KD_1*VDUO_1.X_DUO_SOLID_1*(SOLIF_DUO_1-VDUO_1.X_DUO_DISS_1/VDUO_1)*
235	VDUO_1.X_DUO_DISS_1 -> VJEJ1_1.X_JEJ1_DISS_1
	VDUO_1.X_DUO_DISS_1/TDUO
236	VDUO_1.X_DUO_DISS_1 -> MDUO_1.MEM_DUO_1
	*(DIFF_duo_1*NI_DUO_1*switch_SFdiffapi_1*VDUO_1.X_DUO_DISS_1)/VDUO_1*
237	MDUO_1.MEM_DUO_1 -> VDUO_1.X_DUO_DISS_1
	*((switchVmax_efflux_1==zero_1)*efflux_inhib_duo*CLINT_efflux_DUO_1*efflux_factor_duo*switch_SFefflux_1+efflux_inhib_duo*switchVmax_efflux_1*phys_Normalized_ESA*phys_BW*surfaceRatio_DUO*efflux_factor_duo*switch_SFefflux_1/(drug_Km_efflux_1+MDUO_1.MEM_DUO_1*fu_mem_1/MDUO_1/drug_molar_mass_1))*MDUO_1.MEM_DUO_1*fu_mem_1/MDUO_1*
238	VDUO_1.X_DUO_DISS_1 -> MDUO_1.MEM_DUO_1
	*((switchVmax_influx_1==zero)*CLINT_influx_DUO_1*influx_factor_duo*switch_SFinflux_1+switchVmax_influx_1*phys_Normalized_ESA*phys_BW*surfaceRatio_DUO*influx_factor_duo/(drug_Km_influx_1+VDUO_1.X_DUO_DISS_1/VDUO_1/drug_molar_mass_1))*VDUO_1.X_DUO_DISS_1/VDUO_1*
239	MDUO_1.MEM_DUO_1 -> null
	*(CLINT_metabolism_1*metabolism_factor_duo_1*switch_SFgutmet_1*MDUO_1.MEM_DUO_1*fu_mem_1)/MDUO_1*
240	VJEJ1_1.X_JEJ1_SOLID_1 -> VJEJ2_1.X_JEJ2_SOLID_1
	VJEJ1_1.X_JEJ1_SOLID_1/TJEJ1
241	VJEJ1_1.X_JEJ1_SOLID_1 -> VJEJ1_1.X_JEJ1_DISS_1
	*KD_1*VJEJ1_1.X_JEJ1_SOLID_1*(SOLIF_JEJ1_1-VJEJ1_1.X_JEJ1_DISS_1/VJEJ1_1)*

	Reactions
242	VJEJ1_1.X_JEJ1_DISS_1 -> VJEJ2_1.X_JEJ2_DISS_1<br>*VJEJ1_1.X_JEJ1_DISS_1/TJEJ1*
243	VJEJ1_1.X_JEJ1_DISS_1 -> MJEJ1_1.MEM_JEJ1_1<br>*(DIFF_jej1_1*NI_JEJ1_1*switch_SFdiffapi_1*VJEJ1_1.X_JEJ1_DISS_1)/VJEJ1_1*
244	MJEJ1_1.MEM_JEJ1_1 -> VJEJ1_1.X_JEJ1_DISS_1<br>*((switchVmax_efflux_1==zero)*efflux_inhib_jej1*CLINT_efflux_JEJ1_1*efflux_factor_jej1*switch_SFefflux_1+efflux_inhib_jej1*switchVmax_efflux_1*phys_Normalized_ESA*phys_BW*surfaceRatio_JEJ1*efflux_factor_jej1*switch_SFefflux_1/(drug_Km_efflux_1+MJEJ1_1.MEM_JEJ1_1*fu_mem_1/MJEJ1_1/drug_molar_mass_1))*MJEJ1_1.MEM_JEJ1_1*fu_mem_1/MJEJ1_1*
245	VJEJ1_1.X_JEJ1_DISS_1 -> MJEJ1_1.MEM_JEJ1_1<br>*((switchVmax_influx_1==zero)*CLINT_influx_JEJ1_1*influx_factor_jej1*switch_SFinflux_1+switchVmax_influx_1*phys_Normalized_ESA*phys_BW*surfaceRatio_JEJ1*influx_factor_jej1/(drug_Km_influx_1+VJEJ1_1.X_JEJ1_DISS_1/VJEJ1_1/drug_molar_mass_1))*VJEJ1_1.X_JEJ1_DISS_1/VJEJ1_1*
246	MJEJ1_1.MEM_JEJ1_1 -> null<br>*(CLINT_metabolism_1*metabolism_factor_jej1_1*switch_SFgutmet_1*MJEJ1_1.MEM_JEJ1_1*fu_mem_1)/MJEJ1_1*
247	VJEJ2_1.X_JEJ2_SOLID_1 -> VILL1_1.X_ILL1_SOLID_1<br>*VJEJ2_1.X_JEJ2_SOLID_1/TJEJ2*
248	VJEJ2_1.X_JEJ2_SOLID_1 -> VJEJ2_1.X_JEJ2_DISS_1<br>*KD_1*VJEJ2_1.X_JEJ2_SOLID_1*(SOLIF_JEJ2_1-VJEJ2_1.X_JEJ2_DISS_1/VJEJ2_1)*
249	VJEJ2_1.X_JEJ2_DISS_1 -> VILL1_1.X_ILL1_DISS_1<br>*VJEJ2_1.X_JEJ2_DISS_1/TJEJ2*
250	VJEJ2_1.X_JEJ2_DISS_1 -> MJEJ2_1.MEM_JEJ2_1<br>*(DIFF_jej2_1*NI_JEJ2_1*switch_SFdiffapi_1*VJEJ2_1.X_JEJ2_DISS_1)/VJEJ2_1*
251	MJEJ2_1.MEM_JEJ2_1 -> VJEJ2_1.X_JEJ2_DISS_1<br>*((switchVmax_efflux_1==zero)*efflux_inhib_jej2*CLINT_efflux_JEJ2_1*efflux_factor_jej2*switch_SFefflux_1+efflux_inhib_jej2*switchVmax_efflux_1*phys_Normalized_ESA*phys_BW*surfaceRatio_JEJ2*efflux_factor_jej2*switch_SFefflux_1/(drug_Km_efflux_1+MJEJ2_1.MEM_JEJ2_1*fu_mem_1/MJEJ2_1/drug_molar_mass_1))*MJEJ2_1.MEM_JEJ2_1*fu_mem_1/MJEJ2_1*
252	VJEJ2_1.X_JEJ2_DISS_1 -> MJEJ2_1.MEM_JEJ2_1<br>*((switchVmax_influx_1==zero)*CLINT_influx_JEJ2_1*influx_factor_jej2*switch_SFinflux_1+switchVmax_influx_1*phys_Normalized_ESA*phys_BW*surfaceRatio_JEJ2*influx_factor_jej2/(drug_Km_influx_1+VJEJ2_1.X_JEJ2_DISS_1/VJEJ2_1/drug_molar_mass_1))*VJEJ2_1.X_JEJ2_DISS_1/VJEJ2_1*
253	MJEJ2_1.MEM_JEJ2_1 -> null<br>*(CLINT_metabolism_1*metabolism_factor_jej2_1*switch_SFgutmet_1*MJEJ2_1.MEM_JEJ2_1*fu_mem_1)/MJEJ2_1*
254	VILL1_1.X_ILL1_SOLID_1 -> VILL2_1.X_ILL2_SOLID_1<br>*VILL1_1.X_ILL1_SOLID_1/TILL1*
255	VILL1_1.X_ILL1_SOLID_1 -> VILL1_1.X_ILL1_DISS_1<br>*KD_1*VILL1_1.X_ILL1_SOLID_1*(SOLIF_ILL1_1-VILL1_1.X_ILL1_DISS_1/VILL1_1)*
256	VILL1_1.X_ILL1_DISS_1 -> VILL2_1.X_ILL2_DISS_1<br>*VILL1_1.X_ILL1_DISS_1/TILL1*
257	VILL1_1.X_ILL1_DISS_1 -> MILL1_1.MEM_ILL1_1<br>*(DIFF_ill1_1*NI_ILL1_1*switch_SFdiffapi_1*VILL1_1.X_ILL1_DISS_1)/VILL1_1*
258	MILL1_1.MEM_ILL1_1 -> VILL1_1.X_ILL1_DISS_1<br>*((switchVmax_efflux_1==zero)*efflux_inhib_ill1*CLINT_efflux_ILL1_1*efflux_factor_ill1*switch_SFefflux_1+efflux_inhib_ill1*switchVmax_efflux_1*phys_Normalized_ESA*phys_BW*surfaceRatio_ILL1*efflux_factor_ill1*switch_SFefflux_1/(drug_*

	Reactions
	Km_efflux_1+MILL1_1.MEM_ILL1_1*fu_mem_1/MILL1_1/drug_molar_mass_1))*MILL1_1.MEM_ILL1_1*fu_mem_1/MILL1_1
259	VILL1_1.X_ILL1_DISS_1 -> MILL1_1.MEM_ILL1_1
	((switchVmax_influx_1==zero)*CLINT_influx_ILL1_1*influx_factor_ill1*switch_SFinflux_1+switchVmax_influx_1*phys_Normalized_ESA*phys_BW*surfaceRatio_ILL1*influx_factor_ill1/(drug_Km_influx_1+VILL1_1.X_ILL1_DISS_1/VILL1_1/drug_molar_mass_1))*VILL1_1.X_ILL1_DISS_1/VILL1_1
260	MILL1_1.MEM_ILL1_1 -> null
	(CLINT_metabolism_1*metabolism_factor_ill1_1*switch_SFgutmet_1*MILL1_1.MEM_ILL1_1*fu_mem_1)/MILL1_1
261	VILL2_1.X_ILL2_SOLID_1 -> VILL3_1.X_ILL3_SOLID_1
	VILL2_1.X_ILL2_SOLID_1/TILL2
262	VILL2_1.X_ILL2_SOLID_1 -> VILL2_1.X_ILL2_DISS_1
	KD_1*VILL2_1.X_ILL2_SOLID_1*(SOLIF_ILL2_1-VILL2_1.X_ILL2_DISS_1/VILL2_1)
263	VILL2_1.X_ILL2_DISS_1 -> VILL3_1.X_ILL3_DISS_1
	VILL2_1.X_ILL2_DISS_1/TILL2
264	VILL2_1.X_ILL2_DISS_1 -> MILL2_1.MEM_ILL2_1
	(DIFF_ill2_1*NI_ILL2_1*switch_SFdiffapi_1*VILL2_1.X_ILL2_DISS_1)/VILL2_1
265	MILL2_1.MEM_ILL2_1 -> VILL2_1.X_ILL2_DISS_1
	((switchVmax_efflux_1==zero)*efflux_inhib_ill2*CLINT_efflux_ILL2_1*efflux_factor_ill2*switch_SFefflux_1+efflux_inhib_ill2*switchVmax_efflux_1*phys_Normalized_ESA*phys_BW*surfaceRatio_ILL2*efflux_factor_ill2*switch_SFefflux_1/(drug_Km_efflux_1+MILL2_1.MEM_ILL2_1*fu_mem_1/MILL2_1/drug_molar_mass_1))*MILL2_1.MEM_ILL2_1*fu_mem_1/MILL2_1
266	VILL2_1.X_ILL2_DISS_1 -> MILL2_1.MEM_ILL2_1
	((switchVmax_influx_1==zero)*CLINT_influx_ILL2_1*influx_factor_ill2*switch_SFinflux_1+switchVmax_influx_1*phys_Normalized_ESA*phys_BW*surfaceRatio_ILL2*influx_factor_ill2/(drug_Km_influx_1+VILL2_1.X_ILL2_DISS_1/VILL2_1/drug_molar_mass_1))*VILL2_1.X_ILL2_DISS_1/VILL2_1
267	MILL2_1.MEM_ILL2_1 -> null
	(CLINT_metabolism_1*metabolism_factor_ill2_1*switch_SFgutmet_1*MILL2_1.MEM_ILL2_1*fu_mem_1)/MILL2_1
268	VILL3_1.X_ILL3_SOLID_1 -> VILL4_1.X_ILL4_SOLID_1
	VILL3_1.X_ILL3_SOLID_1/TILL3
269	VILL3_1.X_ILL3_SOLID_1 -> VILL3_1.X_ILL3_DISS_1
	KD_1*VILL3_1.X_ILL3_SOLID_1*(SOLIF_ILL3_1-VILL3_1.X_ILL3_DISS_1/VILL3_1)
270	VILL3_1.X_ILL3_DISS_1 -> VILL4_1.X_ILL4_DISS_1
	VILL3_1.X_ILL3_DISS_1/TILL3
271	VILL3_1.X_ILL3_DISS_1 -> MILL3_1.MEM_ILL3_1
	(DIFF_ill3_1*NI_ILL3_1*switch_SFdiffapi_1*VILL3_1.X_ILL3_DISS_1)/VILL3_1
272	MILL3_1.MEM_ILL3_1 -> VILL3_1.X_ILL3_DISS_1
	((switchVmax_efflux_1==zero)*efflux_inhib_ill3*CLINT_efflux_ILL3_1*efflux_factor_ill3*switch_SFefflux_1+efflux_inhib_ill3*switchVmax_efflux_1*phys_Normalized_ESA*phys_BW*surfaceRatio_ILL3*efflux_factor_ill3*switch_SFefflux_1/(drug_Km_efflux_1+MILL3_1.MEM_ILL3_1*fu_mem_1/MILL3_1/drug_molar_mass_1))*MILL3_1.MEM_ILL3_1*fu_mem_1/MILL3_1
273	VILL3_1.X_ILL3_DISS_1 -> MILL3_1.MEM_ILL3_1
	((switchVmax_influx_1==zero)*CLINT_influx_ILL3_1*influx_factor_ill3*switch_SFinflux_1+switchVmax_influx_1*phys_Normalized_ESA*phys_BW*surfaceRatio_ILL3*influx_factor_ill3/(drug_Km_influx_1+VILL3_1.X_ILL3_DISS_1/VILL3_1/drug_molar_mass_1))*VILL3_1.X_ILL3_DISS_1/VILL3_1
274	MILL3_1.MEM_ILL3_1 -> null
	(CLINT_metabolism_1*metabolism_factor_ill3_1*switch_SFgutmet_1*MILL3_1.MEM_ILL3_1*fu_mem_1)/MILL3_1

	Reactions
275	VILL4_1.X_ILL4_SOLID_1 -> Colon_1.X_CECUM_SOLID_1
	VILL4_1.X_ILL4_SOLID_1/TILL4
276	VILL4_1.X_ILL4_SOLID_1 -> VILL4_1.X_ILL4_DISS_1
	*KD_1*VILL4_1.X_ILL4_SOLID_1*(SOLIF_ILL4_1-VILL4_1.X_ILL4_DISS_1/VILL4_1)*
277	VILL4_1.X_ILL4_DISS_1 -> Colon_1.X_CECUM_DISS_1
	VILL4_1.X_ILL4_DISS_1/TILL4
278	VILL4_1.X_ILL4_DISS_1 -> MILL4_1.MEM_ILL4_1
	*(DIFF_ill4_1*NI_ILL4_1*switch_SFdiffapi_1*VILL4_1.X_ILL4_DISS_1)/VILL4_1*
279	MILL4_1.MEM_ILL4_1 -> VILL4_1.X_ILL4_DISS_1
	*((switchVmax_efflux_1==zero)*efflux_inhib_ill4*CLINT_efflux_ILL4_1*efflux_factor_ill4*switch_SFefflux_1+efflux_inhib_ill4*switchVmax_efflux_1*phys_Normalized_ESA*phys_BW*surfaceRatio_ILL4*efflux_factor_ill4*switch_SFefflux_1/(drug_Km_efflux_1+MILL4_1.MEM_ILL4_1*fu_mem_1/MILL4_1/drug_molar_mass_1))*MILL4_1.MEM_ILL4_1*fu_mem_1/MILL4_1*
280	VILL4_1.X_ILL4_DISS_1 -> MILL4_1.MEM_ILL4_1
	*((switchVmax_influx_1==zero)*CLINT_influx_ILL4_1*influx_factor_ill4*switch_SFinflux_1+switchVmax_influx_1*phys_Normalized_ESA*phys_BW*surfaceRatio_ILL4*influx_factor_ill4/(drug_Km_influx_1+VILL4_1.X_ILL4_DISS_1/VILL4_1/drug_molar_mass_1))*VILL4_1.X_ILL4_DISS_1/VILL4_1*
281	MILL4_1.MEM_ILL4_1 -> null
	*(CLINT_metabolism_1*metabolism_factor_ill4_1*switch_SFgutmet_1*MILL4_1.MEM_ILL4_1*fu_mem_1)/MILL4_1*
282	STOMACH_1.X_STOMACH_SOLID_1 -> STOMACH_1.X_STOMACH_DISS_1
	*KD_1*STOMACH_1.X_STOMACH_SOLID_1*(SOLIF_STOMACH_1-STOMACH_1.X_STOMACH_DISS_1/STOMACH_1)*
283	MDUO_1.MEM_DUO_1 -> VillousDUO_1.Villous_DUO_1
	*DIFF_BASO_duo_1*switch_SFdiffbaso_1*MDUO_1.MEM_DUO_1*fu_mem_1/MDUO_1*
284	VillousDUO_1.Villous_DUO_1 -> MDUO_1.MEM_DUO_1
	*DIFF_BASO_duo_1*switch_SFdiffbaso_1*VillousDUO_1.Villous_DUO_1*fu_blood_1/VillousDUO_1*
285	MDUO_1.MEM_DUO_1 -> VDUO_1.X_DUO_DISS_1
	*DIFF_duo_1*switch_SFdiffapi_1*MDUO_1.MEM_DUO_1*fu_mem_1/MDUO_1*
286	MJEJ1_1.MEM_JEJ1_1 -> VillousJEJ1_1.Villous_JEJ1_1
	*DIFF_BASO_jej1_1*switch_SFdiffbaso_1*MJEJ1_1.MEM_JEJ1_1*fu_mem_1/MJEJ1_1*
287	VillousJEJ1_1.Villous_JEJ1_1 -> MJEJ1_1.MEM_JEJ1_1
	*DIFF_BASO_jej1_1*switch_SFdiffbaso_1*VillousJEJ1_1.Villous_JEJ1_1*fu_blood_1/VillousJEJ1_1*
288	MJEJ1_1.MEM_JEJ1_1 -> VJEJ1_1.X_JEJ1_DISS_1
	*DIFF_jej1_1*switch_SFdiffapi_1*MJEJ1_1.MEM_JEJ1_1*fu_mem_1/MJEJ1_1*
289	MJEJ2_1.MEM_JEJ2_1 -> VillousJEJ2_1.Villous_JEJ2_1
	*DIFF_BASO_jej2_1*switch_SFdiffbaso_1*MJEJ2_1.MEM_JEJ2_1*fu_mem_1/MJEJ2_1*
290	VillousJEJ2_1.Villous_JEJ2_1 -> MJEJ2_1.MEM_JEJ2_1
	*DIFF_BASO_jej2_1*switch_SFdiffbaso_1*VillousJEJ2_1.Villous_JEJ2_1*fu_blood_1/VillousJEJ2_1*
291	MJEJ2_1.MEM_JEJ2_1 -> VJEJ2_1.X_JEJ2_DISS_1
	*DIFF_jej2_1*switch_SFdiffapi_1*MJEJ2_1.MEM_JEJ2_1*fu_mem_1/MJEJ2_1*
292	MILL1_1.MEM_ILL1_1 -> VillousILL1_1.Villous_ILL1_1
	*DIFF_BASO_ill1_1*switch_SFdiffbaso_1*MILL1_1.MEM_ILL1_1*fu_mem_1/MILL1_1*
293	VillousILL1_1.Villous_ILL1_1 -> MILL1_1.MEM_ILL1_1

	Reactions
	*DIFF_BASO_ill1_1*switch_SFdiffbaso_1*VillousILL1_1.Villous_ILL1_1*fu_blood_1/VillousILL1_1*
294	MILL1_1.MEM_ILL1_1 -> VILL1_1.X_ILL1_DISS_1
	*DIFF_ill1_1*switch_SFdiffapi_1*MILL1_1.MEM_ILL1_1*fu_mem_1/MILL1_1*
295	VillousILL2_1.Villous_ILL2_1 -> MILL2_1.MEM_ILL2_1
	*DIFF_BASO_ill2_1*switch_SFdiffbaso_1*VillousILL2_1.Villous_ILL2_1*fu_blood_1/VillousILL2_1*
296	MILL2_1.MEM_ILL2_1 -> VillousILL2_1.Villous_ILL2_1
	*DIFF_BASO_ill2_1*switch_SFdiffbaso_1*MILL2_1.MEM_ILL2_1*fu_mem_1/MILL2_1*
297	MILL2_1.MEM_ILL2_1 -> VILL2_1.X_ILL2_DISS_1
	*DIFF_ill2_1*switch_SFdiffapi_1*MILL2_1.MEM_ILL2_1*fu_mem_1/MILL2_1*
298	VillousILL3_1.Villous_ILL3_1 -> MILL3_1.MEM_ILL3_1
	*DIFF_BASO_ill3_1*switch_SFdiffbaso_1*VillousILL3_1.Villous_ILL3_1*fu_blood_1/VillousILL3_1*
299	MILL3_1.MEM_ILL3_1 -> VillousILL3_1.Villous_ILL3_1
	*DIFF_BASO_ill3_1*switch_SFdiffbaso_1*MILL3_1.MEM_ILL3_1*fu_mem_1/MILL3_1*
300	MILL3_1.MEM_ILL3_1 -> VILL3_1.X_ILL3_DISS_1
	*DIFF_ill3_1*switch_SFdiffapi_1*MILL3_1.MEM_ILL3_1*fu_mem_1/MILL3_1*
301	MILL4_1.MEM_ILL4_1 -> VILL4_1.X_ILL4_DISS_1
	*DIFF_ill4_1*switch_SFdiffapi_1*MILL4_1.MEM_ILL4_1*fu_mem_1/MILL4_1*
302	VillousILL4_1.Villous_ILL4_1 -> MILL4_1.MEM_ILL4_1
	*DIFF_BASO_ill4_1*switch_SFdiffbaso_1*VillousILL4_1.Villous_ILL4_1*fu_blood_1/VillousILL4_1*
303	MILL4_1.MEM_ILL4_1 -> VillousILL4_1.Villous_ILL4_1
	*DIFF_BASO_ill4_1*switch_SFdiffbaso_1*MILL4_1.MEM_ILL4_1*fu_mem_1/MILL4_1*
304	VillousDUO_1.Villous_DUO_1 -> MDUO_1.MEM_DUO_1
	*CLINT_influx_baso_DUO_1*influx_factor_duo_baso*switch_SFinflux_1*VillousDUO_1.Villous_DUO_1/VillousDUO_1*
305	VillousJEJ1_1.Villous_JEJ1_1 -> MJEJ1_1.MEM_JEJ1_1
	*CLINT_influx_baso_JEJ1_1*influx_factor_jej1_baso*switch_SFinflux_1*VillousJEJ1_1.Villous_JEJ1_1/VillousJEJ1_1*
306	VillousJEJ2_1.Villous_JEJ2_1 -> MJEJ2_1.MEM_JEJ2_1
	*CLINT_influx_baso_JEJ2_1*influx_factor_jej2_baso*switch_SFinflux_1*VillousJEJ2_1.Villous_JEJ2_1/VillousJEJ2_1*
307	VillousILL1_1.Villous_ILL1_1 -> MILL1_1.MEM_ILL1_1
	*CLINT_influx_baso_ILL1_1*influx_factor_ill1_baso*switch_SFinflux_1*VillousILL1_1.Villous_ILL1_1/VillousILL1_1*
308	VillousILL2_1.Villous_ILL2_1 -> MILL2_1.MEM_ILL2_1
	*CLINT_influx_baso_ILL2_1*influx_factor_ill2_baso*switch_SFinflux_1*VillousILL2_1.Villous_ILL2_1/VillousILL2_1*
309	VillousILL3_1.Villous_ILL3_1 -> MILL3_1.MEM_ILL3_1
	*CLINT_influx_baso_ILL3_1*influx_factor_ill3_baso*switch_SFinflux_1*VillousILL3_1.Villous_ILL3_1/VillousILL3_1*
310	VillousILL4_1.Villous_ILL4_1 -> MILL4_1.MEM_ILL4_1
	*CLINT_influx_baso_ILL4_1*influx_factor_ill4_baso*switch_SFinflux_1*VillousILL4_1.Villous_ILL4_1/VillousILL4_1*
311	Artery_1.Artery_drug_1 -> VillousDUO_1.Villous_DUO_1
	*Qmuc_DUO_1*Artery_1.Artery_drug_1*
312	Artery_1.Artery_drug_1 -> VillousJEJ1_1.Villous_JEJ1_1
	*Qmuc_JEJ1_1*Artery_1.Artery_drug_1*
313	Artery_1.Artery_drug_1 -> VillousJEJ2_1.Villous_JEJ2_1
	*Qmuc_JEJ2_1*Artery_1.Artery_drug_1*

	Reactions
314	Artery_1.Artery_drug_1 -> VillousILL1_1.Villous_ILL1_1
	*Qmuc_ILL1_1*Artery_1.Artery_drug_1*
315	Artery_1.Artery_drug_1 -> VillousILL2_1.Villous_ILL2_1
	*Qmuc_ILL2_1*Artery_1.Artery_drug_1*
316	Artery_1.Artery_drug_1 -> VillousILL3_1.Villous_ILL3_1
	*Qmuc_ILL3_1*Artery_1.Artery_drug_1*
317	Artery_1.Artery_drug_1 -> VillousILL4_1.Villous_ILL4_1
	*Qmuc_ILL4_1*Artery_1.Artery_drug_1*
318	Liver_1.Liver_drug_1 -> Venous_1.Venous_drug_1
	*switch_liverFlag_1*k_Liver_Venous_1*Liver_1.Liver_drug_1*
319	VillousDUO_1.Villous_DUO_1 -> Liver_1.Liver_drug_1
	*switch_liverFlag_1*VillousDUO_1.Villous_DUO_1*Qmuc_DUO_1/VillousDUO_1*
320	VillousJEJ1_1.Villous_JEJ1_1 -> Liver_1.Liver_drug_1
	*switch_liverFlag_1*VillousJEJ1_1.Villous_JEJ1_1*Qmuc_JEJ1_1/VillousJEJ1_1*
321	VillousJEJ2_1.Villous_JEJ2_1 -> Liver_1.Liver_drug_1
	*switch_liverFlag_1*VillousJEJ2_1.Villous_JEJ2_1*Qmuc_JEJ2_1/VillousJEJ2_1*
322	VillousILL1_1.Villous_ILL1_1 -> Liver_1.Liver_drug_1
	*switch_liverFlag_1*VillousILL1_1.Villous_ILL1_1*Qmuc_ILL1_1/VillousILL1_1*
323	VillousILL2_1.Villous_ILL2_1 -> Liver_1.Liver_drug_1
	*switch_liverFlag_1*VillousILL2_1.Villous_ILL2_1*Qmuc_ILL2_1/VillousILL2_1*
324	VillousILL3_1.Villous_ILL3_1 -> Liver_1.Liver_drug_1
	*switch_liverFlag_1*VillousILL3_1.Villous_ILL3_1*Qmuc_ILL3_1/VillousILL3_1*
325	VillousILL4_1.Villous_ILL4_1 -> Liver_1.Liver_drug_1
	*switch_liverFlag_1*VillousILL4_1.Villous_ILL4_1*Qmuc_ILL4_1/VillousILL4_1*
326	VillousDUO_1.Villous_DUO_1 -> Liver_EC_S1_1.Liver_EC_S1_drug_1
	*(1-switch_liverFlag_1)*VillousDUO_1.Villous_DUO_1*Qmuc_DUO_1/VillousDUO_1*
327	VillousJEJ1_1.Villous_JEJ1_1 -> Liver_EC_S1_1.Liver_EC_S1_drug_1
	*(1-switch_liverFlag_1)*VillousJEJ1_1.Villous_JEJ1_1*Qmuc_JEJ1_1/VillousJEJ1_1*
328	VillousJEJ2_1.Villous_JEJ2_1 -> Liver_EC_S1_1.Liver_EC_S1_drug_1
	*(1-switch_liverFlag_1)*VillousJEJ2_1.Villous_JEJ2_1*Qmuc_JEJ2_1/VillousJEJ2_1*
329	VillousILL1_1.Villous_ILL1_1 -> Liver_EC_S1_1.Liver_EC_S1_drug_1
	*(1-switch_liverFlag_1)*VillousILL1_1.Villous_ILL1_1*Qmuc_ILL1_1/VillousILL1_1*
330	VillousILL2_1.Villous_ILL2_1 -> Liver_EC_S1_1.Liver_EC_S1_drug_1
	*(1-switch_liverFlag_1)*VillousILL2_1.Villous_ILL2_1*Qmuc_ILL2_1/VillousILL2_1*
331	VillousILL3_1.Villous_ILL3_1 -> Liver_EC_S1_1.Liver_EC_S1_drug_1
	*(1-switch_liverFlag_1)*VillousILL3_1.Villous_ILL3_1*Qmuc_ILL3_1/VillousILL3_1*
332	VillousILL4_1.Villous_ILL4_1 -> Liver_EC_S1_1.Liver_EC_S1_drug_1
	*(1-switch_liverFlag_1)*VillousILL4_1.Villous_ILL4_1*Qmuc_ILL4_1/VillousILL4_1*
333	Artery_1.Artery_drug_1 -> Serosa_1.Serosa_drug_1
	*k_artery_serosa_1*Artery_1.Artery_drug_1*
334	Serosa_1.Serosa_drug_1 -> Liver_1.Liver_drug_1

	Reactions
	switch_liverFlag_1*k_serosa_liver_1*Serosa_1.Serosa_drug_1
335	Serosa_1.Serosa_drug_1 -> Liver_EC_S1_1.Liver_EC_S1_drug_1
	(1-switch_liverFlag_1)*k_serosa_liver_1*Serosa_1.Serosa_drug_1
336	Artery_1.Artery_drug_1 -> Liver_1.Liver_drug_1
	switch_liverFlag_1*k_artery_liver_1*Artery_1.Artery_drug_1
337	Spleen_1.Spleen_drug_1 -> Liver_1.Liver_drug_1
	switch_liverFlag_1*k_spleen_liver_1*Spleen_1.Spleen_drug_1
338	Liver_1.Liver_drug_1 -> Metabolites_1.Metabolites_drug_1
	switch_liverFlag_1*k_liver_metabolites_1*Liver_1.Liver_drug_1
339	Main_compartment_1.Bile_drug_1 -> VDUO_1.X_DUO_DISS_1
	k_transit_1*Main_compartment_1.Bile_drug_1
340	Liver_1.Liver_drug_1 -> Main_compartment_1.Bile_drug_1
	switch_liverFlag_1*k_liver_bile_1*Liver_1.Liver_drug_1
341	MDUO.MEM_DUO -> VillousDUO.Villous_DUO
	((switchVmax_efflux_baso==zero)*CLINT_efflux_baso_DUO*switch_SFefflux_baso*baso_efflux_factor_duo+switchVmax_efflux_baso*baso_efflux_factor_duo*phys_Normalized_ESA_baso*phys_BW*basoSurfaceRatio_DUO*switch_SFefflux_baso/(drug_Km_efflux_baso+MEM_DUO*fu_mem/MDUO/drug_molar_mass))*MDUO.MEM_DUO*fu_mem/MDUO
342	MJEJ1.MEM_JEJ1 -> VillousJEJ1.Villous_JEJ1
	((switchVmax_efflux_baso==zero)*CLINT_efflux_baso_JEJ1*switch_SFefflux_baso*baso_efflux_factor_jej1+switchVmax_efflux_baso*baso_efflux_factor_jej1*phys_Normalized_ESA_baso*phys_BW*basoSurfaceRatio_JEJ1*switch_SFefflux_baso/(drug_Km_efflux_baso+MEM_JEJ1*fu_mem/MJEJ1/drug_molar_mass))*MJEJ1.MEM_JEJ1*fu_mem/MJEJ1
343	MJEJ2.MEM_JEJ2 -> VillousJEJ2.Villous_JEJ2
	((switchVmax_efflux_baso==zero)*CLINT_efflux_baso_JEJ2*switch_SFefflux_baso*baso_efflux_factor_jej2+switchVmax_efflux_baso*baso_efflux_factor_jej2*phys_Normalized_ESA_baso*phys_BW*basoSurfaceRatio_JEJ2*switch_SFefflux_baso/(drug_Km_efflux_baso+MEM_JEJ2*fu_mem/MJEJ2/drug_molar_mass))*MJEJ2.MEM_JEJ2*fu_mem/MJEJ2
344	MILL1.MEM_ILL1 -> VillousILL1.Villous_ILL1
	((switchVmax_efflux_baso==zero)*CLINT_efflux_baso_ILL1*switch_SFefflux_baso*baso_efflux_factor_ill1+switchVmax_efflux_baso*baso_efflux_factor_ill1*phys_Normalized_ESA_baso*phys_BW*basoSurfaceRatio_ILL1*switch_SFefflux_baso/(drug_Km_efflux_baso+MEM_ILL1*fu_mem/MILL1/drug_molar_mass))*MILL1.MEM_ILL1*fu_mem/MILL1
345	MILL2.MEM_ILL2 -> VillousILL2.Villous_ILL2
	((switchVmax_efflux_baso==zero)*CLINT_efflux_baso_ILL2*switch_SFefflux_baso*baso_efflux_factor_ill2+switchVmax_efflux_baso*baso_efflux_factor_ill2*phys_Normalized_ESA_baso*phys_BW*basoSurfaceRatio_ILL2*switch_SFefflux_baso/(drug_Km_efflux_baso+MEM_ILL2*fu_mem/MILL2/drug_molar_mass))*MILL2.MEM_ILL2*fu_mem/MILL2
346	MILL3.MEM_ILL3 -> VillousILL3.Villous_ILL3
	((switchVmax_efflux_baso==zero)*CLINT_efflux_baso_ILL3*switch_SFefflux_baso*baso_efflux_factor_ill3+switchVmax_efflux_baso*baso_efflux_factor_ill3*phys_Normalized_ESA_baso*phys_BW*basoSurfaceRatio_ILL3*switch_SFefflux_baso/(drug_Km_efflux_baso+MEM_ILL3*fu_mem/MILL3/drug_molar_mass))*MILL3.MEM_ILL3*fu_mem/MILL3
347	MILL4.MEM_ILL4 -> VillousILL4.Villous_ILL4
	((switchVmax_efflux_baso==zero)*CLINT_efflux_baso_ILL4*switch_SFefflux_baso*baso_efflux_factor_ill4+switchVmax_efflux_baso*baso_efflux_factor_ill4*phys_Normalized_ESA_baso*phys_BW*basoSurfaceRatio_ILL4*switch_SFefflux_baso/(drug_Km_efflux_baso+MEM_ILL4*fu_mem/MILL4/drug_molar_mass))*MILL4.MEM_ILL4*fu_mem/MILL4
348	MDUO_1.MEM_DUO_1 -> VillousDUO_1.Villous_DUO_1
	((switchVmax_efflux_baso_1==zero)*CLINT_efflux_baso_DUO_1*switch_SFefflux_baso_1*baso_efflux_factor_duo+switchVmax_efflux_baso_1*baso_efflux_factor_duo*phys_Normalized_ESA_baso*phys_BW*basoSurfaceRatio_DUO*switch_SFefflux_baso_1/(drug_Km_efflux_baso_1+MEM_DUO_1*fu_mem_1/MDUO_1/drug_molar_mass_1))*MDUO_1.MEM_DUO_1*fu_mem_1/MDUO_1
349	MJEJ1_1.MEM_JEJ1_1 -> VillousJEJ1_1.Villous_JEJ1_1

	Reactions
	*((switchVmax_efflux_baso_1==zero)*CLINT_efflux_baso_JEJ1_1*switch_SFefflux_baso_1*baso_efflux_factor_jej1+switchVmax_efflux_baso_1*baso_efflux_factor_jej1*phys_Normalized_ESA_baso*phys_BW*basoSurfaceRatio_JEJ1*switch_SFefflux_baso_1/(drug_Km_efflux_baso_1+MEM_JEJ1_1*fu_mem_1/MJEJ1_1/drug_molar_mass_1))*MJEJ1_1.MEM_JEJ1_1*fu_mem_1/MJEJ1_1*
350	MJEJ2_1.MEM_JEJ2_1 -> VillousJEJ2_1.Villous_JEJ2_1
	*((switchVmax_efflux_baso_1==zero)*CLINT_efflux_baso_JEJ2_1*switch_SFefflux_baso_1*baso_efflux_factor_jej2+switchVmax_efflux_baso_1*baso_efflux_factor_jej2*phys_Normalized_ESA_baso*phys_BW*basoSurfaceRatio_JEJ2*switch_SFefflux_baso_1/(drug_Km_efflux_baso_1+MEM_JEJ2_1*fu_mem_1/MJEJ2_1/drug_molar_mass_1))*MJEJ2_1.MEM_JEJ2_1*fu_mem_1/MJEJ2_1*
351	MILL1_1.MEM_ILL1_1 -> VillousILL1_1.Villous_ILL1_1
	*((switchVmax_efflux_baso_1==zero)*CLINT_efflux_baso_ILL1_1*switch_SFefflux_baso_1*baso_efflux_factor_ill1+switchVmax_efflux_baso_1*baso_efflux_factor_ill1*phys_Normalized_ESA_baso*phys_BW*basoSurfaceRatio_ILL1*switch_SFefflux_baso_1/(drug_Km_efflux_baso_1+MEM_ILL1_1*fu_mem_1/MILL1_1/drug_molar_mass_1))*MILL1_1.MEM_ILL1_1*fu_mem_1/MILL1_1*
352	MILL2_1.MEM_ILL2_1 -> VillousILL2_1.Villous_ILL2_1
	*((switchVmax_efflux_baso_1==zero)*CLINT_efflux_baso_ILL2_1*switch_SFefflux_baso_1*baso_efflux_factor_ill2+switchVmax_efflux_baso_1*baso_efflux_factor_ill2*phys_Normalized_ESA_baso*phys_BW*basoSurfaceRatio_ILL2*switch_SFefflux_baso_1/(drug_Km_efflux_baso_1+MEM_ILL2_1*fu_mem_1/MILL2_1/drug_molar_mass_1))*MILL2_1.MEM_ILL2_1*fu_mem_1/MILL2_1*
353	MILL3_1.MEM_ILL3_1 -> VillousILL3_1.Villous_ILL3_1
	*((switchVmax_efflux_baso_1==zero)*CLINT_efflux_baso_ILL3_1*switch_SFefflux_baso_1*baso_efflux_factor_ill3+switchVmax_efflux_baso_1*baso_efflux_factor_ill3*phys_Normalized_ESA_baso*phys_BW*basoSurfaceRatio_ILL3*switch_SFefflux_baso_1/(drug_Km_efflux_baso_1+MEM_ILL3_1*fu_mem_1/MILL3_1/drug_molar_mass_1))*MILL3_1.MEM_ILL3_1*fu_mem_1/MILL3_1*
354	MILL4_1.MEM_ILL4_1 -> VillousILL4_1.Villous_ILL4_1
	*((switchVmax_efflux_baso_1==zero)*CLINT_efflux_baso_ILL4_1*switch_SFefflux_baso_1*baso_efflux_factor_ill4+switchVmax_efflux_baso_1*baso_efflux_factor_ill4*phys_Normalized_ESA_baso*phys_BW*basoSurfaceRatio_ILL4*switch_SFefflux_baso_1/(drug_Km_efflux_baso_1+MEM_ILL4_1*fu_mem_1/MILL4_1/drug_molar_mass_1))*MILL4_1.MEM_ILL4_1*fu_mem_1/MILL4_1*

Observables

	Observables	Units
1	AUCPlasma_1 = trapz(time,Plasma_total_1.Plasma_total_uM_1)	micromole/liter*hour
2	CendLiver_1 = Liver_total_1.Liver_tissue_total_uM_1(end)	micromole/liter
3	CmaxLiver_1 = max(Liver_total_1.Liver_tissue_total_uM_1)	micromole/liter
4	AUCLiver_1 = trapz(time, Liver_total_1.Liver_tissue_total_uM_1)	micromole/liter*hour
5	AUCLiver0to24_1 = trapz(time(time<24), Liver_total_1.Liver_tissue_total_uM_1(time<24))	micromole/liter*hour
6	AUCLiver72to96_1 = trapz(time(time>72&time<96), Liver_total_1.Liver_tissue_total_uM_1(time>72&time<96))	micromole/liter*hour
7	finalLiverTissuetotal = Liver_total.Liver_tissue_total_uM(end)	micromole/liter
8	maxLiverTissueTotal = max(Liver_total.Liver_tissue_total_uM)	micromole/liter
9	AUCLiver = trapz(time, Liver_total.Liver_tissue_total_uM)	micromole/liter*hour
10	AUCLiver0to24 = trapz(time(time<24), Liver_total.Liver_tissue_total_uM(time<24))	micromole/liter*hour

	Observables	Units
11	AUCLiver72to96 = trapz(time(time>72&time<96), Liver_total.Liver_tissue_total_uM(time>72&time<96))	micromole/liter*hour
12	AUCPlasma0to24 = trapz(time(time<24), Plasma_total.Plasma_total_uM(time<24))	micromole/liter*hour
13	AUCPlasma0to24_1 = trapz(time(time<24), Plasma_total_1.Plasma_total_uM_1(time<24))	micromole/liter*hour
14	AUCPlasma = trapz(time,Plasma_total.Plasma_total_uM)	micromole/liter*hour
15	CL_1 = max(drug_dose_amount_IV_1/trapz(time,Plasma_total_1.Plasma_total_drug_1))	liter/hour
16	Vss_1 = max(drug_dose_amount_IV_1*trapz(time,time.*Plasma_total_1.Plasma_total_drug_1)/trapz(time,Plasma_total_1.Plasma_total_drug_1)^2)	liter
17	AUCPlasmaTotal0to24 = trapz(time(time<24), Plasma_total_1.Plasma_total_drug_1(time<24))	nanogram/milliliter*hour
18	AUCPlasmaTotal = trapz(time, Plasma_total_1.Plasma_total_drug_1)	nanogram/milliliter*hour
19	Cmax = max(Plasma_total.Plasma_total_drug)	nanogram/milliliter
20	Tmax = max(vertcat(NaN, time(Plasma_total.Plasma_total_drug==max(Plasma_total.Plasma_total_drug))))	hour

Model Equations

ODEs

	ODEs
1	d(Bile_drug)/dt = ((1-switch_liverFlag)*k_Liver_IC_S5_Bile*Liver_IC_S5_drug) + ((1-switch_liverFlag)*k_Liver_IC_S4_Bile*Liver_IC_S4_drug) + ((1-switch_liverFlag)*k_Liver_IC_S3_Bile*Liver_IC_S3_drug) + ((1-switch_liverFlag)*k_Liver_IC_S2_Bile*Liver_IC_S2_drug) + ((1-switch_liverFlag)*k_Liver_IC_S1_Bile*Liver_IC_S1_drug) - (k_transit*Bile_drug) - (drug_k_bile_deg*Bile_drug) + (switch_liverFlag*k_liver_bile*Liver_drug)
2	d(Venous_drug)/dt = 1/Venous*((switch_liverFlag*k_Liver_Venous*Liver_drug) + (k_rest_venous*Rest_drug) + (k_bone_venous*Bone_drug) + (k_skin_venous*Skin_drug) + (k_heart_venous*Heart_drug) + (k_adipos_venous*Adipose_drug) + (k_muscle_venous*Muscle_drug) + (k_brain_venous*Brain_drug) + (k_kidney_venous*Kidney_drug) - (k_venous_lung*Venous_drug) - (k_venous_urine_CLR*Venous_drug) + (k_testes_venous*Testes_drug) - (k_venous_urine_GFR*Venous_drug) + ((1-switch_liverFlag)*k_Liver_EC_S5_Venous*Liver_EC_S5_drug))
3	d(Lung_drug)/dt = 1/Lung*((k_venous_lung*Venous_drug) - (k_lung_artery*Lung_drug))
4	d(Kidney_drug)/dt = 1/Kidney*(-(k_kidney_venous*Kidney_drug) + (k_artery_kidney*Artery_drug))
5	d(Brain_drug)/dt = 1/Brain*(-(k_brain_venous*Brain_drug) + (k_artery_brain*Artery_drug))
6	d(Muscle_drug)/dt = 1/Muscle*(-(k_muscle_venous*Muscle_drug) + (k_artery_muscle*Artery_drug))
7	d(Adipose_drug)/dt = 1/Adipose*(-(k_adipos_venous*Adipose_drug) + (k_artery_adipos*Artery_drug))
8	d(Heart_drug)/dt = 1/Heart*(-(k_heart_venous*Heart_drug) + (k_artery_heart*Artery_drug))
9	d(Skin_drug)/dt = 1/Skin*(-(k_skin_venous*Skin_drug) + (k_artery_skin*Artery_drug))
10	d(Bone_drug)/dt = 1/Bone*(-(k_bone_venous*Bone_drug) + (k_artery_bone*Artery_drug))
11	d(Rest_drug)/dt = 1/Rest*(-(k_rest_venous*Rest_drug) + (k_artery_rest*Artery_drug))
12	d(Artery_drug)/dt = 1/Artery*(-(switch_liverFlag*k_artery_liver*Artery_drug) - (Qmuc_ILL1*Artery_drug) - (k_artery_spleen*Artery_drug) - (k_artery_rest*Artery_drug) - (k_artery_bone*Artery_drug) - (k_artery_skin*Artery_drug) - (k_artery_heart*Artery_drug) - (k_artery_adipos*Artery_drug) - (k_artery_muscle*Artery_drug) - (k_artery_brain*Artery_drug) - (k_artery_kidney*Artery_drug) + (k_lung_artery*Lung_drug) - (k_artery_testes*Artery_drug)

	ODEs
	- (Qmuc_DUO*Artery_drug) - (Qmuc_JEJ1*Artery_drug) - (Qmuc_JEJ2*Artery_drug) - (Qmuc_ILL2*Artery_drug) - (Qmuc_ILL3*Artery_drug) - (Qmuc_ILL4*Artery_drug) - (k_artery_serosa*Artery_drug) - ((1-switch_liverFlag)*k_artery_liver*Artery_drug))
13	d(Spleen_drug)/dt = 1/Spleen*((k_artery_spleen*Artery_drug) - (switch_liverFlag*k_spleen_liver*Spleen_drug) - ((1-switch_liverFlag)*k_spleen_liver*Spleen_drug))
14	d(Liver_EC_S1_drug)/dt = 1/Liver_EC_S1*((k_Liver_IC_S1_Liver_EC_S1*Liver_IC_S1_drug) - (k_Liver_EC_S1_Liver_IC_S1*Liver_EC_S1_drug) - (k_Liver_EC_S1_Liver_EC_S2*Liver_EC_S1_drug) + (k_Liver_IC_S1_Liver_EC_S1_efflux*Liver_IC_S1_drug) + ((1-switch_liverFlag)*k_spleen_liver*Spleen_drug) + ((1-switch_liverFlag)*k_artery_liver*Artery_drug) + ((1-switch_liverFlag)*Villous_DUO*Qmuc_DUO/VillousDUO) + ((1-switch_liverFlag)*Villous_JEJ1*Qmuc_JEJ1/VillousJEJ1) + ((1-switch_liverFlag)*Villous_JEJ2*Qmuc_JEJ2/VillousJEJ2) + ((1-switch_liverFlag)*Villous_ILL1*Qmuc_ILL1/VillousILL1) + ((1-switch_liverFlag)*Villous_ILL2*Qmuc_ILL2/VillousILL2) + ((1-switch_liverFlag)*Villous_ILL3*Qmuc_ILL3/VillousILL3) + ((1-switch_liverFlag)*Villous_ILL4*Qmuc_ILL4/VillousILL4) + ((1-switch_liverFlag)*k_serosa_liver*Serosa_drug))
15	d(Liver_EC_S2_drug)/dt = 1/Liver_EC_S2*((k_Liver_IC_S2_Liver_EC_S2*Liver_IC_S2_drug) - (k_Liver_EC_S2_Liver_IC_S2*Liver_EC_S2_drug) - (k_Liver_EC_S2_Liver_EC_S3*Liver_EC_S2_drug) + (k_Liver_EC_S1_Liver_EC_S2*Liver_EC_S1_drug) + (k_Liver_IC_S2_Liver_EC_S2_efflux*Liver_IC_S2_drug))
16	d(Liver_EC_S3_drug)/dt = 1/Liver_EC_S3*((k_Liver_IC_S3_Liver__EC_S3*Liver_IC_S3_drug) - (k_Liver_EC_S3_Liver_IC_S3*Liver_EC_S3_drug) - (k_Liver_EC_S3_Liver_EC_S4*Liver_EC_S3_drug) + (k_Liver_EC_S2_Liver_EC_S3*Liver_EC_S2_drug) + (k_Liver_IC_S3_Liver_EC_S3_efflux*Liver_IC_S3_drug))
17	d(Liver_EC_S4_drug)/dt = 1/Liver_EC_S4*((k_Liver_IC_S4_Liver_EC_S4*Liver_IC_S4_drug) - (k_Liver_EC_S4_Liver_IC_S4*Liver_EC_S4_drug) - (k_Liver_EC_S4_Liver_EC_S5*Liver_EC_S4_drug) + (k_Liver_EC_S3_Liver_EC_S4*Liver_EC_S3_drug) + (k_Liver_IC_S4_Liver_EC_S4_efflux*Liver_IC_S4_drug))
18	d(Liver_EC_S5_drug)/dt = 1/Liver_EC_S5*((k_Liver_IC_S5_Liver_EC_S5*Liver_IC_S5_drug) - (k_Liver_EC_S5_Liver_IC_S5*Liver_EC_S5_drug) + (k_Liver_EC_S4_Liver_EC_S5*Liver_EC_S4_drug) + (k_Liver_IC_S5_Liver_EC_S5_efflux*Liver_IC_S5_drug) - ((1-switch_liverFlag)*k_Liver_EC_S5_Venous*Liver_EC_S5_drug))
19	d(Liver_IC_S5_drug)/dt = 1/Liver_IC_S5*(-((1-switch_liverFlag)*k_Liver_IC_S5_Bile*Liver_IC_S5_drug) - ((1-switch_liverFlag)*k_Liver_IC_S5_Metabolites*Liver_IC_S5_drug) - (k_Liver_IC_S5_Liver_EC_S5*Liver_IC_S5_drug) + (k_Liver_EC_S5_Liver_IC_S5*Liver_EC_S5_drug) - (k_Liver_IC_S5_Liver_EC_S5_efflux*Liver_IC_S5_drug))
20	d(Liver_IC_S3_drug)/dt = 1/Liver_IC_S3*(-((1-switch_liverFlag)*k_Liver_IC_S3_Metabolites*Liver_IC_S3_drug) - ((1-switch_liverFlag)*k_Liver_IC_S3_Bile*Liver_IC_S3_drug) - (k_Liver_IC_S3_Liver__EC_S3*Liver_IC_S3_drug) + (k_Liver_EC_S3_Liver_IC_S3*Liver_EC_S3_drug) - (k_Liver_IC_S3_Liver_EC_S3_efflux*Liver_IC_S3_drug))
21	d(Liver_IC_S1_drug)/dt = 1/Liver_IC_S1*(-((1-switch_liverFlag)*k_Liver_IC_S1_Metabolites*Liver_IC_S1_drug) - ((1-switch_liverFlag)*k_Liver_IC_S1_Bile*Liver_IC_S1_drug) - (k_Liver_IC_S1_Liver_EC_S1*Liver_IC_S1_drug) + (k_Liver_EC_S1_Liver_IC_S1*Liver_EC_S1_drug) - (k_Liver_IC_S1_Liver_EC_S1_efflux*Liver_IC_S1_drug))
22	d(Liver_IC_S2_drug)/dt = 1/Liver_IC_S2*(-((1-switch_liverFlag)*k_Liver_IC_S2_Metabolites*Liver_IC_S2_drug) - ((1-switch_liverFlag)*k_Liver_IC_S2_Bile*Liver_IC_S2_drug) - (k_Liver_IC_S2_Liver_EC_S2*Liver_IC_S2_drug) + (k_Liver_EC_S2_Liver_IC_S2*Liver_EC_S2_drug) - (k_Liver_IC_S2_Liver_EC_S2_efflux*Liver_IC_S2_drug))
23	d(Metabolites_drug)/dt = ((1-switch_liverFlag)*k_Liver_IC_S5_Metabolites*Liver_IC_S5_drug) + ((1-switch_liverFlag)*k_Liver_IC_S4_Metabolites*Liver_IC_S4_drug) + ((1-switch_liverFlag)*k_Liver_IC_S3_Metabolites*Liver_IC_S3_drug) + ((1-switch_liverFlag)*k_Liver_IC_S2_Metabolites*Liver_IC_S2_drug) + ((1-switch_liverFlag)*k_Liver_IC_S1_Metabolites*Liver_IC_S1_drug) + (switch_liverFlag*k_liver_metabolites*Liver_drug)
24	d(Testes_drug)/dt = 1/Testes*((k_artery_testes*Artery_drug) - (k_testes_venous*Testes_drug))
25	d(Urine_drug)/dt = (k_venous_urine_CLR*Venous_drug) + (k_venous_urine_GFR*Venous_drug)
26	d(Liver_IC_S4_drug)/dt = 1/Liver_IC_S4*(-((1-switch_liverFlag)*k_Liver_IC_S4_Metabolites*Liver_IC_S4_drug) - ((1-switch_liverFlag)*k_Liver_IC_S4_Bile*Liver_IC_S4_drug) - (k_Liver_IC_S4_Liver_EC_S4*Liver_IC_S4_drug) + (k_Liver_EC_S4_Liver_IC_S4*Liver_EC_S4_drug) - (k_Liver_IC_S4_Liver_EC_S4_efflux*Liver_IC_S4_drug))
27	d(X_STOMACH_SOLID)/dt = -(X_STOMACH_SOLID/TSTOMACH) - (KD*X_STOMACH_SOLID*(SOLIF_STOMACH-X_STOMACH_DISS/STOMACH))
28	d(X_STOMACH_DISS)/dt = -(X_STOMACH_DISS/TSTOMACH) + (KD*X_STOMACH_SOLID*(SOLIF_STOMACH-X_STOMACH_DISS/STOMACH))
29	d(X_DUO_SOLID)/dt = (X_STOMACH_SOLID/TSTOMACH) - (X_DUO_SOLID/TDUO) - (KD*X_DUO_SOLID*(SOLIF_DUO-X_DUO_DISS/VDUO))

	ODEs
30	d(X_DUO_DISS)/dt = (k_transit*Bile_drug) + (X_STOMACH_DISS/TSTOMACH) + (KD*X_DUO_SOLID*(SOLIF_DUO-X_DUO_DISS/VDUO)) - (X_DUO_DISS/TDUO) - ((DIFF_duo*NI_DUO*switch_SFdiffapi*X_DUO_DISS)/VDUO) + (((switchVmax_efflux==zero)*CLINT_efflux_DUO*efflux_factor_duo*switch_SFefflux+switchVmax_efflux*phys_Normalized_ESA*phys_BW*surfaceRatio_DUO*efflux_factor_duo*switch_SFefflux/(drug_Km_efflux+MEM_DUO*fu_mem/MDUO/drug_molar_mass))*MEM_DUO*fu_mem/MDUO) - (((switchVmax_influx==zero)*CLINT_influx_DUO*influx_factor_duo*switch_SFinflux+switchVmax_influx*phys_Normalized_ESA*phys_BW*surfaceRatio_DUO*influx_factor_duo/(drug_Km_influx+X_DUO_DISS/VDUO/drug_molar_mass))*X_DUO_DISS/VDUO) + (DIFF_duo*switch_SFdiffapi*MEM_DUO*fu_mem/MDUO)
31	d(X_JEJ1_SOLID)/dt = (X_DUO_SOLID/TDUO) - (X_JEJ1_SOLID/TJEJ1) - (KD*X_JEJ1_SOLID*(SOLIF_JEJ1-X_JEJ1_DISS/VJEJ1))
32	d(X_JEJ1_DISS)/dt = (X_DUO_DISS/TDUO) + (KD*X_JEJ1_SOLID*(SOLIF_JEJ1-X_JEJ1_DISS/VJEJ1)) - (X_JEJ1_DISS/TJEJ1) - ((DIFF_jej1*NI_JEJ1*switch_SFdiffapi*X_JEJ1_DISS)/VJEJ1) + (((switchVmax_efflux==zero)*CLINT_efflux_JEJ1*efflux_factor_jej1*switch_SFefflux+switchVmax_efflux*phys_Normalized_ESA*phys_BW*surfaceRatio_JEJ1*efflux_factor_jej1*switch_SFefflux/(drug_Km_efflux+MEM_JEJ1*fu_mem/MJEJ1/drug_molar_mass))*MEM_JEJ1*fu_mem/MJEJ1) - (((switchVmax_influx==zero)*CLINT_influx_JEJ1*influx_factor_jej1*switch_SFinflux+switchVmax_influx*phys_Normalized_ESA*phys_BW*surfaceRatio_JEJ1*influx_factor_jej1/(drug_Km_influx+X_JEJ1_DISS/VJEJ1/drug_molar_mass))*X_JEJ1_DISS/VJEJ1) + (DIFF_jej1*switch_SFdiffapi*MEM_JEJ1*fu_mem/MJEJ1)
33	d(MEM_DUO)/dt = ((DIFF_duo*NI_DUO*switch_SFdiffapi*X_DUO_DISS)/VDUO) - (((switchVmax_efflux==zero)*CLINT_efflux_DUO*efflux_factor_duo*switch_SFefflux+switchVmax_efflux*phys_Normalized_ESA*phys_BW*surfaceRatio_DUO*efflux_factor_duo*switch_SFefflux/(drug_Km_efflux+MEM_DUO*fu_mem/MDUO/drug_molar_mass))*MEM_DUO*fu_mem/MDUO) + (((switchVmax_influx==zero)*CLINT_influx_DUO*influx_factor_duo*switch_SFinflux+switchVmax_influx*phys_Normalized_ESA*phys_BW*surfaceRatio_DUO*influx_factor_duo/(drug_Km_influx+X_DUO_DISS/VDUO/drug_molar_mass))*X_DUO_DISS/VDUO) - ((CLINT_metabolism*metabolism_factor_duo*switch_SFgutmet*MEM_DUO*fu_mem)/MDUO) - (DIFF_BASO_duo*switch_SFdiffbaso*MEM_DUO*fu_mem/MDUO) + (DIFF_BASO_duo*switch_SFdiffbaso*Villous_DUO*fu_blood/VillousDUO) - (DIFF_duo*switch_SFdiffapi*MEM_DUO*fu_mem/MDUO) + (CLINT_influx_baso_DUO*influx_factor_duo_baso*switch_SFinflux*Villous_DUO/VillousDUO) - (((switchVmax_efflux_baso==zero)*CLINT_efflux_baso_DUO*switch_SFefflux_baso*baso_efflux_factor_duo+switchVmax_efflux_baso*baso_efflux_factor_duo*phys_Normalized_ESA_baso*phys_BW*basoSurfaceRatio_DUO*switch_SFefflux_baso/(drug_Km_efflux_baso+MEM_DUO*fu_mem/MDUO/drug_molar_mass))*MEM_DUO*fu_mem/MDUO)
34	d(MEM_JEJ1)/dt = ((DIFF_jej1*NI_JEJ1*switch_SFdiffapi*X_JEJ1_DISS)/VJEJ1) - (((switchVmax_efflux==zero)*CLINT_efflux_JEJ1*efflux_factor_jej1*switch_SFefflux+switchVmax_efflux*phys_Normalized_ESA*phys_BW*surfaceRatio_JEJ1*efflux_factor_jej1*switch_SFefflux/(drug_Km_efflux+MEM_JEJ1*fu_mem/MJEJ1/drug_molar_mass))*MEM_JEJ1*fu_mem/MJEJ1) + (((switchVmax_influx==zero)*CLINT_influx_JEJ1*influx_factor_jej1*switch_SFinflux+switchVmax_influx*phys_Normalized_ESA*phys_BW*surfaceRatio_JEJ1*influx_factor_jej1/(drug_Km_influx+X_JEJ1_DISS/VJEJ1/drug_molar_mass))*X_JEJ1_DISS/VJEJ1) - ((CLINT_metabolism*metabolism_factor_jej1*switch_SFgutmet*MEM_JEJ1*fu_mem)/MJEJ1) - (DIFF_BASO_jej1*switch_SFdiffbaso*MEM_JEJ1*fu_mem/MJEJ1) + (DIFF_BASO_jej1*switch_SFdiffbaso*Villous_JEJ1*fu_blood/VillousJEJ1) - (DIFF_jej1*switch_SFdiffapi*MEM_JEJ1*fu_mem/MJEJ1) + (CLINT_influx_baso_JEJ1*influx_factor_jej1_baso*switch_SFinflux*Villous_JEJ1/VillousJEJ1) - (((switchVmax_efflux_baso==zero)*CLINT_efflux_baso_JEJ1*switch_SFefflux_baso*baso_efflux_factor_jej1+switchVmax_efflux_baso*baso_efflux_factor_jej1*phys_Normalized_ESA_baso*phys_BW*basoSurfaceRatio_JEJ1*switch_SFefflux_baso/(drug_Km_efflux_baso+MEM_JEJ1*fu_mem/MJEJ1/drug_molar_mass))*MEM_JEJ1*fu_mem/MJEJ1)
35	d(X_JEJ2_SOLID)/dt = (X_JEJ1_SOLID/TJEJ1) - (X_JEJ2_SOLID/TJEJ2) - (KD*X_JEJ2_SOLID*(SOLIF_JEJ2-X_JEJ2_DISS/VJEJ2))
36	d(X_JEJ2_DISS)/dt = (X_JEJ1_DISS/TJEJ1) + (KD*X_JEJ2_SOLID*(SOLIF_JEJ2-X_JEJ2_DISS/VJEJ2)) - (X_JEJ2_DISS/TJEJ2) - ((DIFF_jej2*NI_JEJ2*switch_SFdiffapi*X_JEJ2_DISS)/VJEJ2) + (((switchVmax_efflux==zero)*CLINT_efflux_JEJ2*efflux_factor_jej2*switch_SFefflux+switchVmax_efflux*phys_Normalized_ESA*phys_BW*surfaceRatio_JEJ2*efflux_factor_jej2*switch_SFefflux/(drug_Km_efflux+MEM_JEJ2*fu_mem/MJEJ2/drug_molar_mass))*MEM_JEJ2*fu_mem/MJEJ2) - (((switchVmax_influx==zero)*CLINT_influx_JEJ2*influx_factor_jej2*switch_SFinflux+switchVmax_influx*phys_Normalized_ESA*phys_BW*surfaceRatio_JEJ2*influx_factor_jej2/(drug_Km_influx+X_JEJ2_DISS/VJEJ2/drug_molar_mass))*X_JEJ2_DISS/VJEJ2) + (DIFF_jej2*switch_SFdiffapi*MEM_JEJ2*fu_mem/MJEJ2)
37	d(MEM_JEJ2)/dt = ((DIFF_jej2*NI_JEJ2*switch_SFdiffapi*X_JEJ2_DISS)/VJEJ2) - (((switchVmax_efflux==zero)*CLINT_efflux_JEJ2*efflux_factor_jej2*switch_SFefflux+switchVmax_efflux*phys_Normalized_ESA*phys_BW*surfaceRatio_JEJ2*efflux_factor_jej2*switch_SFefflux/(drug_Km_efflux+MEM_JEJ2*fu_mem/MJEJ2/drug_molar_mass))*MEM_JEJ2*fu_mem/MJEJ2) + (((switchVmax_influx==zero)*CLINT_influx_JEJ2*influx_factor_jej2*switch_SFinflux+switchVmax_influx*phys_Normalized

	ODEs
	_ESA*phys_BW*surfaceRatio_JEJ2*influx_factor_jej2/(drug_Km_influx+X_JEJ2_DISS/VJEJ2/drug_molar_mass))*X_JEJ2_DISS/VJEJ2) - ((CLINT_metabolism*metabolism_factor_jej2*switch_SFgutmet*MEM_JEJ2*fu_mem)/MJEJ2) - (DIFF_BASO_jej2*switch_SFdiffbaso*MEM_JEJ2*fu_mem/MJEJ2) + (DIFF_BASO_jej2*switch_SFdiffbaso*Villous_JEJ2*fu_blood/VillousJEJ2) - (DIFF_jej2*switch_SFdiffapi*MEM_JEJ2*fu_mem/MJEJ2) + (CLINT_influx_baso_JEJ2*influx_factor_jej2_baso*switch_SFinflux*Villous_JEJ2/VillousJEJ2) - (((switchVmax_efflux_baso==zero)*CLINT_efflux_baso_JEJ2*switch_SFefflux_baso*baso_efflux_factor_jej2+switchVmax_efflux_baso*baso_efflux_factor_jej2*phys_Normalized_ESA_baso*phys_BW*basoSurfaceRatio_JEJ2*switch_SFefflux_baso/(drug_Km_efflux_baso+MEM_JEJ2*fu_mem/MJEJ2/drug_molar_mass))*MEM_JEJ2*fu_mem/MJEJ2)
38	d(X_ILL1_SOLID)/dt = (X_JEJ2_SOLID/TJEJ2) - (X_ILL1_SOLID/TILL1) - (KD*X_ILL1_SOLID*(SOLIF_ILL1-X_ILL1_DISS/VILL1))
39	d(X_ILL1_DISS)/dt = (X_JEJ2_DISS/TJEJ2) + (KD*X_ILL1_SOLID*(SOLIF_ILL1-X_ILL1_DISS/VILL1)) - (X_ILL1_DISS/TILL1) - ((DIFF_ill1*NI_ILL1*switch_SFdiffapi*X_ILL1_DISS)/VILL1) + (((switchVmax_efflux==zero)*CLINT_efflux_ILL1*efflux_factor_ill1*switch_SFefflux+switchVmax_efflux*phys_Normalized_ESA*phys_BW*surfaceRatio_ILL1*efflux_factor_ill1*switch_SFefflux/(drug_Km_efflux+MEM_ILL1*fu_mem/MILL1/drug_molar_mass))*MEM_ILL1*fu_mem/MILL1) - (((switchVmax_influx==zero)*CLINT_influx_ILL1*influx_factor_ill1*switch_SFinflux+switchVmax_influx*phys_Normalized_ESA*phys_BW*surfaceRatio_ILL1*influx_factor_ill1/(drug_Km_influx+X_ILL1_DISS/VILL1/drug_molar_mass))*X_ILL1_DISS/VILL1) + (DIFF_ill1*switch_SFdiffapi*MEM_ILL1*fu_mem/MILL1)
40	d(MEM_ILL1)/dt = ((DIFF_ill1*NI_ILL1*switch_SFdiffapi*X_ILL1_DISS)/VILL1) - (((switchVmax_efflux==zero)*CLINT_efflux_ILL1*efflux_factor_ill1*switch_SFefflux+switchVmax_efflux*phys_Normalized_ESA*phys_BW*surfaceRatio_ILL1*efflux_factor_ill1*switch_SFefflux/(drug_Km_efflux+MEM_ILL1*fu_mem/MILL1/drug_molar_mass))*MEM_ILL1*fu_mem/MILL1) + (((switchVmax_influx==zero)*CLINT_influx_ILL1*influx_factor_ill1*switch_SFinflux+switchVmax_influx*phys_Normalized_ESA*phys_BW*surfaceRatio_ILL1*influx_factor_ill1/(drug_Km_influx+X_ILL1_DISS/VILL1/drug_molar_mass))*X_ILL1_DISS/VILL1) - ((CLINT_metabolism*metabolism_factor_ill1*switch_SFgutmet*MEM_ILL1*fu_mem)/MILL1) - (DIFF_BASO_ill1*switch_SFdiffbaso*MEM_ILL1*fu_mem/MILL1) + (DIFF_BASO_ill1*switch_SFdiffbaso*Villous_ILL1*fu_blood/VillousILL1) - (DIFF_ill1*switch_SFdiffapi*MEM_ILL1*fu_mem/MILL1) + (CLINT_influx_baso_ILL1*influx_factor_ill1_baso*switch_SFinflux*Villous_ILL1/VillousILL1) - (((switchVmax_efflux_baso==zero)*CLINT_efflux_baso_ILL1*switch_SFefflux_baso*baso_efflux_factor_ill1+switchVmax_efflux_baso*baso_efflux_factor_ill1*phys_Normalized_ESA_baso*phys_BW*basoSurfaceRatio_ILL1*switch_SFefflux_baso/(drug_Km_efflux_baso+MEM_ILL1*fu_mem/MILL1/drug_molar_mass))*MEM_ILL1*fu_mem/MILL1)
41	d(X_ILL2_SOLID)/dt = (X_ILL1_SOLID/TILL1) - (X_ILL2_SOLID/TILL2) - (KD*X_ILL2_SOLID*(SOLIF_ILL2-X_ILL2_DISS/VILL2))
42	d(X_ILL2_DISS)/dt = (X_ILL1_DISS/TILL1) + (KD*X_ILL2_SOLID*(SOLIF_ILL2-X_ILL2_DISS/VILL2)) - (X_ILL2_DISS/TILL2) - ((DIFF_ill2*NI_ILL2*switch_SFdiffapi*X_ILL2_DISS)/VILL2) + (((switchVmax_efflux==zero)*CLINT_efflux_ILL2*efflux_factor_ill2*switch_SFefflux+switchVmax_efflux*phys_Normalized_ESA*phys_BW*surfaceRatio_ILL2*efflux_factor_ill2*switch_SFefflux/(drug_Km_efflux+MEM_ILL2*fu_mem/MILL2/drug_molar_mass))*MEM_ILL2*fu_mem/MILL2) - (((switchVmax_influx==zero)*CLINT_influx_ILL2*influx_factor_ill2*switch_SFinflux+switchVmax_influx*phys_Normalized_ESA*phys_BW*surfaceRatio_ILL2*influx_factor_ill2/(drug_Km_influx+X_ILL2_DISS/VILL2/drug_molar_mass))*X_ILL2_DISS/VILL2) + (DIFF_ill2*switch_SFdiffapi*MEM_ILL2*fu_mem/MILL2)
43	d(MEM_ILL2)/dt = ((DIFF_ill2*NI_ILL2*switch_SFdiffapi*X_ILL2_DISS)/VILL2) - (((switchVmax_efflux==zero)*CLINT_efflux_ILL2*efflux_factor_ill2*switch_SFefflux+switchVmax_efflux*phys_Normalized_ESA*phys_BW*surfaceRatio_ILL2*efflux_factor_ill2*switch_SFefflux/(drug_Km_efflux+MEM_ILL2*fu_mem/MILL2/drug_molar_mass))*MEM_ILL2*fu_mem/MILL2) + (((switchVmax_influx==zero)*CLINT_influx_ILL2*influx_factor_ill2*switch_SFinflux+switchVmax_influx*phys_Normalized_ESA*phys_BW*surfaceRatio_ILL2*influx_factor_ill2/(drug_Km_influx+X_ILL2_DISS/VILL2/drug_molar_mass))*X_ILL2_DISS/VILL2) - ((CLINT_metabolism*metabolism_factor_ill2*switch_SFgutmet*MEM_ILL2*fu_mem)/MILL2) + (DIFF_BASO_ill2*switch_SFdiffbaso*Villous_ILL2*fu_blood/VillousILL2) - (DIFF_BASO_ill2*switch_SFdiffbaso*MEM_ILL2*fu_mem/MILL2) - (DIFF_ill2*switch_SFdiffapi*MEM_ILL2*fu_mem/MILL2) + (CLINT_influx_baso_ILL2*influx_factor_ill2_baso*switch_SFinflux*Villous_ILL2/VillousILL2) - (((switchVmax_efflux_baso==zero)*CLINT_efflux_baso_ILL2*switch_SFefflux_baso*baso_efflux_factor_ill2+switchVmax_efflux_baso*baso_efflux_factor_ill2*phys_Normalized_ESA_baso*phys_BW*basoSurfaceRatio_ILL2*switch_SFefflux_baso/(drug_Km_efflux_baso+MEM_ILL2*fu_mem/MILL2/drug_molar_mass))*MEM_ILL2*fu_mem/MILL2)
44	d(X_ILL3_SOLID)/dt = (X_ILL2_SOLID/TILL2) - (X_ILL3_SOLID/TILL3) - (KD*X_ILL3_SOLID*(SOLIF_ILL3-X_ILL3_DISS/VILL3))

	ODEs
45	d(X_ILL3_DISS)/dt = (X_ILL2_DISS/TILL2) + (KD*X_ILL3_SOLID*(SOLIF_ILL3-X_ILL3_DISS/VILL3)) - (X_ILL3_DISS/TILL3) - ((DIFF_ill3*NI_ILL3*switch_SFdiffapi*X_ILL3_DISS)/VILL3) + (((switchVmax_efflux==zero)*CLINT_efflux_ILL3*efflux_factor_ill3*switch_SFefflux+switchVmax_efflux*phys_Normalized_ESA*phys_BW*surfaceRatio_ILL3*efflux_factor_ill3*switch_SFefflux/(drug_Km_efflux+MEM_ILL3*fu_mem/MILL3/drug_molar_mass))*MEM_ILL3*fu_mem/MILL3) - (((switchVmax_influx==zero)*CLINT_influx_ILL3*influx_factor_ill3*switch_SFinflux+switchVmax_influx*phys_Normalized_ESA*phys_BW*surfaceRatio_ILL3*influx_factor_ill3/(drug_Km_influx+X_ILL3_DISS/VILL3/drug_molar_mass))*X_ILL3_DISS/VILL3) + (DIFF_ill3*switch_SFdiffapi*MEM_ILL3*fu_mem/MILL3)
46	d(MEM_ILL3)/dt = ((DIFF_ill3*NI_ILL3*switch_SFdiffapi*X_ILL3_DISS)/VILL3) - (((switchVmax_efflux==zero)*CLINT_efflux_ILL3*efflux_factor_ill3*switch_SFefflux+switchVmax_efflux*phys_Normalized_ESA*phys_BW*surfaceRatio_ILL3*efflux_factor_ill3*switch_SFefflux/(drug_Km_efflux+MEM_ILL3*fu_mem/MILL3/drug_molar_mass))*MEM_ILL3*fu_mem/MILL3) + (((switchVmax_influx==zero)*CLINT_influx_ILL3*influx_factor_ill3*switch_SFinflux+switchVmax_influx*phys_Normalized_ESA*phys_BW*surfaceRatio_ILL3*influx_factor_ill3/(drug_Km_influx+X_ILL3_DISS/VILL3/drug_molar_mass))*X_ILL3_DISS/VILL3) - ((CLINT_metabolism*metabolism_factor_ill3*switch_SFgutmet*MEM_ILL3*fu_mem)/MILL3) + (DIFF_BASO_ill3*switch_SFdiffbaso*Villous_ILL3*fu_blood/VillousILL3) - (DIFF_BASO_ill3*switch_SFdiffbaso*MEM_ILL3*fu_mem/MILL3) - (DIFF_ill3*switch_SFdiffapi*MEM_ILL3*fu_mem/MILL3) + (CLINT_influx_baso_ILL3*influx_factor_ill3_baso*switch_SFinflux*Villous_ILL3/VillousILL3) - (((switchVmax_efflux_baso==zero)*CLINT_efflux_baso_ILL3*switch_SFefflux_baso*baso_efflux_factor_ill3+switchVmax_efflux_baso*baso_efflux_factor_ill3*phys_Normalized_ESA_baso*phys_BW*basoSurfaceRatio_ILL3*switch_SFefflux_baso/(drug_Km_efflux_baso+MEM_ILL3*fu_mem/MILL3/drug_molar_mass))*MEM_ILL3*fu_mem/MILL3)
47	d(X_ILL4_SOLID)/dt = (X_ILL3_SOLID/TILL3) - (X_ILL4_SOLID/TILL4) - (KD*X_ILL4_SOLID*(SOLIF_ILL4-X_ILL4_DISS/VILL4))
48	d(X_ILL4_DISS)/dt = (X_ILL3_DISS/TILL3) + (KD*X_ILL4_SOLID*(SOLIF_ILL4-X_ILL4_DISS/VILL4)) - (X_ILL4_DISS/TILL4) - ((DIFF_ill4*NI_ILL4*switch_SFdiffapi*X_ILL4_DISS)/VILL4) + (((switchVmax_efflux==zero)*CLINT_efflux_ILL4*efflux_factor_ill4*switch_SFefflux+switchVmax_efflux*phys_Normalized_ESA*phys_BW*surfaceRatio_ILL4*efflux_factor_ill4*switch_SFefflux/(drug_Km_efflux+MEM_ILL4*fu_mem/MILL4/drug_molar_mass))*MEM_ILL4*fu_mem/MILL4) - (((switchVmax_influx==zero)*CLINT_influx_ILL4*influx_factor_ill4*switch_SFinflux+switchVmax_influx*phys_Normalized_ESA*phys_BW*surfaceRatio_ILL4*influx_factor_ill4/(drug_Km_influx+X_ILL4_DISS/VILL4/drug_molar_mass))*X_ILL4_DISS/VILL4) + (DIFF_ill4*switch_SFdiffapi*MEM_ILL4*fu_mem/MILL4)
49	d(MEM_ILL4)/dt = ((DIFF_ill4*NI_ILL4*switch_SFdiffapi*X_ILL4_DISS)/VILL4) - (((switchVmax_efflux==zero)*CLINT_efflux_ILL4*efflux_factor_ill4*switch_SFefflux+switchVmax_efflux*phys_Normalized_ESA*phys_BW*surfaceRatio_ILL4*efflux_factor_ill4*switch_SFefflux/(drug_Km_efflux+MEM_ILL4*fu_mem/MILL4/drug_molar_mass))*MEM_ILL4*fu_mem/MILL4) + (((switchVmax_influx==zero)*CLINT_influx_ILL4*influx_factor_ill4*switch_SFinflux+switchVmax_influx*phys_Normalized_ESA*phys_BW*surfaceRatio_ILL4*influx_factor_ill4/(drug_Km_influx+X_ILL4_DISS/VILL4/drug_molar_mass))*X_ILL4_DISS/VILL4) - ((CLINT_metabolism*metabolism_factor_ill4*switch_SFgutmet*MEM_ILL4*fu_mem)/MILL4) - (DIFF_ill4*switch_SFdiffapi*MEM_ILL4*fu_mem/MILL4) + (DIFF_BASO_ill4*switch_SFdiffbaso*Villous_ILL4*fu_blood/VillousILL4) - (DIFF_BASO_ill4*switch_SFdiffbaso*MEM_ILL4*fu_mem/MILL4) + (CLINT_influx_baso_ILL4*influx_factor_ill4_baso*switch_SFinflux*Villous_ILL4/VillousILL4) - (((switchVmax_efflux_baso==zero)*CLINT_efflux_baso_ILL4*switch_SFefflux_baso*baso_efflux_factor_ill4+switchVmax_efflux_baso*baso_efflux_factor_ill4*phys_Normalized_ESA_baso*phys_BW*basoSurfaceRatio_ILL4*switch_SFefflux_baso/(drug_Km_efflux_baso+MEM_ILL4*fu_mem/MILL4/drug_molar_mass))*MEM_ILL4*fu_mem/MILL4)
50	d(X_CECUM_SOLID)/dt = (X_ILL4_SOLID/TILL4)
51	d(X_CECUM_DISS)/dt = (X_ILL4_DISS/TILL4)
52	d(Villous_DUO)/dt = -(switch_liverFlag*Villous_DUO*Qmuc_DUO/VillousDUO) + (Qmuc_DUO*Artery_drug) + (DIFF_BASO_duo*switch_SFdiffbaso*MEM_DUO*fu_mem/MDUO) - (DIFF_BASO_duo*switch_SFdiffbaso*Villous_DUO*fu_blood/VillousDUO) - (CLINT_influx_baso_DUO*influx_factor_duo_baso*switch_SFinflux*Villous_DUO/VillousDUO) - ((1-switch_liverFlag)*Villous_DUO*Qmuc_DUO/VillousDUO) + (((switchVmax_efflux_baso==zero)*CLINT_efflux_baso_DUO*switch_SFefflux_baso*baso_efflux_factor_duo+switchVmax_efflux_baso*baso_efflux_factor_duo*phys_Normalized_ESA_baso*phys_BW*basoSurfaceRatio_DUO*switch_SFefflux_baso/(drug_Km_efflux_baso+MEM_DUO*fu_mem/MDUO/drug_molar_mass))*MEM_DUO*fu_mem/MDUO)
53	d(Villous_JEJ2)/dt = -(switch_liverFlag*Villous_JEJ2*Qmuc_JEJ2/VillousJEJ2) + (Qmuc_JEJ2*Artery_drug) + (DIFF_BASO_jej2*switch_SFdiffbaso*MEM_JEJ2*fu_mem/MJEJ2) - (DIFF_BASO_jej2*switch_SFdiffbaso*Villous_JEJ2*fu_blood/VillousJEJ2) - (CLINT_influx_baso_JEJ2*influx_factor_jej2_baso*switch_SFinflux*Villous_JEJ2/VillousJEJ2) - ((1-

	ODEs
	switch_liverFlag)*Villous_JEJ2*Qmuc_JEJ2/VillousJEJ2) + (((switchVmax_efflux_baso==zero)*CLINT_efflux_baso_JEJ2*switch_SFefflux_baso*baso_efflux_factor_jej2+switchVmax_efflux_baso*baso_efflux_factor_jej2*phys_Normalized_ESA_baso*phys_BW*basoSurfaceRatio_JEJ2*switch_SFefflux_baso/(drug_Km_efflux_baso+MEM_JEJ2*fu_mem/MJEJ2/drug_molar_mass))*MEM_JEJ2*fu_mem/MJEJ2)
54	d(Villous_ILL1)/dt = (Qmuc_ILL1*Artery_drug) - (switch_liverFlag*Villous_ILL1*Qmuc_ILL1/VillousILL1) + (DIFF_BASO_ill1*switch_SFdiffbaso*MEM_ILL1*fu_mem/MILL1) - (DIFF_BASO_ill1*switch_SFdiffbaso*Villous_ILL1*fu_blood/VillousILL1) - (CLINT_influx_baso_ILL1*influx_factor_ill1_baso*switch_SFinflux*Villous_ILL1/VillousILL1) - ((1-switch_liverFlag)*Villous_ILL1*Qmuc_ILL1/VillousILL1) + (((switchVmax_efflux_baso==zero)*CLINT_efflux_baso_ILL1*switch_SFefflux_baso*baso_efflux_factor_ill1+switchVmax_efflux_baso*baso_efflux_factor_ill1*phys_Normalized_ESA_baso*phys_BW*basoSurfaceRatio_ILL1*switch_SFefflux_baso/(drug_Km_efflux_baso+MEM_ILL1*fu_mem/MILL1/drug_molar_mass))*MEM_ILL1*fu_mem/MILL1)
55	d(Villous_ILL2)/dt = -(switch_liverFlag*Villous_ILL2*Qmuc_ILL2/VillousILL2) + (Qmuc_ILL2*Artery_drug) - (DIFF_BASO_ill2*switch_SFdiffbaso*Villous_ILL2*fu_blood/VillousILL2) + (DIFF_BASO_ill2*switch_SFdiffbaso*MEM_ILL2*fu_mem/MILL2) - (CLINT_influx_baso_ILL2*influx_factor_ill2_baso*switch_SFinflux*Villous_ILL2/VillousILL2) - ((1-switch_liverFlag)*Villous_ILL2*Qmuc_ILL2/VillousILL2) + (((switchVmax_efflux_baso==zero)*CLINT_efflux_baso_ILL2*switch_SFefflux_baso*baso_efflux_factor_ill2+switchVmax_efflux_baso*baso_efflux_factor_ill2*phys_Normalized_ESA_baso*phys_BW*basoSurfaceRatio_ILL2*switch_SFefflux_baso/(drug_Km_efflux_baso+MEM_ILL2*fu_mem/MILL2/drug_molar_mass))*MEM_ILL2*fu_mem/MILL2)
56	d(Villous_ILL3)/dt = -(switch_liverFlag*Villous_ILL3*Qmuc_ILL3/VillousILL3) + (Qmuc_ILL3*Artery_drug) - (DIFF_BASO_ill3*switch_SFdiffbaso*Villous_ILL3*fu_blood/VillousILL3) + (DIFF_BASO_ill3*switch_SFdiffbaso*MEM_ILL3*fu_mem/MILL3) - (CLINT_influx_baso_ILL3*influx_factor_ill3_baso*switch_SFinflux*Villous_ILL3/VillousILL3) - ((1-switch_liverFlag)*Villous_ILL3*Qmuc_ILL3/VillousILL3) + (((switchVmax_efflux_baso==zero)*CLINT_efflux_baso_ILL3*switch_SFefflux_baso*baso_efflux_factor_ill3+switchVmax_efflux_baso*baso_efflux_factor_ill3*phys_Normalized_ESA_baso*phys_BW*basoSurfaceRatio_ILL3*switch_SFefflux_baso/(drug_Km_efflux_baso+MEM_ILL3*fu_mem/MILL3/drug_molar_mass))*MEM_ILL3*fu_mem/MILL3)
57	d(Villous_ILL4)/dt = -(switch_liverFlag*Villous_ILL4*Qmuc_ILL4/VillousILL4) + (Qmuc_ILL4*Artery_drug) - (DIFF_BASO_ill4*switch_SFdiffbaso*Villous_ILL4*fu_blood/VillousILL4) + (DIFF_BASO_ill4*switch_SFdiffbaso*MEM_ILL4*fu_mem/MILL4) - (CLINT_influx_baso_ILL4*influx_factor_ill4_baso*switch_SFinflux*Villous_ILL4/VillousILL4) - ((1-switch_liverFlag)*Villous_ILL4*Qmuc_ILL4/VillousILL4) + (((switchVmax_efflux_baso==zero)*CLINT_efflux_baso_ILL4*switch_SFefflux_baso*baso_efflux_factor_ill4+switchVmax_efflux_baso*baso_efflux_factor_ill4*phys_Normalized_ESA_baso*phys_BW*basoSurfaceRatio_ILL4*switch_SFefflux_baso/(drug_Km_efflux_baso+MEM_ILL4*fu_mem/MILL4/drug_molar_mass))*MEM_ILL4*fu_mem/MILL4)
58	d(Villous_JEJ1)/dt = -(switch_liverFlag*Villous_JEJ1*Qmuc_JEJ1/VillousJEJ1) + (Qmuc_JEJ1*Artery_drug) + (DIFF_BASO_jej1*switch_SFdiffbaso*MEM_JEJ1*fu_mem/MJEJ1) - (DIFF_BASO_jej1*switch_SFdiffbaso*Villous_JEJ1*fu_blood/VillousJEJ1) - (CLINT_influx_baso_JEJ1*influx_factor_jej1_baso*switch_SFinflux*Villous_JEJ1/VillousJEJ1) - ((1-switch_liverFlag)*Villous_JEJ1*Qmuc_JEJ1/VillousJEJ1) + (((switchVmax_efflux_baso==zero)*CLINT_efflux_baso_JEJ1*switch_SFefflux_baso*baso_efflux_factor_jej1+switchVmax_efflux_baso*baso_efflux_factor_jej1*phys_Normalized_ESA_baso*phys_BW*basoSurfaceRatio_JEJ1*switch_SFefflux_baso/(drug_Km_efflux_baso+MEM_JEJ1*fu_mem/MJEJ1/drug_molar_mass))*MEM_JEJ1*fu_mem/MJEJ1)
59	d(Liver_drug)/dt = 1/Liver*(-(switch_liverFlag*k_Liver_Venous*Liver_drug) + (switch_liverFlag*k_artery_liver*Artery_drug) + (switch_liverFlag*k_spleen_liver*Spleen_drug) + (switch_liverFlag*Villous_DUO*Qmuc_DUO/VillousDUO) + (switch_liverFlag*Villous_JEJ1*Qmuc_JEJ1/VillousJEJ1) + (switch_liverFlag*Villous_JEJ2*Qmuc_JEJ2/VillousJEJ2) + (switch_liverFlag*Villous_ILL1*Qmuc_ILL1/VillousILL1) + (switch_liverFlag*Villous_ILL2*Qmuc_ILL2/VillousILL2) + (switch_liverFlag*Villous_ILL3*Qmuc_ILL3/VillousILL3) + (switch_liverFlag*Villous_ILL4*Qmuc_ILL4/VillousILL4) - (switch_liverFlag*k_liver_bile*Liver_drug) - (switch_liverFlag*k_liver_metabolites*Liver_drug) + (switch_liverFlag*k_serosa_liver*Serosa_drug))
60	d(Serosa_drug)/dt = 1/Serosa*((k_artery_serosa*Artery_drug) - (switch_liverFlag*k_serosa_liver*Serosa_drug) - ((1-switch_liverFlag)*k_serosa_liver*Serosa_drug))
61	d(Bile_drug_1)/dt = ((1-switch_liverFlag_1)*k_Liver_IC_S5_Bile_1*Liver_IC_S5_drug_1) + ((1-switch_liverFlag_1)*k_Liver_IC_S4_Bile_1*Liver_IC_S4_drug_1) + ((1-switch_liverFlag_1)*k_Liver_IC_S3_Bile_1*Liver_IC_S3_drug_1) + ((1-switch_liverFlag_1)*k_Liver_IC_S2_Bile_1*Liver_IC_S2_drug_1) + ((1-switch_liverFlag_1)*k_Liver_IC_S1_Bile_1*Liver_IC_S1_drug_1) - (drug_k_bile_deg_1*Bile_drug_1) - (k_transit_1*Bile_drug_1) + (switch_liverFlag_1*k_liver_bile_1*Liver_drug_1)

	ODEs
62	d(Venous_drug_1)/dt = 1/Venous_1*(((1-switch_liverFlag_1)*k_Liver_EC_S5_Venous_1*Liver_EC_S5_drug_1) + (k_rest_venous_1*Rest_drug_1) + (k_bone_venous_1*Bone_drug_1) + (k_skin_venous_1*Skin_drug_1) + (k_heart_venous_1*Heart_drug_1) + (k_adipos_venous_1*Adipose_drug_1) + (k_muscle_venous_1*Muscle_drug_1) + (k_brain_venous_1*Brain_drug_1) + (k_kidney_venous_1*Kidney_drug_1) - (k_venous_lung_1*Venous_drug_1) - (k_venous_urine_CLR_1*Venous_drug_1) + (k_testes_venous_1*Testes_drug_1) - (k_venous_urine_GFR_1*Venous_drug_1) + (switch_liverFlag_1*k_Liver_Venous_1*Liver_drug_1))
63	d(Lung_drug_1)/dt = 1/Lung_1*((k_venous_lung_1*Venous_drug_1) - (k_lung_artery_1*Lung_drug_1))
64	d(Kidney_drug_1)/dt = 1/Kidney_1*(-(k_kidney_venous_1*Kidney_drug_1) + (k_artery_kidney_1*Artery_drug_1))
65	d(Brain_drug_1)/dt = 1/Brain_1*(-(k_brain_venous_1*Brain_drug_1) + (k_artery_brain_1*Artery_drug_1))
66	d(Muscle_drug_1)/dt = 1/Muscle_1*(-(k_muscle_venous_1*Muscle_drug_1) + (k_artery_muscle_1*Artery_drug_1))
67	d(Adipose_drug_1)/dt = 1/Adipose_1*(-(k_adipos_venous_1*Adipose_drug_1) + (k_artery_adipos_1*Artery_drug_1))
68	d(Heart_drug_1)/dt = 1/Heart_1*(-(k_heart_venous_1*Heart_drug_1) + (k_artery_heart_1*Artery_drug_1))
69	d(Skin_drug_1)/dt = 1/Skin_1*(-(k_skin_venous_1*Skin_drug_1) + (k_artery_skin_1*Artery_drug_1))
70	d(Bone_drug_1)/dt = 1/Bone_1*(-(k_bone_venous_1*Bone_drug_1) + (k_artery_bone_1*Artery_drug_1))
71	d(Rest_drug_1)/dt = 1/Rest_1*(-(k_rest_venous_1*Rest_drug_1) + (k_artery_rest_1*Artery_drug_1))
72	d(Artery_drug_1)/dt = 1/Artery_1*(-((1-switch_liverFlag_1)*k_artery_liver_1*Artery_drug_1) - (k_artery_spleen_1*Artery_drug_1) - (k_artery_rest_1*Artery_drug_1) - (k_artery_bone_1*Artery_drug_1) - (k_artery_skin_1*Artery_drug_1) - (k_artery_heart_1*Artery_drug_1) - (k_artery_adipos_1*Artery_drug_1) - (k_artery_muscle_1*Artery_drug_1) - (k_artery_brain_1*Artery_drug_1) - (k_artery_kidney_1*Artery_drug_1) + (k_lung_artery_1*Lung_drug_1) - (k_artery_testes_1*Artery_drug_1) - (Qmuc_DUO_1*Artery_drug_1) - (Qmuc_JEJ1_1*Artery_drug_1) - (Qmuc_JEJ2_1*Artery_drug_1) - (Qmuc_ILL1_1*Artery_drug_1) - (Qmuc_ILL2_1*Artery_drug_1) - (Qmuc_ILL3_1*Artery_drug_1) - (Qmuc_ILL4_1*Artery_drug_1) - (k_artery_serosa_1*Artery_drug_1) - (switch_liverFlag_1*k_artery_liver_1*Artery_drug_1))
73	d(Spleen_drug_1)/dt = 1/Spleen_1*(-((1-switch_liverFlag_1)*k_spleen_liver_1*Spleen_drug_1) + (k_artery_spleen_1*Artery_drug_1) - (switch_liverFlag_1*k_spleen_liver_1*Spleen_drug_1))
74	d(Liver_EC_S1_drug_1)/dt = 1/Liver_EC_S1_1*((k_Liver_IC_S1_Liver_EC_S1_1*Liver_IC_S1_drug_1) - (k_Liver_EC_S1_Liver_IC_S1_1*Liver_EC_S1_drug_1) - (k_Liver_EC_S1_Liver_EC_S2_1*Liver_EC_S1_drug_1) + ((1-switch_liverFlag_1)*k_spleen_liver_1*Spleen_drug_1) + ((1-switch_liverFlag_1)*k_artery_liver_1*Artery_drug_1) + (k_Liver_IC_S1_Liver_EC_S1_efflux_1*Liver_IC_S1_drug_1) + ((1-switch_liverFlag_1)*Villous_DUO_1*Qmuc_DUO_1/VillousDUO_1) + ((1-switch_liverFlag_1)*Villous_JEJ1_1*Qmuc_JEJ1_1/VillousJEJ1_1) + ((1-switch_liverFlag_1)*Villous_JEJ2_1*Qmuc_JEJ2_1/VillousJEJ2_1) + ((1-switch_liverFlag_1)*Villous_ILL1_1*Qmuc_ILL1_1/VillousILL1_1) + ((1-switch_liverFlag_1)*Villous_ILL2_1*Qmuc_ILL2_1/VillousILL2_1) + ((1-switch_liverFlag_1)*Villous_ILL3_1*Qmuc_ILL3_1/VillousILL3_1) + ((1-switch_liverFlag_1)*Villous_ILL4_1*Qmuc_ILL4_1/VillousILL4_1) + ((1-switch_liverFlag_1)*k_serosa_liver_1*Serosa_drug_1))
75	d(Liver_EC_S2_drug_1)/dt = 1/Liver_EC_S2_1*((k_Liver_IC_S2_Liver_EC_S2_1*Liver_IC_S2_drug_1) - (k_Liver_EC_S2_Liver_IC_S2_1*Liver_EC_S2_drug_1) - (k_Liver_EC_S2_Liver_EC_S3_1*Liver_EC_S2_drug_1) + (k_Liver_EC_S1_Liver_EC_S2_1*Liver_EC_S1_drug_1) + (k_Liver_IC_S2_Liver_EC_S2_efflux_1*Liver_IC_S2_drug_1))
76	d(Liver_EC_S3_drug_1)/dt = 1/Liver_EC_S3_1*((k_Liver_IC_S3_Liver__EC_S3_1*Liver_IC_S3_drug_1) - (k_Liver_EC_S3_Liver_IC_S3_1*Liver_EC_S3_drug_1) - (k_Liver_EC_S3_Liver_EC_S4_1*Liver_EC_S3_drug_1) + (k_Liver_EC_S2_Liver_EC_S3_1*Liver_EC_S2_drug_1) + (k_Liver_IC_S3_Liver_EC_S3_efflux_1*Liver_IC_S3_drug_1))
77	d(Liver_EC_S4_drug_1)/dt = 1/Liver_EC_S4_1*((k_Liver_IC_S4_Liver_EC_S4_1*Liver_IC_S4_drug_1) - (k_Liver_EC_S4_Liver_IC_S4_1*Liver_EC_S4_drug_1) - (k_Liver_EC_S4_Liver_EC_S5_1*Liver_EC_S4_drug_1) + (k_Liver_EC_S3_Liver_EC_S4_1*Liver_EC_S3_drug_1) + (k_Liver_IC_S4_Liver_EC_S4_efflux_1*Liver_IC_S4_drug_1))
78	d(Liver_EC_S5_drug_1)/dt = 1/Liver_EC_S5_1*((k_Liver_IC_S5_Liver_EC_S5_1*Liver_IC_S5_drug_1) - (k_Liver_EC_S5_Liver_IC_S5_1*Liver_EC_S5_drug_1) - ((1-switch_liverFlag_1)*k_Liver_EC_S5_Venous_1*Liver_EC_S5_drug_1) + (k_Liver_EC_S4_Liver_EC_S5_1*Liver_EC_S4_drug_1) + (k_Liver_IC_S5_Liver_EC_S5_efflux_1*Liver_IC_S5_drug_1))
79	d(Liver_IC_S5_drug_1)/dt = 1/Liver_IC_S5_1*(-((1-switch_liverFlag_1)*k_Liver_IC_S5_Bile_1*Liver_IC_S5_drug_1) - ((1-switch_liverFlag_1)*k_Liver_IC_S5_Metabolites_1*Liver_IC_S5_drug_1) - (k_Liver_IC_S5_Liver_EC_S5_1*Liver_IC_S5_drug_1) + (k_Liver_EC_S5_Liver_IC_S5_1*Liver_EC_S5_drug_1) - (k_Liver_IC_S5_Liver_EC_S5_efflux_1*Liver_IC_S5_drug_1))

	ODEs
80	d(Liver_IC_S3_drug_1)/dt = 1/Liver_IC_S3_1*(-((1-switch_liverFlag_1)*k_Liver_IC_S3_Metabolites_1*Liver_IC_S3_drug_1) - ((1-switch_liverFlag_1)*k_Liver_IC_S3_Bile_1*Liver_IC_S3_drug_1) - (k_Liver_IC_S3_Liver__EC_S3_1*Liver_IC_S3_drug_1) + (k_Liver_EC_S3_Liver_IC_S3_1*Liver_EC_S3_drug_1) - (k_Liver_IC_S3_Liver_EC_S3_efflux_1*Liver_IC_S3_drug_1))
81	d(Liver_IC_S1_drug_1)/dt = 1/Liver_IC_S1_1*(-((1-switch_liverFlag_1)*k_Liver_IC_S1_Metabolites_1*Liver_IC_S1_drug_1) - ((1-switch_liverFlag_1)*k_Liver_IC_S1_Bile_1*Liver_IC_S1_drug_1) - (k_Liver_IC_S1_Liver_EC_S1_1*Liver_IC_S1_drug_1) + (k_Liver_EC_S1_Liver_IC_S1_1*Liver_EC_S1_drug_1) - (k_Liver_IC_S1_Liver_EC_S1_efflux_1*Liver_IC_S1_drug_1))
82	d(Liver_IC_S2_drug_1)/dt = 1/Liver_IC_S2_1*(-((1-switch_liverFlag_1)*k_Liver_IC_S2_Metabolites_1*Liver_IC_S2_drug_1) - ((1-switch_liverFlag_1)*k_Liver_IC_S2_Bile_1*Liver_IC_S2_drug_1) - (k_Liver_IC_S2_Liver_EC_S2_1*Liver_IC_S2_drug_1) + (k_Liver_EC_S2_Liver_IC_S2_1*Liver_EC_S2_drug_1) - (k_Liver_IC_S2_Liver_EC_S2_efflux_1*Liver_IC_S2_drug_1))
83	d(Metabolites_drug_1)/dt = ((1-switch_liverFlag_1)*k_Liver_IC_S5_Metabolites_1*Liver_IC_S5_drug_1) + ((1-switch_liverFlag_1)*k_Liver_IC_S4_Metabolites_1*Liver_IC_S4_drug_1) + ((1-switch_liverFlag_1)*k_Liver_IC_S3_Metabolites_1*Liver_IC_S3_drug_1) + ((1-switch_liverFlag_1)*k_Liver_IC_S2_Metabolites_1*Liver_IC_S2_drug_1) + ((1-switch_liverFlag_1)*k_Liver_IC_S1_Metabolites_1*Liver_IC_S1_drug_1) + (switch_liverFlag_1*k_liver_metabolites_1*Liver_drug_1)
84	d(Testes_drug_1)/dt = 1/Testes_1*((k_artery_testes_1*Artery_drug_1) - (k_testes_venous_1*Testes_drug_1))
85	d(Urine_drug_1)/dt = (k_venous_urine_CLR_1*Venous_drug_1) + (k_venous_urine_GFR_1*Venous_drug_1)
86	d(Liver_IC_S4_drug_1)/dt = 1/Liver_IC_S4_1*(-((1-switch_liverFlag_1)*k_Liver_IC_S4_Metabolites_1*Liver_IC_S4_drug_1) - ((1-switch_liverFlag_1)*k_Liver_IC_S4_Bile_1*Liver_IC_S4_drug_1) - (k_Liver_IC_S4_Liver_EC_S4_1*Liver_IC_S4_drug_1) + (k_Liver_EC_S4_Liver_IC_S4_1*Liver_EC_S4_drug_1) - (k_Liver_IC_S4_Liver_EC_S4_efflux_1*Liver_IC_S4_drug_1))
87	d(X_STOMACH_SOLID_1)/dt = -(X_STOMACH_SOLID_1/TSTOMACH) - (KD_1*X_STOMACH_SOLID_1*(SOLIF_STOMACH_1-X_STOMACH_DISS_1/STOMACH_1))
88	d(X_STOMACH_DISS_1)/dt = -(X_STOMACH_DISS_1/TSTOMACH) + (KD_1*X_STOMACH_SOLID_1*(SOLIF_STOMACH_1-X_STOMACH_DISS_1/STOMACH_1))
89	d(X_DUO_SOLID_1)/dt = (X_STOMACH_SOLID_1/TSTOMACH) - (X_DUO_SOLID_1/TDUO) - (KD_1*X_DUO_SOLID_1*(SOLIF_DUO_1-X_DUO_DISS_1/VDUO_1))
90	d(X_DUO_DISS_1)/dt = (X_STOMACH_DISS_1/TSTOMACH) + (KD_1*X_DUO_SOLID_1*(SOLIF_DUO_1-X_DUO_DISS_1/VDUO_1)) - (X_DUO_DISS_1/TDUO) - ((DIFF_duo_1*NI_DUO_1*switch_SFdiffapi_1*X_DUO_DISS_1)/VDUO_1) + (((switchVmax_efflux_1==zero_1)*efflux_inhib_duo*CLINT_efflux_DUO_1*efflux_factor_duo*switch_SFefflux_1+efflux_inhib_duo*switchVmax_efflux_1*phys_Normalized_ESA*phys_BW*surfaceRatio_DUO*efflux_factor_duo*switch_SFefflux_1/(drug_Km_efflux_1+MEM_DUO_1*fu_mem_1/MDUO_1/drug_molar_mass_1))*MEM_DUO_1*fu_mem_1/MDUO_1) - (((switchVmax_influx_1==zero)*CLINT_influx_DUO_1*influx_factor_duo*switch_SFinflux_1+switchVmax_influx_1*phys_Normalized_ESA*phys_BW*surfaceRatio_DUO*influx_factor_duo/(drug_Km_influx_1+X_DUO_DISS_1/VDUO_1/drug_molar_mass_1))*X_DUO_DISS_1/VDUO_1) + (DIFF_duo_1*switch_SFdiffapi_1*MEM_DUO_1*fu_mem_1/MDUO_1) + (k_transit_1*Bile_drug_1)
91	d(X_JEJ1_SOLID_1)/dt = (X_DUO_SOLID_1/TDUO) - (X_JEJ1_SOLID_1/TJEJ1) - (KD_1*X_JEJ1_SOLID_1*(SOLIF_JEJ1_1-X_JEJ1_DISS_1/VJEJ1_1))
92	d(X_JEJ1_DISS_1)/dt = (X_DUO_DISS_1/TDUO) + (KD_1*X_JEJ1_SOLID_1*(SOLIF_JEJ1_1-X_JEJ1_DISS_1/VJEJ1_1)) - (X_JEJ1_DISS_1/TJEJ1) - ((DIFF_jej1_1*NI_JEJ1_1*switch_SFdiffapi_1*X_JEJ1_DISS_1)/VJEJ1_1) + (((switchVmax_efflux_1==zero)*efflux_inhib_jej1*CLINT_efflux_JEJ1_1*efflux_factor_jej1*switch_SFefflux_1+efflux_inhib_jej1*switchVmax_efflux_1*phys_Normalized_ESA*phys_BW*surfaceRatio_JEJ1*efflux_factor_jej1*switch_SFefflux_1/(drug_Km_efflux_1+MEM_JEJ1_1*fu_mem_1/MJEJ1_1/drug_molar_mass_1))*MEM_JEJ1_1*fu_mem_1/MJEJ1_1) - (((switchVmax_influx_1==zero)*CLINT_influx_JEJ1_1*influx_factor_jej1*switch_SFinflux_1+switchVmax_influx_1*phys_Normalized_ESA*phys_BW*surfaceRatio_JEJ1*influx_factor_jej1/(drug_Km_influx_1+X_JEJ1_DISS_1/VJEJ1_1/drug_molar_mass_1))*X_JEJ1_DISS_1/VJEJ1_1) + (DIFF_jej1_1*switch_SFdiffapi_1*MEM_JEJ1_1*fu_mem_1/MJEJ1_1)
93	d(MEM_DUO_1)/dt = ((DIFF_duo_1*NI_DUO_1*switch_SFdiffapi_1*X_DUO_DISS_1)/VDUO_1) - (((switchVmax_efflux_1==zero_1)*efflux_inhib_duo*CLINT_efflux_DUO_1*efflux_factor_duo*switch_SFefflux_1+efflux_in

	ODEs
	hib_duo*switchVmax_efflux_1*phys_Normalized_ESA*phys_BW*surfaceRatio_DUO*efflux_factor_duo*switch_SFefflux_1/(drug_Km_efflux_1+MEM_DUO_1*fu_mem_1/MDUO_1/drug_molar_mass_1))*MEM_DUO_1*fu_mem_1/MDUO_1) + (((switchVmax_influx_1==zero)*CLINT_influx_DUO_1*influx_factor_duo*switch_SFinflux_1+switchVmax_influx_1*phys_Normalized_ESA*phys_BW*surfaceRatio_DUO*influx_factor_duo/(drug_Km_influx_1+X_DUO_DISS_1/VDUO_1/drug_molar_mass_1))*X_DUO_DISS_1/VDUO_1) - ((CLINT_metabolism_1*metabolism_factor_duo_1*switch_SFgutmet_1*MEM_DUO_1*fu_mem_1)/MDUO_1) - (DIFF_BASO_duo_1*switch_SFdiffbaso_1*MEM_DUO_1*fu_mem_1/MDUO_1) + (DIFF_BASO_duo_1*switch_SFdiffbaso_1*Villous_DUO_1*fu_blood_1/VillousDUO_1) - (DIFF_duo_1*switch_SFdiffapi_1*MEM_DUO_1*fu_mem_1/MDUO_1) + (CLINT_influx_baso_DUO_1*influx_factor_duo_baso*switch_SFinflux_1*Villous_DUO_1/VillousDUO_1) - (((switchVmax_efflux_baso_1==zero)*CLINT_efflux_baso_DUO_1*switch_SFefflux_baso_1*baso_efflux_factor_duo+switchVmax_efflux_baso_1*baso_efflux_factor_duo*phys_Normalized_ESA_baso*phys_BW*basoSurfaceRatio_DUO*switch_SFefflux_baso_1/(drug_Km_efflux_baso_1+MEM_DUO_1*fu_mem_1/MDUO_1/drug_molar_mass_1))*MEM_DUO_1*fu_mem_1/MDUO_1)
94	d(MEM_JEJ1_1)/dt = ((DIFF_jej1_1*NI_JEJ1_1*switch_SFdiffapi_1*X_JEJ1_DISS_1)/VJEJ1_1) - (((switchVmax_efflux_1==zero)*efflux_inhib_jej1*CLINT_efflux_JEJ1_1*efflux_factor_jej1*switch_SFefflux_1+efflux_inhib_jej1*switchVmax_efflux_1*phys_Normalized_ESA*phys_BW*surfaceRatio_JEJ1*efflux_factor_jej1*switch_SFefflux_1/(drug_Km_efflux_1+MEM_JEJ1_1*fu_mem_1/MJEJ1_1/drug_molar_mass_1))*MEM_JEJ1_1*fu_mem_1/MJEJ1_1) + (((switchVmax_influx_1==zero)*CLINT_influx_JEJ1_1*influx_factor_jej1*switch_SFinflux_1+switchVmax_influx_1*phys_Normalized_ESA*phys_BW*surfaceRatio_JEJ1*influx_factor_jej1/(drug_Km_influx_1+X_JEJ1_DISS_1/VJEJ1_1/drug_molar_mass_1))*X_JEJ1_DISS_1/VJEJ1_1) - ((CLINT_metabolism_1*metabolism_factor_jej1_1*switch_SFgutmet_1*MEM_JEJ1_1*fu_mem_1)/MJEJ1_1) - (DIFF_BASO_jej1_1*switch_SFdiffbaso_1*MEM_JEJ1_1*fu_mem_1/MJEJ1_1) + (DIFF_BASO_jej1_1*switch_SFdiffbaso_1*Villous_JEJ1_1*fu_blood_1/VillousJEJ1_1) - (DIFF_jej1_1*switch_SFdiffapi_1*MEM_JEJ1_1*fu_mem_1/MJEJ1_1) + (CLINT_influx_baso_JEJ1_1*influx_factor_jej1_baso*switch_SFinflux_1*Villous_JEJ1_1/VillousJEJ1_1) - (((switchVmax_efflux_baso_1==zero)*CLINT_efflux_baso_JEJ1_1*switch_SFefflux_baso_1*baso_efflux_factor_jej1+switchVmax_efflux_baso_1*baso_efflux_factor_jej1*phys_Normalized_ESA_baso*phys_BW*basoSurfaceRatio_JEJ1*switch_SFefflux_baso_1/(drug_Km_efflux_baso_1+MEM_JEJ1_1*fu_mem_1/MJEJ1_1/drug_molar_mass_1))*MEM_JEJ1_1*fu_mem_1/MJEJ1_1)
95	d(X_JEJ2_SOLID_1)/dt = (X_JEJ1_SOLID_1/TJEJ1) - (X_JEJ2_SOLID_1/TJEJ2) - (KD_1*X_JEJ2_SOLID_1*(SOLIF_JEJ2_1-X_JEJ2_DISS_1/VJEJ2_1))
96	d(X_JEJ2_DISS_1)/dt = (X_JEJ1_DISS_1/TJEJ1) + (KD_1*X_JEJ2_SOLID_1*(SOLIF_JEJ2_1-X_JEJ2_DISS_1/VJEJ2_1)) - (X_JEJ2_DISS_1/TJEJ2) - ((DIFF_jej2_1*NI_JEJ2_1*switch_SFdiffapi_1*X_JEJ2_DISS_1)/VJEJ2_1) + (((switchVmax_efflux_1==zero)*efflux_inhib_jej2*CLINT_efflux_JEJ2_1*efflux_factor_jej2*switch_SFefflux_1+efflux_inhib_jej2*switchVmax_efflux_1*phys_Normalized_ESA*phys_BW*surfaceRatio_JEJ2*efflux_factor_jej2*switch_SFefflux_1/(drug_Km_efflux_1+MEM_JEJ2_1*fu_mem_1/MJEJ2_1/drug_molar_mass_1))*MEM_JEJ2_1*fu_mem_1/MJEJ2_1) - (((switchVmax_influx_1==zero)*CLINT_influx_JEJ2_1*influx_factor_jej2*switch_SFinflux_1+switchVmax_influx_1*phys_Normalized_ESA*phys_BW*surfaceRatio_JEJ2*influx_factor_jej2/(drug_Km_influx_1+X_JEJ2_DISS_1/VJEJ2_1/drug_molar_mass_1))*X_JEJ2_DISS_1/VJEJ2_1) + (DIFF_jej2_1*switch_SFdiffapi_1*MEM_JEJ2_1*fu_mem_1/MJEJ2_1)
97	d(MEM_JEJ2_1)/dt = ((DIFF_jej2_1*NI_JEJ2_1*switch_SFdiffapi_1*X_JEJ2_DISS_1)/VJEJ2_1) - (((switchVmax_efflux_1==zero)*efflux_inhib_jej2*CLINT_efflux_JEJ2_1*efflux_factor_jej2*switch_SFefflux_1+efflux_inhib_jej2*switchVmax_efflux_1*phys_Normalized_ESA*phys_BW*surfaceRatio_JEJ2*efflux_factor_jej2*switch_SFefflux_1/(drug_Km_efflux_1+MEM_JEJ2_1*fu_mem_1/MJEJ2_1/drug_molar_mass_1))*MEM_JEJ2_1*fu_mem_1/MJEJ2_1) + (((switchVmax_influx_1==zero)*CLINT_influx_JEJ2_1*influx_factor_jej2*switch_SFinflux_1+switchVmax_influx_1*phys_Normalized_ESA*phys_BW*surfaceRatio_JEJ2*influx_factor_jej2/(drug_Km_influx_1+X_JEJ2_DISS_1/VJEJ2_1/drug_molar_mass_1))*X_JEJ2_DISS_1/VJEJ2_1) - ((CLINT_metabolism_1*metabolism_factor_jej2_1*switch_SFgutmet_1*MEM_JEJ2_1*fu_mem_1)/MJEJ2_1) - (DIFF_BASO_jej2_1*switch_SFdiffbaso_1*MEM_JEJ2_1*fu_mem_1/MJEJ2_1) + (DIFF_BASO_jej2_1*switch_SFdiffbaso_1*Villous_JEJ2_1*fu_blood_1/VillousJEJ2_1) - (DIFF_jej2_1*switch_SFdiffapi_1*MEM_JEJ2_1*fu_mem_1/MJEJ2_1) + (CLINT_influx_baso_JEJ2_1*influx_factor_jej2_baso*switch_SFinflux_1*Villous_JEJ2_1/VillousJEJ2_1) - (((switchVmax_efflux_baso_1==zero)*CLINT_efflux_baso_JEJ2_1*switch_SFefflux_baso_1*baso_efflux_factor_jej2+switchVmax_efflux_baso_1*baso_efflux_factor_jej2*phys_Normalized_ESA_baso*phys_BW*basoSurfaceRatio_JEJ2*switch_SFefflux_baso_1/(drug_Km_efflux_baso_1+MEM_JEJ2_1*fu_mem_1/MJEJ2_1/drug_molar_mass_1))*MEM_JEJ2_1*fu_mem_1/MJEJ2_1)
98	d(X_ILL1_SOLID_1)/dt = (X_JEJ2_SOLID_1/TJEJ2) - (X_ILL1_SOLID_1/TILL1) - (KD_1*X_ILL1_SOLID_1*(SOLIF_ILL1_1-X_ILL1_DISS_1/VILL1_1))
99	d(X_ILL1_DISS_1)/dt = (X_JEJ2_DISS_1/TJEJ2) + (KD_1*X_ILL1_SOLID_1*(SOLIF_ILL1_1-X_ILL1_DISS_1/VILL1_1)) - (X_ILL1_DISS_1/TILL1) - ((DIFF_ill1_1*NI_ILL1_1*switch_SFdiffapi_1*X_ILL1_DISS_1)/VILL1_1) +

	ODEs
	(((switchVmax_efflux_1==zero)*efflux_inhib_ill1*CLINT_efflux_ILL1_1*efflux_factor_ill1*switch_SFefflux_1+efflux_inhib_ill1*switchVmax_efflux_1*phys_Normalized_ESA*phys_BW*surfaceRatio_ILL1*efflux_factor_ill1*switch_SFefflux_1/(drug_Km_efflux_1+MEM_ILL1_1*fu_mem_1/MILL1_1/drug_molar_mass_1))*MEM_ILL1_1*fu_mem_1/MILL1_1) - (((switchVmax_influx_1==zero)*CLINT_influx_ILL1_1*influx_factor_ill1*switch_SFinflux_1+switchVmax_influx_1*phys_Normalized_ESA*phys_BW*surfaceRatio_ILL1*influx_factor_ill1/(drug_Km_influx_1+X_ILL1_DISS_1/VILL1_1/drug_molar_mass_1))*X_ILL1_DISS_1/VILL1_1) + (DIFF_ill1_1*switch_SFdiffapi_1*MEM_ILL1_1*fu_mem_1/MILL1_1)
100	d(MEM_ILL1_1)/dt = ((DIFF_ill1_1*NI_ILL1_1*switch_SFdiffapi_1*X_ILL1_DISS_1)/VILL1_1) - (((switchVmax_efflux_1==zero)*efflux_inhib_ill1*CLINT_efflux_ILL1_1*efflux_factor_ill1*switch_SFefflux_1+efflux_inhib_ill1*switchVmax_efflux_1*phys_Normalized_ESA*phys_BW*surfaceRatio_ILL1*efflux_factor_ill1*switch_SFefflux_1/(drug_Km_efflux_1+MEM_ILL1_1*fu_mem_1/MILL1_1/drug_molar_mass_1))*MEM_ILL1_1*fu_mem_1/MILL1_1) + (((switchVmax_influx_1==zero)*CLINT_influx_ILL1_1*influx_factor_ill1*switch_SFinflux_1+switchVmax_influx_1*phys_Normalized_ESA*phys_BW*surfaceRatio_ILL1*influx_factor_ill1/(drug_Km_influx_1+X_ILL1_DISS_1/VILL1_1/drug_molar_mass_1))*X_ILL1_DISS_1/VILL1_1) - ((CLINT_metabolism_1*metabolism_factor_ill1_1*switch_SFgutmet_1*MEM_ILL1_1*fu_mem_1)/MILL1_1) - (DIFF_BASO_ill1_1*switch_SFdiffbaso_1*MEM_ILL1_1*fu_mem_1/MILL1_1) + (DIFF_BASO_ill1_1*switch_SFdiffbaso_1*Villous_ILL1_1*fu_blood_1/VillousILL1_1) - (DIFF_ill1_1*switch_SFdiffapi_1*MEM_ILL1_1*fu_mem_1/MILL1_1) + (CLINT_influx_baso_ILL1_1*influx_factor_ill1_baso*switch_SFinflux_1*Villous_ILL1_1/VillousILL1_1) - (((switchVmax_efflux_baso_1==zero)*CLINT_efflux_baso_ILL1_1*switch_SFefflux_baso_1*baso_efflux_factor_ill1+switchVmax_efflux_baso_1*baso_efflux_factor_ill1*phys_Normalized_ESA_baso*phys_BW*basoSurfaceRatio_ILL1*switch_SFefflux_baso_1/(drug_Km_efflux_baso_1+MEM_ILL1_1*fu_mem_1/MILL1_1/drug_molar_mass_1))*MEM_ILL1_1*fu_mem_1/MILL1_1)
101	d(X_ILL2_SOLID_1)/dt = (X_ILL1_SOLID_1/TILL1) - (X_ILL2_SOLID_1/TILL2) - (KD_1*X_ILL2_SOLID_1*(SOLIF_ILL2_1-X_ILL2_DISS_1/VILL2_1))
102	d(X_ILL2_DISS_1)/dt = (X_ILL1_DISS_1/TILL1) + (KD_1*X_ILL2_SOLID_1*(SOLIF_ILL2_1-X_ILL2_DISS_1/VILL2_1)) - (X_ILL2_DISS_1/TILL2) - ((DIFF_ill2_1*NI_ILL2_1*switch_SFdiffapi_1*X_ILL2_DISS_1)/VILL2_1) + (((switchVmax_efflux_1==zero)*efflux_inhib_ill2*CLINT_efflux_ILL2_1*efflux_factor_ill2*switch_SFefflux_1+efflux_inhib_ill2*switchVmax_efflux_1*phys_Normalized_ESA*phys_BW*surfaceRatio_ILL2*efflux_factor_ill2*switch_SFefflux_1/(drug_Km_efflux_1+MEM_ILL2_1*fu_mem_1/MILL2_1/drug_molar_mass_1))*MEM_ILL2_1*fu_mem_1/MILL2_1) - (((switchVmax_influx_1==zero)*CLINT_influx_ILL2_1*influx_factor_ill2*switch_SFinflux_1+switchVmax_influx_1*phys_Normalized_ESA*phys_BW*surfaceRatio_ILL2*influx_factor_ill2/(drug_Km_influx_1+X_ILL2_DISS_1/VILL2_1/drug_molar_mass_1))*X_ILL2_DISS_1/VILL2_1) + (DIFF_ill2_1*switch_SFdiffapi_1*MEM_ILL2_1*fu_mem_1/MILL2_1)
103	d(MEM_ILL2_1)/dt = ((DIFF_ill2_1*NI_ILL2_1*switch_SFdiffapi_1*X_ILL2_DISS_1)/VILL2_1) - (((switchVmax_efflux_1==zero)*efflux_inhib_ill2*CLINT_efflux_ILL2_1*efflux_factor_ill2*switch_SFefflux_1+efflux_inhib_ill2*switchVmax_efflux_1*phys_Normalized_ESA*phys_BW*surfaceRatio_ILL2*efflux_factor_ill2*switch_SFefflux_1/(drug_Km_efflux_1+MEM_ILL2_1*fu_mem_1/MILL2_1/drug_molar_mass_1))*MEM_ILL2_1*fu_mem_1/MILL2_1) + (((switchVmax_influx_1==zero)*CLINT_influx_ILL2_1*influx_factor_ill2*switch_SFinflux_1+switchVmax_influx_1*phys_Normalized_ESA*phys_BW*surfaceRatio_ILL2*influx_factor_ill2/(drug_Km_influx_1+X_ILL2_DISS_1/VILL2_1/drug_molar_mass_1))*X_ILL2_DISS_1/VILL2_1) - ((CLINT_metabolism_1*metabolism_factor_ill2_1*switch_SFgutmet_1*MEM_ILL2_1*fu_mem_1)/MILL2_1) + (DIFF_BASO_ill2_1*switch_SFdiffbaso_1*Villous_ILL2_1*fu_blood_1/VillousILL2_1) - (DIFF_BASO_ill2_1*switch_SFdiffbaso_1*MEM_ILL2_1*fu_mem_1/MILL2_1) - (DIFF_ill2_1*switch_SFdiffapi_1*MEM_ILL2_1*fu_mem_1/MILL2_1) + (CLINT_influx_baso_ILL2_1*influx_factor_ill2_baso*switch_SFinflux_1*Villous_ILL2_1/VillousILL2_1) - (((switchVmax_efflux_baso_1==zero)*CLINT_efflux_baso_ILL2_1*switch_SFefflux_baso_1*baso_efflux_factor_ill2+switchVmax_efflux_baso_1*baso_efflux_factor_ill2*phys_Normalized_ESA_baso*phys_BW*basoSurfaceRatio_ILL2*switch_SFefflux_baso_1/(drug_Km_efflux_baso_1+MEM_ILL2_1*fu_mem_1/MILL2_1/drug_molar_mass_1))*MEM_ILL2_1*fu_mem_1/MILL2_1)
104	d(X_ILL3_SOLID_1)/dt = (X_ILL2_SOLID_1/TILL2) - (X_ILL3_SOLID_1/TILL3) - (KD_1*X_ILL3_SOLID_1*(SOLIF_ILL3_1-X_ILL3_DISS_1/VILL3_1))
105	d(X_ILL3_DISS_1)/dt = (X_ILL2_DISS_1/TILL2) + (KD_1*X_ILL3_SOLID_1*(SOLIF_ILL3_1-X_ILL3_DISS_1/VILL3_1)) - (X_ILL3_DISS_1/TILL3) - ((DIFF_ill3_1*NI_ILL3_1*switch_SFdiffapi_1*X_ILL3_DISS_1)/VILL3_1) + (((switchVmax_efflux_1==zero)*efflux_inhib_ill3*CLINT_efflux_ILL3_1*efflux_factor_ill3*switch_SFefflux_1+efflux_inhib_ill3*switchVmax_efflux_1*phys_Normalized_ESA*phys_BW*surfaceRatio_ILL3*efflux_factor_ill3*switch_SFefflux_1/(drug_Km_efflux_1+MEM_ILL3_1*fu_mem_1/MILL3_1/drug_molar_mass_1))*MEM_ILL3_1*fu_mem_1/MILL3_1) - (((switchVmax_influx_1==zero)*CLINT_influx_ILL3_1*influx_factor_ill3*switch_SFinflux_1+switchVmax_influx_1*phys_Normalized_ESA*phys_BW*surfaceRatio_ILL3*influx_factor_ill3/(drug_Km_influx_1+X_ILL3_DISS_1/VILL3_1/drug_molar_mass_1))*X_ILL3_DISS_1/VILL3_1) + (DIFF_ill3_1*switch_SFdiffapi_1*MEM_ILL3_1*fu_mem_1/MILL3_1)
106	d(MEM_ILL3_1)/dt = ((DIFF_ill3_1*NI_ILL3_1*switch_SFdiffapi_1*X_ILL3_DISS_1)/VILL3_1) - (((switchVmax_efflux_1==zero)*efflux_inhib_ill3*CLINT_efflux_ILL3_1*efflux_factor_ill3*switch_SFefflux_1+efflux_inhib_ill3*switchVmax_efflux_1*phys_Normalized_ESA*phys_BW*surfaceRatio_ILL3*efflux_factor_ill3*switch_SFefflux_1/(drug_

	ODEs
	Km_efflux_1+MEM_ILL3_1*fu_mem_1/MILL3_1/drug_molar_mass_1))*MEM_ILL3_1*fu_mem_1/MILL3_1) + (((switchVmax_influx_1==zero)*CLINT_influx_ILL3_1*influx_factor_ill3*switch_SFinflux_1+switchVmax_influx_1*phys_Normalized_ESA*phys_BW*surfaceRatio_ILL3*influx_factor_ill3/(drug_Km_influx_1+X_ILL3_DISS_1/VILL3_1/drug_molar_mass_1))*X_ILL3_DISS_1/VILL3_1) - ((CLINT_metabolism_1*metabolism_factor_ill3_1*switch_SFgutmet_1*MEM_ILL3_1*fu_mem_1)/MILL3_1) + (DIFF_BASO_ill3_1*switch_SFdiffbaso_1*Villous_ILL3_1*fu_blood_1/VillousILL3_1) - (DIFF_BASO_ill3_1*switch_SFdiffbaso_1*MEM_ILL3_1*fu_mem_1/MILL3_1) - (DIFF_ill3_1*switch_SFdiffapi_1*MEM_ILL3_1*fu_mem_1/MILL3_1) + (CLINT_influx_baso_ILL3_1*influx_factor_ill3_baso*switch_SFinflux_1*Villous_ILL3_1/VillousILL3_1) - (((switchVmax_efflux_baso_1==zero)*CLINT_efflux_baso_ILL3_1*switch_SFefflux_baso_1*baso_efflux_factor_ill3+switchVmax_efflux_baso_1*baso_efflux_factor_ill3*phys_Normalized_ESA_baso*phys_BW*basoSurfaceRatio_ILL3*switch_SFefflux_baso_1/(drug_Km_efflux_baso_1+MEM_ILL3_1*fu_mem_1/MILL3_1/drug_molar_mass_1))*MEM_ILL3_1*fu_mem_1/MILL3_1)
107	d(X_ILL4_SOLID_1)/dt = (X_ILL3_SOLID_1/TILL3) - (X_ILL4_SOLID_1/TILL4) - (KD_1*X_ILL4_SOLID_1*(SOLIF_ILL4_1-X_ILL4_DISS_1/VILL4_1))
108	d(X_ILL4_DISS_1)/dt = (X_ILL3_DISS_1/TILL3) + (KD_1*X_ILL4_SOLID_1*(SOLIF_ILL4_1-X_ILL4_DISS_1/VILL4_1)) - (X_ILL4_DISS_1/TILL4) - ((DIFF_ill4_1*NI_ILL4_1*switch_SFdiffapi_1*X_ILL4_DISS_1)/VILL4_1) + (((switchVmax_efflux_1==zero)*efflux_inhib_ill4*CLINT_efflux_ILL4_1*efflux_factor_ill4*switch_SFefflux_1+efflux_inhib_ill4*switchVmax_efflux_1*phys_Normalized_ESA*phys_BW*surfaceRatio_ILL4*efflux_factor_ill4*switch_SFefflux_1/(drug_Km_efflux_1+MEM_ILL4_1*fu_mem_1/MILL4_1/drug_molar_mass_1))*MEM_ILL4_1*fu_mem_1/MILL4_1) - (((switchVmax_influx_1==zero)*CLINT_influx_ILL4_1*influx_factor_ill4*switch_SFinflux_1+switchVmax_influx_1*phys_Normalized_ESA*phys_BW*surfaceRatio_ILL4*influx_factor_ill4/(drug_Km_influx_1+X_ILL4_DISS_1/VILL4_1/drug_molar_mass_1))*X_ILL4_DISS_1/VILL4_1) + (DIFF_ill4_1*switch_SFdiffapi_1*MEM_ILL4_1*fu_mem_1/MILL4_1)
109	d(MEM_ILL4_1)/dt = ((DIFF_ill4_1*NI_ILL4_1*switch_SFdiffapi_1*X_ILL4_DISS_1)/VILL4_1) - (((switchVmax_efflux_1==zero)*efflux_inhib_ill4*CLINT_efflux_ILL4_1*efflux_factor_ill4*switch_SFefflux_1+efflux_inhib_ill4*switchVmax_efflux_1*phys_Normalized_ESA*phys_BW*surfaceRatio_ILL4*efflux_factor_ill4*switch_SFefflux_1/(drug_Km_efflux_1+MEM_ILL4_1*fu_mem_1/MILL4_1/drug_molar_mass_1))*MEM_ILL4_1*fu_mem_1/MILL4_1) + (((switchVmax_influx_1==zero)*CLINT_influx_ILL4_1*influx_factor_ill4*switch_SFinflux_1+switchVmax_influx_1*phys_Normalized_ESA*phys_BW*surfaceRatio_ILL4*influx_factor_ill4/(drug_Km_influx_1+X_ILL4_DISS_1/VILL4_1/drug_molar_mass_1))*X_ILL4_DISS_1/VILL4_1) - ((CLINT_metabolism_1*metabolism_factor_ill4_1*switch_SFgutmet_1*MEM_ILL4_1*fu_mem_1)/MILL4_1) - (DIFF_ill4_1*switch_SFdiffapi_1*MEM_ILL4_1*fu_mem_1/MILL4_1) + (DIFF_BASO_ill4_1*switch_SFdiffbaso_1*Villous_ILL4_1*fu_blood_1/VillousILL4_1) - (DIFF_BASO_ill4_1*switch_SFdiffbaso_1*MEM_ILL4_1*fu_mem_1/MILL4_1) + (CLINT_influx_baso_ILL4_1*influx_factor_ill4_baso*switch_SFinflux_1*Villous_ILL4_1/VillousILL4_1) - (((switchVmax_efflux_baso_1==zero)*CLINT_efflux_baso_ILL4_1*switch_SFefflux_baso_1*baso_efflux_factor_ill4+switchVmax_efflux_baso_1*baso_efflux_factor_ill4*phys_Normalized_ESA_baso*phys_BW*basoSurfaceRatio_ILL4*switch_SFefflux_baso_1/(drug_Km_efflux_baso_1+MEM_ILL4_1*fu_mem_1/MILL4_1/drug_molar_mass_1))*MEM_ILL4_1*fu_mem_1/MILL4_1)
110	d(X_CECUM_SOLID_1)/dt = (X_ILL4_SOLID_1/TILL4)
111	d(X_CECUM_DISS_1)/dt = (X_ILL4_DISS_1/TILL4)
112	d(Villous_DUO_1)/dt = (DIFF_BASO_duo_1*switch_SFdiffbaso_1*MEM_DUO_1*fu_mem_1/MDUO_1) - (DIFF_BASO_duo_1*switch_SFdiffbaso_1*Villous_DUO_1*fu_blood_1/VillousDUO_1) - (CLINT_influx_baso_DUO_1*influx_factor_duo_baso*switch_SFinflux_1*Villous_DUO_1/VillousDUO_1) + (Qmuc_DUO_1*Artery_drug_1) - (switch_liverFlag_1*Villous_DUO_1*Qmuc_DUO_1/VillousDUO_1) - ((1-switch_liverFlag_1)*Villous_DUO_1*Qmuc_DUO_1/VillousDUO_1) + (((switchVmax_efflux_baso_1==zero)*CLINT_efflux_baso_DUO_1*switch_SFefflux_baso_1*baso_efflux_factor_duo+switchVmax_efflux_baso_1*baso_efflux_factor_duo*phys_Normalized_ESA_baso*phys_BW*basoSurfaceRatio_DUO*switch_SFefflux_baso_1/(drug_Km_efflux_baso_1+MEM_DUO_1*fu_mem_1/MDUO_1/drug_molar_mass_1))*MEM_DUO_1*fu_mem_1/MDUO_1)
113	d(Villous_JEJ2_1)/dt = (DIFF_BASO_jej2_1*switch_SFdiffbaso_1*MEM_JEJ2_1*fu_mem_1/MJEJ2_1) - (DIFF_BASO_jej2_1*switch_SFdiffbaso_1*Villous_JEJ2_1*fu_blood_1/VillousJEJ2_1) - (CLINT_influx_baso_JEJ2_1*influx_factor_jej2_baso*switch_SFinflux_1*Villous_JEJ2_1/VillousJEJ2_1) + (Qmuc_JEJ2_1*Artery_drug_1) - (switch_liverFlag_1*Villous_JEJ2_1*Qmuc_JEJ2_1/VillousJEJ2_1) - ((1-switch_liverFlag_1)*Villous_JEJ2_1*Qmuc_JEJ2_1/VillousJEJ2_1) + (((switchVmax_efflux_baso_1==zero)*CLINT_efflux_baso_JEJ2_1*switch_SFefflux_baso_1*baso_efflux_factor_jej2+switchVmax_efflux_baso_1*baso_efflux_factor_jej2*phys_Normalized_ESA_baso*phys_BW*basoSurfaceRatio_JEJ2*switch_SFefflux_baso_1/(drug_Km_efflux_baso_1+MEM_JEJ2_1*fu_mem_1/MJEJ2_1/drug_molar_mass_1))*MEM_JEJ2_1*fu_mem_1/MJEJ2_1)

	ODEs
114	d(Villous_ILL1_1)/dt = (DIFF_BASO_ill1_1*switch_SFdiffbaso_1*MEM_ILL1_1*fu_mem_1/MILL1_1) - (DIFF_BASO_ill1_1*switch_SFdiffbaso_1*Villous_ILL1_1*fu_blood_1/VillousILL1_1) - (CLINT_influx_baso_ILL1_1*influx_factor_ill1_baso*switch_SFinflux_1*Villous_ILL1_1/VillousILL1_1) + (Qmuc_ILL1_1*Artery_drug_1) - (switch_liverFlag_1*Villous_ILL1_1*Qmuc_ILL1_1/VillousILL1_1) - ((1-switch_liverFlag_1)*Villous_ILL1_1*Qmuc_ILL1_1/VillousILL1_1) + (((switchVmax_efflux_baso_1==zero)*CLINT_efflux_baso_ILL1_1*switch_SFefflux_baso_1*baso_efflux_factor_ill1+switchVmax_efflux_baso_1*baso_efflux_factor_ill1*phys_Normalized_ESA_baso*phys_BW*basoSurfaceRatio_ILL1*switch_SFefflux_baso_1/(drug_Km_efflux_baso_1+MEM_ILL1_1*fu_mem_1/MILL1_1/drug_molar_mass_1))*MEM_ILL1_1*fu_mem_1/MILL1_1)
115	d(Villous_ILL2_1)/dt = -(DIFF_BASO_ill2_1*switch_SFdiffbaso_1*Villous_ILL2_1*fu_blood_1/VillousILL2_1) + (DIFF_BASO_ill2_1*switch_SFdiffbaso_1*MEM_ILL2_1*fu_mem_1/MILL2_1) - (CLINT_influx_baso_ILL2_1*influx_factor_ill2_baso*switch_SFinflux_1*Villous_ILL2_1/VillousILL2_1) + (Qmuc_ILL2_1*Artery_drug_1) - (switch_liverFlag_1*Villous_ILL2_1*Qmuc_ILL2_1/VillousILL2_1) - ((1-switch_liverFlag_1)*Villous_ILL2_1*Qmuc_ILL2_1/VillousILL2_1) + (((switchVmax_efflux_baso_1==zero)*CLINT_efflux_baso_ILL2_1*switch_SFefflux_baso_1*baso_efflux_factor_ill2+switchVmax_efflux_baso_1*baso_efflux_factor_ill2*phys_Normalized_ESA_baso*phys_BW*basoSurfaceRatio_ILL2*switch_SFefflux_baso_1/(drug_Km_efflux_baso_1+MEM_ILL2_1*fu_mem_1/MILL2_1/drug_molar_mass_1))*MEM_ILL2_1*fu_mem_1/MILL2_1)
116	d(Villous_ILL3_1)/dt = -(DIFF_BASO_ill3_1*switch_SFdiffbaso_1*Villous_ILL3_1*fu_blood_1/VillousILL3_1) + (DIFF_BASO_ill3_1*switch_SFdiffbaso_1*MEM_ILL3_1*fu_mem_1/MILL3_1) - (CLINT_influx_baso_ILL3_1*influx_factor_ill3_baso*switch_SFinflux_1*Villous_ILL3_1/VillousILL3_1) + (Qmuc_ILL3_1*Artery_drug_1) - (switch_liverFlag_1*Villous_ILL3_1*Qmuc_ILL3_1/VillousILL3_1) - ((1-switch_liverFlag_1)*Villous_ILL3_1*Qmuc_ILL3_1/VillousILL3_1) + (((switchVmax_efflux_baso_1==zero)*CLINT_efflux_baso_ILL3_1*switch_SFefflux_baso_1*baso_efflux_factor_ill3+switchVmax_efflux_baso_1*baso_efflux_factor_ill3*phys_Normalized_ESA_baso*phys_BW*basoSurfaceRatio_ILL3*switch_SFefflux_baso_1/(drug_Km_efflux_baso_1+MEM_ILL3_1*fu_mem_1/MILL3_1/drug_molar_mass_1))*MEM_ILL3_1*fu_mem_1/MILL3_1)
117	d(Villous_ILL4_1)/dt = -(DIFF_BASO_ill4_1*switch_SFdiffbaso_1*Villous_ILL4_1*fu_blood_1/VillousILL4_1) + (DIFF_BASO_ill4_1*switch_SFdiffbaso_1*MEM_ILL4_1*fu_mem_1/MILL4_1) - (CLINT_influx_baso_ILL4_1*influx_factor_ill4_baso*switch_SFinflux_1*Villous_ILL4_1/VillousILL4_1) + (Qmuc_ILL4_1*Artery_drug_1) - (switch_liverFlag_1*Villous_ILL4_1*Qmuc_ILL4_1/VillousILL4_1) - ((1-switch_liverFlag_1)*Villous_ILL4_1*Qmuc_ILL4_1/VillousILL4_1) + (((switchVmax_efflux_baso_1==zero)*CLINT_efflux_baso_ILL4_1*switch_SFefflux_baso_1*baso_efflux_factor_ill4+switchVmax_efflux_baso_1*baso_efflux_factor_ill4*phys_Normalized_ESA_baso*phys_BW*basoSurfaceRatio_ILL4*switch_SFefflux_baso_1/(drug_Km_efflux_baso_1+MEM_ILL4_1*fu_mem_1/MILL4_1/drug_molar_mass_1))*MEM_ILL4_1*fu_mem_1/MILL4_1)
118	d(Villous_JEJ1_1)/dt = (DIFF_BASO_jej1_1*switch_SFdiffbaso_1*MEM_JEJ1_1*fu_mem_1/MJEJ1_1) - (DIFF_BASO_jej1_1*switch_SFdiffbaso_1*Villous_JEJ1_1*fu_blood_1/VillousJEJ1_1) - (CLINT_influx_baso_JEJ1_1*influx_factor_jej1_baso*switch_SFinflux_1*Villous_JEJ1_1/VillousJEJ1_1) + (Qmuc_JEJ1_1*Artery_drug_1) - (switch_liverFlag_1*Villous_JEJ1_1*Qmuc_JEJ1_1/VillousJEJ1_1) - ((1-switch_liverFlag_1)*Villous_JEJ1_1*Qmuc_JEJ1_1/VillousJEJ1_1) + (((switchVmax_efflux_baso_1==zero)*CLINT_efflux_baso_JEJ1_1*switch_SFefflux_baso_1*baso_efflux_factor_jej1+switchVmax_efflux_baso_1*baso_efflux_factor_jej1*phys_Normalized_ESA_baso*phys_BW*basoSurfaceRatio_JEJ1*switch_SFefflux_baso_1/(drug_Km_efflux_baso_1+MEM_JEJ1_1*fu_mem_1/MJEJ1_1/drug_molar_mass_1))*MEM_JEJ1_1*fu_mem_1/MJEJ1_1)
119	d(Liver_drug_1)/dt = 1/Liver_1*(-(switch_liverFlag_1*k_Liver_Venous_1*Liver_drug_1) + (switch_liverFlag_1*Villous_DUO_1*Qmuc_DUO_1/VillousDUO_1) + (switch_liverFlag_1*Villous_JEJ1_1*Qmuc_JEJ1_1/VillousJEJ1_1) + (switch_liverFlag_1*Villous_JEJ2_1*Qmuc_JEJ2_1/VillousJEJ2_1) + (switch_liverFlag_1*Villous_ILL1_1*Qmuc_ILL1_1/VillousILL1_1) + (switch_liverFlag_1*Villous_ILL2_1*Qmuc_ILL2_1/VillousILL2_1) + (switch_liverFlag_1*Villous_ILL3_1*Qmuc_ILL3_1/VillousILL3_1) + (switch_liverFlag_1*Villous_ILL4_1*Qmuc_ILL4_1/VillousILL4_1) + (switch_liverFlag_1*k_serosa_liver_1*Serosa_drug_1) + (switch_liverFlag_1*k_artery_liver_1*Artery_drug_1) + (switch_liverFlag_1*k_spleen_liver_1*Spleen_drug_1) - (switch_liverFlag_1*k_liver_metabolites_1*Liver_drug_1) - (switch_liverFlag_1*k_liver_bile_1*Liver_drug_1))
120	d(Serosa_drug_1)/dt = 1/Serosa_1*((k_artery_serosa_1*Artery_drug_1) - (switch_liverFlag_1*k_serosa_liver_1*Serosa_drug_1) - ((1-switch_liverFlag_1)*k_serosa_liver_1*Serosa_drug_1))

Program Setup

Model Setup

Variants

	Type	Name	Human_phys	Human_physiology_ADAM	Digoxin	Pgp_Ratios	Digoxin_Caco2
1	parameter	phys_BW	70		70		
2	parameter	phys_Normalized_Q_adipose	4				
3	parameter	phys_Normalized_Q_bone	4				
4	parameter	phys_Normalized_Q_brain	10				
5	parameter	phys_Normalized_Q_gut	17				
6	parameter	phys_Normalized_Q_heart	3				
7	parameter	phys_Normalized_Q_kidney	15				
8	parameter	phys_Normalized_Q_liver	20				
9	parameter	phys_Normalized_Q_lung	80				
10	parameter	phys_Normalized_Q_muscle	14				
11	parameter	phys_Normalized_Q_remainder	1				
12	parameter	phys_Normalized_Q_skin	4				
13	parameter	phys_Normalized_Q_spleen	2				
14	parameter	phys_Normalized_Q_testes	0				
15	parameter	phys_Normalized_weight_adipose	197				
16	parameter	phys_Normalized_weight_artery	25.7				
17	parameter	phys_Normalized_weight_bone	158				
18	parameter	phys_Normalized_weight_brain	21				
19	parameter	phys_Normalized_weight_gut	18				

	Type	Name	Human_phys	Human_physiology_ADAM	Digoxin	Pgp_Ratios	Digoxin_Caco2
20	parameter	phys_Normalized_weight_heart	5				
21	parameter	phys_Normalized_weight_kidney	5				
22	parameter	phys_Normalized_weight_liver_blood	4.9				
23	parameter	phys_Normalized_weight_liver_tissue	18				
24	parameter	phys_Normalized_weight_lung	8				
25	parameter	phys_Normalized_weight_muscle	416				
26	parameter	phys_Normalized_weight_remainder	100				
27	parameter	phys_Normalized_weight_skin	41				
28	parameter	phys_Normalized_weight_spleen	3				
29	parameter	phys_HPGL	125				
30	parameter	phys_Normalized_weight_venous	51.4				
31	parameter	LL		680			
32	parameter	LR		1.75			
33	parameter	phys_ESA		120000			
34	parameter	TSTOMACH		16.2			
35	parameter	TDUO		9.384			
36	parameter	TJEJ1		35.292			
37	parameter	TJEJ2		35.292			
38	parameter	TILL1		31.008			
39	parameter	TILL2		31.008			
40	parameter	TILL3		31.008			
41	parameter	TILL4		31.008			
42	parameter	phys_Normalized_weight_stomach		2.1			

	Type	Name	Human_phys	Human_physiology_ADAM	Digoxin	Pgp_Ratios	Digoxin_Caco2
43	parameter	pHStomach		1.5			
44	parameter	pHDuo		6.4			
45	parameter	pHJej1		6.5			
46	parameter	pHJej2		6.6			
47	parameter	pHIll1		6.8			
48	parameter	pHIll2		7			
49	parameter	pHIll3		7.7			
50	parameter	pHIll4		7.3			
51	parameter	BW_average		70			
52	parameter	numIntestinalCompartments		7			
53	parameter	Gut_EC_fraction		0.3719			
54	parameter	Gut_IC_fraction		0.6281			
55	parameter	influx_factor_duo		1			
56	parameter	influx_factor_jej1		1			
57	parameter	influx_factor_jej2		1			
58	parameter	influx_factor_ill1		1			
59	parameter	influx_factor_ill2		1			
60	parameter	influx_factor_ill3		1			
61	parameter	influx_factor_ill4		1			
62	parameter	efflux_factor_duo		0.51		0.23	
63	parameter	efflux_factor_jej1		1		1	
64	parameter	efflux_factor_jej2		1.46		1.44	
65	parameter	efflux_factor_ill1		1.5		2.14	
66	parameter	efflux_factor_ill2		1.51		2.14	
67	parameter	efflux_factor_ill3		1.52		2.14	
68	parameter	efflux_factor_ill4		1.51		2.14	
69	parameter	volumeRatio_DUO		0.11534			
70	parameter	volumeRatio_JEJ1		0.22722			
71	parameter	volumeRatio_JEJ2		0.15917			
72	parameter	volumeRatio_ILL1		0.12687			

	Type	Name	Human_phys	Human_physiology_ADAM	Digoxin	Pgp_Ratios	Digoxin_Caco2
73	parameter	volumeRatio_ILL2		0.12687			
74	parameter	volumeRatio_ILL3		0.12457			
75	parameter	volumeRatio_ILL4		0.11995			
76	parameter	flowRatio_DUO		0.088			
77	parameter	flowRatio_JEJ1		0.242			
78	parameter	flowRatio_JEJ2		0.242			
79	parameter	flowRatio_ILL1		0.107			
80	parameter	flowRatio_ILL2		0.107			
81	parameter	flowRatio_ILL3		0.107			
82	parameter	flowRatio_ILL4		0.107			
83	parameter	metabolism_factor_duo		1			
84	parameter	metabolism_factor_jej1		1			
85	parameter	metabolism_factor_jej2		1			
86	parameter	metabolism_factor_ill1		1			
87	parameter	metabolism_factor_ill2		1			
88	parameter	metabolism_factor_ill3		1			
89	parameter	metabolism_factor_ill4		1			
90	parameter	phys_Normalized_weight_enterocyte		7.3857			
91	parameter	LumenTotal		126.95			
92	parameter	lumenvolumeRatio_DUO		0.27058			
93	parameter	lumenvolumeRatio_JEJ1		0.16621			
94	parameter	lumenvolumeRatio_JEJ2		0.16621			
95	parameter	lumenvolumeRatio_ILL1		0.099252			

	Type	Name	Human_phys	Human_physiology_ADAM	Digoxin	Pgp_Ratios	Digoxin_Caco2
96	parameter	lumenvolumeRatio_ILL2		0.099252			
97	parameter	lumenvolumeRatio_ILL3		0.099252			
98	parameter	lumenvolumeRatio_ILL4		0.099252			
99	parameter	StomachLumenTotal		50			
100	parameter	phys_ESA_baso		6703			
101	parameter	surfaceRatio_DUO		0.115			
102	parameter	surfaceRatio_JEJ1		0.227			
103	parameter	surfaceRatio_JEJ2		0.159			
104	parameter	surfaceRatio_ILL1		0.127			
105	parameter	surfaceRatio_ILL2		0.127			
106	parameter	surfaceRatio_ILL3		0.125			
107	parameter	surfaceRatio_ILL4		0.12			
108	parameter	basoSurfaceRatio_DUO		0.0526			
109	parameter	basoSurfaceRatio_JEJ1		0.2026			
110	parameter	basoSurfaceRatio_JEJ2		0.2026			
111	parameter	basoSurfaceRatio_ILL1		0.1356			
112	parameter	basoSurfaceRatio_ILL2		0.1356			
113	parameter	basoSurfaceRatio_ILL3		0.1356			
114	parameter	basoSurfaceRatio_ILL4		0.1356			
115	parameter	drug_fQ		0.2571			
116	parameter	influx_factor_duo_baso		1			

	Type	Name	Human_phys	Human_physiology_ADAM	Digoxin	Pgp_Ratios	Digoxin_Caco2
117	parameter	influx_factor_jej1_baso		1			
118	parameter	influx_factor_jej2_baso		1			
119	parameter	influx_factor_ill1_baso		1			
120	parameter	influx_factor_ill2_baso		1			
121	parameter	influx_factor_ill3_baso		1			
122	parameter	influx_factor_ill4_baso		1			
123	parameter	fu_mem			0.42635		
124	parameter	LOGP			1.26		
125	parameter	MW			780940		
126	parameter	REFPHSOL			6.5		
127	parameter	PSIZE			0.0065		
128	parameter	PDENSITY			1000000		
129	parameter	pKA			0		
130	parameter	SOLFASSIF			64		
131	parameter	drug_Km_influx			1		
132	parameter	drug_Km_efflux			177		2.43
133	parameter	switchVmax_influx			0		
134	parameter	switchVmax_efflux			0		2.2076e-05
135	parameter	switch_SFinflux			1		
136	parameter	switch_SFefflux			1		
137	parameter	switch_SFgutmet			1		
138	parameter	switch_SFdiffapi			1		

	Type	Name	Human_phys	Human_physiology_ADAM	Digoxin	Pgp_Ratios	Digoxin_Caco2
139	parameter	switch_SFdiffbaso			1		
140	parameter	CL_inf_api			0		
141	parameter	CL_eff			0		
142	parameter	CLINT_metabolism			0		
143	parameter	CL_inf_baso			0		
144	parameter	drug_Kp_serosa_raw			1.2434		
145	parameter	drug_Kp_liver_raw			1.1308		
146	parameter	diff_baso			4.98		0.162
147	parameter	diff_api			4.98		
148	parameter	drug_PSbileg			0		
149	parameter	drug_fuLiver			0.42635		
150	parameter	drug_fumic			0.92106		
151	parameter	drug_funic			0.92106		
152	parameter	drug_fuplasma			0.71		
153	parameter	drug_CLmetg			0.37		
154	parameter	drug_HLM_CLint			0		
155	parameter	drug_Kp_adipose_raw			0.78172		
156	parameter	drug_Kp_bone_raw			1.2843		
157	parameter	drug_Kp_brain_raw			1.4623		
158	parameter	drug_Kp_gut_raw			1.2434		
159	parameter	drug_Kp_heart_raw			60.76		
160	parameter	drug_Kp_kidney_raw			0.92637		
161	parameter	drug_Kp_lung_raw			0.69336		
162	parameter	drug_Kp_muscle_raw			15.96		

	Type	Name	Human_phys	Human_physiology_ADAM	Digoxin	Pgp_Ratios	Digoxin_Caco2
163	parameter	drug_Kp_rest_raw			0.78172		
164	parameter	switch_SFKp			1		
165	parameter	drug_Kp_skin_raw			0.94756		
166	parameter	drug_Kp_spleen_raw			0.95363		
167	parameter	drug_molar_mass			780940		
168	parameter	drug_BRP			1.07		
169	parameter	switch_SFbile			1		
170	parameter	switch_SFmet			1		
171	parameter	switch_SFdiff			1		
172	parameter	drug_CLrenal			9.66		
173	parameter	drug_FR			0		
174	parameter	switch_slow_dist_Kp			1		
175	parameter	switch_SFrenal			1		
176	parameter	switch_liverFlag			1		
177	parameter	fu_blood			0.66355		
178	parameter	drug_inputFlag			6		
179	parameter	drug_dose_amount_IV			1		
180	parameter	switchVmax_efflux_baso					3.049e-09
181	parameter	drug_Km_efflux_baso					658

Data Step

Data Map

Classification	Value
group	Group

Classification	Value
independent	Time_hr
response	Conc_ctrl_ng_ml ~ Plasma_total.Plasma_total_drug
dose from data	Dose_IV_injection_mg -> Venous.Venous_drug
	Infusion Data Column: Dose_rate_IV_infusion_mg_hr
dose from data	Dose_PO_mg -> STOMACH.X_STOMACH_DISS
	Bolus

Variant and Dose Setup Step

Variant and Dose Setup

Group	Variants1	Variants2	Variants3	Variants4	Variants5	Variants6	Doses1	Doses2	Doses3
	**Baseline**	**Baseline**	**Baseline**	**Baseline**	**Baseline**	**Group Specific**	**Group Specific**	**Data**	**Data**
1	Digoxin_Caco2	Digoxin	Pgp_Ratios	Human_physiology_ADAM	Human_phys			Dose_IV_injection_mg	Dose_PO_mg
2	Digoxin_Caco2	Digoxin	Pgp_Ratios	Human_physiology_ADAM	Human_phys			Dose_IV_injection_mg	Dose_PO_mg
3	Digoxin_Caco2	Digoxin	Pgp_Ratios	Human_physiology_ADAM	Human_phys			Dose_IV_injection_mg	Dose_PO_mg
4	Digoxin_Caco2	Digoxin	Pgp_Ratios	Human_physiology_ADAM	Human_phys			Dose_IV_injection_mg	Dose_PO_mg
5	Digoxin_Caco2	Digoxin	Pgp_Ratios	Human_physiology_ADAM	Human_phys			Dose_IV_injection_mg	Dose_PO_mg
6	Digoxin_Caco2	Digoxin	Pgp_Ratios	Human_physiology_ADAM	Human_phys			Dose_IV_injection_mg	Dose_PO_mg
7	Digoxin_Caco2	Digoxin	Pgp_Ratios	Human_physiology_ADAM	Human_phys			Dose_IV_injection_mg	Dose_PO_mg
8	Digoxin_Caco2	Digoxin	Pgp_Ratios	Human_physiology_ADAM	Human_phys			Dose_IV_injection_mg	Dose_PO_mg
9	Digoxin_Caco2	Digoxin	Pgp_Ratios	Human_physiology_ADAM	Human_phys			Dose_IV_injection_mg	Dose_PO_mg
10	Digoxin_Caco2	Digoxin	Pgp_Ratios	Human_physiology_ADAM	Human_phys			Dose_IV_injection_mg	Dose_PO_mg
11	Digoxin_Caco2	Digoxin	Pgp_Ratios	Human_physiology_ADAM	Human_phys			Dose_IV_injection_mg	Dose_PO_mg

Fit Step

Estimated Parameters (Pooled Fit)

Name	Transformation	Initial Untransformed Value	Untransformed Bounds
switchVmax_efflux_baso	log	3.049e-09	[3.049e-10 0.03]

Error Model

Use one common error model for all responses: proportional

Algorithm Settings

Property	Value
EstimationFcn	scattersearch
MaxIterations	400
FunctionTolerance	1e-08
MaxStallIterations	50
MaxTime	Inf
NumInitialPoints	300
NumTrialPoints	auto
XTolerance	1e-06
LocalSolver	lsqnonlin

Local Solver Settings

Property	Value
StepTolerance	1e-08
FunctionTolerance	1e-08
OptimalityTolerance	1e-06
MaxIterations	400

Program Results

Fit Step

Pooled Parameter Estimates

Name	Estimate	StandardError
switchVmax_efflux_baso	0.0064738	0.0019437

Statistics

Name	Value
AIC	364.6947
BIC	367.7636
LogLikelihood	-181.3473
DFE	158
MSE	9.6677
SSE	1527.4936

Pooled Beta

Name	Estimate	StandardError
log(switchVmax_efflux_baso)	-5.04	0.30024

Residuals

Group	Time_hr	Conc_ctrl_ng_ml
1	0	NaN
1	0.25	0.23147
1	0.5	0.12291
1	0.75	0.058227
1	1	0.032497
1	1.5	-0.22366
1	2	-0.18705
1	2.5	-0.29267
1	3	-0.24368
1	4	-0.20848
1	6	-0.070324
1	9	0.046843
1	12	0.089631
1	16	0.058159
2	0	NaN
2	0.033333	-15.0022
2	0.066667	-6.1917
2	0.1	-9.3654
2	0.13333	-5.286
2	0.16667	-4.0302
2	0.23333	-8.823
2	0.3	-7.4533
2	0.36667	-6.1125
2	0.5	-4.4757
2	0.75	-3.8755
2	1	-2.986
2	2	-0.82117
2	3	-0.32161
2	4	-0.1875
2	5	0.2805
2	8	0.34204
2	12	0.49899
2	16	0.42685
2	24	0.29345
2	36	0.31566
2	48	0.26405
2	72	0.12146
2	96	0.0029351
2	169	NaN
2	240	NaN

Group	Time_hr	Conc_ctrl_ng_ml
3	0	NaN
3	0.25	0.5494
3	0.5	-0.33706
3	0.75	-0.66688
3	1	-0.36747
3	1.5	-0.1496
3	2	0.16382
3	3	0.13418
3	4	-0.011644
3	5	0.12362
3	6	0.12344
3	8	0.016629
3	12	0.032529
3	16	-0.029016
3	24	-0.015506
4	0	NaN
4	0.17	0.35727
4	0.33	-3.2939
4	0.5	-7.8365
4	0.58	-8.5242
4	0.67	-8.5302
4	0.75	-7.5616
4	1	-5.0139
4	1.25	-3.7002
4	1.5	-2.796
4	2	-1.5804
4	3	-0.29891
4	5	0.17605
4	7	0.25203
4	240	NaN
5	0	NaN
5	0.17	-0.87701
5	0.33	-2.0376
5	0.5	-2.004
5	0.58	-2.0589
5	0.67	-1.861
5	0.75	-1.6563
5	1	-1.1001
5	1.25	-0.54144
5	1.5	-0.28023
5	2	0.14249

Group	Time_hr	Conc_ctrl_ng_ml
5	3	0.37994
5	5	0.26065
5	7	0.2411
6	0	NaN
6	0.25	-0.19976
6	0.5	-0.30307
6	0.75	-0.26587
6	1	-0.33824
6	1.5	-0.24932
6	2	-0.23746
6	4	-0.23425
6	6	-0.054269
6	8	-0.010378
6	12	0.054831
6	24	-0.0095391
6	48	0.019792
7	0	NaN
7	0.25	-0.047338
7	0.5	-0.5594
7	0.75	-0.94645
7	1	-0.86987
7	1.5	-0.78243
7	2	-0.59632
7	4	-0.42077
7	6	-0.23378
7	8	-0.15432
7	12	-0.15296
7	24	-0.16958
7	48	-0.053262
8	0	NaN
8	0.5	-0.30213
8	1	-0.48404
8	1.5	-0.30942
8	2	-0.18271
8	2.5	-0.1408
8	3	-0.07089
8	3.5	-0.025554
8	4	-0.0025215
8	5	0.0048864
8	6	0.034648
8	8	-0.0075699

Group	Time_hr	Conc_ctrl_ng_ml
8	10	0.0037642
8	14	0.011454
9	0	NaN
9	0.16667	0.30606
9	0.33333	-0.10125
9	0.5	-0.36479
9	0.66667	-0.40191
9	0.83333	-0.39693
9	1	-0.3236
9	1.25	-0.34787
9	1.5	-0.35859
9	2	-0.07278
9	2.5	0.031113
9	3	0.041622
9	4	0.063438
9	6	0.18172
9	8	0.35085
9	10	0.35024
9	12	0.3656
9	24	0.31349
10	0	NaN
10	0.033333	21.1079
10	0.16667	-2.9423
10	0.25	-6.7742
10	0.33333	-5.1917
10	0.5	-4.5422
10	0.75	-2.4729
10	1	-1.9567
10	1.25	-1.1041
10	1.5	-0.91207
10	2	-0.36141
10	3	-0.059992
10	4	0.30134
10	6	0.38236
10	8	0.31614
10	10	0.48619
10	240	NaN
11	0	NaN
11	0.33333	0.67958
11	0.5	0.54165
11	0.66667	0.85829

Group	Time_hr	Conc_ctrl_ng_ml
11	0.83333	0.61394
11	1	0.6504
11	1.25	0.28393
11	1.5	0.14702
11	2	0.010433
11	2.5	-0.063989
11	3	-0.011174
11	4	0.034425
11	6	0.20455
11	8	0.32887
11	10	0.36676

Covariance Matrix

Name	switchVmax_efflux_baso
switchVmax_efflux_baso	3.778e-06

Error Model

Response	ErrorModel	b
Conc_ctrl_ng_ml	proportional	0.52223

Report generated by SimBiology v. 25.1 (R2025a) on 13-Sep-2025 12:26:24

Model: PBPK_MADAM

Quantities

	Quantity Name	Type	Scope	Value	Initial Value	Units
1	Main_compartment	compartment	PBPK_MADAM	1	1	liter
2	Bile_drug	species	Main_compartment	0	0	milligram
3	Venous	compartment	Main_compartment	1	3.598	liter
4	Venous_drug	species	Venous	0	0	milligram/liter
5	Lung	compartment	Main_compartment	1	0.56	liter
6	Lung_drug	species	Lung	0	0	milligram/liter
7	Kidney	compartment	Main_compartment	1	0.35	liter
8	Kidney_drug	species	Kidney	0	0	milligram/liter
9	Brain	compartment	Main_compartment	1	1.47	liter
10	Brain_drug	species	Brain	0	0	milligram/liter
11	Muscle	compartment	Main_compartment	1	29.12	liter
12	Muscle_drug	species	Muscle	0	0	milligram/liter
13	Adipose	compartment	Main_compartment	1	13.79	liter
14	Adipose_drug	species	Adipose	0	0	milligram/liter
15	Heart	compartment	Main_compartment	1	0.35	liter
16	Heart_drug	species	Heart	0	0	milligram/liter
17	Skin	compartment	Main_compartment	1	2.87	liter
18	Skin_drug	species	Skin	0	0	milligram/liter
19	Bone	compartment	Main_compartment	1	11.06	liter
20	Bone_drug	species	Bone	0	0	milligram/liter
21	Rest	compartment	Main_compartment	1	7	liter
22	Rest_drug	species	Rest	0	0	milligram/liter
23	Artery	compartment	Main_compartment	1	1.799	liter

	Quantity Name	Type	Scope	Value	Initial Value	Units
24	Artery_drug	species	Artery	0	0	milligram/liter
25	Gut	compartment	Main_compartment	1	1.26	liter
26	Gut_drug	species	Gut	0	0	milligram/liter
27	Spleen	compartment	Main_compartment	1	0.21	liter
28	Spleen_drug	species	Spleen	0	0	milligram/liter
29	Liver_EC_S1	compartment	Main_compartment	1	0.0686	liter
30	Liver_EC_S1_drug	species	Liver_EC_S1	0	0	milligram/liter
31	Liver_EC_S2	compartment	Main_compartment	1	0.0686	liter
32	Liver_EC_S2_drug	species	Liver_EC_S2	0	0	milligram/liter
33	Liver_EC_S3	compartment	Main_compartment	1	0.0686	liter
34	Liver_EC_S3_drug	species	Liver_EC_S3	0	0	milligram/liter
35	Liver_EC_S4	compartment	Main_compartment	1	0.0686	liter
36	Liver_EC_S4_drug	species	Liver_EC_S4	0	0	milligram/liter
37	Liver_EC_S5	compartment	Main_compartment	1	0.0686	liter
38	Liver_EC_S5_drug	species	Liver_EC_S5	0	0	milligram/liter
39	Liver_IC_S5	compartment	Main_compartment	1	0.252	liter
40	Liver_IC_S5_drug	species	Liver_IC_S5	0	0	milligram/liter
41	Liver_IC_S4	compartment	Main_compartment	1	0.252	liter
42	Liver_IC_S4_drug	species	Liver_IC_S4	0	0	milligram/liter
43	Liver_IC_S3	compartment	Main_compartment	1	0.252	liter
44	Liver_IC_S3_drug	species	Liver_IC_S3	0	0	milligram/liter
45	Liver_IC_S1	compartment	Main_compartment	1	0.252	liter
46	Liver_IC_S1_drug	species	Liver_IC_S1	0	0	milligram/liter

	Quantity Name	Type	Scope	Value	Initial Value	Units
47	Liver_IC_S2	compartment	Main_compartment	1	0.252	liter
48	Liver_IC_S2_drug	species	Liver_IC_S2	0	0	milligram/liter
49	Metabolites	compartment	Main_compartment	1	1	liter
50	Metabolites_drug	species	Metabolites	0	0	milligram
51	Testes	compartment	Main_compartment	1	0.07	liter
52	Testes_drug	species	Testes	0	0	milligram/liter
53	Blood_total	compartment	Main_compartment	1	1	liter
54	Blood_total_drug	species	Blood_total	0	0	milligram/liter
55	Plasma_total	compartment	Main_compartment	1	1	liter
56	Plasma_total_drug	species	Plasma_total	0	0	nanogram/milliliter
57	Plasma_free_uM	species	Plasma_total	0	0	micromole/liter
58	Plasma_total_uM	species	Plasma_total	0	0	micromole/liter
59	Portal	compartment	Main_compartment	1	1	liter
60	Portal_drug	species	Portal	0	0	milligram/liter
61	Mass_Balance	compartment	Main_compartment	1	1	liter
62	Amount_body	species	Mass_Balance	0	0	milligram
63	Amount_total	species	Mass_Balance	0	0	milligram
64	Urine	compartment	Main_compartment	1	1	liter
65	Urine_drug	species	Urine	0	0	milligram
66	Liver_total	compartment	Main_compartment	1	1	liter
67	Liver_tissue_total	species	Liver_total	0	0	milligram/liter
68	Liver_blood_total	species	Liver_total	0	0	milligram/liter
69	Liver_blood_free	species	Liver_total	0	0	milligram/liter
70	Liver_tissue_free	species	Liver_total	0	0	milligram/liter

	Quantity Name	Type	Scope	Value	Initial Value	Units
71	Liver_tissue_total_uM	species	Liver_total	0	0	micromole/liter
72	convert_to_nmole_per_kg	compartment	Main_compartment	1	1	liter
73	Venous_nmole	species	convert_to_nmole_per_kg	0	0	nanomole
74	Artery_nmole	species	convert_to_nmole_per_kg	0	0	nanomole
75	Bone_nmole	species	convert_to_nmole_per_kg	0	0	nanomole
76	Adipose_nmole	species	convert_to_nmole_per_kg	0	0	nanomole
77	Muscle_nmole	species	convert_to_nmole_per_kg	0	0	nanomole
78	Urine_nmole	species	convert_to_nmole_per_kg	0	0	nanomole
79	Liver_EC1_nmole	species	convert_to_nmole_per_kg	0	0	nanomole
80	Liver_IC1_nmole	species	convert_to_nmole_per_kg	0	0	nanomole
81	Bile_nmole	species	convert_to_nmole_per_kg	0	0	nanomole
82	Kidney_nmole	species	convert_to_nmole_per_kg	0	0	nanomole
83	Lung_nmole	species	convert_to_nmole_per_kg	0	0	nanomole
84	Metabolites_nmole	species	convert_to_nmole_per_kg	0	0	nanomole
85	Liver_IC2_nmole	species	convert_to_nmole_per_kg	0	0	nanomole
86	Liver_EC2_nmole	species	convert_to_nmole_per_kg	0	0	nanomole
87	Liver_EC3_nmole	species	convert_to_nmole_per_	0	0	nanomole

	Quantity Name	Type	Scope	Value	Initial Value	Units
			kg			
88	Liver_IC3_nmole	species	convert_to_nmole_per_kg	0	0	nanomole
89	Liver_IC4_nmole	species	convert_to_nmole_per_kg	0	0	nanomole
90	Liver_EC4_nmole	species	convert_to_nmole_per_kg	0	0	nanomole
91	Liver_EC5_nmole	species	convert_to_nmole_per_kg	0	0	nanomole
92	Liver_IC5_nmole	species	convert_to_nmole_per_kg	0	0	nanomole
93	Gut_nmole	species	convert_to_nmole_per_kg	0	0	nanomole
94	Spleen_nmole	species	convert_to_nmole_per_kg	0	0	nanomole
95	Skin_nmole	species	convert_to_nmole_per_kg	0	0	nanomole
96	Brain_nmole	species	convert_to_nmole_per_kg	0	0	nanomole
97	Rest_nmole	species	convert_to_nmole_per_kg	0	0	nanomole
98	Heart_nmole	species	convert_to_nmole_per_kg	0	0	nanomole
99	X_CECUM_DISS_nmole	species	convert_to_nmole_per_kg	0	0	nanomole
100	Main_compartment_1	compartment	PBPK_MADAM	1	1	liter
101	Bile_drug_1	species	Main_compartment_1	0	0	milligram
102	Venous_1	compartment	Main_compartment_1	1	3.598	liter
103	Venous_drug_1	species	Venous_1	0	0	milligram/liter
104	Lung_1	compartment	Main_compartment_1	1	0.56	liter

	Quantity Name	Type	Scope	Value	Initial Value	Units
105	Lung_drug_1	species	Lung_1	0	0	milligram/liter
106	Kidney_1	compartment	Main_compartment_1	1	0.35	liter
107	Kidney_drug_1	species	Kidney_1	0	0	milligram/liter
108	Brain_1	compartment	Main_compartment_1	1	1.47	liter
109	Brain_drug_1	species	Brain_1	0	0	milligram/liter
110	Muscle_1	compartment	Main_compartment_1	1	29.12	liter
111	Muscle_drug_1	species	Muscle_1	0	0	milligram/liter
112	Adipose_1	compartment	Main_compartment_1	1	13.79	liter
113	Adipose_drug_1	species	Adipose_1	0	0	milligram/liter
114	Heart_1	compartment	Main_compartment_1	1	0.35	liter
115	Heart_drug_1	species	Heart_1	0	0	milligram/liter
116	Skin_1	compartment	Main_compartment_1	1	2.87	liter
117	Skin_drug_1	species	Skin_1	0	0	milligram/liter
118	Bone_1	compartment	Main_compartment_1	1	11.06	liter
119	Bone_drug_1	species	Bone_1	0	0	milligram/liter
120	Rest_1	compartment	Main_compartment_1	1	7	liter
121	Rest_drug_1	species	Rest_1	0	0	milligram/liter
122	Artery_1	compartment	Main_compartment_1	1	1.799	liter
123	Artery_drug_1	species	Artery_1	0	0	milligram/liter
124	Gut_1	compartment	Main_compartment_1	1	1.26	liter
125	Gut_drug_1	species	Gut_1	0	0	milligram/liter
126	Spleen_1	compartment	Main_compartment_1	1	0.21	liter
127	Spleen_drug_1	species	Spleen_1	0	0	milligram/liter

	Quantity Name	Type	Scope	Value	Initial Value	Units
128	Liver_EC_S1_1	compartment	Main_compartment_1	1	0.0686	liter
129	Liver_EC_S1_drug_1	species	Liver_EC_S1_1	0	0	milligram/liter
130	Liver_EC_S2_1	compartment	Main_compartment_1	1	0.0686	liter
131	Liver_EC_S2_drug_1	species	Liver_EC_S2_1	0	0	milligram/liter
132	Liver_EC_S3_1	compartment	Main_compartment_1	1	0.0686	liter
133	Liver_EC_S3_drug_1	species	Liver_EC_S3_1	0	0	milligram/liter
134	Liver_EC_S4_1	compartment	Main_compartment_1	1	0.0686	liter
135	Liver_EC_S4_drug_1	species	Liver_EC_S4_1	0	0	milligram/liter
136	Liver_EC_S5_1	compartment	Main_compartment_1	1	0.0686	liter
137	Liver_EC_S5_drug_1	species	Liver_EC_S5_1	0	0	milligram/liter
138	Liver_IC_S5_1	compartment	Main_compartment_1	1	0.252	liter
139	Liver_IC_S5_drug_1	species	Liver_IC_S5_1	0	0	milligram/liter
140	Liver_IC_S3_1	compartment	Main_compartment_1	1	0.252	liter
141	Liver_IC_S3_drug_1	species	Liver_IC_S3_1	0	0	milligram/liter
142	Liver_IC_S1_1	compartment	Main_compartment_1	1	0.252	liter
143	Liver_IC_S1_drug_1	species	Liver_IC_S1_1	0	0	milligram/liter
144	Liver_IC_S2_1	compartment	Main_compartment_1	1	0.252	liter
145	Liver_IC_S2_drug_1	species	Liver_IC_S2_1	0	0	milligram/liter
146	Metabolites_1	compartment	Main_compartment_1	1	1	liter
147	Metabolites_drug_1	species	Metabolites_1	0	0	milligram
148	Testes_1	compartment	Main_compartment_1	1	0.07	liter
149	Testes_drug_1	species	Testes_1	0	0	milligram/liter

	Quantity Name	Type	Scope	Value	Initial Value	Units
150	Blood_total_1	compartment	Main_compartment_1	1	1	liter
151	Blood_total_drug_1	species	Blood_total_1	0	0	milligram/liter
152	Plasma_total_1	compartment	Main_compartment_1	1	1	liter
153	Plasma_total_drug_1	species	Plasma_total_1	0	0	nanogram/milliliter
154	Plasma_total_uM_1	species	Plasma_total_1	0	0	micromole/liter
155	Plasma_free_uM_1	species	Plasma_total_1	0	0	micromole/liter
156	Portal_1	compartment	Main_compartment_1	1	1	liter
157	Portal_drug_1	species	Portal_1	0	0	milligram/liter
158	Portal_plasma_drug_1	species	Portal_1	0	0	milligram/liter
159	Portal_plasma_drug	species	Portal_1	0	0	milligram/liter
160	Mass_Balance_1	compartment	Main_compartment_1	1	1	liter
161	Amount_body_1	species	Mass_Balance_1	0	0	milligram
162	Amount_total_1	species	Mass_Balance_1	0	0	milligram
163	Urine_1	compartment	Main_compartment_1	1	1	liter
164	Urine_drug_1	species	Urine_1	0	0	milligram
165	Liver_IC_S4_1	compartment	Main_compartment_1	1	0.252	liter
166	Liver_IC_S4_drug_1	species	Liver_IC_S4_1	0	0	milligram/liter
167	Liver_total_1	compartment	Main_compartment_1	1	1	liter
168	Liver_tissue_free_uM_1	species	Liver_total_1	0	0	micromole/liter
169	Liver_tissue_total_1	species	Liver_total_1	0	0	milligram/liter
170	Liver_blood_free_1	species	Liver_total_1	0	0	milligram/liter
171	Liver_tissue_total_uM_1	species	Liver_total_1	0	0	micromole/liter

	Quantity Name	Type	Scope	Value	Initial Value	Units
172	Liver_blood_total_1	species	Liver_total_1	0	0	milligram/liter
173	convert_to_nmole_per_kg_1	compartment	Main_compartment_1	1	1	liter
174	Venous_nmole_1	species	convert_to_nmole_per_kg_1	0	0	nanomole
175	Artery_nmole_1	species	convert_to_nmole_per_kg_1	0	0	nanomole
176	Bone_nmole_1	species	convert_to_nmole_per_kg_1	0	0	nanomole
177	Adipose_nmole_1	species	convert_to_nmole_per_kg_1	0	0	nanomole
178	Muscle_nmole_1	species	convert_to_nmole_per_kg_1	0	0	nanomole
179	Urine_nmole_1	species	convert_to_nmole_per_kg_1	0	0	nanomole
180	Liver_EC1_nmole_1	species	convert_to_nmole_per_kg_1	0	0	nanomole
181	Liver_IC1_nmole_1	species	convert_to_nmole_per_kg_1	0	0	nanomole
182	Bile_nmole_1	species	convert_to_nmole_per_kg_1	0	0	nanomole
183	Kidney_nmole_1	species	convert_to_nmole_per_kg_1	0	0	nanomole
184	Lung_nmole_1	species	convert_to_nmole_per_kg_1	0	0	nanomole
185	Metabolites_nmole_1	species	convert_to_nmole_per_kg_1	0	0	nanomole
186	Liver_IC2_nmole_1	species	convert_to_nmole_per_kg_1	0	0	nanomole
187	Liver_EC2_nmole_1	species	convert_to_nmole_per_kg_1	0	0	nanomole
188	Liver_EC3_nmole_1	species	convert_to_nmole_per_	0	0	nanomole

	Quantity Name	Type	Scope	Value	Initial Value	Units
			kg_1			
189	Liver_IC3_nmole_1	species	convert_to_nmole_per_kg_1	0	0	nanomole
190	Liver_IC4_nmole_1	species	convert_to_nmole_per_kg_1	0	0	nanomole
191	Liver_EC4_nmole_1	species	convert_to_nmole_per_kg_1	0	0	nanomole
192	Liver_EC5_nmole_1	species	convert_to_nmole_per_kg_1	0	0	nanomole
193	Liver_IC5_nmole_1	species	convert_to_nmole_per_kg_1	0	0	nanomole
194	Gut_nmole_1	species	convert_to_nmole_per_kg_1	0	0	nanomole
195	Spleen_nmole_1	species	convert_to_nmole_per_kg_1	0	0	nanomole
196	Skin_nmole_1	species	convert_to_nmole_per_kg_1	0	0	nanomole
197	Brain_nmole_1	species	convert_to_nmole_per_kg_1	0	0	nanomole
198	Rest_nmole_1	species	convert_to_nmole_per_kg_1	0	0	nanomole
199	Heart_nmole_1	species	convert_to_nmole_per_kg_1	0	0	nanomole
200	Gut_Lumen	compartment	Main_compartment	1	1	liter
201	Gut_Lumen_drug	species	Gut_Lumen	0	0	milligram
202	Gut_Lumen_1	compartment	Main_compartment_1	1	1	liter
203	Gut_Lumen_drug_1	species	Gut_Lumen_1	0	0	milligram
204	STOMACH	compartment	Main_compartment	0.147	0.05	liter
205	X_STOMACH_SOLID	species	STOMACH	0	0	microgram
206	X_STOMACH_DISS	species	STOMACH	0	0	microgram

	Quantity Name	Type	Scope	Value	Initial Value	Units
207	VDUO	compartment	Main_compartment	1	0.03435	liter
208	X_DUO_SOLID	species	VDUO	0	0	microgram
209	X_DUO_DISS	species	VDUO	0	0	microgram
210	VJEJ1	compartment	Main_compartment	1	0.0211	liter
211	X_JEJ1_SOLID	species	VJEJ1	0	0	microgram
212	X_JEJ1_DISS	species	VJEJ1	0	0	microgram
213	MDUO	compartment	Main_compartment	1	0.037454	liter
214	MEM_DUO	species	MDUO	0	0	microgram
215	MJEJ1	compartment	Main_compartment	1	0.073785	liter
216	MEM_JEJ1	species	MJEJ1	0	0	microgram
217	VJEJ2	compartment	Main_compartment	1	0.0211	liter
218	X_JEJ2_SOLID	species	VJEJ2	0	0	microgram
219	X_JEJ2_DISS	species	VJEJ2	0	0	microgram
220	MJEJ2	compartment	Main_compartment	1	0.051687	liter
221	MEM_JEJ2	species	MJEJ2	0	0	microgram
222	VILL1	compartment	Main_compartment	1	0.0126	liter
223	X_ILL1_SOLID	species	VILL1	0	0	microgram
224	X_ILL1_DISS	species	VILL1	0	0	microgram
225	MILL1	compartment	Main_compartment	1	0.0412	liter
226	MEM_ILL1	species	MILL1	0	0	microgram
227	VILL2	compartment	Main_compartment	1	0.0126	liter
228	X_ILL2_SOLID	species	VILL2	0	0	microgram
229	X_ILL2_DISS	species	VILL2	0	0	microgram
230	MILL2	compartment	Main_compartment	1	0.0412	liter

	Quantity Name	Type	Scope	Value	Initial Value	Units
231	MEM_ILL2	species	MILL2	0	0	microgram
232	VILL3	compartment	Main_compartment	1	0.0126	liter
233	X_ILL3_SOLID	species	VILL3	0	0	microgram
234	X_ILL3_DISS	species	VILL3	0	0	microgram
235	MILL3	compartment	Main_compartment	1	0.04045	liter
236	MEM_ILL3	species	MILL3	0	0	microgram
237	VILL4	compartment	Main_compartment	1	0.0126	liter
238	X_ILL4_SOLID	species	VILL4	0	0	microgram
239	X_ILL4_DISS	species	VILL4	0	0	microgram
240	MILL4	compartment	Main_compartment	1	0.038952	liter
241	MEM_ILL4	species	MILL4	0	0	microgram
242	Colon	compartment	Main_compartment	1	1	liter
243	X_CECUM_SOLID	species	Colon	0	0	microgram
244	X_CECUM_DISS	species	Colon	0	0	microgram
245	VillousDUO	compartment	Main_compartment	1	0.0057016	liter
246	Villous_DUO	species	VillousDUO	0	0	microgram
247	VillousJEJ1	compartment	Main_compartment	1	0.011232	liter
248	Villous_JEJ1	species	VillousJEJ1	0	0	microgram
249	VillousJEJ2	compartment	Main_compartment	1	0.0078682	liter
250	Villous_JEJ2	species	VillousJEJ2	0	0	microgram
251	VillousILL1	compartment	Main_compartment	1	0.0062718	liter
252	Villous_ILL1	species	VillousILL1	0	0	microgram
253	VillousILL2	compartment	Main_compartment	1	0.0062718	liter
254	Villous_ILL2	species	VillousILL2	0	0	microgram

	Quantity Name	Type	Scope	Value	Initial Value	Units
255	VillousILL3	compartment	Main_compartment	1	0.0061578	liter
256	Villous_ILL3	species	VillousILL3	0	0	microgram
257	VillousILL4	compartment	Main_compartment	1	0.0059297	liter
258	Villous_ILL4	species	VillousILL4	0	0	microgram
259	Liver	compartment	Main_compartment	1	1.603	liter
260	Liver_drug	species	Liver	0	0	milligram/liter
261	Serosa	compartment	Main_compartment	1	0.14284	liter
262	Serosa_drug	species	Serosa	0	0	milligram/liter
263	STOMACH_1	compartment	Main_compartment_1	0.147	0.05	liter
264	X_STOMACH_SOLID_1	species	STOMACH_1	0	0	microgram
265	X_STOMACH_DISS_1	species	STOMACH_1	0	0	microgram
266	VDUO_1	compartment	Main_compartment_1	1	0.03435	liter
267	X_DUO_SOLID_1	species	VDUO_1	0	0	microgram
268	X_DUO_DISS_1	species	VDUO_1	0	0	microgram
269	VJEJ1_1	compartment	Main_compartment_1	1	0.0211	liter
270	X_JEJ1_SOLID_1	species	VJEJ1_1	0	0	microgram
271	X_JEJ1_DISS_1	species	VJEJ1_1	0	0	microgram
272	MDUO_1	compartment	Main_compartment_1	1	0.037454	liter
273	MEM_DUO_1	species	MDUO_1	0	0	microgram
274	MJEJ1_1	compartment	Main_compartment_1	1	0.073785	liter
275	MEM_JEJ1_1	species	MJEJ1_1	0	0	microgram
276	VJEJ2_1	compartment	Main_compartment_1	1	0.0211	liter
277	X_JEJ2_SOLID_1	species	VJEJ2_1	0	0	microgram
278	X_JEJ2_DISS_1	species	VJEJ2_1	0	0	microgram

	Quantity Name	Type	Scope	Value	Initial Value	Units
279	MJEJ2_1	compartment	Main_compartment_1	1	0.051687	liter
280	MEM_JEJ2_1	species	MJEJ2_1	0	0	microgram
281	VILL1_1	compartment	Main_compartment_1	1	0.0126	liter
282	X_ILL1_SOLID_1	species	VILL1_1	0	0	microgram
283	X_ILL1_DISS_1	species	VILL1_1	0	0	microgram
284	MILL1_1	compartment	Main_compartment_1	1	0.0412	liter
285	MEM_ILL1_1	species	MILL1_1	0	0	microgram
286	VILL2_1	compartment	Main_compartment_1	1	0.0126	liter
287	X_ILL2_SOLID_1	species	VILL2_1	0	0	microgram
288	X_ILL2_DISS_1	species	VILL2_1	0	0	microgram
289	MILL2_1	compartment	Main_compartment_1	1	0.0412	liter
290	MEM_ILL2_1	species	MILL2_1	0	0	microgram
291	VILL3_1	compartment	Main_compartment_1	1	0.0126	liter
292	X_ILL3_SOLID_1	species	VILL3_1	0	0	microgram
293	X_ILL3_DISS_1	species	VILL3_1	0	0	microgram
294	MILL3_1	compartment	Main_compartment_1	1	0.04045	liter
295	MEM_ILL3_1	species	MILL3_1	0	0	microgram
296	VILL4_1	compartment	Main_compartment_1	1	0.0126	liter
297	X_ILL4_SOLID_1	species	VILL4_1	0	0	microgram
298	X_ILL4_DISS_1	species	VILL4_1	0	0	microgram
299	MILL4_1	compartment	Main_compartment_1	1	0.038952	liter
300	MEM_ILL4_1	species	MILL4_1	0	0	microgram

	Quantity Name	Type	Scope	Value	Initial Value	Units
301	Colon_1	compartment	Main_compartment_1	1	1	liter
302	X_CECUM_SOLID_1	species	Colon_1	0	0	microgram
303	X_CECUM_DISS_1	species	Colon_1	0	0	microgram
304	VillousDUO_1	compartment	Main_compartment_1	1	0.0057016	liter
305	Villous_DUO_1	species	VillousDUO_1	0	0	microgram
306	VillousJEJ2_1	compartment	Main_compartment_1	1	0.0078682	liter
307	Villous_JEJ2_1	species	VillousJEJ2_1	0	0	microgram
308	VillousILL1_1	compartment	Main_compartment_1	1	0.0062718	liter
309	Villous_ILL1_1	species	VillousILL1_1	0	0	microgram
310	VillousILL2_1	compartment	Main_compartment_1	1	0.0062718	liter
311	Villous_ILL2_1	species	VillousILL2_1	0	0	microgram
312	VillousILL3_1	compartment	Main_compartment_1	1	0.0061578	liter
313	Villous_ILL3_1	species	VillousILL3_1	0	0	microgram
314	VillousILL4_1	compartment	Main_compartment_1	1	0.0059297	liter
315	Villous_ILL4_1	species	VillousILL4_1	0	0	microgram
316	VillousJEJ1_1	compartment	Main_compartment_1	1	0.011232	liter
317	Villous_JEJ1_1	species	VillousJEJ1_1	0	0	microgram
318	Liver_1	compartment	Main_compartment_1	1	1.603	liter
319	Liver_drug_1	species	Liver_1	0	0	milligram/liter
320	Serosa_1	compartment	Main_compartment_1	1	0.14284	liter
321	Serosa_drug_1	species	Serosa_1	0	0	milligram/liter
322	Qlung	parameter	PBPK_MADAM	1	336	liter/hour

	Quantity Name	Type	Scope	Value	Initial Value	Units
323	k_lung_artery	parameter	PBPK_MADAM	1	112.8085	liter/hour
324	k_venous_lung	parameter	PBPK_MADAM	1	336	liter/hour
325	Qkidney	parameter	PBPK_MADAM	1	63	liter/hour
326	k_artery_kidney	parameter	PBPK_MADAM	1	63	liter/hour
327	Qbrain	parameter	PBPK_MADAM	1	42	liter/hour
328	k_artery_brain	parameter	PBPK_MADAM	1	42	liter/hour
329	Qmuscle	parameter	PBPK_MADAM	1	58.8	liter/hour
330	k_artery_muscle	parameter	PBPK_MADAM	1	58.8	liter/hour
331	Qadipose	parameter	PBPK_MADAM	1	16.8	liter/hour
332	k_artery_adipos	parameter	PBPK_MADAM	1	16.8	liter/hour
333	Qskin	parameter	PBPK_MADAM	1	16.8	liter/hour
334	k_artery_skin	parameter	PBPK_MADAM	1	16.8	liter/hour
335	Qbone	parameter	PBPK_MADAM	1	16.8	liter/hour
336	k_artery_bone	parameter	PBPK_MADAM	1	16.8	liter/hour
337	Qrest	parameter	PBPK_MADAM	1	4.2	liter/hour
338	k_artery_rest	parameter	PBPK_MADAM	1	4.2	liter/hour
339	k_kidney_venous	parameter	PBPK_MADAM	1	21.3472	liter/hour
340	k_brain_venous	parameter	PBPK_MADAM	1	57.3582	liter/hour
341	k_muscle_venous	parameter	PBPK_MADAM	1	30.5359	liter/hour
342	k_adipos_venous	parameter	PBPK_MADAM	1	25.3489	liter/hour
343	Qheart	parameter	PBPK_MADAM	1	12.6	liter/hour
344	k_heart_venous	parameter	PBPK_MADAM	1	3.5143	liter/hour

	Quantity Name	Type	Scope	Value	Initial Value	Units
345	k_skin_venous	parameter	PBPK_MADAM	1	11.8896	liter/hour
346	k_bone_venous	parameter	PBPK_MADAM	1	16.87	liter/hour
347	k_rest_venous	parameter	PBPK_MADAM	1	1.6425	liter/hour
348	Q_artery_spleen	parameter	PBPK_MADAM	1	8.4	liter/hour
349	k_artery_spleen	parameter	PBPK_MADAM	1	8.4	liter/hour
350	Q_artery_gut	parameter	PBPK_MADAM	1	71.4	liter/hour
351	k_artery_gut	parameter	PBPK_MADAM	1	71.4	liter/hour
352	Q_artery_liver	parameter	PBPK_MADAM	1	4.2	liter/hour
353	Q_spleen_liver	parameter	PBPK_MADAM	1	8.4	liter/hour
354	k_spleen_liver	parameter	PBPK_MADAM	1	4.4881	liter/hour
355	Q_gut_liver	parameter	PBPK_MADAM	1	71.4	liter/hour
356	k_gut_liver	parameter	PBPK_MADAM	1	20.9108	liter/hour
357	k_Liver_EC_S1_Liver_IC_S1	parameter	PBPK_MADAM	1	513.8127	liter/hour
358	k_Liver_IC_S1_Liver_EC_S1	parameter	PBPK_MADAM	0	0.15452	liter/hour
359	k_Liver_EC_S2_Liver_IC_S2	parameter	PBPK_MADAM	1	513.8127	liter/hour
360	k_Liver_IC_S2_Liver_EC_S2	parameter	PBPK_MADAM	0	0.15452	liter/hour
361	k_Liver_IC_S4_Liver_EC_S4	parameter	PBPK_MADAM	0	0.15452	liter/hour
362	k_Liver_EC_S4_Liver_IC_S4	parameter	PBPK_MADAM	1	513.8127	liter/hour
363	k_Liver_IC_S5_Liver_EC_S5	parameter	PBPK_MADAM	0	0.15452	liter/hour
364	k_Liver_EC_S5_Liver_I	parameter	PBPK_MADAM	1	513.8127	liter/hour

	Quantity Name	Type	Scope	Value	Initial Value	Units
	C_S5					
365	k_Liver_EC_S3_Liver_IC_S3	parameter	PBPK_MADAM	1	513.8127	liter/hour
366	Q_li	parameter	PBPK_MADAM	1	84	liter/hour
367	k_Liver_EC_S1_Liver_EC_S2	parameter	PBPK_MADAM	1	84	liter/hour
368	k_Liver_EC_S2_Liver_EC_S3	parameter	PBPK_MADAM	1	84	liter/hour
369	k_Liver_EC_S3_Liver_EC_S4	parameter	PBPK_MADAM	1	84	liter/hour
370	k_Liver_EC_S4_Liver_EC_S5	parameter	PBPK_MADAM	1	84	liter/hour
371	k_Liver_IC_S5_Bile	parameter	PBPK_MADAM	1	0	liter/hour
372	k_Liver_IC_S4_Bile	parameter	PBPK_MADAM	1	0	liter/hour
373	k_Liver_IC_S3_Bile	parameter	PBPK_MADAM	1	0	liter/hour
374	k_Liver_IC_S2_Bile	parameter	PBPK_MADAM	1	0	liter/hour
375	k_Liver_IC_S1_Bile	parameter	PBPK_MADAM	1	0	liter/hour
376	k_Liver_IC_S1_Metabolites	parameter	PBPK_MADAM	1	0.7973	liter/hour
377	k_Liver_IC_S2_Metabolites	parameter	PBPK_MADAM	1	0.7973	liter/hour
378	k_Liver_IC_S3_Metabolites	parameter	PBPK_MADAM	1	0.7973	liter/hour
379	k_Liver_IC_S4_Metabolites	parameter	PBPK_MADAM	1	0.7973	liter/hour
380	k_Liver_IC_S5_Metabolites	parameter	PBPK_MADAM	1	0.7973	liter/hour
381	drug_fB	parameter	PBPK_MADAM	1	0.18	dimensionless
382	drug_fuLiver	parameter	PBPK_MADAM	0.018	0.016351	dimensionless

	Quantity Name	Type	Scope	Value	Initial Value	Units
383	Kp_kidney	parameter	PBPK_MADAM	0.134	2.9512	dimensionless
384	drug_BRP	parameter	PBPK_MADAM	0.65	1	dimensionless
385	Kp_heart	parameter	PBPK_MADAM	0.16	3.5854	dimensionless
386	Kp_gut	parameter	PBPK_MADAM	0.165	3.4145	dimensionless
387	Kp_brain	parameter	PBPK_MADAM	0.057	0.73224	dimensionless
388	Kp_bone	parameter	PBPK_MADAM	0.108	0.99585	dimensionless
389	Kp_adipose	parameter	PBPK_MADAM	0.047	0.66275	dimensionless
390	Kp_muscle	parameter	PBPK_MADAM	0.038	1.9256	dimensionless
391	Kp_rest	parameter	PBPK_MADAM	0.12	2.557	dimensionless
392	Kp_lung	parameter	PBPK_MADAM	0.21	2.9785	dimensionless
393	drug_CLrenal	parameter	PBPK_MADAM	0	7.2	liter/hour
394	drug_fuplasma	parameter	PBPK_MADAM	0.0382	0.18	dimensionless
395	Kp_spleen	parameter	PBPK_MADAM	0.1	1.8716	dimensionless
396	k_Liver_Venous	parameter	PBPK_MADAM	1	14.1602	liter/hour
397	k_artery_heart	parameter	PBPK_MADAM	1	12.6	liter/hour
398	k_Liver_IC_S3_Liver__EC_S3	parameter	PBPK_MADAM	0	0.15452	liter/hour
399	k_artery_liver	parameter	PBPK_MADAM	1	4.2	liter/hour
400	drug_PSinfg	parameter	PBPK_MADAM	471	471	microliter/minute
401	phys_HPGL	parameter	PBPK_MADAM	122	125	1/gram
402	switch_SFinf	parameter	PBPK_MADAM	3.196	3.196	dimensionless
403	Specific_volume	parameter	PBPK_MADAM	1	1	milliliter/gram
404	drug_PSbileg	parameter	PBPK_MADAM	2.5	0	microliter/minute

	Quantity Name	Type	Scope	Value	Initial Value	Units
405	switch_SFbile	parameter	PBPK_MADAM	0.23114	1	dimensionless
406	drug_PSdifg	parameter	PBPK_MADAM	5	5	microliter/minute
407	switch_SFdiff	parameter	PBPK_MADAM	1.5621	1	dimensionless
408	drug_CLmetg	parameter	PBPK_MADAM	19	0	microliter/minute
409	drug_HLM_CLint	parameter	PBPK_MADAM	0	0.0436	milliliter/minute/milligram
410	phys_MPGL	parameter	PBPK_MADAM	45	45	milligram/gram
411	drug_fumic	parameter	PBPK_MADAM	1	0.60838	dimensionless
412	drug_funic	parameter	PBPK_MADAM	1	0.60838	dimensionless
413	Kp_skin	parameter	PBPK_MADAM	0.28	1.413	dimensionless
414	phys_BW	parameter	PBPK_MADAM	1	70	kilogram
415	switch_SFrenal	parameter	PBPK_MADAM	1	1	dimensionless
416	k_venous_urine_CLR	parameter	PBPK_MADAM	1	7.2	liter/hour
417	Qtestes	parameter	PBPK_MADAM	1	0	liter/hour
418	k_artery_testes	parameter	PBPK_MADAM	1	0	liter/hour
419	Kp_testes	parameter	PBPK_MADAM	1	1	dimensionless
420	k_testes_venous	parameter	PBPK_MADAM	1	0	liter/hour
421	k_Liver_IC_S2_Liver_EC_S2_efflux	parameter	PBPK_MADAM	1	0	liter/hour
422	k_Liver_IC_S3_Liver_EC_S3_efflux	parameter	PBPK_MADAM	1	0	liter/hour
423	k_Liver_IC_S4_Liver_EC_S4_efflux	parameter	PBPK_MADAM	1	0	liter/hour
424	k_Liver_IC_S5_Liver_EC_S5_efflux	parameter	PBPK_MADAM	1	0	liter/hour

	Quantity Name	Type	Scope	Value	Initial Value	Units
425	k_Liver_IC_S1_Liver_EC_S1_efflux	parameter	PBPK_MADAM	1	0	liter/hour
426	drug_fa	parameter	PBPK_MADAM	1	1	dimensionless
427	drug_dose_rate_IV	parameter	PBPK_MADAM	1	1	milligram/hour
428	phys_Normalized_weight_adipose	parameter	PBPK_MADAM	92	197	gram/kilogram
429	phys_Normalized_weight_lung	parameter	PBPK_MADAM	5	8	gram/kilogram
430	phys_Normalized_weight_kidney	parameter	PBPK_MADAM	4	5	gram/kilogram
431	phys_Normalized_weight_brain	parameter	PBPK_MADAM	21	21	gram/kilogram
432	phys_Normalized_weight_muscle	parameter	PBPK_MADAM	409	416	gram/kilogram
433	phys_Normalized_weight_heart	parameter	PBPK_MADAM	4	5	gram/kilogram
434	phys_Normalized_weight_skin	parameter	PBPK_MADAM	85	41	gram/kilogram
435	phys_Normalized_weight_bone	parameter	PBPK_MADAM	203	158	gram/kilogram
436	phys_Normalized_weight_remainder	parameter	PBPK_MADAM	100	100	gram/kilogram
437	phys_Normalized_weight_spleen	parameter	PBPK_MADAM	2	3	gram/kilogram
438	phys_Normalized_weight_gut	parameter	PBPK_MADAM	47	18	gram/kilogram
439	phys_Normalized_weight_liver_blood	parameter	PBPK_MADAM	5	4.9	gram/kilogram
440	phys_Normalized_weight_liver_tissue	parameter	PBPK_MADAM	20	18	gram/kilogram

	Quantity Name	Type	Scope	Value	Initial Value	Units
441	phys_Normalized_weight_artery	parameter	PBPK_MADAM	22.4	25.7	gram/kilogram
442	phys_Normalized_weight_venous	parameter	PBPK_MADAM	45.2	51.4	gram/kilogram
443	phys_Normalized_weight_testes	parameter	PBPK_MADAM	1	1	gram/kilogram
444	phys_Normalized_Q_adipose	parameter	PBPK_MADAM	15	4	milliliter/minute/kilogram
445	phys_Normalized_Q_lung	parameter	PBPK_MADAM	200	80	milliliter/minute/kilogram
446	phys_Normalized_Q_brain	parameter	PBPK_MADAM	11	10	milliliter/minute/kilogram
447	phys_Normalized_Q_muscle	parameter	PBPK_MADAM	65	14	milliliter/minute/kilogram
448	phys_Normalized_Q_heart	parameter	PBPK_MADAM	12	3	milliliter/minute/kilogram
449	phys_Normalized_Q_bone	parameter	PBPK_MADAM	19	4	milliliter/minute/kilogram
450	phys_Normalized_Q_remainder	parameter	PBPK_MADAM	1	1	milliliter/minute/kilogram
451	phys_Normalized_Q_gut	parameter	PBPK_MADAM	17	17	milliliter/minute/kilogram
452	phys_Normalized_Q_spleen	parameter	PBPK_MADAM	2	2	milliliter/minute/kilogram
453	phys_Normalized_Q_liver	parameter	PBPK_MADAM	26	20	milliliter/minute/kilogram
454	phys_Normalized_Q_kidney	parameter	PBPK_MADAM	25	15	milliliter/minute/kilogram
455	phys_Normalized_Q_skin	parameter	PBPK_MADAM	20	4	milliliter/minute/kilogram
456	phys_Normalized_Q_testes	parameter	PBPK_MADAM	0	0	milliliter/minute/kilogram

	Quantity Name	Type	Scope	Value	Initial Value	Units
457	switch_SFmet	parameter	PBPK_MADAM	0.32509	1	dimensionless
458	drug_Km_uptake	parameter	PBPK_MADAM	1	1	micromole/liter
459	switch_Vmax_uptake	parameter	PBPK_MADAM	0	0	micromole/kilogram/hour
460	drug_molar_mass	parameter	PBPK_MADAM	712800	747950	milligram/mole
461	drug_Km_met	parameter	PBPK_MADAM	1	1	micromole/liter
462	switch_Vmax_met	parameter	PBPK_MADAM	0	0	micromole/hour/kilogram
463	drug_Kp_adipose_raw	parameter	PBPK_MADAM	0.45	0.66275	dimensionless
464	switch_SFKp	parameter	PBPK_MADAM	0.51837	1	dimensionless
465	drug_Kp_bone_raw	parameter	PBPK_MADAM	0.6	0.99585	dimensionless
466	drug_Kp_brain_raw	parameter	PBPK_MADAM	0.32	0.73224	dimensionless
467	drug_Kp_gut_raw	parameter	PBPK_MADAM	0.47	3.4145	dimensionless
468	drug_Kp_heart_raw	parameter	PBPK_MADAM	0.2	3.5854	dimensionless
469	drug_Kp_kidney_raw	parameter	PBPK_MADAM	0.26	2.9512	dimensionless
470	drug_Kp_lung_raw	parameter	PBPK_MADAM	0.4	2.9785	dimensionless
471	drug_Kp_muscle_raw	parameter	PBPK_MADAM	0.05	1.9256	dimensionless
472	drug_Kp_rest_raw	parameter	PBPK_MADAM	0.0065	2.557	dimensionless
473	drug_Kp_skin_raw	parameter	PBPK_MADAM	0.37	1.413	dimensionless
474	drug_Kp_spleen_raw	parameter	PBPK_MADAM	0.35	1.8716	dimensionless
475	drug_Kp_testes_raw	parameter	PBPK_MADAM	1	1	dimensionless
476	k_transit	parameter	PBPK_MADAM	0	0	1/(hour)
477	drug_t_lag	parameter	PBPK_MADAM	1	1	hour
478	drug_k_oral	parameter	PBPK_MADAM	3.381	3.381	1/(hour)

	Quantity Name	Type	Scope	Value	Initial Value	Units
479	switch_SFeff	parameter	PBPK_MADAM	0	0	dimensionless
480	nanomole_per_mole	parameter	PBPK_MADAM	1000000000	1000000000	nanomole/mole
481	kilogram	parameter	PBPK_MADAM	1	1	kilogram
482	k_venous_urine_GFR	parameter	PBPK_MADAM	1	0	liter/hour
483	drug_GFR	parameter	PBPK_MADAM	1	0	milliliter/minute
484	drug_CLeffluxHep	parameter	PBPK_MADAM	0	0	microliter/minute
485	drug_FR	parameter	PBPK_MADAM	0	0	dimensionless
486	switch_slow_dist_Kp	parameter	PBPK_MADAM	1	1	dimensionless
487	Qlung_1	parameter	PBPK_MADAM	1	336	liter/hour
488	k_lung_artery_1	parameter	PBPK_MADAM	1	1053.3017	liter/hour
489	k_venous_lung_1	parameter	PBPK_MADAM	1	336	liter/hour
490	Qkidney_1	parameter	PBPK_MADAM	1	63	liter/hour
491	k_artery_kidney_1	parameter	PBPK_MADAM	1	63	liter/hour
492	Qbrain_1	parameter	PBPK_MADAM	1	42	liter/hour
493	k_artery_brain_1	parameter	PBPK_MADAM	1	42	liter/hour
494	Qmuscle_1	parameter	PBPK_MADAM	1	58.8	liter/hour
495	k_artery_muscle_1	parameter	PBPK_MADAM	1	58.8	liter/hour
496	Qadipose_1	parameter	PBPK_MADAM	1	16.8	liter/hour
497	k_artery_adipos_1	parameter	PBPK_MADAM	1	16.8	liter/hour
498	Qskin_1	parameter	PBPK_MADAM	1	16.8	liter/hour
499	k_artery_skin_1	parameter	PBPK_MADAM	1	16.8	liter/hour
500	Qbone_1	parameter	PBPK_MADAM	1	16.8	liter/hour

	Quantity Name	Type	Scope	Value	Initial Value	Units
501	k_artery_bone_1	parameter	PBPK_MADAM	1	16.8	liter/hour
502	Qrest_1	parameter	PBPK_MADAM	1	4.2	liter/hour
503	k_artery_rest_1	parameter	PBPK_MADAM	1	4.2	liter/hour
504	k_kidney_venous_1	parameter	PBPK_MADAM	1	303.837	liter/hour
505	k_brain_venous_1	parameter	PBPK_MADAM	1	164.5784	liter/hour
506	k_muscle_venous_1	parameter	PBPK_MADAM	1	1474.6224	liter/hour
507	k_adipos_venous_1	parameter	PBPK_MADAM	1	46.8134	liter/hour
508	Qheart_1	parameter	PBPK_MADAM	1	12.6	liter/hour
509	k_heart_venous_1	parameter	PBPK_MADAM	1	78.9976	liter/hour
510	k_skin_venous_1	parameter	PBPK_MADAM	1	56.9352	liter/hour
511	k_bone_venous_1	parameter	PBPK_MADAM	1	35.1101	liter/hour
512	k_rest_venous_1	parameter	PBPK_MADAM	1	420	liter/hour
513	Q_artery_spleen_1	parameter	PBPK_MADAM	1	8.4	liter/hour
514	k_artery_spleen_1	parameter	PBPK_MADAM	1	8.4	liter/hour
515	Q_artery_gut_1	parameter	PBPK_MADAM	1	71.4	liter/hour
516	k_artery_gut_1	parameter	PBPK_MADAM	1	71.4	liter/hour
517	Q_artery_liver_1	parameter	PBPK_MADAM	1	4.2	liter/hour
518	Q_spleen_liver_1	parameter	PBPK_MADAM	1	8.4	liter/hour
519	k_spleen_liver_1	parameter	PBPK_MADAM	1	30.0943	liter/hour
520	Q_gut_liver_1	parameter	PBPK_MADAM	1	71.4	liter/hour
521	k_gut_liver_1	parameter	PBPK_MADAM	1	190.4907	liter/hour
522	k_Liver_EC_S1_Liver_IC_S1_1	parameter	PBPK_MADAM	1	168.0698	liter/hour

	Quantity Name	Type	Scope	Value	Initial Value	Units
523	k_Liver_IC_S1_Liver_EC_S1_1	parameter	PBPK_MADAM	0	0.26572	liter/hour
524	k_Liver_EC_S2_Liver_IC_S2_1	parameter	PBPK_MADAM	1	168.0698	liter/hour
525	k_Liver_IC_S2_Liver_EC_S2_1	parameter	PBPK_MADAM	0	0.26572	liter/hour
526	k_Liver_IC_S4_Liver_EC_S4_1	parameter	PBPK_MADAM	0	0.26572	liter/hour
527	k_Liver_EC_S4_Liver_IC_S4_1	parameter	PBPK_MADAM	1	168.0698	liter/hour
528	k_Liver_IC_S5_Liver_EC_S5_1	parameter	PBPK_MADAM	0	0.26572	liter/hour
529	k_Liver_EC_S5_Liver_IC_S5_1	parameter	PBPK_MADAM	1	168.0698	liter/hour
530	k_Liver_EC_S3_Liver_IC_S3_1	parameter	PBPK_MADAM	1	168.0698	liter/hour
531	Q_li_1	parameter	PBPK_MADAM	1	84	liter/hour
532	k_Liver_EC_S1_Liver_EC_S2_1	parameter	PBPK_MADAM	1	84	liter/hour
533	k_Liver_EC_S2_Liver_EC_S3_1	parameter	PBPK_MADAM	1	84	liter/hour
534	k_Liver_EC_S3_Liver_EC_S4_1	parameter	PBPK_MADAM	1	84	liter/hour
535	k_Liver_EC_S4_Liver_EC_S5_1	parameter	PBPK_MADAM	1	84	liter/hour
536	k_Liver_IC_S5_Bile_1	parameter	PBPK_MADAM	1	0.019658	liter/hour
537	k_Liver_IC_S4_Bile_1	parameter	PBPK_MADAM	1	0.019658	liter/hour
538	k_Liver_IC_S3_Bile_1	parameter	PBPK_MADAM	1	0.019658	liter/hour
539	k_Liver_IC_S2_Bile_1	parameter	PBPK_MADAM	1	0.019658	liter/hour
540	k_Liver_IC_S1_Bile_1	parameter	PBPK_MADAM	1	0.019658	liter/hour

	Quantity Name	Type	Scope	Value	Initial Value	Units
541	k_Liver_IC_S1_Metabolites_1	parameter	PBPK_MADAM	1	0.21013	liter/hour
542	k_Liver_IC_S2_Metabolites_1	parameter	PBPK_MADAM	1	0.21013	liter/hour
543	k_Liver_IC_S3_Metabolites_1	parameter	PBPK_MADAM	1	0.21013	liter/hour
544	k_Liver_IC_S4_Metabolites_1	parameter	PBPK_MADAM	1	0.21013	liter/hour
545	k_Liver_IC_S5_Metabolites_1	parameter	PBPK_MADAM	1	0.21013	liter/hour
546	drug_fB_1	parameter	PBPK_MADAM	1	0.058769	dimensionless
547	drug_fuLiver_1	parameter	PBPK_MADAM	0.018	0.018	dimensionless
548	Kp_kidney_1	parameter	PBPK_MADAM	0.134	0.13478	dimensionless
549	drug_BRP_1	parameter	PBPK_MADAM	0.65	0.65	dimensionless
550	Kp_heart_1	parameter	PBPK_MADAM	0.16	0.10367	dimensionless
551	Kp_gut_1	parameter	PBPK_MADAM	0.165	0.24363	dimensionless
552	Kp_brain_1	parameter	PBPK_MADAM	0.057	0.16588	dimensionless
553	Kp_bone_1	parameter	PBPK_MADAM	0.108	0.31102	dimensionless
554	Kp_adipose_1	parameter	PBPK_MADAM	0.047	0.23327	dimensionless
555	Kp_muscle_1	parameter	PBPK_MADAM	0.038	0.025919	dimensionless
556	Kp_rest_1	parameter	PBPK_MADAM	0.12	0.0065	dimensionless
557	Kp_lung_1	parameter	PBPK_MADAM	0.21	0.20735	dimensionless
558	drug_CLrenal_1	parameter	PBPK_MADAM	0	0	liter/hour
559	drug_fuplasma_1	parameter	PBPK_MADAM	0.0382	0.0382	dimensionless
560	Kp_spleen_1	parameter	PBPK_MADAM	0.1	0.18143	dimensionless

	Quantity Name	Type	Scope	Value	Initial Value	Units
561	k_Liver_EC_S5_Venous_1	parameter	PBPK_MADAM	1	84	liter/hour
562	k_artery_heart_1	parameter	PBPK_MADAM	1	12.6	liter/hour
563	k_Liver_IC_S3_Liver__EC_S3_1	parameter	PBPK_MADAM	0	0.26572	liter/hour
564	k_artery_liver_1	parameter	PBPK_MADAM	1	4.2	liter/hour
565	drug_PSinfg_1	parameter	PBPK_MADAM	471	471	microliter/minute
566	phys_HPGL_1	parameter	PBPK_MADAM	122	122	1/gram
567	switch_SFinf_1	parameter	PBPK_MADAM	3.196	3.196	dimensionless
568	drug_PSbileg_1	parameter	PBPK_MADAM	2.5	2.5	microliter/minute
569	switch_SFbile_1	parameter	PBPK_MADAM	0.23114	0.23114	dimensionless
570	drug_PSdifg_1	parameter	PBPK_MADAM	5	5	microliter/minute
571	switch_SFdiff_1	parameter	PBPK_MADAM	1.5621	1.5621	dimensionless
572	drug_CLmetg_1	parameter	PBPK_MADAM	19	19	microliter/minute
573	drug_HLM_CLint_1	parameter	PBPK_MADAM	0	0	milliliter/minute/milligram
574	drug_fumic_1	parameter	PBPK_MADAM	1	1	dimensionless
575	drug_funic_1	parameter	PBPK_MADAM	1	1	dimensionless
576	Kp_skin_1	parameter	PBPK_MADAM	0.28	0.1918	dimensionless
577	switch_SFrenal_1	parameter	PBPK_MADAM	1	1	dimensionless
578	k_venous_urine_CLR_1	parameter	PBPK_MADAM	1	0	liter/hour
579	Qtestes_1	parameter	PBPK_MADAM	1	0	liter/hour
580	k_artery_testes_1	parameter	PBPK_MADAM	1	0	liter/hour
581	Kp_testes_1	parameter	PBPK_MADAM	1	0.51837	dimensionless
582	k_testes_venous_1	parameter	PBPK_MADAM	1	0	liter/hour

	Quantity Name	Type	Scope	Value	Initial Value	Units
583	k_Liver_IC_S2_Liver_EC_S2_efflux_1	parameter	PBPK_MADAM	1	0	liter/hour
584	k_Liver_IC_S3_Liver_EC_S3_efflux_1	parameter	PBPK_MADAM	1	0	liter/hour
585	k_Liver_IC_S4_Liver_EC_S4_efflux_1	parameter	PBPK_MADAM	1	0	liter/hour
586	k_Liver_IC_S5_Liver_EC_S5_efflux_1	parameter	PBPK_MADAM	1	0	liter/hour
587	k_Liver_IC_S1_Liver_EC_S1_efflux_1	parameter	PBPK_MADAM	1	0	liter/hour
588	drug_fa_1	parameter	PBPK_MADAM	1	1	dimensionless
589	drug_dose_rate_IV_1	parameter	PBPK_MADAM	1	1	milligram/hour
590	switch_SFmet_1	parameter	PBPK_MADAM	0.32509	0.32509	dimensionless
591	drug_Km_uptake_1	parameter	PBPK_MADAM	1	1	micromole/liter
592	switch_Vmax_uptake_1	parameter	PBPK_MADAM	0	0	micromole/kilogram/hour
593	drug_molar_mass_1	parameter	PBPK_MADAM	712800	712800	milligram/mole
594	drug_Km_met_1	parameter	PBPK_MADAM	1	1	micromole/liter
595	switch_Vmax_met_1	parameter	PBPK_MADAM	0	0	micromole/hour/kilogram
596	drug_Kp_adipose_raw_1	parameter	PBPK_MADAM	0.45	0.45	dimensionless
597	switch_SFKp_1	parameter	PBPK_MADAM	0.51837	0.51837	dimensionless
598	drug_Kp_bone_raw_1	parameter	PBPK_MADAM	0.6	0.6	dimensionless
599	drug_Kp_brain_raw_1	parameter	PBPK_MADAM	0.32	0.32	dimensionless
600	drug_Kp_gut_raw_1	parameter	PBPK_MADAM	0.47	0.47	dimensionless

	Quantity Name	Type	Scope	Value	Initial Value	Units
601	drug_Kp_heart_raw_1	parameter	PBPK_MADAM	0.2	0.2	dimensionless
602	drug_Kp_kidney_raw_1	parameter	PBPK_MADAM	0.26	0.26	dimensionless
603	drug_Kp_lung_raw_1	parameter	PBPK_MADAM	0.4	0.4	dimensionless
604	drug_Kp_muscle_raw_1	parameter	PBPK_MADAM	0.05	0.05	dimensionless
605	drug_Kp_rest_raw_1	parameter	PBPK_MADAM	0.0065	0.0065	dimensionless
606	drug_Kp_skin_raw_1	parameter	PBPK_MADAM	0.37	0.37	dimensionless
607	drug_Kp_spleen_raw_1	parameter	PBPK_MADAM	0.35	0.35	dimensionless
608	drug_Kp_testes_raw_1	parameter	PBPK_MADAM	1	1	dimensionless
609	k_transit_1	parameter	PBPK_MADAM	0	0	1/(hour)
610	drug_k_oral_1	parameter	PBPK_MADAM	10	10	1/(hour)
611	switch_SFeff_1	parameter	PBPK_MADAM	0	0	dimensionless
612	k_venous_urine_GFR_1	parameter	PBPK_MADAM	1	0	liter/hour
613	drug_GFR_1	parameter	PBPK_MADAM	1	0	milliliter/minute
614	drug_CLefflluxHep_1	parameter	PBPK_MADAM	0	0	microliter/minute
615	drug_FR_1	parameter	PBPK_MADAM	0	0	dimensionless
616	switch_slow_dist_Kp_1	parameter	PBPK_MADAM	1	1	dimensionless
617	drug_uptake_Ki	parameter	PBPK_MADAM	0.226	0.226	micromole/liter
618	drug_dose_amount_IV	parameter	PBPK_MADAM	0	0	milligram
619	drug_dose_amount_PO_1	parameter	PBPK_MADAM	0	0	milligram
620	drug_dose_amount_IV_1	parameter	PBPK_MADAM	0	0	milligram
621	drug_dose_amount_PO	parameter	PBPK_MADAM	0	0	milligram

	Quantity Name	Type	Scope	Value	Initial Value	Units
622	uptake_inhib_S1	parameter	PBPK_MADAM	1	1	dimensionless
623	uptake_inhib_S2	parameter	PBPK_MADAM	1	1	dimensionless
624	uptake_inhib_S3	parameter	PBPK_MADAM	1	1	dimensionless
625	uptake_inhib_S4	parameter	PBPK_MADAM	1	1	dimensionless
626	uptake_inhib_S5	parameter	PBPK_MADAM	1	1	dimensionless
627	switch_biliary_inhib_1	parameter	PBPK_MADAM	0	0	dimensionless
628	switch_met_inhib_1	parameter	PBPK_MADAM	0	0	dimensionless
629	biliary_inhib_S1	parameter	PBPK_MADAM	1	1	dimensionless
630	biliary_inhib_S2	parameter	PBPK_MADAM	1	1	dimensionless
631	biliary_inhib_S3	parameter	PBPK_MADAM	1	1	dimensionless
632	biliary_inhib_S4	parameter	PBPK_MADAM	1	1	dimensionless
633	biliary_inhib_S5	parameter	PBPK_MADAM	1	1	dimensionless
634	switch_uptake_inhib_1	parameter	PBPK_MADAM	0	0	dimensionless
635	met_inhib_S1	parameter	PBPK_MADAM	1	1	dimensionless
636	met_inhib_S2	parameter	PBPK_MADAM	1	1	dimensionless
637	met_inhib_S3	parameter	PBPK_MADAM	1	1	dimensionless
638	met_inhib_S4	parameter	PBPK_MADAM	1	1	dimensionless
639	met_inhib_S5	parameter	PBPK_MADAM	1	1	dimensionless
640	drug_biliary_Ki	parameter	PBPK_MADAM	0.226	0.226	micromole/liter
641	drug_met_Ki	parameter	PBPK_MADAM	0.226	0.226	micromole/liter
642	drug_dose_IV_start_1	parameter	PBPK_MADAM	3	3	hour
643	drug_dose_IV_start	parameter	PBPK_MADAM	3	3	hour

	Quantity Name	Type	Scope	Value	Initial Value	Units
644	Kpuu_Liver_1	parameter	PBPK_MADAM	1	NaN	dimensionless
645	drug_k_bile_deg	parameter	PBPK_MADAM	0	0	1/hour
646	drug_k_bile_deg_1	parameter	PBPK_MADAM	0	0	1/hour
647	TSTOMACH	parameter	PBPK_MADAM	15	16.2	minute
648	TDUO	parameter	PBPK_MADAM	15.6	9.384	minute
649	TJEJ1	parameter	PBPK_MADAM	56.4	35.292	minute
650	TJEJ2	parameter	PBPK_MADAM	42	35.292	minute
651	TILL1	parameter	PBPK_MADAM	34.8	31.008	minute
652	TILL2	parameter	PBPK_MADAM	25.2	31.008	minute
653	TILL3	parameter	PBPK_MADAM	17.4	31.008	minute
654	TILL4	parameter	PBPK_MADAM	261	31.008	minute
655	QMUC	parameter	PBPK_MADAM	142.8571	170	milliliter/minute
656	SOLIF_STOMACH	parameter	PBPK_MADAM	1476.373	7.9433	milligram/liter
657	SOLIF_DUO	parameter	PBPK_MADAM	1476.373	0.00010026	milligram/liter
658	SOLIF_JEJ1	parameter	PBPK_MADAM	1476.373	7.969e-05	milligram/liter
659	SOLIF_JEJ2	parameter	PBPK_MADAM	1476.373	6.3353e-05	milligram/liter
660	SOLIF_ILL1	parameter	PBPK_MADAM	1476.373	4.0068e-05	milligram/liter
661	SOLIF_ILL2	parameter	PBPK_MADAM	1476.373	2.5376e-05	milligram/liter
662	SOLIF_ILL3	parameter	PBPK_MADAM	1476.373	5.2689e-06	milligram/liter
663	SOLIF_ILL4	parameter	PBPK_MADAM	1476.373	1.2846e-05	milligram/liter
664	DIFF	parameter	PBPK_MADAM	0.010286	204	centimeter^3/minute
665	NI_DUO	parameter	PBPK_MADAM	1	0.0025638	dimensionless

	Quantity Name	Type	Scope	Value	Initial Value	Units
666	NI_JEJ1	parameter	PBPK_MADAM	1	0.0032255	dimensionless
667	NI_JEJ2	parameter	PBPK_MADAM	1	0.0040573	dimensionless
668	NI_ILL1	parameter	PBPK_MADAM	1	0.0064151	dimensionless
669	NI_ILL2	parameter	PBPK_MADAM	1	0.010129	dimensionless
670	NI_ILL3	parameter	PBPK_MADAM	1	0.048784	dimensionless
671	NI_ILL4	parameter	PBPK_MADAM	1	0.020009	dimensionless
672	KD	parameter	PBPK_MADAM	0.0002	1.5385e-05	liter/milligram/minute
673	fu_mem	parameter	PBPK_MADAM	1	0.016351	dimensionless
674	CLINT_efflux_DUO	parameter	PBPK_MADAM	1	0	milliliter/minute
675	CLINT_influx_DUO	parameter	PBPK_MADAM	1	0	milliliter/minute
676	CLINT_metabolic_DUO	parameter	PBPK_MADAM	1	1	milliliter/minute
677	CLINT_efflux_JEJ1	parameter	PBPK_MADAM	1	0	milliliter/minute
678	CLINT_influx_JEJ1	parameter	PBPK_MADAM	1	0	milliliter/minute
679	CLINT_metabolic_JEJ1	parameter	PBPK_MADAM	1	1	milliliter/minute
680	CLINT_efflux_JEJ2	parameter	PBPK_MADAM	1	0	milliliter/minute
681	CLINT_influx_JEJ2	parameter	PBPK_MADAM	1	0	milliliter/minute
682	CLINT_metabolic_JEJ2	parameter	PBPK_MADAM	1	1	milliliter/minute
683	CLINT_efflux_ILL1	parameter	PBPK_MADAM	1	0	milliliter/minute
684	CLINT_influx_ILL1	parameter	PBPK_MADAM	1	0	milliliter/minute
685	CLINT_metabolic_ILL1	parameter	PBPK_MADAM	1	1	milliliter/minute
686	CLINT_efflux_ILL2	parameter	PBPK_MADAM	1	0	milliliter/minute
687	CLINT_influx_ILL2	parameter	PBPK_MADAM	1	0	milliliter/minute

	Quantity Name	Type	Scope	Value	Initial Value	Units
688	CLINT_metabolic_ILL2	parameter	PBPK_MADAM	1	1	milliliter/minute
689	CLINT_efflux_ILL3	parameter	PBPK_MADAM	1	0	milliliter/minute
690	CLINT_influx_ILL3	parameter	PBPK_MADAM	1	0	milliliter/minute
691	CLINT_metabolic_ILL3	parameter	PBPK_MADAM	1	1	milliliter/minute
692	CLINT_efflux_ILL4	parameter	PBPK_MADAM	1	0	milliliter/minute
693	CLINT_influx_ILL4	parameter	PBPK_MADAM	1	0	milliliter/minute
694	CLINT_metabolic_ILL4	parameter	PBPK_MADAM	1	1	milliliter/minute
695	BW_average	parameter	PBPK_MADAM	70	70	kilogram
696	liter_to_milliliter	parameter	PBPK_MADAM	1000	1000	milliliter/liter
697	phys_Normalized_weight_stomach	parameter	PBPK_MADAM	2.1	2.1	gram/kilogram
698	V_LUM_TOT	parameter	PBPK_MADAM	6543.215	126.95	milliliter
699	V_ONECOMP	parameter	PBPK_MADAM	934.745	18.1357	milliliter
700	VGut	parameter	PBPK_MADAM	50	0.517	liter
701	V_MEM	parameter	PBPK_MADAM	7142.8571	73.857	milliliter
702	HHINT	parameter	PBPK_MADAM	1	3890452.4499	dimensionless
703	HHSTOMACH	parameter	PBPK_MADAM	1	30902955.3251	dimensionless
704	HHDUO	parameter	PBPK_MADAM	1	390.0451	dimensionless
705	HHJEJ1	parameter	PBPK_MADAM	1	310.0295	dimensionless
706	HHJEJ2	parameter	PBPK_MADAM	1	246.4709	dimensionless
707	HHILL1	parameter	PBPK_MADAM	1	155.8817	dimensionless
708	HHILL2	parameter	PBPK_MADAM	1	98.7237	dimensionless
709	HHILL3	parameter	PBPK_MADAM	1	20.4984	dimensionless

	Quantity Name	Type	Scope	Value	Initial Value	Units
710	HHILL4	parameter	PBPK_MADAM	1	49.9779	dimensionless
711	LOGP	parameter	PBPK_MADAM	1	3.16	dimensionless
712	MW	parameter	PBPK_MADAM	1	747970	microgram/micromole
713	CACO2AB	parameter	PBPK_MADAM	1e-06	1e-06	centimeter/second
714	CACO2BA	parameter	PBPK_MADAM	1e-06	1e-06	centimeter/second
715	HPeff_exp	parameter	PBPK_MADAM	0.0001	0.0001	centimeter/second
716	SOLWATER	parameter	PBPK_MADAM	1	1	milligram/liter
717	REFPHSOL	parameter	PBPK_MADAM	7.4	2.4	dimensionless
718	PSIZE	parameter	PBPK_MADAM	0.0005	0.0065	centimeter
719	PDENSITY	parameter	PBPK_MADAM	1000000	1000000	microgram/milliliter
720	DLT	parameter	PBPK_MADAM	0.003	0.003	centimeter
721	DIFFCOEFF	parameter	PBPK_MADAM	0.0001	0.0001	centimeter^2/minute
722	LL	parameter	PBPK_MADAM	680	680	centimeter
723	LR	parameter	PBPK_MADAM	1.75	1.75	centimeter
724	phys_ESA	parameter	PBPK_MADAM	120000	120000	centimeter^2
725	LOGSR	parameter	PBPK_MADAM	3.02	4.64	dimensionless
726	SOLBILE	parameter	PBPK_MADAM	368.8433	368.8433	milligram/liter
727	NATC	parameter	PBPK_MADAM	4	4	nanomole/liter
728	SOLFASSIF	parameter	PBPK_MADAM	1	1	milligram/liter
729	SOLINT	parameter	PBPK_MADAM	1476.373	2.5704e-07	milligram/liter
730	HPeff_est	parameter	PBPK_MADAM	0.0001	0.00019833	centimeter/second
731	numIntestinalCompartments	parameter	PBPK_MADAM	7	7	dimensionless

	Quantity Name	Type	Scope	Value	Initial Value	Units
732	second_per_minute	parameter	PBPK_MADAM	60	60	second/minute
733	minute_per_hour	parameter	PBPK_MADAM	60	60	minute/hour
734	milligram_per_microgram	parameter	PBPK_MADAM	0.001	0.001	milligram/microgram
735	pHStomach	parameter	PBPK_MADAM	1	1.5	dimensionless
736	pHDuo	parameter	PBPK_MADAM	1	6.4	dimensionless
737	pHJej1	parameter	PBPK_MADAM	1	6.5	dimensionless
738	pHJej2	parameter	PBPK_MADAM	1	6.6	dimensionless
739	pHIll1	parameter	PBPK_MADAM	1	6.8	dimensionless
740	pHIll2	parameter	PBPK_MADAM	1	7	dimensionless
741	pHIll3	parameter	PBPK_MADAM	1	7.7	dimensionless
742	pHIll4	parameter	PBPK_MADAM	1	7.3	dimensionless
743	fu_blood	parameter	PBPK_MADAM	1	0.18	dimensionless
744	drug_Km_influx	parameter	PBPK_MADAM	1	1	micromole/liter
745	drug_Km_efflux	parameter	PBPK_MADAM	1	1	micromole/liter
746	Gut_EC_fraction	parameter	PBPK_MADAM	0.3719	0.3719	dimensionless
747	Gut_IC_fraction	parameter	PBPK_MADAM	0.6281	0.6281	dimensionless
748	influx_factor_duo	parameter	PBPK_MADAM	1	1	dimensionless
749	influx_factor_jej1	parameter	PBPK_MADAM	1	1	dimensionless
750	influx_factor_jej2	parameter	PBPK_MADAM	1	1	dimensionless
751	influx_factor_ill1	parameter	PBPK_MADAM	1	1	dimensionless
752	influx_factor_ill2	parameter	PBPK_MADAM	1	1	dimensionless
753	influx_factor_ill3	parameter	PBPK_MADAM	1	1	dimensionless

	Quantity Name	Type	Scope	Value	Initial Value	Units
754	influx_factor_ill4	parameter	PBPK_MADAM	1	1	dimensionless
755	efflux_factor_duo	parameter	PBPK_MADAM	1	0.51	dimensionless
756	efflux_factor_jej1	parameter	PBPK_MADAM	1	1	dimensionless
757	efflux_factor_jej2	parameter	PBPK_MADAM	1	1.46	dimensionless
758	efflux_factor_ill1	parameter	PBPK_MADAM	1	1.5	dimensionless
759	efflux_factor_ill2	parameter	PBPK_MADAM	1	1.51	dimensionless
760	efflux_factor_ill3	parameter	PBPK_MADAM	1	1.52	dimensionless
761	efflux_factor_ill4	parameter	PBPK_MADAM	1	1.51	dimensionless
762	switchVmax_influx	parameter	PBPK_MADAM	0	0	micromole/minute/centimeter^2
763	switchVmax_efflux	parameter	PBPK_MADAM	0	0	micromole/minute/centimeter^2
764	Qmuc_DUO	parameter	PBPK_MADAM	1	26.9235	milliliter/minute
765	Qmuc_JEJ1	parameter	PBPK_MADAM	1	74.0397	milliliter/minute
766	Qmuc_JEJ2	parameter	PBPK_MADAM	1	74.0397	milliliter/minute
767	Qmuc_ILL1	parameter	PBPK_MADAM	1	32.7365	milliliter/minute
768	Qmuc_ILL2	parameter	PBPK_MADAM	1	32.7365	milliliter/minute
769	Qmuc_ILL3	parameter	PBPK_MADAM	1	32.7365	milliliter/minute
770	Qmuc_ILL4	parameter	PBPK_MADAM	1	32.7365	milliliter/minute
771	volumeRatio_DUO	parameter	PBPK_MADAM	0.1429	0.11534	dimensionless
772	volumeRatio_JEJ1	parameter	PBPK_MADAM	0.1429	0.22722	dimensionless
773	volumeRatio_JEJ2	parameter	PBPK_MADAM	0.1429	0.15917	dimensionless
774	volumeRatio_ILL1	parameter	PBPK_MADAM	0.1429	0.12687	dimensionless
775	volumeRatio_ILL2	parameter	PBPK_MADAM	0.1429	0.12687	dimensionless

	Quantity Name	Type	Scope	Value	Initial Value	Units
776	volumeRatio_ILL3	parameter	PBPK_MADAM	0.1429	0.12457	dimensionless
777	volumeRatio_ILL4	parameter	PBPK_MADAM	0.1429	0.11995	dimensionless
778	flowRatio_DUO	parameter	PBPK_MADAM	0.1429	0.088	dimensionless
779	flowRatio_JEJ1	parameter	PBPK_MADAM	0.1429	0.242	dimensionless
780	flowRatio_JEJ2	parameter	PBPK_MADAM	0.1429	0.242	dimensionless
781	flowRatio_ILL1	parameter	PBPK_MADAM	0.1429	0.107	dimensionless
782	flowRatio_ILL2	parameter	PBPK_MADAM	0.1429	0.107	dimensionless
783	flowRatio_ILL3	parameter	PBPK_MADAM	0.1429	0.107	dimensionless
784	flowRatio_ILL4	parameter	PBPK_MADAM	0.1429	0.107	dimensionless
785	switch_SFinflux	parameter	PBPK_MADAM	1	1	dimensionless
786	switch_SFefflux	parameter	PBPK_MADAM	1	1	dimensionless
787	switch_SFgutmet	parameter	PBPK_MADAM	1	1	dimensionless
788	switch_SFdiffapi	parameter	PBPK_MADAM	1	1	dimensionless
789	switch_SFdiffbaso	parameter	PBPK_MADAM	1	1	dimensionless
790	zero	parameter	PBPK_MADAM	0	0	micromole/minute/centimeter^2
791	CLINT_metabolism	parameter	PBPK_MADAM	1	0	milliliter/minute
792	metabolism_factor_duo	parameter	PBPK_MADAM	1	1	dimensionless
793	metabolism_factor_jej1	parameter	PBPK_MADAM	1	1	dimensionless
794	metabolism_factor_jej2	parameter	PBPK_MADAM	1	1	dimensionless
795	metabolism_factor_ill1	parameter	PBPK_MADAM	1	1	dimensionless
796	metabolism_factor_ill2	parameter	PBPK_MADAM	1	1	dimensionless
797	metabolism_factor_ill3	parameter	PBPK_MADAM	1	1	dimensionless

	Quantity Name	Type	Scope	Value	Initial Value	Units
798	metabolism_factor_ill4	parameter	PBPK_MADAM	1	1	dimensionless
799	k_liver_metabolites	parameter	PBPK_MADAM	1	3.9865	liter/hour
800	k_liver_bile	parameter	PBPK_MADAM	1	0	liter/hour
801	Kp_liver	parameter	PBPK_MADAM	1	5.9321	dimensionless
802	drug_Kp_liver_raw	parameter	PBPK_MADAM	1.1	5.9321	dimensionless
803	phys_Normalized_Q_villi	parameter	PBPK_MADAM	4.8	4.8	milliliter/minute/kilogram
804	phys_Normalized_weight_enterocyte	parameter	PBPK_MADAM	7.3857	7.3857	gram/kilogram
805	LumenTotal	parameter	PBPK_MADAM	126.95	126.95	milliliter
806	lumenvolumeRatio_DUO	parameter	PBPK_MADAM	1	0.27058	dimensionless
807	lumenvolumeRatio_JEJ1	parameter	PBPK_MADAM	1	0.16621	dimensionless
808	lumenvolumeRatio_JEJ2	parameter	PBPK_MADAM	1	0.16621	dimensionless
809	lumenvolumeRatio_ILL1	parameter	PBPK_MADAM	1	0.099252	dimensionless
810	lumenvolumeRatio_ILL2	parameter	PBPK_MADAM	1	0.099252	dimensionless
811	lumenvolumeRatio_ILL3	parameter	PBPK_MADAM	1	0.099252	dimensionless
812	lumenvolumeRatio_ILL4	parameter	PBPK_MADAM	1	0.099252	dimensionless
813	Q_villi	parameter	PBPK_MADAM	1	336	milliliter/minute
814	StomachLumenTotal	parameter	PBPK_MADAM	50	50	milliliter
815	phys_ESA_baso	parameter	PBPK_MADAM	1	6703	centimeter^2
816	DIFF_duo	parameter	PBPK_MADAM	1	164.22	centimeter^3/minute
817	DIFF_jej1	parameter	PBPK_MADAM	1	324.156	centimeter^3/minute

	Quantity Name	Type	Scope	Value	Initial Value	Units
818	DIFF_jej2	parameter	PBPK_MADAM	1	227.052	centimeter^3/minute
819	DIFF_ill1	parameter	PBPK_MADAM	1	181.356	centimeter^3/minute
820	DIFF_ill2	parameter	PBPK_MADAM	1	181.356	centimeter^3/minute
821	DIFF_ill3	parameter	PBPK_MADAM	1	178.5	centimeter^3/minute
822	DIFF_ill4	parameter	PBPK_MADAM	1	171.36	centimeter^3/minute
823	surfaceRatio_DUO	parameter	PBPK_MADAM	1	0.115	dimensionless
824	surfaceRatio_JEJ1	parameter	PBPK_MADAM	1	0.227	dimensionless
825	surfaceRatio_JEJ2	parameter	PBPK_MADAM	1	0.159	dimensionless
826	surfaceRatio_ILL1	parameter	PBPK_MADAM	1	0.127	dimensionless
827	surfaceRatio_ILL2	parameter	PBPK_MADAM	1	0.127	dimensionless
828	surfaceRatio_ILL3	parameter	PBPK_MADAM	1	0.125	dimensionless
829	surfaceRatio_ILL4	parameter	PBPK_MADAM	1	0.12	dimensionless
830	DIFF_BASO_duo	parameter	PBPK_MADAM	1	4.1957	milliliter/minute
831	DIFF_BASO_jej1	parameter	PBPK_MADAM	1	16.1605	milliliter/minute
832	DIFF_BASO_jej2	parameter	PBPK_MADAM	1	16.1605	milliliter/minute
833	DIFF_BASO_ill1	parameter	PBPK_MADAM	1	10.8162	milliliter/minute
834	DIFF_BASO_ill2	parameter	PBPK_MADAM	1	10.8162	milliliter/minute
835	DIFF_BASO_ill3	parameter	PBPK_MADAM	1	10.8162	milliliter/minute
836	DIFF_BASO_ill4	parameter	PBPK_MADAM	1	10.8162	milliliter/minute
837	basoSurfaceRatio_DUO	parameter	PBPK_MADAM	1	0.0526	dimensionless
838	basoSurfaceRatio_JEJ1	parameter	PBPK_MADAM	1	0.2026	dimensionless
839	basoSurfaceRatio_JEJ	parameter	PBPK_MADAM	1	0.2026	dimensionless

	Quantity Name	Type	Scope	Value	Initial Value	Units
	2					
840	basoSurfaceRatio_ILL1	parameter	PBPK_MADAM	1	0.1356	dimensionless
841	basoSurfaceRatio_ILL2	parameter	PBPK_MADAM	1	0.1356	dimensionless
842	basoSurfaceRatio_ILL3	parameter	PBPK_MADAM	1	0.1356	dimensionless
843	basoSurfaceRatio_ILL4	parameter	PBPK_MADAM	1	0.1356	dimensionless
844	drug_fQ	parameter	PBPK_MADAM	1	0.2571	dimensionless
845	k_artery_serosa	parameter	PBPK_MADAM	1	53.0431	liter/hour
846	k_serosa_liver	parameter	PBPK_MADAM	1	15.5346	liter/hour
847	Kp_serosa	parameter	PBPK_MADAM	1	3.4145	dimensionless
848	drug_Kp_serosa_raw	parameter	PBPK_MADAM	1	3.4145	dimensionless
849	pKA	parameter	PBPK_MADAM	9.3	8.99	dimensionless
850	CL_inf_api	parameter	PBPK_MADAM	1	0	microliter/minute/centimeter^2
851	CL_eff	parameter	PBPK_MADAM	1	0	microliter/minute/centimeter^2
852	CLINT_influx_baso_DUO	parameter	PBPK_MADAM	1	0	milliliter/minute
853	CLINT_influx_baso_JEJ1	parameter	PBPK_MADAM	1	0	milliliter/minute
854	CLINT_influx_baso_JEJ2	parameter	PBPK_MADAM	1	0	milliliter/minute
855	CLINT_influx_baso_ILL1	parameter	PBPK_MADAM	1	0	milliliter/minute
856	CLINT_influx_baso_ILL2	parameter	PBPK_MADAM	1	0	milliliter/minute
857	CLINT_influx_baso_ILL3	parameter	PBPK_MADAM	1	0	milliliter/minute
858	CLINT_influx_baso_ILL4	parameter	PBPK_MADAM	1	0	milliliter/minute

	Quantity Name	Type	Scope	Value	Initial Value	Units
859	diff_api	parameter	PBPK_MADAM	1	11.9	microliter/minute/centimeter^2
860	HPeff_est_baso	parameter	PBPK_MADAM	1	0.00019833	centimeter/second
861	diff_baso	parameter	PBPK_MADAM	1	11.9	microliter/minute/centimeter^2
862	CL_inf_baso	parameter	PBPK_MADAM	1	0	microliter/minute/centimeter^2
863	influx_factor_duo_baso	parameter	PBPK_MADAM	1	1	dimensionless
864	influx_factor_jej1_baso	parameter	PBPK_MADAM	1	1	dimensionless
865	influx_factor_jej2_baso	parameter	PBPK_MADAM	1	1	dimensionless
866	influx_factor_ill1_baso	parameter	PBPK_MADAM	1	1	dimensionless
867	influx_factor_ill2_baso	parameter	PBPK_MADAM	1	1	dimensionless
868	influx_factor_ill3_baso	parameter	PBPK_MADAM	1	1	dimensionless
869	influx_factor_ill4_baso	parameter	PBPK_MADAM	1	1	dimensionless
870	k_Liver_EC_S5_Venous	parameter	PBPK_MADAM	1	84	liter/hour
871	switch_liverFlag	parameter	PBPK_MADAM	1	1	dimensionless
872	phys_Normalized_ESA	parameter	PBPK_MADAM	1	1714.2857	centimeter^2/kilogram
873	phys_Normalized_ESA_baso	parameter	PBPK_MADAM	1	95.7571	centimeter^2/kilogram
874	drug_inputFlag	parameter	PBPK_MADAM	1	1	dimensionless
875	drug_pKABase1	parameter	PBPK_MADAM	1	8.99	dimensionless
876	drug_pKABase2	parameter	PBPK_MADAM	1	1	dimensionless
877	drug_pKAAcid1	parameter	PBPK_MADAM	1	1	dimensionless
878	drug_pKAAcid2	parameter	PBPK_MADAM	1	1	dimensionless
879	SOLIF_STOMACH_1	parameter	PBPK_MADAM	1476.373	1.3162	milligram/liter
880	SOLIF_DUO_1	parameter	PBPK_MADAM	1476.373	1	milligram/liter

	Quantity Name	Type	Scope	Value	Initial Value	Units
881	SOLIF_JEJ1_1	parameter	PBPK_MADAM	1476.373	1	milligram/liter
882	SOLIF_JEJ2_1	parameter	PBPK_MADAM	1476.373	1	milligram/liter
883	SOLIF_ILL1_1	parameter	PBPK_MADAM	1476.373	1	milligram/liter
884	SOLIF_ILL2_1	parameter	PBPK_MADAM	1476.373	1	milligram/liter
885	SOLIF_ILL3_1	parameter	PBPK_MADAM	1476.373	1	milligram/liter
886	SOLIF_ILL4_1	parameter	PBPK_MADAM	1476.373	1	milligram/liter
887	NI_DUO_1	parameter	PBPK_MADAM	1	1	dimensionless
888	NI_JEJ1_1	parameter	PBPK_MADAM	1	1	dimensionless
889	NI_JEJ2_1	parameter	PBPK_MADAM	1	1	dimensionless
890	NI_ILL1_1	parameter	PBPK_MADAM	1	1	dimensionless
891	NI_ILL2_1	parameter	PBPK_MADAM	1	1	dimensionless
892	NI_ILL3_1	parameter	PBPK_MADAM	1	1	dimensionless
893	NI_ILL4_1	parameter	PBPK_MADAM	1	1	dimensionless
894	KD_1	parameter	PBPK_MADAM	0.0002	0.0002	liter/milligram/minute
895	fu_mem_1	parameter	PBPK_MADAM	1	1	dimensionless
896	CLINT_efflux_DUO_1	parameter	PBPK_MADAM	1	13.8	milliliter/minute
897	CLINT_influx_DUO_1	parameter	PBPK_MADAM	1	13.8	milliliter/minute
898	CLINT_efflux_JEJ1_1	parameter	PBPK_MADAM	1	27.24	milliliter/minute
899	CLINT_influx_JEJ1_1	parameter	PBPK_MADAM	1	27.24	milliliter/minute
900	CLINT_efflux_JEJ2_1	parameter	PBPK_MADAM	1	19.08	milliliter/minute
901	CLINT_influx_JEJ2_1	parameter	PBPK_MADAM	1	19.08	milliliter/minute
902	CLINT_efflux_ILL1_1	parameter	PBPK_MADAM	1	15.24	milliliter/minute

	Quantity Name	Type	Scope	Value	Initial Value	Units
903	CLINT_influx_ILL1_1	parameter	PBPK_MADAM	1	15.24	milliliter/minute
904	CLINT_efflux_ILL2_1	parameter	PBPK_MADAM	1	15.24	milliliter/minute
905	CLINT_influx_ILL2_1	parameter	PBPK_MADAM	1	15.24	milliliter/minute
906	CLINT_efflux_ILL3_1	parameter	PBPK_MADAM	1	15	milliliter/minute
907	CLINT_influx_ILL3_1	parameter	PBPK_MADAM	1	15	milliliter/minute
908	CLINT_efflux_ILL4_1	parameter	PBPK_MADAM	1	14.4	milliliter/minute
909	CLINT_influx_ILL4_1	parameter	PBPK_MADAM	1	14.4	milliliter/minute
910	fu_blood_1	parameter	PBPK_MADAM	1	1	dimensionless
911	drug_Km_influx_1	parameter	PBPK_MADAM	1	1	micromole/liter
912	drug_Km_efflux_1	parameter	PBPK_MADAM	1	1	micromole/liter
913	switchVmax_influx_1	parameter	PBPK_MADAM	0	0	micromole/minute/centimeter^2
914	switchVmax_efflux_1	parameter	PBPK_MADAM	0	0	micromole/minute/centimeter^2
915	switch_SFinflux_1	parameter	PBPK_MADAM	1	1	dimensionless
916	switch_SFefflux_1	parameter	PBPK_MADAM	1	1	dimensionless
917	switch_SFgutmet_1	parameter	PBPK_MADAM	1	1	dimensionless
918	switch_SFdiffapi_1	parameter	PBPK_MADAM	1	1	dimensionless
919	switch_SFdiffbaso_1	parameter	PBPK_MADAM	1	1	dimensionless
920	zero_1	parameter	PBPK_MADAM	0	0	micromole/minute/centimeter^2
921	CLINT_metabolism_1	parameter	PBPK_MADAM	1	1	milliliter/minute
922	metabolism_factor_duo_1	parameter	PBPK_MADAM	1	1	dimensionless
923	metabolism_factor_jej1_1	parameter	PBPK_MADAM	1	1	dimensionless
924	metabolism_factor_jej2	parameter	PBPK_MADAM	1	1	dimensionless

	Quantity Name	Type	Scope	Value	Initial Value	Units
	_1					
925	metabolism_factor_ill1_1	parameter	PBPK_MADAM	1	1	dimensionless
926	metabolism_factor_ill2_1	parameter	PBPK_MADAM	1	1	dimensionless
927	metabolism_factor_ill3_1	parameter	PBPK_MADAM	1	1	dimensionless
928	metabolism_factor_ill4_1	parameter	PBPK_MADAM	1	1	dimensionless
929	DIFF_duo_1	parameter	PBPK_MADAM	1	13.8	centimeter^3/minute
930	DIFF_jej1_1	parameter	PBPK_MADAM	1	27.24	centimeter^3/minute
931	DIFF_jej2_1	parameter	PBPK_MADAM	1	19.08	centimeter^3/minute
932	DIFF_ill1_1	parameter	PBPK_MADAM	1	15.24	centimeter^3/minute
933	DIFF_ill2_1	parameter	PBPK_MADAM	1	15.24	centimeter^3/minute
934	DIFF_ill3_1	parameter	PBPK_MADAM	1	15	centimeter^3/minute
935	DIFF_ill4_1	parameter	PBPK_MADAM	1	14.4	centimeter^3/minute
936	DIFF_BASO_duo_1	parameter	PBPK_MADAM	1	0.35258	milliliter/minute
937	DIFF_BASO_jej1_1	parameter	PBPK_MADAM	1	1.358	milliliter/minute
938	DIFF_BASO_jej2_1	parameter	PBPK_MADAM	1	1.358	milliliter/minute
939	DIFF_BASO_ill1_1	parameter	PBPK_MADAM	1	0.90893	milliliter/minute
940	DIFF_BASO_ill2_1	parameter	PBPK_MADAM	1	0.90893	milliliter/minute
941	DIFF_BASO_ill3_1	parameter	PBPK_MADAM	1	0.90893	milliliter/minute
942	DIFF_BASO_ill4_1	parameter	PBPK_MADAM	1	0.90893	milliliter/minute
943	CLINT_influx_baso_DUO_1	parameter	PBPK_MADAM	1	0.35258	milliliter/minute
944	CLINT_influx_baso_JEJ1_1	parameter	PBPK_MADAM	1	1.358	milliliter/minute

	Quantity Name	Type	Scope	Value	Initial Value	Units
945	CLINT_influx_baso_JEJ2_1	parameter	PBPK_MADAM	1	1.358	milliliter/minute
946	CLINT_influx_baso_ILL1_1	parameter	PBPK_MADAM	1	0.90893	milliliter/minute
947	CLINT_influx_baso_ILL2_1	parameter	PBPK_MADAM	1	0.90893	milliliter/minute
948	CLINT_influx_baso_ILL3_1	parameter	PBPK_MADAM	1	0.90893	milliliter/minute
949	CLINT_influx_baso_ILL4_1	parameter	PBPK_MADAM	1	0	milliliter/minute
950	Qmuc_DUO_1	parameter	PBPK_MADAM	1	26.9235	milliliter/minute
951	Qmuc_JEJ1_1	parameter	PBPK_MADAM	1	74.0397	milliliter/minute
952	Qmuc_JEJ2_1	parameter	PBPK_MADAM	1	74.0397	milliliter/minute
953	Qmuc_ILL1_1	parameter	PBPK_MADAM	1	32.7365	milliliter/minute
954	Qmuc_ILL2_1	parameter	PBPK_MADAM	1	32.7365	milliliter/minute
955	Qmuc_ILL3_1	parameter	PBPK_MADAM	1	32.7365	milliliter/minute
956	Qmuc_ILL4_1	parameter	PBPK_MADAM	1	32.7365	milliliter/minute
957	switch_liverFlag_1	parameter	PBPK_MADAM	1	1	dimensionless
958	k_Liver_Venous_1	parameter	PBPK_MADAM	1	105.3302	liter/hour
959	k_artery_serosa_1	parameter	PBPK_MADAM	1	53.0431	liter/hour
960	k_serosa_liver_1	parameter	PBPK_MADAM	1	66.5123	liter/hour
961	k_liver_metabolites_1	parameter	PBPK_MADAM	1	1.0507	liter/hour
962	k_liver_bile_1	parameter	PBPK_MADAM	1	0.098292	liter/hour
963	Kp_serosa_1	parameter	PBPK_MADAM	1	0.51837	dimensionless
964	drug_Kp_serosa_raw_1	parameter	PBPK_MADAM	1	1	dimensionless

	Quantity Name	Type	Scope	Value	Initial Value	Units
965	HPeff_est_baso_1	parameter	PBPK_MADAM	1	1.6667e-05	centimeter/second
966	diff_baso_1	parameter	PBPK_MADAM	1	1	microliter/minute/centimeter^2
967	CL_inf_baso_1	parameter	PBPK_MADAM	1	1	microliter/minute/centimeter^2
968	CL_inf_api_1	parameter	PBPK_MADAM	1	1	microliter/minute/centimeter^2
969	HPeff_est_1	parameter	PBPK_MADAM	0.0001	1.6667e-05	centimeter/second
970	diff_api_1	parameter	PBPK_MADAM	1	1	microliter/minute/centimeter^2
971	CL_eff_1	parameter	PBPK_MADAM	1	1	microliter/minute/centimeter^2
972	HHSTOMACH_1	parameter	PBPK_MADAM	1	1.3162	dimensionless
973	SOLINT_1	parameter	PBPK_MADAM	1476.373	1	milligram/liter
974	HHDUO_1	parameter	PBPK_MADAM	1	1	dimensionless
975	HHJEJ1_1	parameter	PBPK_MADAM	1	1	dimensionless
976	HHJEJ2_1	parameter	PBPK_MADAM	1	1	dimensionless
977	HHILL1_1	parameter	PBPK_MADAM	1	1	dimensionless
978	HHILL2_1	parameter	PBPK_MADAM	1	1	dimensionless
979	HHILL3_1	parameter	PBPK_MADAM	1	1	dimensionless
980	HHILL4_1	parameter	PBPK_MADAM	1	1	dimensionless
981	HHINT_1	parameter	PBPK_MADAM	1	1	dimensionless
982	SOLFASSIF_1	parameter	PBPK_MADAM	1	1	milligram/liter
983	REFPHSOL_1	parameter	PBPK_MADAM	7.4	7.4	dimensionless
984	drug_inputFlag_1	parameter	PBPK_MADAM	1	1	dimensionless
985	drug_pKABase1_1	parameter	PBPK_MADAM	1	1	dimensionless
986	drug_pKABase2_1	parameter	PBPK_MADAM	1	1	dimensionless

	Quantity Name	Type	Scope	Value	Initial Value	Units
987	drug_pKAAcid1_1	parameter	PBPK_MADAM	1	1	dimensionless
988	drug_pKAAcid2_1	parameter	PBPK_MADAM	1	1	dimensionless
989	PSIZE_1	parameter	PBPK_MADAM	0.0005	0.0005	centimeter
990	PDENSITY_1	parameter	PBPK_MADAM	1000000	1000000	microgram/milliliter
991	DLT_1	parameter	PBPK_MADAM	0.003	0.003	centimeter
992	DIFFCOEFF_1	parameter	PBPK_MADAM	0.0001	0.0001	centimeter^2/minute
993	efflux_inhib_duo	parameter	PBPK_MADAM	1	1	dimensionless
994	switch_efflux_inhib_1	parameter	PBPK_MADAM	1	1	dimensionless
995	drug_efflux_Ki	parameter	PBPK_MADAM	1	4	micromole/liter
996	efflux_inhib_jej1	parameter	PBPK_MADAM	1	1	dimensionless
997	efflux_inhib_jej2	parameter	PBPK_MADAM	1	1	dimensionless
998	efflux_inhib_ill1	parameter	PBPK_MADAM	1	1	dimensionless
999	efflux_inhib_ill2	parameter	PBPK_MADAM	1	1	dimensionless
1000	efflux_inhib_ill3	parameter	PBPK_MADAM	1	1	dimensionless
1001	efflux_inhib_ill4	parameter	PBPK_MADAM	1	1	dimensionless
1002	Kp_liver_1	parameter	PBPK_MADAM	1	0.51837	dimensionless
1003	drug_Kp_liver_raw_1	parameter	PBPK_MADAM	1	1	dimensionless
1004	LOGP_1	parameter	PBPK_MADAM	1	1	dimensionless
1005	LOGSR_1	parameter	PBPK_MADAM	1	3.02	dimensionless
1006	MW_1	parameter	PBPK_MADAM	1	1	microgram/micromole
1007	pKA_1	parameter	PBPK_MADAM	1	1	dimensionless
1008	CLINT_efflux_baso_DUO	parameter	PBPK_MADAM	1	0	milliliter/minute

	Quantity Name	Type	Scope	Value	Initial Value	Units
1009	CLINT_efflux_baso_JEJ1	parameter	PBPK_MADAM	1	0	milliliter/minute
1010	CLINT_efflux_baso_JEJ2	parameter	PBPK_MADAM	1	0	milliliter/minute
1011	CLINT_efflux_baso_ILL1	parameter	PBPK_MADAM	1	0	milliliter/minute
1012	CLINT_efflux_baso_ILL2	parameter	PBPK_MADAM	1	0	milliliter/minute
1013	CLINT_efflux_baso_ILL3	parameter	PBPK_MADAM	1	0	milliliter/minute
1014	CLINT_efflux_baso_ILL4	parameter	PBPK_MADAM	1	0	milliliter/minute
1015	CL_eff_baso	parameter	PBPK_MADAM	0	0	microliter/minute/centimeter^2
1016	switch_SFefflux_baso	parameter	PBPK_MADAM	1	1	dimensionless
1017	baso_efflux_factor_duo	parameter	PBPK_MADAM	1	1	dimensionless
1018	baso_efflux_factor_jej1	parameter	PBPK_MADAM	1	1	dimensionless
1019	baso_efflux_factor_jej2	parameter	PBPK_MADAM	1	1	dimensionless
1020	baso_efflux_factor_ill1	parameter	PBPK_MADAM	1	1	dimensionless
1021	baso_efflux_factor_ill2	parameter	PBPK_MADAM	1	1	dimensionless
1022	baso_efflux_factor_ill3	parameter	PBPK_MADAM	1	1	dimensionless
1023	baso_efflux_factor_ill4	parameter	PBPK_MADAM	1	1	dimensionless
1024	switchVmax_efflux_baso	parameter	PBPK_MADAM	0.00016	0.00016	micromole/minute/centimeter^2
1025	drug_Km_efflux_baso	parameter	PBPK_MADAM	89	89	micromole/liter
1026	switchVmax_efflux_baso_1	parameter	PBPK_MADAM	0	0	micromole/minute/centimeter^2
1027	CLINT_efflux_baso_DUO_1	parameter	PBPK_MADAM	1	0	milliliter/minute

	Quantity Name	Type	Scope	Value	Initial Value	Units
1028	switch_SFefflux_baso_1	parameter	PBPK_MADAM	1	1	dimensionless
1029	drug_Km_efflux_baso_1	parameter	PBPK_MADAM	1	1	micromole/liter
1030	CLINT_efflux_baso_JEJ1_1	parameter	PBPK_MADAM	1	0	milliliter/minute
1031	CLINT_efflux_baso_JEJ2_1	parameter	PBPK_MADAM	1	0	milliliter/minute
1032	CLINT_efflux_baso_ILL1_1	parameter	PBPK_MADAM	1	0	milliliter/minute
1033	CLINT_efflux_baso_ILL2_1	parameter	PBPK_MADAM	1	0	milliliter/minute
1034	CLINT_efflux_baso_ILL3_1	parameter	PBPK_MADAM	1	0	milliliter/minute
1035	CLINT_efflux_baso_ILL4_1	parameter	PBPK_MADAM	1	0	milliliter/minute
1036	CL_eff_baso_1	parameter	PBPK_MADAM	0	0	microliter/minute/centimeter^2

Initial Assignments

	Initial Assignments	Initial Value
1	k_Liver_IC_S5_Bile = drug_PSbileg*switch_SFbile*phys_HPGL*(phys_BW*phys_Normalized_weight_liver_tissue*Specific_volume)/Specific_volume/5*drug_fuLiver	0
2	k_Liver_IC_S4_Bile = drug_PSbileg*switch_SFbile*phys_HPGL*(phys_BW*phys_Normalized_weight_liver_tissue*Specific_volume)/Specific_volume/5*drug_fuLiver	0
3	k_Liver_IC_S3_Bile = drug_PSbileg*switch_SFbile*phys_HPGL*(phys_BW*phys_Normalized_weight_liver_tissue*Specific_volume)/Specific_volume/5*drug_fuLiver	0
4	k_Liver_IC_S2_Bile = drug_PSbileg*switch_SFbile*phys_HPGL*(phys_BW*phys_Normalized_weight_liver_tissue*Specific_volume)/Specific_volume/5*drug_fuLiver	0
5	k_Liver_IC_S1_Bile = drug_PSbileg*switch_SFbile*phys_HPGL*(phys_BW*phys_Normalized_weight_liver_tissue*Specific_volume)/Specific_volume/5*drug_fuLiver	0
6	k_Liver_EC_S4_Liver_EC_S5 = Q_li	84

	Initial Assignments	Initial Value
7	k_Liver_EC_S3_Liver_EC_S4 = Q_li	84
8	k_Liver_EC_S2_Liver_EC_S3 = Q_li	84
9	k_Liver_EC_S1_Liver_EC_S2 = Q_li	84
10	k_Liver_IC_S5_Liver_EC_S5 = drug_PSdifg*switch_SFdiff*phys_HPGL*(phys_BW*phys_Normalized_weight_liver_tissue*Specific_volume)/Specific_volume/5*drug_fuLiver	0.15452
11	k_Liver_IC_S4_Liver_EC_S4 = drug_PSdifg*switch_SFdiff*phys_HPGL*(phys_BW*phys_Normalized_weight_liver_tissue*Specific_volume)/Specific_volume/5*drug_fuLiver	0.15452
12	k_Liver_IC_S2_Liver_EC_S2 = drug_PSdifg*switch_SFdiff*phys_HPGL*(phys_BW*phys_Normalized_weight_liver_tissue*Specific_volume)/Specific_volume/5*drug_fuLiver	0.15452
13	k_Liver_IC_S1_Liver_EC_S1 = drug_PSdifg*switch_SFdiff*phys_HPGL*(phys_BW*phys_Normalized_weight_liver_tissue*Specific_volume)/Specific_volume/5*drug_fuLiver	0.15452
14	k_rest_venous = Qrest/Kp_rest*drug_BRP	1.6425
15	k_artery_spleen = Q_artery_spleen	8.4
16	k_artery_gut = Q_artery_gut	71.4
17	k_gut_liver = Q_gut_liver/Kp_gut*drug_BRP	20.9108
18	k_spleen_liver = Q_spleen_liver/Kp_spleen*drug_BRP	4.4881
19	k_artery_rest = Qrest	4.2
20	k_kidney_venous = Qkidney/Kp_kidney*drug_BRP	21.3472
21	k_muscle_venous = Qmuscle/Kp_muscle*drug_BRP	30.5359
22	k_brain_venous = Qbrain/Kp_brain*drug_BRP	57.3582
23	k_skin_venous = Qskin/Kp_skin*drug_BRP	11.8896
24	k_bone_venous = Qbone/Kp_bone*drug_BRP	16.87
25	k_heart_venous = Qheart/Kp_heart*drug_BRP	3.5143
26	k_adipos_venous = Qadipose/Kp_adipose*drug_BRP	25.3489
27	k_artery_muscle = Qmuscle	58.8
28	k_artery_adipos = Qadipose	16.8
29	k_artery_bone = Qbone	16.8
30	k_artery_skin = Qskin	16.8
31	k_artery_kidney = Qkidney	63
32	k_artery_brain = Qbrain	42
33	k_venous_lung = Qlung	336
34	k_lung_artery = Qlung/Kp_lung*drug_BRP	112.8085
35	k_artery_heart = Qheart	12.6

	Initial Assignments	Initial Value
36	k_Liver_Venous = Q_li/Kp_liver*drug_BRP	14.1602
37	k_Liver_IC_S3_Liver__EC_S3 = drug_PSdifg*switch_SFdiff*phys_HPGL*(phys_BW*phys_Normalized_weight_liver_tissue*Specific_volume)/Specific_volume/5*drug_fuLiver	0.15452
38	k_artery_liver = Q_artery_liver	4.2
39	Venous = phys_BW*phys_Normalized_weight_venous*Specific_volume	3.598
40	Lung = phys_BW*phys_Normalized_weight_lung*Specific_volume	0.56
41	Kidney = phys_BW*phys_Normalized_weight_kidney*Specific_volume	0.35
42	Brain = phys_BW*phys_Normalized_weight_brain*Specific_volume	1.47
43	Muscle = phys_BW*phys_Normalized_weight_muscle*Specific_volume	29.12
44	Adipose = phys_BW*phys_Normalized_weight_adipose*Specific_volume	13.79
45	Heart = phys_BW*phys_Normalized_weight_heart*Specific_volume	0.35
46	Skin = phys_BW*phys_Normalized_weight_skin*Specific_volume	2.87
47	Bone = phys_BW*phys_Normalized_weight_bone*Specific_volume	11.06
48	Rest = phys_BW*phys_Normalized_weight_remainder*Specific_volume	7
49	Artery = phys_BW*phys_Normalized_weight_artery*Specific_volume	1.799
50	Spleen = phys_BW*phys_Normalized_weight_spleen*Specific_volume	0.21
51	Gut = phys_BW*phys_Normalized_weight_gut*Specific_volume	1.26
52	Liver_EC_S1 = phys_BW*phys_Normalized_weight_liver_blood*Specific_volume/5	0.0686

	Initial Assignments	Initial Value
53	Liver_EC_S2 = phys_BW*phys_Normalized_weight_liver_blood*Specific_volume/5	0.0686
54	Liver_EC_S3 = phys_BW*phys_Normalized_weight_liver_blood*Specific_volume/5	0.0686
55	Liver_EC_S4 = phys_BW*phys_Normalized_weight_liver_blood*Specific_volume/5	0.0686
56	Liver_EC_S5 = phys_BW*phys_Normalized_weight_liver_blood*Specific_volume/5	0.0686
57	Liver_IC_S5 = phys_BW*phys_Normalized_weight_liver_tissue*Specific_volume/5	0.252
58	Liver_IC_S4 = phys_BW*phys_Normalized_weight_liver_tissue*Specific_volume/5	0.252
59	Liver_IC_S3 = phys_BW*phys_Normalized_weight_liver_tissue*Specific_volume/5	0.252
60	Liver_IC_S2 = phys_BW*phys_Normalized_weight_liver_tissue*Specific_volume/5	0.252
61	Liver_IC_S1 = phys_BW*phys_Normalized_weight_liver_tissue*Specific_volume/5	0.252
62	Q_artery_gut = phys_BW*phys_Normalized_Q_gut	71.4
63	Q_artery_liver = Q_li-Q_artery_gut-Q_artery_spleen	4.2
64	Q_artery_spleen = phys_BW*phys_Normalized_Q_spleen	8.4
65	Q_gut_liver = phys_BW*phys_Normalized_Q_gut	71.4
66	Q_spleen_liver = phys_BW*phys_Normalized_Q_spleen	8.4
67	Qadipose = phys_BW*phys_Normalized_Q_adipose	16.8
68	Qbone = phys_BW*phys_Normalized_Q_bone	16.8
69	Qbrain = phys_BW*phys_Normalized_Q_brain	42
70	Qheart = phys_BW*phys_Normalized_Q_heart	12.6
71	Qkidney = phys_BW*phys_Normalized_Q_kidney	63
72	Q_li = phys_BW*phys_Normalized_Q_liver	84
73	Qlung = phys_BW*phys_Normalized_Q_lung	336
74	Qmuscle = phys_BW*phys_Normalized_Q_muscle	58.8
75	Qrest = phys_BW*phys_Normalized_Q_remainder	4.2
76	Qskin = phys_BW*phys_Normalized_Q_skin	16.8
77	drug_fB = drug_fuplasma/drug_BRP	0.18

	Initial Assignments	Initial Value
78	k_venous_urine_CLR = drug_CLrenal*switch_SFrenal	7.2
79	k_artery_testes = Qtestes	0
80	k_testes_venous = Qtestes/Kp_testes*drug_BRP	0
81	Qtestes = phys_BW*phys_Normalized_Q_testes	0
82	k_Liver_IC_S2_Liver_EC_S2_efflux = switch_SFeff*drug_CLeffluxHep*phys_HPGL*(phys_BW*phys_Normalized_weight_liver_tissue*Specific_volume)/Specific_volume/5*drug_fuLiver	0
83	k_Liver_IC_S3_Liver_EC_S3_efflux = switch_SFeff*drug_CLeffluxHep*phys_HPGL*(phys_BW*phys_Normalized_weight_liver_tissue*Specific_volume)/Specific_volume/5*drug_fuLiver	0
84	k_Liver_IC_S4_Liver_EC_S4_efflux = switch_SFeff*drug_CLeffluxHep*phys_HPGL*(phys_BW*phys_Normalized_weight_liver_tissue*Specific_volume)/Specific_volume/5*drug_fuLiver	0
85	k_Liver_IC_S5_Liver_EC_S5_efflux = switch_SFeff*drug_CLeffluxHep*phys_HPGL*(phys_BW*phys_Normalized_weight_liver_tissue*Specific_volume)/Specific_volume/5*drug_fuLiver	0
86	k_Liver_IC_S1_Liver_EC_S1_efflux = switch_SFeff*drug_CLeffluxHep*phys_HPGL*(phys_BW*phys_Normalized_weight_liver_tissue*Specific_volume)/Specific_volume/5*drug_fuLiver	0
87	Testes = phys_BW*phys_Normalized_weight_testes*Specific_volume	0.07
88	Kp_adipose = drug_Kp_adipose_raw*switch_SFKp	0.66275
89	Kp_bone = drug_Kp_bone_raw*switch_SFKp	0.99585
90	Kp_brain = drug_Kp_brain_raw*switch_SFKp	0.73224
91	Kp_gut = drug_Kp_gut_raw*switch_SFKp	3.4145
92	Kp_heart = drug_Kp_heart_raw*switch_SFKp	3.5854
93	Kp_kidney = drug_Kp_kidney_raw*switch_SFKp	2.9512
94	Kp_lung = drug_Kp_lung_raw*switch_SFKp	2.9785
95	Kp_muscle = drug_Kp_muscle_raw*switch_SFKp	1.9256
96	Kp_skin = drug_Kp_skin_raw*switch_SFKp	1.413
97	Kp_spleen = drug_Kp_spleen_raw*switch_SFKp	1.8716
98	Kp_testes = drug_Kp_testes_raw*switch_SFKp	1
99	k_venous_urine_GFR = drug_GFR*drug_fuplasma*switch_SFrenal	0
100	drug_GFR = drug_FR*Qkidney	0
101	Kp_rest = switch_slow_dist_Kp*drug_Kp_rest_raw+(1-switch_slow_dist_Kp)*drug_Kp_rest_raw*switch_SFKp	2.557

	Initial Assignments	Initial Value
102	k_Liver_EC_S4_Liver_EC_S5_1 = Q_li_1	84
103	k_Liver_EC_S3_Liver_EC_S4_1 = Q_li_1	84
104	k_Liver_EC_S2_Liver_EC_S3_1 = Q_li_1	84
105	k_Liver_EC_S1_Liver_EC_S2_1 = Q_li_1	84
106	k_Liver_IC_S5_Liver_EC_S5_1 = drug_PSdifg_1*switch_SFdiff_1*phys_HPGL*(phys_BW*phys_Normalized_weight_liver_tissue*Specific_volume)/Specific_volume/5*drug_fuLiver_1	0.26572
107	k_Liver_IC_S4_Liver_EC_S4_1 = drug_PSdifg_1*switch_SFdiff_1*phys_HPGL*(phys_BW*phys_Normalized_weight_liver_tissue*Specific_volume)/Specific_volume/5*drug_fuLiver_1	0.26572
108	k_Liver_IC_S2_Liver_EC_S2_1 = drug_PSdifg_1*switch_SFdiff_1*phys_HPGL*(phys_BW*phys_Normalized_weight_liver_tissue*Specific_volume)/Specific_volume/5*drug_fuLiver_1	0.26572
109	k_Liver_IC_S1_Liver_EC_S1_1 = drug_PSdifg_1*switch_SFdiff_1*phys_HPGL*(phys_BW*phys_Normalized_weight_liver_tissue*Specific_volume)/Specific_volume/5*drug_fuLiver_1	0.26572
110	k_rest_venous_1 = Qrest_1/Kp_rest_1*drug_BRP_1	420
111	k_artery_spleen_1 = Q_artery_spleen_1	8.4
112	k_artery_gut_1 = Q_artery_gut_1	71.4
113	k_gut_liver_1 = Q_gut_liver_1/Kp_gut_1*drug_BRP_1	190.4907
114	k_spleen_liver_1 = Q_spleen_liver_1/Kp_spleen_1*drug_BRP_1	30.0943
115	k_artery_rest_1 = Qrest_1	4.2
116	k_kidney_venous_1 = Qkidney_1/Kp_kidney_1*drug_BRP_1	303.837
117	k_muscle_venous_1 = Qmuscle_1/Kp_muscle_1*drug_BRP_1	1474.6224
118	k_brain_venous_1 = Qbrain_1/Kp_brain_1*drug_BRP_1	164.5784
119	k_skin_venous_1 = Qskin_1/Kp_skin_1*drug_BRP_1	56.9352
120	k_bone_venous_1 = Qbone_1/Kp_bone_1*drug_BRP_1	35.1101
121	k_heart_venous_1 = Qheart_1/Kp_heart_1*drug_BRP_1	78.9976
122	k_adipos_venous_1 = Qadipose_1/Kp_adipose_1*drug_BRP_1	46.8134
123	k_artery_muscle_1 = Qmuscle_1	58.8
124	k_artery_adipos_1 = Qadipose_1	16.8
125	k_artery_bone_1 = Qbone_1	16.8
126	k_artery_skin_1 = Qskin_1	16.8

	Initial Assignments	Initial Value
127	k_artery_kidney_1 = Qkidney_1	63
128	k_artery_brain_1 = Qbrain_1	42
129	k_venous_lung_1 = Qlung_1	336
130	k_lung_artery_1 = Qlung_1/Kp_lung_1*drug_BRP_1	1053.3017
131	k_artery_heart_1 = Qheart_1	12.6
132	k_Liver_EC_S5_Venous_1 = Q_li_1	84
133	k_Liver_IC_S3_Liver__EC_S3_1 = drug_PSdifg_1*switch_SFdiff_1*phys_HPGL*(phys_BW*phys_Normalized_weight_liver_tissue*Specific_volume)/Specific_volume/5*drug_fuLiver_1	0.26572
134	k_artery_liver_1 = Q_artery_liver_1	4.2
135	Venous_1 = phys_BW*phys_Normalized_weight_venous*Specific_volume	3.598
136	Lung_1 = phys_BW*phys_Normalized_weight_lung*Specific_volume	0.56
137	Kidney_1 = phys_BW*phys_Normalized_weight_kidney*Specific_volume	0.35
138	Brain_1 = phys_BW*phys_Normalized_weight_brain*Specific_volume	1.47
139	Muscle_1 = phys_BW*phys_Normalized_weight_muscle*Specific_volume	29.12
140	Adipose_1 = phys_BW*phys_Normalized_weight_adipose*Specific_volume	13.79
141	Heart_1 = phys_BW*phys_Normalized_weight_heart*Specific_volume	0.35
142	Skin_1 = phys_BW*phys_Normalized_weight_skin*Specific_volume	2.87
143	Bone_1 = phys_BW*phys_Normalized_weight_bone*Specific_volume	11.06
144	Rest_1 = phys_BW*phys_Normalized_weight_remainder*Specific_volume	7
145	Artery_1 = phys_BW*phys_Normalized_weight_artery*Specific_volume	1.799
146	Spleen_1 = phys_BW*phys_Normalized_weight_spleen*Specific_volume	0.21

	Initial Assignments	Initial Value
147	Gut_1 = phys_BW*phys_Normalized_weight_gut*Specific_volume	1.26
148	Liver_EC_S1_1 = phys_BW*phys_Normalized_weight_liver_blood*Specific_volume/5	0.0686
149	Liver_EC_S2_1 = phys_BW*phys_Normalized_weight_liver_blood*Specific_volume/5	0.0686
150	Liver_EC_S3_1 = phys_BW*phys_Normalized_weight_liver_blood*Specific_volume/5	0.0686
151	Liver_EC_S4_1 = phys_BW*phys_Normalized_weight_liver_blood*Specific_volume/5	0.0686
152	Liver_EC_S5_1 = phys_BW*phys_Normalized_weight_liver_blood*Specific_volume/5	0.0686
153	Liver_IC_S5_1 = phys_BW*phys_Normalized_weight_liver_tissue*Specific_volume/5	0.252
154	Liver_IC_S4_1 = phys_BW*phys_Normalized_weight_liver_tissue*Specific_volume/5	0.252
155	Liver_IC_S3_1 = phys_BW*phys_Normalized_weight_liver_tissue*Specific_volume/5	0.252
156	Liver_IC_S2_1 = phys_BW*phys_Normalized_weight_liver_tissue*Specific_volume/5	0.252
157	Liver_IC_S1_1 = phys_BW*phys_Normalized_weight_liver_tissue*Specific_volume/5	0.252
158	Q_artery_gut_1 = phys_BW*phys_Normalized_Q_gut	71.4
159	Q_artery_liver_1 = Q_li-Q_artery_gut-Q_artery_spleen	4.2
160	Q_artery_spleen_1 = phys_BW*phys_Normalized_Q_spleen	8.4
161	Q_gut_liver_1 = phys_BW*phys_Normalized_Q_gut	71.4
162	Q_spleen_liver_1 = phys_BW*phys_Normalized_Q_spleen	8.4
163	Qadipose_1 = phys_BW*phys_Normalized_Q_adipose	16.8
164	Qbone_1 = phys_BW*phys_Normalized_Q_bone	16.8
165	Qbrain_1 = phys_BW*phys_Normalized_Q_brain	42
166	Qheart_1 = phys_BW*phys_Normalized_Q_heart	12.6

	Initial Assignments	Initial Value
167	Qkidney_1 = phys_BW*phys_Normalized_Q_kidney	63
168	Q_li_1 = phys_BW*phys_Normalized_Q_liver	84
169	Qlung_1 = phys_BW*phys_Normalized_Q_lung	336
170	Qmuscle_1 = phys_BW*phys_Normalized_Q_muscle	58.8
171	Qrest_1 = phys_BW*phys_Normalized_Q_remainder	4.2
172	Qskin_1 = phys_BW*phys_Normalized_Q_skin	16.8
173	drug_fB_1 = drug_fuplasma_1/drug_BRP_1	0.058769
174	k_venous_urine_CLR_1 = drug_CLrenal_1*switch_SFrenal_1	0
175	k_artery_testes_1 = Qtestes_1	0
176	k_testes_venous_1 = Qtestes_1/Kp_testes*drug_BRP_1	0
177	Qtestes_1 = phys_BW*phys_Normalized_Q_testes	0
178	k_Liver_IC_S2_Liver_EC_S2_efflux_1 = switch_SFeff_1*drug_CLeffluxHep_1*phys_HPGL*(phys_BW*phys_Normalized_weight_liver_tissue*Specific_volume)/Specific_volume/5*drug_fuLiver_1	0
179	k_Liver_IC_S3_Liver_EC_S3_efflux_1 = switch_SFeff_1*drug_CLeffluxHep_1*phys_HPGL*(phys_BW*phys_Normalized_weight_liver_tissue*Specific_volume)/Specific_volume/5*drug_fuLiver_1	0
180	k_Liver_IC_S4_Liver_EC_S4_efflux_1 = switch_SFeff_1*drug_CLeffluxHep_1*phys_HPGL*(phys_BW*phys_Normalized_weight_liver_tissue*Specific_volume)/Specific_volume/5*drug_fuLiver_1	0
181	k_Liver_IC_S5_Liver_EC_S5_efflux_1 = switch_SFeff_1*drug_CLeffluxHep_1*phys_HPGL*(phys_BW*phys_Normalized_weight_liver_tissue*Specific_volume)/Specific_volume/5*drug_fuLiver_1	0
182	k_Liver_IC_S1_Liver_EC_S1_efflux_1 = switch_SFeff_1*drug_CLeffluxHep_1*phys_HPGL*(phys_BW*phys_Normalized_weight_liver_tissue*Specific_volume)/Specific_volume/5*drug_fuLiver_1	0
183	Testes_1 = phys_BW*phys_Normalized_weight_testes*Specific_volume	0.07
184	Kp_adipose_1 = drug_Kp_adipose_raw_1*switch_SFKp_1	0.23327
185	Kp_bone_1 = drug_Kp_bone_raw_1*switch_SFKp_1	0.31102
186	Kp_brain_1 = drug_Kp_brain_raw_1*switch_SFKp_1	0.16588
187	Kp_gut_1 = drug_Kp_gut_raw_1*switch_SFKp_1	0.24363
188	Kp_heart_1 = drug_Kp_heart_raw_1*switch_SFKp_1	0.10367
189	Kp_kidney_1 = drug_Kp_kidney_raw_1*switch_SFKp_1	0.13478
190	Kp_lung_1 = drug_Kp_lung_raw_1*switch_SFKp_1	0.20735

	Initial Assignments	Initial Value
191	Kp_muscle_1 = drug_Kp_muscle_raw_1*switch_SFKp_1	0.025919
192	Kp_skin_1 = drug_Kp_skin_raw_1*switch_SFKp_1	0.1918
193	Kp_spleen_1 = drug_Kp_spleen_raw_1*switch_SFKp_1	0.18143
194	Kp_testes_1 = drug_Kp_testes_raw_1*switch_SFKp_1	0.51837
195	k_venous_urine_GFR_1 = drug_GFR_1*drug_fuplasma_1*switch_SFrenal_1	0
196	drug_GFR_1 = drug_FR_1*Qkidney_1	0
197	Kp_rest_1 = switch_slow_dist_Kp_1*drug_Kp_rest_raw_1+(1-switch_slow_dist_Kp_1)*drug_Kp_rest_raw_1*switch_SFKp_1	0.0065
198	STOMACH = StomachLumenTotal/BW_average*phys_BW	0.05
199	QMUC = (Q_gut_liver/numIntestinalCompartments)*liter_to_milliliter/minute_per_hour	170
200	V_LUM_TOT = LumenTotal/BW_average*phys_BW	126.95
201	V_ONECOMP = V_LUM_TOT/numIntestinalCompartments	18.1357
202	V_MEM = (VGut/numIntestinalCompartments)*liter_to_milliliter	73.857
203	LOGSR = 0.75*LOGP+2.27	4.64
204	SOLINT = SOLFASSIF/HHINT	2.5704e-07
205	SOLIF_STOMACH = SOLINT*HHSTOMACH	7.9433
206	SOLIF_DUO = SOLINT*HHDUO	0.00010026
207	SOLIF_JEJ1 = SOLINT*HHJEJ1	7.969e-05
208	SOLIF_JEJ2 = SOLINT*HHJEJ2	6.3353e-05
209	SOLIF_ILL1 = SOLINT*HHILL1	4.0068e-05
210	SOLIF_ILL2 = SOLINT*HHILL2	2.5376e-05
211	SOLIF_ILL3 = SOLINT*HHILL3	5.2689e-06
212	SOLIF_ILL4 = SOLINT*HHILL4	1.2846e-05
213	HPeff_est = diff_api	0.00019833
214	DIFF = second_per_minute*HPeff_est*phys_ESA/numIntestinalCompartments	204
215	NI_DUO = 1/HHDUO	0.0025638
216	NI_JEJ1 = 1/HHJEJ1	0.0032255
217	NI_JEJ2 = 1/HHJEJ2	0.0040573
218	NI_ILL1 = 1/HHILL1	0.0064151
219	NI_ILL2 = 1/HHILL2	0.010129

	Initial Assignments	Initial Value
220	NI_ILL3 = 1/HHILL3	0.048784
221	NI_ILL4 = 1/HHILL4	0.020009
222	KD = 3*DIFFCOEFF/(PDENSITY*PSIZE*DLT)	1.5385e-05
223	VDUO = V_LUM_TOT*lumenvolumeRatio_DUO	0.03435
224	VJEJ1 = V_LUM_TOT*lumenvolumeRatio_JEJ1	0.0211
225	VJEJ2 = V_LUM_TOT*lumenvolumeRatio_JEJ2	0.0211
226	VILL1 = V_LUM_TOT*lumenvolumeRatio_ILL1	0.0126
227	VILL2 = V_LUM_TOT*lumenvolumeRatio_ILL2	0.0126
228	VILL3 = V_LUM_TOT*lumenvolumeRatio_ILL3	0.0126
229	VILL4 = V_LUM_TOT*lumenvolumeRatio_ILL4	0.0126
230	MDUO = VGut*volumeRatio_DUO*Gut_IC_fraction	0.037454
231	MJEJ1 = VGut*volumeRatio_JEJ1*Gut_IC_fraction	0.073785
232	MJEJ2 = VGut*volumeRatio_JEJ2*Gut_IC_fraction	0.051687
233	MILL1 = VGut*volumeRatio_ILL1*Gut_IC_fraction	0.0412
234	MILL2 = VGut*volumeRatio_ILL2*Gut_IC_fraction	0.0412
235	MILL3 = VGut*volumeRatio_ILL3*Gut_IC_fraction	0.04045
236	MILL4 = VGut*volumeRatio_ILL4*Gut_IC_fraction	0.038952
237	VGut = phys_BW*phys_Normalized_weight_enterocyte*Specific_volume	0.517
238	VillousDUO = VGut*volumeRatio_DUO*Gut_EC_fraction*drug_fQ	0.0057016
239	VillousJEJ1 = VGut*volumeRatio_JEJ1*Gut_EC_fraction*drug_fQ	0.011232
240	VillousJEJ2 = VGut*volumeRatio_JEJ2*Gut_EC_fraction*drug_fQ	0.0078682
241	VillousILL1 = VGut*volumeRatio_ILL1*Gut_EC_fraction*drug_fQ	0.0062718
242	VillousILL2 = VGut*volumeRatio_ILL2*Gut_EC_fraction*drug_fQ	0.0062718
243	VillousILL3 = VGut*volumeRatio_ILL3*Gut_EC_fraction*drug_fQ	0.0061578
244	VillousILL4 = VGut*volumeRatio_ILL4*Gut_EC_fraction*drug_fQ	0.0059297
245	Qmuc_DUO = drug_fQ*Q_artery_gut*flowRatio_DUO	26.9235
246	Qmuc_JEJ1 = drug_fQ*Q_artery_gut*flowRatio_JEJ1	74.0397
247	Qmuc_JEJ2 = drug_fQ*Q_artery_gut*flowRatio_JEJ2	74.0397
248	Qmuc_ILL1 = drug_fQ*Q_artery_gut*flowRatio_ILL1	32.7365
249	Qmuc_ILL2 = drug_fQ*Q_artery_gut*flowRatio_ILL2	32.7365

	Initial Assignments	Initial Value
250	Qmuc_ILL3 = drug_fQ*Q_artery_gut*flowRatio_ILL3	32.7365
251	Qmuc_ILL4 = drug_fQ*Q_artery_gut*flowRatio_ILL4	32.7365
252	Liver = phys_BW*(phys_Normalized_weight_liver_blood + phys_Normalized_weight_liver_tissue)*Specific_volume	1.603
253	Kp_liver = drug_Kp_liver_raw*switch_SFKp	5.9321
254	k_liver_bile = drug_PSbileg*switch_SFbile*phys_HPGL*(phys_BW*phys_Normalized_weight_liver_tissue*Specific_volume)/Specific_volume*drug_fuLiver	0
255	k_liver_metabolites = (switch_SFmet*((drug_HLM_CLint/drug_fumic*phys_MPGL)+(drug_CLmetg/drug_funic*phys_HPGL))*(phys_BW*phys_Normalized_weight_liver_tissue*Specific_volume)/Specific_volume)*drug_fuLiver	3.9865
256	Q_villi = phys_BW*phys_Normalized_Q_villi	336
257	DIFF_duo = HPeff_est*phys_Normalized_ESA*phys_BW*surfaceRatio_DUO	164.22
258	DIFF_jej1 = HPeff_est*phys_Normalized_ESA*phys_BW*surfaceRatio_JEJ1	324.156
259	DIFF_jej2 = HPeff_est*phys_Normalized_ESA*phys_BW*surfaceRatio_JEJ2	227.052
260	DIFF_ill1 = HPeff_est*phys_Normalized_ESA*phys_BW*surfaceRatio_ILL1	181.356
261	DIFF_ill2 = HPeff_est*phys_Normalized_ESA*phys_BW*surfaceRatio_ILL2	181.356
262	DIFF_ill3 = HPeff_est*phys_Normalized_ESA*phys_BW*surfaceRatio_ILL3	178.5
263	DIFF_ill4 = HPeff_est*phys_Normalized_ESA*phys_BW*surfaceRatio_ILL4	171.36
264	DIFF_BASO_duo = HPeff_est_baso*phys_Normalized_ESA_baso*phys_BW*basoSurfaceRatio_DUO	4.1957
265	DIFF_BASO_jej1 = HPeff_est_baso*phys_Normalized_ESA_baso*phys_BW*basoSurfaceRatio_JEJ1	16.1605
266	DIFF_BASO_jej2 = HPeff_est_baso*phys_Normalized_ESA_baso*phys_BW*basoSurfaceRatio_JEJ2	16.1605
267	DIFF_BASO_ill1 = HPeff_est_baso*phys_Normalized_ESA_baso*phys_	10.8162

	Initial Assignments	Initial Value
	BW*basoSurfaceRatio_ILL1	
268	DIFF_BASO_ill2 = HPeff_est_baso*phys_Normalized_ESA_baso*phys_BW*basoSurfaceRatio_ILL2	10.8162
269	DIFF_BASO_ill3 = HPeff_est_baso*phys_Normalized_ESA_baso*phys_BW*basoSurfaceRatio_ILL3	10.8162
270	DIFF_BASO_ill4 = HPeff_est_baso*phys_Normalized_ESA_baso*phys_BW*basoSurfaceRatio_ILL4	10.8162
271	k_artery_serosa = (1-drug_fQ)*Q_artery_gut	53.0431
272	k_serosa_liver = (1-drug_fQ)*Q_gut_liver/Kp_serosa*drug_BRP	15.5346
273	Kp_serosa = drug_Kp_serosa_raw*switch_SFKp	3.4145
274	HHINT = calculateHH(drug_inputFlag, REFPHSOL, drug_pKABase1, drug_pKABase2, drug_pKAAcid1, drug_pKAAcid2)	3890452.4499
275	HHSTOMACH = calculateHH(drug_inputFlag, pHStomach, drug_pKABase1, drug_pKABase2, drug_pKAAcid1, drug_pKAAcid2)	30902955.3251
276	HHDUO = calculateHH(drug_inputFlag, pHDuo, drug_pKABase1, drug_pKABase2, drug_pKAAcid1, drug_pKAAcid2)	390.0451
277	HHJEJ1 = calculateHH(drug_inputFlag, pHJej1, drug_pKABase1, drug_pKABase2, drug_pKAAcid1, drug_pKAAcid2)	310.0295
278	HHJEJ2 = calculateHH(drug_inputFlag, pHJej2, drug_pKABase1, drug_pKABase2, drug_pKAAcid1, drug_pKAAcid2)	246.4709
279	HHILL1 = calculateHH(drug_inputFlag, pHIll1, drug_pKABase1, drug_pKABase2, drug_pKAAcid1, drug_pKAAcid2)	155.8817
280	HHILL2 = calculateHH(drug_inputFlag, pHIll2, drug_pKABase1, drug_pKABase2, drug_pKAAcid1, drug_pKAAcid2)	98.7237
281	HHILL3 = calculateHH(drug_inputFlag, pHIll3, drug_pKABase1, drug_pKABase2, drug_pKAAcid1, drug_pKAAcid2)	20.4984
282	HHILL4 = calculateHH(drug_inputFlag, pHIll4, drug_pKABase1, drug_pKABase2, drug_pKAAcid1, drug_pKAAcid2)	49.9779
283	CLINT_influx_DUO = CL_inf_api*phys_Normalized_ESA*phys_BW*surfaceRatio_DUO	0
284	CLINT_influx_JEJ1 = CL_inf_api*phys_Normalized_ESA*phys_BW*surfaceRatio_JEJ1	0
285	CLINT_influx_JEJ2 = CL_inf_api*phys_Normalized_ESA*phys_BW*surfac	0

	Initial Assignments	Initial Value
	eRatio_JEJ2	
286	CLINT_influx_ILL1 = CL_inf_api*phys_Normalized_ESA*phys_BW*surfaceRatio_ILL1	0
287	CLINT_influx_ILL2 = CL_inf_api*phys_Normalized_ESA*phys_BW*surfaceRatio_ILL2	0
288	CLINT_influx_ILL3 = CL_inf_api*phys_Normalized_ESA*phys_BW*surfaceRatio_ILL3	0
289	CLINT_influx_ILL4 = CL_inf_api*phys_Normalized_ESA*phys_BW*surfaceRatio_ILL4	0
290	CLINT_efflux_DUO = CL_eff*phys_Normalized_ESA*phys_BW*surfaceRatio_DUO	0
291	CLINT_efflux_JEJ1 = CL_eff*phys_Normalized_ESA*phys_BW*surfaceRatio_JEJ1	0
292	CLINT_efflux_JEJ2 = CL_eff*phys_Normalized_ESA*phys_BW*surfaceRatio_JEJ2	0
293	CLINT_efflux_ILL1 = CL_eff*phys_Normalized_ESA*phys_BW*surfaceRatio_ILL1	0
294	CLINT_efflux_ILL2 = CL_eff*phys_Normalized_ESA*phys_BW*surfaceRatio_ILL2	0
295	CLINT_efflux_ILL3 = CL_eff*phys_Normalized_ESA*phys_BW*surfaceRatio_ILL3	0
296	CLINT_efflux_ILL4 = CL_eff*phys_Normalized_ESA*phys_BW*surfaceRatio_ILL4	0
297	HPeff_est_baso = diff_baso	0.00019833
298	CLINT_influx_baso_DUO = CL_inf_baso*phys_Normalized_ESA_baso*phys_BW*basoSurfaceRatio_DUO	0
299	CLINT_influx_baso_JEJ1 = CL_inf_baso*phys_Normalized_ESA_baso*phys_BW*basoSurfaceRatio_JEJ1	0
300	CLINT_influx_baso_JEJ2 = CL_inf_baso*phys_Normalized_ESA_baso*phys_BW*basoSurfaceRatio_JEJ2	0
301	CLINT_influx_baso_ILL1 = CL_inf_baso*phys_Normalized_ESA_baso*phys_BW*basoSurfaceRatio_ILL1	0
302	CLINT_influx_baso_ILL2 = CL_inf_baso*phys_Normalized_ESA_baso*phys_B	0

	Initial Assignments	Initial Value
	W*basoSurfaceRatio_ILL2	
303	CLINT_influx_baso_ILL3 = CL_inf_baso*phys_Normalized_ESA_baso*phys_BW*basoSurfaceRatio_ILL3	0
304	CLINT_influx_baso_ILL4 = CL_inf_baso*phys_Normalized_ESA_baso*phys_BW*basoSurfaceRatio_ILL4	0
305	Serosa = VGut*Gut_EC_fraction*(1-drug_fQ)	0.14284
306	diff_api = diff_baso	11.9
307	k_Liver_EC_S5_Venous = Q_li	84
308	phys_Normalized_ESA = phys_ESA/BW_average	1714.2857
309	phys_Normalized_ESA_baso = phys_ESA_baso/BW_average	95.7571
310	k_artery_serosa_1 = (1-drug_fQ)*Q_artery_gut_1	53.0431
311	k_serosa_liver_1 = (1-drug_fQ)*Q_gut_liver_1/Kp_serosa_1*drug_BRP_1	66.5123
312	Kp_serosa_1 = drug_Kp_serosa_raw_1*switch_SFKp_1	0.51837
313	Qmuc_JEJ1_1 = drug_fQ*Q_artery_gut*flowRatio_JEJ1	74.0397
314	Qmuc_DUO_1 = drug_fQ*Q_artery_gut*flowRatio_DUO	26.9235
315	Qmuc_JEJ2_1 = drug_fQ*Q_artery_gut*flowRatio_JEJ2	74.0397
316	Qmuc_ILL1_1 = drug_fQ*Q_artery_gut*flowRatio_ILL1	32.7365
317	Qmuc_ILL2_1 = drug_fQ*Q_artery_gut*flowRatio_ILL2	32.7365
318	Qmuc_ILL3_1 = drug_fQ*Q_artery_gut*flowRatio_ILL3	32.7365
319	Qmuc_ILL4_1 = drug_fQ*Q_artery_gut*flowRatio_ILL4	32.7365
320	HPeff_est_baso_1 = diff_baso_1	1.6667e-05
321	DIFF_BASO_duo_1 = HPeff_est_baso_1*phys_Normalized_ESA_baso*phys_BW*basoSurfaceRatio_DUO	0.35258
322	DIFF_BASO_jej1_1 = HPeff_est_baso_1*phys_Normalized_ESA_baso*phys_BW*basoSurfaceRatio_JEJ1	1.358
323	DIFF_BASO_jej2_1 = HPeff_est_baso_1*phys_Normalized_ESA_baso*phys_BW*basoSurfaceRatio_JEJ2	1.358
324	DIFF_BASO_ill1_1 = HPeff_est_baso_1*phys_Normalized_ESA_baso*phys_BW*basoSurfaceRatio_ILL1	0.90893

	Initial Assignments	Initial Value
325	DIFF_BASO_ill2_1 = HPeff_est_baso_1*phys_Normalized_ESA_baso*phys_BW*basoSurfaceRatio_ILL2	0.90893
326	DIFF_BASO_ill3_1 = HPeff_est_baso_1*phys_Normalized_ESA_baso*phys_BW*basoSurfaceRatio_ILL3	0.90893
327	DIFF_BASO_ill4_1 = HPeff_est_baso_1*phys_Normalized_ESA_baso*phys_BW*basoSurfaceRatio_ILL4	0.90893
328	CLINT_influx_baso_DUO_1 = CL_inf_baso_1*phys_Normalized_ESA_baso*phys_BW*basoSurfaceRatio_DUO	0.35258
329	CLINT_influx_baso_JEJ1_1 = CL_inf_baso_1*phys_Normalized_ESA_baso*phys_BW*basoSurfaceRatio_JEJ1	1.358
330	CLINT_influx_baso_JEJ2_1 = CL_inf_baso_1*phys_Normalized_ESA_baso*phys_BW*basoSurfaceRatio_JEJ2	1.358
331	CLINT_influx_baso_ILL1_1 = CL_inf_baso_1*phys_Normalized_ESA_baso*phys_BW*basoSurfaceRatio_ILL1	0.90893
332	CLINT_influx_baso_ILL2_1 = CL_inf_baso_1*phys_Normalized_ESA_baso*phys_BW*basoSurfaceRatio_ILL2	0.90893
333	CLINT_influx_baso_ILL3_1 = CL_inf_baso_1*phys_Normalized_ESA_baso*phys_BW*basoSurfaceRatio_ILL3	0.90893
334	CLINT_influx_baso_ILL4_1 = CL_inf_baso*phys_Normalized_ESA_baso*phys_BW*basoSurfaceRatio_ILL4	0
335	CLINT_influx_DUO_1 = CL_inf_api_1*phys_Normalized_ESA*phys_BW*surfaceRatio_DUO	13.8
336	CLINT_influx_JEJ1_1 = CL_inf_api_1*phys_Normalized_ESA*phys_BW*surfaceRatio_JEJ1	27.24
337	CLINT_influx_JEJ2_1 = CL_inf_api_1*phys_Normalized_ESA*phys_BW*surfaceRatio_JEJ2	19.08
338	CLINT_influx_ILL1_1 = CL_inf_api_1*phys_Normalized_ESA*phys_BW*surfaceRatio_ILL1	15.24
339	CLINT_influx_ILL2_1 = CL_inf_api_1*phys_Normalized_ESA*phys_BW*surfaceRatio_ILL2	15.24
340	CLINT_influx_ILL3_1 = CL_inf_api_1*phys_Normalized_ESA*phys_BW*surfaceRatio_ILL3	15
341	CLINT_influx_ILL4_1 = CL_inf_api_1*phys_Normalized_ESA*phys_BW*surf	14.4

	Initial Assignments	Initial Value
	aceRatio_ILL4	
342	HPeff_est_1 = diff_api_1	1.6667e-05
343	DIFF_duo_1 = HPeff_est_1*phys_Normalized_ESA*phys_BW*surfaceRatio_DUO	13.8
344	DIFF_jej1_1 = HPeff_est_1*phys_Normalized_ESA*phys_BW*surfaceRatio_JEJ1	27.24
345	DIFF_jej2_1 = HPeff_est_1*phys_Normalized_ESA*phys_BW*surfaceRatio_JEJ2	19.08
346	DIFF_ill1_1 = HPeff_est_1*phys_Normalized_ESA*phys_BW*surfaceRatio_ILL1	15.24
347	DIFF_ill2_1 = HPeff_est_1*phys_Normalized_ESA*phys_BW*surfaceRatio_ILL2	15.24
348	DIFF_ill3_1 = HPeff_est_1*phys_Normalized_ESA*phys_BW*surfaceRatio_ILL3	15
349	DIFF_ill4_1 = HPeff_est_1*phys_Normalized_ESA*phys_BW*surfaceRatio_ILL4	14.4
350	CLINT_efflux_DUO_1 = CL_eff_1*phys_Normalized_ESA*phys_BW*surfaceRatio_DUO	13.8
351	CLINT_efflux_JEJ1_1 = CL_eff_1*phys_Normalized_ESA*phys_BW*surfaceRatio_JEJ1	27.24
352	CLINT_efflux_JEJ2_1 = CL_eff_1*phys_Normalized_ESA*phys_BW*surfaceRatio_JEJ2	19.08
353	CLINT_efflux_ILL1_1 = CL_eff_1*phys_Normalized_ESA*phys_BW*surfaceRatio_ILL1	15.24
354	CLINT_efflux_ILL2_1 = CL_eff_1*phys_Normalized_ESA*phys_BW*surfaceRatio_ILL2	15.24
355	CLINT_efflux_ILL3_1 = CL_eff_1*phys_Normalized_ESA*phys_BW*surfaceRatio_ILL3	15
356	CLINT_efflux_ILL4_1 = CL_eff_1*phys_Normalized_ESA*phys_BW*surfaceRatio_ILL4	14.4
357	SOLIF_STOMACH_1 = SOLINT_1*HHSTOMACH_1	1.3162
358	SOLIF_DUO_1 = SOLINT_1*HHDUO_1	1
359	SOLIF_JEJ1_1 = SOLINT_1*HHJEJ1_1	1
360	SOLIF_JEJ2_1 = SOLINT_1*HHJEJ2_1	1

	Initial Assignments	Initial Value
361	SOLIF_ILL1_1 = SOLINT_1*HHILL1_1	1
362	SOLIF_ILL2_1 = SOLINT_1*HHILL2_1	1
363	SOLIF_ILL3_1 = SOLINT_1*HHILL3_1	1
364	SOLIF_ILL4_1 = SOLINT_1*HHILL4_1	1
365	SOLINT_1 = SOLFASSIF_1/HHINT_1	1
366	HHINT_1 = calculateHH(drug_inputFlag_1,REFPHSOL_1,drug_pKABase1_1,drug_pKABase2_1,drug_pKAAcid1_1, drug_pKAAcid2_1)	1
367	HHSTOMACH_1 = calculateHH(drug_inputFlag_1,pHStomach,drug_pKABase1_1,drug_pKABase2_1,drug_pKAAcid1_1,drug_pKAAcid2_1)	1.3162
368	HHDUO_1 = calculateHH(drug_inputFlag_1, pHDuo, drug_pKABase1_1, drug_pKABase2_1, drug_pKAAcid1_1, drug_pKAAcid2_1)	1
369	HHJEJ1_1 = calculateHH(drug_inputFlag_1, pHJej1, drug_pKABase1_1, drug_pKABase2_1, drug_pKAAcid1_1, drug_pKAAcid2_1)	1
370	HHJEJ2_1 = calculateHH(drug_inputFlag_1, pHJej2, drug_pKABase1_1, drug_pKABase2_1, drug_pKAAcid1_1, drug_pKAAcid2_1)	1
371	HHILL1_1 = calculateHH(drug_inputFlag_1, pHIll1, drug_pKABase1_1, drug_pKABase2_1, drug_pKAAcid1_1, drug_pKAAcid2_1)	1
372	HHILL2_1 = calculateHH(drug_inputFlag_1, pHIll2, drug_pKABase1_1, drug_pKABase2_1, drug_pKAAcid1_1, drug_pKAAcid2_1)	1
373	HHILL3_1 = calculateHH(drug_inputFlag_1, pHIll3, drug_pKABase1_1, drug_pKABase2_1, drug_pKAAcid1_1, drug_pKAAcid2_1)	1
374	HHILL4_1 = calculateHH(drug_inputFlag_1, pHIll4, drug_pKABase1_1, drug_pKABase2_1, drug_pKAAcid1_1, drug_pKAAcid2_1)	1
375	KD_1 = 3*DIFFCOEFF_1/(PDENSITY_1*PSIZE_1*DLT_1)	0.0002
376	NI_DUO_1 = 1/HHDUO_1	1
377	NI_JEJ1_1 = 1/HHJEJ1_1	1
378	NI_JEJ2_1 = 1/HHJEJ2_1	1
379	NI_ILL1_1 = 1/HHILL1_1	1
380	NI_ILL2_1 = 1/HHILL2_1	1
381	NI_ILL3_1 = 1/HHILL3_1	1
382	NI_ILL4_1 = 1/HHILL4_1	1
383	Serosa_1 = VGut*Gut_EC_fraction*(1-drug_fQ)	0.14284
384	STOMACH_1 = StomachLumenTotal/BW_average*phys_BW	0.05

	Initial Assignments	Initial Value
385	VDUO_1 = V_LUM_TOT*lumenvolumeRatio_DUO	0.03435
386	VJEJ1_1 = V_LUM_TOT*lumenvolumeRatio_JEJ1	0.0211
387	VJEJ2_1 = V_LUM_TOT*lumenvolumeRatio_JEJ2	0.0211
388	VILL1_1 = V_LUM_TOT*lumenvolumeRatio_ILL1	0.0126
389	VILL2_1 = V_LUM_TOT*lumenvolumeRatio_ILL2	0.0126
390	VILL3_1 = V_LUM_TOT*lumenvolumeRatio_ILL3	0.0126
391	VILL4_1 = V_LUM_TOT*lumenvolumeRatio_ILL4	0.0126
392	MDUO_1 = VGut*volumeRatio_DUO*Gut_IC_fraction	0.037454
393	MJEJ1_1 = VGut*volumeRatio_JEJ1*Gut_IC_fraction	0.073785
394	MJEJ2_1 = VGut*volumeRatio_JEJ2*Gut_IC_fraction	0.051687
395	MILL1_1 = VGut*volumeRatio_ILL1*Gut_IC_fraction	0.0412
396	MILL2_1 = VGut*volumeRatio_ILL2*Gut_IC_fraction	0.0412
397	MILL3_1 = VGut*volumeRatio_ILL3*Gut_IC_fraction	0.04045
398	MILL4_1 = VGut*volumeRatio_ILL4*Gut_IC_fraction	0.038952
399	VillousDUO_1 = VGut*volumeRatio_DUO*Gut_EC_fraction*drug_fQ	0.0057016
400	VillousJEJ1_1 = VGut*volumeRatio_JEJ1*Gut_EC_fraction*drug_fQ	0.011232
401	VillousJEJ2_1 = VGut*volumeRatio_JEJ2*Gut_EC_fraction*drug_fQ	0.0078682
402	VillousILL1_1 = VGut*volumeRatio_ILL1*Gut_EC_fraction*drug_fQ	0.0062718
403	VillousILL2_1 = VGut*volumeRatio_ILL2*Gut_EC_fraction*drug_fQ	0.0062718
404	VillousILL3_1 = VGut*volumeRatio_ILL3*Gut_EC_fraction*drug_fQ	0.0061578
405	VillousILL4_1 = VGut*volumeRatio_ILL4*Gut_EC_fraction*drug_fQ	0.0059297
406	Liver_1 = phys_BW*(phys_Normalized_weight_liver_blood + phys_Normalized_weight_liver_tissue)*Specific_volume	1.603
407	Kp_liver_1 = drug_Kp_liver_raw_1*switch_SFKp_1	0.51837
408	k_liver_metabolites_1 = (switch_SFmet_1*((drug_HLM_CLint_1/drug_fumic_1*phys_MPGL)+(drug_CLmetg_1/drug_funic_1*phys_HPGL))*(phys_BW*phys_Normalized_weight_liver_tissue*Specific_volume)/Specific_volume)*drug_fuLiver_1	1.0507
409	k_Liver_Venous_1 = Q_li_1/Kp_liver_1*drug_BRP_1	105.3302
410	k_liver_bile_1 = drug_PSbileg_1*switch_SFbile_1*phys_HPGL*	0.098292

	Initial Assignments	Initial Value
	(phys_BW*phys_Normalized_weight_liver_tissue*Specific_volume)/Specific_volume*drug_fuLiver_1	
411	LOGSR_1 = 0.75*LOGP_1+2.27	3.02
412	CLINT_efflux_baso_DUO = CL_eff_baso*phys_Normalized_ESA_baso*phys_BW*basoSurfaceRatio_DUO	0
413	CLINT_efflux_baso_ILL1 = CL_eff_baso*phys_Normalized_ESA_baso*phys_BW*basoSurfaceRatio_ILL1	0
414	CLINT_efflux_baso_ILL2 = CL_eff_baso*phys_Normalized_ESA_baso*phys_BW*basoSurfaceRatio_ILL2	0
415	CLINT_efflux_baso_ILL3 = CL_eff_baso*phys_Normalized_ESA_baso*phys_BW*basoSurfaceRatio_ILL3	0
416	CLINT_efflux_baso_ILL4 = CL_eff_baso*phys_Normalized_ESA_baso*phys_BW*basoSurfaceRatio_ILL4	0
417	CLINT_efflux_baso_JEJ1 = CL_eff_baso*phys_Normalized_ESA_baso*phys_BW*basoSurfaceRatio_JEJ1	0
418	CLINT_efflux_baso_JEJ2 = CL_eff_baso*phys_Normalized_ESA_baso*phys_BW*basoSurfaceRatio_JEJ2	0
419	CLINT_efflux_baso_DUO_1 = CL_eff_baso_1*phys_Normalized_ESA_baso*phys_BW*basoSurfaceRatio_DUO	0
420	CLINT_efflux_baso_JEJ1_1 = CL_eff_baso_1*phys_Normalized_ESA_baso*phys_BW*basoSurfaceRatio_JEJ1	0
421	CLINT_efflux_baso_JEJ2_1 = CL_eff_baso_1*phys_Normalized_ESA_baso*phys_BW*basoSurfaceRatio_JEJ2	0
422	CLINT_efflux_baso_ILL1_1 = CL_eff_baso_1*phys_Normalized_ESA_baso*phys_BW*basoSurfaceRatio_ILL1	0
423	CLINT_efflux_baso_ILL2_1 = CL_eff_baso_1*phys_Normalized_ESA_baso*phys_BW*basoSurfaceRatio_ILL2	0
424	CLINT_efflux_baso_ILL3_1 = CL_eff_baso_1*phys_Normalized_ESA_baso*phys_BW*basoSurfaceRatio_ILL3	0
425	CLINT_efflux_baso_ILL4_1 = CL_eff_baso_1*phys_Normalized_ESA_baso*phys_BW*basoSurfaceRatio_ILL4	0
426	diff_api_1 = diff_baso_1	1

Repeated Assignments

	Repeated Assignments	Initial Value
1	k_Liver_IC_S5_Metabolites = (switch_Vmax_met*phys_BW/(drug_Km_met+drug_fuLiver*Liver_IC_S5.Liver_IC_S5_drug/drug_molar_mass)+switch_SFmet*((drug_HLM_CLint/drug_fumic*phys_MPGL)+(drug_CLmetg/drug_funic*phys_HPGL))*(phys_BW*phys_Normalized_weight_liver_tissue*Specific_volume)/Specific_volume)/5*drug_fuLiver	0.7973
2	k_Liver_IC_S4_Metabolites = (switch_Vmax_met*phys_BW/(drug_Km_met+drug_fuLiver*Liver_IC_S4.Liver_IC_S4_drug/drug_molar_mass)+switch_SFmet*((drug_HLM_CLint/drug_fumic*phys_MPGL)+(drug_CLmetg/drug_funic*phys_HPGL))*(phys_BW*phys_Normalized_weight_liver_tissue*Specific_volume)/Specific_volume)/5*drug_fuLiver	0.7973
3	k_Liver_IC_S3_Metabolites = (switch_Vmax_met*phys_BW/(drug_Km_met+drug_fuLiver*Liver_IC_S3.Liver_IC_S3_drug/drug_molar_mass)+switch_SFmet*((drug_HLM_CLint/drug_fumic*phys_MPGL)+(drug_CLmetg/drug_funic*phys_HPGL))*(phys_BW*phys_Normalized_weight_liver_tissue*Specific_volume)/Specific_volume)/5*drug_fuLiver	0.7973
4	k_Liver_IC_S2_Metabolites = (switch_Vmax_met*phys_BW/(drug_Km_met+drug_fuLiver*Liver_IC_S2.Liver_IC_S2_drug/drug_molar_mass)+switch_SFmet*((drug_HLM_CLint/drug_fumic*phys_MPGL)+(drug_CLmetg/drug_funic*phys_HPGL))*(phys_BW*phys_Normalized_weight_liver_tissue*Specific_volume)/Specific_volume)/5*drug_fuLiver	0.7973
5	k_Liver_IC_S1_Metabolites = (switch_Vmax_met*phys_BW/(drug_Km_met+drug_fuLiver*Liver_IC_S1.Liver_IC_S1_drug/drug_molar_mass)+switch_SFmet*((drug_HLM_CLint/drug_fumic*phys_MPGL)+(drug_CLmetg/drug_funic*phys_HPGL))*(phys_BW*phys_Normalized_weight_liver_tissue*Specific_volume)/Specific_volume)/5*drug_fuLiver	0.7973
6	k_Liver_EC_S5_Liver_IC_S5 = (drug_PSdifg*switch_SFdiff*phys_HPGL*(phys_BW*phys_Normalized_weight_liver_tissue*Specific_volume)/Specific_volume+switch_Vmax_uptake*phys_BW/(drug_Km_uptake+drug_fB*Liver_EC_S5.Liver_EC_S5_drug/drug_molar_mass)+drug_PSinfg*switch_SFinf*phys_HPGL*(phys_BW*phys_Normalized_weight_liver_tissue*Specific_volume)/Specific_volume)/5*drug_fB	513.8127
7	k_Liver_EC_S4_Liver_IC_S4 = (drug_PSdifg*switch_SFdiff*phys_HPGL*(phys_BW*phys_Normalized_weight_liver_tissue*Specific_volume)/Specific_volume+switch_Vmax_uptake*phys_BW/(drug_Km_uptake+drug_fB*Liver_EC_S4.Liver_EC_S4_drug/drug_molar_mass)+drug_PSinf	513.8127

	Repeated Assignments	Initial Value
	g*switch_SFinf*phys_HPGL*(phys_BW*phys_Normalized_weight_liver_tissue*Specific_volume)/Specific_volume)/5*drug_fB	
8	k_Liver_EC_S3_Liver_IC_S3 = (drug_PSdifg*switch_SFdiff*phys_HPGL*(phys_BW*phys_Normalized_weight_liver_tissue*Specific_volume)/Specific_volume+switch_Vmax_uptake*phys_BW/(drug_Km_uptake+drug_fB*Liver_EC_S3.Liver_EC_S3_drug/drug_molar_mass)+drug_PSinfg*switch_SFinf*phys_HPGL*(phys_BW*phys_Normalized_weight_liver_tissue*Specific_volume)/Specific_volume)/5*drug_fB	513.8127
9	k_Liver_EC_S2_Liver_IC_S2 = (drug_PSdifg*switch_SFdiff*phys_HPGL*(phys_BW*phys_Normalized_weight_liver_tissue*Specific_volume)/Specific_volume+switch_Vmax_uptake*phys_BW/(drug_Km_uptake+drug_fB*Liver_EC_S2.Liver_EC_S2_drug/drug_molar_mass)+drug_PSinfg*switch_SFinf*phys_HPGL*(phys_BW*phys_Normalized_weight_liver_tissue*Specific_volume)/Specific_volume)/5*drug_fB	513.8127
10	k_Liver_EC_S1_Liver_IC_S1 = (drug_PSdifg*switch_SFdiff*phys_HPGL*(phys_BW*phys_Normalized_weight_liver_tissue*Specific_volume)/Specific_volume+switch_Vmax_uptake*phys_BW/(drug_Km_uptake+drug_fB*Liver_EC_S1.Liver_EC_S1_drug/drug_molar_mass)+drug_PSinfg*switch_SFinf*phys_HPGL*(phys_BW*phys_Normalized_weight_liver_tissue*Specific_volume)/Specific_volume)/5*drug_fB	513.8127
11	Blood_total.Blood_total_drug = (Artery.Artery_drug*Artery+Venous.Venous_drug*Venous)/(Artery+Venous)	0
12	Plasma_total.Plasma_total_drug = Blood_total.Blood_total_drug/drug_BRP	0
13	Portal.Portal_drug = (Q_gut_liver/numIntestinalCompartments*(VillousDUO.Villous_DUO/VillousDUO+VillousJEJ1.Villous_JEJ1/VillousJEJ1+VillousJEJ2.Villous_JEJ2/VillousJEJ2+VillousILL1.Villous_ILL1/VillousILL1+VillousILL2.Villous_ILL2/VillousILL2+VillousILL3.Villous_ILL3/VillousILL3+VillousILL4.Villous_ILL4/VillousILL4)+Q_spleen_liver*(Spleen.Spleen_drug/Kp_spleen*drug_BRP))/(Q_gut_liver+Q_spleen_liver)	0
14	Portal_1.Portal_plasma_drug = Portal.Portal_drug/drug_BRP	0
15	Mass_Balance.Amount_body = Venous.Venous_drug*Venous+Artery.Artery_drug*Artery+(1-switch_liverFlag)*(Liver_IC_S1.Liver_IC_S1_drug*Liver_IC_S1+Liver_IC_S2.Liver_IC_S2_drug*Liver_IC_S2+Liver_IC_S3.Liver_IC_S3_drug*Liver_IC_S3+Liver_IC_S4.Liver_IC_S4_drug*Liver_IC_S4+Liver_IC_S5.Liver_IC_S5_drug*Liver_IC_S5+Liver_EC_S1.Liver_EC_S1_drug*	0

	Repeated Assignments	Initial Value
	Liver_EC_S1+Liver_EC_S2.Liver_EC_S2_drug*Liver_EC_S2+Liver_EC_S3.Liver_EC_S3_drug*Liver_EC_S3+Liver_EC_S4.Liver_EC_S4_drug*Liver_EC_S4+Liver_EC_S5.Liver_EC_S5_drug*Liver_EC_S5)+switch_liverFlag*Liver.Liver_drug*Liver+Lung.Lung_drug*Lung+Adipose.Adipose_drug*Adipose+Heart.Heart_drug*Heart+Muscle.Muscle_drug*Muscle+Skin.Skin_drug*Skin+Kidney.Kidney_drug*Kidney+Bone.Bone_drug*Bone+Testes.Testes_drug*Testes+Rest.Rest_drug*Rest+Gut.Gut_drug*Gut+MDUO.MEM_DUO*milligram_per_microgram+MJEJ1.MEM_JEJ1*milligram_per_microgram+MJEJ2.MEM_JEJ2*milligram_per_microgram+MILL1.MEM_ILL1*milligram_per_microgram+MILL2.MEM_ILL2*milligram_per_microgram+MILL3.MEM_ILL3*milligram_per_microgram+MILL4.MEM_ILL4*milligram_per_microgram+Spleen.Spleen_drug*Spleen+Brain.Brain_drug*Brain+VillousDUO.Villous_DUO+VillousJEJ1.Villous_JEJ1+VillousJEJ2.Villous_JEJ2+VillousILL1.Villous_ILL1+VillousILL2.Villous_ILL2+VillousILL3.Villous_ILL3+VillousILL4.Villous_ILL4+Serosa*Serosa.Serosa_drug	
16	Mass_Balance.Amount_total = Venous.Venous_drug*Venous+Artery.Artery_drug*Artery+(1-switch_liverFlag)*(Liver_IC_S1.Liver_IC_S1_drug*Liver_IC_S1+Liver_IC_S2.Liver_IC_S2_drug*Liver_IC_S2+Liver_IC_S3.Liver_IC_S3_drug*Liver_IC_S3+Liver_IC_S4.Liver_IC_S4_drug*Liver_IC_S4+Liver_IC_S5.Liver_IC_S5_drug*Liver_IC_S5+Liver_EC_S1.Liver_EC_S1_drug*Liver_EC_S1+Liver_EC_S2.Liver_EC_S2_drug*Liver_EC_S2+Liver_EC_S3.Liver_EC_S3_drug*Liver_EC_S3+Liver_EC_S4.Liver_EC_S4_drug*Liver_EC_S4+Liver_EC_S5.Liver_EC_S5_drug*Liver_EC_S5)+switch_liverFlag*Liver.Liver_drug*Liver+Lung.Lung_drug*Lung+Adipose.Adipose_drug*Adipose+Heart.Heart_drug*Heart+Muscle.Muscle_drug*Muscle+Skin.Skin_drug*Skin+Kidney.Kidney_drug*Kidney+Bone.Bone_drug*Bone+Testes.Testes_drug*Testes+Rest.Rest_drug*Rest+(MDUO.MEM_DUO+MJEJ1.MEM_JEJ1+MJEJ2.MEM_JEJ2+MILL1.MEM_ILL1+MILL2.MEM_ILL2+MILL3.MEM_ILL3+MILL4.MEM_ILL4)*milligram_per_microgram+Spleen.Spleen_drug*Spleen+Brain.Brain_drug*Brain+Main_compartment.Bile_drug+Urine.Urine_drug+Gut_Lumen.Gut_Lumen_drug+(STOMACH.X_STOMACH_DISS+VDUO.X_DUO_DISS+VJEJ1.X_JEJ1_DISS+VJEJ2.X_JEJ2_DISS+VILL1.X_ILL1_DISS+VILL2.X_ILL2_DISS+VILL3.X_ILL3_DISS+VILL4.X_ILL4_DISS+Colon.X_CECUM_DISS)*milligram_per_microgram+(STOMACH.X_STOMACH_SOLID+VDUO.X_DUO_SOLID+VJEJ1.X_JEJ1_SOLID+VJEJ2.X_JEJ2_SOLID+VILL1.X_ILL1_SOLID+VILL2.X_ILL2_SOLID+VILL3.X_ILL3_SOLID+VILL4.X_ILL4_SOLID+Colon.X_CECUM_SOLID)*milligram_per_microgram+VillousDUO.Villous_DUO+VillousJEJ1.Villous_JEJ1+VillousJEJ2.Villous_JEJ2+VillousILL1.Villous_ILL1+VillousIL	0

	Repeated Assignments	Initial Value
	L2.Villous_ILL2+VillousILL3.Villous_ILL3+VillousILL4.Villous_ILL4+Serosa.Serosa_drug*Serosa	
17	Plasma_total.Plasma_free_uM = Plasma_total.Plasma_total_drug*drug_fuplasma/drug_molar_mass	0
18	Liver_total.Liver_blood_total = (1-switch_liverFlag)*((Liver_EC_S1.Liver_EC_S1_drug+Liver_EC_S2.Liver_EC_S2_drug+Liver_EC_S3.Liver_EC_S3_drug+Liver_EC_S4.Liver_EC_S4_drug+Liver_EC_S5.Liver_EC_S5_drug)/5)	0
19	Liver_total.Liver_tissue_total = (1-switch_liverFlag)*((Liver_IC_S1.Liver_IC_S1_drug+Liver_IC_S2.Liver_IC_S2_drug+Liver_IC_S3.Liver_IC_S3_drug+Liver_IC_S4.Liver_IC_S4_drug+Liver_IC_S5.Liver_IC_S5_drug)/5)	0
20	Liver_total.Liver_blood_free = Liver_total.Liver_blood_total*drug_fuplasma/drug_BRP	0
21	Liver_total.Liver_tissue_free = Liver_total.Liver_tissue_total*drug_fuLiver	0
22	convert_to_nmole_per_kg.Adipose_nmole = Adipose.Adipose_drug*Adipose/drug_molar_mass*nanomole_per_mole*kilogram/phys_BW	0
23	convert_to_nmole_per_kg.Artery_nmole = Artery.Artery_drug*Artery/drug_molar_mass*nanomole_per_mole*kilogram/phys_BW	0
24	convert_to_nmole_per_kg.Bone_nmole = Bone.Bone_drug*Bone/drug_molar_mass*nanomole_per_mole*kilogram/phys_BW	0
25	convert_to_nmole_per_kg.Liver_EC1_nmole = Liver_EC_S1.Liver_EC_S1_drug*Liver_EC_S1/drug_molar_mass*nanomole_per_mole*kilogram/phys_BW	0
26	convert_to_nmole_per_kg.Liver_IC1_nmole = Liver_IC_S1.Liver_IC_S1_drug*Liver_IC_S1/drug_molar_mass*nanomole_per_mole*kilogram/phys_BW	0
27	convert_to_nmole_per_kg.Muscle_nmole = Muscle.Muscle_drug*Muscle/drug_molar_mass*nanomole_per_mole*kilogram/phys_BW	0
28	convert_to_nmole_per_kg.Urine_nmole = Urine.Urine_drug/drug_molar_mass*nanomole_per_mole*kilogram/phys_BW	0
29	convert_to_nmole_per_kg.Venous_nmole = Venous.Venous_drug*Venous/drug_molar_mass*nanomole_per_mole*kilogram/phys_BW	0
30	convert_to_nmole_per_kg.Bile_nmole = Main_compartment.Bile_drug/drug_molar_mass*nanomole_per_mole*kilogram/phys_BW	0
31	Plasma_total.Plasma_total_uM = Plasma_total.Plasma_total_drug/drug_molar_mass	0

	Repeated Assignments	Initial Value
32	Liver_total.Liver_tissue_total_uM = Liver_total.Liver_tissue_total/drug_molar_mass	0
33	convert_to_nmole_per_kg.Kidney_nmole = Kidney.Kidney_drug*Kidney/drug_molar_mass*nanomole_per_mole*kilogram/phys_BW	0
34	convert_to_nmole_per_kg.Lung_nmole = Lung.Lung_drug*Lung/drug_molar_mass*nanomole_per_mole*kilogram/phys_BW	0
35	convert_to_nmole_per_kg.Metabolites_nmole = Metabolites.Metabolites_drug/drug_molar_mass*nanomole_per_mole*kilogram/phys_BW	0
36	convert_to_nmole_per_kg.Liver_IC2_nmole = Liver_IC_S2.Liver_IC_S2_drug*Liver_IC_S2/drug_molar_mass*nanomole_per_mole*kilogram/phys_BW	0
37	convert_to_nmole_per_kg.Liver_EC2_nmole = Liver_EC_S2.Liver_EC_S2_drug*Liver_EC_S2/drug_molar_mass*nanomole_per_mole*kilogram/phys_BW	0
38	convert_to_nmole_per_kg.Liver_EC3_nmole = Liver_EC_S3.Liver_EC_S3_drug*Liver_EC_S3/drug_molar_mass*nanomole_per_mole*kilogram/phys_BW	0
39	convert_to_nmole_per_kg.Liver_IC3_nmole = Liver_IC_S3.Liver_IC_S3_drug*Liver_IC_S3/drug_molar_mass*nanomole_per_mole*kilogram/phys_BW	0
40	convert_to_nmole_per_kg.Liver_IC4_nmole = Liver_IC_S4.Liver_IC_S4_drug*Liver_IC_S4/drug_molar_mass*nanomole_per_mole*kilogram/phys_BW	0
41	convert_to_nmole_per_kg.Liver_EC4_nmole = Liver_EC_S4.Liver_EC_S4_drug*Liver_EC_S4/drug_molar_mass*nanomole_per_mole*kilogram/phys_BW	0
42	convert_to_nmole_per_kg.Liver_EC5_nmole = Liver_EC_S5.Liver_EC_S5_drug*Liver_EC_S5/drug_molar_mass*nanomole_per_mole*kilogram/phys_BW	0
43	convert_to_nmole_per_kg.Liver_IC5_nmole = Liver_IC_S5.Liver_IC_S5_drug*Liver_IC_S5/drug_molar_mass*nanomole_per_mole*kilogram/phys_BW	0
44	convert_to_nmole_per_kg.Gut_nmole = Gut.Gut_drug*Gut/drug_molar_mass*nanomole_per_mole*kilogram/phys_BW	0
45	convert_to_nmole_per_kg.Spleen_nmole = Spleen.Spleen_drug*Spleen/drug_molar_mass*nanomole_per_mole*kilogram/phys_BW	0
46	convert_to_nmole_per_kg.Skin_nmole = Skin.Skin_drug*Skin/drug_molar_mass*nanomole_per_mole*kilogram/phys_BW	0
47	convert_to_nmole_per_kg.Brain_nmole = Brain.Brain_drug*Brain/drug_molar_mass*nanomole	0

	Repeated Assignments	Initial Value
	_per_mole*kilogram/phys_BW	
48	convert_to_nmole_per_kg.Rest_nmole = Rest.Rest_drug*Rest/drug_molar_mass*nanomole_per_mole*kilogram/phys_BW	0
49	convert_to_nmole_per_kg.Heart_nmole = Heart.Heart_drug*Heart/drug_molar_mass*nanomole_per_mole*kilogram/phys_BW	0
50	k_Liver_IC_S5_Metabolites_1 = met_inhib_S5*(switch_Vmax_met_1*phys_BW/(drug_Km_met_1+drug_fuLiver_1*Liver_IC_S5_1.Liver_IC_S5_drug_1/drug_molar_mass_1)+switch_SFmet_1*((drug_HLM_CLint_1/drug_fumic_1*phys_MPGL)+(drug_CLmetg_1/drug_funic_1*phys_HPGL))*(phys_BW*phys_Normalized_weight_liver_tissue*Specific_volume)/Specific_volume)/5*drug_fuLiver_1	0.21013
51	k_Liver_IC_S4_Metabolites_1 = met_inhib_S4*(switch_Vmax_met_1*phys_BW/(drug_Km_met_1+drug_fuLiver_1*Liver_IC_S4_1.Liver_IC_S4_drug_1/drug_molar_mass_1)+switch_SFmet_1*((drug_HLM_CLint_1/drug_fumic_1*phys_MPGL)+(drug_CLmetg_1/drug_funic_1*phys_HPGL))*(phys_BW*phys_Normalized_weight_liver_tissue*Specific_volume)/Specific_volume)/5*drug_fuLiver_1	0.21013
52	k_Liver_IC_S3_Metabolites_1 = met_inhib_S3*(switch_Vmax_met_1*phys_BW/(drug_Km_met_1+drug_fuLiver_1*Liver_IC_S3_1.Liver_IC_S3_drug_1/drug_molar_mass_1)+switch_SFmet_1*((drug_HLM_CLint_1/drug_fumic_1*phys_MPGL)+(drug_CLmetg_1/drug_funic_1*phys_HPGL))*(phys_BW*phys_Normalized_weight_liver_tissue*Specific_volume)/Specific_volume)/5*drug_fuLiver_1	0.21013
53	k_Liver_IC_S2_Metabolites_1 = met_inhib_S2*(switch_Vmax_met_1*phys_BW/(drug_Km_met_1+drug_fuLiver_1*Liver_IC_S2_1.Liver_IC_S2_drug_1/drug_molar_mass_1)+switch_SFmet_1*((drug_HLM_CLint_1/drug_fumic_1*phys_MPGL)+(drug_CLmetg_1/drug_funic_1*phys_HPGL))*(phys_BW*phys_Normalized_weight_liver_tissue*Specific_volume)/Specific_volume)/5*drug_fuLiver_1	0.21013
54	k_Liver_IC_S1_Metabolites_1 = met_inhib_S1*(switch_Vmax_met_1*phys_BW/(drug_Km_met_1+drug_fuLiver_1*Liver_IC_S1_1.Liver_IC_S1_drug_1/drug_molar_mass_1)+switch_SFmet_1*((drug_HLM_CLint_1/drug_fumic_1*phys_MPGL)+(drug_CLmetg_1/drug_funic_1*phys_HPGL))*(phys_BW*phys_Normalized_weight_liver_tissue*Specific_volume)/Specific_volume)/5*drug_fuLiver_1	0.21013
55	k_Liver_IC_S5_Bile_1 = biliary_inhib_S5*drug_PSbileg_1*switch_SFbile_1*phys_HPGL*(phys_BW*phys_Normalized_weight_liver_tissue*Specific_volume)/Specific_volume/5*drug_fuLiver_1	0.019658
56	k_Liver_IC_S4_Bile_1 = biliary_inhib_S4*drug_PSbileg_1*switch_SFbile_1*p	0.019658

	Repeated Assignments	Initial Value
	hys_HPGL* (phys_BW*phys_Normalized_weight_liver_tissue*Specific_volume)/Specific_volume/5*drug_fuLiver_1	
57	k_Liver_IC_S3_Bile_1 = biliary_inhib_S3*drug_PSbileg_1*switch_SFbile_1*phys_HPGL* (phys_BW*phys_Normalized_weight_liver_tissue*Specific_volume)/Specific_volume/5*drug_fuLiver_1	0.019658
58	k_Liver_IC_S2_Bile_1 = biliary_inhib_S2*drug_PSbileg_1*switch_SFbile_1*phys_HPGL* (phys_BW*phys_Normalized_weight_liver_tissue*Specific_volume)/Specific_volume/5*drug_fuLiver_1	0.019658
59	k_Liver_IC_S1_Bile_1 = biliary_inhib_S1*drug_PSbileg_1*switch_SFbile_1*phys_HPGL* (phys_BW*phys_Normalized_weight_liver_tissue*Specific_volume)/Specific_volume/5*drug_fuLiver_1	0.019658
60	k_Liver_EC_S5_Liver_IC_S5_1 = (drug_PSdifg_1*switch_SFdiff_1*phys_HPGL* (phys_BW*phys_Normalized_weight_liver_tissue*Specific_volume)/Specific_volume+uptake_inhib_S5*switch_Vmax_uptake_1*phys_BW/(drug_Km_uptake_1+drug_fB_1*Liver_EC_S5_1.Liver_EC_S5_drug_1/drug_molar_mass_1)+uptake_inhib_S5*drug_PSinfg_1*switch_SFinf_1*phys_HPGL* (phys_BW*phys_Normalized_weight_liver_tissue*Specific_volume)/Specific_volume)/5*drug_fB_1	168.0698
61	k_Liver_EC_S4_Liver_IC_S4_1 = (drug_PSdifg_1*switch_SFdiff_1*phys_HPGL* (phys_BW*phys_Normalized_weight_liver_tissue*Specific_volume)/Specific_volume+uptake_inhib_S4*switch_Vmax_uptake_1*phys_BW/(drug_Km_uptake_1+drug_fB_1*Liver_EC_S4_1.Liver_EC_S4_drug_1/drug_molar_mass_1)+uptake_inhib_S4*drug_PSinfg_1*switch_SFinf_1*phys_HPGL* (phys_BW*phys_Normalized_weight_liver_tissue*Specific_volume)/Specific_volume)/5*drug_fB_1	168.0698
62	k_Liver_EC_S3_Liver_IC_S3_1 = (drug_PSdifg_1*switch_SFdiff_1*phys_HPGL* (phys_BW*phys_Normalized_weight_liver_tissue*Specific_volume)/Specific_volume+uptake_inhib_S3*switch_Vmax_uptake_1*phys_BW/(drug_Km_uptake_1+drug_fB_1*Liver_EC_S3_1.Liver_EC_S3_drug_1/drug_molar_mass_1)+uptake_inhib_S3*drug_PSinfg_1*switch_SFinf_1*phys_HPGL* (phys_BW*phys_Normalized_weight_liver_tissue*Specific_volume)/Specific_volume)/5*drug_fB_1	168.0698
63	k_Liver_EC_S2_Liver_IC_S2_1 = (drug_PSdifg_1*switch_SFdiff_1*phys_HPGL* (phys_BW*phys_Normalized_weight_liver_tissue*Specific_volume)/Specific_volume+uptake_inhib_S2*switch_Vmax_uptake_1*phys_BW/(drug_Km_uptake_1+drug_fB_1*Liver_EC_S2_1.Liver_EC_S2_drug_1/drug_molar_mass_1)+uptake_inhib_S2*drug_PSinfg_1	168.0698

	Repeated Assignments	Initial Value
	*switch_SFinf_1*phys_HPGL*(phys_BW*phys_Normalized_weight_liver_tissue*Specific_volume)/Specific_volume)/5*drug_fB_1	
64	k_Liver_EC_S1_Liver_IC_S1_1 = (drug_PSdifg_1*switch_SFdiff_1*phys_HPGL*(phys_BW*phys_Normalized_weight_liver_tissue*Specific_volume)/Specific_volume+uptake_inhib_S1*switch_Vmax_uptake_1*phys_BW/(drug_Km_uptake_1+drug_fB_1*Liver_EC_S1_1.Liver_EC_S1_drug_1/drug_molar_mass_1)+uptake_inhib_S1*drug_PSinfg_1*switch_SFinf_1*phys_HPGL*(phys_BW*phys_Normalized_weight_liver_tissue*Specific_volume)/Specific_volume)/5*drug_fB_1	168.0698
65	Blood_total_1.Blood_total_drug_1 = (Artery_1.Artery_drug_1*Artery_1+Venous_1.Venous_drug_1*Venous_1)/(Artery_1+Venous_1)	0
66	Plasma_total_1.Plasma_total_drug_1 = Blood_total_1.Blood_total_drug_1/drug_BRP_1	0
67	Portal_1.Portal_drug_1 = (Q_gut_liver_1*(Gut_1.Gut_drug_1/Kp_gut_1*drug_BRP_1)+Q_spleen_liver_1*(Spleen_1.Spleen_drug_1/Kp_spleen_1*drug_BRP_1))/(Q_gut_liver_1+Q_spleen_liver_1)	0
68	Portal_1.Portal_plasma_drug_1 = Portal_1.Portal_drug_1/drug_BRP_1	0
69	Mass_Balance_1.Amount_body_1 = Venous_1.Venous_drug_1*Venous_1+Artery_1.Artery_drug_1*Artery_1+Liver_IC_S1_1.Liver_IC_S1_drug_1*Liver_IC_S1_1+Liver_IC_S2_1.Liver_IC_S2_drug_1*Liver_IC_S2_1+Liver_IC_S3_1.Liver_IC_S3_drug_1*Liver_IC_S3_1+Liver_IC_S4_1.Liver_IC_S4_drug_1*Liver_IC_S4_1+Liver_IC_S5_1.Liver_IC_S5_drug_1*Liver_IC_S5_1+Liver_EC_S1_1.Liver_EC_S1_drug_1*Liver_EC_S1_1+Liver_EC_S2_1.Liver_EC_S2_drug_1*Liver_EC_S2_1+Liver_EC_S3_1.Liver_EC_S3_drug_1*Liver_EC_S3_1+Liver_EC_S4_1.Liver_EC_S4_drug_1*Liver_EC_S4_1+Liver_EC_S5_1.Liver_EC_S5_drug_1*Liver_EC_S5_1+Lung_1.Lung_drug_1*Lung_1+Adipose_1.Adipose_drug_1*Adipose_1+Heart_1.Heart_drug_1*Heart_1+Muscle_1.Muscle_drug_1*Muscle_1+Skin_1.Skin_drug_1*Skin_1+Kidney_1.Kidney_drug_1*Kidney_1+Bone_1.Bone_drug_1*Bone_1+Testes_1.Testes_drug_1*Testes_1+Rest_1.Rest_drug_1*Rest_1+Gut_1.Gut_drug_1*Gut_1+Spleen_1.Spleen_drug_1*Spleen_1+Brain_1.Brain_drug_1*Brain_1	0
70	Mass_Balance_1.Amount_total_1 = Venous_1.Venous_drug_1*Venous_1+Artery_1.Artery_drug_1*Artery_1+Liver_IC_S1_1.Liver_IC_S1_drug_1*Liver_IC_S1_1+Liver_IC_S2_1.Liver_IC_S2_drug_1*Liver_IC_S2_1+Liver_IC_S3_1.Liver_IC_S3_drug_1*Liver_IC_S3_1+Liver_IC_S4_1.Liver_IC_S4_drug_1*Liver_IC_S4_1+Liver_IC_S5_1.Liver_IC_S5_drug_1*Liver_IC_S5_1+Liver_EC_S1_1.Liver_EC_S1_drug_1*Liver_EC_S1_1+Liver_EC_S2_1.Liver_E	0

	Repeated Assignments	Initial Value
	C_S2_drug_1*Liver_EC_S2_1+Liver_EC_S3_1.Liver_EC_S3_drug_1*Liver_EC_S3_1+Liver_EC_S4_1.Liver_EC_S4_drug_1*Liver_EC_S4_1+Liver_EC_S5_1.Liver_EC_S5_drug_1*Liver_EC_S5_1+Lung_1.Lung_drug_1*Lung_1+Adipose_1.Adipose_drug_1*Adipose_1+Heart_1.Heart_drug_1*Heart_1+Muscle_1.Muscle_drug_1*Muscle_1+Skin_1.Skin_drug_1*Skin_1+Kidney_1.Kidney_drug_1*Kidney_1+Bone_1.Bone_drug_1*Bone_1+Testes_1.Testes_drug_1*Testes_1+Rest_1.Rest_drug_1*Rest_1+Gut_1.Gut_drug_1*Gut_1+Spleen_1.Spleen_drug_1*Spleen_1+Brain_1.Brain_drug_1*Brain_1+Main_compartment_1.Bile_drug_1+Metabolites_1.Metabolites_drug_1+Urine_1.Urine_drug_1+Gut_Lumen_1.Gut_Lumen_drug_1	
71	Plasma_total_1.Plasma_free_uM_1 = Plasma_total_1.Plasma_total_drug_1*drug_fuplasma_1/drug_molar_mass_1	0
72	Liver_total_1.Liver_blood_total_1 = (Liver_EC_S1_1.Liver_EC_S1_drug_1+Liver_EC_S2_1.Liver_EC_S2_drug_1+Liver_EC_S3_1.Liver_EC_S3_drug_1+Liver_EC_S4_1.Liver_EC_S4_drug_1+Liver_EC_S5_1.Liver_EC_S5_drug_1)/5	0
73	Liver_total_1.Liver_tissue_total_1 = (Liver_IC_S1_1.Liver_IC_S1_drug_1+Liver_IC_S2_1.Liver_IC_S2_drug_1+Liver_IC_S3_1.Liver_IC_S3_drug_1+Liver_IC_S4_1.Liver_IC_S4_drug_1+Liver_IC_S5_1.Liver_IC_S5_drug_1)/5	0
74	Liver_total_1.Liver_blood_free_1 = Liver_total_1.Liver_blood_total_1*drug_fuplasma_1/drug_BRP_1	0
75	Liver_total_1.Liver_tissue_free_uM_1 = Liver_total_1.Liver_tissue_total_1*drug_fuLiver_1/drug_molar_mass_1	0
76	convert_to_nmole_per_kg_1.Adipose_nmole_1 = Adipose_1.Adipose_drug_1*Adipose_1/drug_molar_mass_1*nanomole_per_mole*kilogram/phys_BW	0
77	convert_to_nmole_per_kg_1.Artery_nmole_1 = Artery_1.Artery_drug_1*Artery_1/drug_molar_mass_1*nanomole_per_mole*kilogram/phys_BW	0
78	convert_to_nmole_per_kg_1.Bone_nmole_1 = Bone_1.Bone_drug_1*Bone_1/drug_molar_mass_1*nanomole_per_mole*kilogram/phys_BW	0
79	convert_to_nmole_per_kg_1.Liver_EC1_nmole_1 = Liver_EC_S1_1.Liver_EC_S1_drug_1*Liver_EC_S1_1/drug_molar_mass_1*nanomole_per_mole*kilogram/phys_BW	0
80	convert_to_nmole_per_kg_1.Liver_IC1_nmole_1 = Liver_IC_S1_1.Liver_IC_S1_drug_1*Liver_IC_S1_1/drug_molar_mass_1*nanomole_per_mole*kilogram/phys_BW	0
81	convert_to_nmole_per_kg_1.Muscle_nmole_1 = Muscle_1.Muscle_drug_1*Muscle_1/drug_molar_mass_1*nanomole_per_mole*kilogram/phys_BW	0

	Repeated Assignments	Initial Value
82	convert_to_nmole_per_kg_1.Urine_nmole_1 = Urine_1.Urine_drug_1/drug_molar_mass_1*nanomole_per_mole*kilogram/phys_BW	0
83	convert_to_nmole_per_kg_1.Venous_nmole_1 = Venous_1.Venous_drug_1*Venous_1/drug_molar_mass_1*nanomole_per_mole*kilogram/phys_BW	0
84	convert_to_nmole_per_kg_1.Bile_nmole_1 = Main_compartment_1.Bile_drug_1/drug_molar_mass_1*nanomole_per_mole*kilogram/phys_BW	0
85	Plasma_total_1.Plasma_total_uM_1 = Plasma_total_1.Plasma_total_drug_1/drug_molar_mass_1	0
86	Liver_total_1.Liver_tissue_total_uM_1 = Liver_total_1.Liver_tissue_total_1/drug_molar_mass_1	0
87	convert_to_nmole_per_kg_1.Kidney_nmole_1 = Kidney_1.Kidney_drug_1*Kidney_1/drug_molar_mass_1*nanomole_per_mole*kilogram/phys_BW	0
88	convert_to_nmole_per_kg_1.Lung_nmole_1 = Lung_1.Lung_drug_1*Lung_1/drug_molar_mass_1*nanomole_per_mole*kilogram/phys_BW	0
89	convert_to_nmole_per_kg_1.Metabolites_nmole_1 = Metabolites_1.Metabolites_drug_1/drug_molar_mass_1*nanomole_per_mole*kilogram/phys_BW	0
90	convert_to_nmole_per_kg_1.Liver_IC2_nmole_1 = Liver_IC_S2_1.Liver_IC_S2_drug_1*Liver_IC_S2_1/drug_molar_mass_1*nanomole_per_mole*kilogram/phys_BW	0
91	convert_to_nmole_per_kg_1.Liver_EC2_nmole_1 = Liver_EC_S2_1.Liver_EC_S2_drug_1*Liver_EC_S2_1/drug_molar_mass_1*nanomole_per_mole*kilogram/phys_BW	0
92	convert_to_nmole_per_kg_1.Liver_EC3_nmole_1 = Liver_EC_S3_1.Liver_EC_S3_drug_1*Liver_EC_S3_1/drug_molar_mass_1*nanomole_per_mole*kilogram/phys_BW	0
93	convert_to_nmole_per_kg_1.Liver_IC3_nmole_1 = Liver_IC_S3_1.Liver_IC_S3_drug_1*Liver_IC_S3_1/drug_molar_mass_1*nanomole_per_mole*kilogram/phys_BW	0
94	convert_to_nmole_per_kg_1.Liver_IC4_nmole_1 = Liver_IC_S4_1.Liver_IC_S4_drug_1*Liver_IC_S4_1/drug_molar_mass_1*nanomole_per_mole*kilogram/phys_BW	0
95	convert_to_nmole_per_kg_1.Liver_EC4_nmole_1 = Liver_EC_S4_1.Liver_EC_S4_drug_1*Liver_EC_S4_1/drug_molar_mass_1*nanomole_per_mole*kilogram/phys_BW	0
96	convert_to_nmole_per_kg_1.Liver_EC5_nmole_1 = Liver_EC_S5_1.Liver_EC_S5_drug_1*Liver_EC_S5	0

	Repeated Assignments	Initial Value
	_1/drug_molar_mass_1*nanomole_per_mole*kilogram/phys_BW	
97	convert_to_nmole_per_kg_1.Liver_IC5_nmole_1 = Liver_IC_S5_1.Liver_IC_S5_drug_1*Liver_IC_S5_1/drug_molar_mass_1*nanomole_per_mole*kilogram/phys_BW	0
98	convert_to_nmole_per_kg_1.Gut_nmole_1 = Gut_1.Gut_drug_1*Gut_1/drug_molar_mass_1*nanomole_per_mole*kilogram/phys_BW	0
99	convert_to_nmole_per_kg_1.Spleen_nmole_1 = Spleen_1.Spleen_drug_1*Spleen_1/drug_molar_mass_1*nanomole_per_mole*kilogram/phys_BW	0
100	convert_to_nmole_per_kg_1.Skin_nmole_1 = Skin_1.Skin_drug_1*Skin_1/drug_molar_mass_1*nanomole_per_mole*kilogram/phys_BW	0
101	convert_to_nmole_per_kg_1.Brain_nmole_1 = Brain_1.Brain_drug_1*Brain_1/drug_molar_mass_1*nanomole_per_mole*kilogram/phys_BW	0
102	convert_to_nmole_per_kg_1.Rest_nmole_1 = Rest_1.Rest_drug_1*Rest_1/drug_molar_mass_1*nanomole_per_mole*kilogram/phys_BW	0
103	convert_to_nmole_per_kg_1.Heart_nmole_1 = Heart_1.Heart_drug_1*Heart_1/drug_molar_mass_1*nanomole_per_mole*kilogram/phys_BW	0
104	uptake_inhib_S1 = (1-switch_uptake_inhib_1)+switch_uptake_inhib_1/(1+drug_fB*Liver_EC_S1.Liver_EC_S1_drug/(drug_uptake_Ki*drug_molar_mass))	1
105	uptake_inhib_S3 = (1-switch_uptake_inhib_1)+switch_uptake_inhib_1/(1+drug_fB*Liver_EC_S3.Liver_EC_S3_drug/(drug_uptake_Ki*drug_molar_mass))	1
106	uptake_inhib_S2 = (1-switch_uptake_inhib_1)+switch_uptake_inhib_1/(1+drug_fB*Liver_EC_S2.Liver_EC_S2_drug/(drug_uptake_Ki*drug_molar_mass))	1
107	uptake_inhib_S4 = (1-switch_uptake_inhib_1)+switch_uptake_inhib_1/(1+drug_fB*Liver_EC_S4.Liver_EC_S4_drug/(drug_uptake_Ki*drug_molar_mass))	1
108	uptake_inhib_S5 = (1-switch_uptake_inhib_1)+switch_uptake_inhib_1/(1+drug_fB*Liver_EC_S5.Liver_EC_S5_drug/(drug_uptake_Ki*drug_molar_mass))	1
109	biliary_inhib_S1 = (1-switch_biliary_inhib_1)+switch_biliary_inhib_1/(1+drug_fuLiver*Liver_IC_S1.Liver_IC_S1_drug/(drug_biliary_Ki*drug_molar_mass))	1
110	biliary_inhib_S2 = (1-switch_biliary_inhib_1)+switch_biliary_inhib_1/(1+dru	1

	Repeated Assignments	Initial Value
	g_fuLiver*Liver_IC_S2.Liver_IC_S2_drug/(drug_biliary_Ki*drug_molar_mass))	
111	biliary_inhib_S3 = (1-switch_biliary_inhib_1)+switch_biliary_inhib_1/(1+drug_fuLiver*Liver_IC_S3.Liver_IC_S3_drug/(drug_biliary_Ki*drug_molar_mass))	1
112	biliary_inhib_S4 = (1-switch_biliary_inhib_1)+switch_biliary_inhib_1/(1+drug_fuLiver*Liver_IC_S4.Liver_IC_S4_drug/(drug_biliary_Ki*drug_molar_mass))	1
113	biliary_inhib_S5 = (1-switch_biliary_inhib_1)+switch_biliary_inhib_1/(1+drug_fuLiver*Liver_IC_S5.Liver_IC_S5_drug/(drug_biliary_Ki*drug_molar_mass))	1
114	met_inhib_S1 = (1-switch_met_inhib_1)+switch_met_inhib_1/(1+drug_fuLiver*Liver_IC_S1.Liver_IC_S1_drug/(drug_met_Ki*drug_molar_mass))	1
115	met_inhib_S2 = (1-switch_met_inhib_1)+switch_met_inhib_1/(1+drug_fuLiver*Liver_IC_S2.Liver_IC_S2_drug/(drug_met_Ki*drug_molar_mass))	1
116	met_inhib_S3 = (1-switch_met_inhib_1)+switch_met_inhib_1/(1+drug_fuLiver*Liver_IC_S3.Liver_IC_S3_drug/(drug_met_Ki*drug_molar_mass))	1
117	met_inhib_S4 = (1-switch_met_inhib_1)+switch_met_inhib_1/(1+drug_fuLiver*Liver_IC_S4.Liver_IC_S4_drug/(drug_met_Ki*drug_molar_mass))	1
118	met_inhib_S5 = (1-switch_met_inhib_1)+switch_met_inhib_1/(1+drug_fuLiver*Liver_IC_S5.Liver_IC_S5_drug/(drug_met_Ki*drug_molar_mass))	1
119	Kpuu_Liver_1 = Liver_total_1.Liver_tissue_free_uM_1/Plasma_total_1.Plasma_free_uM_1	NaN
120	convert_to_nmole_per_kg.X_CECUM_DISS_nmole = Colon.X_CECUM_DISS/drug_molar_mass*nanomole_per_mole*kilogram/phys_BW	0
121	efflux_inhib_duo = (1-switch_efflux_inhib_1)+switch_efflux_inhib_1/(1+fu_mem*MDUO.MEM_DUO/VDUO/(drug_efflux_Ki*drug_molar_mass))	1
122	efflux_inhib_jej1 = (1-switch_efflux_inhib_1)+switch_efflux_inhib_1/(1+fu_mem*MJEJ1.MEM_JEJ1/VJEJ1/(drug_efflux_Ki*drug_molar_mass))	1
123	efflux_inhib_jej2 = (1-switch_efflux_inhib_1)+switch_efflux_inhib_1/(1+fu_	1

	Repeated Assignments	Initial Value
	mem*MJEJ2.MEM_JEJ2/VJEJ2/(drug_efflux_Ki*drug_molar_mass))	
124	efflux_inhib_ill1 = (1-switch_efflux_inhib_1)+switch_efflux_inhib_1/(1+fu_mem*MILL1.MEM_ILL1/VILL1/(drug_efflux_Ki*drug_molar_mass))	1
125	efflux_inhib_ill2 = (1-switch_efflux_inhib_1)+switch_efflux_inhib_1/(1+fu_mem*MILL2.MEM_ILL2/VILL2/(drug_efflux_Ki*drug_molar_mass))	1
126	efflux_inhib_ill3 = (1-switch_efflux_inhib_1)+switch_efflux_inhib_1/(1+fu_mem*MILL3.MEM_ILL3/VILL3/(drug_efflux_Ki*drug_molar_mass))	1
127	efflux_inhib_ill4 = (1-switch_efflux_inhib_1)+switch_efflux_inhib_1/(1+fu_mem*MILL4.MEM_ILL4/VILL4/(drug_efflux_Ki*drug_molar_mass))	1

Reactions

	Reactions
1	Liver_IC_S5.Liver_IC_S5_drug -> Main_compartment.Bile_drug
	*(1-switch_liverFlag)*k_Liver_IC_S5_Bile*Liver_IC_S5.Liver_IC_S5_drug*
2	Liver_IC_S5.Liver_IC_S5_drug -> Metabolites.Metabolites_drug
	*(1-switch_liverFlag)*k_Liver_IC_S5_Metabolites*Liver_IC_S5.Liver_IC_S5_drug*
3	Liver_IC_S4.Liver_IC_S4_drug -> Metabolites.Metabolites_drug
	*(1-switch_liverFlag)*k_Liver_IC_S4_Metabolites*Liver_IC_S4.Liver_IC_S4_drug*
4	Liver_IC_S3.Liver_IC_S3_drug -> Metabolites.Metabolites_drug
	*(1-switch_liverFlag)*k_Liver_IC_S3_Metabolites*Liver_IC_S3.Liver_IC_S3_drug*
5	Liver_IC_S2.Liver_IC_S2_drug -> Metabolites.Metabolites_drug
	*(1-switch_liverFlag)*k_Liver_IC_S2_Metabolites*Liver_IC_S2.Liver_IC_S2_drug*
6	Liver_IC_S1.Liver_IC_S1_drug -> Metabolites.Metabolites_drug
	*(1-switch_liverFlag)*k_Liver_IC_S1_Metabolites*Liver_IC_S1.Liver_IC_S1_drug*
7	Liver_IC_S4.Liver_IC_S4_drug -> Main_compartment.Bile_drug
	*(1-switch_liverFlag)*k_Liver_IC_S4_Bile*Liver_IC_S4.Liver_IC_S4_drug*
8	Liver_IC_S3.Liver_IC_S3_drug -> Main_compartment.Bile_drug
	*(1-switch_liverFlag)*k_Liver_IC_S3_Bile*Liver_IC_S3.Liver_IC_S3_drug*
9	Liver_IC_S2.Liver_IC_S2_drug -> Main_compartment.Bile_drug
	*(1-switch_liverFlag)*k_Liver_IC_S2_Bile*Liver_IC_S2.Liver_IC_S2_drug*
10	Liver_IC_S1.Liver_IC_S1_drug -> Main_compartment.Bile_drug
	*(1-switch_liverFlag)*k_Liver_IC_S1_Bile*Liver_IC_S1.Liver_IC_S1_drug*
11	Liver_IC_S5.Liver_IC_S5_drug -> Liver_EC_S5.Liver_EC_S5_drug
	*k_Liver_IC_S5_Liver_EC_S5*Liver_IC_S5.Liver_IC_S5_drug*

	Reactions
12	Liver_EC_S5.Liver_EC_S5_drug -> Liver_IC_S5.Liver_IC_S5_drug
	*k_Liver_EC_S5_Liver_IC_S5*Liver_EC_S5.Liver_EC_S5_drug*
13	Liver_IC_S4.Liver_IC_S4_drug -> Liver_EC_S4.Liver_EC_S4_drug
	*k_Liver_IC_S4_Liver_EC_S4*Liver_IC_S4.Liver_IC_S4_drug*
14	Liver_EC_S4.Liver_EC_S4_drug -> Liver_IC_S4.Liver_IC_S4_drug
	*k_Liver_EC_S4_Liver_IC_S4*Liver_EC_S4.Liver_EC_S4_drug*
15	Liver_IC_S3.Liver_IC_S3_drug -> Liver_EC_S3.Liver_EC_S3_drug
	*k_Liver_IC_S3_Liver__EC_S3*Liver_IC_S3.Liver_IC_S3_drug*
16	Liver_EC_S3.Liver_EC_S3_drug -> Liver_IC_S3.Liver_IC_S3_drug
	*k_Liver_EC_S3_Liver_IC_S3*Liver_EC_S3.Liver_EC_S3_drug*
17	Liver_IC_S2.Liver_IC_S2_drug -> Liver_EC_S2.Liver_EC_S2_drug
	*k_Liver_IC_S2_Liver_EC_S2*Liver_IC_S2.Liver_IC_S2_drug*
18	Liver_EC_S2.Liver_EC_S2_drug -> Liver_IC_S2.Liver_IC_S2_drug
	*k_Liver_EC_S2_Liver_IC_S2*Liver_EC_S2.Liver_EC_S2_drug*
19	Liver_IC_S1.Liver_IC_S1_drug -> Liver_EC_S1.Liver_EC_S1_drug
	*k_Liver_IC_S1_Liver_EC_S1*Liver_IC_S1.Liver_IC_S1_drug*
20	Liver_EC_S1.Liver_EC_S1_drug -> Liver_IC_S1.Liver_IC_S1_drug
	*k_Liver_EC_S1_Liver_IC_S1*Liver_EC_S1.Liver_EC_S1_drug*
21	Liver.Liver_drug -> Venous.Venous_drug
	*switch_liverFlag*k_Liver_Venous*Liver.Liver_drug*
22	Liver_EC_S4.Liver_EC_S4_drug -> Liver_EC_S5.Liver_EC_S5_drug
	*k_Liver_EC_S4_Liver_EC_S5*Liver_EC_S4.Liver_EC_S4_drug*
23	Liver_EC_S3.Liver_EC_S3_drug -> Liver_EC_S4.Liver_EC_S4_drug
	*k_Liver_EC_S3_Liver_EC_S4*Liver_EC_S3.Liver_EC_S3_drug*
24	Liver_EC_S2.Liver_EC_S2_drug -> Liver_EC_S3.Liver_EC_S3_drug
	*k_Liver_EC_S2_Liver_EC_S3*Liver_EC_S2.Liver_EC_S2_drug*
25	Liver_EC_S1.Liver_EC_S1_drug -> Liver_EC_S2.Liver_EC_S2_drug
	*k_Liver_EC_S1_Liver_EC_S2*Liver_EC_S1.Liver_EC_S1_drug*
26	Artery.Artery_drug -> Liver.Liver_drug
	*switch_liverFlag*k_artery_liver*Artery.Artery_drug*
27	Artery.Artery_drug -> VillousILL1.Villous_ILL1
	*Qmuc_ILL1*Artery.Artery_drug*
28	Artery.Artery_drug -> Spleen.Spleen_drug
	*k_artery_spleen*Artery.Artery_drug*
29	Rest.Rest_drug -> Venous.Venous_drug
	*k_rest_venous*Rest.Rest_drug*
30	Bone.Bone_drug -> Venous.Venous_drug
	*k_bone_venous*Bone.Bone_drug*

	Reactions
31	Skin.Skin_drug -> Venous.Venous_drug
	*k_skin_venous*Skin.Skin_drug*
32	Heart.Heart_drug -> Venous.Venous_drug
	*k_heart_venous*Heart.Heart_drug*
33	Adipose.Adipose_drug -> Venous.Venous_drug
	*k_adipos_venous*Adipose.Adipose_drug*
34	Muscle.Muscle_drug -> Venous.Venous_drug
	*k_muscle_venous*Muscle.Muscle_drug*
35	Brain.Brain_drug -> Venous.Venous_drug
	*k_brain_venous*Brain.Brain_drug*
36	Kidney.Kidney_drug -> Venous.Venous_drug
	*k_kidney_venous*Kidney.Kidney_drug*
37	Artery.Artery_drug -> Rest.Rest_drug
	*k_artery_rest*Artery.Artery_drug*
38	Artery.Artery_drug -> Bone.Bone_drug
	*k_artery_bone*Artery.Artery_drug*
39	Artery.Artery_drug -> Skin.Skin_drug
	*k_artery_skin*Artery.Artery_drug*
40	Artery.Artery_drug -> Heart.Heart_drug
	*k_artery_heart*Artery.Artery_drug*
41	Artery.Artery_drug -> Adipose.Adipose_drug
	*k_artery_adipos*Artery.Artery_drug*
42	Artery.Artery_drug -> Muscle.Muscle_drug
	*k_artery_muscle*Artery.Artery_drug*
43	Artery.Artery_drug -> Brain.Brain_drug
	*k_artery_brain*Artery.Artery_drug*
44	Artery.Artery_drug -> Kidney.Kidney_drug
	*k_artery_kidney*Artery.Artery_drug*
45	Venous.Venous_drug -> Lung.Lung_drug
	*k_venous_lung*Venous.Venous_drug*
46	Lung.Lung_drug -> Artery.Artery_drug
	*k_lung_artery*Lung.Lung_drug*
47	Venous.Venous_drug -> Urine.Urine_drug
	*k_venous_urine_CLR*Venous.Venous_drug*
48	Artery.Artery_drug -> Testes.Testes_drug
	*k_artery_testes*Artery.Artery_drug*
49	Testes.Testes_drug -> Venous.Venous_drug
	*k_testes_venous*Testes.Testes_drug*

	Reactions
50	Liver_IC_S4.Liver_IC_S4_drug -> Liver_EC_S4.Liver_EC_S4_drug
	*k_Liver_IC_S4_Liver_EC_S4_efflux*Liver_IC_S4.Liver_IC_S4_drug*
51	Liver_IC_S3.Liver_IC_S3_drug -> Liver_EC_S3.Liver_EC_S3_drug
	*k_Liver_IC_S3_Liver_EC_S3_efflux*Liver_IC_S3.Liver_IC_S3_drug*
52	Liver_IC_S2.Liver_IC_S2_drug -> Liver_EC_S2.Liver_EC_S2_drug
	*k_Liver_IC_S2_Liver_EC_S2_efflux*Liver_IC_S2.Liver_IC_S2_drug*
53	Liver_IC_S5.Liver_IC_S5_drug -> Liver_EC_S5.Liver_EC_S5_drug
	*k_Liver_IC_S5_Liver_EC_S5_efflux*Liver_IC_S5.Liver_IC_S5_drug*
54	Liver_IC_S1.Liver_IC_S1_drug -> Liver_EC_S1.Liver_EC_S1_drug
	*k_Liver_IC_S1_Liver_EC_S1_efflux*Liver_IC_S1.Liver_IC_S1_drug*
55	Venous.Venous_drug -> Urine.Urine_drug
	*k_venous_urine_GFR*Venous.Venous_drug*
56	Liver_IC_S5_1.Liver_IC_S5_drug_1 -> Main_compartment_1.Bile_drug_1
	*(1-switch_liverFlag_1)*k_Liver_IC_S5_Bile_1*Liver_IC_S5_1.Liver_IC_S5_drug_1*
57	Liver_IC_S5_1.Liver_IC_S5_drug_1 -> Metabolites_1.Metabolites_drug_1
	*(1-switch_liverFlag_1)*k_Liver_IC_S5_Metabolites_1*Liver_IC_S5_1.Liver_IC_S5_drug_1*
58	Liver_IC_S4_1.Liver_IC_S4_drug_1 -> Metabolites_1.Metabolites_drug_1
	*(1-switch_liverFlag_1)*k_Liver_IC_S4_Metabolites_1*Liver_IC_S4_1.Liver_IC_S4_drug_1*
59	Liver_IC_S3_1.Liver_IC_S3_drug_1 -> Metabolites_1.Metabolites_drug_1
	*(1-switch_liverFlag_1)*k_Liver_IC_S3_Metabolites_1*Liver_IC_S3_1.Liver_IC_S3_drug_1*
60	Liver_IC_S2_1.Liver_IC_S2_drug_1 -> Metabolites_1.Metabolites_drug_1
	*(1-switch_liverFlag_1)*k_Liver_IC_S2_Metabolites_1*Liver_IC_S2_1.Liver_IC_S2_drug_1*
61	Liver_IC_S1_1.Liver_IC_S1_drug_1 -> Metabolites_1.Metabolites_drug_1
	*(1-switch_liverFlag_1)*k_Liver_IC_S1_Metabolites_1*Liver_IC_S1_1.Liver_IC_S1_drug_1*
62	Liver_IC_S4_1.Liver_IC_S4_drug_1 -> Main_compartment_1.Bile_drug_1
	*(1-switch_liverFlag_1)*k_Liver_IC_S4_Bile_1*Liver_IC_S4_1.Liver_IC_S4_drug_1*
63	Liver_IC_S3_1.Liver_IC_S3_drug_1 -> Main_compartment_1.Bile_drug_1
	*(1-switch_liverFlag_1)*k_Liver_IC_S3_Bile_1*Liver_IC_S3_1.Liver_IC_S3_drug_1*
64	Liver_IC_S2_1.Liver_IC_S2_drug_1 -> Main_compartment_1.Bile_drug_1
	*(1-switch_liverFlag_1)*k_Liver_IC_S2_Bile_1*Liver_IC_S2_1.Liver_IC_S2_drug_1*
65	Liver_IC_S1_1.Liver_IC_S1_drug_1 -> Main_compartment_1.Bile_drug_1
	*(1-switch_liverFlag_1)*k_Liver_IC_S1_Bile_1*Liver_IC_S1_1.Liver_IC_S1_drug_1*
66	Liver_IC_S5_1.Liver_IC_S5_drug_1 -> Liver_EC_S5_1.Liver_EC_S5_drug_1
	*k_Liver_IC_S5_Liver_EC_S5_1*Liver_IC_S5_1.Liver_IC_S5_drug_1*
67	Liver_EC_S5_1.Liver_EC_S5_drug_1 -> Liver_IC_S5_1.Liver_IC_S5_drug_1
	*k_Liver_EC_S5_Liver_IC_S5_1*Liver_EC_S5_1.Liver_EC_S5_drug_1*
68	Liver_IC_S4_1.Liver_IC_S4_drug_1 -> Liver_EC_S4_1.Liver_EC_S4_drug_1
	*k_Liver_IC_S4_Liver_EC_S4_1*Liver_IC_S4_1.Liver_IC_S4_drug_1*

	Reactions
69	Liver_EC_S4_1.Liver_EC_S4_drug_1 -> Liver_IC_S4_1.Liver_IC_S4_drug_1
	*k_Liver_EC_S4_Liver_IC_S4_1*Liver_EC_S4_1.Liver_EC_S4_drug_1*
70	Liver_IC_S3_1.Liver_IC_S3_drug_1 -> Liver_EC_S3_1.Liver_EC_S3_drug_1
	*k_Liver_IC_S3_Liver__EC_S3_1*Liver_IC_S3_1.Liver_IC_S3_drug_1*
71	Liver_EC_S3_1.Liver_EC_S3_drug_1 -> Liver_IC_S3_1.Liver_IC_S3_drug_1
	*k_Liver_EC_S3_Liver_IC_S3_1*Liver_EC_S3_1.Liver_EC_S3_drug_1*
72	Liver_IC_S2_1.Liver_IC_S2_drug_1 -> Liver_EC_S2_1.Liver_EC_S2_drug_1
	*k_Liver_IC_S2_Liver_EC_S2_1*Liver_IC_S2_1.Liver_IC_S2_drug_1*
73	Liver_EC_S2_1.Liver_EC_S2_drug_1 -> Liver_IC_S2_1.Liver_IC_S2_drug_1
	*k_Liver_EC_S2_Liver_IC_S2_1*Liver_EC_S2_1.Liver_EC_S2_drug_1*
74	Liver_IC_S1_1.Liver_IC_S1_drug_1 -> Liver_EC_S1_1.Liver_EC_S1_drug_1
	*k_Liver_IC_S1_Liver_EC_S1_1*Liver_IC_S1_1.Liver_IC_S1_drug_1*
75	Liver_EC_S1_1.Liver_EC_S1_drug_1 -> Liver_IC_S1_1.Liver_IC_S1_drug_1
	*k_Liver_EC_S1_Liver_IC_S1_1*Liver_EC_S1_1.Liver_EC_S1_drug_1*
76	Liver_EC_S5_1.Liver_EC_S5_drug_1 -> Venous_1.Venous_drug_1
	*(1-switch_liverFlag_1)*k_Liver_EC_S5_Venous_1*Liver_EC_S5_1.Liver_EC_S5_drug_1*
77	Liver_EC_S4_1.Liver_EC_S4_drug_1 -> Liver_EC_S5_1.Liver_EC_S5_drug_1
	*k_Liver_EC_S4_Liver_EC_S5_1*Liver_EC_S4_1.Liver_EC_S4_drug_1*
78	Liver_EC_S3_1.Liver_EC_S3_drug_1 -> Liver_EC_S4_1.Liver_EC_S4_drug_1
	*k_Liver_EC_S3_Liver_EC_S4_1*Liver_EC_S3_1.Liver_EC_S3_drug_1*
79	Liver_EC_S2_1.Liver_EC_S2_drug_1 -> Liver_EC_S3_1.Liver_EC_S3_drug_1
	*k_Liver_EC_S2_Liver_EC_S3_1*Liver_EC_S2_1.Liver_EC_S2_drug_1*
80	Liver_EC_S1_1.Liver_EC_S1_drug_1 -> Liver_EC_S2_1.Liver_EC_S2_drug_1
	*k_Liver_EC_S1_Liver_EC_S2_1*Liver_EC_S1_1.Liver_EC_S1_drug_1*
81	Gut_1.Gut_drug_1 -> Liver_EC_S1_1.Liver_EC_S1_drug_1
	*k_gut_liver_1*Gut_1.Gut_drug_1*
82	Spleen_1.Spleen_drug_1 -> Liver_EC_S1_1.Liver_EC_S1_drug_1
	*(1-switch_liverFlag_1)*k_spleen_liver_1*Spleen_1.Spleen_drug_1*
83	Artery_1.Artery_drug_1 -> Liver_EC_S1_1.Liver_EC_S1_drug_1
	*(1-switch_liverFlag_1)*k_artery_liver_1*Artery_1.Artery_drug_1*
84	Artery_1.Artery_drug_1 -> Gut_1.Gut_drug_1
	*k_artery_gut_1*Artery_1.Artery_drug_1*
85	Artery_1.Artery_drug_1 -> Spleen_1.Spleen_drug_1
	*k_artery_spleen_1*Artery_1.Artery_drug_1*
86	Rest_1.Rest_drug_1 -> Venous_1.Venous_drug_1
	*k_rest_venous_1*Rest_1.Rest_drug_1*
87	Bone_1.Bone_drug_1 -> Venous_1.Venous_drug_1
	*k_bone_venous_1*Bone_1.Bone_drug_1*

	Reactions
88	Skin_1.Skin_drug_1 -> Venous_1.Venous_drug_1
	*k_skin_venous_1*Skin_1.Skin_drug_1*
89	Heart_1.Heart_drug_1 -> Venous_1.Venous_drug_1
	*k_heart_venous_1*Heart_1.Heart_drug_1*
90	Adipose_1.Adipose_drug_1 -> Venous_1.Venous_drug_1
	*k_adipos_venous_1*Adipose_1.Adipose_drug_1*
91	Muscle_1.Muscle_drug_1 -> Venous_1.Venous_drug_1
	*k_muscle_venous_1*Muscle_1.Muscle_drug_1*
92	Brain_1.Brain_drug_1 -> Venous_1.Venous_drug_1
	*k_brain_venous_1*Brain_1.Brain_drug_1*
93	Kidney_1.Kidney_drug_1 -> Venous_1.Venous_drug_1
	*k_kidney_venous_1*Kidney_1.Kidney_drug_1*
94	Artery_1.Artery_drug_1 -> Rest_1.Rest_drug_1
	*k_artery_rest_1*Artery_1.Artery_drug_1*
95	Artery_1.Artery_drug_1 -> Bone_1.Bone_drug_1
	*k_artery_bone_1*Artery_1.Artery_drug_1*
96	Artery_1.Artery_drug_1 -> Skin_1.Skin_drug_1
	*k_artery_skin_1*Artery_1.Artery_drug_1*
97	Artery_1.Artery_drug_1 -> Heart_1.Heart_drug_1
	*k_artery_heart_1*Artery_1.Artery_drug_1*
98	Artery_1.Artery_drug_1 -> Adipose_1.Adipose_drug_1
	*k_artery_adipos_1*Artery_1.Artery_drug_1*
99	Artery_1.Artery_drug_1 -> Muscle_1.Muscle_drug_1
	*k_artery_muscle_1*Artery_1.Artery_drug_1*
100	Artery_1.Artery_drug_1 -> Brain_1.Brain_drug_1
	*k_artery_brain_1*Artery_1.Artery_drug_1*
101	Artery_1.Artery_drug_1 -> Kidney_1.Kidney_drug_1
	*k_artery_kidney_1*Artery_1.Artery_drug_1*
102	Venous_1.Venous_drug_1 -> Lung_1.Lung_drug_1
	*k_venous_lung_1*Venous_1.Venous_drug_1*
103	Lung_1.Lung_drug_1 -> Artery_1.Artery_drug_1
	*k_lung_artery_1*Lung_1.Lung_drug_1*
104	Venous_1.Venous_drug_1 -> Urine_1.Urine_drug_1
	*k_venous_urine_CLR_1*Venous_1.Venous_drug_1*
105	Artery_1.Artery_drug_1 -> Testes_1.Testes_drug_1
	*k_artery_testes_1*Artery_1.Artery_drug_1*
106	Testes_1.Testes_drug_1 -> Venous_1.Venous_drug_1
	*k_testes_venous_1*Testes_1.Testes_drug_1*

	Reactions
107	Venous_1.Venous_drug_1 -> Urine_1.Urine_drug_1
	*k_venous_urine_GFR_1*Venous_1.Venous_drug_1*
108	Liver_IC_S5_1.Liver_IC_S5_drug_1 -> Liver_EC_S5_1.Liver_EC_S5_drug_1
	*k_Liver_IC_S5_Liver_EC_S5_efflux_1*Liver_IC_S5_1.Liver_IC_S5_drug_1*
109	Liver_IC_S4_1.Liver_IC_S4_drug_1 -> Liver_EC_S4_1.Liver_EC_S4_drug_1
	*k_Liver_IC_S4_Liver_EC_S4_efflux_1*Liver_IC_S4_1.Liver_IC_S4_drug_1*
110	Liver_IC_S3_1.Liver_IC_S3_drug_1 -> Liver_EC_S3_1.Liver_EC_S3_drug_1
	*k_Liver_IC_S3_Liver_EC_S3_efflux_1*Liver_IC_S3_1.Liver_IC_S3_drug_1*
111	Liver_IC_S2_1.Liver_IC_S2_drug_1 -> Liver_EC_S2_1.Liver_EC_S2_drug_1
	*k_Liver_IC_S2_Liver_EC_S2_efflux_1*Liver_IC_S2_1.Liver_IC_S2_drug_1*
112	Liver_IC_S1_1.Liver_IC_S1_drug_1 -> Liver_EC_S1_1.Liver_EC_S1_drug_1
	*k_Liver_IC_S1_Liver_EC_S1_efflux_1*Liver_IC_S1_1.Liver_IC_S1_drug_1*
113	Main_compartment.Bile_drug -> VDUO.X_DUO_DISS
	*k_transit*Main_compartment.Bile_drug*
114	Gut_Lumen.Gut_Lumen_drug -> Gut.Gut_drug
	*drug_k_oral*drug_fa*Gut_Lumen.Gut_Lumen_drug*
115	Main_compartment.Bile_drug -> null
	*drug_k_bile_deg*Main_compartment.Bile_drug*
116	Gut_Lumen.Gut_Lumen_drug -> null
	drug_k_oral(1-drug_fa)*Gut_Lumen.Gut_Lumen_drug*
117	Gut.Gut_drug -> null
	*k_gut_liver*Gut.Gut_drug*
118	Spleen.Spleen_drug -> Liver.Liver_drug
	*switch_liverFlag*k_spleen_liver*Spleen.Spleen_drug*
119	Gut_Lumen_1.Gut_Lumen_drug_1 -> Gut_1.Gut_drug_1
	*drug_k_oral_1*drug_fa_1*Gut_Lumen_1.Gut_Lumen_drug_1*
120	Main_compartment_1.Bile_drug_1 -> Gut_Lumen_1.Gut_Lumen_drug_1
	*k_transit_1*Main_compartment_1.Bile_drug_1*
121	Main_compartment_1.Bile_drug_1 -> null
	*drug_k_bile_deg_1*Main_compartment_1.Bile_drug_1*
122	Gut_Lumen_1.Gut_Lumen_drug_1 -> null
	drug_k_oral_1(1-drug_fa_1)*Gut_Lumen_1.Gut_Lumen_drug_1*
123	STOMACH.X_STOMACH_SOLID -> VDUO.X_DUO_SOLID
	STOMACH.X_STOMACH_SOLID/TSTOMACH
124	STOMACH.X_STOMACH_DISS -> VDUO.X_DUO_DISS
	STOMACH.X_STOMACH_DISS/TSTOMACH
125	VDUO.X_DUO_SOLID -> VJEJ1.X_JEJ1_SOLID
	VDUO.X_DUO_SOLID/TDUO

	**Reactions**
126	VDUO.X_DUO_SOLID -> VDUO.X_DUO_DISS
	*KD*VDUO.X_DUO_SOLID*(SOLIF_DUO-VDUO.X_DUO_DISS/VDUO)*
127	VDUO.X_DUO_DISS -> VJEJ1.X_JEJ1_DISS
	VDUO.X_DUO_DISS/TDUO
128	VDUO.X_DUO_DISS -> MDUO.MEM_DUO
	*(DIFF_duo*NI_DUO*switch_SFdiffapi*VDUO.X_DUO_DISS)/VDUO*
129	MDUO.MEM_DUO -> VDUO.X_DUO_DISS
	*((switchVmax_efflux==zero)*CLINT_efflux_DUO*efflux_factor_duo*switch_SFefflux+switchVmax_efflux*phys_Normalized_ESA*phys_BW*surfaceRatio_DUO*efflux_factor_duo*switch_SFefflux/(drug_Km_efflux+MDUO.MEM_DUO*fu_mem/MDUO/drug_molar_mass))*MDUO.MEM_DUO*fu_mem/MDUO*
130	VDUO.X_DUO_DISS -> MDUO.MEM_DUO
	*((switchVmax_influx==zero)*CLINT_influx_DUO*influx_factor_duo*switch_SFinflux+switchVmax_influx*phys_Normalized_ESA*phys_BW*surfaceRatio_DUO*influx_factor_duo/(drug_Km_influx+VDUO.X_DUO_DISS/VDUO/drug_molar_mass))*VDUO.X_DUO_DISS/VDUO*
131	VillousDUO.Villous_DUO -> Liver.Liver_drug
	*switch_liverFlag*VillousDUO.Villous_DUO*Qmuc_DUO/VillousDUO*
132	MDUO.MEM_DUO -> null
	*(CLINT_metabolism*metabolism_factor_duo*switch_SFgutmet*MDUO.MEM_DUO*fu_mem)/MDUO*
133	VJEJ1.X_JEJ1_SOLID -> VJEJ2.X_JEJ2_SOLID
	VJEJ1.X_JEJ1_SOLID/TJEJ1
134	VJEJ1.X_JEJ1_SOLID -> VJEJ1.X_JEJ1_DISS
	*KD*VJEJ1.X_JEJ1_SOLID*(SOLIF_JEJ1-VJEJ1.X_JEJ1_DISS/VJEJ1)*
135	VJEJ1.X_JEJ1_DISS -> VJEJ2.X_JEJ2_DISS
	VJEJ1.X_JEJ1_DISS/TJEJ1
136	VJEJ1.X_JEJ1_DISS -> MJEJ1.MEM_JEJ1
	*(DIFF_jej1*NI_JEJ1*switch_SFdiffapi*VJEJ1.X_JEJ1_DISS)/VJEJ1*
137	MJEJ1.MEM_JEJ1 -> VJEJ1.X_JEJ1_DISS
	*((switchVmax_efflux==zero)*CLINT_efflux_JEJ1*efflux_factor_jej1*switch_SFefflux+switchVmax_efflux*phys_Normalized_ESA*phys_BW*surfaceRatio_JEJ1*efflux_factor_jej1*switch_SFefflux/(drug_Km_efflux+MJEJ1.MEM_JEJ1*fu_mem/MJEJ1/drug_molar_mass))*MJEJ1.MEM_JEJ1*fu_mem/MJEJ1*
138	VJEJ1.X_JEJ1_DISS -> MJEJ1.MEM_JEJ1
	*((switchVmax_influx==zero)*CLINT_influx_JEJ1*influx_factor_jej1*switch_SFinflux+switchVmax_influx*phys_Normalized_ESA*phys_BW*surfaceRatio_JEJ1*influx_factor_jej1/(drug_Km_influx+VJEJ1.X_JEJ1_DISS/VJEJ1/drug_molar_mass))*VJEJ1.X_JEJ1_DISS/VJEJ1*
139	VillousJEJ1.Villous_JEJ1 -> Liver.Liver_drug
	*switch_liverFlag*VillousJEJ1.Villous_JEJ1*Qmuc_JEJ1/VillousJEJ1*
140	MJEJ1.MEM_JEJ1 -> null
	*(CLINT_metabolism*metabolism_factor_jej1*switch_SFgutmet*MJEJ1.MEM_JEJ1*fu_mem)/MJEJ1*
141	VJEJ2.X_JEJ2_SOLID -> VILL1.X_ILL1_SOLID
	VJEJ2.X_JEJ2_SOLID/TJEJ2

	Reactions
142	VJEJ2.X_JEJ2_SOLID -> VJEJ2.X_JEJ2_DISS
	*KD*VJEJ2.X_JEJ2_SOLID*(SOLIF_JEJ2-VJEJ2.X_JEJ2_DISS/VJEJ2)*
143	VJEJ2.X_JEJ2_DISS -> VILL1.X_ILL1_DISS
	VJEJ2.X_JEJ2_DISS/TJEJ2
144	VJEJ2.X_JEJ2_DISS -> MJEJ2.MEM_JEJ2
	*(DIFF_jej2*NI_JEJ2*switch_SFdiffapi*VJEJ2.X_JEJ2_DISS)/VJEJ2*
145	MJEJ2.MEM_JEJ2 -> VJEJ2.X_JEJ2_DISS
	*((switchVmax_efflux==zero)*CLINT_efflux_JEJ2*efflux_factor_jej2*switch_SFefflux+switchVmax_efflux*phys_Normalized_ESA*phys_BW*surfaceRatio_JEJ2*efflux_factor_jej2*switch_SFefflux/(drug_Km_efflux+MJEJ2.MEM_JEJ2*fu_mem/MJEJ2/drug_molar_mass))*MJEJ2.MEM_JEJ2*fu_mem/MJEJ2*
146	VJEJ2.X_JEJ2_DISS -> MJEJ2.MEM_JEJ2
	*((switchVmax_influx==zero)*CLINT_influx_JEJ2*influx_factor_jej2*switch_SFinflux+switchVmax_influx*phys_Normalized_ESA*phys_BW*surfaceRatio_JEJ2*influx_factor_jej2/(drug_Km_influx+VJEJ2.X_JEJ2_DISS/VJEJ2/drug_molar_mass))*VJEJ2.X_JEJ2_DISS/VJEJ2*
147	VillousJEJ2.Villous_JEJ2 -> Liver.Liver_drug
	*switch_liverFlag*VillousJEJ2.Villous_JEJ2*Qmuc_JEJ2/VillousJEJ2*
148	MJEJ2.MEM_JEJ2 -> null
	*(CLINT_metabolism*metabolism_factor_jej2*switch_SFgutmet*MJEJ2.MEM_JEJ2*fu_mem)/MJEJ2*
149	VILL1.X_ILL1_SOLID -> VILL2.X_ILL2_SOLID
	VILL1.X_ILL1_SOLID/TILL1
150	VILL1.X_ILL1_SOLID -> VILL1.X_ILL1_DISS
	*KD*VILL1.X_ILL1_SOLID*(SOLIF_ILL1-VILL1.X_ILL1_DISS/VILL1)*
151	VILL1.X_ILL1_DISS -> VILL2.X_ILL2_DISS
	VILL1.X_ILL1_DISS/TILL1
152	VILL1.X_ILL1_DISS -> MILL1.MEM_ILL1
	*(DIFF_ill1*NI_ILL1*switch_SFdiffapi*VILL1.X_ILL1_DISS)/VILL1*
153	MILL1.MEM_ILL1 -> VILL1.X_ILL1_DISS
	*((switchVmax_efflux==zero)*CLINT_efflux_ILL1*efflux_factor_ill1*switch_SFefflux+switchVmax_efflux*phys_Normalized_ESA*phys_BW*surfaceRatio_ILL1*efflux_factor_ill1*switch_SFefflux/(drug_Km_efflux+MILL1.MEM_ILL1*fu_mem/MILL1/drug_molar_mass))*MILL1.MEM_ILL1*fu_mem/MILL1*
154	VILL1.X_ILL1_DISS -> MILL1.MEM_ILL1
	*((switchVmax_influx==zero)*CLINT_influx_ILL1*influx_factor_ill1*switch_SFinflux+switchVmax_influx*phys_Normalized_ESA*phys_BW*surfaceRatio_ILL1*influx_factor_ill1/(drug_Km_influx+VILL1.X_ILL1_DISS/VILL1/drug_molar_mass))*VILL1.X_ILL1_DISS/VILL1*
155	VillousILL1.Villous_ILL1 -> Liver.Liver_drug
	*switch_liverFlag*VillousILL1.Villous_ILL1*Qmuc_ILL1/VillousILL1*
156	MILL1.MEM_ILL1 -> null
	*(CLINT_metabolism*metabolism_factor_ill1*switch_SFgutmet*MILL1.MEM_ILL1*fu_mem)/MILL1*
157	VILL2.X_ILL2_SOLID -> VILL3.X_ILL3_SOLID
	VILL2.X_ILL2_SOLID/TILL2

	Reactions
158	VILL2.X_ILL2_SOLID -> VILL2.X_ILL2_DISS
	*KD*VILL2.X_ILL2_SOLID*(SOLIF_ILL2-VILL2.X_ILL2_DISS/VILL2)*
159	VILL2.X_ILL2_DISS -> VILL3.X_ILL3_DISS
	VILL2.X_ILL2_DISS/TILL2
160	VILL2.X_ILL2_DISS -> MILL2.MEM_ILL2
	*(DIFF_ill2*NI_ILL2*switch_SFdiffapi*VILL2.X_ILL2_DISS)/VILL2*
161	MILL2.MEM_ILL2 -> VILL2.X_ILL2_DISS
	*((switchVmax_efflux==zero)*CLINT_efflux_ILL2*efflux_factor_ill2*switch_SFefflux+switchVmax_efflux*phys_Normalized_ESA*phys_BW*surfaceRatio_ILL2*efflux_factor_ill2*switch_SFefflux/(drug_Km_efflux+MILL2.MEM_ILL2*fu_mem/MILL2/drug_molar_mass))*MILL2.MEM_ILL2*fu_mem/MILL2*
162	VILL2.X_ILL2_DISS -> MILL2.MEM_ILL2
	*((switchVmax_influx==zero)*CLINT_influx_ILL2*influx_factor_ill2*switch_SFinflux+switchVmax_influx*phys_Normalized_ESA*phys_BW*surfaceRatio_ILL2*influx_factor_ill2/(drug_Km_influx+VILL2.X_ILL2_DISS/VILL2/drug_molar_mass))*VILL2.X_ILL2_DISS/VILL2*
163	VillousILL2.Villous_ILL2 -> Liver.Liver_drug
	*switch_liverFlag*VillousILL2.Villous_ILL2*Qmuc_ILL2/VillousILL2*
164	MILL2.MEM_ILL2 -> null
	*(CLINT_metabolism*metabolism_factor_ill2*switch_SFgutmet*MILL2.MEM_ILL2*fu_mem)/MILL2*
165	VILL3.X_ILL3_SOLID -> VILL4.X_ILL4_SOLID
	VILL3.X_ILL3_SOLID/TILL3
166	VILL3.X_ILL3_SOLID -> VILL3.X_ILL3_DISS
	*KD*VILL3.X_ILL3_SOLID*(SOLIF_ILL3-VILL3.X_ILL3_DISS/VILL3)*
167	VILL3.X_ILL3_DISS -> VILL4.X_ILL4_DISS
	VILL3.X_ILL3_DISS/TILL3
168	VILL3.X_ILL3_DISS -> MILL3.MEM_ILL3
	*(DIFF_ill3*NI_ILL3*switch_SFdiffapi*VILL3.X_ILL3_DISS)/VILL3*
169	MILL3.MEM_ILL3 -> VILL3.X_ILL3_DISS
	*((switchVmax_efflux==zero)*CLINT_efflux_ILL3*efflux_factor_ill3*switch_SFefflux+switchVmax_efflux*phys_Normalized_ESA*phys_BW*surfaceRatio_ILL3*efflux_factor_ill3*switch_SFefflux/(drug_Km_efflux+MILL3.MEM_ILL3*fu_mem/MILL3/drug_molar_mass))*MILL3.MEM_ILL3*fu_mem/MILL3*
170	VILL3.X_ILL3_DISS -> MILL3.MEM_ILL3
	*((switchVmax_influx==zero)*CLINT_influx_ILL3*influx_factor_ill3*switch_SFinflux+switchVmax_influx*phys_Normalized_ESA*phys_BW*surfaceRatio_ILL3*influx_factor_ill3/(drug_Km_influx+VILL3.X_ILL3_DISS/VILL3/drug_molar_mass))*VILL3.X_ILL3_DISS/VILL3*
171	VillousILL3.Villous_ILL3 -> Liver.Liver_drug
	*switch_liverFlag*VillousILL3.Villous_ILL3*Qmuc_ILL3/VillousILL3*
172	MILL3.MEM_ILL3 -> null
	*(CLINT_metabolism*metabolism_factor_ill3*switch_SFgutmet*MILL3.MEM_ILL3*fu_mem)/MILL3*
173	VILL4.X_ILL4_SOLID -> Colon.X_CECUM_SOLID
	VILL4.X_ILL4_SOLID/TILL4

	Reactions
174	VILL4.X_ILL4_SOLID -> VILL4.X_ILL4_DISS
	*KD*VILL4.X_ILL4_SOLID*(SOLIF_ILL4-VILL4.X_ILL4_DISS/VILL4)*
175	VILL4.X_ILL4_DISS -> Colon.X_CECUM_DISS
	VILL4.X_ILL4_DISS/TILL4
176	VILL4.X_ILL4_DISS -> MILL4.MEM_ILL4
	*(DIFF_ill4*NI_ILL4*switch_SFdiffapi*VILL4.X_ILL4_DISS)/VILL4*
177	MILL4.MEM_ILL4 -> VILL4.X_ILL4_DISS
	*((switchVmax_efflux==zero)*CLINT_efflux_ILL4*efflux_factor_ill4*switch_SFefflux+switchVmax_efflux*phys_Normalized_ESA*phys_BW*surfaceRatio_ILL4*efflux_factor_ill4*switch_SFefflux/(drug_Km_efflux+MILL4.MEM_ILL4*fu_mem/MILL4/drug_molar_mass))*MILL4.MEM_ILL4*fu_mem/MILL4*
178	VILL4.X_ILL4_DISS -> MILL4.MEM_ILL4
	*((switchVmax_influx==zero)*CLINT_influx_ILL4*influx_factor_ill4*switch_SFinflux+switchVmax_influx*phys_Normalized_ESA*phys_BW*surfaceRatio_ILL4*influx_factor_ill4/(drug_Km_influx+VILL4.X_ILL4_DISS/VILL4/drug_molar_mass))*VILL4.X_ILL4_DISS/VILL4*
179	VillousILL4.Villous_ILL4 -> Liver.Liver_drug
	*switch_liverFlag*VillousILL4.Villous_ILL4*Qmuc_ILL4/VillousILL4*
180	MILL4.MEM_ILL4 -> null
	*(CLINT_metabolism*metabolism_factor_ill4*switch_SFgutmet*MILL4.MEM_ILL4*fu_mem)/MILL4*
181	Artery.Artery_drug -> VillousDUO.Villous_DUO
	*Qmuc_DUO*Artery.Artery_drug*
182	Artery.Artery_drug -> VillousJEJ1.Villous_JEJ1
	*Qmuc_JEJ1*Artery.Artery_drug*
183	Artery.Artery_drug -> VillousJEJ2.Villous_JEJ2
	*Qmuc_JEJ2*Artery.Artery_drug*
184	Artery.Artery_drug -> VillousILL2.Villous_ILL2
	*Qmuc_ILL2*Artery.Artery_drug*
185	Artery.Artery_drug -> VillousILL3.Villous_ILL3
	*Qmuc_ILL3*Artery.Artery_drug*
186	Artery.Artery_drug -> VillousILL4.Villous_ILL4
	*Qmuc_ILL4*Artery.Artery_drug*
187	STOMACH.X_STOMACH_SOLID -> STOMACH.X_STOMACH_DISS
	*KD*STOMACH.X_STOMACH_SOLID*(SOLIF_STOMACH-STOMACH.X_STOMACH_DISS/STOMACH)*
188	MDUO.MEM_DUO -> VillousDUO.Villous_DUO
	*DIFF_BASO_duo*switch_SFdiffbaso*MDUO.MEM_DUO*fu_mem/MDUO*
189	VillousDUO.Villous_DUO -> MDUO.MEM_DUO
	*DIFF_BASO_duo*switch_SFdiffbaso*VillousDUO.Villous_DUO*fu_blood/VillousDUO*
190	MDUO.MEM_DUO -> VDUO.X_DUO_DISS
	*DIFF_duo*switch_SFdiffapi*MDUO.MEM_DUO*fu_mem/MDUO*
191	MJEJ1.MEM_JEJ1 -> VillousJEJ1.Villous_JEJ1

	Reactions
	*DIFF_BASO_jej1*switch_SFdiffbaso*MJEJ1.MEM_JEJ1*fu_mem/MJEJ1*
192	VillousJEJ1.Villous_JEJ1 -> MJEJ1.MEM_JEJ1
	*DIFF_BASO_jej1*switch_SFdiffbaso*VillousJEJ1.Villous_JEJ1*fu_blood/VillousJEJ1*
193	MJEJ1.MEM_JEJ1 -> VJEJ1.X_JEJ1_DISS
	*DIFF_jej1*switch_SFdiffapi*MJEJ1.MEM_JEJ1*fu_mem/MJEJ1*
194	MJEJ2.MEM_JEJ2 -> VillousJEJ2.Villous_JEJ2
	*DIFF_BASO_jej2*switch_SFdiffbaso*MJEJ2.MEM_JEJ2*fu_mem/MJEJ2*
195	VillousJEJ2.Villous_JEJ2 -> MJEJ2.MEM_JEJ2
	*DIFF_BASO_jej2*switch_SFdiffbaso*VillousJEJ2.Villous_JEJ2*fu_blood/VillousJEJ2*
196	MJEJ2.MEM_JEJ2 -> VJEJ2.X_JEJ2_DISS
	*DIFF_jej2*switch_SFdiffapi*MJEJ2.MEM_JEJ2*fu_mem/MJEJ2*
197	MILL1.MEM_ILL1 -> VillousILL1.Villous_ILL1
	*DIFF_BASO_ill1*switch_SFdiffbaso*MILL1.MEM_ILL1*fu_mem/MILL1*
198	VillousILL1.Villous_ILL1 -> MILL1.MEM_ILL1
	*DIFF_BASO_ill1*switch_SFdiffbaso*VillousILL1.Villous_ILL1*fu_blood/VillousILL1*
199	MILL1.MEM_ILL1 -> VILL1.X_ILL1_DISS
	*DIFF_ill1*switch_SFdiffapi*MILL1.MEM_ILL1*fu_mem/MILL1*
200	VillousILL2.Villous_ILL2 -> MILL2.MEM_ILL2
	*DIFF_BASO_ill2*switch_SFdiffbaso*VillousILL2.Villous_ILL2*fu_blood/VillousILL2*
201	MILL2.MEM_ILL2 -> VillousILL2.Villous_ILL2
	*DIFF_BASO_ill2*switch_SFdiffbaso*MILL2.MEM_ILL2*fu_mem/MILL2*
202	MILL2.MEM_ILL2 -> VILL2.X_ILL2_DISS
	*DIFF_ill2*switch_SFdiffapi*MILL2.MEM_ILL2*fu_mem/MILL2*
203	VillousILL3.Villous_ILL3 -> MILL3.MEM_ILL3
	*DIFF_BASO_ill3*switch_SFdiffbaso*VillousILL3.Villous_ILL3*fu_blood/VillousILL3*
204	MILL3.MEM_ILL3 -> VillousILL3.Villous_ILL3
	*DIFF_BASO_ill3*switch_SFdiffbaso*MILL3.MEM_ILL3*fu_mem/MILL3*
205	MILL3.MEM_ILL3 -> VILL3.X_ILL3_DISS
	*DIFF_ill3*switch_SFdiffapi*MILL3.MEM_ILL3*fu_mem/MILL3*
206	MILL4.MEM_ILL4 -> VILL4.X_ILL4_DISS
	*DIFF_ill4*switch_SFdiffapi*MILL4.MEM_ILL4*fu_mem/MILL4*
207	VillousILL4.Villous_ILL4 -> MILL4.MEM_ILL4
	*DIFF_BASO_ill4*switch_SFdiffbaso*VillousILL4.Villous_ILL4*fu_blood/VillousILL4*
208	MILL4.MEM_ILL4 -> VillousILL4.Villous_ILL4
	*DIFF_BASO_ill4*switch_SFdiffbaso*MILL4.MEM_ILL4*fu_mem/MILL4*
209	Liver.Liver_drug -> Main_compartment.Bile_drug
	*switch_liverFlag*k_liver_bile*Liver.Liver_drug*
210	Liver.Liver_drug -> Metabolites.Metabolites_drug

	Reactions
	*switch_liverFlag*k_liver_metabolites*Liver.Liver_drug*
211	Artery.Artery_drug -> Serosa.Serosa_drug
	*k_artery_serosa*Artery.Artery_drug*
212	Serosa.Serosa_drug -> Liver.Liver_drug
	*switch_liverFlag*k_serosa_liver*Serosa.Serosa_drug*
213	VillousDUO.Villous_DUO -> MDUO.MEM_DUO
	*CLINT_influx_baso_DUO*influx_factor_duo_baso*switch_SFinflux*VillousDUO.Villous_DUO/VillousDUO*
214	VillousJEJ1.Villous_JEJ1 -> MJEJ1.MEM_JEJ1
	*CLINT_influx_baso_JEJ1*influx_factor_jej1_baso*switch_SFinflux*VillousJEJ1.Villous_JEJ1/VillousJEJ1*
215	VillousJEJ2.Villous_JEJ2 -> MJEJ2.MEM_JEJ2
	*CLINT_influx_baso_JEJ2*influx_factor_jej2_baso*switch_SFinflux*VillousJEJ2.Villous_JEJ2/VillousJEJ2*
216	VillousILL1.Villous_ILL1 -> MILL1.MEM_ILL1
	*CLINT_influx_baso_ILL1*influx_factor_ill1_baso*switch_SFinflux*VillousILL1.Villous_ILL1/VillousILL1*
217	VillousILL2.Villous_ILL2 -> MILL2.MEM_ILL2
	*CLINT_influx_baso_ILL2*influx_factor_ill2_baso*switch_SFinflux*VillousILL2.Villous_ILL2/VillousILL2*
218	VillousILL3.Villous_ILL3 -> MILL3.MEM_ILL3
	*CLINT_influx_baso_ILL3*influx_factor_ill3_baso*switch_SFinflux*VillousILL3.Villous_ILL3/VillousILL3*
219	VillousILL4.Villous_ILL4 -> MILL4.MEM_ILL4
	*CLINT_influx_baso_ILL4*influx_factor_ill4_baso*switch_SFinflux*VillousILL4.Villous_ILL4/VillousILL4*
220	Spleen.Spleen_drug -> Liver_EC_S1.Liver_EC_S1_drug
	*(1-switch_liverFlag)*k_spleen_liver*Spleen.Spleen_drug*
221	Artery.Artery_drug -> Liver_EC_S1.Liver_EC_S1_drug
	*(1-switch_liverFlag)*k_artery_liver*Artery.Artery_drug*
222	VillousDUO.Villous_DUO -> Liver_EC_S1.Liver_EC_S1_drug
	*(1-switch_liverFlag)*VillousDUO.Villous_DUO*Qmuc_DUO/VillousDUO*
223	VillousJEJ1.Villous_JEJ1 -> Liver_EC_S1.Liver_EC_S1_drug
	*(1-switch_liverFlag)*VillousJEJ1.Villous_JEJ1*Qmuc_JEJ1/VillousJEJ1*
224	VillousJEJ2.Villous_JEJ2 -> Liver_EC_S1.Liver_EC_S1_drug
	*(1-switch_liverFlag)*VillousJEJ2.Villous_JEJ2*Qmuc_JEJ2/VillousJEJ2*
225	VillousILL1.Villous_ILL1 -> Liver_EC_S1.Liver_EC_S1_drug
	*(1-switch_liverFlag)*VillousILL1.Villous_ILL1*Qmuc_ILL1/VillousILL1*
226	VillousILL2.Villous_ILL2 -> Liver_EC_S1.Liver_EC_S1_drug
	*(1-switch_liverFlag)*VillousILL2.Villous_ILL2*Qmuc_ILL2/VillousILL2*
227	VillousILL3.Villous_ILL3 -> Liver_EC_S1.Liver_EC_S1_drug
	*(1-switch_liverFlag)*VillousILL3.Villous_ILL3*Qmuc_ILL3/VillousILL3*
228	VillousILL4.Villous_ILL4 -> Liver_EC_S1.Liver_EC_S1_drug
	*(1-switch_liverFlag)*VillousILL4.Villous_ILL4*Qmuc_ILL4/VillousILL4*
229	Serosa.Serosa_drug -> Liver_EC_S1.Liver_EC_S1_drug

	Reactions
	*(1-switch_liverFlag)*k_serosa_liver*Serosa.Serosa_drug*
230	Liver_EC_S5.Liver_EC_S5_drug -> Venous.Venous_drug
	*(1-switch_liverFlag)*k_Liver_EC_S5_Venous*Liver_EC_S5.Liver_EC_S5_drug*
231	STOMACH_1.X_STOMACH_SOLID_1 -> VDUO_1.X_DUO_SOLID_1
	STOMACH_1.X_STOMACH_SOLID_1/TSTOMACH
232	STOMACH_1.X_STOMACH_DISS_1 -> VDUO_1.X_DUO_DISS_1
	STOMACH_1.X_STOMACH_DISS_1/TSTOMACH
233	VDUO_1.X_DUO_SOLID_1 -> VJEJ1_1.X_JEJ1_SOLID_1
	VDUO_1.X_DUO_SOLID_1/TDUO
234	VDUO_1.X_DUO_SOLID_1 -> VDUO_1.X_DUO_DISS_1
	*KD_1*VDUO_1.X_DUO_SOLID_1*(SOLIF_DUO_1-VDUO_1.X_DUO_DISS_1/VDUO_1)*
235	VDUO_1.X_DUO_DISS_1 -> VJEJ1_1.X_JEJ1_DISS_1
	VDUO_1.X_DUO_DISS_1/TDUO
236	VDUO_1.X_DUO_DISS_1 -> MDUO_1.MEM_DUO_1
	*(DIFF_duo_1*NI_DUO_1*switch_SFdiffapi_1*VDUO_1.X_DUO_DISS_1)/VDUO_1*
237	MDUO_1.MEM_DUO_1 -> VDUO_1.X_DUO_DISS_1
	*((switchVmax_efflux_1==zero_1)*efflux_inhib_duo*CLINT_efflux_DUO_1*efflux_factor_duo*switch_SFefflux_1+efflux_inhib_duo*switchVmax_efflux_1*phys_Normalized_ESA*phys_BW*surfaceRatio_DUO*efflux_factor_duo*switch_SFefflux_1/(drug_Km_efflux_1+MDUO_1.MEM_DUO_1*fu_mem_1/MDUO_1/drug_molar_mass_1))*MDUO_1.MEM_DUO_1*fu_mem_1/MDUO_1*
238	VDUO_1.X_DUO_DISS_1 -> MDUO_1.MEM_DUO_1
	*((switchVmax_influx_1==zero)*CLINT_influx_DUO_1*influx_factor_duo*switch_SFinflux_1+switchVmax_influx_1*phys_Normalized_ESA*phys_BW*surfaceRatio_DUO*influx_factor_duo/(drug_Km_influx_1+VDUO_1.X_DUO_DISS_1/VDUO_1/drug_molar_mass_1))*VDUO_1.X_DUO_DISS_1/VDUO_1*
239	MDUO_1.MEM_DUO_1 -> null
	*(CLINT_metabolism_1*metabolism_factor_duo_1*switch_SFgutmet_1*MDUO_1.MEM_DUO_1*fu_mem_1)/MDUO_1*
240	VJEJ1_1.X_JEJ1_SOLID_1 -> VJEJ2_1.X_JEJ2_SOLID_1
	VJEJ1_1.X_JEJ1_SOLID_1/TJEJ1
241	VJEJ1_1.X_JEJ1_SOLID_1 -> VJEJ1_1.X_JEJ1_DISS_1
	*KD_1*VJEJ1_1.X_JEJ1_SOLID_1*(SOLIF_JEJ1_1-VJEJ1_1.X_JEJ1_DISS_1/VJEJ1_1)*
242	VJEJ1_1.X_JEJ1_DISS_1 -> VJEJ2_1.X_JEJ2_DISS_1
	VJEJ1_1.X_JEJ1_DISS_1/TJEJ1
243	VJEJ1_1.X_JEJ1_DISS_1 -> MJEJ1_1.MEM_JEJ1_1
	*(DIFF_jej1_1*NI_JEJ1_1*switch_SFdiffapi_1*VJEJ1_1.X_JEJ1_DISS_1)/VJEJ1_1*
244	MJEJ1_1.MEM_JEJ1_1 -> VJEJ1_1.X_JEJ1_DISS_1
	*((switchVmax_efflux_1==zero)*efflux_inhib_jej1*CLINT_efflux_JEJ1_1*efflux_factor_jej1*switch_SFefflux_1+efflux_inhib_jej1*switchVmax_efflux_1*phys_Normalized_ESA*phys_BW*surfaceRatio_JEJ1*efflux_factor_jej1*switch_SFefflux_1/(drug_Km_efflux_1+MJEJ1_1.MEM_JEJ1_1*fu_mem_1/MJEJ1_1/drug_molar_mass_1))*MJEJ1_1.MEM_JEJ1_1*fu_mem_1/MJEJ1_1*
245	VJEJ1_1.X_JEJ1_DISS_1 -> MJEJ1_1.MEM_JEJ1_1

	**Reactions**
	*((switchVmax_influx_1==zero)*CLINT_influx_JEJ1_1*influx_factor_jej1*switch_SFinflux_1+switchVmax_influx_1*phys_Normalized_ESA*phys_BW*surfaceRatio_JEJ1*influx_factor_jej1/(drug_Km_influx_1+VJEJ1_1.X_JEJ1_DISS_1/VJEJ1_1/drug_molar_mass_1))*VJEJ1_1.X_JEJ1_DISS_1/VJEJ1_1*
246	MJEJ1_1.MEM_JEJ1_1 -> null
	*(CLINT_metabolism_1*metabolism_factor_jej1_1*switch_SFgutmet_1*MJEJ1_1.MEM_JEJ1_1*fu_mem_1)/MJEJ1_1*
247	VJEJ2_1.X_JEJ2_SOLID_1 -> VILL1_1.X_ILL1_SOLID_1
	VJEJ2_1.X_JEJ2_SOLID_1/TJEJ2
248	VJEJ2_1.X_JEJ2_SOLID_1 -> VJEJ2_1.X_JEJ2_DISS_1
	*KD_1*VJEJ2_1.X_JEJ2_SOLID_1*(SOLIF_JEJ2_1-VJEJ2_1.X_JEJ2_DISS_1/VJEJ2_1)*
249	VJEJ2_1.X_JEJ2_DISS_1 -> VILL1_1.X_ILL1_DISS_1
	VJEJ2_1.X_JEJ2_DISS_1/TJEJ2
250	VJEJ2_1.X_JEJ2_DISS_1 -> MJEJ2_1.MEM_JEJ2_1
	*(DIFF_jej2_1*NI_JEJ2_1*switch_SFdiffapi_1*VJEJ2_1.X_JEJ2_DISS_1)/VJEJ2_1*
251	MJEJ2_1.MEM_JEJ2_1 -> VJEJ2_1.X_JEJ2_DISS_1
	*((switchVmax_efflux_1==zero)*efflux_inhib_jej2*CLINT_efflux_JEJ2_1*efflux_factor_jej2*switch_SFefflux_1+efflux_inhib_jej2*switchVmax_efflux_1*phys_Normalized_ESA*phys_BW*surfaceRatio_JEJ2*efflux_factor_jej2*switch_SFefflux_1/(drug_Km_efflux_1+MJEJ2_1.MEM_JEJ2_1*fu_mem_1/MJEJ2_1/drug_molar_mass_1))*MJEJ2_1.MEM_JEJ2_1*fu_mem_1/MJEJ2_1*
252	VJEJ2_1.X_JEJ2_DISS_1 -> MJEJ2_1.MEM_JEJ2_1
	*((switchVmax_influx_1==zero)*CLINT_influx_JEJ2_1*influx_factor_jej2*switch_SFinflux_1+switchVmax_influx_1*phys_Normalized_ESA*phys_BW*surfaceRatio_JEJ2*influx_factor_jej2/(drug_Km_influx_1+VJEJ2_1.X_JEJ2_DISS_1/VJEJ2_1/drug_molar_mass_1))*VJEJ2_1.X_JEJ2_DISS_1/VJEJ2_1*
253	MJEJ2_1.MEM_JEJ2_1 -> null
	*(CLINT_metabolism_1*metabolism_factor_jej2_1*switch_SFgutmet_1*MJEJ2_1.MEM_JEJ2_1*fu_mem_1)/MJEJ2_1*
254	VILL1_1.X_ILL1_SOLID_1 -> VILL2_1.X_ILL2_SOLID_1
	VILL1_1.X_ILL1_SOLID_1/TILL1
255	VILL1_1.X_ILL1_SOLID_1 -> VILL1_1.X_ILL1_DISS_1
	*KD_1*VILL1_1.X_ILL1_SOLID_1*(SOLIF_ILL1_1-VILL1_1.X_ILL1_DISS_1/VILL1_1)*
256	VILL1_1.X_ILL1_DISS_1 -> VILL2_1.X_ILL2_DISS_1
	VILL1_1.X_ILL1_DISS_1/TILL1
257	VILL1_1.X_ILL1_DISS_1 -> MILL1_1.MEM_ILL1_1
	*(DIFF_ill1_1*NI_ILL1_1*switch_SFdiffapi_1*VILL1_1.X_ILL1_DISS_1)/VILL1_1*
258	MILL1_1.MEM_ILL1_1 -> VILL1_1.X_ILL1_DISS_1
	*((switchVmax_efflux_1==zero)*efflux_inhib_ill1*CLINT_efflux_ILL1_1*efflux_factor_ill1*switch_SFefflux_1+efflux_inhib_ill1*switchVmax_efflux_1*phys_Normalized_ESA*phys_BW*surfaceRatio_ILL1*efflux_factor_ill1*switch_SFefflux_1/(drug_Km_efflux_1+MILL1_1.MEM_ILL1_1*fu_mem_1/MILL1_1/drug_molar_mass_1))*MILL1_1.MEM_ILL1_1*fu_mem_1/MILL1_1*
259	VILL1_1.X_ILL1_DISS_1 -> MILL1_1.MEM_ILL1_1
	*((switchVmax_influx_1==zero)*CLINT_influx_ILL1_1*influx_factor_ill1*switch_SFinflux_1+switchVmax_influx_1*phys_Normalized_ESA*phys_BW*surfaceRatio_ILL1*influx_factor_ill1/(drug_Km_influx_1+VILL1_1.X_ILL1_DISS_1/VILL1_1/drug_molar_mass_1))*VILL1_1.X_ILL1_DISS_1/VILL1_1*

	Reactions
260	MILL1_1.MEM_ILL1_1 -> null
	*(CLINT_metabolism_1*metabolism_factor_ill1_1*switch_SFgutmet_1*MILL1_1.MEM_ILL1_1*fu_mem_1)/MILL1_1*
261	VILL2_1.X_ILL2_SOLID_1 -> VILL3_1.X_ILL3_SOLID_1
	VILL2_1.X_ILL2_SOLID_1/TILL2
262	VILL2_1.X_ILL2_SOLID_1 -> VILL2_1.X_ILL2_DISS_1
	*KD_1*VILL2_1.X_ILL2_SOLID_1*(SOLIF_ILL2_1-VILL2_1.X_ILL2_DISS_1/VILL2_1)*
263	VILL2_1.X_ILL2_DISS_1 -> VILL3_1.X_ILL3_DISS_1
	VILL2_1.X_ILL2_DISS_1/TILL2
264	VILL2_1.X_ILL2_DISS_1 -> MILL2_1.MEM_ILL2_1
	*(DIFF_ill2_1*NI_ILL2_1*switch_SFdiffapi_1*VILL2_1.X_ILL2_DISS_1)/VILL2_1*
265	MILL2_1.MEM_ILL2_1 -> VILL2_1.X_ILL2_DISS_1
	*((switchVmax_efflux_1==zero)*efflux_inhib_ill2*CLINT_efflux_ILL2_1*efflux_factor_ill2*switch_SFefflux_1+efflux_inhib_ill2*switchVmax_efflux_1*phys_Normalized_ESA*phys_BW*surfaceRatio_ILL2*efflux_factor_ill2*switch_SFefflux_1/(drug_Km_efflux_1+MILL2_1.MEM_ILL2_1*fu_mem_1/MILL2_1/drug_molar_mass_1))*MILL2_1.MEM_ILL2_1*fu_mem_1/MILL2_1*
266	VILL2_1.X_ILL2_DISS_1 -> MILL2_1.MEM_ILL2_1
	*((switchVmax_influx_1==zero)*CLINT_influx_ILL2_1*influx_factor_ill2*switch_SFinflux_1+switchVmax_influx_1*phys_Normalized_ESA*phys_BW*surfaceRatio_ILL2*influx_factor_ill2/(drug_Km_influx_1+VILL2_1.X_ILL2_DISS_1/VILL2_1/drug_molar_mass_1))*VILL2_1.X_ILL2_DISS_1/VILL2_1*
267	MILL2_1.MEM_ILL2_1 -> null
	*(CLINT_metabolism_1*metabolism_factor_ill2_1*switch_SFgutmet_1*MILL2_1.MEM_ILL2_1*fu_mem_1)/MILL2_1*
268	VILL3_1.X_ILL3_SOLID_1 -> VILL4_1.X_ILL4_SOLID_1
	VILL3_1.X_ILL3_SOLID_1/TILL3
269	VILL3_1.X_ILL3_SOLID_1 -> VILL3_1.X_ILL3_DISS_1
	*KD_1*VILL3_1.X_ILL3_SOLID_1*(SOLIF_ILL3_1-VILL3_1.X_ILL3_DISS_1/VILL3_1)*
270	VILL3_1.X_ILL3_DISS_1 -> VILL4_1.X_ILL4_DISS_1
	VILL3_1.X_ILL3_DISS_1/TILL3
271	VILL3_1.X_ILL3_DISS_1 -> MILL3_1.MEM_ILL3_1
	*(DIFF_ill3_1*NI_ILL3_1*switch_SFdiffapi_1*VILL3_1.X_ILL3_DISS_1)/VILL3_1*
272	MILL3_1.MEM_ILL3_1 -> VILL3_1.X_ILL3_DISS_1
	*((switchVmax_efflux_1==zero)*efflux_inhib_ill3*CLINT_efflux_ILL3_1*efflux_factor_ill3*switch_SFefflux_1+efflux_inhib_ill3*switchVmax_efflux_1*phys_Normalized_ESA*phys_BW*surfaceRatio_ILL3*efflux_factor_ill3*switch_SFefflux_1/(drug_Km_efflux_1+MILL3_1.MEM_ILL3_1*fu_mem_1/MILL3_1/drug_molar_mass_1))*MILL3_1.MEM_ILL3_1*fu_mem_1/MILL3_1*
273	VILL3_1.X_ILL3_DISS_1 -> MILL3_1.MEM_ILL3_1
	*((switchVmax_influx_1==zero)*CLINT_influx_ILL3_1*influx_factor_ill3*switch_SFinflux_1+switchVmax_influx_1*phys_Normalized_ESA*phys_BW*surfaceRatio_ILL3*influx_factor_ill3/(drug_Km_influx_1+VILL3_1.X_ILL3_DISS_1/VILL3_1/drug_molar_mass_1))*VILL3_1.X_ILL3_DISS_1/VILL3_1*
274	MILL3_1.MEM_ILL3_1 -> null

	Reactions
	*(CLINT_metabolism_1*metabolism_factor_ill3_1*switch_SFgutmet_1*MILL3_1.MEM_ILL3_1*fu_mem_1)/MILL3_1*
275	VILL4_1.X_ILL4_SOLID_1 -> Colon_1.X_CECUM_SOLID_1
	VILL4_1.X_ILL4_SOLID_1/TILL4
276	VILL4_1.X_ILL4_SOLID_1 -> VILL4_1.X_ILL4_DISS_1
	*KD_1*VILL4_1.X_ILL4_SOLID_1*(SOLIF_ILL4_1-VILL4_1.X_ILL4_DISS_1/VILL4_1)*
277	VILL4_1.X_ILL4_DISS_1 -> Colon_1.X_CECUM_DISS_1
	VILL4_1.X_ILL4_DISS_1/TILL4
278	VILL4_1.X_ILL4_DISS_1 -> MILL4_1.MEM_ILL4_1
	*(DIFF_ill4_1*NI_ILL4_1*switch_SFdiffapi_1*VILL4_1.X_ILL4_DISS_1)/VILL4_1*
279	MILL4_1.MEM_ILL4_1 -> VILL4_1.X_ILL4_DISS_1
	*((switchVmax_efflux_1==zero)*efflux_inhib_ill4*CLINT_efflux_ILL4_1*efflux_factor_ill4*switch_SFefflux_1+efflux_inhib_ill4*switchVmax_efflux_1*phys_Normalized_ESA*phys_BW*surfaceRatio_ILL4*efflux_factor_ill4*switch_SFefflux_1/(drug_Km_efflux_1+MILL4_1.MEM_ILL4_1*fu_mem_1/MILL4_1/drug_molar_mass_1))*MILL4_1.MEM_ILL4_1*fu_mem_1/MILL4_1*
280	VILL4_1.X_ILL4_DISS_1 -> MILL4_1.MEM_ILL4_1
	*((switchVmax_influx_1==zero)*CLINT_influx_ILL4_1*influx_factor_ill4*switch_SFinflux_1+switchVmax_influx_1*phys_Normalized_ESA*phys_BW*surfaceRatio_ILL4*influx_factor_ill4/(drug_Km_influx_1+VILL4_1.X_ILL4_DISS_1/VILL4_1/drug_molar_mass_1))*VILL4_1.X_ILL4_DISS_1/VILL4_1*
281	MILL4_1.MEM_ILL4_1 -> null
	*(CLINT_metabolism_1*metabolism_factor_ill4_1*switch_SFgutmet_1*MILL4_1.MEM_ILL4_1*fu_mem_1)/MILL4_1*
282	STOMACH_1.X_STOMACH_SOLID_1 -> STOMACH_1.X_STOMACH_DISS_1
	*KD_1*STOMACH_1.X_STOMACH_SOLID_1*(SOLIF_STOMACH_1-STOMACH_1.X_STOMACH_DISS_1/STOMACH_1)*
283	MDUO_1.MEM_DUO_1 -> VillousDUO_1.Villous_DUO_1
	*DIFF_BASO_duo_1*switch_SFdiffbaso_1*MDUO_1.MEM_DUO_1*fu_mem_1/MDUO_1*
284	VillousDUO_1.Villous_DUO_1 -> MDUO_1.MEM_DUO_1
	*DIFF_BASO_duo_1*switch_SFdiffbaso_1*VillousDUO_1.Villous_DUO_1*fu_blood_1/VillousDUO_1*
285	MDUO_1.MEM_DUO_1 -> VDUO_1.X_DUO_DISS_1
	*DIFF_duo_1*switch_SFdiffapi_1*MDUO_1.MEM_DUO_1*fu_mem_1/MDUO_1*
286	MJEJ1_1.MEM_JEJ1_1 -> VillousJEJ1_1.Villous_JEJ1_1
	*DIFF_BASO_jej1_1*switch_SFdiffbaso_1*MJEJ1_1.MEM_JEJ1_1*fu_mem_1/MJEJ1_1*
287	VillousJEJ1_1.Villous_JEJ1_1 -> MJEJ1_1.MEM_JEJ1_1
	*DIFF_BASO_jej1_1*switch_SFdiffbaso_1*VillousJEJ1_1.Villous_JEJ1_1*fu_blood_1/VillousJEJ1_1*
288	MJEJ1_1.MEM_JEJ1_1 -> VJEJ1_1.X_JEJ1_DISS_1
	*DIFF_jej1_1*switch_SFdiffapi_1*MJEJ1_1.MEM_JEJ1_1*fu_mem_1/MJEJ1_1*
289	MJEJ2_1.MEM_JEJ2_1 -> VillousJEJ2_1.Villous_JEJ2_1
	*DIFF_BASO_jej2_1*switch_SFdiffbaso_1*MJEJ2_1.MEM_JEJ2_1*fu_mem_1/MJEJ2_1*
290	VillousJEJ2_1.Villous_JEJ2_1 -> MJEJ2_1.MEM_JEJ2_1

	Reactions
	*DIFF_BASO_jej2_1*switch_SFdiffbaso_1*VillousJEJ2_1.Villous_JEJ2_1*fu_blood_1/VillousJEJ2_1*
291	MJEJ2_1.MEM_JEJ2_1 -> VJEJ2_1.X_JEJ2_DISS_1
	*DIFF_jej2_1*switch_SFdiffapi_1*MJEJ2_1.MEM_JEJ2_1*fu_mem_1/MJEJ2_1*
292	MILL1_1.MEM_ILL1_1 -> VillousILL1_1.Villous_ILL1_1
	*DIFF_BASO_ill1_1*switch_SFdiffbaso_1*MILL1_1.MEM_ILL1_1*fu_mem_1/MILL1_1*
293	VillousILL1_1.Villous_ILL1_1 -> MILL1_1.MEM_ILL1_1
	*DIFF_BASO_ill1_1*switch_SFdiffbaso_1*VillousILL1_1.Villous_ILL1_1*fu_blood_1/VillousILL1_1*
294	MILL1_1.MEM_ILL1_1 -> VILL1_1.X_ILL1_DISS_1
	*DIFF_ill1_1*switch_SFdiffapi_1*MILL1_1.MEM_ILL1_1*fu_mem_1/MILL1_1*
295	VillousILL2_1.Villous_ILL2_1 -> MILL2_1.MEM_ILL2_1
	*DIFF_BASO_ill2_1*switch_SFdiffbaso_1*VillousILL2_1.Villous_ILL2_1*fu_blood_1/VillousILL2_1*
296	MILL2_1.MEM_ILL2_1 -> VillousILL2_1.Villous_ILL2_1
	*DIFF_BASO_ill2_1*switch_SFdiffbaso_1*MILL2_1.MEM_ILL2_1*fu_mem_1/MILL2_1*
297	MILL2_1.MEM_ILL2_1 -> VILL2_1.X_ILL2_DISS_1
	*DIFF_ill2_1*switch_SFdiffapi_1*MILL2_1.MEM_ILL2_1*fu_mem_1/MILL2_1*
298	VillousILL3_1.Villous_ILL3_1 -> MILL3_1.MEM_ILL3_1
	*DIFF_BASO_ill3_1*switch_SFdiffbaso_1*VillousILL3_1.Villous_ILL3_1*fu_blood_1/VillousILL3_1*
299	MILL3_1.MEM_ILL3_1 -> VillousILL3_1.Villous_ILL3_1
	*DIFF_BASO_ill3_1*switch_SFdiffbaso_1*MILL3_1.MEM_ILL3_1*fu_mem_1/MILL3_1*
300	MILL3_1.MEM_ILL3_1 -> VILL3_1.X_ILL3_DISS_1
	*DIFF_ill3_1*switch_SFdiffapi_1*MILL3_1.MEM_ILL3_1*fu_mem_1/MILL3_1*
301	MILL4_1.MEM_ILL4_1 -> VILL4_1.X_ILL4_DISS_1
	*DIFF_ill4_1*switch_SFdiffapi_1*MILL4_1.MEM_ILL4_1*fu_mem_1/MILL4_1*
302	VillousILL4_1.Villous_ILL4_1 -> MILL4_1.MEM_ILL4_1
	*DIFF_BASO_ill4_1*switch_SFdiffbaso_1*VillousILL4_1.Villous_ILL4_1*fu_blood_1/VillousILL4_1*
303	MILL4_1.MEM_ILL4_1 -> VillousILL4_1.Villous_ILL4_1
	*DIFF_BASO_ill4_1*switch_SFdiffbaso_1*MILL4_1.MEM_ILL4_1*fu_mem_1/MILL4_1*
304	VillousDUO_1.Villous_DUO_1 -> MDUO_1.MEM_DUO_1
	*CLINT_influx_baso_DUO_1*influx_factor_duo_baso*switch_SFinflux_1*VillousDUO_1.Villous_DUO_1/VillousDUO_1*
305	VillousJEJ1_1.Villous_JEJ1_1 -> MJEJ1_1.MEM_JEJ1_1
	*CLINT_influx_baso_JEJ1_1*influx_factor_jej1_baso*switch_SFinflux_1*VillousJEJ1_1.Villous_JEJ1_1/VillousJEJ1_1*
306	VillousJEJ2_1.Villous_JEJ2_1 -> MJEJ2_1.MEM_JEJ2_1
	*CLINT_influx_baso_JEJ2_1*influx_factor_jej2_baso*switch_SFinflux_1*VillousJEJ2_1.Villous_JEJ2_1/VillousJEJ2_1*
307	VillousILL1_1.Villous_ILL1_1 -> MILL1_1.MEM_ILL1_1
	*CLINT_influx_baso_ILL1_1*influx_factor_ill1_baso*switch_SFinflux_1*VillousILL1_1.Villous_ILL1_1/VillousILL1_1*

	Reactions
308	VillousILL2_1.Villous_ILL2_1 -> MILL2_1.MEM_ILL2_1
	*CLINT_influx_baso_ILL2_1*influx_factor_ill2_baso*switch_SFinflux_1*VillousILL2_1.Villous_ILL2_1/VillousILL2_1*
309	VillousILL3_1.Villous_ILL3_1 -> MILL3_1.MEM_ILL3_1
	*CLINT_influx_baso_ILL3_1*influx_factor_ill3_baso*switch_SFinflux_1*VillousILL3_1.Villous_ILL3_1/VillousILL3_1*
310	VillousILL4_1.Villous_ILL4_1 -> MILL4_1.MEM_ILL4_1
	*CLINT_influx_baso_ILL4_1*influx_factor_ill4_baso*switch_SFinflux_1*VillousILL4_1.Villous_ILL4_1/VillousILL4_1*
311	Artery_1.Artery_drug_1 -> VillousDUO_1.Villous_DUO_1
	*Qmuc_DUO_1*Artery_1.Artery_drug_1*
312	Artery_1.Artery_drug_1 -> VillousJEJ1_1.Villous_JEJ1_1
	*Qmuc_JEJ1_1*Artery_1.Artery_drug_1*
313	Artery_1.Artery_drug_1 -> VillousJEJ2_1.Villous_JEJ2_1
	*Qmuc_JEJ2_1*Artery_1.Artery_drug_1*
314	Artery_1.Artery_drug_1 -> VillousILL1_1.Villous_ILL1_1
	*Qmuc_ILL1_1*Artery_1.Artery_drug_1*
315	Artery_1.Artery_drug_1 -> VillousILL2_1.Villous_ILL2_1
	*Qmuc_ILL2_1*Artery_1.Artery_drug_1*
316	Artery_1.Artery_drug_1 -> VillousILL3_1.Villous_ILL3_1
	*Qmuc_ILL3_1*Artery_1.Artery_drug_1*
317	Artery_1.Artery_drug_1 -> VillousILL4_1.Villous_ILL4_1
	*Qmuc_ILL4_1*Artery_1.Artery_drug_1*
318	Liver_1.Liver_drug_1 -> Venous_1.Venous_drug_1
	*switch_liverFlag_1*k_Liver_Venous_1*Liver_1.Liver_drug_1*
319	VillousDUO_1.Villous_DUO_1 -> Liver_1.Liver_drug_1
	*switch_liverFlag_1*VillousDUO_1.Villous_DUO_1*Qmuc_DUO_1/VillousDUO_1*
320	VillousJEJ1_1.Villous_JEJ1_1 -> Liver_1.Liver_drug_1
	*switch_liverFlag_1*VillousJEJ1_1.Villous_JEJ1_1*Qmuc_JEJ1_1/VillousJEJ1_1*
321	VillousJEJ2_1.Villous_JEJ2_1 -> Liver_1.Liver_drug_1
	*switch_liverFlag_1*VillousJEJ2_1.Villous_JEJ2_1*Qmuc_JEJ2_1/VillousJEJ2_1*
322	VillousILL1_1.Villous_ILL1_1 -> Liver_1.Liver_drug_1
	*switch_liverFlag_1*VillousILL1_1.Villous_ILL1_1*Qmuc_ILL1_1/VillousILL1_1*
323	VillousILL2_1.Villous_ILL2_1 -> Liver_1.Liver_drug_1
	*switch_liverFlag_1*VillousILL2_1.Villous_ILL2_1*Qmuc_ILL2_1/VillousILL2_1*
324	VillousILL3_1.Villous_ILL3_1 -> Liver_1.Liver_drug_1
	*switch_liverFlag_1*VillousILL3_1.Villous_ILL3_1*Qmuc_ILL3_1/VillousILL3_1*
325	VillousILL4_1.Villous_ILL4_1 -> Liver_1.Liver_drug_1
	*switch_liverFlag_1*VillousILL4_1.Villous_ILL4_1*Qmuc_ILL4_1/VillousILL4_1*

	Reactions
326	VillousDUO_1.Villous_DUO_1 -> Liver_EC_S1_1.Liver_EC_S1_drug_1
	*(1-switch_liverFlag_1)*VillousDUO_1.Villous_DUO_1*Qmuc_DUO_1/VillousDUO_1*
327	VillousJEJ1_1.Villous_JEJ1_1 -> Liver_EC_S1_1.Liver_EC_S1_drug_1
	*(1-switch_liverFlag_1)*VillousJEJ1_1.Villous_JEJ1_1*Qmuc_JEJ1_1/VillousJEJ1_1*
328	VillousJEJ2_1.Villous_JEJ2_1 -> Liver_EC_S1_1.Liver_EC_S1_drug_1
	*(1-switch_liverFlag_1)*VillousJEJ2_1.Villous_JEJ2_1*Qmuc_JEJ2_1/VillousJEJ2_1*
329	VillousILL1_1.Villous_ILL1_1 -> Liver_EC_S1_1.Liver_EC_S1_drug_1
	*(1-switch_liverFlag_1)*VillousILL1_1.Villous_ILL1_1*Qmuc_ILL1_1/VillousILL1_1*
330	VillousILL2_1.Villous_ILL2_1 -> Liver_EC_S1_1.Liver_EC_S1_drug_1
	*(1-switch_liverFlag_1)*VillousILL2_1.Villous_ILL2_1*Qmuc_ILL2_1/VillousILL2_1*
331	VillousILL3_1.Villous_ILL3_1 -> Liver_EC_S1_1.Liver_EC_S1_drug_1
	*(1-switch_liverFlag_1)*VillousILL3_1.Villous_ILL3_1*Qmuc_ILL3_1/VillousILL3_1*
332	VillousILL4_1.Villous_ILL4_1 -> Liver_EC_S1_1.Liver_EC_S1_drug_1
	*(1-switch_liverFlag_1)*VillousILL4_1.Villous_ILL4_1*Qmuc_ILL4_1/VillousILL4_1*
333	Artery_1.Artery_drug_1 -> Serosa_1.Serosa_drug_1
	*k_artery_serosa_1*Artery_1.Artery_drug_1*
334	Serosa_1.Serosa_drug_1 -> Liver_1.Liver_drug_1
	*switch_liverFlag_1*k_serosa_liver_1*Serosa_1.Serosa_drug_1*
335	Serosa_1.Serosa_drug_1 -> Liver_EC_S1_1.Liver_EC_S1_drug_1
	*(1-switch_liverFlag_1)*k_serosa_liver_1*Serosa_1.Serosa_drug_1*
336	Artery_1.Artery_drug_1 -> Liver_1.Liver_drug_1
	*switch_liverFlag_1*k_artery_liver_1*Artery_1.Artery_drug_1*
337	Spleen_1.Spleen_drug_1 -> Liver_1.Liver_drug_1
	*switch_liverFlag_1*k_spleen_liver_1*Spleen_1.Spleen_drug_1*
338	Liver_1.Liver_drug_1 -> Metabolites_1.Metabolites_drug_1
	*switch_liverFlag_1*k_liver_metabolites_1*Liver_1.Liver_drug_1*
339	Main_compartment_1.Bile_drug_1 -> VDUO_1.X_DUO_DISS_1
	*k_transit_1*Main_compartment_1.Bile_drug_1*
340	Liver_1.Liver_drug_1 -> Main_compartment_1.Bile_drug_1
	*switch_liverFlag_1*k_liver_bile_1*Liver_1.Liver_drug_1*
341	MDUO.MEM_DUO -> VillousDUO.Villous_DUO
	*((switchVmax_efflux_baso==zero)*CLINT_efflux_baso_DUO*switch_SFefflux_baso*baso_efflux_factor_duo+ switchVmax_efflux_baso*baso_efflux_factor_duo*phys_Normalized_ESA_baso*phys_BW*basoSurfaceRatio _DUO*switch_SFefflux_baso/(drug_Km_efflux_baso+MEM_DUO*fu_mem/MDUO/drug_molar_mass))*MDUO .MEM_DUO*fu_mem/MDUO*
342	MJEJ1.MEM_JEJ1 -> VillousJEJ1.Villous_JEJ1
	*((switchVmax_efflux_baso==zero)*CLINT_efflux_baso_JEJ1*switch_SFefflux_baso*baso_efflux_factor_jej1+ switchVmax_efflux_baso*baso_efflux_factor_jej1*phys_Normalized_ESA_baso*phys_BW*basoSurfaceRatio_ JEJ1*switch_SFefflux_baso/(drug_Km_efflux_baso+MEM_JEJ1*fu_mem/MJEJ1/drug_molar_mass))*MJEJ1. MEM_JEJ1*fu_mem/MJEJ1*

	Reactions
343	MJEJ2.MEM_JEJ2 -> VillousJEJ2.Villous_JEJ2
	*((switchVmax_efflux_baso==zero)*CLINT_efflux_baso_JEJ2*switch_SFefflux_baso*baso_efflux_factor_jej2+switchVmax_efflux_baso*baso_efflux_factor_jej2*phys_Normalized_ESA_baso*phys_BW*basoSurfaceRatio_JEJ2*switch_SFefflux_baso/(drug_Km_efflux_baso+MEM_JEJ2*fu_mem/MJEJ2/drug_molar_mass))*MJEJ2.MEM_JEJ2*fu_mem/MJEJ2*
344	MILL1.MEM_ILL1 -> VillousILL1.Villous_ILL1
	*((switchVmax_efflux_baso==zero)*CLINT_efflux_baso_ILL1*switch_SFefflux_baso*baso_efflux_factor_ill1+switchVmax_efflux_baso*baso_efflux_factor_ill1*phys_Normalized_ESA_baso*phys_BW*basoSurfaceRatio_ILL1*switch_SFefflux_baso/(drug_Km_efflux_baso+MEM_ILL1*fu_mem/MILL1/drug_molar_mass))*MILL1.MEM_ILL1*fu_mem/MILL1*
345	MILL2.MEM_ILL2 -> VillousILL2.Villous_ILL2
	*((switchVmax_efflux_baso==zero)*CLINT_efflux_baso_ILL2*switch_SFefflux_baso*baso_efflux_factor_ill2+switchVmax_efflux_baso*baso_efflux_factor_ill2*phys_Normalized_ESA_baso*phys_BW*basoSurfaceRatio_ILL2*switch_SFefflux_baso/(drug_Km_efflux_baso+MEM_ILL2*fu_mem/MILL2/drug_molar_mass))*MILL2.MEM_ILL2*fu_mem/MILL2*
346	MILL3.MEM_ILL3 -> VillousILL3.Villous_ILL3
	*((switchVmax_efflux_baso==zero)*CLINT_efflux_baso_ILL3*switch_SFefflux_baso*baso_efflux_factor_ill3+switchVmax_efflux_baso*baso_efflux_factor_ill3*phys_Normalized_ESA_baso*phys_BW*basoSurfaceRatio_ILL3*switch_SFefflux_baso/(drug_Km_efflux_baso+MEM_ILL3*fu_mem/MILL3/drug_molar_mass))*MILL3.MEM_ILL3*fu_mem/MILL3*
347	MILL4.MEM_ILL4 -> VillousILL4.Villous_ILL4
	*((switchVmax_efflux_baso==zero)*CLINT_efflux_baso_ILL4*switch_SFefflux_baso*baso_efflux_factor_ill4+switchVmax_efflux_baso*baso_efflux_factor_ill4*phys_Normalized_ESA_baso*phys_BW*basoSurfaceRatio_ILL4*switch_SFefflux_baso/(drug_Km_efflux_baso+MEM_ILL4*fu_mem/MILL4/drug_molar_mass))*MILL4.MEM_ILL4*fu_mem/MILL4*
348	MDUO_1.MEM_DUO_1 -> VillousDUO_1.Villous_DUO_1
	*((switchVmax_efflux_baso_1==zero)*CLINT_efflux_baso_DUO_1*switch_SFefflux_baso_1*baso_efflux_factor_duo+switchVmax_efflux_baso_1*baso_efflux_factor_duo*phys_Normalized_ESA_baso*phys_BW*basoSurfaceRatio_DUO*switch_SFefflux_baso_1/(drug_Km_efflux_baso_1+MEM_DUO_1*fu_mem_1/MDUO_1/drug_molar_mass_1))*MDUO_1.MEM_DUO_1*fu_mem_1/MDUO_1*
349	MJEJ1_1.MEM_JEJ1_1 -> VillousJEJ1_1.Villous_JEJ1_1
	*((switchVmax_efflux_baso_1==zero)*CLINT_efflux_baso_JEJ1_1*switch_SFefflux_baso_1*baso_efflux_factor_jej1+switchVmax_efflux_baso_1*baso_efflux_factor_jej1*phys_Normalized_ESA_baso*phys_BW*basoSurfaceRatio_JEJ1*switch_SFefflux_baso_1/(drug_Km_efflux_baso_1+MEM_JEJ1_1*fu_mem_1/MJEJ1_1/drug_molar_mass_1))*MJEJ1_1.MEM_JEJ1_1*fu_mem_1/MJEJ1_1*
350	MJEJ2_1.MEM_JEJ2_1 -> VillousJEJ2_1.Villous_JEJ2_1
	*((switchVmax_efflux_baso_1==zero)*CLINT_efflux_baso_JEJ2_1*switch_SFefflux_baso_1*baso_efflux_factor_jej2+switchVmax_efflux_baso_1*baso_efflux_factor_jej2*phys_Normalized_ESA_baso*phys_BW*basoSurfaceRatio_JEJ2*switch_SFefflux_baso_1/(drug_Km_efflux_baso_1+MEM_JEJ2_1*fu_mem_1/MJEJ2_1/drug_molar_mass_1))*MJEJ2_1.MEM_JEJ2_1*fu_mem_1/MJEJ2_1*
351	MILL1_1.MEM_ILL1_1 -> VillousILL1_1.Villous_ILL1_1
	*((switchVmax_efflux_baso_1==zero)*CLINT_efflux_baso_ILL1_1*switch_SFefflux_baso_1*baso_efflux_factor_ill1+switchVmax_efflux_baso_1*baso_efflux_factor_ill1*phys_Normalized_ESA_baso*phys_BW*basoSurfaceRatio_ILL1*switch_SFefflux_baso_1/(drug_Km_efflux_baso_1+MEM_ILL1_1*fu_mem_1/MILL1_1/drug_molar_mass_1))*MILL1_1.MEM_ILL1_1*fu_mem_1/MILL1_1*
352	MILL2_1.MEM_ILL2_1 -> VillousILL2_1.Villous_ILL2_1
	*((switchVmax_efflux_baso_1==zero)*CLINT_efflux_baso_ILL2_1*switch_SFefflux_baso_1*baso_efflux_factor_ill2+switchVmax_efflux_baso_1*baso_efflux_factor_ill2*phys_Normalized_ESA_baso*phys_BW*basoSurfac*

	Reactions
	eRatio_ILL2*switch_SFefflux_baso_1/(drug_Km_efflux_baso_1+MEM_ILL2_1*fu_mem_1/MILL2_1/drug_molar_mass_1))*MILL2_1.MEM_ILL2_1*fu_mem_1/MILL2_1
353	MILL3_1.MEM_ILL3_1 -> VillousILL3_1.Villous_ILL3_1
	((switchVmax_efflux_baso_1==zero)*CLINT_efflux_baso_ILL3_1*switch_SFefflux_baso_1*baso_efflux_factor_ill3+switchVmax_efflux_baso_1*baso_efflux_factor_ill3*phys_Normalized_ESA_baso*phys_BW*basoSurfaceRatio_ILL3*switch_SFefflux_baso_1/(drug_Km_efflux_baso_1+MEM_ILL3_1*fu_mem_1/MILL3_1/drug_molar_mass_1))*MILL3_1.MEM_ILL3_1*fu_mem_1/MILL3_1
354	MILL4_1.MEM_ILL4_1 -> VillousILL4_1.Villous_ILL4_1
	((switchVmax_efflux_baso_1==zero)*CLINT_efflux_baso_ILL4_1*switch_SFefflux_baso_1*baso_efflux_factor_ill4+switchVmax_efflux_baso_1*baso_efflux_factor_ill4*phys_Normalized_ESA_baso*phys_BW*basoSurfaceRatio_ILL4*switch_SFefflux_baso_1/(drug_Km_efflux_baso_1+MEM_ILL4_1*fu_mem_1/MILL4_1/drug_molar_mass_1))*MILL4_1.MEM_ILL4_1*fu_mem_1/MILL4_1

Observables

	Observables	Units
1	AUCPlasma_1 = trapz(time,Plasma_total_1.Plasma_total_uM_1)	micromole/liter*hour
2	CendLiver_1 = Liver_total_1.Liver_tissue_total_uM_1(end)	micromole/liter
3	CmaxLiver_1 = max(Liver_total_1.Liver_tissue_total_uM_1)	micromole/liter
4	AUCLiver_1 = trapz(time, Liver_total_1.Liver_tissue_total_uM_1)	micromole/liter*hour
5	AUCLiver0to24_1 = trapz(time(time<24), Liver_total_1.Liver_tissue_total_uM_1(time<24))	micromole/liter*hour
6	AUCLiver72to96_1 = trapz(time(time>72&time<96), Liver_total_1.Liver_tissue_total_uM_1(time>72&time<96))	micromole/liter*hour
7	finalLiverTissuetotal = Liver_total.Liver_tissue_total_uM(end)	micromole/liter
8	maxLiverTissueTotal = max(Liver_total.Liver_tissue_total_uM)	micromole/liter
9	AUCLiver = trapz(time, Liver_total.Liver_tissue_total_uM)	micromole/liter*hour
10	AUCLiver0to24 = trapz(time(time<24), Liver_total.Liver_tissue_total_uM(time<24))	micromole/liter*hour
11	AUCLiver72to96 = trapz(time(time>72&time<96), Liver_total.Liver_tissue_total_uM(time>72&time<96))	micromole/liter*hour
12	AUCPlasma0to24 = trapz(time(time<24), Plasma_total.Plasma_total_uM(time<24))	micromole/liter*hour
13	AUCPlasma0to24_1 = trapz(time(time<24), Plasma_total_1.Plasma_total_uM_1(time<24))	micromole/liter*hour
14	AUCPlasma = trapz(time,Plasma_total.Plasma_total_uM)	micromole/liter*hour
15	CL_1 = max(drug_dose_amount_IV_1/trapz(time,Plasma_total_1.Plasma_total_drug_1))	liter/hour

	Observables	Units
16	Vss_1 = max(drug_dose_amount_IV_1*trapz(time,time.*Plasma_total_1.Plasma_total_drug_1)/trapz(time,Plasma_total_1.Plasma_total_drug_1)^2)	liter
17	AUCPlasmaTotal0to24 = trapz(time(time<24), Plasma_total_1.Plasma_total_drug_1(time<24))	nanogram/milliliter*hour
18	AUCPlasmaTotal = trapz(time, Plasma_total_1.Plasma_total_drug_1)	nanogram/milliliter*hour
19	Cmax = max(Plasma_total.Plasma_total_drug)	nanogram/milliliter
20	Tmax = max(vertcat(NaN, time(Plasma_total.Plasma_total_drug==max(Plasma_total.Plasma_total_drug))))	hour

Model Equations

ODEs

	ODEs
1	d(Bile_drug)/dt = ((1-switch_liverFlag)*k_Liver_IC_S5_Bile*Liver_IC_S5_drug) + ((1-switch_liverFlag)*k_Liver_IC_S4_Bile*Liver_IC_S4_drug) + ((1-switch_liverFlag)*k_Liver_IC_S3_Bile*Liver_IC_S3_drug) + ((1-switch_liverFlag)*k_Liver_IC_S2_Bile*Liver_IC_S2_drug) + ((1-switch_liverFlag)*k_Liver_IC_S1_Bile*Liver_IC_S1_drug) - (k_transit*Bile_drug) - (drug_k_bile_deg*Bile_drug) + (switch_liverFlag*k_liver_bile*Liver_drug)
2	d(Venous_drug)/dt = 1/Venous*((switch_liverFlag*k_Liver_Venous*Liver_drug) + (k_rest_venous*Rest_drug) + (k_bone_venous*Bone_drug) + (k_skin_venous*Skin_drug) + (k_heart_venous*Heart_drug) + (k_adipos_venous*Adipose_drug) + (k_muscle_venous*Muscle_drug) + (k_brain_venous*Brain_drug) + (k_kidney_venous*Kidney_drug) - (k_venous_lung*Venous_drug) - (k_venous_urine_CLR*Venous_drug) + (k_testes_venous*Testes_drug) - (k_venous_urine_GFR*Venous_drug) + ((1-switch_liverFlag)*k_Liver_EC_S5_Venous*Liver_EC_S5_drug))
3	d(Lung_drug)/dt = 1/Lung*((k_venous_lung*Venous_drug) - (k_lung_artery*Lung_drug))
4	d(Kidney_drug)/dt = 1/Kidney*(-(k_kidney_venous*Kidney_drug) + (k_artery_kidney*Artery_drug))
5	d(Brain_drug)/dt = 1/Brain*(-(k_brain_venous*Brain_drug) + (k_artery_brain*Artery_drug))
6	d(Muscle_drug)/dt = 1/Muscle*(-(k_muscle_venous*Muscle_drug) + (k_artery_muscle*Artery_drug))
7	d(Adipose_drug)/dt = 1/Adipose*(-(k_adipos_venous*Adipose_drug) + (k_artery_adipos*Artery_drug))
8	d(Heart_drug)/dt = 1/Heart*(-(k_heart_venous*Heart_drug) + (k_artery_heart*Artery_drug))
9	d(Skin_drug)/dt = 1/Skin*(-(k_skin_venous*Skin_drug) + (k_artery_skin*Artery_drug))
10	d(Bone_drug)/dt = 1/Bone*(-(k_bone_venous*Bone_drug) + (k_artery_bone*Artery_drug))
11	d(Rest_drug)/dt = 1/Rest*(-(k_rest_venous*Rest_drug) + (k_artery_rest*Artery_drug))
12	d(Artery_drug)/dt = 1/Artery*(-(switch_liverFlag*k_artery_liver*Artery_drug) - (Qmuc_ILL1*Artery_drug) - (k_artery_spleen*Artery_drug) - (k_artery_rest*Artery_drug) - (k_artery_bone*Artery_drug) - (k_artery_skin*Artery_drug) - (k_artery_heart*Artery_drug) - (k_artery_adipos*Artery_drug) - (k_artery_muscle*Artery_drug) - (k_artery_brain*Artery_drug) - (k_artery_kidney*Artery_drug) + (k_lung_artery*Lung_drug) - (k_artery_testes*Artery_drug) - (Qmuc_DUO*Artery_drug) - (Qmuc_JEJ1*Artery_drug) - (Qmuc_JEJ2*Artery_drug) - (Qmuc_ILL2*Artery_drug) - (Qmuc_ILL3*Artery_drug) - (Qmuc_ILL4*Artery_drug) - (k_artery_serosa*Artery_drug) - ((1-switch_liverFlag)*k_artery_liver*Artery_drug))
13	d(Spleen_drug)/dt = 1/Spleen*((k_artery_spleen*Artery_drug) - (switch_liverFlag*k_spleen_liver*Spleen_drug) - ((1-switch_liverFlag)*k_spleen_liver*Spleen_drug))

	ODEs
14	d(Liver_EC_S1_drug)/dt = 1/Liver_EC_S1*((k_Liver_IC_S1_Liver_EC_S1*Liver_IC_S1_drug) - (k_Liver_EC_S1_Liver_IC_S1*Liver_EC_S1_drug) - (k_Liver_EC_S1_Liver_EC_S2*Liver_EC_S1_drug) + (k_Liver_IC_S1_Liver_EC_S1_efflux*Liver_IC_S1_drug) + ((1-switch_liverFlag)*k_spleen_liver*Spleen_drug) + ((1-switch_liverFlag)*k_artery_liver*Artery_drug) + ((1-switch_liverFlag)*Villous_DUO*Qmuc_DUO/VillousDUO) + ((1-switch_liverFlag)*Villous_JEJ1*Qmuc_JEJ1/VillousJEJ1) + ((1-switch_liverFlag)*Villous_JEJ2*Qmuc_JEJ2/VillousJEJ2) + ((1-switch_liverFlag)*Villous_ILL1*Qmuc_ILL1/VillousILL1) + ((1-switch_liverFlag)*Villous_ILL2*Qmuc_ILL2/VillousILL2) + ((1-switch_liverFlag)*Villous_ILL3*Qmuc_ILL3/VillousILL3) + ((1-switch_liverFlag)*Villous_ILL4*Qmuc_ILL4/VillousILL4) + ((1-switch_liverFlag)*k_serosa_liver*Serosa_drug))
15	d(Liver_EC_S2_drug)/dt = 1/Liver_EC_S2*((k_Liver_IC_S2_Liver_EC_S2*Liver_IC_S2_drug) - (k_Liver_EC_S2_Liver_IC_S2*Liver_EC_S2_drug) - (k_Liver_EC_S2_Liver_EC_S3*Liver_EC_S2_drug) + (k_Liver_EC_S1_Liver_EC_S2*Liver_EC_S1_drug) + (k_Liver_IC_S2_Liver_EC_S2_efflux*Liver_IC_S2_drug))
16	d(Liver_EC_S3_drug)/dt = 1/Liver_EC_S3*((k_Liver_IC_S3_Liver__EC_S3*Liver_IC_S3_drug) - (k_Liver_EC_S3_Liver_IC_S3*Liver_EC_S3_drug) - (k_Liver_EC_S3_Liver_EC_S4*Liver_EC_S3_drug) + (k_Liver_EC_S2_Liver_EC_S3*Liver_EC_S2_drug) + (k_Liver_IC_S3_Liver_EC_S3_efflux*Liver_IC_S3_drug))
17	d(Liver_EC_S4_drug)/dt = 1/Liver_EC_S4*((k_Liver_IC_S4_Liver_EC_S4*Liver_IC_S4_drug) - (k_Liver_EC_S4_Liver_IC_S4*Liver_EC_S4_drug) - (k_Liver_EC_S4_Liver_EC_S5*Liver_EC_S4_drug) + (k_Liver_EC_S3_Liver_EC_S4*Liver_EC_S3_drug) + (k_Liver_IC_S4_Liver_EC_S4_efflux*Liver_IC_S4_drug))
18	d(Liver_EC_S5_drug)/dt = 1/Liver_EC_S5*((k_Liver_IC_S5_Liver_EC_S5*Liver_IC_S5_drug) - (k_Liver_EC_S5_Liver_IC_S5*Liver_EC_S5_drug) + (k_Liver_EC_S4_Liver_EC_S5*Liver_EC_S4_drug) + (k_Liver_IC_S5_Liver_EC_S5_efflux*Liver_IC_S5_drug) - ((1-switch_liverFlag)*k_Liver_EC_S5_Venous*Liver_EC_S5_drug))
19	d(Liver_IC_S5_drug)/dt = 1/Liver_IC_S5*(-((1-switch_liverFlag)*k_Liver_IC_S5_Bile*Liver_IC_S5_drug) - ((1-switch_liverFlag)*k_Liver_IC_S5_Metabolites*Liver_IC_S5_drug) - (k_Liver_IC_S5_Liver_EC_S5*Liver_IC_S5_drug) + (k_Liver_EC_S5_Liver_IC_S5*Liver_EC_S5_drug) - (k_Liver_IC_S5_Liver_EC_S5_efflux*Liver_IC_S5_drug))
20	d(Liver_IC_S3_drug)/dt = 1/Liver_IC_S3*(-((1-switch_liverFlag)*k_Liver_IC_S3_Metabolites*Liver_IC_S3_drug) - ((1-switch_liverFlag)*k_Liver_IC_S3_Bile*Liver_IC_S3_drug) - (k_Liver_IC_S3_Liver__EC_S3*Liver_IC_S3_drug) + (k_Liver_EC_S3_Liver_IC_S3*Liver_EC_S3_drug) - (k_Liver_IC_S3_Liver_EC_S3_efflux*Liver_IC_S3_drug))
21	d(Liver_IC_S1_drug)/dt = 1/Liver_IC_S1*(-((1-switch_liverFlag)*k_Liver_IC_S1_Metabolites*Liver_IC_S1_drug) - ((1-switch_liverFlag)*k_Liver_IC_S1_Bile*Liver_IC_S1_drug) - (k_Liver_IC_S1_Liver_EC_S1*Liver_IC_S1_drug) + (k_Liver_EC_S1_Liver_IC_S1*Liver_EC_S1_drug) - (k_Liver_IC_S1_Liver_EC_S1_efflux*Liver_IC_S1_drug))
22	d(Liver_IC_S2_drug)/dt = 1/Liver_IC_S2*(-((1-switch_liverFlag)*k_Liver_IC_S2_Metabolites*Liver_IC_S2_drug) - ((1-switch_liverFlag)*k_Liver_IC_S2_Bile*Liver_IC_S2_drug) - (k_Liver_IC_S2_Liver_EC_S2*Liver_IC_S2_drug) + (k_Liver_EC_S2_Liver_IC_S2*Liver_EC_S2_drug) - (k_Liver_IC_S2_Liver_EC_S2_efflux*Liver_IC_S2_drug))
23	d(Metabolites_drug)/dt = ((1-switch_liverFlag)*k_Liver_IC_S5_Metabolites*Liver_IC_S5_drug) + ((1-switch_liverFlag)*k_Liver_IC_S4_Metabolites*Liver_IC_S4_drug) + ((1-switch_liverFlag)*k_Liver_IC_S3_Metabolites*Liver_IC_S3_drug) + ((1-switch_liverFlag)*k_Liver_IC_S2_Metabolites*Liver_IC_S2_drug) + ((1-switch_liverFlag)*k_Liver_IC_S1_Metabolites*Liver_IC_S1_drug) + (switch_liverFlag*k_liver_metabolites*Liver_drug)
24	d(Testes_drug)/dt = 1/Testes*((k_artery_testes*Artery_drug) - (k_testes_venous*Testes_drug))

	ODEs
25	d(Urine_drug)/dt = (k_venous_urine_CLR*Venous_drug) + (k_venous_urine_GFR*Venous_drug)
26	d(Liver_IC_S4_drug)/dt = 1/Liver_IC_S4*(-((1-switch_liverFlag)*k_Liver_IC_S4_Metabolites*Liver_IC_S4_drug) - ((1-switch_liverFlag)*k_Liver_IC_S4_Bile*Liver_IC_S4_drug) - (k_Liver_IC_S4_Liver_EC_S4*Liver_IC_S4_drug) + (k_Liver_EC_S4_Liver_IC_S4*Liver_EC_S4_drug) - (k_Liver_IC_S4_Liver_EC_S4_efflux*Liver_IC_S4_drug))
27	d(X_STOMACH_SOLID)/dt = -(X_STOMACH_SOLID/TSTOMACH) - (KD*X_STOMACH_SOLID*(SOLIF_STOMACH-X_STOMACH_DISS/STOMACH))
28	d(X_STOMACH_DISS)/dt = -(X_STOMACH_DISS/TSTOMACH) + (KD*X_STOMACH_SOLID*(SOLIF_STOMACH-X_STOMACH_DISS/STOMACH))
29	d(X_DUO_SOLID)/dt = (X_STOMACH_SOLID/TSTOMACH) - (X_DUO_SOLID/TDUO) - (KD*X_DUO_SOLID*(SOLIF_DUO-X_DUO_DISS/VDUO))
30	d(X_DUO_DISS)/dt = (k_transit*Bile_drug) + (X_STOMACH_DISS/TSTOMACH) + (KD*X_DUO_SOLID*(SOLIF_DUO-X_DUO_DISS/VDUO)) - (X_DUO_DISS/TDUO) - ((DIFF_duo*NI_DUO*switch_SFdiffapi*X_DUO_DISS)/VDUO) + (((switchVmax_efflux==zero)*CLINT_efflux_DUO*efflux_factor_duo*switch_SFefflux+switchVmax_efflux*phys_Normalized_ESA*phys_BW*surfaceRatio_DUO*efflux_factor_duo*switch_SFefflux/(drug_Km_efflux+MEM_DUO*fu_mem/MDUO/drug_molar_mass))*MEM_DUO*fu_mem/MDUO) - (((switchVmax_influx==zero)*CLINT_influx_DUO*influx_factor_duo*switch_SFinflux+switchVmax_influx*phys_Normalized_ESA*phys_BW*surfaceRatio_DUO*influx_factor_duo/(drug_Km_influx+X_DUO_DISS/VDUO/drug_molar_mass))*X_DUO_DISS/VDUO) + (DIFF_duo*switch_SFdiffapi*MEM_DUO*fu_mem/MDUO)
31	d(X_JEJ1_SOLID)/dt = (X_DUO_SOLID/TDUO) - (X_JEJ1_SOLID/TJEJ1) - (KD*X_JEJ1_SOLID*(SOLIF_JEJ1-X_JEJ1_DISS/VJEJ1))
32	d(X_JEJ1_DISS)/dt = (X_DUO_DISS/TDUO) + (KD*X_JEJ1_SOLID*(SOLIF_JEJ1-X_JEJ1_DISS/VJEJ1)) - (X_JEJ1_DISS/TJEJ1) - ((DIFF_jej1*NI_JEJ1*switch_SFdiffapi*X_JEJ1_DISS)/VJEJ1) + (((switchVmax_efflux==zero)*CLINT_efflux_JEJ1*efflux_factor_jej1*switch_SFefflux+switchVmax_efflux*phys_Normalized_ESA*phys_BW*surfaceRatio_JEJ1*efflux_factor_jej1*switch_SFefflux/(drug_Km_efflux+MEM_JEJ1*fu_mem/MJEJ1/drug_molar_mass))*MEM_JEJ1*fu_mem/MJEJ1) - (((switchVmax_influx==zero)*CLINT_influx_JEJ1*influx_factor_jej1*switch_SFinflux+switchVmax_influx*phys_Normalized_ESA*phys_BW*surfaceRatio_JEJ1*influx_factor_jej1/(drug_Km_influx+X_JEJ1_DISS/VJEJ1/drug_molar_mass))*X_JEJ1_DISS/VJEJ1) + (DIFF_jej1*switch_SFdiffapi*MEM_JEJ1*fu_mem/MJEJ1)
33	d(MEM_DUO)/dt = ((DIFF_duo*NI_DUO*switch_SFdiffapi*X_DUO_DISS)/VDUO) - (((switchVmax_efflux==zero)*CLINT_efflux_DUO*efflux_factor_duo*switch_SFefflux+switchVmax_efflux*phys_Normalized_ESA*phys_BW*surfaceRatio_DUO*efflux_factor_duo*switch_SFefflux/(drug_Km_efflux+MEM_DUO*fu_mem/MDUO/drug_molar_mass))*MEM_DUO*fu_mem/MDUO) + (((switchVmax_influx==zero)*CLINT_influx_DUO*influx_factor_duo*switch_SFinflux+switchVmax_influx*phys_Normalized_ESA*phys_BW*surfaceRatio_DUO*influx_factor_duo/(drug_Km_influx+X_DUO_DISS/VDUO/drug_molar_mass))*X_DUO_DISS/VDUO) - ((CLINT_metabolism*metabolism_factor_duo*switch_SFgutmet*MEM_DUO*fu_mem)/MDUO) - (DIFF_BASO_duo*switch_SFdiffbaso*MEM_DUO*fu_mem/MDUO) + (DIFF_BASO_duo*switch_SFdiffbaso*Villous_DUO*fu_blood/VillousDUO) - (DIFF_duo*switch_SFdiffapi*MEM_DUO*fu_mem/MDUO) + (CLINT_influx_baso_DUO*influx_factor_duo_baso*switch_SFinflux*Villous_DUO/VillousDUO) - (((switchVmax_efflux_baso==zero)*CLINT_efflux_baso_DUO*switch_SFefflux_baso*baso_efflux_factor_duo+switchVmax_efflux_baso*baso_efflux_factor_duo*phys_Normalized_ESA_baso*phys_BW*basoSurfaceRatio_DUO*switch_SFefflux_baso/(drug_Km_efflux_baso+MEM_DUO*fu_mem/MDUO/drug_molar_mass))*MEM_DUO*fu_mem/MDUO)
34	d(MEM_JEJ1)/dt = ((DIFF_jej1*NI_JEJ1*switch_SFdiffapi*X_JEJ1_DISS)/VJEJ1) - (((switchVmax_efflux==zero)*CLINT_efflux_JEJ1*efflux_factor_jej1*switch_SFefflux+switchVmax_efflux*phys_Normalized_ESA*phys_BW*surfaceRatio_JEJ1*efflux_factor_jej1*switch_SFefflux/(drug_Km_efflux+MEM_JEJ1*fu_mem/MJEJ1/drug_molar_mass))*MEM_JEJ1*fu_mem/MJEJ1) + (((switchVmax_influx==zero)*CLINT_influx_JEJ1*influx_factor_jej1*switch_SFinflux+switchVmax_influx*phys_Normalized_ESA*phys_BW*surfaceRatio_JEJ1*influx_factor_jej1/(drug_Km_influx+X_JEJ1_DISS/VJEJ1/drug_molar_mass))*X_JEJ1_DISS/VJEJ1) -

	ODEs
	((CLINT_metabolism*metabolism_factor_jej1*switch_SFgutmet*MEM_JEJ1*fu_mem)/MJEJ1) - (DIFF_BASO_jej1*switch_SFdiffbaso*MEM_JEJ1*fu_mem/MJEJ1) + (DIFF_BASO_jej1*switch_SFdiffbaso*Villous_JEJ1*fu_blood/VillousJEJ1) - (DIFF_jej1*switch_SFdiffapi*MEM_JEJ1*fu_mem/MJEJ1) + (CLINT_influx_baso_JEJ1*influx_factor_jej1_baso*switch_SFinflux*Villous_JEJ1/VillousJEJ1) - (((switchVmax_efflux_baso==zero)*CLINT_efflux_baso_JEJ1*switch_SFefflux_baso*baso_efflux_factor_jej1+switchVmax_efflux_baso*baso_efflux_factor_jej1*phys_Normalized_ESA_baso*phys_BW*basoSurfaceRatio_JEJ1*switch_SFefflux_baso/(drug_Km_efflux_baso+MEM_JEJ1*fu_mem/MJEJ1/drug_molar_mass))*MEM_JEJ1*fu_mem/MJEJ1)
35	d(X_JEJ2_SOLID)/dt = (X_JEJ1_SOLID/TJEJ1) - (X_JEJ2_SOLID/TJEJ2) - (KD*X_JEJ2_SOLID*(SOLIF_JEJ2-X_JEJ2_DISS/VJEJ2))
36	d(X_JEJ2_DISS)/dt = (X_JEJ1_DISS/TJEJ1) + (KD*X_JEJ2_SOLID*(SOLIF_JEJ2-X_JEJ2_DISS/VJEJ2)) - (X_JEJ2_DISS/TJEJ2) - ((DIFF_jej2*NI_JEJ2*switch_SFdiffapi*X_JEJ2_DISS)/VJEJ2) + (((switchVmax_efflux==zero)*CLINT_efflux_JEJ2*efflux_factor_jej2*switch_SFefflux+switchVmax_efflux*phys_Normalized_ESA*phys_BW*surfaceRatio_JEJ2*efflux_factor_jej2*switch_SFefflux/(drug_Km_efflux+MEM_JEJ2*fu_mem/MJEJ2/drug_molar_mass))*MEM_JEJ2*fu_mem/MJEJ2) - (((switchVmax_influx==zero)*CLINT_influx_JEJ2*influx_factor_jej2*switch_SFinflux+switchVmax_influx*phys_Normalized_ESA*phys_BW*surfaceRatio_JEJ2*influx_factor_jej2/(drug_Km_influx+X_JEJ2_DISS/VJEJ2/drug_molar_mass))*X_JEJ2_DISS/VJEJ2) + (DIFF_jej2*switch_SFdiffapi*MEM_JEJ2*fu_mem/MJEJ2)
37	d(MEM_JEJ2)/dt = ((DIFF_jej2*NI_JEJ2*switch_SFdiffapi*X_JEJ2_DISS)/VJEJ2) - (((switchVmax_efflux==zero)*CLINT_efflux_JEJ2*efflux_factor_jej2*switch_SFefflux+switchVmax_efflux*phys_Normalized_ESA*phys_BW*surfaceRatio_JEJ2*efflux_factor_jej2*switch_SFefflux/(drug_Km_efflux+MEM_JEJ2*fu_mem/MJEJ2/drug_molar_mass))*MEM_JEJ2*fu_mem/MJEJ2) + (((switchVmax_influx==zero)*CLINT_influx_JEJ2*influx_factor_jej2*switch_SFinflux+switchVmax_influx*phys_Normalized_ESA*phys_BW*surfaceRatio_JEJ2*influx_factor_jej2/(drug_Km_influx+X_JEJ2_DISS/VJEJ2/drug_molar_mass))*X_JEJ2_DISS/VJEJ2) - ((CLINT_metabolism*metabolism_factor_jej2*switch_SFgutmet*MEM_JEJ2*fu_mem)/MJEJ2) - (DIFF_BASO_jej2*switch_SFdiffbaso*MEM_JEJ2*fu_mem/MJEJ2) + (DIFF_BASO_jej2*switch_SFdiffbaso*Villous_JEJ2*fu_blood/VillousJEJ2) - (DIFF_jej2*switch_SFdiffapi*MEM_JEJ2*fu_mem/MJEJ2) + (CLINT_influx_baso_JEJ2*influx_factor_jej2_baso*switch_SFinflux*Villous_JEJ2/VillousJEJ2) - (((switchVmax_efflux_baso==zero)*CLINT_efflux_baso_JEJ2*switch_SFefflux_baso*baso_efflux_factor_jej2+switchVmax_efflux_baso*baso_efflux_factor_jej2*phys_Normalized_ESA_baso*phys_BW*basoSurfaceRatio_JEJ2*switch_SFefflux_baso/(drug_Km_efflux_baso+MEM_JEJ2*fu_mem/MJEJ2/drug_molar_mass))*MEM_JEJ2*fu_mem/MJEJ2)
38	d(X_ILL1_SOLID)/dt = (X_JEJ2_SOLID/TJEJ2) - (X_ILL1_SOLID/TILL1) - (KD*X_ILL1_SOLID*(SOLIF_ILL1-X_ILL1_DISS/VILL1))
39	d(X_ILL1_DISS)/dt = (X_JEJ2_DISS/TJEJ2) + (KD*X_ILL1_SOLID*(SOLIF_ILL1-X_ILL1_DISS/VILL1)) - (X_ILL1_DISS/TILL1) - ((DIFF_ill1*NI_ILL1*switch_SFdiffapi*X_ILL1_DISS)/VILL1) + (((switchVmax_efflux==zero)*CLINT_efflux_ILL1*efflux_factor_ill1*switch_SFefflux+switchVmax_efflux*phys_Normalized_ESA*phys_BW*surfaceRatio_ILL1*efflux_factor_ill1*switch_SFefflux/(drug_Km_efflux+MEM_ILL1*fu_mem/MILL1/drug_molar_mass))*MEM_ILL1*fu_mem/MILL1) - (((switchVmax_influx==zero)*CLINT_influx_ILL1*influx_factor_ill1*switch_SFinflux+switchVmax_influx*phys_Normalized_ESA*phys_BW*surfaceRatio_ILL1*influx_factor_ill1/(drug_Km_influx+X_ILL1_DISS/VILL1/drug_molar_mass))*X_ILL1_DISS/VILL1) + (DIFF_ill1*switch_SFdiffapi*MEM_ILL1*fu_mem/MILL1)
40	d(MEM_ILL1)/dt = ((DIFF_ill1*NI_ILL1*switch_SFdiffapi*X_ILL1_DISS)/VILL1) - (((switchVmax_efflux==zero)*CLINT_efflux_ILL1*efflux_factor_ill1*switch_SFefflux+switchVmax_efflux*phys_Normalized_ESA*phys_BW*surfaceRatio_ILL1*efflux_factor_ill1*switch_SFefflux/(drug_Km_efflux+MEM_ILL1*fu_mem/MILL1/drug_molar_mass))*MEM_ILL1*fu_mem/MILL1) + (((switchVmax_influx==zero)*CLINT_influx_ILL1*influx_factor_ill1*switch_SFinflux+switchVmax_influx*phys_Normalized_ESA*phys_BW*surfaceRatio_ILL1*influx_factor_ill1/(drug_Km_influx+X_ILL1_DISS/VILL1/drug_molar_mass))*X_ILL1_DISS/VILL1) - ((CLINT_metabolism*metabolism_factor_ill1*switch_SFgutmet*MEM_ILL1*fu_mem)/MILL1) - (DIFF_BASO_ill1*switch_SFdiffbaso*MEM_ILL1*fu_mem/MILL1) + (DIFF_BASO_ill1*switch_SFdiffbaso*Villous_ILL1*fu_blood/VillousILL1) - (DIFF_ill1*switch_SFdiffapi*MEM_ILL1*fu_mem/MILL1) +

	ODEs
	(CLINT_influx_baso_ILL1*influx_factor_ill1_baso*switch_SFinflux*Villous_ILL1/VillousILL1) - (((switchVmax_efflux_baso==zero)*CLINT_efflux_baso_ILL1*switch_SFefflux_baso*baso_efflux_factor_ill1+switchVmax_efflux_baso*baso_efflux_factor_ill1*phys_Normalized_ESA_baso*phys_BW*basoSurfaceRatio_ILL1*switch_SFefflux_baso/(drug_Km_efflux_baso+MEM_ILL1*fu_mem/MILL1/drug_molar_mass))*MEM_ILL1*fu_mem/MILL1)
41	d(X_ILL2_SOLID)/dt = (X_ILL1_SOLID/TILL1) - (X_ILL2_SOLID/TILL2) - (KD*X_ILL2_SOLID*(SOLIF_ILL2-X_ILL2_DISS/VILL2))
42	d(X_ILL2_DISS)/dt = (X_ILL1_DISS/TILL1) + (KD*X_ILL2_SOLID*(SOLIF_ILL2-X_ILL2_DISS/VILL2)) - (X_ILL2_DISS/TILL2) - ((DIFF_ill2*NI_ILL2*switch_SFdiffapi*X_ILL2_DISS)/VILL2) + (((switchVmax_efflux==zero)*CLINT_efflux_ILL2*efflux_factor_ill2*switch_SFefflux+switchVmax_efflux*phys_Normalized_ESA*phys_BW*surfaceRatio_ILL2*efflux_factor_ill2*switch_SFefflux/(drug_Km_efflux+MEM_ILL2*fu_mem/MILL2/drug_molar_mass))*MEM_ILL2*fu_mem/MILL2) - (((switchVmax_influx==zero)*CLINT_influx_ILL2*influx_factor_ill2*switch_SFinflux+switchVmax_influx*phys_Normalized_ESA*phys_BW*surfaceRatio_ILL2*influx_factor_ill2/(drug_Km_influx+X_ILL2_DISS/VILL2/drug_molar_mass))*X_ILL2_DISS/VILL2) + (DIFF_ill2*switch_SFdiffapi*MEM_ILL2*fu_mem/MILL2)
43	d(MEM_ILL2)/dt = ((DIFF_ill2*NI_ILL2*switch_SFdiffapi*X_ILL2_DISS)/VILL2) - (((switchVmax_efflux==zero)*CLINT_efflux_ILL2*efflux_factor_ill2*switch_SFefflux+switchVmax_efflux*phys_Normalized_ESA*phys_BW*surfaceRatio_ILL2*efflux_factor_ill2*switch_SFefflux/(drug_Km_efflux+MEM_ILL2*fu_mem/MILL2/drug_molar_mass))*MEM_ILL2*fu_mem/MILL2) + (((switchVmax_influx==zero)*CLINT_influx_ILL2*influx_factor_ill2*switch_SFinflux+switchVmax_influx*phys_Normalized_ESA*phys_BW*surfaceRatio_ILL2*influx_factor_ill2/(drug_Km_influx+X_ILL2_DISS/VILL2/drug_molar_mass))*X_ILL2_DISS/VILL2) - ((CLINT_metabolism*metabolism_factor_ill2*switch_SFgutmet*MEM_ILL2*fu_mem)/MILL2) + (DIFF_BASO_ill2*switch_SFdiffbaso*Villous_ILL2*fu_blood/VillousILL2) - (DIFF_BASO_ill2*switch_SFdiffbaso*MEM_ILL2*fu_mem/MILL2) - (DIFF_ill2*switch_SFdiffapi*MEM_ILL2*fu_mem/MILL2) + (CLINT_influx_baso_ILL2*influx_factor_ill2_baso*switch_SFinflux*Villous_ILL2/VillousILL2) - (((switchVmax_efflux_baso==zero)*CLINT_efflux_baso_ILL2*switch_SFefflux_baso*baso_efflux_factor_ill2+switchVmax_efflux_baso*baso_efflux_factor_ill2*phys_Normalized_ESA_baso*phys_BW*basoSurfaceRatio_ILL2*switch_SFefflux_baso/(drug_Km_efflux_baso+MEM_ILL2*fu_mem/MILL2/drug_molar_mass))*MEM_ILL2*fu_mem/MILL2)
44	d(X_ILL3_SOLID)/dt = (X_ILL2_SOLID/TILL2) - (X_ILL3_SOLID/TILL3) - (KD*X_ILL3_SOLID*(SOLIF_ILL3-X_ILL3_DISS/VILL3))
45	d(X_ILL3_DISS)/dt = (X_ILL2_DISS/TILL2) + (KD*X_ILL3_SOLID*(SOLIF_ILL3-X_ILL3_DISS/VILL3)) - (X_ILL3_DISS/TILL3) - ((DIFF_ill3*NI_ILL3*switch_SFdiffapi*X_ILL3_DISS)/VILL3) + (((switchVmax_efflux==zero)*CLINT_efflux_ILL3*efflux_factor_ill3*switch_SFefflux+switchVmax_efflux*phys_Normalized_ESA*phys_BW*surfaceRatio_ILL3*efflux_factor_ill3*switch_SFefflux/(drug_Km_efflux+MEM_ILL3*fu_mem/MILL3/drug_molar_mass))*MEM_ILL3*fu_mem/MILL3) - (((switchVmax_influx==zero)*CLINT_influx_ILL3*influx_factor_ill3*switch_SFinflux+switchVmax_influx*phys_Normalized_ESA*phys_BW*surfaceRatio_ILL3*influx_factor_ill3/(drug_Km_influx+X_ILL3_DISS/VILL3/drug_molar_mass))*X_ILL3_DISS/VILL3) + (DIFF_ill3*switch_SFdiffapi*MEM_ILL3*fu_mem/MILL3)
46	d(MEM_ILL3)/dt = ((DIFF_ill3*NI_ILL3*switch_SFdiffapi*X_ILL3_DISS)/VILL3) - (((switchVmax_efflux==zero)*CLINT_efflux_ILL3*efflux_factor_ill3*switch_SFefflux+switchVmax_efflux*phys_Normalized_ESA*phys_BW*surfaceRatio_ILL3*efflux_factor_ill3*switch_SFefflux/(drug_Km_efflux+MEM_ILL3*fu_mem/MILL3/drug_molar_mass))*MEM_ILL3*fu_mem/MILL3) + (((switchVmax_influx==zero)*CLINT_influx_ILL3*influx_factor_ill3*switch_SFinflux+switchVmax_influx*phys_Normalized_ESA*phys_BW*surfaceRatio_ILL3*influx_factor_ill3/(drug_Km_influx+X_ILL3_DISS/VILL3/drug_molar_mass))*X_ILL3_DISS/VILL3) - ((CLINT_metabolism*metabolism_factor_ill3*switch_SFgutmet*MEM_ILL3*fu_mem)/MILL3) + (DIFF_BASO_ill3*switch_SFdiffbaso*Villous_ILL3*fu_blood/VillousILL3) - (DIFF_BASO_ill3*switch_SFdiffbaso*MEM_ILL3*fu_mem/MILL3) - (DIFF_ill3*switch_SFdiffapi*MEM_ILL3*fu_mem/MILL3) + (CLINT_influx_baso_ILL3*influx_factor_ill3_baso*switch_SFinflux*Villous_ILL3/VillousILL3) - (((switchVmax_efflux_baso==zero)*CLINT_efflux_baso_ILL3*switch_SFefflux_baso*baso_efflux_factor_ill3+switchVmax_efflux_baso*baso_efflux_factor_ill3*phys_Normalized_ESA_baso*phys_BW*basoSurfaceRatio_IL

	ODEs
	L3*switch_SFefflux_baso/(drug_Km_efflux_baso+MEM_ILL3*fu_mem/MILL3/drug_molar_mass))*MEM_ILL3*fu_mem/MILL3)
47	d(X_ILL4_SOLID)/dt = (X_ILL3_SOLID/TILL3) - (X_ILL4_SOLID/TILL4) - (KD*X_ILL4_SOLID*(SOLIF_ILL4-X_ILL4_DISS/VILL4))
48	d(X_ILL4_DISS)/dt = (X_ILL3_DISS/TILL3) + (KD*X_ILL4_SOLID*(SOLIF_ILL4-X_ILL4_DISS/VILL4)) - (X_ILL4_DISS/TILL4) - ((DIFF_ill4*NI_ILL4*switch_SFdiffapi*X_ILL4_DISS)/VILL4) + (((switchVmax_efflux==zero)*CLINT_efflux_ILL4*efflux_factor_ill4*switch_SFefflux+switchVmax_efflux*phys_Normalized_ESA*phys_BW*surfaceRatio_ILL4*efflux_factor_ill4*switch_SFefflux/(drug_Km_efflux+MEM_ILL4*fu_mem/MILL4/drug_molar_mass))*MEM_ILL4*fu_mem/MILL4) - (((switchVmax_influx==zero)*CLINT_influx_ILL4*influx_factor_ill4*switch_SFinflux+switchVmax_influx*phys_Normalized_ESA*phys_BW*surfaceRatio_ILL4*influx_factor_ill4/(drug_Km_influx+X_ILL4_DISS/VILL4/drug_molar_mass))*X_ILL4_DISS/VILL4) + (DIFF_ill4*switch_SFdiffapi*MEM_ILL4*fu_mem/MILL4)
49	d(MEM_ILL4)/dt = ((DIFF_ill4*NI_ILL4*switch_SFdiffapi*X_ILL4_DISS)/VILL4) - (((switchVmax_efflux==zero)*CLINT_efflux_ILL4*efflux_factor_ill4*switch_SFefflux+switchVmax_efflux*phys_Normalized_ESA*phys_BW*surfaceRatio_ILL4*efflux_factor_ill4*switch_SFefflux/(drug_Km_efflux+MEM_ILL4*fu_mem/MILL4/drug_molar_mass))*MEM_ILL4*fu_mem/MILL4) + (((switchVmax_influx==zero)*CLINT_influx_ILL4*influx_factor_ill4*switch_SFinflux+switchVmax_influx*phys_Normalized_ESA*phys_BW*surfaceRatio_ILL4*influx_factor_ill4/(drug_Km_influx+X_ILL4_DISS/VILL4/drug_molar_mass))*X_ILL4_DISS/VILL4) - ((CLINT_metabolism*metabolism_factor_ill4*switch_SFgutmet*MEM_ILL4*fu_mem)/MILL4) - (DIFF_ill4*switch_SFdiffapi*MEM_ILL4*fu_mem/MILL4) + (DIFF_BASO_ill4*switch_SFdiffbaso*Villous_ILL4*fu_blood/VillousILL4) - (DIFF_BASO_ill4*switch_SFdiffbaso*MEM_ILL4*fu_mem/MILL4) + (CLINT_influx_baso_ILL4*influx_factor_ill4_baso*switch_SFinflux*Villous_ILL4/VillousILL4) - (((switchVmax_efflux_baso==zero)*CLINT_efflux_baso_ILL4*switch_SFefflux_baso*baso_efflux_factor_ill4+switchVmax_efflux_baso*baso_efflux_factor_ill4*phys_Normalized_ESA_baso*phys_BW*basoSurfaceRatio_ILL4*switch_SFefflux_baso/(drug_Km_efflux_baso+MEM_ILL4*fu_mem/MILL4/drug_molar_mass))*MEM_ILL4*fu_mem/MILL4)
50	d(X_CECUM_SOLID)/dt = (X_ILL4_SOLID/TILL4)
51	d(X_CECUM_DISS)/dt = (X_ILL4_DISS/TILL4)
52	d(Villous_DUO)/dt = -(switch_liverFlag*Villous_DUO*Qmuc_DUO/VillousDUO) + (Qmuc_DUO*Artery_drug) + (DIFF_BASO_duo*switch_SFdiffbaso*MEM_DUO*fu_mem/MDUO) - (DIFF_BASO_duo*switch_SFdiffbaso*Villous_DUO*fu_blood/VillousDUO) - (CLINT_influx_baso_DUO*influx_factor_duo_baso*switch_SFinflux*Villous_DUO/VillousDUO) - ((1-switch_liverFlag)*Villous_DUO*Qmuc_DUO/VillousDUO) + (((switchVmax_efflux_baso==zero)*CLINT_efflux_baso_DUO*switch_SFefflux_baso*baso_efflux_factor_duo+switchVmax_efflux_baso*baso_efflux_factor_duo*phys_Normalized_ESA_baso*phys_BW*basoSurfaceRatio_DUO*switch_SFefflux_baso/(drug_Km_efflux_baso+MEM_DUO*fu_mem/MDUO/drug_molar_mass))*MEM_DUO*fu_mem/MDUO)
53	d(Villous_JEJ2)/dt = -(switch_liverFlag*Villous_JEJ2*Qmuc_JEJ2/VillousJEJ2) + (Qmuc_JEJ2*Artery_drug) + (DIFF_BASO_jej2*switch_SFdiffbaso*MEM_JEJ2*fu_mem/MJEJ2) - (DIFF_BASO_jej2*switch_SFdiffbaso*Villous_JEJ2*fu_blood/VillousJEJ2) - (CLINT_influx_baso_JEJ2*influx_factor_jej2_baso*switch_SFinflux*Villous_JEJ2/VillousJEJ2) - ((1-switch_liverFlag)*Villous_JEJ2*Qmuc_JEJ2/VillousJEJ2) + (((switchVmax_efflux_baso==zero)*CLINT_efflux_baso_JEJ2*switch_SFefflux_baso*baso_efflux_factor_jej2+switchVmax_efflux_baso*baso_efflux_factor_jej2*phys_Normalized_ESA_baso*phys_BW*basoSurfaceRatio_JEJ2*switch_SFefflux_baso/(drug_Km_efflux_baso+MEM_JEJ2*fu_mem/MJEJ2/drug_molar_mass))*MEM_JEJ2*fu_mem/MJEJ2)
54	d(Villous_ILL1)/dt = (Qmuc_ILL1*Artery_drug) - (switch_liverFlag*Villous_ILL1*Qmuc_ILL1/VillousILL1) + (DIFF_BASO_ill1*switch_SFdiffbaso*MEM_ILL1*fu_mem/MILL1) - (DIFF_BASO_ill1*switch_SFdiffbaso*Villous_ILL1*fu_blood/VillousILL1) - (CLINT_influx_baso_ILL1*influx_factor_ill1_baso*switch_SFinflux*Villous_ILL1/VillousILL1) - ((1-switch_liverFlag)*Villous_ILL1*Qmuc_ILL1/VillousILL1) + (((switchVmax_efflux_baso==zero)*CLINT_efflux_baso_ILL1*switch_SFefflux_baso*baso_efflux_factor_ill1+switchVmax_efflux_baso*baso_efflux_factor_ill1*phys_Normalized_ESA_baso*phys_BW*basoSurfaceRatio_IL

	ODEs
	L1*switch_SFefflux_baso/(drug_Km_efflux_baso+MEM_ILL1*fu_mem/MILL1/drug_molar_mass))*MEM_ILL1*fu_mem/MILL1)
55	d(Villous_ILL2)/dt = -(switch_liverFlag*Villous_ILL2*Qmuc_ILL2/VillousILL2) + (Qmuc_ILL2*Artery_drug) - (DIFF_BASO_ill2*switch_SFdiffbaso*Villous_ILL2*fu_blood/VillousILL2) + (DIFF_BASO_ill2*switch_SFdiffbaso*MEM_ILL2*fu_mem/MILL2) - (CLINT_influx_baso_ILL2*influx_factor_ill2_baso*switch_SFinflux*Villous_ILL2/VillousILL2) - ((1-switch_liverFlag)*Villous_ILL2*Qmuc_ILL2/VillousILL2) + (((switchVmax_efflux_baso==zero)*CLINT_efflux_baso_ILL2*switch_SFefflux_baso*baso_efflux_factor_ill2+switchVmax_efflux_baso*baso_efflux_factor_ill2*phys_Normalized_ESA_baso*phys_BW*basoSurfaceRatio_ILL2*switch_SFefflux_baso/(drug_Km_efflux_baso+MEM_ILL2*fu_mem/MILL2/drug_molar_mass))*MEM_ILL2*fu_mem/MILL2)
56	d(Villous_ILL3)/dt = -(switch_liverFlag*Villous_ILL3*Qmuc_ILL3/VillousILL3) + (Qmuc_ILL3*Artery_drug) - (DIFF_BASO_ill3*switch_SFdiffbaso*Villous_ILL3*fu_blood/VillousILL3) + (DIFF_BASO_ill3*switch_SFdiffbaso*MEM_ILL3*fu_mem/MILL3) - (CLINT_influx_baso_ILL3*influx_factor_ill3_baso*switch_SFinflux*Villous_ILL3/VillousILL3) - ((1-switch_liverFlag)*Villous_ILL3*Qmuc_ILL3/VillousILL3) + (((switchVmax_efflux_baso==zero)*CLINT_efflux_baso_ILL3*switch_SFefflux_baso*baso_efflux_factor_ill3+switchVmax_efflux_baso*baso_efflux_factor_ill3*phys_Normalized_ESA_baso*phys_BW*basoSurfaceRatio_ILL3*switch_SFefflux_baso/(drug_Km_efflux_baso+MEM_ILL3*fu_mem/MILL3/drug_molar_mass))*MEM_ILL3*fu_mem/MILL3)
57	d(Villous_ILL4)/dt = -(switch_liverFlag*Villous_ILL4*Qmuc_ILL4/VillousILL4) + (Qmuc_ILL4*Artery_drug) - (DIFF_BASO_ill4*switch_SFdiffbaso*Villous_ILL4*fu_blood/VillousILL4) + (DIFF_BASO_ill4*switch_SFdiffbaso*MEM_ILL4*fu_mem/MILL4) - (CLINT_influx_baso_ILL4*influx_factor_ill4_baso*switch_SFinflux*Villous_ILL4/VillousILL4) - ((1-switch_liverFlag)*Villous_ILL4*Qmuc_ILL4/VillousILL4) + (((switchVmax_efflux_baso==zero)*CLINT_efflux_baso_ILL4*switch_SFefflux_baso*baso_efflux_factor_ill4+switchVmax_efflux_baso*baso_efflux_factor_ill4*phys_Normalized_ESA_baso*phys_BW*basoSurfaceRatio_ILL4*switch_SFefflux_baso/(drug_Km_efflux_baso+MEM_ILL4*fu_mem/MILL4/drug_molar_mass))*MEM_ILL4*fu_mem/MILL4)
58	d(Villous_JEJ1)/dt = -(switch_liverFlag*Villous_JEJ1*Qmuc_JEJ1/VillousJEJ1) + (Qmuc_JEJ1*Artery_drug) + (DIFF_BASO_jej1*switch_SFdiffbaso*MEM_JEJ1*fu_mem/MJEJ1) - (DIFF_BASO_jej1*switch_SFdiffbaso*Villous_JEJ1*fu_blood/VillousJEJ1) - (CLINT_influx_baso_JEJ1*influx_factor_jej1_baso*switch_SFinflux*Villous_JEJ1/VillousJEJ1) - ((1-switch_liverFlag)*Villous_JEJ1*Qmuc_JEJ1/VillousJEJ1) + (((switchVmax_efflux_baso==zero)*CLINT_efflux_baso_JEJ1*switch_SFefflux_baso*baso_efflux_factor_jej1+switchVmax_efflux_baso*baso_efflux_factor_jej1*phys_Normalized_ESA_baso*phys_BW*basoSurfaceRatio_JEJ1*switch_SFefflux_baso/(drug_Km_efflux_baso+MEM_JEJ1*fu_mem/MJEJ1/drug_molar_mass))*MEM_JEJ1*fu_mem/MJEJ1)
59	d(Liver_drug)/dt = 1/Liver*(-(switch_liverFlag*k_Liver_Venous*Liver_drug) + (switch_liverFlag*k_artery_liver*Artery_drug) + (switch_liverFlag*k_spleen_liver*Spleen_drug) + (switch_liverFlag*Villous_DUO*Qmuc_DUO/VillousDUO) + (switch_liverFlag*Villous_JEJ1*Qmuc_JEJ1/VillousJEJ1) + (switch_liverFlag*Villous_JEJ2*Qmuc_JEJ2/VillousJEJ2) + (switch_liverFlag*Villous_ILL1*Qmuc_ILL1/VillousILL1) + (switch_liverFlag*Villous_ILL2*Qmuc_ILL2/VillousILL2) + (switch_liverFlag*Villous_ILL3*Qmuc_ILL3/VillousILL3) + (switch_liverFlag*Villous_ILL4*Qmuc_ILL4/VillousILL4) - (switch_liverFlag*k_liver_bile*Liver_drug) - (switch_liverFlag*k_liver_metabolites*Liver_drug) + (switch_liverFlag*k_serosa_liver*Serosa_drug))
60	d(Serosa_drug)/dt = 1/Serosa*((k_artery_serosa*Artery_drug) - (switch_liverFlag*k_serosa_liver*Serosa_drug) - ((1-switch_liverFlag)*k_serosa_liver*Serosa_drug))
61	d(Bile_drug_1)/dt = ((1-switch_liverFlag_1)*k_Liver_IC_S5_Bile_1*Liver_IC_S5_drug_1) + ((1-switch_liverFlag_1)*k_Liver_IC_S4_Bile_1*Liver_IC_S4_drug_1) + ((1-switch_liverFlag_1)*k_Liver_IC_S3_Bile_1*Liver_IC_S3_drug_1) + ((1-switch_liverFlag_1)*k_Liver_IC_S2_Bile_1*Liver_IC_S2_drug_1) + ((1-

	ODEs
	switch_liverFlag_1)*k_Liver_IC_S1_Bile_1*Liver_IC_S1_drug_1) - (drug_k_bile_deg_1*Bile_drug_1) - (k_transit_1*Bile_drug_1) + (switch_liverFlag_1*k_liver_bile_1*Liver_drug_1)
62	d(Venous_drug_1)/dt = 1/Venous_1*(((1-switch_liverFlag_1)*k_Liver_EC_S5_Venous_1*Liver_EC_S5_drug_1) + (k_rest_venous_1*Rest_drug_1) + (k_bone_venous_1*Bone_drug_1) + (k_skin_venous_1*Skin_drug_1) + (k_heart_venous_1*Heart_drug_1) + (k_adipos_venous_1*Adipose_drug_1) + (k_muscle_venous_1*Muscle_drug_1) + (k_brain_venous_1*Brain_drug_1) + (k_kidney_venous_1*Kidney_drug_1) - (k_venous_lung_1*Venous_drug_1) - (k_venous_urine_CLR_1*Venous_drug_1) + (k_testes_venous_1*Testes_drug_1) - (k_venous_urine_GFR_1*Venous_drug_1) + (switch_liverFlag_1*k_Liver_Venous_1*Liver_drug_1))
63	d(Lung_drug_1)/dt = 1/Lung_1*((k_venous_lung_1*Venous_drug_1) - (k_lung_artery_1*Lung_drug_1))
64	d(Kidney_drug_1)/dt = 1/Kidney_1*(-(k_kidney_venous_1*Kidney_drug_1) + (k_artery_kidney_1*Artery_drug_1))
65	d(Brain_drug_1)/dt = 1/Brain_1*(-(k_brain_venous_1*Brain_drug_1) + (k_artery_brain_1*Artery_drug_1))
66	d(Muscle_drug_1)/dt = 1/Muscle_1*(-(k_muscle_venous_1*Muscle_drug_1) + (k_artery_muscle_1*Artery_drug_1))
67	d(Adipose_drug_1)/dt = 1/Adipose_1*(-(k_adipos_venous_1*Adipose_drug_1) + (k_artery_adipos_1*Artery_drug_1))
68	d(Heart_drug_1)/dt = 1/Heart_1*(-(k_heart_venous_1*Heart_drug_1) + (k_artery_heart_1*Artery_drug_1))
69	d(Skin_drug_1)/dt = 1/Skin_1*(-(k_skin_venous_1*Skin_drug_1) + (k_artery_skin_1*Artery_drug_1))
70	d(Bone_drug_1)/dt = 1/Bone_1*(-(k_bone_venous_1*Bone_drug_1) + (k_artery_bone_1*Artery_drug_1))
71	d(Rest_drug_1)/dt = 1/Rest_1*(-(k_rest_venous_1*Rest_drug_1) + (k_artery_rest_1*Artery_drug_1))
72	d(Artery_drug_1)/dt = 1/Artery_1*(-((1-switch_liverFlag_1)*k_artery_liver_1*Artery_drug_1) - (k_artery_spleen_1*Artery_drug_1) - (k_artery_rest_1*Artery_drug_1) - (k_artery_bone_1*Artery_drug_1) - (k_artery_skin_1*Artery_drug_1) - (k_artery_heart_1*Artery_drug_1) - (k_artery_adipos_1*Artery_drug_1) - (k_artery_muscle_1*Artery_drug_1) - (k_artery_brain_1*Artery_drug_1) - (k_artery_kidney_1*Artery_drug_1) + (k_lung_artery_1*Lung_drug_1) - (k_artery_testes_1*Artery_drug_1) - (Qmuc_DUO_1*Artery_drug_1) - (Qmuc_JEJ1_1*Artery_drug_1) - (Qmuc_JEJ2_1*Artery_drug_1) - (Qmuc_ILL1_1*Artery_drug_1) - (Qmuc_ILL2_1*Artery_drug_1) - (Qmuc_ILL3_1*Artery_drug_1) - (Qmuc_ILL4_1*Artery_drug_1) - (k_artery_serosa_1*Artery_drug_1) - (switch_liverFlag_1*k_artery_liver_1*Artery_drug_1))
73	d(Spleen_drug_1)/dt = 1/Spleen_1*(-((1-switch_liverFlag_1)*k_spleen_liver_1*Spleen_drug_1) + (k_artery_spleen_1*Artery_drug_1) - (switch_liverFlag_1*k_spleen_liver_1*Spleen_drug_1))
74	d(Liver_EC_S1_drug_1)/dt = 1/Liver_EC_S1_1*((k_Liver_IC_S1_Liver_EC_S1_1*Liver_IC_S1_drug_1) - (k_Liver_EC_S1_Liver_IC_S1_1*Liver_EC_S1_drug_1) - (k_Liver_EC_S1_Liver_EC_S2_1*Liver_EC_S1_drug_1) + ((1-switch_liverFlag_1)*k_spleen_liver_1*Spleen_drug_1) + ((1-switch_liverFlag_1)*k_artery_liver_1*Artery_drug_1) + (k_Liver_IC_S1_Liver_EC_S1_efflux_1*Liver_IC_S1_drug_1) + ((1-switch_liverFlag_1)*Villous_DUO_1*Qmuc_DUO_1/VillousDUO_1) + ((1-switch_liverFlag_1)*Villous_JEJ1_1*Qmuc_JEJ1_1/VillousJEJ1_1) + ((1-switch_liverFlag_1)*Villous_JEJ2_1*Qmuc_JEJ2_1/VillousJEJ2_1) + ((1-switch_liverFlag_1)*Villous_ILL1_1*Qmuc_ILL1_1/VillousILL1_1) + ((1-switch_liverFlag_1)*Villous_ILL2_1*Qmuc_ILL2_1/VillousILL2_1) + ((1-switch_liverFlag_1)*Villous_ILL3_1*Qmuc_ILL3_1/VillousILL3_1) + ((1-switch_liverFlag_1)*Villous_ILL4_1*Qmuc_ILL4_1/VillousILL4_1) + ((1-switch_liverFlag_1)*k_serosa_liver_1*Serosa_drug_1))
75	d(Liver_EC_S2_drug_1)/dt = 1/Liver_EC_S2_1*((k_Liver_IC_S2_Liver_EC_S2_1*Liver_IC_S2_drug_1) - (k_Liver_EC_S2_Liver_IC_S2_1*Liver_EC_S2_drug_1) - (k_Liver_EC_S2_Liver_EC_S3_1*Liver_EC_S2_drug_1) + (k_Liver_EC_S1_Liver_EC_S2_1*Liver_EC_S1_drug_1) + (k_Liver_IC_S2_Liver_EC_S2_efflux_1*Liver_IC_S2_drug_1))

	ODEs
76	d(Liver_EC_S3_drug_1)/dt = 1/Liver_EC_S3_1*((k_Liver_IC_S3_Liver__EC_S3_1*Liver_IC_S3_drug_1) - (k_Liver_EC_S3_Liver_IC_S3_1*Liver_EC_S3_drug_1) - (k_Liver_EC_S3_Liver_EC_S4_1*Liver_EC_S3_drug_1) + (k_Liver_EC_S2_Liver_EC_S3_1*Liver_EC_S2_drug_1) + (k_Liver_IC_S3_Liver_EC_S3_efflux_1*Liver_IC_S3_drug_1))
77	d(Liver_EC_S4_drug_1)/dt = 1/Liver_EC_S4_1*((k_Liver_IC_S4_Liver_EC_S4_1*Liver_IC_S4_drug_1) - (k_Liver_EC_S4_Liver_IC_S4_1*Liver_EC_S4_drug_1) - (k_Liver_EC_S4_Liver_EC_S5_1*Liver_EC_S4_drug_1) + (k_Liver_EC_S3_Liver_EC_S4_1*Liver_EC_S3_drug_1) + (k_Liver_IC_S4_Liver_EC_S4_efflux_1*Liver_IC_S4_drug_1))
78	d(Liver_EC_S5_drug_1)/dt = 1/Liver_EC_S5_1*((k_Liver_IC_S5_Liver_EC_S5_1*Liver_IC_S5_drug_1) - (k_Liver_EC_S5_Liver_IC_S5_1*Liver_EC_S5_drug_1) - ((1-switch_liverFlag_1)*k_Liver_EC_S5_Venous_1*Liver_EC_S5_drug_1) + (k_Liver_EC_S4_Liver_EC_S5_1*Liver_EC_S4_drug_1) + (k_Liver_IC_S5_Liver_EC_S5_efflux_1*Liver_IC_S5_drug_1))
79	d(Liver_IC_S5_drug_1)/dt = 1/Liver_IC_S5_1*(-((1-switch_liverFlag_1)*k_Liver_IC_S5_Bile_1*Liver_IC_S5_drug_1) - ((1-switch_liverFlag_1)*k_Liver_IC_S5_Metabolites_1*Liver_IC_S5_drug_1) - (k_Liver_IC_S5_Liver_EC_S5_1*Liver_IC_S5_drug_1) + (k_Liver_EC_S5_Liver_IC_S5_1*Liver_EC_S5_drug_1) - (k_Liver_IC_S5_Liver_EC_S5_efflux_1*Liver_IC_S5_drug_1))
80	d(Liver_IC_S3_drug_1)/dt = 1/Liver_IC_S3_1*(-((1-switch_liverFlag_1)*k_Liver_IC_S3_Metabolites_1*Liver_IC_S3_drug_1) - ((1-switch_liverFlag_1)*k_Liver_IC_S3_Bile_1*Liver_IC_S3_drug_1) - (k_Liver_IC_S3_Liver__EC_S3_1*Liver_IC_S3_drug_1) + (k_Liver_EC_S3_Liver_IC_S3_1*Liver_EC_S3_drug_1) - (k_Liver_IC_S3_Liver_EC_S3_efflux_1*Liver_IC_S3_drug_1))
81	d(Liver_IC_S1_drug_1)/dt = 1/Liver_IC_S1_1*(-((1-switch_liverFlag_1)*k_Liver_IC_S1_Metabolites_1*Liver_IC_S1_drug_1) - ((1-switch_liverFlag_1)*k_Liver_IC_S1_Bile_1*Liver_IC_S1_drug_1) - (k_Liver_IC_S1_Liver_EC_S1_1*Liver_IC_S1_drug_1) + (k_Liver_EC_S1_Liver_IC_S1_1*Liver_EC_S1_drug_1) - (k_Liver_IC_S1_Liver_EC_S1_efflux_1*Liver_IC_S1_drug_1))
82	d(Liver_IC_S2_drug_1)/dt = 1/Liver_IC_S2_1*(-((1-switch_liverFlag_1)*k_Liver_IC_S2_Metabolites_1*Liver_IC_S2_drug_1) - ((1-switch_liverFlag_1)*k_Liver_IC_S2_Bile_1*Liver_IC_S2_drug_1) - (k_Liver_IC_S2_Liver_EC_S2_1*Liver_IC_S2_drug_1) + (k_Liver_EC_S2_Liver_IC_S2_1*Liver_EC_S2_drug_1) - (k_Liver_IC_S2_Liver_EC_S2_efflux_1*Liver_IC_S2_drug_1))
83	d(Metabolites_drug_1)/dt = ((1-switch_liverFlag_1)*k_Liver_IC_S5_Metabolites_1*Liver_IC_S5_drug_1) + ((1-switch_liverFlag_1)*k_Liver_IC_S4_Metabolites_1*Liver_IC_S4_drug_1) + ((1-switch_liverFlag_1)*k_Liver_IC_S3_Metabolites_1*Liver_IC_S3_drug_1) + ((1-switch_liverFlag_1)*k_Liver_IC_S2_Metabolites_1*Liver_IC_S2_drug_1) + ((1-switch_liverFlag_1)*k_Liver_IC_S1_Metabolites_1*Liver_IC_S1_drug_1) + (switch_liverFlag_1*k_liver_metabolites_1*Liver_drug_1)
84	d(Testes_drug_1)/dt = 1/Testes_1*((k_artery_testes_1*Artery_drug_1) - (k_testes_venous_1*Testes_drug_1))
85	d(Urine_drug_1)/dt = (k_venous_urine_CLR_1*Venous_drug_1) + (k_venous_urine_GFR_1*Venous_drug_1)
86	d(Liver_IC_S4_drug_1)/dt = 1/Liver_IC_S4_1*(-((1-switch_liverFlag_1)*k_Liver_IC_S4_Metabolites_1*Liver_IC_S4_drug_1) - ((1-switch_liverFlag_1)*k_Liver_IC_S4_Bile_1*Liver_IC_S4_drug_1) - (k_Liver_IC_S4_Liver_EC_S4_1*Liver_IC_S4_drug_1) + (k_Liver_EC_S4_Liver_IC_S4_1*Liver_EC_S4_drug_1) - (k_Liver_IC_S4_Liver_EC_S4_efflux_1*Liver_IC_S4_drug_1))

	ODEs
87	d(X_STOMACH_SOLID_1)/dt = -(X_STOMACH_SOLID_1/TSTOMACH) - (KD_1*X_STOMACH_SOLID_1*(SOLIF_STOMACH_1-X_STOMACH_DISS_1/STOMACH_1))
88	d(X_STOMACH_DISS_1)/dt = -(X_STOMACH_DISS_1/TSTOMACH) + (KD_1*X_STOMACH_SOLID_1*(SOLIF_STOMACH_1-X_STOMACH_DISS_1/STOMACH_1))
89	d(X_DUO_SOLID_1)/dt = (X_STOMACH_SOLID_1/TSTOMACH) - (X_DUO_SOLID_1/TDUO) - (KD_1*X_DUO_SOLID_1*(SOLIF_DUO_1-X_DUO_DISS_1/VDUO_1))
90	d(X_DUO_DISS_1)/dt = (X_STOMACH_DISS_1/TSTOMACH) + (KD_1*X_DUO_SOLID_1*(SOLIF_DUO_1-X_DUO_DISS_1/VDUO_1)) - (X_DUO_DISS_1/TDUO) - ((DIFF_duo_1*NI_DUO_1*switch_SFdiffapi_1*X_DUO_DISS_1)/VDUO_1) + (((switchVmax_efflux_1==zero_1)*efflux_inhib_duo*CLINT_efflux_DUO_1*efflux_factor_duo*switch_SFefflux_1+efflux_inhib_duo*switchVmax_efflux_1*phys_Normalized_ESA*phys_BW*surfaceRatio_DUO*efflux_factor_duo*switch_SFefflux_1/(drug_Km_efflux_1+MEM_DUO_1*fu_mem_1/MDUO_1/drug_molar_mass_1))*MEM_DUO_1*fu_mem_1/MDUO_1) - (((switchVmax_influx_1==zero)*CLINT_influx_DUO_1*influx_factor_duo*switch_SFinflux_1+switchVmax_influx_1*phys_Normalized_ESA*phys_BW*surfaceRatio_DUO*influx_factor_duo/(drug_Km_influx_1+X_DUO_DISS_1/VDUO_1/drug_molar_mass_1))*X_DUO_DISS_1/VDUO_1) + (DIFF_duo_1*switch_SFdiffapi_1*MEM_DUO_1*fu_mem_1/MDUO_1) + (k_transit_1*Bile_drug_1)
91	d(X_JEJ1_SOLID_1)/dt = (X_DUO_SOLID_1/TDUO) - (X_JEJ1_SOLID_1/TJEJ1) - (KD_1*X_JEJ1_SOLID_1*(SOLIF_JEJ1_1-X_JEJ1_DISS_1/VJEJ1_1))
92	d(X_JEJ1_DISS_1)/dt = (X_DUO_DISS_1/TDUO) + (KD_1*X_JEJ1_SOLID_1*(SOLIF_JEJ1_1-X_JEJ1_DISS_1/VJEJ1_1)) - (X_JEJ1_DISS_1/TJEJ1) - ((DIFF_jej1_1*NI_JEJ1_1*switch_SFdiffapi_1*X_JEJ1_DISS_1)/VJEJ1_1) + (((switchVmax_efflux_1==zero)*efflux_inhib_jej1*CLINT_efflux_JEJ1_1*efflux_factor_jej1*switch_SFefflux_1+efflux_inhib_jej1*switchVmax_efflux_1*phys_Normalized_ESA*phys_BW*surfaceRatio_JEJ1*efflux_factor_jej1*switch_SFefflux_1/(drug_Km_efflux_1+MEM_JEJ1_1*fu_mem_1/MJEJ1_1/drug_molar_mass_1))*MEM_JEJ1_1*fu_mem_1/MJEJ1_1) - (((switchVmax_influx_1==zero)*CLINT_influx_JEJ1_1*influx_factor_jej1*switch_SFinflux_1+switchVmax_influx_1*phys_Normalized_ESA*phys_BW*surfaceRatio_JEJ1*influx_factor_jej1/(drug_Km_influx_1+X_JEJ1_DISS_1/VJEJ1_1/drug_molar_mass_1))*X_JEJ1_DISS_1/VJEJ1_1) + (DIFF_jej1_1*switch_SFdiffapi_1*MEM_JEJ1_1*fu_mem_1/MJEJ1_1)
93	d(MEM_DUO_1)/dt = ((DIFF_duo_1*NI_DUO_1*switch_SFdiffapi_1*X_DUO_DISS_1)/VDUO_1) - (((switchVmax_efflux_1==zero_1)*efflux_inhib_duo*CLINT_efflux_DUO_1*efflux_factor_duo*switch_SFefflux_1+efflux_inhib_duo*switchVmax_efflux_1*phys_Normalized_ESA*phys_BW*surfaceRatio_DUO*efflux_factor_duo*switch_SFefflux_1/(drug_Km_efflux_1+MEM_DUO_1*fu_mem_1/MDUO_1/drug_molar_mass_1))*MEM_DUO_1*fu_mem_1/MDUO_1) + (((switchVmax_influx_1==zero)*CLINT_influx_DUO_1*influx_factor_duo*switch_SFinflux_1+switchVmax_influx_1*phys_Normalized_ESA*phys_BW*surfaceRatio_DUO*influx_factor_duo/(drug_Km_influx_1+X_DUO_DISS_1/VDUO_1/drug_molar_mass_1))*X_DUO_DISS_1/VDUO_1) - ((CLINT_metabolism_1*metabolism_factor_duo_1*switch_SFgutmet_1*MEM_DUO_1*fu_mem_1)/MDUO_1) - (DIFF_BASO_duo_1*switch_SFdiffbaso_1*MEM_DUO_1*fu_mem_1/MDUO_1) + (DIFF_BASO_duo_1*switch_SFdiffbaso_1*Villous_DUO_1*fu_blood_1/VillousDUO_1) - (DIFF_duo_1*switch_SFdiffapi_1*MEM_DUO_1*fu_mem_1/MDUO_1) + (CLINT_influx_baso_DUO_1*influx_factor_duo_baso*switch_SFinflux_1*Villous_DUO_1/VillousDUO_1) - (((switchVmax_efflux_baso_1==zero)*CLINT_efflux_baso_DUO_1*switch_SFefflux_baso_1*baso_efflux_factor_duo+switchVmax_efflux_baso_1*baso_efflux_factor_duo*phys_Normalized_ESA_baso*phys_BW*basoSurfaceRatio_DUO*switch_SFefflux_baso_1/(drug_Km_efflux_baso_1+MEM_DUO_1*fu_mem_1/MDUO_1/drug_molar_mass_1))*MEM_DUO_1*fu_mem_1/MDUO_1)
94	d(MEM_JEJ1_1)/dt = ((DIFF_jej1_1*NI_JEJ1_1*switch_SFdiffapi_1*X_JEJ1_DISS_1)/VJEJ1_1) - (((switchVmax_efflux_1==zero)*efflux_inhib_jej1*CLINT_efflux_JEJ1_1*efflux_factor_jej1*switch_SFefflux_1+efflux_inhib_jej1*switchVmax_efflux_1*phys_Normalized_ESA*phys_BW*surfaceRatio_JEJ1*efflux_factor_jej1*switch_SFefflux_1/(drug_Km_efflux_1+MEM_JEJ1_1*fu_mem_1/MJEJ1_1/drug_molar_mass_1))*MEM_JEJ1_1*fu_mem_1/MJEJ1_1) + (((switchVmax_influx_1==zero)*CLINT_influx_JEJ1_1*influx_factor_jej1*switch_SFinflux_1+switchVmax_influx_1*phys_Normalized_ESA*phys_BW*surfaceRatio_JEJ1*influx_factor_jej1/(drug_Km_influx_1+X_JEJ1_DISS_1/VJEJ1_1/drug_molar_mass_1))*X_JEJ1_DISS_1/VJEJ1_1) -

	ODEs
	((CLINT_metabolism_1*metabolism_factor_jej1_1*switch_SFgutmet_1*MEM_JEJ1_1*fu_mem_1)/MJEJ1_1) - (DIFF_BASO_jej1_1*switch_SFdiffbaso_1*MEM_JEJ1_1*fu_mem_1/MJEJ1_1) + (DIFF_BASO_jej1_1*switch_SFdiffbaso_1*Villous_JEJ1_1*fu_blood_1/VillousJEJ1_1) - (DIFF_jej1_1*switch_SFdiffapi_1*MEM_JEJ1_1*fu_mem_1/MJEJ1_1) + (CLINT_influx_baso_JEJ1_1*influx_factor_jej1_baso*switch_SFinflux_1*Villous_JEJ1_1/VillousJEJ1_1) - (((switchVmax_efflux_baso_1==zero)*CLINT_efflux_baso_JEJ1_1*switch_SFefflux_baso_1*baso_efflux_factor_jej1+switchVmax_efflux_baso_1*baso_efflux_factor_jej1*phys_Normalized_ESA_baso*phys_BW*basoSurfaceRatio_JEJ1*switch_SFefflux_baso_1/(drug_Km_efflux_baso_1+MEM_JEJ1_1*fu_mem_1/MJEJ1_1/drug_molar_mass_1))*MEM_JEJ1_1*fu_mem_1/MJEJ1_1)
95	d(X_JEJ2_SOLID_1)/dt = (X_JEJ1_SOLID_1/TJEJ1) - (X_JEJ2_SOLID_1/TJEJ2) - (KD_1*X_JEJ2_SOLID_1*(SOLIF_JEJ2_1-X_JEJ2_DISS_1/VJEJ2_1))
96	d(X_JEJ2_DISS_1)/dt = (X_JEJ1_DISS_1/TJEJ1) + (KD_1*X_JEJ2_SOLID_1*(SOLIF_JEJ2_1-X_JEJ2_DISS_1/VJEJ2_1)) - (X_JEJ2_DISS_1/TJEJ2) - ((DIFF_jej2_1*NI_JEJ2_1*switch_SFdiffapi_1*X_JEJ2_DISS_1)/VJEJ2_1) + (((switchVmax_efflux_1==zero)*efflux_inhib_jej2*CLINT_efflux_JEJ2_1*efflux_factor_jej2*switch_SFefflux_1+efflux_inhib_jej2*switchVmax_efflux_1*phys_Normalized_ESA*phys_BW*surfaceRatio_JEJ2*efflux_factor_jej2*switch_SFefflux_1/(drug_Km_efflux_1+MEM_JEJ2_1*fu_mem_1/MJEJ2_1/drug_molar_mass_1))*MEM_JEJ2_1*fu_mem_1/MJEJ2_1) - (((switchVmax_influx_1==zero)*CLINT_influx_JEJ2_1*influx_factor_jej2*switch_SFinflux_1+switchVmax_influx_1*phys_Normalized_ESA*phys_BW*surfaceRatio_JEJ2*influx_factor_jej2/(drug_Km_influx_1+X_JEJ2_DISS_1/VJEJ2_1/drug_molar_mass_1))*X_JEJ2_DISS_1/VJEJ2_1) + (DIFF_jej2_1*switch_SFdiffapi_1*MEM_JEJ2_1*fu_mem_1/MJEJ2_1)
97	d(MEM_JEJ2_1)/dt = ((DIFF_jej2_1*NI_JEJ2_1*switch_SFdiffapi_1*X_JEJ2_DISS_1)/VJEJ2_1) - (((switchVmax_efflux_1==zero)*efflux_inhib_jej2*CLINT_efflux_JEJ2_1*efflux_factor_jej2*switch_SFefflux_1+efflux_inhib_jej2*switchVmax_efflux_1*phys_Normalized_ESA*phys_BW*surfaceRatio_JEJ2*efflux_factor_jej2*switch_SFefflux_1/(drug_Km_efflux_1+MEM_JEJ2_1*fu_mem_1/MJEJ2_1/drug_molar_mass_1))*MEM_JEJ2_1*fu_mem_1/MJEJ2_1) + (((switchVmax_influx_1==zero)*CLINT_influx_JEJ2_1*influx_factor_jej2*switch_SFinflux_1+switchVmax_influx_1*phys_Normalized_ESA*phys_BW*surfaceRatio_JEJ2*influx_factor_jej2/(drug_Km_influx_1+X_JEJ2_DISS_1/VJEJ2_1/drug_molar_mass_1))*X_JEJ2_DISS_1/VJEJ2_1) - ((CLINT_metabolism_1*metabolism_factor_jej2_1*switch_SFgutmet_1*MEM_JEJ2_1*fu_mem_1)/MJEJ2_1) - (DIFF_BASO_jej2_1*switch_SFdiffbaso_1*MEM_JEJ2_1*fu_mem_1/MJEJ2_1) + (DIFF_BASO_jej2_1*switch_SFdiffbaso_1*Villous_JEJ2_1*fu_blood_1/VillousJEJ2_1) - (DIFF_jej2_1*switch_SFdiffapi_1*MEM_JEJ2_1*fu_mem_1/MJEJ2_1) + (CLINT_influx_baso_JEJ2_1*influx_factor_jej2_baso*switch_SFinflux_1*Villous_JEJ2_1/VillousJEJ2_1) - (((switchVmax_efflux_baso_1==zero)*CLINT_efflux_baso_JEJ2_1*switch_SFefflux_baso_1*baso_efflux_factor_jej2+switchVmax_efflux_baso_1*baso_efflux_factor_jej2*phys_Normalized_ESA_baso*phys_BW*basoSurfaceRatio_JEJ2*switch_SFefflux_baso_1/(drug_Km_efflux_baso_1+MEM_JEJ2_1*fu_mem_1/MJEJ2_1/drug_molar_mass_1))*MEM_JEJ2_1*fu_mem_1/MJEJ2_1)
98	d(X_ILL1_SOLID_1)/dt = (X_JEJ2_SOLID_1/TJEJ2) - (X_ILL1_SOLID_1/TILL1) - (KD_1*X_ILL1_SOLID_1*(SOLIF_ILL1_1-X_ILL1_DISS_1/VILL1_1))
99	d(X_ILL1_DISS_1)/dt = (X_JEJ2_DISS_1/TJEJ2) + (KD_1*X_ILL1_SOLID_1*(SOLIF_ILL1_1-X_ILL1_DISS_1/VILL1_1)) - (X_ILL1_DISS_1/TILL1) - ((DIFF_ill1_1*NI_ILL1_1*switch_SFdiffapi_1*X_ILL1_DISS_1)/VILL1_1) + (((switchVmax_efflux_1==zero)*efflux_inhib_ill1*CLINT_efflux_ILL1_1*efflux_factor_ill1*switch_SFefflux_1+efflux_inhib_ill1*switchVmax_efflux_1*phys_Normalized_ESA*phys_BW*surfaceRatio_ILL1*efflux_factor_ill1*switch_SFefflux_1/(drug_Km_efflux_1+MEM_ILL1_1*fu_mem_1/MILL1_1/drug_molar_mass_1))*MEM_ILL1_1*fu_mem_1/MILL1_1) - (((switchVmax_influx_1==zero)*CLINT_influx_ILL1_1*influx_factor_ill1*switch_SFinflux_1+switchVmax_influx_1*phys_Normalized_ESA*phys_BW*surfaceRatio_ILL1*influx_factor_ill1/(drug_Km_influx_1+X_ILL1_DISS_1/VILL1_1/drug_molar_mass_1))*X_ILL1_DISS_1/VILL1_1) + (DIFF_ill1_1*switch_SFdiffapi_1*MEM_ILL1_1*fu_mem_1/MILL1_1)
100	d(MEM_ILL1_1)/dt = ((DIFF_ill1_1*NI_ILL1_1*switch_SFdiffapi_1*X_ILL1_DISS_1)/VILL1_1) - (((switchVmax_efflux_1==zero)*efflux_inhib_ill1*CLINT_efflux_ILL1_1*efflux_factor_ill1*switch_SFefflux_1+efflux_inhib_ill1*switchVmax_efflux_1*phys_Normalized_ESA*phys_BW*surfaceRatio_ILL1*efflux_factor_ill1*switch_SFefflux_1/(drug_Km_efflux_1+MEM_ILL1_1*fu_mem_1/MILL1_1/drug_molar_mass_1))*MEM_ILL1_1*f

	ODEs
	u_mem_1/MILL1_1) + (((switchVmax_influx_1==zero)*CLINT_influx_ILL1_1*influx_factor_ill1*switch_SFinflux_1+switchVmax_influx_1*phys_Normalized_ESA*phys_BW*surfaceRatio_ILL1*influx_factor_ill1/(drug_Km_influx_1+X_ILL1_DISS_1/VILL1_1/drug_molar_mass_1))*X_ILL1_DISS_1/VILL1_1) - ((CLINT_metabolism_1*metabolism_factor_ill1_1*switch_SFgutmet_1*MEM_ILL1_1*fu_mem_1)/MILL1_1) - (DIFF_BASO_ill1_1*switch_SFdiffbaso_1*MEM_ILL1_1*fu_mem_1/MILL1_1) + (DIFF_BASO_ill1_1*switch_SFdiffbaso_1*Villous_ILL1_1*fu_blood_1/VillousILL1_1) - (DIFF_ill1_1*switch_SFdiffapi_1*MEM_ILL1_1*fu_mem_1/MILL1_1) + (CLINT_influx_baso_ILL1_1*influx_factor_ill1_baso*switch_SFinflux_1*Villous_ILL1_1/VillousILL1_1) - (((switchVmax_efflux_baso_1==zero)*CLINT_efflux_baso_ILL1_1*switch_SFefflux_baso_1*baso_efflux_factor_ill1+switchVmax_efflux_baso_1*baso_efflux_factor_ill1*phys_Normalized_ESA_baso*phys_BW*basoSurfaceRatio_ILL1*switch_SFefflux_baso_1/(drug_Km_efflux_baso_1+MEM_ILL1_1*fu_mem_1/MILL1_1/drug_molar_mass_1))*MEM_ILL1_1*fu_mem_1/MILL1_1)
101	d(X_ILL2_SOLID_1)/dt = (X_ILL1_SOLID_1/TILL1) - (X_ILL2_SOLID_1/TILL2) - (KD_1*X_ILL2_SOLID_1*(SOLIF_ILL2_1-X_ILL2_DISS_1/VILL2_1))
102	d(X_ILL2_DISS_1)/dt = (X_ILL1_DISS_1/TILL1) + (KD_1*X_ILL2_SOLID_1*(SOLIF_ILL2_1-X_ILL2_DISS_1/VILL2_1)) - (X_ILL2_DISS_1/TILL2) - ((DIFF_ill2_1*NI_ILL2_1*switch_SFdiffapi_1*X_ILL2_DISS_1)/VILL2_1) + (((switchVmax_efflux_1==zero)*efflux_inhib_ill2*CLINT_efflux_ILL2_1*efflux_factor_ill2*switch_SFefflux_1+efflux_inhib_ill2*switchVmax_efflux_1*phys_Normalized_ESA*phys_BW*surfaceRatio_ILL2*efflux_factor_ill2*switch_SFefflux_1/(drug_Km_efflux_1+MEM_ILL2_1*fu_mem_1/MILL2_1/drug_molar_mass_1))*MEM_ILL2_1*fu_mem_1/MILL2_1) - (((switchVmax_influx_1==zero)*CLINT_influx_ILL2_1*influx_factor_ill2*switch_SFinflux_1+switchVmax_influx_1*phys_Normalized_ESA*phys_BW*surfaceRatio_ILL2*influx_factor_ill2/(drug_Km_influx_1+X_ILL2_DISS_1/VILL2_1/drug_molar_mass_1))*X_ILL2_DISS_1/VILL2_1) + (DIFF_ill2_1*switch_SFdiffapi_1*MEM_ILL2_1*fu_mem_1/MILL2_1)
103	d(MEM_ILL2_1)/dt = ((DIFF_ill2_1*NI_ILL2_1*switch_SFdiffapi_1*X_ILL2_DISS_1)/VILL2_1) - (((switchVmax_efflux_1==zero)*efflux_inhib_ill2*CLINT_efflux_ILL2_1*efflux_factor_ill2*switch_SFefflux_1+efflux_inhib_ill2*switchVmax_efflux_1*phys_Normalized_ESA*phys_BW*surfaceRatio_ILL2*efflux_factor_ill2*switch_SFefflux_1/(drug_Km_efflux_1+MEM_ILL2_1*fu_mem_1/MILL2_1/drug_molar_mass_1))*MEM_ILL2_1*fu_mem_1/MILL2_1) + (((switchVmax_influx_1==zero)*CLINT_influx_ILL2_1*influx_factor_ill2*switch_SFinflux_1+switchVmax_influx_1*phys_Normalized_ESA*phys_BW*surfaceRatio_ILL2*influx_factor_ill2/(drug_Km_influx_1+X_ILL2_DISS_1/VILL2_1/drug_molar_mass_1))*X_ILL2_DISS_1/VILL2_1) - ((CLINT_metabolism_1*metabolism_factor_ill2_1*switch_SFgutmet_1*MEM_ILL2_1*fu_mem_1)/MILL2_1) + (DIFF_BASO_ill2_1*switch_SFdiffbaso_1*Villous_ILL2_1*fu_blood_1/VillousILL2_1) - (DIFF_BASO_ill2_1*switch_SFdiffbaso_1*MEM_ILL2_1*fu_mem_1/MILL2_1) - (DIFF_ill2_1*switch_SFdiffapi_1*MEM_ILL2_1*fu_mem_1/MILL2_1) + (CLINT_influx_baso_ILL2_1*influx_factor_ill2_baso*switch_SFinflux_1*Villous_ILL2_1/VillousILL2_1) - (((switchVmax_efflux_baso_1==zero)*CLINT_efflux_baso_ILL2_1*switch_SFefflux_baso_1*baso_efflux_factor_ill2+switchVmax_efflux_baso_1*baso_efflux_factor_ill2*phys_Normalized_ESA_baso*phys_BW*basoSurfaceRatio_ILL2*switch_SFefflux_baso_1/(drug_Km_efflux_baso_1+MEM_ILL2_1*fu_mem_1/MILL2_1/drug_molar_mass_1))*MEM_ILL2_1*fu_mem_1/MILL2_1)
104	d(X_ILL3_SOLID_1)/dt = (X_ILL2_SOLID_1/TILL2) - (X_ILL3_SOLID_1/TILL3) - (KD_1*X_ILL3_SOLID_1*(SOLIF_ILL3_1-X_ILL3_DISS_1/VILL3_1))
105	d(X_ILL3_DISS_1)/dt = (X_ILL2_DISS_1/TILL2) + (KD_1*X_ILL3_SOLID_1*(SOLIF_ILL3_1-X_ILL3_DISS_1/VILL3_1)) - (X_ILL3_DISS_1/TILL3) - ((DIFF_ill3_1*NI_ILL3_1*switch_SFdiffapi_1*X_ILL3_DISS_1)/VILL3_1) + (((switchVmax_efflux_1==zero)*efflux_inhib_ill3*CLINT_efflux_ILL3_1*efflux_factor_ill3*switch_SFefflux_1+efflux_inhib_ill3*switchVmax_efflux_1*phys_Normalized_ESA*phys_BW*surfaceRatio_ILL3*efflux_factor_ill3*switch_SFefflux_1/(drug_Km_efflux_1+MEM_ILL3_1*fu_mem_1/MILL3_1/drug_molar_mass_1))*MEM_ILL3_1*fu_mem_1/MILL3_1) - (((switchVmax_influx_1==zero)*CLINT_influx_ILL3_1*influx_factor_ill3*switch_SFinflux_1+switchVmax_influx_1*phys_Normalized_ESA*phys_BW*surfaceRatio_ILL3*influx_factor_ill3/(drug_Km_influx_1+X_ILL3_DISS_1/VILL3_1/drug_molar_mass_1))*X_ILL3_DISS_1/VILL3_1) + (DIFF_ill3_1*switch_SFdiffapi_1*MEM_ILL3_1*fu_mem_1/MILL3_1)

	ODEs
106	d(MEM_ILL3_1)/dt = ((DIFF_ill3_1*NI_ILL3_1*switch_SFdiffapi_1*X_ILL3_DISS_1)/VILL3_1) - (((switchVmax_efflux_1==zero)*efflux_inhib_ill3*CLINT_efflux_ILL3_1*efflux_factor_ill3*switch_SFefflux_1+efflux_inhib_ill3*switchVmax_efflux_1*phys_Normalized_ESA*phys_BW*surfaceRatio_ILL3*efflux_factor_ill3*switch_SFefflux_1/(drug_Km_efflux_1+MEM_ILL3_1*fu_mem_1/MILL3_1/drug_molar_mass_1))*MEM_ILL3_1*fu_mem_1/MILL3_1) + (((switchVmax_influx_1==zero)*CLINT_influx_ILL3_1*influx_factor_ill3*switch_SFinflux_1+switchVmax_influx_1*phys_Normalized_ESA*phys_BW*surfaceRatio_ILL3*influx_factor_ill3/(drug_Km_influx_1+X_ILL3_DISS_1/VILL3_1/drug_molar_mass_1))*X_ILL3_DISS_1/VILL3_1) - ((CLINT_metabolism_1*metabolism_factor_ill3_1*switch_SFgutmet_1*MEM_ILL3_1*fu_mem_1)/MILL3_1) + (DIFF_BASO_ill3_1*switch_SFdiffbaso_1*Villous_ILL3_1*fu_blood_1/VillousILL3_1) - (DIFF_BASO_ill3_1*switch_SFdiffbaso_1*MEM_ILL3_1*fu_mem_1/MILL3_1) - (DIFF_ill3_1*switch_SFdiffapi_1*MEM_ILL3_1*fu_mem_1/MILL3_1) + (CLINT_influx_baso_ILL3_1*influx_factor_ill3_baso*switch_SFinflux_1*Villous_ILL3_1/VillousILL3_1) - (((switchVmax_efflux_baso_1==zero)*CLINT_efflux_baso_ILL3_1*switch_SFefflux_baso_1*baso_efflux_factor_ill3+switchVmax_efflux_baso_1*baso_efflux_factor_ill3*phys_Normalized_ESA_baso*phys_BW*basoSurfaceRatio_ILL3*switch_SFefflux_baso_1/(drug_Km_efflux_baso_1+MEM_ILL3_1*fu_mem_1/MILL3_1/drug_molar_mass_1))*MEM_ILL3_1*fu_mem_1/MILL3_1)
107	d(X_ILL4_SOLID_1)/dt = (X_ILL3_SOLID_1/TILL3) - (X_ILL4_SOLID_1/TILL4) - (KD_1*X_ILL4_SOLID_1*(SOLIF_ILL4_1-X_ILL4_DISS_1/VILL4_1))
108	d(X_ILL4_DISS_1)/dt = (X_ILL3_DISS_1/TILL3) + (KD_1*X_ILL4_SOLID_1*(SOLIF_ILL4_1-X_ILL4_DISS_1/VILL4_1)) - (X_ILL4_DISS_1/TILL4) - ((DIFF_ill4_1*NI_ILL4_1*switch_SFdiffapi_1*X_ILL4_DISS_1)/VILL4_1) + (((switchVmax_efflux_1==zero)*efflux_inhib_ill4*CLINT_efflux_ILL4_1*efflux_factor_ill4*switch_SFefflux_1+efflux_inhib_ill4*switchVmax_efflux_1*phys_Normalized_ESA*phys_BW*surfaceRatio_ILL4*efflux_factor_ill4*switch_SFefflux_1/(drug_Km_efflux_1+MEM_ILL4_1*fu_mem_1/MILL4_1/drug_molar_mass_1))*MEM_ILL4_1*fu_mem_1/MILL4_1) - (((switchVmax_influx_1==zero)*CLINT_influx_ILL4_1*influx_factor_ill4*switch_SFinflux_1+switchVmax_influx_1*phys_Normalized_ESA*phys_BW*surfaceRatio_ILL4*influx_factor_ill4/(drug_Km_influx_1+X_ILL4_DISS_1/VILL4_1/drug_molar_mass_1))*X_ILL4_DISS_1/VILL4_1) + (DIFF_ill4_1*switch_SFdiffapi_1*MEM_ILL4_1*fu_mem_1/MILL4_1)
109	d(MEM_ILL4_1)/dt = ((DIFF_ill4_1*NI_ILL4_1*switch_SFdiffapi_1*X_ILL4_DISS_1)/VILL4_1) - (((switchVmax_efflux_1==zero)*efflux_inhib_ill4*CLINT_efflux_ILL4_1*efflux_factor_ill4*switch_SFefflux_1+efflux_inhib_ill4*switchVmax_efflux_1*phys_Normalized_ESA*phys_BW*surfaceRatio_ILL4*efflux_factor_ill4*switch_SFefflux_1/(drug_Km_efflux_1+MEM_ILL4_1*fu_mem_1/MILL4_1/drug_molar_mass_1))*MEM_ILL4_1*fu_mem_1/MILL4_1) + (((switchVmax_influx_1==zero)*CLINT_influx_ILL4_1*influx_factor_ill4*switch_SFinflux_1+switchVmax_influx_1*phys_Normalized_ESA*phys_BW*surfaceRatio_ILL4*influx_factor_ill4/(drug_Km_influx_1+X_ILL4_DISS_1/VILL4_1/drug_molar_mass_1))*X_ILL4_DISS_1/VILL4_1) - ((CLINT_metabolism_1*metabolism_factor_ill4_1*switch_SFgutmet_1*MEM_ILL4_1*fu_mem_1)/MILL4_1) - (DIFF_ill4_1*switch_SFdiffapi_1*MEM_ILL4_1*fu_mem_1/MILL4_1) + (DIFF_BASO_ill4_1*switch_SFdiffbaso_1*Villous_ILL4_1*fu_blood_1/VillousILL4_1) - (DIFF_BASO_ill4_1*switch_SFdiffbaso_1*MEM_ILL4_1*fu_mem_1/MILL4_1) + (CLINT_influx_baso_ILL4_1*influx_factor_ill4_baso*switch_SFinflux_1*Villous_ILL4_1/VillousILL4_1) - (((switchVmax_efflux_baso_1==zero)*CLINT_efflux_baso_ILL4_1*switch_SFefflux_baso_1*baso_efflux_factor_ill4+switchVmax_efflux_baso_1*baso_efflux_factor_ill4*phys_Normalized_ESA_baso*phys_BW*basoSurfaceRatio_ILL4*switch_SFefflux_baso_1/(drug_Km_efflux_baso_1+MEM_ILL4_1*fu_mem_1/MILL4_1/drug_molar_mass_1))*MEM_ILL4_1*fu_mem_1/MILL4_1)
110	d(X_CECUM_SOLID_1)/dt = (X_ILL4_SOLID_1/TILL4)
111	d(X_CECUM_DISS_1)/dt = (X_ILL4_DISS_1/TILL4)
112	d(Villous_DUO_1)/dt = (DIFF_BASO_duo_1*switch_SFdiffbaso_1*MEM_DUO_1*fu_mem_1/MDUO_1) - (DIFF_BASO_duo_1*switch_SFdiffbaso_1*Villous_DUO_1*fu_blood_1/VillousDUO_1) - (CLINT_influx_baso_DUO_1*influx_factor_duo_baso*switch_SFinflux_1*Villous_DUO_1/VillousDUO_1) + (Qmuc_DUO_1*Artery_drug_1) - (switch_liverFlag_1*Villous_DUO_1*Qmuc_DUO_1/VillousDUO_1) - ((1-switch_liverFlag_1)*Villous_DUO_1*Qmuc_DUO_1/VillousDUO_1) + (((switchVmax_efflux_baso_1==zero)*CLINT_efflux_baso_DUO_1*switch_SFefflux_baso_1*baso_efflux_fact

	ODEs
	or_duo+switchVmax_efflux_baso_1*baso_efflux_factor_duo*phys_Normalized_ESA_baso*phys_BW*basoSurfaceRatio_DUO*switch_SFefflux_baso_1/(drug_Km_efflux_baso_1+MEM_DUO_1*fu_mem_1/MDUO_1/drug_molar_mass_1))*MEM_DUO_1*fu_mem_1/MDUO_1)
113	d(Villous_JEJ2_1)/dt = (DIFF_BASO_jej2_1*switch_SFdiffbaso_1*MEM_JEJ2_1*fu_mem_1/MJEJ2_1) - (DIFF_BASO_jej2_1*switch_SFdiffbaso_1*Villous_JEJ2_1*fu_blood_1/VillousJEJ2_1) - (CLINT_influx_baso_JEJ2_1*influx_factor_jej2_baso*switch_SFinflux_1*Villous_JEJ2_1/VillousJEJ2_1) + (Qmuc_JEJ2_1*Artery_drug_1) - (switch_liverFlag_1*Villous_JEJ2_1*Qmuc_JEJ2_1/VillousJEJ2_1) - ((1-switch_liverFlag_1)*Villous_JEJ2_1*Qmuc_JEJ2_1/VillousJEJ2_1) + (((switchVmax_efflux_baso_1==zero)*CLINT_efflux_baso_JEJ2_1*switch_SFefflux_baso_1*baso_efflux_factor_jej2+switchVmax_efflux_baso_1*baso_efflux_factor_jej2*phys_Normalized_ESA_baso*phys_BW*basoSurfaceRatio_JEJ2*switch_SFefflux_baso_1/(drug_Km_efflux_baso_1+MEM_JEJ2_1*fu_mem_1/MJEJ2_1/drug_molar_mass_1))*MEM_JEJ2_1*fu_mem_1/MJEJ2_1)
114	d(Villous_ILL1_1)/dt = (DIFF_BASO_ill1_1*switch_SFdiffbaso_1*MEM_ILL1_1*fu_mem_1/MILL1_1) - (DIFF_BASO_ill1_1*switch_SFdiffbaso_1*Villous_ILL1_1*fu_blood_1/VillousILL1_1) - (CLINT_influx_baso_ILL1_1*influx_factor_ill1_baso*switch_SFinflux_1*Villous_ILL1_1/VillousILL1_1) + (Qmuc_ILL1_1*Artery_drug_1) - (switch_liverFlag_1*Villous_ILL1_1*Qmuc_ILL1_1/VillousILL1_1) - ((1-switch_liverFlag_1)*Villous_ILL1_1*Qmuc_ILL1_1/VillousILL1_1) + (((switchVmax_efflux_baso_1==zero)*CLINT_efflux_baso_ILL1_1*switch_SFefflux_baso_1*baso_efflux_factor_ill1+switchVmax_efflux_baso_1*baso_efflux_factor_ill1*phys_Normalized_ESA_baso*phys_BW*basoSurfaceRatio_ILL1*switch_SFefflux_baso_1/(drug_Km_efflux_baso_1+MEM_ILL1_1*fu_mem_1/MILL1_1/drug_molar_mass_1))*MEM_ILL1_1*fu_mem_1/MILL1_1)
115	d(Villous_ILL2_1)/dt = -(DIFF_BASO_ill2_1*switch_SFdiffbaso_1*Villous_ILL2_1*fu_blood_1/VillousILL2_1) + (DIFF_BASO_ill2_1*switch_SFdiffbaso_1*MEM_ILL2_1*fu_mem_1/MILL2_1) - (CLINT_influx_baso_ILL2_1*influx_factor_ill2_baso*switch_SFinflux_1*Villous_ILL2_1/VillousILL2_1) + (Qmuc_ILL2_1*Artery_drug_1) - (switch_liverFlag_1*Villous_ILL2_1*Qmuc_ILL2_1/VillousILL2_1) - ((1-switch_liverFlag_1)*Villous_ILL2_1*Qmuc_ILL2_1/VillousILL2_1) + (((switchVmax_efflux_baso_1==zero)*CLINT_efflux_baso_ILL2_1*switch_SFefflux_baso_1*baso_efflux_factor_ill2+switchVmax_efflux_baso_1*baso_efflux_factor_ill2*phys_Normalized_ESA_baso*phys_BW*basoSurfaceRatio_ILL2*switch_SFefflux_baso_1/(drug_Km_efflux_baso_1+MEM_ILL2_1*fu_mem_1/MILL2_1/drug_molar_mass_1))*MEM_ILL2_1*fu_mem_1/MILL2_1)
116	d(Villous_ILL3_1)/dt = -(DIFF_BASO_ill3_1*switch_SFdiffbaso_1*Villous_ILL3_1*fu_blood_1/VillousILL3_1) + (DIFF_BASO_ill3_1*switch_SFdiffbaso_1*MEM_ILL3_1*fu_mem_1/MILL3_1) - (CLINT_influx_baso_ILL3_1*influx_factor_ill3_baso*switch_SFinflux_1*Villous_ILL3_1/VillousILL3_1) + (Qmuc_ILL3_1*Artery_drug_1) - (switch_liverFlag_1*Villous_ILL3_1*Qmuc_ILL3_1/VillousILL3_1) - ((1-switch_liverFlag_1)*Villous_ILL3_1*Qmuc_ILL3_1/VillousILL3_1) + (((switchVmax_efflux_baso_1==zero)*CLINT_efflux_baso_ILL3_1*switch_SFefflux_baso_1*baso_efflux_factor_ill3+switchVmax_efflux_baso_1*baso_efflux_factor_ill3*phys_Normalized_ESA_baso*phys_BW*basoSurfaceRatio_ILL3*switch_SFefflux_baso_1/(drug_Km_efflux_baso_1+MEM_ILL3_1*fu_mem_1/MILL3_1/drug_molar_mass_1))*MEM_ILL3_1*fu_mem_1/MILL3_1)
117	d(Villous_ILL4_1)/dt = -(DIFF_BASO_ill4_1*switch_SFdiffbaso_1*Villous_ILL4_1*fu_blood_1/VillousILL4_1) + (DIFF_BASO_ill4_1*switch_SFdiffbaso_1*MEM_ILL4_1*fu_mem_1/MILL4_1) - (CLINT_influx_baso_ILL4_1*influx_factor_ill4_baso*switch_SFinflux_1*Villous_ILL4_1/VillousILL4_1) + (Qmuc_ILL4_1*Artery_drug_1) - (switch_liverFlag_1*Villous_ILL4_1*Qmuc_ILL4_1/VillousILL4_1) - ((1-switch_liverFlag_1)*Villous_ILL4_1*Qmuc_ILL4_1/VillousILL4_1) + (((switchVmax_efflux_baso_1==zero)*CLINT_efflux_baso_ILL4_1*switch_SFefflux_baso_1*baso_efflux_factor_ill4+switchVmax_efflux_baso_1*baso_efflux_factor_ill4*phys_Normalized_ESA_baso*phys_BW*basoSurfaceRatio_ILL4*switch_SFefflux_baso_1/(drug_Km_efflux_baso_1+MEM_ILL4_1*fu_mem_1/MILL4_1/drug_molar_mass_1))*MEM_ILL4_1*fu_mem_1/MILL4_1)
118	d(Villous_JEJ1_1)/dt = (DIFF_BASO_jej1_1*switch_SFdiffbaso_1*MEM_JEJ1_1*fu_mem_1/MJEJ1_1) - (DIFF_BASO_jej1_1*switch_SFdiffbaso_1*Villous_JEJ1_1*fu_blood_1/VillousJEJ1_1) - (CLINT_influx_baso_JEJ1_1*influx_factor_jej1_baso*switch_SFinflux_1*Villous_JEJ1_1/VillousJEJ1_1) + (Qmuc_JEJ1_1*Artery_drug_1) - (switch_liverFlag_1*Villous_JEJ1_1*Qmuc_JEJ1_1/VillousJEJ1_1) - ((1-switch_liverFlag_1)*Villous_JEJ1_1*Qmuc_JEJ1_1/VillousJEJ1_1) + (((switchVmax_efflux_baso_1==zero)*CLINT_efflux_baso_JEJ1_1*switch_SFefflux_baso_1*baso_efflux_factor_jej1+switchVmax_efflux_baso_1*baso_efflux_factor_jej1*phys_Normalized_ESA_baso*phys_BW*basoSur

	ODEs
	faceRatio_JEJ1*switch_SFefflux_baso_1/(drug_Km_efflux_baso_1+MEM_JEJ1_1*fu_mem_1/MJEJ1_1/drug_molar_mass_1))*MEM_JEJ1_1*fu_mem_1/MJEJ1_1)
119	d(Liver_drug_1)/dt = 1/Liver_1*(-(switch_liverFlag_1*k_Liver_Venous_1*Liver_drug_1) + (switch_liverFlag_1*Villous_DUO_1*Qmuc_DUO_1/VillousDUO_1) + (switch_liverFlag_1*Villous_JEJ1_1*Qmuc_JEJ1_1/VillousJEJ1_1) + (switch_liverFlag_1*Villous_JEJ2_1*Qmuc_JEJ2_1/VillousJEJ2_1) + (switch_liverFlag_1*Villous_ILL1_1*Qmuc_ILL1_1/VillousILL1_1) + (switch_liverFlag_1*Villous_ILL2_1*Qmuc_ILL2_1/VillousILL2_1) + (switch_liverFlag_1*Villous_ILL3_1*Qmuc_ILL3_1/VillousILL3_1) + (switch_liverFlag_1*Villous_ILL4_1*Qmuc_ILL4_1/VillousILL4_1) + (switch_liverFlag_1*k_serosa_liver_1*Serosa_drug_1) + (switch_liverFlag_1*k_artery_liver_1*Artery_drug_1) + (switch_liverFlag_1*k_spleen_liver_1*Spleen_drug_1) - (switch_liverFlag_1*k_liver_metabolites_1*Liver_drug_1) - (switch_liverFlag_1*k_liver_bile_1*Liver_drug_1))
120	d(Serosa_drug_1)/dt = 1/Serosa_1*((k_artery_serosa_1*Artery_drug_1) - (switch_liverFlag_1*k_serosa_liver_1*Serosa_drug_1) - ((1-switch_liverFlag_1)*k_serosa_liver_1*Serosa_drug_1))

Program Setup

Model Setup

Variants

	Type	Name	Human_phys	Human_physiology_ADAM	Clarithromycin
1	parameter	phys_BW	70		70
2	parameter	phys_Normalized_Q_adipose	4		
3	parameter	phys_Normalized_Q_bone	4		
4	parameter	phys_Normalized_Q_brain	10		
5	parameter	phys_Normalized_Q_gut	17		
6	parameter	phys_Normalized_Q_heart	3		
7	parameter	phys_Normalized_Q_kidney	15		
8	parameter	phys_Normalized_Q_liver	20		
9	parameter	phys_Normalized_Q_lung	80		
10	parameter	phys_Normalized_Q_muscle	14		
11	parameter	phys_Normalized_Q_remainder	1		
12	parameter	phys_Normalized_Q_skin	4		
13	parameter	phys_Normalized_Q_spleen	2		

	Type	Name	Human_phys	Human_physiology_ADAM	Clarithromycin
14	parameter	phys_Normalized_Q_testes	0		
15	parameter	phys_Normalized_weight_adipose	197		
16	parameter	phys_Normalized_weight_artery	25.7		
17	parameter	phys_Normalized_weight_bone	158		
18	parameter	phys_Normalized_weight_brain	21		
19	parameter	phys_Normalized_weight_gut	18		
20	parameter	phys_Normalized_weight_heart	5		
21	parameter	phys_Normalized_weight_kidney	5		
22	parameter	phys_Normalized_weight_liver_blood	4.9		
23	parameter	phys_Normalized_weight_liver_tissue	18		
24	parameter	phys_Normalized_weight_lung	8		
25	parameter	phys_Normalized_weight_muscle	416		
26	parameter	phys_Normalized_weight_remainder	100		
27	parameter	phys_Normalized_weight_skin	41		
28	parameter	phys_Normalized_weight_spleen	3		
29	parameter	phys_HPGL	125		
30	parameter	phys_Normalized_weight_venous	51.4		
31	parameter	LL		680	
32	parameter	LR		1.75	
33	parameter	phys_ESA		120000	
34	parameter	TSTOMACH		16.2	
35	parameter	TDUO		9.384	
36	parameter	TJEJ1		35.292	

	Type	Name	Human_phys	Human_physiology_ADAM	Clarithromycin
37	parameter	TJEJ2		35.292	
38	parameter	TILL1		31.008	
39	parameter	TILL2		31.008	
40	parameter	TILL3		31.008	
41	parameter	TILL4		31.008	
42	parameter	phys_Normalized_weight_stomach		2.1	
43	parameter	pHStomach		1.5	
44	parameter	pHDuo		6.4	
45	parameter	pHJej1		6.5	
46	parameter	pHJej2		6.6	
47	parameter	pHIll1		6.8	
48	parameter	pHIll2		7	
49	parameter	pHIll3		7.7	
50	parameter	pHIll4		7.3	
51	parameter	BW_average		70	
52	parameter	numIntestinalCompartments		7	
53	parameter	Gut_EC_fraction		0.3719	
54	parameter	Gut_IC_fraction		0.6281	
55	parameter	influx_factor_duo		1	
56	parameter	influx_factor_jej1		1	
57	parameter	influx_factor_jej2		1	
58	parameter	influx_factor_ill1		1	
59	parameter	influx_factor_ill2		1	
60	parameter	influx_factor_ill3		1	
61	parameter	influx_factor_ill4		1	
62	parameter	efflux_factor_duo		0.51	
63	parameter	efflux_factor_jej1		1	
64	parameter	efflux_factor_jej2		1.46	
65	parameter	efflux_factor_ill1		1.5	
66	parameter	efflux_factor_ill2		1.51	
67	parameter	efflux_factor_ill3		1.52	
68	parameter	efflux_factor_ill4		1.51	
69	parameter	volumeRatio_DUO		0.11534	
70	parameter	volumeRatio_JEJ1		0.22722	

	Type	Name	Human_phys	Human_physiology_ADAM	Clarithromycin
71	parameter	volumeRatio_JEJ2		0.15917	
72	parameter	volumeRatio_ILL1		0.12687	
73	parameter	volumeRatio_ILL2		0.12687	
74	parameter	volumeRatio_ILL3		0.12457	
75	parameter	volumeRatio_ILL4		0.11995	
76	parameter	flowRatio_DUO		0.088	
77	parameter	flowRatio_JEJ1		0.242	
78	parameter	flowRatio_JEJ2		0.242	
79	parameter	flowRatio_ILL1		0.107	
80	parameter	flowRatio_ILL2		0.107	
81	parameter	flowRatio_ILL3		0.107	
82	parameter	flowRatio_ILL4		0.107	
83	parameter	metabolism_factor_duo		1	
84	parameter	metabolism_factor_jej1		1	
85	parameter	metabolism_factor_jej2		1	
86	parameter	metabolism_factor_ill1		1	
87	parameter	metabolism_factor_ill2		1	
88	parameter	metabolism_factor_ill3		1	
89	parameter	metabolism_factor_ill4		1	
90	parameter	phys_Normalized_weight_enterocyte		7.3857	
91	parameter	LumenTotal		126.95	
92	parameter	lumenvolumeRatio_DUO		0.27058	
93	parameter	lumenvolumeRatio_JEJ1		0.16621	
94	parameter	lumenvolumeRatio_JEJ2		0.16621	
95	parameter	lumenvolumeRatio_ILL1		0.099252	

	Type	Name	Human_phys	Human_physiology_ADAM	Clarithromycin
96	parameter	lumenvolumeRatio_ILL2		0.099252	
97	parameter	lumenvolumeRatio_ILL3		0.099252	
98	parameter	lumenvolumeRatio_ILL4		0.099252	
99	parameter	StomachLumenTotal		50	
100	parameter	phys_ESA_baso		6703	
101	parameter	surfaceRatio_DUO		0.115	
102	parameter	surfaceRatio_JEJ1		0.227	
103	parameter	surfaceRatio_JEJ2		0.159	
104	parameter	surfaceRatio_ILL1		0.127	
105	parameter	surfaceRatio_ILL2		0.127	
106	parameter	surfaceRatio_ILL3		0.125	
107	parameter	surfaceRatio_ILL4		0.12	
108	parameter	basoSurfaceRatio_DUO		0.0526	
109	parameter	basoSurfaceRatio_JEJ1		0.2026	
110	parameter	basoSurfaceRatio_JEJ2		0.2026	
111	parameter	basoSurfaceRatio_ILL1		0.1356	
112	parameter	basoSurfaceRatio_ILL2		0.1356	
113	parameter	basoSurfaceRatio_ILL3		0.1356	
114	parameter	basoSurfaceRatio_ILL4		0.1356	
115	parameter	drug_fQ		0.2571	
116	parameter	influx_factor_duo_baso		1	
117	parameter	influx_factor_jej1_baso		1	
118	parameter	influx_factor_jej2_baso		1	

	Type	Name	Human_phys	Human_physiology_ADAM	Clarithromycin
119	parameter	influx_factor_ill1_baso		1	
120	parameter	influx_factor_ill2_baso		1	
121	parameter	influx_factor_ill3_baso		1	
122	parameter	influx_factor_ill4_baso		1	
123	parameter	fu_mem			0.016351
124	parameter	LOGP			3.16
125	parameter	MW			747970
126	parameter	REFPHSOL			2.4
127	parameter	PSIZE			0.0065
128	parameter	pKA			8.99
129	parameter	switchVmax_influx			0
130	parameter	switchVmax_efflux			0
131	parameter	switch_SFinflux			1
132	parameter	switch_SFefflux			1
133	parameter	switch_SFgutmet			1
134	parameter	switch_SFdiffapi			1
135	parameter	switch_SFdiffbaso			1
136	parameter	CL_inf_api			0
137	parameter	CL_eff			0
138	parameter	CLINT_metabolism			0
139	parameter	CL_inf_baso			0
140	parameter	drug_Kp_serosa_raw			3.4145
141	parameter	drug_Kp_liver_raw			5.9321
142	parameter	diff_baso			11.9
143	parameter	diff_api			11.9
144	parameter	drug_PSbileg			0
145	parameter	drug_fuLiver			0.016351
146	parameter	drug_fumic			0.60838
147	parameter	drug_funic			0.60838
148	parameter	drug_fuplasma			0.18
149	parameter	drug_HLM_CLint			0.0436

	Type	Name	Human_phys	Human_physiology_ADAM	Clarithromycin
150	parameter	drug_Kp_adipose_raw			0.66275
151	parameter	drug_Kp_bone_raw			0.99585
152	parameter	drug_Kp_brain_raw			0.73224
153	parameter	drug_Kp_gut_raw			3.4145
154	parameter	drug_Kp_heart_raw			3.5854
155	parameter	drug_Kp_kidney_raw			2.9512
156	parameter	drug_Kp_lung_raw			2.9785
157	parameter	drug_Kp_muscle_raw			1.9256
158	parameter	drug_Kp_rest_raw			2.557
159	parameter	switch_SFKp			1
160	parameter	drug_Kp_skin_raw			1.413
161	parameter	drug_Kp_spleen_raw			1.8716
162	parameter	drug_molar_mass			747950
163	parameter	drug_BRP			1
164	parameter	switch_SFbile			1
165	parameter	switch_SFmet			1
166	parameter	switch_SFdiff			1
167	parameter	drug_CLrenal			7.2
168	parameter	drug_FR			0
169	parameter	switch_slow_dist_Kp			1
170	parameter	switch_SFrenal			1
171	parameter	switch_liverFlag			1
172	parameter	fu_blood			0.18
173	parameter	drug_pKABase1			8.99
174	parameter	drug_inputFlag			1
175	parameter	drug_CLmetg			0
176	parameter	drug_efflux_Ki			4

Data Step

Data Map

Classification	Value
group	Group
independent	Time_hr
response	CLA_Conc_ng_mL ~ Plasma_total.Plasma_total_drug
dose from data	Dose_IV_mg -> Venous.Venous_drug
	Infusion Data Column: IV_Rate_mg_hr
dose from data	Dose_PO_milligram -> STOMACH.X_STOMACH_DISS
	Bolus

Variant and Dose Setup Step

Variant and Dose Setup

Group	Variants1	Variants2	Variants3	Variants4	Doses1	Doses2	Doses3
	Baseline	Baseline	Baseline	Group Specific	Group Specific	Data	Data
1	Human_physiology_ADAM	Clarithromycin	Human_phys			Dose_IV_mg	Dose_PO_milligram
2	Human_physiology_ADAM	Clarithromycin	Human_phys			Dose_IV_mg	Dose_PO_milligram
3	Human_physiology_ADAM	Clarithromycin	Human_phys			Dose_IV_mg	Dose_PO_milligram
4	Human_physiology_ADAM	Clarithromycin	Human_phys			Dose_IV_mg	Dose_PO_milligram
5	Human_physiology_ADAM	Clarithromycin	Human_phys			Dose_IV_mg	Dose_PO_milligram
6	Human_physiology_ADAM	Clarithromycin	Human_phys			Dose_IV_mg	Dose_PO_milligram
7	Human_physiology_ADAM	Clarithromycin	Human_phys			Dose_IV_mg	Dose_PO_milligram
8	Human_physiology_ADAM	Clarithromycin	Human_phys			Dose_IV_mg	Dose_PO_milligram
9	Human_physiology_ADAM	Clarithromycin	Human_phys			Dose_IV_mg	Dose_PO_milligram
10	Human_physiology_ADAM	Clarithromycin	Human_phys			Dose_IV_mg	Dose_PO_milligram

Fit Step

Estimated Parameters (Pooled Fit)

Name	Transformation	Initial Untransformed Value	Untransformed Bounds
diff_baso	log	11.9	[4 400]

Error Model

Use one common error model for all responses: exponential

Algorithm Settings

Property	Value
EstimationFcn	scattersearch
MaxIterations	400
FunctionTolerance	1e-08
MaxStallIterations	50
MaxTime	Inf
NumInitialPoints	auto
NumTrialPoints	auto
XTolerance	1e-06
LocalSolver	lsqnonlin

Local Solver Settings

Property	Value
StepTolerance	1e-08
FunctionTolerance	1e-08
OptimalityTolerance	1e-06
MaxIterations	400

Report generated by SimBiology v. 25.1 (R2025a) on 13-Sep-2025 12:25:26

Talinolol_IV_PO_Fitting

Talinolol full PBPK with M-ADAM model

Model: PBPK_MADAM

Quantities

	Quantity Name	Type	Scope	Value	Initial Value	Units
1	Main_compartment	compartment	PBPK_MADAM	1	1	liter
2	Bile_drug	species	Main_compartment	0	0	milligram
3	Venous	compartment	Main_compartment	1	3.598	liter
4	Venous_drug	species	Venous	0	0	milligram/liter
5	Lung	compartment	Main_compartment	1	0.56	liter
6	Lung_drug	species	Lung	0	0	milligram/liter
7	Kidney	compartment	Main_compartment	1	0.35	liter
8	Kidney_drug	species	Kidney	0	0	milligram/liter
9	Brain	compartment	Main_compartment	1	1.47	liter
10	Brain_drug	species	Brain	0	0	milligram/liter
11	Muscle	compartment	Main_compartment	1	29.12	liter
12	Muscle_drug	species	Muscle	0	0	milligram/liter
13	Adipose	compartment	Main_compartment	1	13.79	liter
14	Adipose_drug	species	Adipose	0	0	milligram/liter
15	Heart	compartment	Main_compartment	1	0.35	liter
16	Heart_drug	species	Heart	0	0	milligram/liter
17	Skin	compartment	Main_compartment	1	2.87	liter
18	Skin_drug	species	Skin	0	0	milligram/liter
19	Bone	compartment	Main_compartment	1	11.06	liter
20	Bone_drug	species	Bone	0	0	milligram/liter
21	Rest	compartment	Main_compartment	1	7	liter
22	Rest_drug	species	Rest	0	0	milligram/liter
23	Artery	compartment	Main_compartment	1	1.799	liter
24	Artery_drug	species	Artery	0	0	milligram/liter
25	Gut	compartment	Main_compartment	1	1.26	liter
26	Gut_drug	species	Gut	0	0	milligram/liter

	Quantity Name	Type	Scope	Value	Initial Value	Units
27	Spleen	compartment	Main_compartment	1	0.21	liter
28	Spleen_drug	species	Spleen	0	0	milligram/liter
29	Liver_EC_S1	compartment	Main_compartment	1	0.0686	liter
30	Liver_EC_S1_drug	species	Liver_EC_S1	0	0	milligram/liter
31	Liver_EC_S2	compartment	Main_compartment	1	0.0686	liter
32	Liver_EC_S2_drug	species	Liver_EC_S2	0	0	milligram/liter
33	Liver_EC_S3	compartment	Main_compartment	1	0.0686	liter
34	Liver_EC_S3_drug	species	Liver_EC_S3	0	0	milligram/liter
35	Liver_EC_S4	compartment	Main_compartment	1	0.0686	liter
36	Liver_EC_S4_drug	species	Liver_EC_S4	0	0	milligram/liter
37	Liver_EC_S5	compartment	Main_compartment	1	0.0686	liter
38	Liver_EC_S5_drug	species	Liver_EC_S5	0	0	milligram/liter
39	Liver_IC_S5	compartment	Main_compartment	1	0.252	liter
40	Liver_IC_S5_drug	species	Liver_IC_S5	0	0	milligram/liter
41	Liver_IC_S4	compartment	Main_compartment	1	0.252	liter
42	Liver_IC_S4_drug	species	Liver_IC_S4	0	0	milligram/liter
43	Liver_IC_S3	compartment	Main_compartment	1	0.252	liter
44	Liver_IC_S3_drug	species	Liver_IC_S3	0	0	milligram/liter
45	Liver_IC_S1	compartment	Main_compartment	1	0.252	liter
46	Liver_IC_S1_drug	species	Liver_IC_S1	0	0	milligram/liter
47	Liver_IC_S2	compartment	Main_compartment	1	0.252	liter
48	Liver_IC_S2_drug	species	Liver_IC_S2	0	0	milligram/liter
49	Metabolites	compartment	Main_compartment	1	1	liter
50	Metabolites_drug	species	Metabolites	0	0	milligram
51	Testes	compartment	Main_compartment	1	0.07	liter

	Quantity Name	Type	Scope	Value	Initial Value	Units
52	Testes_drug	species	Testes	0	0	milligram/liter
53	Blood_total	compartment	Main_compartment	1	1	liter
54	Blood_total_drug	species	Blood_total	0	0	milligram/liter
55	Plasma_total	compartment	Main_compartment	1	1	liter
56	Plasma_total_drug	species	Plasma_total	0	0	nanogram/milliliter
57	Plasma_free_uM	species	Plasma_total	0	0	micromole/liter
58	Plasma_total_uM	species	Plasma_total	0	0	micromole/liter
59	Portal	compartment	Main_compartment	1	1	liter
60	Portal_drug	species	Portal	0	0	milligram/liter
61	Mass_Balance	compartment	Main_compartment	1	1	liter
62	Amount_body	species	Mass_Balance	0	0	milligram
63	Amount_total	species	Mass_Balance	0	0	milligram
64	Urine	compartment	Main_compartment	1	1	liter
65	Urine_drug	species	Urine	0	0	milligram
66	Liver_total	compartment	Main_compartment	1	1	liter
67	Liver_tissue_total	species	Liver_total	0	0	milligram/liter
68	Liver_blood_total	species	Liver_total	0	0	milligram/liter
69	Liver_blood_free	species	Liver_total	0	0	milligram/liter
70	Liver_tissue_free	species	Liver_total	0	0	milligram/liter
71	Liver_tissue_total_uM	species	Liver_total	0	0	micromole/liter
72	convert_to_nmole_per_kg	compartment	Main_compartment	1	1	liter
73	Venous_nmole	species	convert_to_nmole_per_kg	0	0	nanomole
74	Artery_nmole	species	convert_to_nmole_per_kg	0	0	nanomole
75	Bone_nmole	species	convert_to_nmole_per_kg	0	0	nanomole
76	Adipose_nmole	species	convert_to_nmole_per_kg	0	0	nanomole
77	Muscle_nmole	species	convert_to_nmole_per_kg	0	0	nanomole

	Quantity Name	Type	Scope	Value	Initial Value	Units
78	Urine_nmole	species	convert_to_nmole_per_kg	0	0	nanomole
79	Liver_EC1_nmole	species	convert_to_nmole_per_kg	0	0	nanomole
80	Liver_IC1_nmole	species	convert_to_nmole_per_kg	0	0	nanomole
81	Bile_nmole	species	convert_to_nmole_per_kg	0	0	nanomole
82	Kidney_nmole	species	convert_to_nmole_per_kg	0	0	nanomole
83	Lung_nmole	species	convert_to_nmole_per_kg	0	0	nanomole
84	Metabolites_nmole	species	convert_to_nmole_per_kg	0	0	nanomole
85	Liver_IC2_nmole	species	convert_to_nmole_per_kg	0	0	nanomole
86	Liver_EC2_nmole	species	convert_to_nmole_per_kg	0	0	nanomole
87	Liver_EC3_nmole	species	convert_to_nmole_per_kg	0	0	nanomole
88	Liver_IC3_nmole	species	convert_to_nmole_per_kg	0	0	nanomole
89	Liver_IC4_nmole	species	convert_to_nmole_per_kg	0	0	nanomole
90	Liver_EC4_nmole	species	convert_to_nmole_per_kg	0	0	nanomole
91	Liver_EC5_nmole	species	convert_to_nmole_per_kg	0	0	nanomole
92	Liver_IC5_nmole	species	convert_to_nmole_per_kg	0	0	nanomole
93	Gut_nmole	species	convert_to_nmole_per_kg	0	0	nanomole
94	Spleen_nmole	species	convert_to_nmole_per_kg	0	0	nanomole
95	Skin_nmole	species	convert_to_nmole_per_kg	0	0	nanomole
96	Brain_nmole	species	convert_to_nmole_per_kg	0	0	nanomole
97	Rest_nmole	species	convert_to_nmole_per_kg	0	0	nanomole
98	Heart_nmole	species	convert_to_nmole_per_kg	0	0	nanomole
99	Main_compartment_1	compartment	PBPK_MADAM	1	1	liter
100	Bile_drug_1	species	Main_compartment_1	0	0	milligram
101	Venous_1	compartment	Main_compartment_1	1	3.598	liter
102	Venous_drug_1	species	Venous_1	0	0	milligram/liter

	Quantity Name	Type	Scope	Value	Initial Value	Units
103	Lung_1	compartment	Main_compartment_1	1	0.56	liter
104	Lung_drug_1	species	Lung_1	0	0	milligram/liter
105	Kidney_1	compartment	Main_compartment_1	1	0.35	liter
106	Kidney_drug_1	species	Kidney_1	0	0	milligram/liter
107	Brain_1	compartment	Main_compartment_1	1	1.47	liter
108	Brain_drug_1	species	Brain_1	0	0	milligram/liter
109	Muscle_1	compartment	Main_compartment_1	1	29.12	liter
110	Muscle_drug_1	species	Muscle_1	0	0	milligram/liter
111	Adipose_1	compartment	Main_compartment_1	1	13.79	liter
112	Adipose_drug_1	species	Adipose_1	0	0	milligram/liter
113	Heart_1	compartment	Main_compartment_1	1	0.35	liter
114	Heart_drug_1	species	Heart_1	0	0	milligram/liter
115	Skin_1	compartment	Main_compartment_1	1	2.87	liter
116	Skin_drug_1	species	Skin_1	0	0	milligram/liter
117	Bone_1	compartment	Main_compartment_1	1	11.06	liter
118	Bone_drug_1	species	Bone_1	0	0	milligram/liter
119	Rest_1	compartment	Main_compartment_1	1	7	liter
120	Rest_drug_1	species	Rest_1	0	0	milligram/liter
121	Artery_1	compartment	Main_compartment_1	1	1.799	liter
122	Artery_drug_1	species	Artery_1	0	0	milligram/liter
123	Gut_1	compartment	Main_compartment_1	1	1.26	liter
124	Gut_drug_1	species	Gut_1	0	0	milligram/liter
125	Spleen_1	compartment	Main_compartment_1	1	0.21	liter
126	Spleen_drug_1	species	Spleen_1	0	0	milligram/liter
127	Liver_EC_S1_1	compartment	Main_compartment_1	1	0.0686	liter
128	Liver_EC_S1_drug_1	species	Liver_EC_S1_1	0	0	milligram/liter
129	Liver_EC_S2_1	compartment	Main_compartment_1	1	0.0686	liter

	Quantity Name	Type	Scope	Value	Initial Value	Units
130	Liver_EC_S2_drug_1	species	Liver_EC_S2_1	0	0	milligram/liter
131	Liver_EC_S3_1	compartment	Main_compartment_1	1	0.0686	liter
132	Liver_EC_S3_drug_1	species	Liver_EC_S3_1	0	0	milligram/liter
133	Liver_EC_S4_1	compartment	Main_compartment_1	1	0.0686	liter
134	Liver_EC_S4_drug_1	species	Liver_EC_S4_1	0	0	milligram/liter
135	Liver_EC_S5_1	compartment	Main_compartment_1	1	0.0686	liter
136	Liver_EC_S5_drug_1	species	Liver_EC_S5_1	0	0	milligram/liter
137	Liver_IC_S5_1	compartment	Main_compartment_1	1	0.252	liter
138	Liver_IC_S5_drug_1	species	Liver_IC_S5_1	0	0	milligram/liter
139	Liver_IC_S3_1	compartment	Main_compartment_1	1	0.252	liter
140	Liver_IC_S3_drug_1	species	Liver_IC_S3_1	0	0	milligram/liter
141	Liver_IC_S1_1	compartment	Main_compartment_1	1	0.252	liter
142	Liver_IC_S1_drug_1	species	Liver_IC_S1_1	0	0	milligram/liter
143	Liver_IC_S2_1	compartment	Main_compartment_1	1	0.252	liter
144	Liver_IC_S2_drug_1	species	Liver_IC_S2_1	0	0	milligram/liter
145	Metabolites_1	compartment	Main_compartment_1	1	1	liter
146	Metabolites_drug_1	species	Metabolites_1	0	0	milligram
147	Testes_1	compartment	Main_compartment_1	1	0.07	liter
148	Testes_drug_1	species	Testes_1	0	0	milligram/liter
149	Blood_total_1	compartment	Main_compartment_1	1	1	liter
150	Blood_total_drug_1	species	Blood_total_1	0	0	milligram/liter
151	Plasma_total_1	compartment	Main_compartment_1	1	1	liter
152	Plasma_total_drug_1	species	Plasma_total_1	0	0	milligram/liter
153	Plasma_total_uM_1	species	Plasma_total_1	0	0	micromole/liter
154	Plasma_free_uM_1	species	Plasma_total_1	0	0	micromole/liter

	Quantity Name	Type	Scope	Value	Initial Value	Units
155	Portal_1	compartment	Main_compartment_1	1	1	liter
156	Portal_drug_1	species	Portal_1	0	0	milligram/liter
157	Portal_plasma_drug_1	species	Portal_1	0	0	milligram/liter
158	Portal_plasma_drug	species	Portal_1	0	0	milligram/liter
159	Mass_Balance_1	compartment	Main_compartment_1	1	1	liter
160	Amount_body_1	species	Mass_Balance_1	0	0	milligram
161	Amount_total_1	species	Mass_Balance_1	0	0	milligram
162	Urine_1	compartment	Main_compartment_1	1	1	liter
163	Urine_drug_1	species	Urine_1	0	0	milligram
164	Liver_IC_S4_1	compartment	Main_compartment_1	1	0.252	liter
165	Liver_IC_S4_drug_1	species	Liver_IC_S4_1	0	0	milligram/liter
166	Liver_total_1	compartment	Main_compartment_1	1	1	liter
167	Liver_tissue_free_uM_1	species	Liver_total_1	0	0	micromole/liter
168	Liver_tissue_total_1	species	Liver_total_1	0	0	milligram/liter
169	Liver_blood_free_1	species	Liver_total_1	0	0	milligram/liter
170	Liver_tissue_total_uM_1	species	Liver_total_1	0	0	micromole/liter
171	Liver_blood_total_1	species	Liver_total_1	0	0	milligram/liter
172	convert_to_nmole_per_kg_1	compartment	Main_compartment_1	1	1	liter
173	Venous_nmole_1	species	convert_to_nmole_per_kg_1	0	0	nanomole
174	Artery_nmole_1	species	convert_to_nmole_per_kg_1	0	0	nanomole
175	Bone_nmole_1	species	convert_to_nmole_per_kg_1	0	0	nanomole
176	Adipose_nmole_1	species	convert_to_nmole_per_kg_1	0	0	nanomole
177	Muscle_nmole_1	species	convert_to_nmole_per_kg_1	0	0	nanomole

	Quantity Name	Type	Scope	Value	Initial Value	Units
178	Urine_nmole_1	species	convert_to_nmole_per_kg_1	0	0	nanomole
179	Liver_EC1_nmole_1	species	convert_to_nmole_per_kg_1	0	0	nanomole
180	Liver_IC1_nmole_1	species	convert_to_nmole_per_kg_1	0	0	nanomole
181	Bile_nmole_1	species	convert_to_nmole_per_kg_1	0	0	nanomole
182	Kidney_nmole_1	species	convert_to_nmole_per_kg_1	0	0	nanomole
183	Lung_nmole_1	species	convert_to_nmole_per_kg_1	0	0	nanomole
184	Metabolites_nmole_1	species	convert_to_nmole_per_kg_1	0	0	nanomole
185	Liver_IC2_nmole_1	species	convert_to_nmole_per_kg_1	0	0	nanomole
186	Liver_EC2_nmole_1	species	convert_to_nmole_per_kg_1	0	0	nanomole
187	Liver_EC3_nmole_1	species	convert_to_nmole_per_kg_1	0	0	nanomole
188	Liver_IC3_nmole_1	species	convert_to_nmole_per_kg_1	0	0	nanomole
189	Liver_IC4_nmole_1	species	convert_to_nmole_per_kg_1	0	0	nanomole
190	Liver_EC4_nmole_1	species	convert_to_nmole_per_kg_1	0	0	nanomole
191	Liver_EC5_nmole_1	species	convert_to_nmole_per_kg_1	0	0	nanomole
192	Liver_IC5_nmole_1	species	convert_to_nmole_per_kg_1	0	0	nanomole
193	Gut_nmole_1	species	convert_to_nmole_per_kg_1	0	0	nanomole
194	Spleen_nmole_1	species	convert_to_nmole_per_kg_1	0	0	nanomole
195	Skin_nmole_1	species	convert_to_nmole_per_kg_1	0	0	nanomole

	Quantity Name	Type	Scope	Value	Initial Value	Units
196	Brain_nmole_1	species	convert_to_nmole_per_kg_1	0	0	nanomole
197	Rest_nmole_1	species	convert_to_nmole_per_kg_1	0	0	nanomole
198	Heart_nmole_1	species	convert_to_nmole_per_kg_1	0	0	nanomole
199	Gut_Lumen	compartment	Main_compartment	1	1	liter
200	Gut_Lumen_drug	species	Gut_Lumen	0	0	milligram
201	Gut_Lumen_1	compartment	Main_compartment_1	1	1	liter
202	Gut_Lumen_drug_1	species	Gut_Lumen_1	0	0	milligram
203	Eliminated	compartment	Main_compartment	1	1	milliliter
204	AMT_ELIM_DUO	species	Eliminated	0	0	microgram
205	AMT_ELIM_JEJ1	species	Eliminated	0	0	microgram
206	AMT_ELIM_JEJ2	species	Eliminated	0	0	microgram
207	AMT_ELIM_ILL1	species	Eliminated	0	0	microgram
208	AMT_ELIM_ILL2	species	Eliminated	0	0	microgram
209	AMT_ELIM_ILL3	species	Eliminated	0	0	microgram
210	AMT_ELIM_ILL4	species	Eliminated	0	0	microgram
211	AMT_ELIM_gut	species	Eliminated	0	0	microgram
212	STOMACH	compartment	Main_compartment	0.147	0.05	liter
213	X_STOMACH_SOLID	species	STOMACH	0	0	microgram
214	X_STOMACH_DISS	species	STOMACH	0	0	microgram
215	VDUO	compartment	Main_compartment	1	0.03435	liter
216	X_DUO_SOLID	species	VDUO	0	0	microgram
217	X_DUO_DISS	species	VDUO	0	0	microgram
218	VJEJ1	compartment	Main_compartment	1	0.0211	liter

	Quantity Name	Type	Scope	Value	Initial Value	Units
219	X_JEJ1_SOLID	species	VJEJ1	0	0	microgram
220	X_JEJ1_DISS	species	VJEJ1	0	0	microgram
221	MDUO	compartment	Main_compartment	1	0.037454	liter
222	MEM_DUO	species	MDUO	0	0	microgram
223	MJEJ1	compartment	Main_compartment	1	0.073785	liter
224	MEM_JEJ1	species	MJEJ1	0	0	microgram
225	VJEJ2	compartment	Main_compartment	1	0.0211	liter
226	X_JEJ2_SOLID	species	VJEJ2	0	0	microgram
227	X_JEJ2_DISS	species	VJEJ2	0	0	microgram
228	MJEJ2	compartment	Main_compartment	1	0.051687	liter
229	MEM_JEJ2	species	MJEJ2	0	0	microgram
230	VILL1	compartment	Main_compartment	1	0.0126	liter
231	X_ILL1_SOLID	species	VILL1	0	0	microgram
232	X_ILL1_DISS	species	VILL1	0	0	microgram
233	MILL1	compartment	Main_compartment	1	0.0412	liter
234	MEM_ILL1	species	MILL1	0	0	microgram
235	VILL2	compartment	Main_compartment	1	0.0126	liter
236	X_ILL2_SOLID	species	VILL2	0	0	microgram
237	X_ILL2_DISS	species	VILL2	0	0	microgram
238	MILL2	compartment	Main_compartment	1	0.0412	liter
239	MEM_ILL2	species	MILL2	0	0	microgram
240	VILL3	compartment	Main_compartment	1	0.0126	liter
241	X_ILL3_SOLID	species	VILL3	0	0	microgram
242	X_ILL3_DISS	species	VILL3	0	0	microgram
243	MILL3	compartment	Main_compartment	1	0.04045	liter
244	MEM_ILL3	species	MILL3	0	0	microgram
245	VILL4	compartment	Main_compartment	1	0.0126	liter
246	X_ILL4_SOLID	species	VILL4	0	0	microgram

	Quantity Name	Type	Scope	Value	Initial Value	Units
247	X_ILL4_DISS	species	VILL4	0	0	microgram
248	MILL4	compartment	Main_compartment	1	0.038952	liter
249	MEM_ILL4	species	MILL4	0	0	microgram
250	Colon	compartment	Main_compartment	1	1	liter
251	X_CECUM_SOLID	species	Colon	0	0	microgram
252	X_CECUM_DISS	species	Colon	0	0	microgram
253	VillousDUO	compartment	Main_compartment	1	0.0057016	liter
254	Villous_DUO	species	VillousDUO	0	0	microgram
255	VillousJEJ1	compartment	Main_compartment	1	0.011232	liter
256	Villous_JEJ1	species	VillousJEJ1	0	0	microgram
257	VillousJEJ2	compartment	Main_compartment	1	0.0078682	liter
258	Villous_JEJ2	species	VillousJEJ2	0	0	microgram
259	VillousILL1	compartment	Main_compartment	1	0.0062718	liter
260	Villous_ILL1	species	VillousILL1	0	0	microgram
261	VillousILL2	compartment	Main_compartment	1	0.0062718	liter
262	Villous_ILL2	species	VillousILL2	0	0	microgram
263	VillousILL3	compartment	Main_compartment	1	0.0061578	liter
264	Villous_ILL3	species	VillousILL3	0	0	microgram
265	VillousILL4	compartment	Main_compartment	1	0.0059297	liter
266	Villous_ILL4	species	VillousILL4	0	0	microgram
267	Liver	compartment	Main_compartment	1	1.603	liter
268	Liver_drug	species	Liver	0	0	milligram/liter
269	Metabolites_Liver	compartment	Main_compartment	1	1	liter
270	Metabolites_liver_drug	species	Metabolites_Liver	0	0	milligram
271	Serosa	compartment	Main_compartment	1	0.14284	liter
272	Serosa_drug	species	Serosa	0	0	milligram/liter
273	Enterocyte_conc_calc	compartment	Main_compartment	1	1	liter
274	memduo_conc	species	Enterocyte_conc_calc	0	0	micromole/liter
275	memjej1_conc	species	Enterocyte_conc_calc	0	0	micromole/liter

	Quantity Name	Type	Scope	Value	Initial Value	Units
276	memjej2_conc	species	Enterocyte_conc_calc	0	0	micromole/liter
277	memill1_conc	species	Enterocyte_conc_calc	0	0	micromole/liter
278	memill2_conc	species	Enterocyte_conc_calc	0	0	micromole/liter
279	memill3_conc	species	Enterocyte_conc_calc	0	0	micromole/liter
280	memill4_conc	species	Enterocyte_conc_calc	0	0	micromole/liter
281	Villous_conc_calc	compartment	Main_compartment	1	1	liter
282	vduo_conc	species	Villous_conc_calc	0	0	micromole/liter
283	vjej1_conc	species	Villous_conc_calc	0	0	micromole/liter
284	vjej2_conc	species	Villous_conc_calc	0	0	micromole/liter
285	vill1_conc	species	Villous_conc_calc	0	0	micromole/liter
286	vill2_conc	species	Villous_conc_calc	0	0	micromole/liter
287	vill3_conc	species	Villous_conc_calc	0	0	micromole/liter
288	vill4_conc	species	Villous_conc_calc	0	0	micromole/liter
289	Qlung	parameter	PBPK_MADAM	1	336	liter/hour
290	k_lung_artery	parameter	PBPK_MADAM	1	36.7032	liter/hour
291	k_venous_lung	parameter	PBPK_MADAM	1	336	liter/hour
292	Qkidney	parameter	PBPK_MADAM	1	63	liter/hour
293	k_artery_kidney	parameter	PBPK_MADAM	1	63	liter/hour
294	Qbrain	parameter	PBPK_MADAM	1	42	liter/hour
295	k_artery_brain	parameter	PBPK_MADAM	1	42	liter/hour
296	Qmuscle	parameter	PBPK_MADAM	1	58.8	liter/hour
297	k_artery_muscle	parameter	PBPK_MADAM	1	58.8	liter/hour
298	Qadipose	parameter	PBPK_MADAM	1	16.8	liter/hour
299	k_artery_adipos	parameter	PBPK_MADAM	1	16.8	liter/hour
300	Qskin	parameter	PBPK_MADAM	1	16.8	liter/hour

	Quantity Name	Type	Scope	Value	Initial Value	Units
301	k_artery_skin	parameter	PBPK_MADAM	1	16.8	liter/hour
302	Qbone	parameter	PBPK_MADAM	1	16.8	liter/hour
303	k_artery_bone	parameter	PBPK_MADAM	1	16.8	liter/hour
304	Qrest	parameter	PBPK_MADAM	1	4.2	liter/hour
305	k_artery_rest	parameter	PBPK_MADAM	1	4.2	liter/hour
306	k_kidney_venous	parameter	PBPK_MADAM	1	12.9165	liter/hour
307	k_brain_venous	parameter	PBPK_MADAM	1	17.8786	liter/hour
308	k_muscle_venous	parameter	PBPK_MADAM	1	11.7407	liter/hour
309	k_adipos_venous	parameter	PBPK_MADAM	1	8.9689	liter/hour
310	Qheart	parameter	PBPK_MADAM	1	12.6	liter/hour
311	k_heart_venous	parameter	PBPK_MADAM	1	2.2437	liter/hour
312	k_skin_venous	parameter	PBPK_MADAM	1	5.8911	liter/hour
313	k_bone_venous	parameter	PBPK_MADAM	1	6.8667	liter/hour
314	k_rest_venous	parameter	PBPK_MADAM	1	0.92605	liter/hour
315	Q_artery_spleen	parameter	PBPK_MADAM	1	8.4	liter/hour
316	k_artery_spleen	parameter	PBPK_MADAM	1	8.4	liter/hour
317	Q_artery_gut	parameter	PBPK_MADAM	1	71.4	liter/hour
318	k_artery_gut	parameter	PBPK_MADAM	1	71.4	liter/hour
319	Q_artery_liver	parameter	PBPK_MADAM	1	4.2	liter/hour
320	Q_spleen_liver	parameter	PBPK_MADAM	1	8.4	liter/hour
321	k_spleen_liver	parameter	PBPK_MADAM	1	1.5414	liter/hour
322	Q_gut_liver	parameter	PBPK_MADAM	1	71.4	liter/hour
323	k_gut_liver	parameter	PBPK_MADAM	1	12.4663	liter/hour
324	k_Liver_EC_S1_Liver_IC_S1	parameter	PBPK_MADAM	1	1369.0662	liter/hour

	Quantity Name	Type	Scope	Value	Initial Value	Units
325	k_Liver_IC_S1_Liver_EC_S1	parameter	PBPK_MADAM	0	1.1398	liter/hour
326	k_Liver_EC_S2_Liver_IC_S2	parameter	PBPK_MADAM	1	1369.0662	liter/hour
327	k_Liver_IC_S2_Liver_EC_S2	parameter	PBPK_MADAM	0	1.1398	liter/hour
328	k_Liver_IC_S4_Liver_EC_S4	parameter	PBPK_MADAM	0	1.1398	liter/hour
329	k_Liver_EC_S4_Liver_IC_S4	parameter	PBPK_MADAM	1	1369.0662	liter/hour
330	k_Liver_IC_S5_Liver_EC_S5	parameter	PBPK_MADAM	0	1.1398	liter/hour
331	k_Liver_EC_S5_Liver_IC_S5	parameter	PBPK_MADAM	1	1369.0662	liter/hour
332	k_Liver_EC_S3_Liver_IC_S3	parameter	PBPK_MADAM	1	1369.0662	liter/hour
333	Q_li	parameter	PBPK_MADAM	1	84	liter/hour
334	k_Liver_EC_S1_Liver_EC_S2	parameter	PBPK_MADAM	1	84	liter/hour
335	k_Liver_EC_S2_Liver_EC_S3	parameter	PBPK_MADAM	1	84	liter/hour
336	k_Liver_EC_S3_Liver_EC_S4	parameter	PBPK_MADAM	1	84	liter/hour
337	k_Liver_EC_S4_Liver_EC_S5	parameter	PBPK_MADAM	1	84	liter/hour
338	k_Liver_IC_S5_Bile	parameter	PBPK_MADAM	1	0.26268	liter/hour
339	k_Liver_IC_S4_Bile	parameter	PBPK_MADAM	1	0.26268	liter/hour
340	k_Liver_IC_S3_Bile	parameter	PBPK_MADAM	1	0.26268	liter/hour
341	k_Liver_IC_S2_Bile	parameter	PBPK_MADAM	1	0.26268	liter/hour
342	k_Liver_IC_S1_Bile	parameter	PBPK_MADAM	1	0.26268	liter/hour
343	k_Liver_IC_S1_Metabolites	parameter	PBPK_MADAM	1	0	liter/hour
344	k_Liver_IC_S2_Metabolites	parameter	PBPK_MADAM	1	0	liter/hour

	Quantity Name	Type	Scope	Value	Initial Value	Units
345	k_Liver_IC_S3_Metabolites	parameter	PBPK_MADAM	1	0	liter/hour
346	k_Liver_IC_S4_Metabolites	parameter	PBPK_MADAM	1	0	liter/hour
347	k_Liver_IC_S5_Metabolites	parameter	PBPK_MADAM	1	0	liter/hour
348	drug_fB	parameter	PBPK_MADAM	1	0.47872	dimensionless
349	drug_fuLiver	parameter	PBPK_MADAM	0.018	0.077212	dimensionless
350	Kp_kidney	parameter	PBPK_MADAM	0.134	4.5849	dimensionless
351	drug_BRP	parameter	PBPK_MADAM	0.65	0.94	dimensionless
352	Kp_heart	parameter	PBPK_MADAM	0.16	5.2789	dimensionless
353	Kp_gut	parameter	PBPK_MADAM	0.165	5.3838	dimensionless
354	Kp_brain	parameter	PBPK_MADAM	0.057	2.2082	dimensionless
355	Kp_bone	parameter	PBPK_MADAM	0.108	2.2998	dimensionless
356	Kp_adipose	parameter	PBPK_MADAM	0.047	1.7608	dimensionless
357	Kp_muscle	parameter	PBPK_MADAM	0.038	4.7077	dimensionless
358	Kp_rest	parameter	PBPK_MADAM	0.12	4.2633	dimensionless
359	Kp_lung	parameter	PBPK_MADAM	0.21	8.6052	dimensionless
360	drug_CLrenal	parameter	PBPK_MADAM	0	18.66	liter/hour
361	drug_fuplasma	parameter	PBPK_MADAM	0.0382	0.45	dimensionless
362	Kp_spleen	parameter	PBPK_MADAM	0.1	5.1227	dimensionless
363	k_Liver_Venous	parameter	PBPK_MADAM	1	9.1758	liter/hour
364	k_artery_heart	parameter	PBPK_MADAM	1	12.6	liter/hour
365	k_Liver_IC_S3_Liver__EC_S3	parameter	PBPK_MADAM	0	1.1398	liter/hour
366	k_artery_liver	parameter	PBPK_MADAM	1	4.2	liter/hour
367	drug_PSinfg	parameter	PBPK_MADAM	471	471	microliter/minute
368	phys_HPGL	parameter	PBPK_MADAM	122	125	1/gram

	Quantity Name	Type	Scope	Value	Initial Value	Units
369	switch_SFinf	parameter	PBPK_MADAM	3.196	3.196	dimensionless
370	Specific_volume	parameter	PBPK_MADAM	1	1	milliliter/gram
371	drug_PSbileg	parameter	PBPK_MADAM	2.5	1.8	microliter/minute
372	switch_SFbile	parameter	PBPK_MADAM	0.23114	1	dimensionless
373	drug_PSdifg	parameter	PBPK_MADAM	5	5	microliter/minute
374	switch_SFdiff	parameter	PBPK_MADAM	1.5621	1.5621	dimensionless
375	drug_CLmetg	parameter	PBPK_MADAM	19	0	microliter/minute
376	drug_HLM_CLint	parameter	PBPK_MADAM	0	0	milliliter/minute/milligram
377	phys_MPGL	parameter	PBPK_MADAM	45	45	milligram/gram
378	drug_fumic	parameter	PBPK_MADAM	1	0.49786	dimensionless
379	drug_funic	parameter	PBPK_MADAM	1	0.49786	dimensionless
380	Kp_skin	parameter	PBPK_MADAM	0.28	2.6806	dimensionless
381	phys_BW	parameter	PBPK_MADAM	1	70	kilogram
382	switch_SFrenal	parameter	PBPK_MADAM	1	1	dimensionless
383	k_venous_urine_CLR	parameter	PBPK_MADAM	1	18.66	liter/hour
384	Qtestes	parameter	PBPK_MADAM	1	0	liter/hour
385	k_artery_testes	parameter	PBPK_MADAM	1	0	liter/hour
386	Kp_testes	parameter	PBPK_MADAM	1	1	dimensionless
387	k_testes_venous	parameter	PBPK_MADAM	1	0	liter/hour
388	k_Liver_IC_S2_Liver_EC_S2_efflux	parameter	PBPK_MADAM	1	0	liter/hour
389	k_Liver_IC_S3_Liver_EC_S3_efflux	parameter	PBPK_MADAM	1	0	liter/hour
390	k_Liver_IC_S4_Liver_EC_S4_efflux	parameter	PBPK_MADAM	1	0	liter/hour
391	k_Liver_IC_S5_Liver_EC_S5_efflux	parameter	PBPK_MADAM	1	0	liter/hour

	Quantity Name	Type	Scope	Value	Initial Value	Units
392	k_Liver_IC_S1_Liver_EC_S1_efflux	parameter	PBPK_MADAM	1	0	liter/hour
393	drug_fa	parameter	PBPK_MADAM	1	1	dimensionless
394	drug_dose_rate_IV	parameter	PBPK_MADAM	1	15	milligram/hour
395	phys_Normalized_weight_adipose	parameter	PBPK_MADAM	92	197	gram/kilogram
396	phys_Normalized_weight_lung	parameter	PBPK_MADAM	5	8	gram/kilogram
397	phys_Normalized_weight_kidney	parameter	PBPK_MADAM	4	5	gram/kilogram
398	phys_Normalized_weight_brain	parameter	PBPK_MADAM	21	21	gram/kilogram
399	phys_Normalized_weight_muscle	parameter	PBPK_MADAM	409	416	gram/kilogram
400	phys_Normalized_weight_heart	parameter	PBPK_MADAM	4	5	gram/kilogram
401	phys_Normalized_weight_skin	parameter	PBPK_MADAM	85	41	gram/kilogram
402	phys_Normalized_weight_bone	parameter	PBPK_MADAM	203	158	gram/kilogram
403	phys_Normalized_weight_remainder	parameter	PBPK_MADAM	100	100	gram/kilogram
404	phys_Normalized_weight_spleen	parameter	PBPK_MADAM	2	3	gram/kilogram
405	phys_Normalized_weight_gut	parameter	PBPK_MADAM	47	18	gram/kilogram
406	phys_Normalized_weight_liver_blood	parameter	PBPK_MADAM	5	4.9	gram/kilogram
407	phys_Normalized_weight_liver_tissue	parameter	PBPK_MADAM	20	18	gram/kilogram
408	phys_Normalized_weight_artery	parameter	PBPK_MADAM	22.4	25.7	gram/kilogram
409	phys_Normalized_weight_venous	parameter	PBPK_MADAM	45.2	51.4	gram/kilogram

	Quantity Name	Type	Scope	Value	Initial Value	Units
410	phys_Normalized_weight_testes	parameter	PBPK_MADAM	1	1	gram/kilogram
411	phys_Normalized_Q_adipose	parameter	PBPK_MADAM	15	4	milliliter/minute/kilogram
412	phys_Normalized_Q_lung	parameter	PBPK_MADAM	200	80	milliliter/minute/kilogram
413	phys_Normalized_Q_brain	parameter	PBPK_MADAM	11	10	milliliter/minute/kilogram
414	phys_Normalized_Q_muscle	parameter	PBPK_MADAM	65	14	milliliter/minute/kilogram
415	phys_Normalized_Q_heart	parameter	PBPK_MADAM	12	3	milliliter/minute/kilogram
416	phys_Normalized_Q_bone	parameter	PBPK_MADAM	19	4	milliliter/minute/kilogram
417	phys_Normalized_Q_remainder	parameter	PBPK_MADAM	1	1	milliliter/minute/kilogram
418	phys_Normalized_Q_gut	parameter	PBPK_MADAM	17	17	milliliter/minute/kilogram
419	phys_Normalized_Q_spleen	parameter	PBPK_MADAM	2	2	milliliter/minute/kilogram
420	phys_Normalized_Q_liver	parameter	PBPK_MADAM	26	20	milliliter/minute/kilogram
421	phys_Normalized_Q_kidney	parameter	PBPK_MADAM	25	15	milliliter/minute/kilogram
422	phys_Normalized_Q_skin	parameter	PBPK_MADAM	20	4	milliliter/minute/kilogram
423	phys_Normalized_Q_testes	parameter	PBPK_MADAM	0	0	milliliter/minute/kilogram
424	switch_SFmet	parameter	PBPK_MADAM	0.32509	1	dimensionless
425	drug_Km_uptake	parameter	PBPK_MADAM	1	1	micromole/liter
426	switch_Vmax_uptake	parameter	PBPK_MADAM	0	0	micromole/kilogram/hour
427	drug_molar_mass	parameter	PBPK_MADAM	712800	363494	milligram/mole
428	drug_Km_met	parameter	PBPK_MADAM	1	1	micromole/liter
429	switch_Vmax_met	parameter	PBPK_MADAM	0	0	micromole/hour/kilogram
430	drug_Kp_adipose_raw	parameter	PBPK_MADAM	0.45	1.7608	dimensionless
431	switch_SFKp	parameter	PBPK_MADAM	0.51837	1	dimensionless

	Quantity Name	Type	Scope	Value	Initial Value	Units
432	drug_Kp_bone_raw	parameter	PBPK_MADAM	0.6	2.2998	dimensionless
433	drug_Kp_brain_raw	parameter	PBPK_MADAM	0.32	2.2082	dimensionless
434	drug_Kp_gut_raw	parameter	PBPK_MADAM	0.47	5.3838	dimensionless
435	drug_Kp_heart_raw	parameter	PBPK_MADAM	0.2	5.2789	dimensionless
436	drug_Kp_kidney_raw	parameter	PBPK_MADAM	0.26	4.5849	dimensionless
437	drug_Kp_lung_raw	parameter	PBPK_MADAM	0.4	8.6052	dimensionless
438	drug_Kp_muscle_raw	parameter	PBPK_MADAM	0.05	4.7077	dimensionless
439	drug_Kp_rest_raw	parameter	PBPK_MADAM	0.0065	4.2633	dimensionless
440	drug_Kp_skin_raw	parameter	PBPK_MADAM	0.37	2.6806	dimensionless
441	drug_Kp_spleen_raw	parameter	PBPK_MADAM	0.35	5.1227	dimensionless
442	drug_Kp_testes_raw	parameter	PBPK_MADAM	1	1	dimensionless
443	k_transit	parameter	PBPK_MADAM	0	0	1/(hour)
444	drug_t_lag	parameter	PBPK_MADAM	1	1	hour
445	drug_k_oral	parameter	PBPK_MADAM	3.381	3.381	1/(hour)
446	switch_SFeff	parameter	PBPK_MADAM	0	0	dimensionless
447	nanomole_per_mole	parameter	PBPK_MADAM	1000000000	1000000000	nanomole/mole
448	kilogram	parameter	PBPK_MADAM	1	1	kilogram
449	k_venous_urine_GFR	parameter	PBPK_MADAM	1	0	liter/hour
450	drug_GFR	parameter	PBPK_MADAM	1	0	milliliter/minute
451	drug_CLeffluxHep	parameter	PBPK_MADAM	0	0	microliter/minute
452	drug_FR	parameter	PBPK_MADAM	0	0	dimensionless
453	switch_slow_dist_Kp	parameter	PBPK_MADAM	1	1	dimensionless
454	Qlung_1	parameter	PBPK_MADAM	1	336	liter/hour
455	k_lung_artery_1	parameter	PBPK_MADAM	1	1053.3017	liter/hour
456	k_venous_lung_1	parameter	PBPK_MADAM	1	336	liter/hour

	Quantity Name	Type	Scope	Value	Initial Value	Units
457	Qkidney_1	parameter	PBPK_MADAM	1	63	liter/hour
458	k_artery_kidney_1	parameter	PBPK_MADAM	1	63	liter/hour
459	Qbrain_1	parameter	PBPK_MADAM	1	42	liter/hour
460	k_artery_brain_1	parameter	PBPK_MADAM	1	42	liter/hour
461	Qmuscle_1	parameter	PBPK_MADAM	1	58.8	liter/hour
462	k_artery_muscle_1	parameter	PBPK_MADAM	1	58.8	liter/hour
463	Qadipose_1	parameter	PBPK_MADAM	1	16.8	liter/hour
464	k_artery_adipos_1	parameter	PBPK_MADAM	1	16.8	liter/hour
465	Qskin_1	parameter	PBPK_MADAM	1	16.8	liter/hour
466	k_artery_skin_1	parameter	PBPK_MADAM	1	16.8	liter/hour
467	Qbone_1	parameter	PBPK_MADAM	1	16.8	liter/hour
468	k_artery_bone_1	parameter	PBPK_MADAM	1	16.8	liter/hour
469	Qrest_1	parameter	PBPK_MADAM	1	4.2	liter/hour
470	k_artery_rest_1	parameter	PBPK_MADAM	1	4.2	liter/hour
471	k_kidney_venous_1	parameter	PBPK_MADAM	1	303.837	liter/hour
472	k_brain_venous_1	parameter	PBPK_MADAM	1	164.5784	liter/hour
473	k_muscle_venous_1	parameter	PBPK_MADAM	1	1474.6224	liter/hour
474	k_adipos_venous_1	parameter	PBPK_MADAM	1	46.8134	liter/hour
475	Qheart_1	parameter	PBPK_MADAM	1	12.6	liter/hour
476	k_heart_venous_1	parameter	PBPK_MADAM	1	78.9976	liter/hour
477	k_skin_venous_1	parameter	PBPK_MADAM	1	56.9352	liter/hour
478	k_bone_venous_1	parameter	PBPK_MADAM	1	35.1101	liter/hour
479	k_rest_venous_1	parameter	PBPK_MADAM	1	420	liter/hour
480	Q_artery_spleen_1	parameter	PBPK_MADAM	1	8.4	liter/hour
481	k_artery_spleen_1	parameter	PBPK_MADAM	1	8.4	liter/hour

	Quantity Name	Type	Scope	Value	Initial Value	Units
482	Q_artery_gut_1	parameter	PBPK_MADAM	1	71.4	liter/hour
483	k_artery_gut_1	parameter	PBPK_MADAM	1	71.4	liter/hour
484	Q_artery_liver_1	parameter	PBPK_MADAM	1	4.2	liter/hour
485	Q_spleen_liver_1	parameter	PBPK_MADAM	1	8.4	liter/hour
486	k_spleen_liver_1	parameter	PBPK_MADAM	1	30.0943	liter/hour
487	Q_gut_liver_1	parameter	PBPK_MADAM	1	71.4	liter/hour
488	k_gut_liver_1	parameter	PBPK_MADAM	1	190.4907	liter/hour
489	k_Liver_EC_S1_Liver_IC_S1_1	parameter	PBPK_MADAM	1	168.0698	liter/hour
490	k_Liver_IC_S1_Liver_EC_S1_1	parameter	PBPK_MADAM	0	0.26572	liter/hour
491	k_Liver_EC_S2_Liver_IC_S2_1	parameter	PBPK_MADAM	1	168.0698	liter/hour
492	k_Liver_IC_S2_Liver_EC_S2_1	parameter	PBPK_MADAM	0	0.26572	liter/hour
493	k_Liver_IC_S4_Liver_EC_S4_1	parameter	PBPK_MADAM	0	0.26572	liter/hour
494	k_Liver_EC_S4_Liver_IC_S4_1	parameter	PBPK_MADAM	1	168.0698	liter/hour
495	k_Liver_IC_S5_Liver_EC_S5_1	parameter	PBPK_MADAM	0	0.26572	liter/hour
496	k_Liver_EC_S5_Liver_IC_S5_1	parameter	PBPK_MADAM	1	168.0698	liter/hour
497	k_Liver_EC_S3_Liver_IC_S3_1	parameter	PBPK_MADAM	1	168.0698	liter/hour
498	Q_li_1	parameter	PBPK_MADAM	1	84	liter/hour
499	k_Liver_EC_S1_Liver_EC_S2_1	parameter	PBPK_MADAM	1	84	liter/hour
500	k_Liver_EC_S2_Liver_EC_S3_1	parameter	PBPK_MADAM	1	84	liter/hour
501	k_Liver_EC_S3_Liver_EC_S4_1	parameter	PBPK_MADAM	1	84	liter/hour

	Quantity Name	Type	Scope	Value	Initial Value	Units
502	k_Liver_EC_S4_Liver_EC_S5_1	parameter	PBPK_MADAM	1	84	liter/hour
503	k_Liver_IC_S5_Bile_1	parameter	PBPK_MADAM	1	0.019658	liter/hour
504	k_Liver_IC_S4_Bile_1	parameter	PBPK_MADAM	1	0.019658	liter/hour
505	k_Liver_IC_S3_Bile_1	parameter	PBPK_MADAM	1	0.019658	liter/hour
506	k_Liver_IC_S2_Bile_1	parameter	PBPK_MADAM	1	0.019658	liter/hour
507	k_Liver_IC_S1_Bile_1	parameter	PBPK_MADAM	1	0.019658	liter/hour
508	k_Liver_IC_S1_Metabolites_1	parameter	PBPK_MADAM	1	0.21013	liter/hour
509	k_Liver_IC_S2_Metabolites_1	parameter	PBPK_MADAM	1	0.21013	liter/hour
510	k_Liver_IC_S3_Metabolites_1	parameter	PBPK_MADAM	1	0.21013	liter/hour
511	k_Liver_IC_S4_Metabolites_1	parameter	PBPK_MADAM	1	0.21013	liter/hour
512	k_Liver_IC_S5_Metabolites_1	parameter	PBPK_MADAM	1	0.21013	liter/hour
513	drug_fB_1	parameter	PBPK_MADAM	1	0.058769	dimensionless
514	drug_fuLiver_1	parameter	PBPK_MADAM	0.018	0.018	dimensionless
515	Kp_kidney_1	parameter	PBPK_MADAM	0.134	0.13478	dimensionless
516	drug_BRP_1	parameter	PBPK_MADAM	0.65	0.65	dimensionless
517	Kp_heart_1	parameter	PBPK_MADAM	0.16	0.10367	dimensionless
518	Kp_gut_1	parameter	PBPK_MADAM	0.165	0.24363	dimensionless
519	Kp_brain_1	parameter	PBPK_MADAM	0.057	0.16588	dimensionless
520	Kp_bone_1	parameter	PBPK_MADAM	0.108	0.31102	dimensionless
521	Kp_adipose_1	parameter	PBPK_MADAM	0.047	0.23327	dimensionless
522	Kp_muscle_1	parameter	PBPK_MADAM	0.038	0.025919	dimensionless
523	Kp_rest_1	parameter	PBPK_MADAM	0.12	0.0065	dimensionless

	Quantity Name	Type	Scope	Value	Initial Value	Units
524	Kp_lung_1	parameter	PBPK_MADAM	0.21	0.20735	dimensionless
525	drug_CLrenal_1	parameter	PBPK_MADAM	0	0	liter/hour
526	drug_fuplasma_1	parameter	PBPK_MADAM	0.0382	0.0382	dimensionless
527	Kp_spleen_1	parameter	PBPK_MADAM	0.1	0.18143	dimensionless
528	k_Liver_EC_S5_Venous_1	parameter	PBPK_MADAM	1	84	liter/hour
529	k_artery_heart_1	parameter	PBPK_MADAM	1	12.6	liter/hour
530	k_Liver_IC_S3_Liver__EC_S3_1	parameter	PBPK_MADAM	0	0.26572	liter/hour
531	k_artery_liver_1	parameter	PBPK_MADAM	1	4.2	liter/hour
532	drug_PSinfg_1	parameter	PBPK_MADAM	471	471	microliter/minute
533	phys_HPGL_1	parameter	PBPK_MADAM	122	122	1/gram
534	switch_SFinf_1	parameter	PBPK_MADAM	3.196	3.196	dimensionless
535	drug_PSbileg_1	parameter	PBPK_MADAM	2.5	2.5	microliter/minute
536	switch_SFbile_1	parameter	PBPK_MADAM	0.23114	0.23114	dimensionless
537	drug_PSdifg_1	parameter	PBPK_MADAM	5	5	microliter/minute
538	switch_SFdiff_1	parameter	PBPK_MADAM	1.5621	1.5621	dimensionless
539	drug_CLmetg_1	parameter	PBPK_MADAM	19	19	microliter/minute
540	drug_HLM_CLint_1	parameter	PBPK_MADAM	0	0	milliliter/minute/milligram
541	phys_MPGL_1	parameter	PBPK_MADAM	45	45	milligram/gram
542	drug_fumic_1	parameter	PBPK_MADAM	1	1	dimensionless
543	drug_funic_1	parameter	PBPK_MADAM	1	1	dimensionless
544	Kp_skin_1	parameter	PBPK_MADAM	0.28	0.1918	dimensionless
545	switch_SFrenal_1	parameter	PBPK_MADAM	1	1	dimensionless
546	k_venous_urine_CLR_1	parameter	PBPK_MADAM	1	0	liter/hour
547	Qtestes_1	parameter	PBPK_MADAM	1	0	liter/hour

	Quantity Name	Type	Scope	Value	Initial Value	Units
548	k_artery_testes_1	parameter	PBPK_MADAM	1	0	liter/hour
549	Kp_testes_1	parameter	PBPK_MADAM	1	0.51837	dimensionless
550	k_testes_venous_1	parameter	PBPK_MADAM	1	0	liter/hour
551	k_Liver_IC_S2_Liver_EC_S2_efflux_1	parameter	PBPK_MADAM	1	0	liter/hour
552	k_Liver_IC_S3_Liver_EC_S3_efflux_1	parameter	PBPK_MADAM	1	0	liter/hour
553	k_Liver_IC_S4_Liver_EC_S4_efflux_1	parameter	PBPK_MADAM	1	0	liter/hour
554	k_Liver_IC_S5_Liver_EC_S5_efflux_1	parameter	PBPK_MADAM	1	0	liter/hour
555	k_Liver_IC_S1_Liver_EC_S1_efflux_1	parameter	PBPK_MADAM	1	0	liter/hour
556	drug_fa_1	parameter	PBPK_MADAM	1	1	dimensionless
557	drug_dose_rate_IV_1	parameter	PBPK_MADAM	1	1	milligram/hour
558	switch_SFmet_1	parameter	PBPK_MADAM	0.32509	0.32509	dimensionless
559	drug_Km_uptake_1	parameter	PBPK_MADAM	1	1	micromole/liter
560	switch_Vmax_uptake_1	parameter	PBPK_MADAM	0	0	micromole/kilogram/hour
561	drug_molar_mass_1	parameter	PBPK_MADAM	712800	712800	milligram/mole
562	drug_Km_met_1	parameter	PBPK_MADAM	1	1	micromole/liter
563	switch_Vmax_met_1	parameter	PBPK_MADAM	0	0	micromole/hour/kilogram
564	drug_Kp_adipose_raw_1	parameter	PBPK_MADAM	0.45	0.45	dimensionless
565	switch_SFKp_1	parameter	PBPK_MADAM	0.51837	0.51837	dimensionless
566	drug_Kp_bone_raw_1	parameter	PBPK_MADAM	0.6	0.6	dimensionless
567	drug_Kp_brain_raw_1	parameter	PBPK_MADAM	0.32	0.32	dimensionless
568	drug_Kp_gut_raw_1	parameter	PBPK_MADAM	0.47	0.47	dimensionless
569	drug_Kp_heart_raw_1	parameter	PBPK_MADAM	0.2	0.2	dimensionless
570	drug_Kp_kidney_raw_1	parameter	PBPK_MADAM	0.26	0.26	dimensionless

	Quantity Name	Type	Scope	Value	Initial Value	Units
571	drug_Kp_lung_raw_1	parameter	PBPK_MADAM	0.4	0.4	dimensionless
572	drug_Kp_muscle_raw_1	parameter	PBPK_MADAM	0.05	0.05	dimensionless
573	drug_Kp_rest_raw_1	parameter	PBPK_MADAM	0.0065	0.0065	dimensionless
574	drug_Kp_skin_raw_1	parameter	PBPK_MADAM	0.37	0.37	dimensionless
575	drug_Kp_spleen_raw_1	parameter	PBPK_MADAM	0.35	0.35	dimensionless
576	drug_Kp_testes_raw_1	parameter	PBPK_MADAM	1	1	dimensionless
577	k_transit_1	parameter	PBPK_MADAM	0	0	1/(hour)
578	drug_k_oral_1	parameter	PBPK_MADAM	10	10	1/(hour)
579	switch_SFeff_1	parameter	PBPK_MADAM	0	0	dimensionless
580	k_venous_urine_GFR_1	parameter	PBPK_MADAM	1	0	liter/hour
581	drug_GFR_1	parameter	PBPK_MADAM	1	0	milliliter/minute
582	drug_CLeffluxHep_1	parameter	PBPK_MADAM	0	0	microliter/minute
583	drug_FR_1	parameter	PBPK_MADAM	0	0	dimensionless
584	switch_slow_dist_Kp_1	parameter	PBPK_MADAM	1	1	dimensionless
585	drug_uptake_Ki	parameter	PBPK_MADAM	0.226	0.226	micromole/liter
586	drug_dose_amount_IV	parameter	PBPK_MADAM	0	30	milligram
587	drug_dose_amount_PO_1	parameter	PBPK_MADAM	0	0	milligram
588	drug_dose_amount_IV_1	parameter	PBPK_MADAM	0	0	milligram
589	drug_dose_amount_PO	parameter	PBPK_MADAM	0	100	milligram
590	uptake_inhib_S1	parameter	PBPK_MADAM	1	1	dimensionless
591	uptake_inhib_S2	parameter	PBPK_MADAM	1	1	dimensionless
592	uptake_inhib_S3	parameter	PBPK_MADAM	1	1	dimensionless
593	uptake_inhib_S4	parameter	PBPK_MADAM	1	1	dimensionless
594	uptake_inhib_S5	parameter	PBPK_MADAM	1	1	dimensionless
595	switch_biliary_inhib_1	parameter	PBPK_MADAM	0	0	dimensionless

	Quantity Name	Type	Scope	Value	Initial Value	Units
596	switch_met_inhib_1	parameter	PBPK_MADAM	0	0	dimensionless
597	biliary_inhib_S1	parameter	PBPK_MADAM	1	1	dimensionless
598	biliary_inhib_S2	parameter	PBPK_MADAM	1	1	dimensionless
599	biliary_inhib_S3	parameter	PBPK_MADAM	1	1	dimensionless
600	biliary_inhib_S4	parameter	PBPK_MADAM	1	1	dimensionless
601	biliary_inhib_S5	parameter	PBPK_MADAM	1	1	dimensionless
602	switch_uptake_inhib_1	parameter	PBPK_MADAM	0	0	dimensionless
603	met_inhib_S1	parameter	PBPK_MADAM	1	1	dimensionless
604	met_inhib_S2	parameter	PBPK_MADAM	1	1	dimensionless
605	met_inhib_S3	parameter	PBPK_MADAM	1	1	dimensionless
606	met_inhib_S4	parameter	PBPK_MADAM	1	1	dimensionless
607	met_inhib_S5	parameter	PBPK_MADAM	1	1	dimensionless
608	drug_biliary_Ki	parameter	PBPK_MADAM	0.226	0.226	micromole/liter
609	drug_met_Ki	parameter	PBPK_MADAM	0.226	0.226	micromole/liter
610	drug_dose_IV_start_1	parameter	PBPK_MADAM	3	3	hour
611	drug_dose_IV_start	parameter	PBPK_MADAM	3	3	hour
612	Kpuu_Liver_1	parameter	PBPK_MADAM	1	NaN	dimensionless
613	drug_k_bile_deg	parameter	PBPK_MADAM	0	0	1/hour
614	drug_k_bile_deg_1	parameter	PBPK_MADAM	0	0	1/hour
615	TSTOMACH	parameter	PBPK_MADAM	15	16.2	minute
616	TDUO	parameter	PBPK_MADAM	15.6	9.384	minute
617	TJEJ1	parameter	PBPK_MADAM	56.4	35.292	minute
618	TJEJ2	parameter	PBPK_MADAM	42	35.292	minute
619	TILL1	parameter	PBPK_MADAM	34.8	31.008	minute
620	TILL2	parameter	PBPK_MADAM	25.2	31.008	minute

	Quantity Name	Type	Scope	Value	Initial Value	Units
621	TILL3	parameter	PBPK_MADAM	17.4	31.008	minute
622	TILL4	parameter	PBPK_MADAM	261	31.008	minute
623	QMUC	parameter	PBPK_MADAM	142.8571	170	milliliter/minute
624	SOLIF_STOMACH	parameter	PBPK_MADAM	1476.373	971137867.2533	milligram/liter
625	SOLIF_DUO	parameter	PBPK_MADAM	1476.373	12237.3111	milligram/liter
626	SOLIF_JEJ1	parameter	PBPK_MADAM	1476.373	9722.7884	milligram/liter
627	SOLIF_JEJ2	parameter	PBPK_MADAM	1476.373	7725.4321	milligram/liter
628	SOLIF_ILL1	parameter	PBPK_MADAM	1476.373	4878.6288	milligram/liter
629	SOLIF_ILL2	parameter	PBPK_MADAM	1476.373	3082.4174	milligram/liter
630	SOLIF_ILL3	parameter	PBPK_MADAM	1476.373	624.1564	milligram/liter
631	SOLIF_ILL4	parameter	PBPK_MADAM	1476.373	1550.5597	milligram/liter
632	DIFF	parameter	PBPK_MADAM	0.010286	120	centimeter^3/minute
633	NI_DUO	parameter	PBPK_MADAM	1	0.00093238	dimensionless
634	NI_JEJ1	parameter	PBPK_MADAM	1	0.0011735	dimensionless
635	NI_JEJ2	parameter	PBPK_MADAM	1	0.0014769	dimensionless
636	NI_ILL1	parameter	PBPK_MADAM	1	0.0023387	dimensionless
637	NI_ILL2	parameter	PBPK_MADAM	1	0.0037016	dimensionless
638	NI_ILL3	parameter	PBPK_MADAM	1	0.01828	dimensionless
639	NI_ILL4	parameter	PBPK_MADAM	1	0.0073586	dimensionless
640	KD	parameter	PBPK_MADAM	0.0002	0.0001306	liter/milligram/minute
641	Kpu_Gut	parameter	PBPK_MADAM	0.2	0.2	dimensionless
642	fu_mem	parameter	PBPK_MADAM	1	0.0772	dimensionless
643	CLINT_efflux_DUO	parameter	PBPK_MADAM	1	18.7873	milliliter/minute
644	CLINT_influx_DUO	parameter	PBPK_MADAM	1	0	milliliter/minute
645	CLINT_metabolic_DUO	parameter	PBPK_MADAM	1	1	milliliter/minute

	Quantity Name	Type	Scope	Value	Initial Value	Units
646	CLINT_efflux_JEJ1	parameter	PBPK_MADAM	1	37.0845	milliliter/minute
647	CLINT_influx_JEJ1	parameter	PBPK_MADAM	1	0	milliliter/minute
648	CLINT_metabolic_JEJ1	parameter	PBPK_MADAM	1	1	milliliter/minute
649	CLINT_efflux_JEJ2	parameter	PBPK_MADAM	1	25.9755	milliliter/minute
650	CLINT_influx_JEJ2	parameter	PBPK_MADAM	1	0	milliliter/minute
651	CLINT_metabolic_JEJ2	parameter	PBPK_MADAM	1	1	milliliter/minute
652	CLINT_efflux_ILL1	parameter	PBPK_MADAM	1	20.7477	milliliter/minute
653	CLINT_influx_ILL1	parameter	PBPK_MADAM	1	0	milliliter/minute
654	CLINT_metabolic_ILL1	parameter	PBPK_MADAM	1	1	milliliter/minute
655	CLINT_efflux_ILL2	parameter	PBPK_MADAM	1	20.7477	milliliter/minute
656	CLINT_influx_ILL2	parameter	PBPK_MADAM	1	0	milliliter/minute
657	CLINT_metabolic_ILL2	parameter	PBPK_MADAM	1	1	milliliter/minute
658	CLINT_efflux_ILL3	parameter	PBPK_MADAM	1	20.421	milliliter/minute
659	CLINT_influx_ILL3	parameter	PBPK_MADAM	1	0	milliliter/minute
660	CLINT_metabolic_ILL3	parameter	PBPK_MADAM	1	1	milliliter/minute
661	CLINT_efflux_ILL4	parameter	PBPK_MADAM	1	19.6042	milliliter/minute
662	CLINT_influx_ILL4	parameter	PBPK_MADAM	1	0	milliliter/minute
663	CLINT_metabolic_ILL4	parameter	PBPK_MADAM	1	1	milliliter/minute
664	BW_average	parameter	PBPK_MADAM	70	70	kilogram
665	liter_to_milliliter	parameter	PBPK_MADAM	1000	1000	milliliter/liter
666	phys_Normalized_weight_stomach	parameter	PBPK_MADAM	2.1	2.1	gram/kilogram
667	Q_Gut	parameter	PBPK_MADAM	1	1	liter/minute
668	QMUC_1	parameter	PBPK_MADAM	142.8571	142.8571	milliliter/minute
669	V_LUM_TOT	parameter	PBPK_MADAM	6543.215	126.95	milliliter

	Quantity Name	Type	Scope	Value	Initial Value	Units
670	V_ONECOMP	parameter	PBPK_MADAM	934.745	18.1357	milliliter
671	VGut	parameter	PBPK_MADAM	50	0.517	liter
672	V_MEM	parameter	PBPK_MADAM	7142.8571	73.857	milliliter
673	HHINT	parameter	PBPK_MADAM	1	108.1519	dimensionless
674	HHSTOMACH	parameter	PBPK_MADAM	1	85113804.8202	dimensionless
675	HHDUO	parameter	PBPK_MADAM	1	1072.5193	dimensionless
676	HHJEJ1	parameter	PBPK_MADAM	1	852.138	dimensionless
677	HHJEJ2	parameter	PBPK_MADAM	1	677.083	dimensionless
678	HHILL1	parameter	PBPK_MADAM	1	427.5795	dimensionless
679	HHILL2	parameter	PBPK_MADAM	1	270.1535	dimensionless
680	HHILL3	parameter	PBPK_MADAM	1	54.7032	dimensionless
681	HHILL4	parameter	PBPK_MADAM	1	135.8963	dimensionless
682	LOGP	parameter	PBPK_MADAM	1	3.466	dimensionless
683	MW	parameter	PBPK_MADAM	1	363494	microgram/micromole
684	CACO2AB	parameter	PBPK_MADAM	1e-06	1e-06	centimeter/second
685	CACO2BA	parameter	PBPK_MADAM	1e-06	1e-06	centimeter/second
686	HPeff_exp	parameter	PBPK_MADAM	0.0001	0.0001	centimeter/second
687	SOLWATER	parameter	PBPK_MADAM	1	1	milligram/liter
688	REFPHSOL	parameter	PBPK_MADAM	7.4	7.4	dimensionless
689	PSIZE	parameter	PBPK_MADAM	0.0005	0.0025	centimeter
690	PDENSITY	parameter	PBPK_MADAM	1000000	1200000	microgram/milliliter
691	DLT	parameter	PBPK_MADAM	0.003	0.003	centimeter
692	DIFFCOEFF	parameter	PBPK_MADAM	0.0001	0.0003918	centimeter^2/minute
693	LL	parameter	PBPK_MADAM	680	680	centimeter
694	LR	parameter	PBPK_MADAM	1.75	1.75	centimeter

	Quantity Name	Type	Scope	Value	Initial Value	Units
695	ESA	parameter	PBPK_MADAM	120000	120000	centimeter^2
696	LOGSR	parameter	PBPK_MADAM	3.02	4.8695	dimensionless
697	SOLBILE	parameter	PBPK_MADAM	368.8433	368.8433	milligram/liter
698	NATC	parameter	PBPK_MADAM	4	4	nanomole/liter
699	SOLFASSIF	parameter	PBPK_MADAM	1	1234	milligram/liter
700	SOLINT	parameter	PBPK_MADAM	1476.373	11.4099	milligram/liter
701	HPeff_est	parameter	PBPK_MADAM	0.0001	0.00011667	centimeter/second
702	numIntestinalCompartments	parameter	PBPK_MADAM	7	7	dimensionless
703	second_per_minute	parameter	PBPK_MADAM	60	60	second/minute
704	minute_per_hour	parameter	PBPK_MADAM	60	60	minute/hour
705	milligram_per_microgram	parameter	PBPK_MADAM	0.001	0.001	milligram/microgram
706	pHStomach	parameter	PBPK_MADAM	1	1.5	dimensionless
707	pHDuo	parameter	PBPK_MADAM	1	6.4	dimensionless
708	pHJej1	parameter	PBPK_MADAM	1	6.5	dimensionless
709	pHJej2	parameter	PBPK_MADAM	1	6.6	dimensionless
710	pHIll1	parameter	PBPK_MADAM	1	6.8	dimensionless
711	pHIll2	parameter	PBPK_MADAM	1	7	dimensionless
712	pHIll3	parameter	PBPK_MADAM	1	7.7	dimensionless
713	pHIll4	parameter	PBPK_MADAM	1	7.3	dimensionless
714	fu_blood	parameter	PBPK_MADAM	1	0.47872	dimensionless
715	DIFF_BASO	parameter	PBPK_MADAM	1	1	milliliter/minute
716	drug_Km_influx	parameter	PBPK_MADAM	1	1	micromole/liter
717	drug_Km_efflux	parameter	PBPK_MADAM	1	37	micromole/liter
718	Gut_EC_fraction	parameter	PBPK_MADAM	0.3719	0.3719	dimensionless

	Quantity Name	Type	Scope	Value	Initial Value	Units
719	Gut_IC_fraction	parameter	PBPK_MADAM	0.6281	0.6281	dimensionless
720	influx_factor_duo	parameter	PBPK_MADAM	1	1	dimensionless
721	influx_factor_jej1	parameter	PBPK_MADAM	1	1	dimensionless
722	influx_factor_jej2	parameter	PBPK_MADAM	1	1	dimensionless
723	influx_factor_ill1	parameter	PBPK_MADAM	1	1	dimensionless
724	influx_factor_ill2	parameter	PBPK_MADAM	1	1	dimensionless
725	influx_factor_ill3	parameter	PBPK_MADAM	1	1	dimensionless
726	influx_factor_ill4	parameter	PBPK_MADAM	1	1	dimensionless
727	efflux_factor_duo	parameter	PBPK_MADAM	1	0.23	dimensionless
728	efflux_factor_jej1	parameter	PBPK_MADAM	1	1	dimensionless
729	efflux_factor_jej2	parameter	PBPK_MADAM	1	1.44	dimensionless
730	efflux_factor_ill1	parameter	PBPK_MADAM	1	2.14	dimensionless
731	efflux_factor_ill2	parameter	PBPK_MADAM	1	2.14	dimensionless
732	efflux_factor_ill3	parameter	PBPK_MADAM	1	2.14	dimensionless
733	efflux_factor_ill4	parameter	PBPK_MADAM	1	2.14	dimensionless
734	switchVmax_influx	parameter	PBPK_MADAM	0	0	micromole/minute/centimeter^2
735	switchVmax_efflux	parameter	PBPK_MADAM	0	0.00016	micromole/minute/centimeter^2
736	Qmuc_DUO	parameter	PBPK_MADAM	1	26.9235	milliliter/minute
737	Qmuc_JEJ1	parameter	PBPK_MADAM	1	74.0397	milliliter/minute
738	Qmuc_JEJ2	parameter	PBPK_MADAM	1	74.0397	milliliter/minute
739	Qmuc_ILL1	parameter	PBPK_MADAM	1	32.7365	milliliter/minute
740	Qmuc_ILL2	parameter	PBPK_MADAM	1	32.7365	milliliter/minute
741	Qmuc_ILL3	parameter	PBPK_MADAM	1	32.7365	milliliter/minute
742	Qmuc_ILL4	parameter	PBPK_MADAM	1	32.7365	milliliter/minute
743	volumeRatio_DUO	parameter	PBPK_MADAM	0.1429	0.11534	dimensionless

	Quantity Name	Type	Scope	Value	Initial Value	Units
744	volumeRatio_JEJ1	parameter	PBPK_MADAM	0.1429	0.22722	dimensionless
745	volumeRatio_JEJ2	parameter	PBPK_MADAM	0.1429	0.15917	dimensionless
746	volumeRatio_ILL1	parameter	PBPK_MADAM	0.1429	0.12687	dimensionless
747	volumeRatio_ILL2	parameter	PBPK_MADAM	0.1429	0.12687	dimensionless
748	volumeRatio_ILL3	parameter	PBPK_MADAM	0.1429	0.12457	dimensionless
749	volumeRatio_ILL4	parameter	PBPK_MADAM	0.1429	0.11995	dimensionless
750	flowRatio_DUO	parameter	PBPK_MADAM	0.1429	0.088	dimensionless
751	flowRatio_JEJ1	parameter	PBPK_MADAM	0.1429	0.242	dimensionless
752	flowRatio_JEJ2	parameter	PBPK_MADAM	0.1429	0.242	dimensionless
753	flowRatio_ILL1	parameter	PBPK_MADAM	0.1429	0.107	dimensionless
754	flowRatio_ILL2	parameter	PBPK_MADAM	0.1429	0.107	dimensionless
755	flowRatio_ILL3	parameter	PBPK_MADAM	0.1429	0.107	dimensionless
756	flowRatio_ILL4	parameter	PBPK_MADAM	0.1429	0.107	dimensionless
757	switch_SFinflux	parameter	PBPK_MADAM	1	1	dimensionless
758	switch_SFefflux	parameter	PBPK_MADAM	1	1	dimensionless
759	switch_SFgutmet	parameter	PBPK_MADAM	1	1	dimensionless
760	switch_SFdiffapi	parameter	PBPK_MADAM	1	1	dimensionless
761	switch_SFdiffbaso	parameter	PBPK_MADAM	1	1	dimensionless
762	zero	parameter	PBPK_MADAM	0	0	micromole/minute/centimeter^2
763	CLINT_influx	parameter	PBPK_MADAM	1	1	milliliter/minute
764	CLINT_efflux	parameter	PBPK_MADAM	1	1	milliliter/minute
765	CLINT_metabolism	parameter	PBPK_MADAM	1	0	milliliter/minute
766	metabolism_factor_duo	parameter	PBPK_MADAM	1	1	dimensionless
767	metabolism_factor_jej1	parameter	PBPK_MADAM	1	1	dimensionless
768	metabolism_factor_jej2	parameter	PBPK_MADAM	1	1	dimensionless

	Quantity Name	Type	Scope	Value	Initial Value	Units
769	metabolism_factor_ill1	parameter	PBPK_MADAM	1	1	dimensionless
770	metabolism_factor_ill2	parameter	PBPK_MADAM	1	1	dimensionless
771	metabolism_factor_ill3	parameter	PBPK_MADAM	1	1	dimensionless
772	metabolism_factor_ill4	parameter	PBPK_MADAM	1	1	dimensionless
773	k_liver_metabolites	parameter	PBPK_MADAM	1	0	liter/hour
774	k_liver_bile	parameter	PBPK_MADAM	1	1.3134	liter/hour
775	Kp_liver	parameter	PBPK_MADAM	1	8.6052	dimensionless
776	drug_Kp_liver_raw	parameter	PBPK_MADAM	1.1	8.6052	dimensionless
777	phys_Normalized_Q_villi	parameter	PBPK_MADAM	4.8	4.8	milliliter/minute/kilogram
778	phys_Normalized_weight_enterocyte	parameter	PBPK_MADAM	7.3857	7.3857	gram/kilogram
779	LumenTotal	parameter	PBPK_MADAM	126.95	126.95	milliliter
780	lumenvolumeRatio_DUO	parameter	PBPK_MADAM	1	0.27058	dimensionless
781	lumenvolumeRatio_JEJ1	parameter	PBPK_MADAM	1	0.16621	dimensionless
782	lumenvolumeRatio_JEJ2	parameter	PBPK_MADAM	1	0.16621	dimensionless
783	lumenvolumeRatio_ILL1	parameter	PBPK_MADAM	1	0.099252	dimensionless
784	lumenvolumeRatio_ILL2	parameter	PBPK_MADAM	1	0.099252	dimensionless
785	lumenvolumeRatio_ILL3	parameter	PBPK_MADAM	1	0.099252	dimensionless
786	lumenvolumeRatio_ILL4	parameter	PBPK_MADAM	1	0.099252	dimensionless
787	Q_villi	parameter	PBPK_MADAM	1	336	milliliter/minute
788	StomachLumenTotal	parameter	PBPK_MADAM	50	50	milliliter
789	ESA_baso	parameter	PBPK_MADAM	1	6703	centimeter^2
790	DIFF_duo	parameter	PBPK_MADAM	1	96.6	centimeter^3/minute
791	DIFF_jej1	parameter	PBPK_MADAM	1	190.68	centimeter^3/minute
792	DIFF_jej2	parameter	PBPK_MADAM	1	133.56	centimeter^3/minute

	Quantity Name	Type	Scope	Value	Initial Value	Units
793	DIFF_ill1	parameter	PBPK_MADAM	1	106.68	centimeter^3/minute
794	DIFF_ill2	parameter	PBPK_MADAM	1	106.68	centimeter^3/minute
795	DIFF_ill3	parameter	PBPK_MADAM	1	105	centimeter^3/minute
796	DIFF_ill4	parameter	PBPK_MADAM	1	100.8	centimeter^3/minute
797	surfaceRatio_DUO	parameter	PBPK_MADAM	1	0.115	dimensionless
798	surfaceRatio_JEJ1	parameter	PBPK_MADAM	1	0.227	dimensionless
799	surfaceRatio_JEJ2	parameter	PBPK_MADAM	1	0.159	dimensionless
800	surfaceRatio_ILL1	parameter	PBPK_MADAM	1	0.127	dimensionless
801	surfaceRatio_ILL2	parameter	PBPK_MADAM	1	0.127	dimensionless
802	surfaceRatio_ILL3	parameter	PBPK_MADAM	1	0.125	dimensionless
803	surfaceRatio_ILL4	parameter	PBPK_MADAM	1	0.12	dimensionless
804	DIFF_BASO_duo	parameter	PBPK_MADAM	1	2.468	milliliter/minute
805	DIFF_BASO_jej1	parameter	PBPK_MADAM	1	9.5062	milliliter/minute
806	DIFF_BASO_jej2	parameter	PBPK_MADAM	1	9.5062	milliliter/minute
807	DIFF_BASO_ill1	parameter	PBPK_MADAM	1	6.3625	milliliter/minute
808	DIFF_BASO_ill2	parameter	PBPK_MADAM	1	6.3625	milliliter/minute
809	DIFF_BASO_ill3	parameter	PBPK_MADAM	1	6.3625	milliliter/minute
810	DIFF_BASO_ill4	parameter	PBPK_MADAM	1	6.3625	milliliter/minute
811	basoSurfaceRatio_DUO	parameter	PBPK_MADAM	1	0.0526	dimensionless
812	basoSurfaceRatio_JEJ1	parameter	PBPK_MADAM	1	0.2026	dimensionless
813	basoSurfaceRatio_JEJ2	parameter	PBPK_MADAM	1	0.2026	dimensionless
814	basoSurfaceRatio_ILL1	parameter	PBPK_MADAM	1	0.1356	dimensionless
815	basoSurfaceRatio_ILL2	parameter	PBPK_MADAM	1	0.1356	dimensionless
816	basoSurfaceRatio_ILL3	parameter	PBPK_MADAM	1	0.1356	dimensionless
817	basoSurfaceRatio_ILL4	parameter	PBPK_MADAM	1	0.1356	dimensionless

	Quantity Name	Type	Scope	Value	Initial Value	Units
818	drug_fQ	parameter	PBPK_MADAM	1	0.2571	dimensionless
819	k_artery_serosa	parameter	PBPK_MADAM	1	53.0431	liter/hour
820	k_serosa_liver	parameter	PBPK_MADAM	1	9.2612	liter/hour
821	Kp_serosa	parameter	PBPK_MADAM	1	5.3838	dimensionless
822	drug_Kp_serosa_raw	parameter	PBPK_MADAM	1	5.3838	dimensionless
823	pKA	parameter	PBPK_MADAM	9.3	9.43	dimensionless
824	CL_inf_api	parameter	PBPK_MADAM	1	0	microliter/minute/centimeter^2
825	CL_eff	parameter	PBPK_MADAM	1	1.3614	microliter/minute/centimeter^2
826	CLINT_influx_baso_DUO	parameter	PBPK_MADAM	1	0	milliliter/minute
827	CLINT_influx_baso_JEJ1	parameter	PBPK_MADAM	1	0	milliliter/minute
828	CLINT_influx_baso_JEJ2	parameter	PBPK_MADAM	1	0	milliliter/minute
829	CLINT_influx_baso_ILL1	parameter	PBPK_MADAM	1	0	milliliter/minute
830	CLINT_influx_baso_ILL2	parameter	PBPK_MADAM	1	0	milliliter/minute
831	CLINT_influx_baso_ILL3	parameter	PBPK_MADAM	1	0	milliliter/minute
832	CLINT_influx_baso_ILL4	parameter	PBPK_MADAM	1	0	milliliter/minute
833	diff_api	parameter	PBPK_MADAM	1	7	microliter/minute/centimeter^2
834	HPeff_est_baso	parameter	PBPK_MADAM	1	0.00011667	centimeter/second
835	diff_baso	parameter	PBPK_MADAM	1	7	microliter/minute/centimeter^2
836	CL_inf_baso	parameter	PBPK_MADAM	1	0	microliter/minute/centimeter^2
837	influx_factor_duo_baso	parameter	PBPK_MADAM	1	1	dimensionless
838	influx_factor_jej1_baso	parameter	PBPK_MADAM	1	1	dimensionless
839	influx_factor_jej2_baso	parameter	PBPK_MADAM	1	1	dimensionless
840	influx_factor_ill1_baso	parameter	PBPK_MADAM	1	1	dimensionless
841	influx_factor_ill2_baso	parameter	PBPK_MADAM	1	1	dimensionless
842	influx_factor_ill3_baso	parameter	PBPK_MADAM	1	1	dimensionless

	Quantity Name	Type	Scope	Value	Initial Value	Units
843	influx_factor_ill4_baso	parameter	PBPK_MADAM	1	1	dimensionless
844	switchVmax_efflux_Lower	parameter	PBPK_MADAM	0	0	micromole/minute/centimeter^2
845	delta	parameter	PBPK_MADAM	1	1	micromole/minute/centimeter^2
846	switch_SFefflux_Lower	parameter	PBPK_MADAM	150	4.18	dimensionless
847	SFefflux	parameter	PBPK_MADAM	4	4.18	dimensionless
848	CLINT_efflux_baso_DUO	parameter	PBPK_MADAM	1	0	milliliter/minute
849	CLINT_efflux_baso_JEJ1	parameter	PBPK_MADAM	1	0	milliliter/minute
850	CLINT_efflux_baso_JEJ2	parameter	PBPK_MADAM	1	0	milliliter/minute
851	CLINT_efflux_baso_ILL1	parameter	PBPK_MADAM	1	0	milliliter/minute
852	CLINT_efflux_baso_ILL2	parameter	PBPK_MADAM	1	0	milliliter/minute
853	CLINT_efflux_baso_ILL3	parameter	PBPK_MADAM	1	0	milliliter/minute
854	CLINT_efflux_baso_ILL4	parameter	PBPK_MADAM	1	0	milliliter/minute
855	switchVmax_efflux_baso	parameter	PBPK_MADAM	0.00016	0.11	micromole/minute/centimeter^2
856	switch_SFefflux_baso	parameter	PBPK_MADAM	1	1	dimensionless
857	drug_Km_efflux_baso	parameter	PBPK_MADAM	89	48.2	micromole/liter
858	switch_SFinflux_Lower	parameter	PBPK_MADAM	0	1	dimensionless
859	SFinflux	parameter	PBPK_MADAM	3	1	dimensionless
860	CL_eff_baso	parameter	PBPK_MADAM	1	0	microliter/minute/centimeter^2
861	baso_efflux_factor_duo	parameter	PBPK_MADAM	1	1	dimensionless
862	baso_efflux_factor_jej1	parameter	PBPK_MADAM	1	1	dimensionless
863	baso_efflux_factor_jej2	parameter	PBPK_MADAM	1	1	dimensionless
864	baso_efflux_factor_ill1	parameter	PBPK_MADAM	1	1	dimensionless
865	baso_efflux_factor_ill2	parameter	PBPK_MADAM	1	1	dimensionless
866	baso_efflux_factor_ill3	parameter	PBPK_MADAM	1	1	dimensionless
867	baso_efflux_factor_ill4	parameter	PBPK_MADAM	1	1	dimensionless

	Quantity Name	Type	Scope	Value	Initial Value	Units
868	HHMembrane	parameter	PBPK_MADAM	1	108.1519	dimensionless
869	HHVillous	parameter	PBPK_MADAM	1	170.8244	dimensionless
870	pHMembrane	parameter	PBPK_MADAM	7.4	7.4	dimensionless
871	pHVillous	parameter	PBPK_MADAM	7.2	7.2	dimensionless
872	NI_Membrane	parameter	PBPK_MADAM	1	0.0092463	dimensionless
873	NI_Villous	parameter	PBPK_MADAM	1	0.005854	dimensionless
874	switch_SFinflux_baso	parameter	PBPK_MADAM	1	1	dimensionless

Initial Assignments

	Initial Assignments	Initial Value
1	k_Liver_IC_S5_Bile = drug_PSbileg*switch_SFbile*phys_HPGL*(phys_BW*phys_Normalized_weight_liver_tissue*Specific_volume)/Specific_volume/5*drug_fuLiver	0.26268
2	k_Liver_IC_S4_Bile = drug_PSbileg*switch_SFbile*phys_HPGL*(phys_BW*phys_Normalized_weight_liver_tissue*Specific_volume)/Specific_volume/5*drug_fuLiver	0.26268
3	k_Liver_IC_S3_Bile = drug_PSbileg*switch_SFbile*phys_HPGL*(phys_BW*phys_Normalized_weight_liver_tissue*Specific_volume)/Specific_volume/5*drug_fuLiver	0.26268
4	k_Liver_IC_S2_Bile = drug_PSbileg*switch_SFbile*phys_HPGL*(phys_BW*phys_Normalized_weight_liver_tissue*Specific_volume)/Specific_volume/5*drug_fuLiver	0.26268
5	k_Liver_IC_S1_Bile = drug_PSbileg*switch_SFbile*phys_HPGL*(phys_BW*phys_Normalized_weight_liver_tissue*Specific_volume)/Specific_volume/5*drug_fuLiver	0.26268
6	k_Liver_EC_S4_Liver_EC_S5 = Q_li	84
7	k_Liver_EC_S3_Liver_EC_S4 = Q_li	84
8	k_Liver_EC_S2_Liver_EC_S3 = Q_li	84
9	k_Liver_EC_S1_Liver_EC_S2 = Q_li	84
10	k_Liver_IC_S5_Liver_EC_S5 = drug_PSdifg*switch_SFdiff*phys_HPGL*(phys_BW*phys_Normalized_weight_liver_tissue*Specific_volume)/Specific_volume/5*drug_fuLiver	1.1398
11	k_Liver_IC_S4_Liver_EC_S4 = drug_PSdifg*switch_SFdiff*phys_HPGL*(phys_BW*phys_Normalized_weight_liver_tissue*Specific_volume)/Specific_volume/5*drug_fuLiver	1.1398
12	k_Liver_IC_S2_Liver_EC_S2 = drug_PSdifg*switch_SFdiff*phys_HPGL*	1.1398

	Initial Assignments	Initial Value
	(phys_BW*phys_Normalized_weight_liver_tissue*Specific_volume)/Specific_volume/5*drug_fuLiver	
13	k_Liver_IC_S1_Liver_EC_S1 = drug_PSdifg*switch_SFdiff*phys_HPGL*(phys_BW*phys_Normalized_weight_liver_tissue*Specific_volume)/Specific_volume/5*drug_fuLiver	1.1398
14	k_rest_venous = Qrest/Kp_rest*drug_BRP	0.92605
15	k_artery_spleen = Q_artery_spleen	8.4
16	k_artery_gut = Q_artery_gut	71.4
17	k_gut_liver = Q_gut_liver/Kp_gut*drug_BRP	12.4663
18	k_spleen_liver = Q_spleen_liver/Kp_spleen*drug_BRP	1.5414
19	k_artery_rest = Qrest	4.2
20	k_kidney_venous = Qkidney/Kp_kidney*drug_BRP	12.9165
21	k_muscle_venous = Qmuscle/Kp_muscle*drug_BRP	11.7407
22	k_brain_venous = Qbrain/Kp_brain*drug_BRP	17.8786
23	k_skin_venous = Qskin/Kp_skin*drug_BRP	5.8911
24	k_bone_venous = Qbone/Kp_bone*drug_BRP	6.8667
25	k_heart_venous = Qheart/Kp_heart*drug_BRP	2.2437
26	k_adipos_venous = Qadipose/Kp_adipose*drug_BRP	8.9689
27	k_artery_muscle = Qmuscle	58.8
28	k_artery_adipos = Qadipose	16.8
29	k_artery_bone = Qbone	16.8
30	k_artery_skin = Qskin	16.8
31	k_artery_kidney = Qkidney	63
32	k_artery_brain = Qbrain	42
33	k_venous_lung = Qlung	336
34	k_lung_artery = Qlung/Kp_lung*drug_BRP	36.7032
35	k_artery_heart = Qheart	12.6
36	k_Liver_Venous = Q_li/Kp_liver*drug_BRP	9.1758
37	k_Liver_IC_S3_Liver__EC_S3 = drug_PSdifg*switch_SFdiff*phys_HPGL*(phys_BW*phys_Normalized_weight_liver_tissue*Specific_volume)/Specific_volume/5*drug_fuLiver	1.1398
38	k_artery_liver = Q_artery_liver	4.2
39	Venous = phys_BW*phys_Normalized_weight_venous*Specific_volume	3.598
40	Lung = phys_BW*phys_Normalized_weight_lung*Specific_volume	0.56
41	Kidney = phys_BW*phys_Normalized_weight_kidney*Specific_volume	0.35
42	Brain = phys_BW*phys_Normalized_weight_brain*Specific_volume	1.47
43	Muscle = phys_BW*phys_Normalized_weight_muscle*Specific_volu	29.12

	Initial Assignments	Initial Value
	me	
44	Adipose = phys_BW*phys_Normalized_weight_adipose*Specific_volume	13.79
45	Heart = phys_BW*phys_Normalized_weight_heart*Specific_volume	0.35
46	Skin = phys_BW*phys_Normalized_weight_skin*Specific_volume	2.87
47	Bone = phys_BW*phys_Normalized_weight_bone*Specific_volume	11.06
48	Rest = phys_BW*phys_Normalized_weight_remainder*Specific_volume	7
49	Artery = phys_BW*phys_Normalized_weight_artery*Specific_volume	1.799
50	Spleen = phys_BW*phys_Normalized_weight_spleen*Specific_volume	0.21
51	Gut = phys_BW*phys_Normalized_weight_gut*Specific_volume	1.26
52	Liver_EC_S1 = phys_BW*phys_Normalized_weight_liver_blood*Specific_volume/5	0.0686
53	Liver_EC_S2 = phys_BW*phys_Normalized_weight_liver_blood*Specific_volume/5	0.0686
54	Liver_EC_S3 = phys_BW*phys_Normalized_weight_liver_blood*Specific_volume/5	0.0686
55	Liver_EC_S4 = phys_BW*phys_Normalized_weight_liver_blood*Specific_volume/5	0.0686
56	Liver_EC_S5 = phys_BW*phys_Normalized_weight_liver_blood*Specific_volume/5	0.0686
57	Liver_IC_S5 = phys_BW*phys_Normalized_weight_liver_tissue*Specific_volume/5	0.252
58	Liver_IC_S4 = phys_BW*phys_Normalized_weight_liver_tissue*Specific_volume/5	0.252
59	Liver_IC_S3 = phys_BW*phys_Normalized_weight_liver_tissue*Specific_volume/5	0.252
60	Liver_IC_S2 = phys_BW*phys_Normalized_weight_liver_tissue*Specific_volume/5	0.252
61	Liver_IC_S1 = phys_BW*phys_Normalized_weight_liver_tissue*Specific_volume/5	0.252
62	Q_artery_gut = phys_BW*phys_Normalized_Q_gut	71.4

	Initial Assignments	Initial Value
63	Q_artery_liver = Q_li-Q_artery_gut-Q_artery_spleen	4.2
64	Q_artery_spleen = phys_BW*phys_Normalized_Q_spleen	8.4
65	Q_gut_liver = phys_BW*phys_Normalized_Q_gut	71.4
66	Q_spleen_liver = phys_BW*phys_Normalized_Q_spleen	8.4
67	Qadipose = phys_BW*phys_Normalized_Q_adipose	16.8
68	Qbone = phys_BW*phys_Normalized_Q_bone	16.8
69	Qbrain = phys_BW*phys_Normalized_Q_brain	42
70	Qheart = phys_BW*phys_Normalized_Q_heart	12.6
71	Qkidney = phys_BW*phys_Normalized_Q_kidney	63
72	Q_li = phys_BW*phys_Normalized_Q_liver	84
73	Qlung = phys_BW*phys_Normalized_Q_lung	336
74	Qmuscle = phys_BW*phys_Normalized_Q_muscle	58.8
75	Qrest = phys_BW*phys_Normalized_Q_remainder	4.2
76	Qskin = phys_BW*phys_Normalized_Q_skin	16.8
77	drug_fB = drug_fuplasma/drug_BRP	0.47872
78	k_venous_urine_CLR = drug_CLrenal*switch_SFrenal	18.66
79	k_artery_testes = Qtestes	0
80	k_testes_venous = Qtestes/Kp_testes*drug_BRP	0
81	Qtestes = phys_BW*phys_Normalized_Q_testes	0
82	k_Liver_IC_S2_Liver_EC_S2_efflux = switch_SFeff*drug_CLeffluxHep*phys_HPGL*(phys_BW*phys_Normalized_weight_liver_tissue*Specific_volume)/Specific_volume/5*drug_fuLiver	0
83	k_Liver_IC_S3_Liver_EC_S3_efflux = switch_SFeff*drug_CLeffluxHep*phys_HPGL*(phys_BW*phys_Normalized_weight_liver_tissue*Specific_volume)/Specific_volume/5*drug_fuLiver	0
84	k_Liver_IC_S4_Liver_EC_S4_efflux = switch_SFeff*drug_CLeffluxHep*phys_HPGL*(phys_BW*phys_Normalized_weight_liver_tissue*Specific_volume)/Specific_volume/5*drug_fuLiver	0
85	k_Liver_IC_S5_Liver_EC_S5_efflux = switch_SFeff*drug_CLeffluxHep*phys_HPGL*(phys_BW*phys_Normalized_weight_liver_tissue*Specific_volume)/Specific_volume/5*drug_fuLiver	0
86	k_Liver_IC_S1_Liver_EC_S1_efflux = switch_SFeff*drug_CLeffluxHep*phys_HPGL*(phys_BW*phys_Normalized_weight_liver_tissue*Specific_volume)/Specific_volume/5*drug_fuLiver	0
87	Testes = phys_BW*phys_Normalized_weight_testes*Specific_volume	0.07
88	Kp_adipose = drug_Kp_adipose_raw*switch_SFKp	1.7608
89	Kp_bone = drug_Kp_bone_raw*switch_SFKp	2.2998
90	Kp_brain = drug_Kp_brain_raw*switch_SFKp	2.2082
91	Kp_gut = drug_Kp_gut_raw*switch_SFKp	5.3838
92	Kp_heart = drug_Kp_heart_raw*switch_SFKp	5.2789

	Initial Assignments	Initial Value
93	Kp_kidney = drug_Kp_kidney_raw*switch_SFKp	4.5849
94	Kp_lung = drug_Kp_lung_raw*switch_SFKp	8.6052
95	Kp_muscle = drug_Kp_muscle_raw*switch_SFKp	4.7077
96	Kp_skin = drug_Kp_skin_raw*switch_SFKp	2.6806
97	Kp_spleen = drug_Kp_spleen_raw*switch_SFKp	5.1227
98	Kp_testes = drug_Kp_testes_raw*switch_SFKp	1
99	k_venous_urine_GFR = drug_GFR*drug_fuplasma*switch_SFrenal	0
100	drug_GFR = drug_FR*Qkidney	0
101	Kp_rest = switch_slow_dist_Kp*drug_Kp_rest_raw+(1-switch_slow_dist_Kp)*drug_Kp_rest_raw*switch_SFKp	4.2633
102	k_Liver_EC_S4_Liver_EC_S5_1 = Q_li_1	84
103	k_Liver_EC_S3_Liver_EC_S4_1 = Q_li_1	84
104	k_Liver_EC_S2_Liver_EC_S3_1 = Q_li_1	84
105	k_Liver_EC_S1_Liver_EC_S2_1 = Q_li_1	84
106	k_Liver_IC_S5_Liver_EC_S5_1 = drug_PSdifg_1*switch_SFdiff_1*phys_HPGL*(phys_BW*phys_Normalized_weight_liver_tissue*Specific_volume)/Specific_volume/5*drug_fuLiver_1	0.26572
107	k_Liver_IC_S4_Liver_EC_S4_1 = drug_PSdifg_1*switch_SFdiff_1*phys_HPGL*(phys_BW*phys_Normalized_weight_liver_tissue*Specific_volume)/Specific_volume/5*drug_fuLiver_1	0.26572
108	k_Liver_IC_S2_Liver_EC_S2_1 = drug_PSdifg_1*switch_SFdiff_1*phys_HPGL*(phys_BW*phys_Normalized_weight_liver_tissue*Specific_volume)/Specific_volume/5*drug_fuLiver_1	0.26572
109	k_Liver_IC_S1_Liver_EC_S1_1 = drug_PSdifg_1*switch_SFdiff_1*phys_HPGL*(phys_BW*phys_Normalized_weight_liver_tissue*Specific_volume)/Specific_volume/5*drug_fuLiver_1	0.26572
110	k_rest_venous_1 = Qrest_1/Kp_rest_1*drug_BRP_1	420
111	k_artery_spleen_1 = Q_artery_spleen_1	8.4
112	k_artery_gut_1 = Q_artery_gut_1	71.4
113	k_gut_liver_1 = Q_gut_liver_1/Kp_gut_1*drug_BRP_1	190.4907
114	k_spleen_liver_1 = Q_spleen_liver_1/Kp_spleen_1*drug_BRP_1	30.0943
115	k_artery_rest_1 = Qrest_1	4.2
116	k_kidney_venous_1 = Qkidney_1/Kp_kidney_1*drug_BRP_1	303.837
117	k_muscle_venous_1 = Qmuscle_1/Kp_muscle_1*drug_BRP_1	1474.6224
118	k_brain_venous_1 = Qbrain_1/Kp_brain_1*drug_BRP_1	164.5784
119	k_skin_venous_1 = Qskin_1/Kp_skin_1*drug_BRP_1	56.9352
120	k_bone_venous_1 = Qbone_1/Kp_bone_1*drug_BRP_1	35.1101
121	k_heart_venous_1 = Qheart_1/Kp_heart_1*drug_BRP_1	78.9976
122	k_adipos_venous_1 = Qadipose_1/Kp_adipose_1*drug_BRP_1	46.8134

	Initial Assignments	Initial Value
123	k_artery_muscle_1 = Qmuscle_1	58.8
124	k_artery_adipos_1 = Qadipose_1	16.8
125	k_artery_bone_1 = Qbone_1	16.8
126	k_artery_skin_1 = Qskin_1	16.8
127	k_artery_kidney_1 = Qkidney_1	63
128	k_artery_brain_1 = Qbrain_1	42
129	k_venous_lung_1 = Qlung_1	336
130	k_lung_artery_1 = Qlung_1/Kp_lung_1*drug_BRP_1	1053.3017
131	k_artery_heart_1 = Qheart_1	12.6
132	k_Liver_EC_S5_Venous_1 = Q_li_1	84
133	k_Liver_IC_S3_Liver__EC_S3_1 = drug_PSdifg_1*switch_SFdiff_1*phys_HPGL*(phys_BW*phys_Normalized_weight_liver_tissue*Specific_volume)/Specific_volume/5*drug_fuLiver_1	0.26572
134	k_artery_liver_1 = Q_artery_liver_1	4.2
135	Venous_1 = phys_BW*phys_Normalized_weight_venous*Specific_volume	3.598
136	Lung_1 = phys_BW*phys_Normalized_weight_lung*Specific_volume	0.56
137	Kidney_1 = phys_BW*phys_Normalized_weight_kidney*Specific_volume	0.35
138	Brain_1 = phys_BW*phys_Normalized_weight_brain*Specific_volume	1.47
139	Muscle_1 = phys_BW*phys_Normalized_weight_muscle*Specific_volume	29.12
140	Adipose_1 = phys_BW*phys_Normalized_weight_adipose*Specific_volume	13.79
141	Heart_1 = phys_BW*phys_Normalized_weight_heart*Specific_volume	0.35
142	Skin_1 = phys_BW*phys_Normalized_weight_skin*Specific_volume	2.87
143	Bone_1 = phys_BW*phys_Normalized_weight_bone*Specific_volume	11.06
144	Rest_1 = phys_BW*phys_Normalized_weight_remainder*Specific_volume	7
145	Artery_1 = phys_BW*phys_Normalized_weight_artery*Specific_volume	1.799
146	Spleen_1 = phys_BW*phys_Normalized_weight_spleen*Specific_volume	0.21
147	Gut_1 = phys_BW*phys_Normalized_weight_gut*Specific_volume	1.26

	Initial Assignments	Initial Value
148	Liver_EC_S1_1 = phys_BW*phys_Normalized_weight_liver_blood*Specific_volume/5	0.0686
149	Liver_EC_S2_1 = phys_BW*phys_Normalized_weight_liver_blood*Specific_volume/5	0.0686
150	Liver_EC_S3_1 = phys_BW*phys_Normalized_weight_liver_blood*Specific_volume/5	0.0686
151	Liver_EC_S4_1 = phys_BW*phys_Normalized_weight_liver_blood*Specific_volume/5	0.0686
152	Liver_EC_S5_1 = phys_BW*phys_Normalized_weight_liver_blood*Specific_volume/5	0.0686
153	Liver_IC_S5_1 = phys_BW*phys_Normalized_weight_liver_tissue*Specific_volume/5	0.252
154	Liver_IC_S4_1 = phys_BW*phys_Normalized_weight_liver_tissue*Specific_volume/5	0.252
155	Liver_IC_S3_1 = phys_BW*phys_Normalized_weight_liver_tissue*Specific_volume/5	0.252
156	Liver_IC_S2_1 = phys_BW*phys_Normalized_weight_liver_tissue*Specific_volume/5	0.252
157	Liver_IC_S1_1 = phys_BW*phys_Normalized_weight_liver_tissue*Specific_volume/5	0.252
158	Q_artery_gut_1 = phys_BW*phys_Normalized_Q_gut	71.4
159	Q_artery_liver_1 = Q_li-Q_artery_gut-Q_artery_spleen	4.2
160	Q_artery_spleen_1 = phys_BW*phys_Normalized_Q_spleen	8.4
161	Q_gut_liver_1 = phys_BW*phys_Normalized_Q_gut	71.4
162	Q_spleen_liver_1 = phys_BW*phys_Normalized_Q_spleen	8.4
163	Qadipose_1 = phys_BW*phys_Normalized_Q_adipose	16.8
164	Qbone_1 = phys_BW*phys_Normalized_Q_bone	16.8
165	Qbrain_1 = phys_BW*phys_Normalized_Q_brain	42
166	Qheart_1 = phys_BW*phys_Normalized_Q_heart	12.6
167	Qkidney_1 = phys_BW*phys_Normalized_Q_kidney	63
168	Q_li_1 = phys_BW*phys_Normalized_Q_liver	84
169	Qlung_1 = phys_BW*phys_Normalized_Q_lung	336
170	Qmuscle_1 = phys_BW*phys_Normalized_Q_muscle	58.8
171	Qrest_1 = phys_BW*phys_Normalized_Q_remainder	4.2
172	Qskin_1 = phys_BW*phys_Normalized_Q_skin	16.8
173	drug_fB_1 = drug_fuplasma_1/drug_BRP_1	0.058769
174	k_venous_urine_CLR_1 = drug_CLrenal_1*switch_SFrenal_1	0

	Initial Assignments	Initial Value
175	k_artery_testes_1 = Qtestes_1	0
176	k_testes_venous_1 = Qtestes_1/Kp_testes*drug_BRP_1	0
177	Qtestes_1 = phys_BW*phys_Normalized_Q_testes	0
178	k_Liver_IC_S2_Liver_EC_S2_efflux_1 = switch_SFeff_1*drug_CLeffluxHep_1*phys_HPGL*(phys_BW*phys_Normalized_weight_liver_tissue*Specific_volume)/Specific_volume/5*drug_fuLiver_1	0
179	k_Liver_IC_S3_Liver_EC_S3_efflux_1 = switch_SFeff_1*drug_CLeffluxHep_1*phys_HPGL*(phys_BW*phys_Normalized_weight_liver_tissue*Specific_volume)/Specific_volume/5*drug_fuLiver_1	0
180	k_Liver_IC_S4_Liver_EC_S4_efflux_1 = switch_SFeff_1*drug_CLeffluxHep_1*phys_HPGL*(phys_BW*phys_Normalized_weight_liver_tissue*Specific_volume)/Specific_volume/5*drug_fuLiver_1	0
181	k_Liver_IC_S5_Liver_EC_S5_efflux_1 = switch_SFeff_1*drug_CLeffluxHep_1*phys_HPGL*(phys_BW*phys_Normalized_weight_liver_tissue*Specific_volume)/Specific_volume/5*drug_fuLiver_1	0
182	k_Liver_IC_S1_Liver_EC_S1_efflux_1 = switch_SFeff_1*drug_CLeffluxHep_1*phys_HPGL*(phys_BW*phys_Normalized_weight_liver_tissue*Specific_volume)/Specific_volume/5*drug_fuLiver_1	0
183	Testes_1 = phys_BW*phys_Normalized_weight_testes*Specific_volume	0.07
184	Kp_adipose_1 = drug_Kp_adipose_raw_1*switch_SFKp_1	0.23327
185	Kp_bone_1 = drug_Kp_bone_raw_1*switch_SFKp_1	0.31102
186	Kp_brain_1 = drug_Kp_brain_raw_1*switch_SFKp_1	0.16588
187	Kp_gut_1 = drug_Kp_gut_raw_1*switch_SFKp_1	0.24363
188	Kp_heart_1 = drug_Kp_heart_raw_1*switch_SFKp_1	0.10367
189	Kp_kidney_1 = drug_Kp_kidney_raw_1*switch_SFKp_1	0.13478
190	Kp_lung_1 = drug_Kp_lung_raw_1*switch_SFKp_1	0.20735
191	Kp_muscle_1 = drug_Kp_muscle_raw_1*switch_SFKp_1	0.025919
192	Kp_skin_1 = drug_Kp_skin_raw_1*switch_SFKp_1	0.1918
193	Kp_spleen_1 = drug_Kp_spleen_raw_1*switch_SFKp_1	0.18143
194	Kp_testes_1 = drug_Kp_testes_raw_1*switch_SFKp_1	0.51837
195	k_venous_urine_GFR_1 = drug_GFR_1*drug_fuplasma_1*switch_SFrenal_1	0
196	drug_GFR_1 = drug_FR_1*Qkidney_1	0
197	Kp_rest_1 = switch_slow_dist_Kp_1*drug_Kp_rest_raw_1+(1-switch_slow_dist_Kp_1)*drug_Kp_rest_raw_1*switch_SFKp_1	0.0065
198	STOMACH = StomachLumenTotal/BW_average*phys_BW	0.05
199	QMUC = (Q_gut_liver/numIntestinalCompartments)*liter_to_milliliter/minute_per_hour	170
200	V_LUM_TOT = LumenTotal/BW_average*phys_BW	126.95

	Initial Assignments	Initial Value
201	V_ONECOMP = V_LUM_TOT/numIntestinalCompartments	18.1357
202	V_MEM = (VGut/numIntestinalCompartments)*liter_to_milliliter	73.857
203	LOGSR = 0.75*LOGP+2.27	4.8695
204	SOLINT = SOLFASSIF/HHINT	11.4099
205	SOLIF_STOMACH = SOLINT*HHSTOMACH	971137867.2533
206	SOLIF_DUO = SOLINT*HHDUO	12237.3111
207	SOLIF_JEJ1 = SOLINT*HHJEJ1	9722.7884
208	SOLIF_JEJ2 = SOLINT*HHJEJ2	7725.4321
209	SOLIF_ILL1 = SOLINT*HHILL1	4878.6288
210	SOLIF_ILL2 = SOLINT*HHILL2	3082.4174
211	SOLIF_ILL3 = SOLINT*HHILL3	624.1564
212	SOLIF_ILL4 = SOLINT*HHILL4	1550.5597
213	HPeff_est = diff_api	0.00011667
214	DIFF = second_per_minute*HPeff_est*ESA/numIntestinalCompartments	120
215	NI_DUO = 1/HHDUO	0.00093238
216	NI_JEJ1 = 1/HHJEJ1	0.0011735
217	NI_JEJ2 = 1/HHJEJ2	0.0014769
218	NI_ILL1 = 1/HHILL1	0.0023387
219	NI_ILL2 = 1/HHILL2	0.0037016
220	NI_ILL3 = 1/HHILL3	0.01828
221	NI_ILL4 = 1/HHILL4	0.0073586
222	KD = 3*DIFFCOEFF/(PDENSITY*PSIZE*DLT)	0.0001306
223	VDUO = V_LUM_TOT*lumenvolumeRatio_DUO	0.03435
224	VJEJ1 = V_LUM_TOT*lumenvolumeRatio_JEJ1	0.0211
225	VJEJ2 = V_LUM_TOT*lumenvolumeRatio_JEJ2	0.0211
226	VILL1 = V_LUM_TOT*lumenvolumeRatio_ILL1	0.0126
227	VILL2 = V_LUM_TOT*lumenvolumeRatio_ILL2	0.0126
228	VILL3 = V_LUM_TOT*lumenvolumeRatio_ILL3	0.0126
229	VILL4 = V_LUM_TOT*lumenvolumeRatio_ILL4	0.0126
230	MDUO = VGut*volumeRatio_DUO*Gut_IC_fraction	0.037454
231	MJEJ1 = VGut*volumeRatio_JEJ1*Gut_IC_fraction	0.073785
232	MJEJ2 = VGut*volumeRatio_JEJ2*Gut_IC_fraction	0.051687
233	MILL1 = VGut*volumeRatio_ILL1*Gut_IC_fraction	0.0412
234	MILL2 = VGut*volumeRatio_ILL2*Gut_IC_fraction	0.0412
235	MILL3 = VGut*volumeRatio_ILL3*Gut_IC_fraction	0.04045
236	MILL4 = VGut*volumeRatio_ILL4*Gut_IC_fraction	0.038952
237	VGut = phys_BW*phys_Normalized_weight_enterocyte*Specific_volume	0.517

	Initial Assignments	Initial Value
238	VillousDUO = VGut*volumeRatio_DUO*Gut_EC_fraction*drug_fQ	0.0057016
239	VillousJEJ1 = VGut*volumeRatio_JEJ1*Gut_EC_fraction*drug_fQ	0.011232
240	VillousJEJ2 = VGut*volumeRatio_JEJ2*Gut_EC_fraction*drug_fQ	0.0078682
241	VillousILL1 = VGut*volumeRatio_ILL1*Gut_EC_fraction*drug_fQ	0.0062718
242	VillousILL2 = VGut*volumeRatio_ILL2*Gut_EC_fraction*drug_fQ	0.0062718
243	VillousILL3 = VGut*volumeRatio_ILL3*Gut_EC_fraction*drug_fQ	0.0061578
244	VillousILL4 = VGut*volumeRatio_ILL4*Gut_EC_fraction*drug_fQ	0.0059297
245	Qmuc_DUO = drug_fQ*Q_artery_gut*flowRatio_DUO	26.9235
246	Qmuc_JEJ1 = drug_fQ*Q_artery_gut*flowRatio_JEJ1	74.0397
247	Qmuc_JEJ2 = drug_fQ*Q_artery_gut*flowRatio_JEJ2	74.0397
248	Qmuc_ILL1 = drug_fQ*Q_artery_gut*flowRatio_ILL1	32.7365
249	Qmuc_ILL2 = drug_fQ*Q_artery_gut*flowRatio_ILL2	32.7365
250	Qmuc_ILL3 = drug_fQ*Q_artery_gut*flowRatio_ILL3	32.7365
251	Qmuc_ILL4 = drug_fQ*Q_artery_gut*flowRatio_ILL4	32.7365
252	Liver = phys_BW*(phys_Normalized_weight_liver_blood + phys_Normalized_weight_liver_tissue)*Specific_volume	1.603
253	Kp_liver = drug_Kp_liver_raw*switch_SFKp	8.6052
254	k_liver_bile = drug_PSbileg*switch_SFbile*phys_HPGL*(phys_BW*phys_Normalized_weight_liver_tissue*Specific_volume)/Specific_volume*drug_fuLiver	1.3134
255	k_liver_metabolites = (switch_SFmet*((drug_HLM_CLint/drug_fumic*phys_MPGL)+(drug_CLmetg/drug_funic*phys_HPGL))*(phys_BW*phys_Normalized_weight_liver_tissue*Specific_volume)/Specific_volume)*drug_fuLiver	0
256	Q_villi = phys_BW*phys_Normalized_Q_villi	336
257	DIFF_duo = HPeff_est*ESA*surfaceRatio_DUO	96.6
258	DIFF_jej1 = HPeff_est*ESA*surfaceRatio_JEJ1	190.68
259	DIFF_jej2 = HPeff_est*ESA*surfaceRatio_JEJ2	133.56
260	DIFF_ill1 = HPeff_est*ESA*surfaceRatio_ILL1	106.68
261	DIFF_ill2 = HPeff_est*ESA*surfaceRatio_ILL2	106.68
262	DIFF_ill3 = HPeff_est*ESA*surfaceRatio_ILL3	105
263	DIFF_ill4 = HPeff_est*ESA*surfaceRatio_ILL4	100.8
264	DIFF_BASO_duo = HPeff_est_baso*ESA_baso*basoSurfaceRatio_DUO	2.468
265	DIFF_BASO_jej1 = HPeff_est_baso*ESA_baso*basoSurfaceRatio_JEJ1	9.5062
266	DIFF_BASO_jej2 = HPeff_est_baso*ESA_baso*basoSurfaceRatio_JEJ2	9.5062
267	DIFF_BASO_ill1 = HPeff_est_baso*ESA_baso*basoSurfaceRatio_ILL1	6.3625

	Initial Assignments	Initial Value
268	DIFF_BASO_ill2 = HPeff_est_baso*ESA_baso*basoSurfaceRatio_ILL2	6.3625
269	DIFF_BASO_ill3 = HPeff_est_baso*ESA_baso*basoSurfaceRatio_ILL3	6.3625
270	DIFF_BASO_ill4 = HPeff_est_baso*ESA_baso*basoSurfaceRatio_ILL4	6.3625
271	k_artery_serosa = (1-drug_fQ)*Q_artery_gut	53.0431
272	k_serosa_liver = (1-drug_fQ)*Q_gut_liver/Kp_serosa*drug_BRP	9.2612
273	Kp_serosa = drug_Kp_serosa_raw*switch_SFKp	5.3838
274	HHINT = 1+(10^(pKA-REFPHSOL))	108.1519
275	HHSTOMACH = 1+(10^(pKA-pHStomach))	85113804.8202
276	HHDUO = 1+(10^(pKA-pHDuo))	1072.5193
277	HHJEJ1 = 1+(10^(pKA-pHJej1))	852.138
278	HHJEJ2 = 1+(10^(pKA-pHJej2))	677.083
279	HHILL1 = 1+(10^(pKA-pHIll1))	427.5795
280	HHILL2 = 1+(10^(pKA-pHIll2))	270.1535
281	HHILL3 = 1+(10^(pKA-pHIll3))	54.7032
282	HHILL4 = 1+(10^(pKA-pHIll4))	135.8963
283	CLINT_influx_DUO = CL_inf_api*ESA*surfaceRatio_DUO	0
284	CLINT_influx_JEJ1 = CL_inf_api * ESA * surfaceRatio_JEJ1	0
285	CLINT_influx_JEJ2 = CL_inf_api * ESA * surfaceRatio_JEJ2	0
286	CLINT_influx_ILL1 = CL_inf_api * ESA * surfaceRatio_ILL1	0
287	CLINT_influx_ILL2 = CL_inf_api * ESA * surfaceRatio_ILL2	0
288	CLINT_influx_ILL3 = CL_inf_api * ESA * surfaceRatio_ILL3	0
289	CLINT_influx_ILL4 = CL_inf_api * ESA * surfaceRatio_ILL4	0
290	CLINT_efflux_DUO = CL_eff * ESA * surfaceRatio_DUO	18.7873
291	CLINT_efflux_JEJ1 = CL_eff * ESA * surfaceRatio_JEJ1	37.0845
292	CLINT_efflux_JEJ2 = CL_eff * ESA * surfaceRatio_JEJ2	25.9755
293	CLINT_efflux_ILL1 = CL_eff * ESA * surfaceRatio_ILL1	20.7477
294	CLINT_efflux_ILL2 = CL_eff * ESA * surfaceRatio_ILL2	20.7477
295	CLINT_efflux_ILL3 = CL_eff * ESA * surfaceRatio_ILL3	20.421
296	CLINT_efflux_ILL4 = CL_eff * ESA * surfaceRatio_ILL4	19.6042
297	HPeff_est_baso = diff_baso	0.00011667
298	CLINT_influx_baso_DUO = CL_inf_baso*ESA_baso*basoSurfaceRatio_DUO	0
299	CLINT_influx_baso_JEJ1 = CL_inf_baso * ESA_baso * basoSurfaceRatio_JEJ1	0
300	CLINT_influx_baso_JEJ2 = CL_inf_baso * ESA_baso * basoSurfaceRatio_JEJ2	0
301	CLINT_influx_baso_ILL1 = CL_inf_baso * ESA_baso * basoSurfaceRatio_ILL1	0

	Initial Assignments	Initial Value
302	CLINT_influx_baso_ILL2 = CL_inf_baso * ESA_baso * basoSurfaceRatio_ILL2	0
303	CLINT_influx_baso_ILL3 = CL_inf_baso * ESA_baso * basoSurfaceRatio_ILL3	0
304	CLINT_influx_baso_ILL4 = CL_inf_baso * ESA_baso * basoSurfaceRatio_ILL4	0
305	Serosa = VGut*Gut_EC_fraction*(1-drug_fQ)	0.14284
306	diff_api = diff_baso	7
307	switch_SFefflux_Lower = switch_SFefflux*SFefflux	4.18
308	CLINT_efflux_baso_JEJ1 = CLINT_efflux_baso_DUO	0
309	CLINT_efflux_baso_JEJ2 = CLINT_efflux_baso_DUO	0
310	switch_SFefflux_Lower = switch_SFefflux	4.18
311	switch_SFinflux_Lower = switch_SFinflux/SFinflux	1
312	CLINT_efflux_baso_ILL1 = CLINT_efflux_baso_DUO	0
313	CLINT_efflux_baso_ILL2 = CLINT_efflux_baso_DUO	0
314	CLINT_efflux_baso_ILL3 = CLINT_efflux_baso_DUO	0
315	CLINT_efflux_baso_ILL4 = CLINT_efflux_baso_DUO	0
316	CLINT_efflux_baso_DUO = CL_eff_baso*ESA_baso*basoSurfaceRatio_DUO	0
317	CLINT_efflux_baso_ILL1 = CL_eff_baso*ESA_baso*basoSurfaceRatio_ILL1	0
318	CLINT_efflux_baso_ILL2 = CL_eff_baso*ESA_baso*basoSurfaceRatio_ILL2	0
319	CLINT_efflux_baso_ILL3 = CL_eff_baso*ESA_baso*basoSurfaceRatio_ILL3	0
320	CLINT_efflux_baso_ILL4 = CL_eff_baso*ESA_baso*basoSurfaceRatio_ILL4	0
321	CLINT_efflux_baso_JEJ1 = CL_eff_baso*ESA_baso*basoSurfaceRatio_JEJ1	0
322	CLINT_efflux_baso_JEJ2 = CL_eff_baso*ESA_baso*basoSurfaceRatio_JEJ2	0
323	HHMembrane = 1 + (10^(pKA - pHMembrane))	108.1519
324	HHVillous = 1 + (10^(pKA - pHVillous))	170.8244
325	NI_Membrane = 1/HHMembrane	0.0092463
326	NI_Villous = 1/HHVillous	0.005854

Repeated Assignments

	Repeated Assignments	Initial Value
1	k_Liver_IC_S5_Metabolites = (switch_Vmax_met*phys_BW/(drug_Km_met+drug_fuLiver *Liver_IC_S5.Liver_IC_S5_drug/drug_molar_mass)+switch_SFmet*((drug_HLM_CLint/drug_fumic*phys_MPGL)+(drug_CLmetg/drug_funic*phys_HPGL))*(phys_BW*phys_Normalized_weight_liver_tissue*Specific_volume)/Specific_volume)/5*drug_fuLiver	0
2	k_Liver_IC_S4_Metabolites = (switch_Vmax_met*phys_BW/(drug_Km_met+drug_fuLiver *Liver_IC_S4.Liver_IC_S4_drug/drug_molar_mass)+switc	0

	Repeated Assignments	Initial Value
	h_SFmet*((drug_HLM_CLint/drug_fumic*phys_MPGL)+(drug_CLmetg/drug_funic*phys_HPGL))*(phys_BW*phys_Normalized_weight_liver_tissue*Specific_volume)/Specific_volume)/5*drug_fuLiver	
3	k_Liver_IC_S3_Metabolites = (switch_Vmax_met*phys_BW/(drug_Km_met+drug_fuLiver*Liver_IC_S3.Liver_IC_S3_drug/drug_molar_mass)+switch_SFmet*((drug_HLM_CLint/drug_fumic*phys_MPGL)+(drug_CLmetg/drug_funic*phys_HPGL))*(phys_BW*phys_Normalized_weight_liver_tissue*Specific_volume)/Specific_volume)/5*drug_fuLiver	0
4	k_Liver_IC_S2_Metabolites = (switch_Vmax_met*phys_BW/(drug_Km_met+drug_fuLiver*Liver_IC_S2.Liver_IC_S2_drug/drug_molar_mass)+switch_SFmet*((drug_HLM_CLint/drug_fumic*phys_MPGL)+(drug_CLmetg/drug_funic*phys_HPGL))*(phys_BW*phys_Normalized_weight_liver_tissue*Specific_volume)/Specific_volume)/5*drug_fuLiver	0
5	k_Liver_IC_S1_Metabolites = (switch_Vmax_met*phys_BW/(drug_Km_met+drug_fuLiver*Liver_IC_S1.Liver_IC_S1_drug/drug_molar_mass)+switch_SFmet*((drug_HLM_CLint/drug_fumic*phys_MPGL)+(drug_CLmetg/drug_funic*phys_HPGL))*(phys_BW*phys_Normalized_weight_liver_tissue*Specific_volume)/Specific_volume)/5*drug_fuLiver	0
6	k_Liver_EC_S5_Liver_IC_S5 = (drug_PSdifg*switch_SFdiff*phys_HPGL*(phys_BW*phys_Normalized_weight_liver_tissue*Specific_volume)/Specific_volume+switch_Vmax_uptake*phys_BW/(drug_Km_uptake+drug_fB*Liver_EC_S5.Liver_EC_S5_drug/drug_molar_mass)+drug_PSinfg*switch_SFinf*phys_HPGL*(phys_BW*phys_Normalized_weight_liver_tissue*Specific_volume)/Specific_volume)/5*drug_fB	1369.0662
7	k_Liver_EC_S4_Liver_IC_S4 = (drug_PSdifg*switch_SFdiff*phys_HPGL*(phys_BW*phys_Normalized_weight_liver_tissue*Specific_volume)/Specific_volume+switch_Vmax_uptake*phys_BW/(drug_Km_uptake+drug_fB*Liver_EC_S4.Liver_EC_S4_drug/drug_molar_mass)+drug_PSinfg*switch_SFinf*phys_HPGL*(phys_BW*phys_Normalized_weight_liver_tissue*Specific_volume)/Specific_volume)/5*drug_fB	1369.0662
8	k_Liver_EC_S3_Liver_IC_S3 = (drug_PSdifg*switch_SFdiff*phys_HPGL*(phys_BW*phys_Normalized_weight_liver_tissue*Specific_volume)/Specific_volume+switch_Vmax_uptake*phys_BW/(drug_Km_uptake+drug_fB*Liver_EC_S3.Liver_EC_S3_drug/drug_molar_mass)+drug_PSinfg*switch_SFinf*phys_HPGL*(phys_BW*phys_Normalized_weight_liver_tissue*Specific_volume)/Specific_volume)/5*drug_fB	1369.0662

	Repeated Assignments	Initial Value
9	k_Liver_EC_S2_Liver_IC_S2 = (drug_PSdifg*switch_SFdiff*phys_HPGL*(phys_BW*phys_Normalized_weight_liver_tissue*Specific_volume)/Specific_volume+switch_Vmax_uptake*phys_BW/(drug_Km_uptake+drug_fB*Liver_EC_S2.Liver_EC_S2_drug/drug_molar_mass)+drug_PSinfg*switch_SFinf*phys_HPGL*(phys_BW*phys_Normalized_weight_liver_tissue*Specific_volume)/Specific_volume)/5*drug_fB	1369.0662
10	k_Liver_EC_S1_Liver_IC_S1 = (drug_PSdifg*switch_SFdiff*phys_HPGL*(phys_BW*phys_Normalized_weight_liver_tissue*Specific_volume)/Specific_volume+switch_Vmax_uptake*phys_BW/(drug_Km_uptake+drug_fB*Liver_EC_S1.Liver_EC_S1_drug/drug_molar_mass)+drug_PSinfg*switch_SFinf*phys_HPGL*(phys_BW*phys_Normalized_weight_liver_tissue*Specific_volume)/Specific_volume)/5*drug_fB	1369.0662
11	Blood_total.Blood_total_drug = (Artery.Artery_drug*Artery+Venous.Venous_drug*Venous)/(Artery+Venous)	0
12	Plasma_total.Plasma_total_drug = Blood_total.Blood_total_drug/drug_BRP	0
13	Portal.Portal_drug = (Q_gut_liver/numIntestinalCompartments*(Villous_DUO/VillousDUO+Villous_JEJ1/VillousJEJ1+Villous_JEJ2/VillousJEJ2+Villous_ILL1/VillousILL1+Villous_ILL2/VillousILL2+Villous_ILL3/VillousILL3+Villous_ILL4/VillousILL4)+Q_spleen_liver*(Spleen.Spleen_drug/Kp_spleen*drug_BRP))/(Q_gut_liver+Q_spleen_liver)	0
14	Portal_1.Portal_plasma_drug = Portal.Portal_drug/drug_BRP	0
15	Mass_Balance.Amount_body = Venous.Venous_drug*Venous+Artery.Artery_drug*Artery+Liver_IC_S1.Liver_IC_S1_drug*Liver_IC_S1+Liver_IC_S2.Liver_IC_S2_drug*Liver_IC_S2+Liver_IC_S3.Liver_IC_S3_drug*Liver_IC_S3+Liver_IC_S4.Liver_IC_S4_drug*Liver_IC_S4+Liver_IC_S5.Liver_IC_S5_drug*Liver_IC_S5+Liver_EC_S1.Liver_EC_S1_drug*Liver_EC_S1+Liver_EC_S2.Liver_EC_S2_drug*Liver_EC_S2+Liver_EC_S3.Liver_EC_S3_drug*Liver_EC_S3+Liver_EC_S4.Liver_EC_S4_drug*Liver_EC_S4+Liver_EC_S5.Liver_EC_S5_drug*Liver_EC_S5+Lung.Lung_drug*Lung+Adipose.Adipose_drug*Adipose+Heart.Heart_drug*Heart+Muscle.Muscle_drug*Muscle+Skin.Skin_drug*Skin+Kidney.Kidney_drug*Kidney+Bone.Bone_drug*Bone+Testes.Testes_drug*Testes+Rest.Rest_drug*Rest+Gut.Gut_drug*Gut+MDUO.MEM_DUO*milligram_per_microgram+MJEJ1.MEM_JEJ1*milligram_per_microgram+MJEJ2.MEM_JEJ2*milligram_per_microgram+MILL1.MEM_ILL1*milligram_per_microgram+MILL2.MEM_ILL2*milligram_per_microgram+MILL3.MEM_ILL3*milligram_per_microgram+MILL4.MEM_ILL4*milligram_per_microgram+Spleen.Spleen_drug*Spleen+Brain.Brain_drug*Brain+Villous_DUO+Villous_JEJ1+Villous_JEJ2+Villous_ILL1+Villous_ILL2+Villous_ILL3+Villous_ILL4	0
16	Mass_Balance.Amount_total = Venous.Venous_drug*Venous+Artery.Artery_drug*Artery+Liver_IC_S1.Liver_IC_S1_drug*Liver_IC_S1+Liver_IC_S2.Liver_IC_S2_drug*Liver_IC_S2+Liver_IC_S3.Liver_IC_S3_	0

	Repeated Assignments	Initial Value
	drug*Liver_IC_S3+Liver_IC_S4.Liver_IC_S4_drug*Liver_IC_S4+Liver_IC_S5.Liver_IC_S5_drug*Liver_IC_S5+Liver_EC_S1.Liver_EC_S1_drug*Liver_EC_S1+Liver_EC_S2.Liver_EC_S2_drug*Liver_EC_S2+Liver_EC_S3.Liver_EC_S3_drug*Liver_EC_S3+Liver_EC_S4.Liver_EC_S4_drug*Liver_EC_S4+Liver_EC_S5.Liver_EC_S5_drug*Liver_EC_S5+Lung.Lung_drug*Lung+Adipose.Adipose_drug*Adipose+Heart.Heart_drug*Heart+Muscle.Muscle_drug*Muscle+Skin.Skin_drug*Skin+Kidney.Kidney_drug*Kidney+Bone.Bone_drug*Bone+Testes.Testes_drug*Testes+Rest.Rest_drug*Rest+ (MDUO.MEM_DUO+MJEJ1.MEM_JEJ1+MJEJ2.MEM_JEJ2+MILL1.MEM_ILL1+MILL2.MEM_ILL2+MILL3.MEM_ILL3+MILL4.MEM_ILL4)*milligram_per_microgram+Spleen.Spleen_drug*Spleen+Brain.Brain_drug*Brain+Main_compartment.Bile_drug+Urine.Urine_drug+Gut_Lumen.Gut_Lumen_drug+ (STOMACH.X_STOMACH_DISS+VDUO.X_DUO_DISS+VJEJ1.X_JEJ1_DISS+VJEJ2.X_JEJ2_DISS+VILL1.X_ILL1_DISS+VILL2.X_ILL2_DISS+VILL3.X_ILL3_DISS+VILL4.X_ILL4_DISS+Colon.X_CECUM_DISS)*milligram_per_microgram+ (STOMACH.X_STOMACH_SOLID+VDUO.X_DUO_SOLID+VJEJ1.X_JEJ1_SOLID+VJEJ2.X_JEJ2_SOLID+VILL1.X_ILL1_SOLID+VILL2.X_ILL2_SOLID+VILL3.X_ILL3_SOLID+VILL4.X_ILL4_SOLID+Colon.X_CECUM_SOLID)*milligram_per_microgram+Villous_DUO+Villous_JEJ1+Villous_JEJ2+Villous_ILL1+Villous_ILL2+Villous_ILL3+Villous_ILL4	
17	Plasma_total.Plasma_free_uM = Plasma_total.Plasma_total_drug*drug_fuplasma/drug_molar_mass	0
18	Liver_total.Liver_blood_total = (Liver_EC_S1.Liver_EC_S1_drug+Liver_EC_S2.Liver_EC_S2_drug+Liver_EC_S3.Liver_EC_S3_drug+Liver_EC_S4.Liver_EC_S4_drug+Liver_EC_S5.Liver_EC_S5_drug)/5	0
19	Liver_total.Liver_tissue_total = (Liver_IC_S1.Liver_IC_S1_drug+Liver_IC_S2.Liver_IC_S2_drug+Liver_IC_S3.Liver_IC_S3_drug+Liver_IC_S4.Liver_IC_S4_drug+Liver_IC_S5.Liver_IC_S5_drug)/5	0
20	Liver_total.Liver_blood_free = Liver_total.Liver_blood_total*drug_fuplasma/drug_BRP	0
21	Liver_total.Liver_tissue_free = Liver_total.Liver_tissue_total*drug_fuLiver	0
22	convert_to_nmole_per_kg.Adipose_nmole = Adipose.Adipose_drug*Adipose/drug_molar_mass*nanomole_per_mole*kilogram/phys_BW	0
23	convert_to_nmole_per_kg.Artery_nmole = Artery.Artery_drug*Artery/drug_molar_mass*nanomole_per_mole*kilogram/phys_BW	0
24	convert_to_nmole_per_kg.Bone_nmole = Bone.Bone_drug*Bone/drug_molar_mass*nanomole_per_mole*kilogram/phys_BW	0
25	convert_to_nmole_per_kg.Liver_EC1_nmole = Liver_EC_S1.Liver_EC_S1_drug*Liver_EC_S1/drug_molar_mass*nanomole_per_mole*kilogram/phys_BW	0
26	convert_to_nmole_per_kg.Liver_IC1_nmole = Liver_IC_S1.Liver_IC_S1_drug*Liver_IC_S1/drug_molar_mass*nanomole_per_mole*kilogram/phys_BW	0

	Repeated Assignments	Initial Value
27	convert_to_nmole_per_kg.Muscle_nmole = Muscle.Muscle_drug*Muscle/drug_molar_mass*nanomole_per_mole*kilogram/phys_BW	0
28	convert_to_nmole_per_kg.Urine_nmole = Urine.Urine_drug/drug_molar_mass*nanomole_per_mole*kilogram/phys_BW	0
29	convert_to_nmole_per_kg.Venous_nmole = Venous.Venous_drug*Venous/drug_molar_mass*nanomole_per_mole*kilogram/phys_BW	0
30	convert_to_nmole_per_kg.Bile_nmole = Main_compartment.Bile_drug/drug_molar_mass*nanomole_per_mole*kilogram/phys_BW	0
31	Plasma_total.Plasma_total_uM = Plasma_total.Plasma_total_drug/drug_molar_mass	0
32	Liver_total.Liver_tissue_total_uM = Liver_total.Liver_tissue_total/drug_molar_mass	0
33	convert_to_nmole_per_kg.Kidney_nmole = Kidney.Kidney_drug*Kidney/drug_molar_mass*nanomole_per_mole*kilogram/phys_BW	0
34	convert_to_nmole_per_kg.Lung_nmole = Lung.Lung_drug*Lung/drug_molar_mass*nanomole_per_mole*kilogram/phys_BW	0
35	convert_to_nmole_per_kg.Metabolites_nmole = Metabolites.Metabolites_drug/drug_molar_mass*nanomole_per_mole*kilogram/phys_BW	0
36	convert_to_nmole_per_kg.Liver_IC2_nmole = Liver_IC_S2.Liver_IC_S2_drug*Liver_IC_S2/drug_molar_mass*nanomole_per_mole*kilogram/phys_BW	0
37	convert_to_nmole_per_kg.Liver_EC2_nmole = Liver_EC_S2.Liver_EC_S2_drug*Liver_EC_S2/drug_molar_mass*nanomole_per_mole*kilogram/phys_BW	0
38	convert_to_nmole_per_kg.Liver_EC3_nmole = Liver_EC_S3.Liver_EC_S3_drug*Liver_EC_S3/drug_molar_mass*nanomole_per_mole*kilogram/phys_BW	0
39	convert_to_nmole_per_kg.Liver_IC3_nmole = Liver_IC_S3.Liver_IC_S3_drug*Liver_IC_S3/drug_molar_mass*nanomole_per_mole*kilogram/phys_BW	0
40	convert_to_nmole_per_kg.Liver_IC4_nmole = Liver_IC_S4.Liver_IC_S4_drug*Liver_IC_S4/drug_molar_mass*nanomole_per_mole*kilogram/phys_BW	0
41	convert_to_nmole_per_kg.Liver_EC4_nmole = Liver_EC_S4.Liver_EC_S4_drug*Liver_EC_S4/drug_molar_mass*nanomole_per_mole*kilogram/phys_BW	0
42	convert_to_nmole_per_kg.Liver_EC5_nmole = Liver_EC_S5.Liver_EC_S5_drug*Liver_EC_S5/drug_molar_mass*nanomole_per_mole*kilogram/phys_BW	0
43	convert_to_nmole_per_kg.Liver_IC5_nmole = Liver_IC_S5.Liver_IC_S5_drug*Liver_IC_S5/drug_molar_mass*nanomole_per_mole*kilogram/phys_BW	0
44	convert_to_nmole_per_kg.Gut_nmole = Gut.Gut_drug*Gut/drug_molar_mass*nanomole_per_mole*kilogram/phys_BW	0
45	convert_to_nmole_per_kg.Spleen_nmole = Spleen.Spleen_drug*Spleen/drug_molar_mass*nanomole_	0

	Repeated Assignments	Initial Value
	per_mole*kilogram/phys_BW	
46	convert_to_nmole_per_kg.Skin_nmole = Skin.Skin_drug*Skin/drug_molar_mass*nanomole_per_mole*kilogram/phys_BW	0
47	convert_to_nmole_per_kg.Brain_nmole = Brain.Brain_drug*Brain/drug_molar_mass*nanomole_per_mole*kilogram/phys_BW	0
48	convert_to_nmole_per_kg.Rest_nmole = Rest.Rest_drug*Rest/drug_molar_mass*nanomole_per_mole*kilogram/phys_BW	0
49	convert_to_nmole_per_kg.Heart_nmole = Heart.Heart_drug*Heart/drug_molar_mass*nanomole_per_mole*kilogram/phys_BW	0
50	k_Liver_IC_S5_Metabolites_1 = met_inhib_S5*(switch_Vmax_met_1*phys_BW/(drug_Km_met_1+drug_fuLiver_1*Liver_IC_S5_1.Liver_IC_S5_drug_1/drug_molar_mass_1)+switch_SFmet_1*((drug_HLM_CLint_1/drug_fumic_1*phys_MPGL)+(drug_CLmetg_1/drug_funic_1*phys_HPGL))*(phys_BW*phys_Normalized_weight_liver_tissue*Specific_volume)/Specific_volume)/5*drug_fuLiver_1	0.21013
51	k_Liver_IC_S4_Metabolites_1 = met_inhib_S4*(switch_Vmax_met_1*phys_BW/(drug_Km_met_1+drug_fuLiver_1*Liver_IC_S4_1.Liver_IC_S4_drug_1/drug_molar_mass_1)+switch_SFmet_1*((drug_HLM_CLint_1/drug_fumic_1*phys_MPGL)+(drug_CLmetg_1/drug_funic_1*phys_HPGL))*(phys_BW*phys_Normalized_weight_liver_tissue*Specific_volume)/Specific_volume)/5*drug_fuLiver_1	0.21013
52	k_Liver_IC_S3_Metabolites_1 = met_inhib_S3*(switch_Vmax_met_1*phys_BW/(drug_Km_met_1+drug_fuLiver_1*Liver_IC_S3_1.Liver_IC_S3_drug_1/drug_molar_mass_1)+switch_SFmet_1*((drug_HLM_CLint_1/drug_fumic_1*phys_MPGL)+(drug_CLmetg_1/drug_funic_1*phys_HPGL))*(phys_BW*phys_Normalized_weight_liver_tissue*Specific_volume)/Specific_volume)/5*drug_fuLiver_1	0.21013
53	k_Liver_IC_S2_Metabolites_1 = met_inhib_S2*(switch_Vmax_met_1*phys_BW/(drug_Km_met_1+drug_fuLiver_1*Liver_IC_S2_1.Liver_IC_S2_drug_1/drug_molar_mass_1)+switch_SFmet_1*((drug_HLM_CLint_1/drug_fumic_1*phys_MPGL)+(drug_CLmetg_1/drug_funic_1*phys_HPGL))*(phys_BW*phys_Normalized_weight_liver_tissue*Specific_volume)/Specific_volume)/5*drug_fuLiver_1	0.21013
54	k_Liver_IC_S1_Metabolites_1 = met_inhib_S1*(switch_Vmax_met_1*phys_BW/(drug_Km_met_1+drug_fuLiver_1*Liver_IC_S1_1.Liver_IC_S1_drug_1/drug_molar_mass_1)+switch_SFmet_1*((drug_HLM_CLint_1/drug_fumic_1*phys_MPGL)+(drug_CLmetg_1/drug_funic_1*phys_HPGL))*(phys_BW*phys_Normalized_weight_liver_tissue*Specific_volume)/Specific_volume)/5*drug_fuLiver_1	0.21013
55	k_Liver_IC_S5_Bile_1 = biliary_inhib_S5*drug_PSbileg_1*switch_SFbile_1*phys_HPGL*(phys_BW*phys_Normalized_weight_liver_tissue*Specific_volume)/Specific_volume/5*drug_fuLiver_1	0.019658

	Repeated Assignments	Initial Value
56	k_Liver_IC_S4_Bile_1 = biliary_inhib_S4*drug_PSbileg_1*switch_SFbile_1*phys_HPGL*(phys_BW*phys_Normalized_weight_liver_tissue*Specific_volume)/Specific_volume/5*drug_fuLiver_1	0.019658
57	k_Liver_IC_S3_Bile_1 = biliary_inhib_S3*drug_PSbileg_1*switch_SFbile_1*phys_HPGL*(phys_BW*phys_Normalized_weight_liver_tissue*Specific_volume)/Specific_volume/5*drug_fuLiver_1	0.019658
58	k_Liver_IC_S2_Bile_1 = biliary_inhib_S2*drug_PSbileg_1*switch_SFbile_1*phys_HPGL*(phys_BW*phys_Normalized_weight_liver_tissue*Specific_volume)/Specific_volume/5*drug_fuLiver_1	0.019658
59	k_Liver_IC_S1_Bile_1 = biliary_inhib_S1*drug_PSbileg_1*switch_SFbile_1*phys_HPGL*(phys_BW*phys_Normalized_weight_liver_tissue*Specific_volume)/Specific_volume/5*drug_fuLiver_1	0.019658
60	k_Liver_EC_S5_Liver_IC_S5_1 = (drug_PSdifg_1*switch_SFdiff_1*phys_HPGL*(phys_BW*phys_Normalized_weight_liver_tissue*Specific_volume)/Specific_volume+uptake_inhib_S5*switch_Vmax_uptake_1*phys_BW/(drug_Km_uptake_1+drug_fB_1*Liver_EC_S5_1.Liver_EC_S5_drug_1/drug_molar_mass_1)+uptake_inhib_S5*drug_PSinfg_1*switch_SFinf_1*phys_HPGL*(phys_BW*phys_Normalized_weight_liver_tissue*Specific_volume)/Specific_volume)/5*drug_fB_1	168.0698
61	k_Liver_EC_S4_Liver_IC_S4_1 = (drug_PSdifg_1*switch_SFdiff_1*phys_HPGL*(phys_BW*phys_Normalized_weight_liver_tissue*Specific_volume)/Specific_volume+uptake_inhib_S4*switch_Vmax_uptake_1*phys_BW/(drug_Km_uptake_1+drug_fB_1*Liver_EC_S4_1.Liver_EC_S4_drug_1/drug_molar_mass_1)+uptake_inhib_S4*drug_PSinfg_1*switch_SFinf_1*phys_HPGL*(phys_BW*phys_Normalized_weight_liver_tissue*Specific_volume)/Specific_volume)/5*drug_fB_1	168.0698
62	k_Liver_EC_S3_Liver_IC_S3_1 = (drug_PSdifg_1*switch_SFdiff_1*phys_HPGL*(phys_BW*phys_Normalized_weight_liver_tissue*Specific_volume)/Specific_volume+uptake_inhib_S3*switch_Vmax_uptake_1*phys_BW/(drug_Km_uptake_1+drug_fB_1*Liver_EC_S3_1.Liver_EC_S3_drug_1/drug_molar_mass_1)+uptake_inhib_S3*drug_PSinfg_1*switch_SFinf_1*phys_HPGL*(phys_BW*phys_Normalized_weight_liver_tissue*Specific_volume)/Specific_volume)/5*drug_fB_1	168.0698
63	k_Liver_EC_S2_Liver_IC_S2_1 = (drug_PSdifg_1*switch_SFdiff_1*phys_HPGL*(phys_BW*phys_Normalized_weight_liver_tissue*Specific_volume)/Specific_volume+uptake_inhib_S2*switch_Vmax_uptake_1*phys_BW/(drug_Km_uptake_1+drug_fB_1*Liver_EC_S2_1.Liver_EC_S2_drug_1/drug_molar_mass_1)+uptake_inhib_S2*drug_PSinfg_1*switch_SFinf_1*phys_HPGL*(phys_BW*phys_Normalized_weight_liver_tissue*Specific_volume)/Specific_volume)/5*drug_fB_1	168.0698

	Repeated Assignments	Initial Value
64	k_Liver_EC_S1_Liver_IC_S1_1 = (drug_PSdifg_1*switch_SFdiff_1*phys_HPGL*(phys_BW*phys_Normalized_weight_liver_tissue*Specific_volume)/Specific_volume+uptake_inhib_S1*switch_Vmax_uptake_1*phys_BW/(drug_Km_uptake_1+drug_fB_1*Liver_EC_S1_1.Liver_EC_S1_drug_1/drug_molar_mass_1)+uptake_inhib_S1*drug_PSinfg_1*switch_SFinf_1*phys_HPGL*(phys_BW*phys_Normalized_weight_liver_tissue*Specific_volume)/Specific_volume)/5*drug_fB_1	168.0698
65	Blood_total_1.Blood_total_drug_1 = (Artery_1.Artery_drug_1*Artery_1+Venous_1.Venous_drug_1*Venous_1)/(Artery_1+Venous_1)	0
66	Plasma_total_1.Plasma_total_drug_1 = Blood_total_1.Blood_total_drug_1/drug_BRP_1	0
67	Portal_1.Portal_drug_1 = (Q_gut_liver_1*(Gut_1.Gut_drug_1/Kp_gut_1*drug_BRP_1)+Q_spleen_liver_1*(Spleen_1.Spleen_drug_1/Kp_spleen_1*drug_BRP_1))/(Q_gut_liver_1+Q_spleen_liver_1)	0
68	Portal_1.Portal_plasma_drug_1 = Portal_1.Portal_drug_1/drug_BRP_1	0
69	Mass_Balance_1.Amount_body_1 = Venous_1.Venous_drug_1*Venous_1+Artery_1.Artery_drug_1*Artery_1+Liver_IC_S1_1.Liver_IC_S1_drug_1*Liver_IC_S1_1+Liver_IC_S2_1.Liver_IC_S2_drug_1*Liver_IC_S2_1+Liver_IC_S3_1.Liver_IC_S3_drug_1*Liver_IC_S3_1+Liver_IC_S4_1.Liver_IC_S4_drug_1*Liver_IC_S4_1+Liver_IC_S5_1.Liver_IC_S5_drug_1*Liver_IC_S5_1+Liver_EC_S1_1.Liver_EC_S1_drug_1*Liver_EC_S1_1+Liver_EC_S2_1.Liver_EC_S2_drug_1*Liver_EC_S2_1+Liver_EC_S3_1.Liver_EC_S3_drug_1*Liver_EC_S3_1+Liver_EC_S4_1.Liver_EC_S4_drug_1*Liver_EC_S4_1+Liver_EC_S5_1.Liver_EC_S5_drug_1*Liver_EC_S5_1+Lung_1.Lung_drug_1*Lung_1+Adipose_1.Adipose_drug_1*Adipose_1+Heart_1.Heart_drug_1*Heart_1+Muscle_1.Muscle_drug_1*Muscle_1+Skin_1.Skin_drug_1*Skin_1+Kidney_1.Kidney_drug_1*Kidney_1+Bone_1.Bone_drug_1*Bone_1+Testes_1.Testes_drug_1*Testes_1+Rest_1.Rest_drug_1*Rest_1+Gut_1.Gut_drug_1*Gut_1+Spleen_1.Spleen_drug_1*Spleen_1+Brain_1.Brain_drug_1*Brain_1	0
70	Mass_Balance_1.Amount_total_1 = Venous_1.Venous_drug_1*Venous_1+Artery_1.Artery_drug_1*Artery_1+Liver_IC_S1_1.Liver_IC_S1_drug_1*Liver_IC_S1_1+Liver_IC_S2_1.Liver_IC_S2_drug_1*Liver_IC_S2_1+Liver_IC_S3_1.Liver_IC_S3_drug_1*Liver_IC_S3_1+Liver_IC_S4_1.Liver_IC_S4_drug_1*Liver_IC_S4_1+Liver_IC_S5_1.Liver_IC_S5_drug_1*Liver_IC_S5_1+Liver_EC_S1_1.Liver_EC_S1_drug_1*Liver_EC_S1_1+Liver_EC_S2_1.Liver_EC_S2_drug_1*Liver_EC_S2_1+Liver_EC_S3_1.Liver_EC_S3_drug_1*Liver_EC_S3_1+Liver_EC_S4_1.Liver_EC_S4_drug_1*Liver_EC_S4_1+Liver_EC_S5_1.Liver_EC_S5_drug_1*Liver_EC_S5_1+Lung_1.Lung_drug_1*Lung_1+Adipose_1.Adipose_drug_1*Adipose_1+Heart_1.Heart_drug_1*Heart_1+Muscle_1.Muscle_drug_1*Muscle_1+Skin_1.Skin_drug_1*Skin_1+Kidney_1.Kidney_drug_1*Kidney_1+Bone_1.Bone_drug_1*Bone_1+Testes_1.Testes_drug_1*Testes_1+Rest_1.Rest_drug_1*Rest_1+Gut_1.Gut_drug_1*Gut_1+Spleen_1.Spleen_drug_1*Spleen_1+Brain_1.Brain_drug_1*Brain_1+Main_compartment_1.Bile_drug	0

	Repeated Assignments	Initial Value
	_1+Metabolites_1.Metabolites_drug_1+Urine_1.Urine_drug_1+Gut_Lumen_1.Gut_Lumen_drug_1	
71	Plasma_total_1.Plasma_free_uM_1 = Plasma_total_1.Plasma_total_drug_1*drug_fuplasma_1/drug_molar_mass_1	0
72	Liver_total_1.Liver_blood_total_1 = (Liver_EC_S1_1.Liver_EC_S1_drug_1+Liver_EC_S2_1.Liver_EC_S2_drug_1+Liver_EC_S3_1.Liver_EC_S3_drug_1+Liver_EC_S4_1.Liver_EC_S4_drug_1+Liver_EC_S5_1.Liver_EC_S5_drug_1)/5	0
73	Liver_total_1.Liver_tissue_total_1 = (Liver_IC_S1_1.Liver_IC_S1_drug_1+Liver_IC_S2_1.Liver_IC_S2_drug_1+Liver_IC_S3_1.Liver_IC_S3_drug_1+Liver_IC_S4_1.Liver_IC_S4_drug_1+Liver_IC_S5_1.Liver_IC_S5_drug_1)/5	0
74	Liver_total_1.Liver_blood_free_1 = Liver_total_1.Liver_blood_total_1*drug_fuplasma_1/drug_BRP_1	0
75	Liver_total_1.Liver_tissue_free_uM_1 = Liver_total_1.Liver_tissue_total_1*drug_fuLiver_1/drug_molar_mass_1	0
76	convert_to_nmole_per_kg_1.Adipose_nmole_1 = Adipose_1.Adipose_drug_1*Adipose_1/drug_molar_mass_1*nanomole_per_mole*kilogram/phys_BW	0
77	convert_to_nmole_per_kg_1.Artery_nmole_1 = Artery_1.Artery_drug_1*Artery_1/drug_molar_mass_1*nanomole_per_mole*kilogram/phys_BW	0
78	convert_to_nmole_per_kg_1.Bone_nmole_1 = Bone_1.Bone_drug_1*Bone_1/drug_molar_mass_1*nanomole_per_mole*kilogram/phys_BW	0
79	convert_to_nmole_per_kg_1.Liver_EC1_nmole_1 = Liver_EC_S1_1.Liver_EC_S1_drug_1*Liver_EC_S1_1/drug_molar_mass_1*nanomole_per_mole*kilogram/phys_BW	0
80	convert_to_nmole_per_kg_1.Liver_IC1_nmole_1 = Liver_IC_S1_1.Liver_IC_S1_drug_1*Liver_IC_S1_1/drug_molar_mass_1*nanomole_per_mole*kilogram/phys_BW	0
81	convert_to_nmole_per_kg_1.Muscle_nmole_1 = Muscle_1.Muscle_drug_1*Muscle_1/drug_molar_mass_1*nanomole_per_mole*kilogram/phys_BW	0
82	convert_to_nmole_per_kg_1.Urine_nmole_1 = Urine_1.Urine_drug_1/drug_molar_mass_1*nanomole_per_mole*kilogram/phys_BW	0
83	convert_to_nmole_per_kg_1.Venous_nmole_1 = Venous_1.Venous_drug_1*Venous_1/drug_molar_mass_1*nanomole_per_mole*kilogram/phys_BW	0
84	convert_to_nmole_per_kg_1.Bile_nmole_1 = Main_compartment_1.Bile_drug_1/drug_molar_mass_1*nanomole_per_mole*kilogram/phys_BW	0
85	Plasma_total_1.Plasma_total_uM_1 = Plasma_total_1.Plasma_total_drug_1/drug_molar_mass_1	0
86	Liver_total_1.Liver_tissue_total_uM_1 = Liver_total_1.Liver_tissue_total_1/drug_molar_mass_1	0
87	convert_to_nmole_per_kg_1.Kidney_nmole_1 = Kidney_1.Kidney_drug_1*Kidney_1/drug_molar_mass_1*nanomole_per_mole*kilogram/phys_BW	0

	Repeated Assignments	Initial Value
88	convert_to_nmole_per_kg_1.Lung_nmole_1 = Lung_1.Lung_drug_1*Lung_1/drug_molar_mass_1*nanomole_per_mole*kilogram/phys_BW	0
89	convert_to_nmole_per_kg_1.Metabolites_nmole_1 = Metabolites_1.Metabolites_drug_1/drug_molar_mass_1*nanomole_per_mole*kilogram/phys_BW	0
90	convert_to_nmole_per_kg_1.Liver_IC2_nmole_1 = Liver_IC_S2_1.Liver_IC_S2_drug_1*Liver_IC_S2_1/drug_molar_mass_1*nanomole_per_mole*kilogram/phys_BW	0
91	convert_to_nmole_per_kg_1.Liver_EC2_nmole_1 = Liver_EC_S2_1.Liver_EC_S2_drug_1*Liver_EC_S2_1/drug_molar_mass_1*nanomole_per_mole*kilogram/phys_BW	0
92	convert_to_nmole_per_kg_1.Liver_EC3_nmole_1 = Liver_EC_S3_1.Liver_EC_S3_drug_1*Liver_EC_S3_1/drug_molar_mass_1*nanomole_per_mole*kilogram/phys_BW	0
93	convert_to_nmole_per_kg_1.Liver_IC3_nmole_1 = Liver_IC_S3_1.Liver_IC_S3_drug_1*Liver_IC_S3_1/drug_molar_mass_1*nanomole_per_mole*kilogram/phys_BW	0
94	convert_to_nmole_per_kg_1.Liver_IC4_nmole_1 = Liver_IC_S4_1.Liver_IC_S4_drug_1*Liver_IC_S4_1/drug_molar_mass_1*nanomole_per_mole*kilogram/phys_BW	0
95	convert_to_nmole_per_kg_1.Liver_EC4_nmole_1 = Liver_EC_S4_1.Liver_EC_S4_drug_1*Liver_EC_S4_1/drug_molar_mass_1*nanomole_per_mole*kilogram/phys_BW	0
96	convert_to_nmole_per_kg_1.Liver_EC5_nmole_1 = Liver_EC_S5_1.Liver_EC_S5_drug_1*Liver_EC_S5_1/drug_molar_mass_1*nanomole_per_mole*kilogram/phys_BW	0
97	convert_to_nmole_per_kg_1.Liver_IC5_nmole_1 = Liver_IC_S5_1.Liver_IC_S5_drug_1*Liver_IC_S5_1/drug_molar_mass_1*nanomole_per_mole*kilogram/phys_BW	0
98	convert_to_nmole_per_kg_1.Gut_nmole_1 = Gut_1.Gut_drug_1*Gut_1/drug_molar_mass_1*nanomole_per_mole*kilogram/phys_BW	0
99	convert_to_nmole_per_kg_1.Spleen_nmole_1 = Spleen_1.Spleen_drug_1*Spleen_1/drug_molar_mass_1*nanomole_per_mole*kilogram/phys_BW	0
100	convert_to_nmole_per_kg_1.Skin_nmole_1 = Skin_1.Skin_drug_1*Skin_1/drug_molar_mass_1*nanomole_per_mole*kilogram/phys_BW	0
101	convert_to_nmole_per_kg_1.Brain_nmole_1 = Brain_1.Brain_drug_1*Brain_1/drug_molar_mass_1*nanomole_per_mole*kilogram/phys_BW	0
102	convert_to_nmole_per_kg_1.Rest_nmole_1 = Rest_1.Rest_drug_1*Rest_1/drug_molar_mass_1*nanomole_per_mole*kilogram/phys_BW	0
103	convert_to_nmole_per_kg_1.Heart_nmole_1 = Heart_1.Heart_drug_1*Heart_1/drug_molar_mass_1*nanomole_per_mole*kilogram/phys_BW	0
104	uptake_inhib_S1 = (1-switch_uptake_inhib_1)+switch_uptake_inhib_1/(1+drug_fB*Liver_EC_S1.Liver_EC_S1_drug/(drug_uptake_Ki*drug_molar_mass))	1
105	uptake_inhib_S3 = (1-switch_uptake_inhib_1)+switch_uptake_inhib_1/(1+drug_f	1

	Repeated Assignments	Initial Value
	B*Liver_EC_S3.Liver_EC_S3_drug/(drug_uptake_Ki*drug_molar_mass))	
106	uptake_inhib_S2 = (1-switch_uptake_inhib_1)+switch_uptake_inhib_1/(1+drug_fB*Liver_EC_S2.Liver_EC_S2_drug/(drug_uptake_Ki*drug_molar_mass))	1
107	uptake_inhib_S4 = (1-switch_uptake_inhib_1)+switch_uptake_inhib_1/(1+drug_fB*Liver_EC_S4.Liver_EC_S4_drug/(drug_uptake_Ki*drug_molar_mass))	1
108	uptake_inhib_S5 = (1-switch_uptake_inhib_1)+switch_uptake_inhib_1/(1+drug_fB*Liver_EC_S5.Liver_EC_S5_drug/(drug_uptake_Ki*drug_molar_mass))	1
109	biliary_inhib_S1 = (1-switch_biliary_inhib_1)+switch_biliary_inhib_1/(1+drug_fuLiver*Liver_IC_S1.Liver_IC_S1_drug/(drug_biliary_Ki*drug_molar_mass))	1
110	biliary_inhib_S2 = (1-switch_biliary_inhib_1)+switch_biliary_inhib_1/(1+drug_fuLiver*Liver_IC_S2.Liver_IC_S2_drug/(drug_biliary_Ki*drug_molar_mass))	1
111	biliary_inhib_S3 = (1-switch_biliary_inhib_1)+switch_biliary_inhib_1/(1+drug_fuLiver*Liver_IC_S3.Liver_IC_S3_drug/(drug_biliary_Ki*drug_molar_mass))	1
112	biliary_inhib_S4 = (1-switch_biliary_inhib_1)+switch_biliary_inhib_1/(1+drug_fuLiver*Liver_IC_S4.Liver_IC_S4_drug/(drug_biliary_Ki*drug_molar_mass))	1
113	biliary_inhib_S5 = (1-switch_biliary_inhib_1)+switch_biliary_inhib_1/(1+drug_fuLiver*Liver_IC_S5.Liver_IC_S5_drug/(drug_biliary_Ki*drug_molar_mass))	1
114	met_inhib_S1 = (1-switch_met_inhib_1)+switch_met_inhib_1/(1+drug_fuLiver*Liver_IC_S1.Liver_IC_S1_drug/(drug_met_Ki*drug_molar_mass))	1
115	met_inhib_S2 = (1-switch_met_inhib_1)+switch_met_inhib_1/(1+drug_fuLiver*Liver_IC_S2.Liver_IC_S2_drug/(drug_met_Ki*drug_molar_mass))	1
116	met_inhib_S3 = (1-switch_met_inhib_1)+switch_met_inhib_1/(1+drug_fuLiver*Liver_IC_S3.Liver_IC_S3_drug/(drug_met_Ki*drug_molar_mass))	1
117	met_inhib_S4 = (1-switch_met_inhib_1)+switch_met_inhib_1/(1+drug_fuLiver*Liver_IC_S4.Liver_IC_S4_drug/(drug_met_Ki*drug_molar_mass))	1
118	met_inhib_S5 = (1-switch_met_inhib_1)+switch_met_inhib_1/(1+drug_fuLiver*Liver_IC_S5.Liver_IC_S5_drug/(drug_met_Ki*drug_molar_mass))	1

	Repeated Assignments	Initial Value
119	Kpuu_Liver_1 = Liver_total_1.Liver_tissue_free_uM_1/Plasma_total_1.Plasma_free_uM_1	NaN
120	memduo_conc = MEM_DUO/MDUO/drug_molar_mass*fu_mem	0
121	memjej1_conc = MEM_JEJ1/MJEJ1/drug_molar_mass*fu_mem	0
122	memjej2_conc = MEM_JEJ2/MJEJ2/drug_molar_mass*fu_mem	0
123	memill1_conc = MEM_ILL1/MILL1/drug_molar_mass*fu_mem	0
124	memill2_conc = MEM_ILL2/MILL2/drug_molar_mass*fu_mem	0
125	memill3_conc = MEM_ILL3/MILL3/drug_molar_mass*fu_mem	0
126	memill4_conc = MEM_ILL4/MILL4/drug_molar_mass*fu_mem	0
127	Villous_conc_calc.vduo_conc = Villous_DUO/VillousDUO/drug_molar_mass*fu_mem	0
128	Villous_conc_calc.vjej1_conc = Villous_JEJ1/VillousJEJ1/drug_molar_mass*fu_mem	0
129	Villous_conc_calc.vjej2_conc = Villous_JEJ2/VillousJEJ2/drug_molar_mass*fu_mem	0
130	Villous_conc_calc.vill1_conc = Villous_ILL1/VillousILL1/drug_molar_mass*fu_mem	0
131	Villous_conc_calc.vill2_conc = Villous_ILL2/VillousILL2/drug_molar_mass*fu_mem	0
132	Villous_conc_calc.vill3_conc = Villous_ILL3/VillousILL3/drug_molar_mass*fu_mem	0
133	Villous_conc_calc.vill4_conc = Villous_ILL4/VillousILL4/drug_molar_mass*fu_mem	0

Reactions

	Reactions
1	Liver_IC_S5.Liver_IC_S5_drug -> null
	k_Liver_IC_S5_Bile*Liver_IC_S5.Liver_IC_S5_drug
2	Liver_IC_S5.Liver_IC_S5_drug -> Metabolites.Metabolites_drug
	k_Liver_IC_S5_Metabolites*Liver_IC_S5.Liver_IC_S5_drug
3	Liver_IC_S4.Liver_IC_S4_drug -> Metabolites.Metabolites_drug
	k_Liver_IC_S4_Metabolites*Liver_IC_S4.Liver_IC_S4_drug
4	Liver_IC_S3.Liver_IC_S3_drug -> Metabolites.Metabolites_drug
	k_Liver_IC_S3_Metabolites*Liver_IC_S3.Liver_IC_S3_drug
5	Liver_IC_S2.Liver_IC_S2_drug -> Metabolites.Metabolites_drug
	k_Liver_IC_S2_Metabolites*Liver_IC_S2.Liver_IC_S2_drug
6	Liver_IC_S1.Liver_IC_S1_drug -> Metabolites.Metabolites_drug
	k_Liver_IC_S1_Metabolites*Liver_IC_S1.Liver_IC_S1_drug
7	Liver_IC_S4.Liver_IC_S4_drug -> null

	Reactions
	k_Liver_IC_S4_Bile*Liver_IC_S4.Liver_IC_S4_drug
8	Liver_IC_S3.Liver_IC_S3_drug -> null
	k_Liver_IC_S3_Bile*Liver_IC_S3.Liver_IC_S3_drug
9	Liver_IC_S2.Liver_IC_S2_drug -> null
	k_Liver_IC_S2_Bile*Liver_IC_S2.Liver_IC_S2_drug
10	Liver_IC_S1.Liver_IC_S1_drug -> null
	k_Liver_IC_S1_Bile*Liver_IC_S1.Liver_IC_S1_drug
11	Liver_IC_S5.Liver_IC_S5_drug -> Liver_EC_S5.Liver_EC_S5_drug
	k_Liver_IC_S5_Liver_EC_S5*Liver_IC_S5.Liver_IC_S5_drug
12	Liver_EC_S5.Liver_EC_S5_drug -> Liver_IC_S5.Liver_IC_S5_drug
	k_Liver_EC_S5_Liver_IC_S5*Liver_EC_S5.Liver_EC_S5_drug
13	Liver_IC_S4.Liver_IC_S4_drug -> Liver_EC_S4.Liver_EC_S4_drug
	k_Liver_IC_S4_Liver_EC_S4*Liver_IC_S4.Liver_IC_S4_drug
14	Liver_EC_S4.Liver_EC_S4_drug -> Liver_IC_S4.Liver_IC_S4_drug
	k_Liver_EC_S4_Liver_IC_S4*Liver_EC_S4.Liver_EC_S4_drug
15	Liver_IC_S3.Liver_IC_S3_drug -> Liver_EC_S3.Liver_EC_S3_drug
	k_Liver_IC_S3_Liver__EC_S3*Liver_IC_S3.Liver_IC_S3_drug
16	Liver_EC_S3.Liver_EC_S3_drug -> Liver_IC_S3.Liver_IC_S3_drug
	k_Liver_EC_S3_Liver_IC_S3*Liver_EC_S3.Liver_EC_S3_drug
17	Liver_IC_S2.Liver_IC_S2_drug -> Liver_EC_S2.Liver_EC_S2_drug
	k_Liver_IC_S2_Liver_EC_S2*Liver_IC_S2.Liver_IC_S2_drug
18	Liver_EC_S2.Liver_EC_S2_drug -> Liver_IC_S2.Liver_IC_S2_drug
	k_Liver_EC_S2_Liver_IC_S2*Liver_EC_S2.Liver_EC_S2_drug
19	Liver_IC_S1.Liver_IC_S1_drug -> Liver_EC_S1.Liver_EC_S1_drug
	k_Liver_IC_S1_Liver_EC_S1*Liver_IC_S1.Liver_IC_S1_drug
20	Liver_EC_S1.Liver_EC_S1_drug -> Liver_IC_S1.Liver_IC_S1_drug
	k_Liver_EC_S1_Liver_IC_S1*Liver_EC_S1.Liver_EC_S1_drug
21	Liver.Liver_drug -> Venous.Venous_drug
	k_Liver_Venous*Liver.Liver_drug
22	Liver_EC_S4.Liver_EC_S4_drug -> Liver_EC_S5.Liver_EC_S5_drug
	k_Liver_EC_S4_Liver_EC_S5*Liver_EC_S4.Liver_EC_S4_drug
23	Liver_EC_S3.Liver_EC_S3_drug -> Liver_EC_S4.Liver_EC_S4_drug
	k_Liver_EC_S3_Liver_EC_S4*Liver_EC_S3.Liver_EC_S3_drug
24	Liver_EC_S2.Liver_EC_S2_drug -> Liver_EC_S3.Liver_EC_S3_drug
	k_Liver_EC_S2_Liver_EC_S3*Liver_EC_S2.Liver_EC_S2_drug
25	Liver_EC_S1.Liver_EC_S1_drug -> Liver_EC_S2.Liver_EC_S2_drug
	k_Liver_EC_S1_Liver_EC_S2*Liver_EC_S1.Liver_EC_S1_drug
26	Artery.Artery_drug -> Liver.Liver_drug
	k_artery_liver*Artery.Artery_drug
27	Artery.Artery_drug -> VillousILL1.Villous_ILL1
	Qmuc_ILL1*Artery.Artery_drug

	Reactions
28	Artery.Artery_drug -> Spleen.Spleen_drug
	*k_artery_spleen*Artery.Artery_drug*
29	Rest.Rest_drug -> Venous.Venous_drug
	*k_rest_venous*Rest.Rest_drug*
30	Bone.Bone_drug -> Venous.Venous_drug
	*k_bone_venous*Bone.Bone_drug*
31	Skin.Skin_drug -> Venous.Venous_drug
	*k_skin_venous*Skin.Skin_drug*
32	Heart.Heart_drug -> Venous.Venous_drug
	*k_heart_venous*Heart.Heart_drug*
33	Adipose.Adipose_drug -> Venous.Venous_drug
	*k_adipos_venous*Adipose.Adipose_drug*
34	Muscle.Muscle_drug -> Venous.Venous_drug
	*k_muscle_venous*Muscle.Muscle_drug*
35	Brain.Brain_drug -> Venous.Venous_drug
	*k_brain_venous*Brain.Brain_drug*
36	Kidney.Kidney_drug -> Venous.Venous_drug
	*k_kidney_venous*Kidney.Kidney_drug*
37	Artery.Artery_drug -> Rest.Rest_drug
	*k_artery_rest*Artery.Artery_drug*
38	Artery.Artery_drug -> Bone.Bone_drug
	*k_artery_bone*Artery.Artery_drug*
39	Artery.Artery_drug -> Skin.Skin_drug
	*k_artery_skin*Artery.Artery_drug*
40	Artery.Artery_drug -> Heart.Heart_drug
	*k_artery_heart*Artery.Artery_drug*
41	Artery.Artery_drug -> Adipose.Adipose_drug
	*k_artery_adipos*Artery.Artery_drug*
42	Artery.Artery_drug -> Muscle.Muscle_drug
	*k_artery_muscle*Artery.Artery_drug*
43	Artery.Artery_drug -> Brain.Brain_drug
	*k_artery_brain*Artery.Artery_drug*
44	Artery.Artery_drug -> Kidney.Kidney_drug
	*k_artery_kidney*Artery.Artery_drug*
45	Venous.Venous_drug -> Lung.Lung_drug
	*k_venous_lung*Venous.Venous_drug*
46	Lung.Lung_drug -> Artery.Artery_drug
	*k_lung_artery*Lung.Lung_drug*
47	Venous.Venous_drug -> Urine.Urine_drug
	*k_venous_urine_CLR*Venous.Venous_drug*
48	Artery.Artery_drug -> Testes.Testes_drug

	Reactions
	*k_artery_testes*Artery.Artery_drug*
49	Testes.Testes_drug -> Venous.Venous_drug
	*k_testes_venous*Testes.Testes_drug*
50	Liver_IC_S4.Liver_IC_S4_drug -> Liver_EC_S4.Liver_EC_S4_drug
	*k_Liver_IC_S4_Liver_EC_S4_efflux*Liver_IC_S4.Liver_IC_S4_drug*
51	Liver_IC_S3.Liver_IC_S3_drug -> Liver_EC_S3.Liver_EC_S3_drug
	*k_Liver_IC_S3_Liver_EC_S3_efflux*Liver_IC_S3.Liver_IC_S3_drug*
52	Liver_IC_S2.Liver_IC_S2_drug -> Liver_EC_S2.Liver_EC_S2_drug
	*k_Liver_IC_S2_Liver_EC_S2_efflux*Liver_IC_S2.Liver_IC_S2_drug*
53	Liver_IC_S5.Liver_IC_S5_drug -> Liver_EC_S5.Liver_EC_S5_drug
	*k_Liver_IC_S5_Liver_EC_S5_efflux*Liver_IC_S5.Liver_IC_S5_drug*
54	Liver_IC_S1.Liver_IC_S1_drug -> Liver_EC_S1.Liver_EC_S1_drug
	*k_Liver_IC_S1_Liver_EC_S1_efflux*Liver_IC_S1.Liver_IC_S1_drug*
55	Venous.Venous_drug -> Urine.Urine_drug
	*k_venous_urine_GFR*Venous.Venous_drug*
56	Liver_IC_S5_1.Liver_IC_S5_drug_1 -> Main_compartment_1.Bile_drug_1
	*k_Liver_IC_S5_Bile_1*Liver_IC_S5_1.Liver_IC_S5_drug_1*
57	Liver_IC_S5_1.Liver_IC_S5_drug_1 -> Metabolites_1.Metabolites_drug_1
	*k_Liver_IC_S5_Metabolites_1*Liver_IC_S5_1.Liver_IC_S5_drug_1*
58	Liver_IC_S4_1.Liver_IC_S4_drug_1 -> Metabolites_1.Metabolites_drug_1
	*k_Liver_IC_S4_Metabolites_1*Liver_IC_S4_1.Liver_IC_S4_drug_1*
59	Liver_IC_S3_1.Liver_IC_S3_drug_1 -> Metabolites_1.Metabolites_drug_1
	*k_Liver_IC_S3_Metabolites_1*Liver_IC_S3_1.Liver_IC_S3_drug_1*
60	Liver_IC_S2_1.Liver_IC_S2_drug_1 -> Metabolites_1.Metabolites_drug_1
	*k_Liver_IC_S2_Metabolites_1*Liver_IC_S2_1.Liver_IC_S2_drug_1*
61	Liver_IC_S1_1.Liver_IC_S1_drug_1 -> Metabolites_1.Metabolites_drug_1
	*k_Liver_IC_S1_Metabolites_1*Liver_IC_S1_1.Liver_IC_S1_drug_1*
62	Liver_IC_S4_1.Liver_IC_S4_drug_1 -> Main_compartment_1.Bile_drug_1
	*k_Liver_IC_S4_Bile_1*Liver_IC_S4_1.Liver_IC_S4_drug_1*
63	Liver_IC_S3_1.Liver_IC_S3_drug_1 -> Main_compartment_1.Bile_drug_1
	*k_Liver_IC_S3_Bile_1*Liver_IC_S3_1.Liver_IC_S3_drug_1*
64	Liver_IC_S2_1.Liver_IC_S2_drug_1 -> Main_compartment_1.Bile_drug_1
	*k_Liver_IC_S2_Bile_1*Liver_IC_S2_1.Liver_IC_S2_drug_1*
65	Liver_IC_S1_1.Liver_IC_S1_drug_1 -> Main_compartment_1.Bile_drug_1
	*k_Liver_IC_S1_Bile_1*Liver_IC_S1_1.Liver_IC_S1_drug_1*
66	Liver_IC_S5_1.Liver_IC_S5_drug_1 -> Liver_EC_S5_1.Liver_EC_S5_drug_1
	*k_Liver_IC_S5_Liver_EC_S5_1*Liver_IC_S5_1.Liver_IC_S5_drug_1*
67	Liver_EC_S5_1.Liver_EC_S5_drug_1 -> Liver_IC_S5_1.Liver_IC_S5_drug_1
	*k_Liver_EC_S5_Liver_IC_S5_1*Liver_EC_S5_1.Liver_EC_S5_drug_1*
68	Liver_IC_S4_1.Liver_IC_S4_drug_1 -> Liver_EC_S4_1.Liver_EC_S4_drug_1
	*k_Liver_IC_S4_Liver_EC_S4_1*Liver_IC_S4_1.Liver_IC_S4_drug_1*

	Reactions
69	Liver_EC_S4_1.Liver_EC_S4_drug_1 -> Liver_IC_S4_1.Liver_IC_S4_drug_1
	k_Liver_EC_S4_Liver_IC_S4_1*Liver_EC_S4_1.Liver_EC_S4_drug_1
70	Liver_IC_S3_1.Liver_IC_S3_drug_1 -> Liver_EC_S3_1.Liver_EC_S3_drug_1
	k_Liver_IC_S3_Liver__EC_S3_1*Liver_IC_S3_1.Liver_IC_S3_drug_1
71	Liver_EC_S3_1.Liver_EC_S3_drug_1 -> Liver_IC_S3_1.Liver_IC_S3_drug_1
	k_Liver_EC_S3_Liver_IC_S3_1*Liver_EC_S3_1.Liver_EC_S3_drug_1
72	Liver_IC_S2_1.Liver_IC_S2_drug_1 -> Liver_EC_S2_1.Liver_EC_S2_drug_1
	k_Liver_IC_S2_Liver_EC_S2_1*Liver_IC_S2_1.Liver_IC_S2_drug_1
73	Liver_EC_S2_1.Liver_EC_S2_drug_1 -> Liver_IC_S2_1.Liver_IC_S2_drug_1
	k_Liver_EC_S2_Liver_IC_S2_1*Liver_EC_S2_1.Liver_EC_S2_drug_1
74	Liver_IC_S1_1.Liver_IC_S1_drug_1 -> Liver_EC_S1_1.Liver_EC_S1_drug_1
	k_Liver_IC_S1_Liver_EC_S1_1*Liver_IC_S1_1.Liver_IC_S1_drug_1
75	Liver_EC_S1_1.Liver_EC_S1_drug_1 -> Liver_IC_S1_1.Liver_IC_S1_drug_1
	k_Liver_EC_S1_Liver_IC_S1_1*Liver_EC_S1_1.Liver_EC_S1_drug_1
76	Liver_EC_S5_1.Liver_EC_S5_drug_1 -> Venous_1.Venous_drug_1
	k_Liver_EC_S5_Venous_1*Liver_EC_S5_1.Liver_EC_S5_drug_1
77	Liver_EC_S4_1.Liver_EC_S4_drug_1 -> Liver_EC_S5_1.Liver_EC_S5_drug_1
	k_Liver_EC_S4_Liver_EC_S5_1*Liver_EC_S4_1.Liver_EC_S4_drug_1
78	Liver_EC_S3_1.Liver_EC_S3_drug_1 -> Liver_EC_S4_1.Liver_EC_S4_drug_1
	k_Liver_EC_S3_Liver_EC_S4_1*Liver_EC_S3_1.Liver_EC_S3_drug_1
79	Liver_EC_S2_1.Liver_EC_S2_drug_1 -> Liver_EC_S3_1.Liver_EC_S3_drug_1
	k_Liver_EC_S2_Liver_EC_S3_1*Liver_EC_S2_1.Liver_EC_S2_drug_1
80	Liver_EC_S1_1.Liver_EC_S1_drug_1 -> Liver_EC_S2_1.Liver_EC_S2_drug_1
	k_Liver_EC_S1_Liver_EC_S2_1*Liver_EC_S1_1.Liver_EC_S1_drug_1
81	Gut_1.Gut_drug_1 -> Liver_EC_S1_1.Liver_EC_S1_drug_1
	k_gut_liver_1*Gut_1.Gut_drug_1
82	Spleen_1.Spleen_drug_1 -> Liver_EC_S1_1.Liver_EC_S1_drug_1
	k_spleen_liver_1*Spleen_1.Spleen_drug_1
83	Artery_1.Artery_drug_1 -> Liver_EC_S1_1.Liver_EC_S1_drug_1
	k_artery_liver_1*Artery_1.Artery_drug_1
84	Artery_1.Artery_drug_1 -> Gut_1.Gut_drug_1
	k_artery_gut_1*Artery_1.Artery_drug_1
85	Artery_1.Artery_drug_1 -> Spleen_1.Spleen_drug_1
	k_artery_spleen_1*Artery_1.Artery_drug_1
86	Rest_1.Rest_drug_1 -> Venous_1.Venous_drug_1
	k_rest_venous_1*Rest_1.Rest_drug_1
87	Bone_1.Bone_drug_1 -> Venous_1.Venous_drug_1
	k_bone_venous_1*Bone_1.Bone_drug_1
88	Skin_1.Skin_drug_1 -> Venous_1.Venous_drug_1
	k_skin_venous_1*Skin_1.Skin_drug_1
89	Heart_1.Heart_drug_1 -> Venous_1.Venous_drug_1

	Reactions
	*k_heart_venous_1*Heart_1.Heart_drug_1*
90	Adipose_1.Adipose_drug_1 -> Venous_1.Venous_drug_1
	*k_adipos_venous_1*Adipose_1.Adipose_drug_1*
91	Muscle_1.Muscle_drug_1 -> Venous_1.Venous_drug_1
	*k_muscle_venous_1*Muscle_1.Muscle_drug_1*
92	Brain_1.Brain_drug_1 -> Venous_1.Venous_drug_1
	*k_brain_venous_1*Brain_1.Brain_drug_1*
93	Kidney_1.Kidney_drug_1 -> Venous_1.Venous_drug_1
	*k_kidney_venous_1*Kidney_1.Kidney_drug_1*
94	Artery_1.Artery_drug_1 -> Rest_1.Rest_drug_1
	*k_artery_rest_1*Artery_1.Artery_drug_1*
95	Artery_1.Artery_drug_1 -> Bone_1.Bone_drug_1
	*k_artery_bone_1*Artery_1.Artery_drug_1*
96	Artery_1.Artery_drug_1 -> Skin_1.Skin_drug_1
	*k_artery_skin_1*Artery_1.Artery_drug_1*
97	Artery_1.Artery_drug_1 -> Heart_1.Heart_drug_1
	*k_artery_heart_1*Artery_1.Artery_drug_1*
98	Artery_1.Artery_drug_1 -> Adipose_1.Adipose_drug_1
	*k_artery_adipos_1*Artery_1.Artery_drug_1*
99	Artery_1.Artery_drug_1 -> Muscle_1.Muscle_drug_1
	*k_artery_muscle_1*Artery_1.Artery_drug_1*
100	Artery_1.Artery_drug_1 -> Brain_1.Brain_drug_1
	*k_artery_brain_1*Artery_1.Artery_drug_1*
101	Artery_1.Artery_drug_1 -> Kidney_1.Kidney_drug_1
	*k_artery_kidney_1*Artery_1.Artery_drug_1*
102	Venous_1.Venous_drug_1 -> Lung_1.Lung_drug_1
	*k_venous_lung_1*Venous_1.Venous_drug_1*
103	Lung_1.Lung_drug_1 -> Artery_1.Artery_drug_1
	*k_lung_artery_1*Lung_1.Lung_drug_1*
104	Venous_1.Venous_drug_1 -> Urine_1.Urine_drug_1
	*k_venous_urine_CLR_1*Venous_1.Venous_drug_1*
105	Artery_1.Artery_drug_1 -> Testes_1.Testes_drug_1
	*k_artery_testes_1*Artery_1.Artery_drug_1*
106	Testes_1.Testes_drug_1 -> Venous_1.Venous_drug_1
	*k_testes_venous_1*Testes_1.Testes_drug_1*
107	Venous_1.Venous_drug_1 -> Urine_1.Urine_drug_1
	*k_venous_urine_GFR_1*Venous_1.Venous_drug_1*
108	Liver_IC_S5_1.Liver_IC_S5_drug_1 -> Liver_EC_S5_1.Liver_EC_S5_drug_1
	*k_Liver_IC_S5_Liver_EC_S5_efflux_1*Liver_IC_S5_1.Liver_IC_S5_drug_1*
109	Liver_IC_S4_1.Liver_IC_S4_drug_1 -> Liver_EC_S4_1.Liver_EC_S4_drug_1
	*k_Liver_IC_S4_Liver_EC_S4_efflux_1*Liver_IC_S4_1.Liver_IC_S4_drug_1*

	Reactions
110	Liver_IC_S3_1.Liver_IC_S3_drug_1 -> Liver_EC_S3_1.Liver_EC_S3_drug_1
	*k_Liver_IC_S3_Liver_EC_S3_efflux_1*Liver_IC_S3_1.Liver_IC_S3_drug_1*
111	Liver_IC_S2_1.Liver_IC_S2_drug_1 -> Liver_EC_S2_1.Liver_EC_S2_drug_1
	*k_Liver_IC_S2_Liver_EC_S2_efflux_1*Liver_IC_S2_1.Liver_IC_S2_drug_1*
112	Liver_IC_S1_1.Liver_IC_S1_drug_1 -> Liver_EC_S1_1.Liver_EC_S1_drug_1
	*k_Liver_IC_S1_Liver_EC_S1_efflux_1*Liver_IC_S1_1.Liver_IC_S1_drug_1*
113	Main_compartment.Bile_drug -> VDUO.X_DUO_DISS
	*k_transit*Main_compartment.Bile_drug*
114	Gut_Lumen.Gut_Lumen_drug -> Gut.Gut_drug
	*drug_k_oral*drug_fa*Gut_Lumen.Gut_Lumen_drug*
115	Main_compartment.Bile_drug -> null
	*drug_k_bile_deg*Main_compartment.Bile_drug*
116	Gut_Lumen.Gut_Lumen_drug -> null
	drug_k_oral(1-drug_fa)*Gut_Lumen.Gut_Lumen_drug*
117	Gut.Gut_drug -> null
	*k_gut_liver*Gut.Gut_drug*
118	Spleen.Spleen_drug -> Liver.Liver_drug
	*k_spleen_liver*Spleen.Spleen_drug*
119	Gut_Lumen_1.Gut_Lumen_drug_1 -> Gut_1.Gut_drug_1
	*drug_k_oral_1*drug_fa_1*Gut_Lumen_1.Gut_Lumen_drug_1*
120	Main_compartment_1.Bile_drug_1 -> Gut_Lumen_1.Gut_Lumen_drug_1
	*k_transit_1*Main_compartment_1.Bile_drug_1*
121	Main_compartment_1.Bile_drug_1 -> null
	*drug_k_bile_deg_1*Main_compartment_1.Bile_drug_1*
122	Gut_Lumen_1.Gut_Lumen_drug_1 -> null
	drug_k_oral_1(1-drug_fa_1)*Gut_Lumen_1.Gut_Lumen_drug_1*
123	STOMACH.X_STOMACH_SOLID -> VDUO.X_DUO_SOLID
	STOMACH.X_STOMACH_SOLID/TSTOMACH
124	STOMACH.X_STOMACH_DISS -> VDUO.X_DUO_DISS
	STOMACH.X_STOMACH_DISS/TSTOMACH
125	VDUO.X_DUO_SOLID -> VJEJ1.X_JEJ1_SOLID
	X_DUO_SOLID/TDUO
126	VDUO.X_DUO_SOLID -> VDUO.X_DUO_DISS
	*KD*X_DUO_SOLID*(SOLIF_DUO-X_DUO_DISS/VDUO)*
127	VDUO.X_DUO_DISS -> VJEJ1.X_JEJ1_DISS
	X_DUO_DISS/TDUO
128	VDUO.X_DUO_DISS -> MDUO.MEM_DUO
	*(DIFF_duo*NI_DUO*switch_SFdiffapi*X_DUO_DISS)/VDUO*
129	MDUO.MEM_DUO + Eliminated.AMT_ELIM_DUO -> VDUO.X_DUO_DISS
	*((switchVmax_efflux==zero)*CLINT_efflux_DUO*efflux_factor_duo*switch_SFefflux+switchVmax_efflux*ESA*surfaceRatio_DUO*efflux_factor_duo*switch_SFefflux/(drug_Km_efflux+MEM_DUO*fu_mem/MDUO/drug_molar_mass))*MEM_DUO*fu_mem/MDUO*

	Reactions
130	VDUO.X_DUO_DISS -> MDUO.MEM_DUO + Eliminated.AMT_ELIM_DUO
	*((switchVmax_influx==zero)*CLINT_influx_DUO*influx_factor_duo*switch_SFinflux+switchVmax_influx*ESA*surfaceRatio_DUO*influx_factor_duo*switch_SFinflux/(drug_Km_influx+X_DUO_DISS/VDUO/drug_molar_mass))*X_DUO_DISS/VDUO*
131	VillousDUO.Villous_DUO -> Liver.Liver_drug
	*Villous_DUO*Qmuc_DUO/VillousDUO*
132	MDUO.MEM_DUO -> Eliminated.AMT_ELIM_DUO + Eliminated.AMT_ELIM_gut
	*(CLINT_metabolism*metabolism_factor_duo*switch_SFgutmet*MEM_DUO*fu_mem)/MDUO*
133	VJEJ1.X_JEJ1_SOLID -> VJEJ2.X_JEJ2_SOLID
	X_JEJ1_SOLID/TJEJ1
134	VJEJ1.X_JEJ1_SOLID -> VJEJ1.X_JEJ1_DISS
	*KD*X_JEJ1_SOLID*(SOLIF_JEJ1-X_JEJ1_DISS/VJEJ1)*
135	VJEJ1.X_JEJ1_DISS -> VJEJ2.X_JEJ2_DISS
	X_JEJ1_DISS/TJEJ1
136	VJEJ1.X_JEJ1_DISS -> MJEJ1.MEM_JEJ1
	*(DIFF_jej1*NI_JEJ1*switch_SFdiffapi*X_JEJ1_DISS)/VJEJ1*
137	MJEJ1.MEM_JEJ1 + Eliminated.AMT_ELIM_JEJ1 -> VJEJ1.X_JEJ1_DISS
	*((switchVmax_efflux==zero)*CLINT_efflux_JEJ1*efflux_factor_jej1*switch_SFefflux+switchVmax_efflux*ESA*surfaceRatio_JEJ1*efflux_factor_jej1*switch_SFefflux/(drug_Km_efflux+MEM_JEJ1*fu_mem/MJEJ1/drug_molar_mass))*MEM_JEJ1*fu_mem/MJEJ1*
138	VJEJ1.X_JEJ1_DISS -> MJEJ1.MEM_JEJ1 + Eliminated.AMT_ELIM_JEJ1
	*((switchVmax_influx==zero)*CLINT_influx_JEJ1*influx_factor_jej1*switch_SFinflux+switchVmax_influx*ESA*surfaceRatio_JEJ1*influx_factor_jej1*switch_SFinflux/(drug_Km_influx+X_JEJ1_DISS/VJEJ1/drug_molar_mass))*X_JEJ1_DISS/VJEJ1*
139	VillousJEJ1.Villous_JEJ1 -> Liver.Liver_drug
	*Villous_JEJ1*Qmuc_JEJ1/VillousJEJ1*
140	MJEJ1.MEM_JEJ1 -> Eliminated.AMT_ELIM_JEJ1 + Eliminated.AMT_ELIM_gut
	*(CLINT_metabolism*metabolism_factor_jej1*switch_SFgutmet*MEM_JEJ1*fu_mem)/MJEJ1*
141	VJEJ2.X_JEJ2_SOLID -> VILL1.X_ILL1_SOLID
	X_JEJ2_SOLID/TJEJ2
142	VJEJ2.X_JEJ2_SOLID -> VJEJ2.X_JEJ2_DISS
	*KD*X_JEJ2_SOLID*(SOLIF_JEJ2-X_JEJ2_DISS/VJEJ2)*
143	VJEJ2.X_JEJ2_DISS -> VILL1.X_ILL1_DISS
	X_JEJ2_DISS/TJEJ2
144	VJEJ2.X_JEJ2_DISS -> MJEJ2.MEM_JEJ2
	*(DIFF_jej2*NI_JEJ2*switch_SFdiffapi*X_JEJ2_DISS)/VJEJ2*
145	MJEJ2.MEM_JEJ2 + Eliminated.AMT_ELIM_JEJ2 -> VJEJ2.X_JEJ2_DISS
	*((switchVmax_efflux==zero)*CLINT_efflux_JEJ2*efflux_factor_jej2*switch_SFefflux+switchVmax_efflux*ESA*surfaceRatio_JEJ2*efflux_factor_jej2*switch_SFefflux/(drug_Km_efflux+MEM_JEJ2*fu_mem/MJEJ2/drug_molar_mass))*MEM_JEJ2*fu_mem/MJEJ2*
146	VJEJ2.X_JEJ2_DISS -> MJEJ2.MEM_JEJ2 + Eliminated.AMT_ELIM_JEJ2
	*((switchVmax_influx==zero)*CLINT_influx_JEJ2*influx_factor_jej2*switch_SFinflux+switchVmax_influx*ESA*surfaceRatio_JEJ2*influx_factor_jej2*switch_SFinflux/(drug_Km_influx+X_JEJ2_DISS/VJEJ2/drug_molar_mass))*X_JEJ2_DISS/VJEJ2*
147	VillousJEJ2.Villous_JEJ2 -> Liver.Liver_drug

	Reactions
	*Villous_JEJ2*Qmuc_JEJ2/VillousJEJ2*
148	MJEJ2.MEM_JEJ2 -> Eliminated.AMT_ELIM_JEJ2 + Eliminated.AMT_ELIM_gut
	*(CLINT_metabolism*metabolism_factor_jej2*switch_SFgutmet*MEM_JEJ2*fu_mem)/MJEJ2*
149	VILL1.X_ILL1_SOLID -> VILL2.X_ILL2_SOLID
	X_ILL1_SOLID/TILL1
150	VILL1.X_ILL1_SOLID -> VILL1.X_ILL1_DISS
	*KD*X_ILL1_SOLID*(SOLIF_ILL1-X_ILL1_DISS/VILL1)*
151	VILL1.X_ILL1_DISS -> VILL2.X_ILL2_DISS
	X_ILL1_DISS/TILL1
152	VILL1.X_ILL1_DISS -> MILL1.MEM_ILL1
	*(DIFF_ill1*NI_ILL1*switch_SFdiffapi*X_ILL1_DISS)/VILL1*
153	MILL1.MEM_ILL1 + Eliminated.AMT_ELIM_ILL1 -> VILL1.X_ILL1_DISS
	*((switchVmax_efflux==zero)*CLINT_efflux_ILL1*efflux_factor_ill1*switch_SFefflux_Lower+switchVmax_efflux*ESA*surfaceRatio_ILL1*efflux_factor_ill1*switch_SFefflux_Lower/(drug_Km_efflux+MEM_ILL1*fu_mem/MILL1/drug_molar_mass))*MEM_ILL1*fu_mem/MILL1*
154	VILL1.X_ILL1_DISS -> MILL1.MEM_ILL1 + Eliminated.AMT_ELIM_ILL1
	*((switchVmax_influx==zero)*CLINT_influx_ILL1*influx_factor_ill1*switch_SFinflux_Lower+switchVmax_influx*ESA*surfaceRatio_ILL1*influx_factor_ill1*switch_SFinflux_Lower/(drug_Km_influx+X_ILL1_DISS/VILL1/drug_molar_mass))*X_ILL1_DISS/VILL1*
155	VillousILL1.Villous_ILL1 -> Liver.Liver_drug
	*Villous_ILL1*Qmuc_ILL1/VillousILL1*
156	MILL1.MEM_ILL1 -> Eliminated.AMT_ELIM_ILL1 + Eliminated.AMT_ELIM_gut
	*(CLINT_metabolism*metabolism_factor_ill1*switch_SFgutmet*MEM_ILL1*fu_mem)/MILL1*
157	VILL2.X_ILL2_SOLID -> VILL3.X_ILL3_SOLID
	X_ILL2_SOLID/TILL2
158	VILL2.X_ILL2_SOLID -> VILL2.X_ILL2_DISS
	*KD*X_ILL2_SOLID*(SOLIF_ILL2-X_ILL2_DISS/VILL2)*
159	VILL2.X_ILL2_DISS -> VILL3.X_ILL3_DISS
	X_ILL2_DISS/TILL2
160	VILL2.X_ILL2_DISS -> MILL2.MEM_ILL2
	*(DIFF_ill2*NI_ILL2*switch_SFdiffapi*X_ILL2_DISS)/VILL2*
161	MILL2.MEM_ILL2 + Eliminated.AMT_ELIM_ILL2 -> VILL2.X_ILL2_DISS
	*((switchVmax_efflux==zero)*CLINT_efflux_ILL2*efflux_factor_ill2*switch_SFefflux_Lower+switchVmax_efflux*ESA*surfaceRatio_ILL2*efflux_factor_ill2*switch_SFefflux_Lower/(drug_Km_efflux+MEM_ILL2*fu_mem/MILL2/drug_molar_mass))*MEM_ILL2*fu_mem/MILL2*
162	VILL2.X_ILL2_DISS -> MILL2.MEM_ILL2 + Eliminated.AMT_ELIM_ILL2
	*((switchVmax_influx==zero)*CLINT_influx_ILL2*influx_factor_ill2*switch_SFinflux_Lower+switchVmax_influx*ESA*surfaceRatio_ILL2*influx_factor_ill2*switch_SFinflux_Lower/(drug_Km_influx+X_ILL2_DISS/VILL2/drug_molar_mass))*X_ILL2_DISS/VILL2*
163	VillousILL2.Villous_ILL2 -> Liver.Liver_drug
	*Villous_ILL2*Qmuc_ILL2/VillousILL2*
164	MILL2.MEM_ILL2 -> Eliminated.AMT_ELIM_ILL2 + Eliminated.AMT_ELIM_gut
	*(CLINT_metabolism*metabolism_factor_ill2*switch_SFgutmet*MEM_ILL2*fu_mem)/MILL2*
165	VILL3.X_ILL3_SOLID -> VILL4.X_ILL4_SOLID

	Reactions
	X_ILL3_SOLID/TILL3
166	VILL3.X_ILL3_SOLID -> VILL3.X_ILL3_DISS
	*KD*X_ILL3_SOLID*(SOLIF_ILL3-X_ILL3_DISS/VILL3)*
167	VILL3.X_ILL3_DISS -> VILL4.X_ILL4_DISS
	X_ILL3_DISS/TILL3
168	VILL3.X_ILL3_DISS -> MILL3.MEM_ILL3
	*(DIFF_ill3*NI_ILL3*switch_SFdiffapi*X_ILL3_DISS)/VILL3*
169	MILL3.MEM_ILL3 + Eliminated.AMT_ELIM_ILL3 -> VILL3.X_ILL3_DISS
	*((switchVmax_efflux==zero)*CLINT_efflux_ILL3*efflux_factor_ill3*switch_SFefflux_Lower+switchVmax_efflux*ESA*surfaceRatio_ILL3*efflux_factor_ill3*switch_SFefflux_Lower/(drug_Km_efflux+MEM_ILL3*fu_mem/MILL3/drug_molar_mass))*MEM_ILL3*fu_mem/MILL3*
170	VILL3.X_ILL3_DISS -> MILL3.MEM_ILL3 + Eliminated.AMT_ELIM_ILL3
	*((switchVmax_influx==zero)*CLINT_influx_ILL3*influx_factor_ill3*switch_SFinflux_Lower+switchVmax_influx*ESA*surfaceRatio_ILL3*influx_factor_ill3*switch_SFinflux_Lower/(drug_Km_influx+X_ILL3_DISS/VILL3/drug_molar_mass))*X_ILL3_DISS/VILL3*
171	VillousILL3.Villous_ILL3 -> Liver.Liver_drug
	*Villous_ILL3*Qmuc_ILL3/VillousILL3*
172	MILL3.MEM_ILL3 -> Eliminated.AMT_ELIM_ILL3 + Eliminated.AMT_ELIM_gut
	*(CLINT_metabolism*metabolism_factor_ill3*switch_SFgutmet*MEM_ILL3*fu_mem)/MILL3*
173	VILL4.X_ILL4_SOLID -> Colon.X_CECUM_SOLID
	X_ILL4_SOLID/TILL4
174	VILL4.X_ILL4_SOLID -> VILL4.X_ILL4_DISS
	*KD*X_ILL4_SOLID*(SOLIF_ILL4-X_ILL4_DISS/VILL4)*
175	VILL4.X_ILL4_DISS -> Colon.X_CECUM_DISS
	X_ILL4_DISS/TILL4
176	VILL4.X_ILL4_DISS -> MILL4.MEM_ILL4
	*(DIFF_ill4*NI_ILL4*switch_SFdiffapi*X_ILL4_DISS)/VILL4*
177	MILL4.MEM_ILL4 + Eliminated.AMT_ELIM_ILL4 -> VILL4.X_ILL4_DISS
	*((switchVmax_efflux==zero)*CLINT_efflux_ILL4*efflux_factor_ill4*switch_SFefflux_Lower+switchVmax_efflux*ESA*surfaceRatio_ILL4*efflux_factor_ill4*switch_SFefflux_Lower/(drug_Km_efflux+MEM_ILL4*fu_mem/MILL4/drug_molar_mass))*MEM_ILL4*fu_mem/MILL4*
178	VILL4.X_ILL4_DISS -> MILL4.MEM_ILL4 + Eliminated.AMT_ELIM_ILL4
	*((switchVmax_influx==zero)*CLINT_influx_ILL4*influx_factor_ill4*switch_SFinflux_Lower+switchVmax_influx*ESA*surfaceRatio_ILL4*influx_factor_ill4*switch_SFinflux_Lower/(drug_Km_influx+X_ILL4_DISS/VILL4/drug_molar_mass))*X_ILL4_DISS/VILL4*
179	VillousILL4.Villous_ILL4 -> Liver.Liver_drug
	*Villous_ILL4*Qmuc_ILL4/VillousILL4*
180	MILL4.MEM_ILL4 -> Eliminated.AMT_ELIM_ILL4 + Eliminated.AMT_ELIM_gut
	*(CLINT_metabolism*metabolism_factor_ill4*switch_SFgutmet*MEM_ILL4*fu_mem)/MILL4*
181	Artery.Artery_drug -> VillousDUO.Villous_DUO
	*Qmuc_DUO*Artery.Artery_drug*
182	Artery.Artery_drug -> VillousJEJ1.Villous_JEJ1
	*Qmuc_JEJ1*Artery.Artery_drug*
183	Artery.Artery_drug -> VillousJEJ2.Villous_JEJ2

	Reactions
	*Qmuc_JEJ2*Artery.Artery_drug*
184	Artery.Artery_drug -> VillousILL2.Villous_ILL2
	*Qmuc_ILL2*Artery.Artery_drug*
185	Artery.Artery_drug -> VillousILL3.Villous_ILL3
	*Qmuc_ILL3*Artery.Artery_drug*
186	Artery.Artery_drug -> VillousILL4.Villous_ILL4
	*Qmuc_ILL4*Artery.Artery_drug*
187	STOMACH.X_STOMACH_SOLID -> STOMACH.X_STOMACH_DISS
	*KD*STOMACH.X_STOMACH_SOLID*(SOLIF_STOMACH-STOMACH.X_STOMACH_DISS/STOMACH)*
188	MDUO.MEM_DUO -> VillousDUO.Villous_DUO
	*DIFF_BASO_duo*NI_Membrane*switch_SFdiffbaso*MDUO.MEM_DUO*fu_mem/MDUO*
189	VillousDUO.Villous_DUO -> MDUO.MEM_DUO
	*DIFF_BASO_duo*NI_Villous*switch_SFdiffbaso*VillousDUO.Villous_DUO*fu_blood/VillousDUO*
190	MDUO.MEM_DUO -> VDUO.X_DUO_DISS
	*DIFF_duo*NI_Membrane*switch_SFdiffapi*MDUO.MEM_DUO*fu_mem/MDUO*
191	MJEJ1.MEM_JEJ1 -> VillousJEJ1.Villous_JEJ1
	*DIFF_BASO_jej1*NI_Membrane*switch_SFdiffbaso*MJEJ1.MEM_JEJ1*fu_mem/MJEJ1*
192	VillousJEJ1.Villous_JEJ1 -> MJEJ1.MEM_JEJ1
	*DIFF_BASO_jej1*NI_Villous*switch_SFdiffbaso*VillousJEJ1.Villous_JEJ1*fu_blood/VillousJEJ1*
193	MJEJ1.MEM_JEJ1 -> VJEJ1.X_JEJ1_DISS
	*DIFF_jej1*NI_Membrane*switch_SFdiffapi*MJEJ1.MEM_JEJ1*fu_mem/MJEJ1*
194	MJEJ2.MEM_JEJ2 -> VillousJEJ2.Villous_JEJ2
	*DIFF_BASO_jej2*NI_Membrane*switch_SFdiffbaso*MJEJ2.MEM_JEJ2*fu_mem/MJEJ2*
195	VillousJEJ2.Villous_JEJ2 -> MJEJ2.MEM_JEJ2
	*DIFF_BASO_jej2*NI_Villous*switch_SFdiffbaso*VillousJEJ2.Villous_JEJ2*fu_blood/VillousJEJ2*
196	MJEJ2.MEM_JEJ2 -> VJEJ2.X_JEJ2_DISS
	*DIFF_jej2*NI_Membrane*switch_SFdiffapi*MJEJ2.MEM_JEJ2*fu_mem/MJEJ2*
197	MILL1.MEM_ILL1 -> VillousILL1.Villous_ILL1
	*DIFF_BASO_ill1*NI_Membrane*switch_SFdiffbaso*MILL1.MEM_ILL1*fu_mem/MILL1*
198	VillousILL1.Villous_ILL1 -> MILL1.MEM_ILL1
	*DIFF_BASO_ill1*NI_Villous*switch_SFdiffbaso*VillousILL1.Villous_ILL1*fu_blood/VillousILL1*
199	MILL1.MEM_ILL1 -> VILL1.X_ILL1_DISS
	*DIFF_ill1*NI_Membrane*switch_SFdiffapi*MILL1.MEM_ILL1*fu_mem/MILL1*
200	VillousILL2.Villous_ILL2 -> MILL2.MEM_ILL2
	*DIFF_BASO_ill2*NI_Villous*switch_SFdiffbaso*VillousILL2.Villous_ILL2*fu_blood/VillousILL2*
201	MILL2.MEM_ILL2 -> VillousILL2.Villous_ILL2
	*DIFF_BASO_ill2*NI_Membrane*switch_SFdiffbaso*MILL2.MEM_ILL2*fu_mem/MILL2*
202	MILL2.MEM_ILL2 -> VILL2.X_ILL2_DISS
	*DIFF_ill2*NI_Membrane*switch_SFdiffapi*MILL2.MEM_ILL2*fu_mem/MILL2*
203	VillousILL3.Villous_ILL3 -> MILL3.MEM_ILL3
	*DIFF_BASO_ill3*NI_Villous*switch_SFdiffbaso*VillousILL3.Villous_ILL3*fu_blood/VillousILL3*

	Reactions
204	MILL3.MEM_ILL3 -> VillousILL3.Villous_ILL3
	*DIFF_BASO_ill3*NI_Membrane*switch_SFdiffbaso*MILL3.MEM_ILL3*fu_mem/MILL3*
205	MILL3.MEM_ILL3 -> VILL3.X_ILL3_DISS
	*DIFF_ill3*NI_Membrane*switch_SFdiffapi*MILL3.MEM_ILL3*fu_mem/MILL3*
206	MILL4.MEM_ILL4 -> VILL4.X_ILL4_DISS
	*DIFF_ill4*NI_Membrane*switch_SFdiffapi*MILL4.MEM_ILL4*fu_mem/MILL4*
207	VillousILL4.Villous_ILL4 -> MILL4.MEM_ILL4
	*DIFF_BASO_ill4*NI_Villous*switch_SFdiffbaso*VillousILL4.Villous_ILL4*fu_blood/VillousILL4*
208	MILL4.MEM_ILL4 -> VillousILL4.Villous_ILL4
	*DIFF_BASO_ill4*NI_Membrane*switch_SFdiffbaso*MILL4.MEM_ILL4*fu_mem/MILL4*
209	Liver.Liver_drug -> Main_compartment.Bile_drug
	*k_liver_bile*Liver.Liver_drug*
210	Liver.Liver_drug -> Metabolites_Liver.Metabolites_liver_drug
	*k_liver_metabolites*Liver.Liver_drug*
211	Artery.Artery_drug -> Serosa.Serosa_drug
	*k_artery_serosa*Artery.Artery_drug*
212	Serosa.Serosa_drug -> Liver.Liver_drug
	*k_serosa_liver*Serosa.Serosa_drug*
213	VillousDUO.Villous_DUO -> MDUO.MEM_DUO
	*CLINT_influx_baso_DUO*influx_factor_duo_baso*switch_SFinflux_baso*Villous_DUO/VillousDUO*
214	VillousJEJ1.Villous_JEJ1 -> MJEJ1.MEM_JEJ1
	*CLINT_influx_baso_JEJ1*influx_factor_jej1_baso*switch_SFinflux_baso*Villous_JEJ1/VillousJEJ1*
215	VillousJEJ2.Villous_JEJ2 -> MJEJ2.MEM_JEJ2
	*CLINT_influx_baso_JEJ2*influx_factor_jej2_baso*switch_SFinflux_baso*Villous_JEJ2/VillousJEJ2*
216	VillousILL1.Villous_ILL1 -> MILL1.MEM_ILL1
	*CLINT_influx_baso_ILL1*influx_factor_ill1_baso*switch_SFinflux_baso*Villous_ILL1/VillousILL1*
217	VillousILL2.Villous_ILL2 -> MILL2.MEM_ILL2
	*CLINT_influx_baso_ILL2*influx_factor_ill2_baso*switch_SFinflux_baso*Villous_ILL2/VillousILL2*
218	VillousILL3.Villous_ILL3 -> MILL3.MEM_ILL3
	*CLINT_influx_baso_ILL3*influx_factor_ill3_baso*switch_SFinflux_baso*Villous_ILL3/VillousILL3*
219	VillousILL4.Villous_ILL4 -> MILL4.MEM_ILL4
	*CLINT_influx_baso_ILL4*influx_factor_ill4_baso*switch_SFinflux_baso*Villous_ILL4/VillousILL4*
220	MDUO.MEM_DUO -> VillousDUO.Villous_DUO
	*((switchVmax_efflux_baso==zero)*CLINT_efflux_baso_DUO*switch_SFefflux_baso*baso_efflux_factor_duo+switchVmax_efflux_baso*baso_efflux_factor_duo*ESA_baso*basoSurfaceRatio_DUO*switch_SFefflux_baso/(drug_Km_efflux_baso+MEM_DUO*fu_mem/MDUO/drug_molar_mass))*MDUO.MEM_DUO*fu_mem/MDUO*
221	MJEJ1.MEM_JEJ1 -> VillousJEJ1.Villous_JEJ1
	*((switchVmax_efflux_baso==zero)*CLINT_efflux_baso_JEJ1*switch_SFefflux_baso*baso_efflux_factor_jej1+switchVmax_efflux_baso*baso_efflux_factor_jej1*ESA_baso*basoSurfaceRatio_JEJ1*switch_SFefflux_baso/(drug_Km_efflux_baso+MEM_JEJ1*fu_mem/MJEJ1/drug_molar_mass))*MJEJ1.MEM_JEJ1*fu_mem/MJEJ1*
222	MJEJ2.MEM_JEJ2 -> VillousJEJ2.Villous_JEJ2

	Reactions
	*((switchVmax_efflux_baso==zero)*CLINT_efflux_baso_JEJ2*switch_SFefflux_baso*baso_efflux_factor_jej2+switchVmax_efflux_baso*baso_efflux_factor_jej2*ESA_baso*basoSurfaceRatio_JEJ2*switch_SFefflux_baso/(drug_Km_efflux_baso+MEM_JEJ2*fu_mem/MJEJ2/drug_molar_mass))*MJEJ2.MEM_JEJ2*fu_mem/MJEJ2*
223	MILL1.MEM_ILL1 -> VillousILL1.Villous_ILL1
	*((switchVmax_efflux_baso==zero)*CLINT_efflux_baso_ILL1*switch_SFefflux_baso*baso_efflux_factor_ill1+switchVmax_efflux_baso*baso_efflux_factor_ill1*ESA_baso*basoSurfaceRatio_ILL1*switch_SFefflux_baso/(drug_Km_efflux_baso+MEM_ILL1*fu_mem/MILL1/drug_molar_mass))*MILL1.MEM_ILL1*fu_mem/MILL1*
224	MILL2.MEM_ILL2 -> VillousILL2.Villous_ILL2
	*((switchVmax_efflux_baso==zero)*CLINT_efflux_baso_ILL2*switch_SFefflux_baso*baso_efflux_factor_ill2+switchVmax_efflux_baso*baso_efflux_factor_ill2*ESA_baso*basoSurfaceRatio_ILL2*switch_SFefflux_baso/(drug_Km_efflux_baso+MEM_ILL2*fu_mem/MILL2/drug_molar_mass))*MILL2.MEM_ILL2*fu_mem/MILL2*
225	MILL3.MEM_ILL3 -> VillousILL3.Villous_ILL3
	*((switchVmax_efflux_baso==zero)*CLINT_efflux_baso_ILL3*switch_SFefflux_baso*baso_efflux_factor_ill3+switchVmax_efflux_baso*baso_efflux_factor_ill3*ESA_baso*basoSurfaceRatio_ILL3*switch_SFefflux_baso/(drug_Km_efflux_baso+MEM_ILL3*fu_mem/MILL3/drug_molar_mass))*MILL3.MEM_ILL3*fu_mem/MILL3*
226	MILL4.MEM_ILL4 -> VillousILL4.Villous_ILL4
	*((switchVmax_efflux_baso==zero)*CLINT_efflux_baso_ILL4*switch_SFefflux_baso*baso_efflux_factor_ill4+switchVmax_efflux_baso*baso_efflux_factor_ill4*ESA_baso*basoSurfaceRatio_ILL4*switch_SFefflux_baso/(drug_Km_efflux_baso+MEM_ILL4*fu_mem/MILL4/drug_molar_mass))*MILL4.MEM_ILL4*fu_mem/MILL4*

Observables

	Observables	Units
1	AUCPlasma_1 = trapz(time,Plasma_total_1.Plasma_total_uM_1)	micromole/liter*hour
2	CendLiver_1 = Liver_total_1.Liver_tissue_total_uM_1(end)	micromole/liter
3	CmaxLiver_1 = max(Liver_total_1.Liver_tissue_total_uM_1)	micromole/liter
4	AUCLiver_1 = trapz(time, Liver_total_1.Liver_tissue_total_uM_1)	micromole/liter*hour
5	AUCLiver0to24_1 = trapz(time(time<24), Liver_total_1.Liver_tissue_total_uM_1(time<24))	micromole/liter*hour
6	AUCLiver72to96_1 = trapz(time(time>72&time<96), Liver_total_1.Liver_tissue_total_uM_1(time>72&time<96))	micromole/liter*hour
7	finalLiverTissuetotal = Liver_total.Liver_tissue_total_uM(end)	micromole/liter
8	maxLiverTissueTotal = max(Liver_total.Liver_tissue_total_uM)	micromole/liter
9	AUCLiver = trapz(time, Liver_total.Liver_tissue_total_uM)	micromole/liter*hour
10	AUCLiver0to24 = trapz(time(time<24), Liver_total.Liver_tissue_total_uM(time<24))	micromole/liter*hour
11	AUCLiver72to96 = trapz(time(time>72&time<96), Liver_total.Liver_tissue_total_uM(time>72&time<96))	micromole/liter*hour
12	AUCPlasma0to24 = trapz(time(time<24), Plasma_total.Plasma_total_uM(time<24))	micromole/liter*hour
13	AUCPlasma0to24_1 = trapz(time(time<24), Plasma_total_1.Plasma_total_uM_1(time<24))	micromole/liter*hour
14	AUCPlasma = trapz(time,Plasma_total.Plasma_total_uM)	micromole/liter*hour
15	CL_1 = max(drug_dose_amount_IV_1/trapz(time,Plasma_total_1.Plasma_total_drug_1))	liter/hour

	Observables	Units
16	Vss_1 = max(drug_dose_amount_IV_1*trapz(time,time.*Plasma_total_1.Plasma_total_drug_1)/trapz(time,Plasma_total_1.Plasma_total_drug_1)^2)	liter
17	AUCPlasmaTotal0to24 = trapz(time(time<24), Plasma_total.Plasma_total_drug(time<24))	nanogram/milliliter*hour
18	AUCPlasmaTotal = trapz(time, Plasma_total.Plasma_total_drug)	nanogram/milliliter*hour
19	ConcDUO = X_DUO_DISS./VDUO	microgram/liter
20	ConcJEJ1 = X_JEJ1_DISS./VJEJ1	microgram/liter
21	ConcJEJ2 = X_JEJ2_DISS./VJEJ2	microgram/liter
22	ConcILL1 = X_ILL1_DISS./VILL1	microgram/liter
23	ConcILL2 = X_ILL2_DISS./VILL2	microgram/liter
24	ConcILL3 = X_ILL3_DISS./VILL3	microgram/liter
25	ConcILL4 = X_ILL4_DISS./VILL4	microgram/liter
26	counterObs = counterObservable(Plasma_total.Plasma_total_drug)	dimensionless

Model Equations

ODEs

	ODEs
1	d(Bile_drug)/dt = -(k_transit*Bile_drug) - (drug_k_bile_deg*Bile_drug) + (k_liver_bile*Liver_drug)
2	d(Venous_drug)/dt = 1/Venous*((k_Liver_Venous*Liver_drug) + (k_rest_venous*Rest_drug) + (k_bone_venous*Bone_drug) + (k_skin_venous*Skin_drug) + (k_heart_venous*Heart_drug) + (k_adipos_venous*Adipose_drug) + (k_muscle_venous*Muscle_drug) + (k_brain_venous*Brain_drug) + (k_kidney_venous*Kidney_drug) - (k_venous_lung*Venous_drug) - (k_venous_urine_CLR*Venous_drug) + (k_testes_venous*Testes_drug) - (k_venous_urine_GFR*Venous_drug))
3	d(Lung_drug)/dt = 1/Lung*((k_venous_lung*Venous_drug) - (k_lung_artery*Lung_drug))
4	d(Kidney_drug)/dt = 1/Kidney*(-(k_kidney_venous*Kidney_drug) + (k_artery_kidney*Artery_drug))
5	d(Brain_drug)/dt = 1/Brain*(-(k_brain_venous*Brain_drug) + (k_artery_brain*Artery_drug))
6	d(Muscle_drug)/dt = 1/Muscle*(-(k_muscle_venous*Muscle_drug) + (k_artery_muscle*Artery_drug))
7	d(Adipose_drug)/dt = 1/Adipose*(-(k_adipos_venous*Adipose_drug) + (k_artery_adipos*Artery_drug))
8	d(Heart_drug)/dt = 1/Heart*(-(k_heart_venous*Heart_drug) + (k_artery_heart*Artery_drug))
9	d(Skin_drug)/dt = 1/Skin*(-(k_skin_venous*Skin_drug) + (k_artery_skin*Artery_drug))
10	d(Bone_drug)/dt = 1/Bone*(-(k_bone_venous*Bone_drug) + (k_artery_bone*Artery_drug))
11	d(Rest_drug)/dt = 1/Rest*(-(k_rest_venous*Rest_drug) + (k_artery_rest*Artery_drug))
12	d(Artery_drug)/dt = 1/Artery*(-(k_artery_liver*Artery_drug) - (Qmuc_ILL1*Artery_drug) - (k_artery_spleen*Artery_drug) - (k_artery_rest*Artery_drug) - (k_artery_bone*Artery_drug) - (k_artery_skin*Artery_drug) - (k_artery_heart*Artery_drug) - (k_artery_adipos*Artery_drug) - (k_artery_muscle*Artery_drug) - (k_artery_brain*Artery_drug) - (k_artery_kidney*Artery_drug) + (k_lung_artery*Lung_drug) - (k_artery_testes*Artery_drug) - (Qmuc_DUO*Artery_drug) - (Qmuc_JEJ1*Artery_drug) - (Qmuc_JEJ2*Artery_drug) - (Qmuc_ILL2*Artery_drug) - (Qmuc_ILL3*Artery_drug) - (Qmuc_ILL4*Artery_drug) - (k_artery_serosa*Artery_drug))
13	d(Spleen_drug)/dt = 1/Spleen*((k_artery_spleen*Artery_drug) - (k_spleen_liver*Spleen_drug))
14	d(Liver_IC_S5_drug)/dt = 1/Liver_IC_S5*(-(k_Liver_IC_S5_Bile*Liver_IC_S5_drug))
15	d(Testes_drug)/dt = 1/Testes*((k_artery_testes*Artery_drug) - (k_testes_venous*Testes_drug))
16	d(Urine_drug)/dt = (k_venous_urine_CLR*Venous_drug) + (k_venous_urine_GFR*Venous_drug)

	**ODEs**
17	d(AMT_ELIM_DUO)/dt = -(((switchVmax_efflux==zero)*CLINT_efflux_DUO*efflux_factor_duo*switch_SFefflux+switchVmax_efflux*ESA*surfaceRatio_DUO*efflux_factor_duo*switch_SFefflux/(drug_Km_efflux+MEM_DUO*fu_mem/MDUO/drug_molar_mass))*MEM_DUO*fu_mem/MDUO) + (((switchVmax_influx==zero)*CLINT_influx_DUO*influx_factor_duo*switch_SFinflux+switchVmax_influx*ESA*surfaceRatio_DUO*influx_factor_duo*switch_SFinflux/(drug_Km_influx+X_DUO_DISS/VDUO/drug_molar_mass))*X_DUO_DISS/VDUO) + ((CLINT_metabolism*metabolism_factor_duo*switch_SFgutmet*MEM_DUO*fu_mem)/MDUO)
18	d(AMT_ELIM_JEJ1)/dt = -(((switchVmax_efflux==zero)*CLINT_efflux_JEJ1*efflux_factor_jej1*switch_SFefflux+switchVmax_efflux*ESA*surfaceRatio_JEJ1*efflux_factor_jej1*switch_SFefflux/(drug_Km_efflux+MEM_JEJ1*fu_mem/MJEJ1/drug_molar_mass))*MEM_JEJ1*fu_mem/MJEJ1) + (((switchVmax_influx==zero)*CLINT_influx_JEJ1*influx_factor_jej1*switch_SFinflux+switchVmax_influx*ESA*surfaceRatio_JEJ1*influx_factor_jej1*switch_SFinflux/(drug_Km_influx+X_JEJ1_DISS/VJEJ1/drug_molar_mass))*X_JEJ1_DISS/VJEJ1) + ((CLINT_metabolism*metabolism_factor_jej1*switch_SFgutmet*MEM_JEJ1*fu_mem)/MJEJ1)
19	d(AMT_ELIM_JEJ2)/dt = -(((switchVmax_efflux==zero)*CLINT_efflux_JEJ2*efflux_factor_jej2*switch_SFefflux+switchVmax_efflux*ESA*surfaceRatio_JEJ2*efflux_factor_jej2*switch_SFefflux/(drug_Km_efflux+MEM_JEJ2*fu_mem/MJEJ2/drug_molar_mass))*MEM_JEJ2*fu_mem/MJEJ2) + (((switchVmax_influx==zero)*CLINT_influx_JEJ2*influx_factor_jej2*switch_SFinflux+switchVmax_influx*ESA*surfaceRatio_JEJ2*influx_factor_jej2*switch_SFinflux/(drug_Km_influx+X_JEJ2_DISS/VJEJ2/drug_molar_mass))*X_JEJ2_DISS/VJEJ2) + ((CLINT_metabolism*metabolism_factor_jej2*switch_SFgutmet*MEM_JEJ2*fu_mem)/MJEJ2)
20	d(AMT_ELIM_ILL1)/dt = -(((switchVmax_efflux==zero)*CLINT_efflux_ILL1*efflux_factor_ill1*switch_SFefflux_Lower+switchVmax_efflux*ESA*surfaceRatio_ILL1*efflux_factor_ill1*switch_SFefflux_Lower/(drug_Km_efflux+MEM_ILL1*fu_mem/MILL1/drug_molar_mass))*MEM_ILL1*fu_mem/MILL1) + (((switchVmax_influx==zero)*CLINT_influx_ILL1*influx_factor_ill1*switch_SFinflux_Lower+switchVmax_influx*ESA*surfaceRatio_ILL1*influx_factor_ill1*switch_SFinflux_Lower/(drug_Km_influx+X_ILL1_DISS/VILL1/drug_molar_mass))*X_ILL1_DISS/VILL1) + ((CLINT_metabolism*metabolism_factor_ill1*switch_SFgutmet*MEM_ILL1*fu_mem)/MILL1)
21	d(AMT_ELIM_ILL2)/dt = -(((switchVmax_efflux==zero)*CLINT_efflux_ILL2*efflux_factor_ill2*switch_SFefflux_Lower+switchVmax_efflux*ESA*surfaceRatio_ILL2*efflux_factor_ill2*switch_SFefflux_Lower/(drug_Km_efflux+MEM_ILL2*fu_mem/MILL2/drug_molar_mass))*MEM_ILL2*fu_mem/MILL2) + (((switchVmax_influx==zero)*CLINT_influx_ILL2*influx_factor_ill2*switch_SFinflux_Lower+switchVmax_influx*ESA*surfaceRatio_ILL2*influx_factor_ill2*switch_SFinflux_Lower/(drug_Km_influx+X_ILL2_DISS/VILL2/drug_molar_mass))*X_ILL2_DISS/VILL2) + ((CLINT_metabolism*metabolism_factor_ill2*switch_SFgutmet*MEM_ILL2*fu_mem)/MILL2)
22	d(AMT_ELIM_ILL3)/dt = -(((switchVmax_efflux==zero)*CLINT_efflux_ILL3*efflux_factor_ill3*switch_SFefflux_Lower+switchVmax_efflux*ESA*surfaceRatio_ILL3*efflux_factor_ill3*switch_SFefflux_Lower/(drug_Km_efflux+MEM_ILL3*fu_mem/MILL3/drug_molar_mass))*MEM_ILL3*fu_mem/MILL3) + (((switchVmax_influx==zero)*CLINT_influx_ILL3*influx_factor_ill3*switch_SFinflux_Lower+switchVmax_influx*ESA*surfaceRatio_ILL3*influx_factor_ill3*switch_SFinflux_Lower/(drug_Km_influx+X_ILL3_DISS/VILL3/drug_molar_mass))*X_ILL3_DISS/VILL3) + ((CLINT_metabolism*metabolism_factor_ill3*switch_SFgutmet*MEM_ILL3*fu_mem)/MILL3)
23	d(AMT_ELIM_ILL4)/dt = -(((switchVmax_efflux==zero)*CLINT_efflux_ILL4*efflux_factor_ill4*switch_SFefflux_Lower+switchVmax_efflux*ESA*surfaceRatio_ILL4*efflux_factor_ill4*switch_SFefflux_Lower/(drug_Km_efflux+MEM_ILL4*fu_mem/MILL4/drug_molar_mass))*MEM_ILL4*fu_mem/MILL4) + (((switchVmax_influx==zero)*CLINT_influx_ILL4*influx_factor_ill4*switch_SFinflux_Lower+switchVmax_influx*ESA*surfaceRatio_ILL4*influx_factor_ill4*switch_SFinflux_Lower/(drug_Km_influx+X_ILL4_DISS/VILL4/drug_molar_mass))*X_ILL4_DISS/VILL4) + ((CLINT_metabolism*metabolism_factor_ill4*switch_SFgutmet*MEM_ILL4*fu_mem)/MILL4)
24	d(AMT_ELIM_gut)/dt = ((CLINT_metabolism*metabolism_factor_duo*switch_SFgutmet*MEM_DUO*fu_mem)/MDUO) + ((CLINT_metabolism*metabolism_factor_jej1*switch_SFgutmet*MEM_JEJ1*fu_mem)/MJEJ1) + ((CLINT_metabolism*metabolism_factor_jej2*switch_SFgutmet*MEM_JEJ2*fu_mem)/MJEJ2) + ((CLINT_metabolism*metabolism_factor_ill1*switch_SFgutmet*MEM_ILL1*fu_mem)/MILL1) + ((CLINT_metabolism*metabolism_factor_ill2*switch_SFgutmet*MEM_ILL2*fu_mem)/MILL2) + ((CLINT_metabolism*metabolism_factor_ill3*switch_SFgutmet*MEM_ILL3*fu_mem)/MILL3) + ((CLINT_metabolism*metabolism_factor_ill4*switch_SFgutmet*MEM_ILL4*fu_mem)/MILL4)
25	d(X_STOMACH_SOLID)/dt = -(X_STOMACH_SOLID/TSTOMACH) - (KD*X_STOMACH_SOLID*(SOLIF_STOMACH-X_STOMACH_DISS/STOMACH))

	ODEs
26	d(X_STOMACH_DISS)/dt = -(X_STOMACH_DISS/TSTOMACH) + (KD*X_STOMACH_SOLID*(SOLIF_STOMACH-X_STOMACH_DISS/STOMACH))
27	d(X_DUO_SOLID)/dt = (X_STOMACH_SOLID/TSTOMACH) - (X_DUO_SOLID/TDUO) - (KD*X_DUO_SOLID*(SOLIF_DUO-X_DUO_DISS/VDUO))
28	d(X_DUO_DISS)/dt = (k_transit*Bile_drug) + (X_STOMACH_DISS/TSTOMACH) + (KD*X_DUO_SOLID*(SOLIF_DUO-X_DUO_DISS/VDUO)) - (X_DUO_DISS/TDUO) - ((DIFF_duo*NI_DUO*switch_SFdiffapi*X_DUO_DISS)/VDUO) + (((switchVmax_efflux==zero)*CLINT_efflux_DUO*efflux_factor_duo*switch_SFefflux+switchVmax_efflux*ESA*surfaceRatio_DUO*efflux_factor_duo*switch_SFefflux/(drug_Km_efflux+MEM_DUO*fu_mem/MDUO/drug_molar_mass))*MEM_DUO*fu_mem/MDUO) - (((switchVmax_influx==zero)*CLINT_influx_DUO*influx_factor_duo*switch_SFinflux+switchVmax_influx*ESA*surfaceRatio_DUO*influx_factor_duo*switch_SFinflux/(drug_Km_influx+X_DUO_DISS/VDUO/drug_molar_mass))*X_DUO_DISS/VDUO) + (DIFF_duo*NI_Membrane*switch_SFdiffapi*MEM_DUO*fu_mem/MDUO)
29	d(X_JEJ1_SOLID)/dt = (X_DUO_SOLID/TDUO) - (X_JEJ1_SOLID/TJEJ1) - (KD*X_JEJ1_SOLID*(SOLIF_JEJ1-X_JEJ1_DISS/VJEJ1))
30	d(X_JEJ1_DISS)/dt = (X_DUO_DISS/TDUO) + (KD*X_JEJ1_SOLID*(SOLIF_JEJ1-X_JEJ1_DISS/VJEJ1)) - (X_JEJ1_DISS/TJEJ1) - ((DIFF_jej1*NI_JEJ1*switch_SFdiffapi*X_JEJ1_DISS)/VJEJ1) + (((switchVmax_efflux==zero)*CLINT_efflux_JEJ1*efflux_factor_jej1*switch_SFefflux+switchVmax_efflux*ESA*surfaceRatio_JEJ1*efflux_factor_jej1*switch_SFefflux/(drug_Km_efflux+MEM_JEJ1*fu_mem/MJEJ1/drug_molar_mass))*MEM_JEJ1*fu_mem/MJEJ1) - (((switchVmax_influx==zero)*CLINT_influx_JEJ1*influx_factor_jej1*switch_SFinflux+switchVmax_influx*ESA*surfaceRatio_JEJ1*influx_factor_jej1*switch_SFinflux/(drug_Km_influx+X_JEJ1_DISS/VJEJ1/drug_molar_mass))*X_JEJ1_DISS/VJEJ1) + (DIFF_jej1*NI_Membrane*switch_SFdiffapi*MEM_JEJ1*fu_mem/MJEJ1)
31	d(MEM_DUO)/dt = ((DIFF_duo*NI_DUO*switch_SFdiffapi*X_DUO_DISS)/VDUO) - (((switchVmax_efflux==zero)*CLINT_efflux_DUO*efflux_factor_duo*switch_SFefflux+switchVmax_efflux*ESA*surfaceRatio_DUO*efflux_factor_duo*switch_SFefflux/(drug_Km_efflux+MEM_DUO*fu_mem/MDUO/drug_molar_mass))*MEM_DUO*fu_mem/MDUO) + (((switchVmax_influx==zero)*CLINT_influx_DUO*influx_factor_duo*switch_SFinflux+switchVmax_influx*ESA*surfaceRatio_DUO*influx_factor_duo*switch_SFinflux/(drug_Km_influx+X_DUO_DISS/VDUO/drug_molar_mass))*X_DUO_DISS/VDUO) - ((CLINT_metabolism*metabolism_factor_duo*switch_SFgutmet*MEM_DUO*fu_mem)/MDUO) - (DIFF_BASO_duo*NI_Membrane*switch_SFdiffbaso*MEM_DUO*fu_mem/MDUO) + (DIFF_BASO_duo*NI_Villous*switch_SFdiffbaso*Villous_DUO*fu_blood/VillousDUO) - (DIFF_duo*NI_Membrane*switch_SFdiffapi*MEM_DUO*fu_mem/MDUO) + (CLINT_influx_baso_DUO*influx_factor_duo_baso*switch_SFinflux_baso*Villous_DUO/VillousDUO) - (((switchVmax_efflux_baso==zero)*CLINT_efflux_baso_DUO*switch_SFefflux_baso*baso_efflux_factor_duo+switchVmax_efflux_baso*baso_efflux_factor_duo*ESA_baso*basoSurfaceRatio_DUO*switch_SFefflux_baso/(drug_Km_efflux_baso+MEM_DUO*fu_mem/MDUO/drug_molar_mass))*MEM_DUO*fu_mem/MDUO)
32	d(MEM_JEJ1)/dt = ((DIFF_jej1*NI_JEJ1*switch_SFdiffapi*X_JEJ1_DISS)/VJEJ1) - (((switchVmax_efflux==zero)*CLINT_efflux_JEJ1*efflux_factor_jej1*switch_SFefflux+switchVmax_efflux*ESA*surfaceRatio_JEJ1*efflux_factor_jej1*switch_SFefflux/(drug_Km_efflux+MEM_JEJ1*fu_mem/MJEJ1/drug_molar_mass))*MEM_JEJ1*fu_mem/MJEJ1) + (((switchVmax_influx==zero)*CLINT_influx_JEJ1*influx_factor_jej1*switch_SFinflux+switchVmax_influx*ESA*surfaceRatio_JEJ1*influx_factor_jej1*switch_SFinflux/(drug_Km_influx+X_JEJ1_DISS/VJEJ1/drug_molar_mass))*X_JEJ1_DISS/VJEJ1) - ((CLINT_metabolism*metabolism_factor_jej1*switch_SFgutmet*MEM_JEJ1*fu_mem)/MJEJ1) - (DIFF_BASO_jej1*NI_Membrane*switch_SFdiffbaso*MEM_JEJ1*fu_mem/MJEJ1) + (DIFF_BASO_jej1*NI_Villous*switch_SFdiffbaso*Villous_JEJ1*fu_blood/VillousJEJ1) - (DIFF_jej1*NI_Membrane*switch_SFdiffapi*MEM_JEJ1*fu_mem/MJEJ1) + (CLINT_influx_baso_JEJ1*influx_factor_jej1_baso*switch_SFinflux_baso*Villous_JEJ1/VillousJEJ1) - (((switchVmax_efflux_baso==zero)*CLINT_efflux_baso_JEJ1*switch_SFefflux_baso*baso_efflux_factor_jej1+switchVmax_efflux_baso*baso_efflux_factor_jej1*ESA_baso*basoSurfaceRatio_JEJ1*switch_SFefflux_baso/(drug_Km_efflux_baso+MEM_JEJ1*fu_mem/MJEJ1/drug_molar_mass))*MEM_JEJ1*fu_mem/MJEJ1)
33	d(X_JEJ2_SOLID)/dt = (X_JEJ1_SOLID/TJEJ1) - (X_JEJ2_SOLID/TJEJ2) - (KD*X_JEJ2_SOLID*(SOLIF_JEJ2-X_JEJ2_DISS/VJEJ2))
34	d(X_JEJ2_DISS)/dt = (X_JEJ1_DISS/TJEJ1) + (KD*X_JEJ2_SOLID*(SOLIF_JEJ2-X_JEJ2_DISS/VJEJ2)) - (X_JEJ2_DISS/TJEJ2) - ((DIFF_jej2*NI_JEJ2*switch_SFdiffapi*X_JEJ2_DISS)/VJEJ2) + (((switchVmax_efflux==zero)*CLINT_efflux_JEJ2*efflux_factor_jej2*switch_SFefflux+switchVmax_efflux*ESA*surfaceRatio_JEJ2*efflux_factor_jej2*switch_SFefflux/(drug_Km_efflux+MEM_JEJ2*fu_mem/MJEJ2/drug_molar_mass))*MEM_JEJ2*fu_mem/MJEJ2) - (((switchVmax_influx==zero)*CLINT_influx_JEJ2*influx_factor_jej2*switch_SFinflux+switchVmax_influx*ESA*surfaceRatio_JEJ2*influx_factor_jej2*switch_SFinflux/(drug_Km_influx+X_JEJ2_DISS/VJEJ2/drug_molar_mass))*X_JEJ2_DISS/VJEJ2) + (DIFF_jej2*NI_Membrane*switch_SFdiffapi*MEM_JEJ2*fu_mem/MJEJ2)

	ODEs
35	d(MEM_JEJ2)/dt = ((DIFF_jej2*NI_JEJ2*switch_SFdiffapi*X_JEJ2_DISS)/VJEJ2) - (((switchVmax_efflux==zero)*CLINT_efflux_JEJ2*efflux_factor_jej2*switch_SFefflux+switchVmax_efflux*ESA*surfaceRatio_JEJ2*efflux_factor_jej2*switch_SFefflux/(drug_Km_efflux+MEM_JEJ2*fu_mem/MJEJ2/drug_molar_mass))*MEM_JEJ2*fu_mem/MJEJ2) + (((switchVmax_influx==zero)*CLINT_influx_JEJ2*influx_factor_jej2*switch_SFinflux+switchVmax_influx*ESA*surfaceRatio_JEJ2*influx_factor_jej2*switch_SFinflux/(drug_Km_influx+X_JEJ2_DISS/VJEJ2/drug_molar_mass))*X_JEJ2_DISS/VJEJ2) - ((CLINT_metabolism*metabolism_factor_jej2*switch_SFgutmet*MEM_JEJ2*fu_mem)/MJEJ2) - (DIFF_BASO_jej2*NI_Membrane*switch_SFdiffbaso*MEM_JEJ2*fu_mem/MJEJ2) + (DIFF_BASO_jej2*NI_Villous*switch_SFdiffbaso*Villous_JEJ2*fu_blood/VillousJEJ2) - (DIFF_jej2*NI_Membrane*switch_SFdiffapi*MEM_JEJ2*fu_mem/MJEJ2) + (CLINT_influx_baso_JEJ2*influx_factor_jej2_baso*switch_SFinflux_baso*Villous_JEJ2/VillousJEJ2) - (((switchVmax_efflux_baso==zero)*CLINT_efflux_baso_JEJ2*switch_SFefflux_baso*baso_efflux_factor_jej2+switchVmax_efflux_baso*baso_efflux_factor_jej2*ESA_baso*basoSurfaceRatio_JEJ2*switch_SFefflux_baso/(drug_Km_efflux_baso+MEM_JEJ2*fu_mem/MJEJ2/drug_molar_mass))*MEM_JEJ2*fu_mem/MJEJ2)
36	d(X_ILL1_SOLID)/dt = (X_JEJ2_SOLID/TJEJ2) - (X_ILL1_SOLID/TILL1) - (KD*X_ILL1_SOLID*(SOLIF_ILL1-X_ILL1_DISS/VILL1))
37	d(X_ILL1_DISS)/dt = (X_JEJ2_DISS/TJEJ2) + (KD*X_ILL1_SOLID*(SOLIF_ILL1-X_ILL1_DISS/VILL1)) - (X_ILL1_DISS/TILL1) - ((DIFF_ill1*NI_ILL1*switch_SFdiffapi*X_ILL1_DISS)/VILL1) + (((switchVmax_efflux==zero)*CLINT_efflux_ILL1*efflux_factor_ill1*switch_SFefflux_Lower+switchVmax_efflux*ESA*surfaceRatio_ILL1*efflux_factor_ill1*switch_SFefflux_Lower/(drug_Km_efflux+MEM_ILL1*fu_mem/MILL1/drug_molar_mass))*MEM_ILL1*fu_mem/MILL1) - (((switchVmax_influx==zero)*CLINT_influx_ILL1*influx_factor_ill1*switch_SFinflux_Lower+switchVmax_influx*ESA*surfaceRatio_ILL1*influx_factor_ill1*switch_SFinflux_Lower/(drug_Km_influx+X_ILL1_DISS/VILL1/drug_molar_mass))*X_ILL1_DISS/VILL1) + (DIFF_ill1*NI_Membrane*switch_SFdiffapi*MEM_ILL1*fu_mem/MILL1)
38	d(MEM_ILL1)/dt = ((DIFF_ill1*NI_ILL1*switch_SFdiffapi*X_ILL1_DISS)/VILL1) - (((switchVmax_efflux==zero)*CLINT_efflux_ILL1*efflux_factor_ill1*switch_SFefflux_Lower+switchVmax_efflux*ESA*surfaceRatio_ILL1*efflux_factor_ill1*switch_SFefflux_Lower/(drug_Km_efflux+MEM_ILL1*fu_mem/MILL1/drug_molar_mass))*MEM_ILL1*fu_mem/MILL1) + (((switchVmax_influx==zero)*CLINT_influx_ILL1*influx_factor_ill1*switch_SFinflux_Lower+switchVmax_influx*ESA*surfaceRatio_ILL1*influx_factor_ill1*switch_SFinflux_Lower/(drug_Km_influx+X_ILL1_DISS/VILL1/drug_molar_mass))*X_ILL1_DISS/VILL1) - ((CLINT_metabolism*metabolism_factor_ill1*switch_SFgutmet*MEM_ILL1*fu_mem)/MILL1) - (DIFF_BASO_ill1*NI_Membrane*switch_SFdiffbaso*MEM_ILL1*fu_mem/MILL1) + (DIFF_BASO_ill1*NI_Villous*switch_SFdiffbaso*Villous_ILL1*fu_blood/VillousILL1) - (DIFF_ill1*NI_Membrane*switch_SFdiffapi*MEM_ILL1*fu_mem/MILL1) + (CLINT_influx_baso_ILL1*influx_factor_ill1_baso*switch_SFinflux_baso*Villous_ILL1/VillousILL1) - (((switchVmax_efflux_baso==zero)*CLINT_efflux_baso_ILL1*switch_SFefflux_baso*baso_efflux_factor_ill1+switchVmax_efflux_baso*baso_efflux_factor_ill1*ESA_baso*basoSurfaceRatio_ILL1*switch_SFefflux_baso/(drug_Km_efflux_baso+MEM_ILL1*fu_mem/MILL1/drug_molar_mass))*MEM_ILL1*fu_mem/MILL1)
39	d(X_ILL2_SOLID)/dt = (X_ILL1_SOLID/TILL1) - (X_ILL2_SOLID/TILL2) - (KD*X_ILL2_SOLID*(SOLIF_ILL2-X_ILL2_DISS/VILL2))
40	d(X_ILL2_DISS)/dt = (X_ILL1_DISS/TILL1) + (KD*X_ILL2_SOLID*(SOLIF_ILL2-X_ILL2_DISS/VILL2)) - (X_ILL2_DISS/TILL2) - ((DIFF_ill2*NI_ILL2*switch_SFdiffapi*X_ILL2_DISS)/VILL2) + (((switchVmax_efflux==zero)*CLINT_efflux_ILL2*efflux_factor_ill2*switch_SFefflux_Lower+switchVmax_efflux*ESA*surfaceRatio_ILL2*efflux_factor_ill2*switch_SFefflux_Lower/(drug_Km_efflux+MEM_ILL2*fu_mem/MILL2/drug_molar_mass))*MEM_ILL2*fu_mem/MILL2) - (((switchVmax_influx==zero)*CLINT_influx_ILL2*influx_factor_ill2*switch_SFinflux_Lower+switchVmax_influx*ESA*surfaceRatio_ILL2*influx_factor_ill2*switch_SFinflux_Lower/(drug_Km_influx+X_ILL2_DISS/VILL2/drug_molar_mass))*X_ILL2_DISS/VILL2) + (DIFF_ill2*NI_Membrane*switch_SFdiffapi*MEM_ILL2*fu_mem/MILL2)
41	d(MEM_ILL2)/dt = ((DIFF_ill2*NI_ILL2*switch_SFdiffapi*X_ILL2_DISS)/VILL2) - (((switchVmax_efflux==zero)*CLINT_efflux_ILL2*efflux_factor_ill2*switch_SFefflux_Lower+switchVmax_efflux*ESA*surfaceRatio_ILL2*efflux_factor_ill2*switch_SFefflux_Lower/(drug_Km_efflux+MEM_ILL2*fu_mem/MILL2/drug_molar_mass))*MEM_ILL2*fu_mem/MILL2) + (((switchVmax_influx==zero)*CLINT_influx_ILL2*influx_factor_ill2*switch_SFinflux_Lower+switchVmax_influx*ESA*surfaceRatio_ILL2*influx_factor_ill2*switch_SFinflux_Lower/(drug_Km_influx+X_ILL2_DISS/VILL2/drug_molar_mass))*X_ILL2_DISS/VILL2) - ((CLINT_metabolism*metabolism_factor_ill2*switch_SFgutmet*MEM_ILL2*fu_mem)/MILL2) + (DIFF_BASO_ill2*NI_Villous*switch_SFdiffbaso*Villous_ILL2*fu_blood/VillousILL2) - (DIFF_BASO_ill2*NI_Membrane*switch_SFdiffbaso*MEM_ILL2*fu_mem/MILL2) - (DIFF_ill2*NI_Membrane*switch_SFdiffapi*MEM_ILL2*fu_mem/MILL2) + (CLINT_influx_baso_ILL2*influx_factor_ill2_baso*switch_SFinflux_baso*Villous_ILL2/VillousILL2) - (((switchVmax_efflux_baso==zero)*CLINT_efflux_baso_ILL2*switch_SFefflux_baso*baso_efflux_factor_ill2+switchVmax_

	ODEs
	efflux_baso*baso_efflux_factor_ill2*ESA_baso*basoSurfaceRatio_ILL2*switch_SFefflux_baso/(drug_Km_efflux_baso+MEM_ILL2*fu_mem/MILL2/drug_molar_mass))*MEM_ILL2*fu_mem/MILL2)
42	d(X_ILL3_SOLID)/dt = (X_ILL2_SOLID/TILL2) - (X_ILL3_SOLID/TILL3) - (KD*X_ILL3_SOLID*(SOLIF_ILL3-X_ILL3_DISS/VILL3))
43	d(X_ILL3_DISS)/dt = (X_ILL2_DISS/TILL2) + (KD*X_ILL3_SOLID*(SOLIF_ILL3-X_ILL3_DISS/VILL3)) - (X_ILL3_DISS/TILL3) - ((DIFF_ill3*NI_ILL3*switch_SFdiffapi*X_ILL3_DISS)/VILL3) + (((switchVmax_efflux==zero)*CLINT_efflux_ILL3*efflux_factor_ill3*switch_SFefflux_Lower+switchVmax_efflux*ESA*surfaceRatio_ILL3*efflux_factor_ill3*switch_SFefflux_Lower/(drug_Km_efflux+MEM_ILL3*fu_mem/MILL3/drug_molar_mass))*MEM_ILL3*fu_mem/MILL3) - (((switchVmax_influx==zero)*CLINT_influx_ILL3*influx_factor_ill3*switch_SFinflux_Lower+switchVmax_influx*ESA*surfaceRatio_ILL3*influx_factor_ill3*switch_SFinflux_Lower/(drug_Km_influx+X_ILL3_DISS/VILL3/drug_molar_mass))*X_ILL3_DISS/VILL3) + (DIFF_ill3*NI_Membrane*switch_SFdiffapi*MEM_ILL3*fu_mem/MILL3)
44	d(MEM_ILL3)/dt = ((DIFF_ill3*NI_ILL3*switch_SFdiffapi*X_ILL3_DISS)/VILL3) - (((switchVmax_efflux==zero)*CLINT_efflux_ILL3*efflux_factor_ill3*switch_SFefflux_Lower+switchVmax_efflux*ESA*surfaceRatio_ILL3*efflux_factor_ill3*switch_SFefflux_Lower/(drug_Km_efflux+MEM_ILL3*fu_mem/MILL3/drug_molar_mass))*MEM_ILL3*fu_mem/MILL3) + (((switchVmax_influx==zero)*CLINT_influx_ILL3*influx_factor_ill3*switch_SFinflux_Lower+switchVmax_influx*ESA*surfaceRatio_ILL3*influx_factor_ill3*switch_SFinflux_Lower/(drug_Km_influx+X_ILL3_DISS/VILL3/drug_molar_mass))*X_ILL3_DISS/VILL3) - ((CLINT_metabolism*metabolism_factor_ill3*switch_SFgutmet*MEM_ILL3*fu_mem)/MILL3) + (DIFF_BASO_ill3*NI_Villous*switch_SFdiffbaso*Villous_ILL3*fu_blood/VillousILL3) - (DIFF_BASO_ill3*NI_Membrane*switch_SFdiffbaso*MEM_ILL3*fu_mem/MILL3) - (DIFF_ill3*NI_Membrane*switch_SFdiffapi*MEM_ILL3*fu_mem/MILL3) + (CLINT_influx_baso_ILL3*influx_factor_ill3_baso*switch_SFinflux_baso*Villous_ILL3/VillousILL3) - (((switchVmax_efflux_baso==zero)*CLINT_efflux_baso_ILL3*switch_SFefflux_baso*baso_efflux_factor_ill3+switchVmax_efflux_baso*baso_efflux_factor_ill3*ESA_baso*basoSurfaceRatio_ILL3*switch_SFefflux_baso/(drug_Km_efflux_baso+MEM_ILL3*fu_mem/MILL3/drug_molar_mass))*MEM_ILL3*fu_mem/MILL3)
45	d(X_ILL4_SOLID)/dt = (X_ILL3_SOLID/TILL3) - (X_ILL4_SOLID/TILL4) - (KD*X_ILL4_SOLID*(SOLIF_ILL4-X_ILL4_DISS/VILL4))
46	d(X_ILL4_DISS)/dt = (X_ILL3_DISS/TILL3) + (KD*X_ILL4_SOLID*(SOLIF_ILL4-X_ILL4_DISS/VILL4)) - (X_ILL4_DISS/TILL4) - ((DIFF_ill4*NI_ILL4*switch_SFdiffapi*X_ILL4_DISS)/VILL4) + (((switchVmax_efflux==zero)*CLINT_efflux_ILL4*efflux_factor_ill4*switch_SFefflux_Lower+switchVmax_efflux*ESA*surfaceRatio_ILL4*efflux_factor_ill4*switch_SFefflux_Lower/(drug_Km_efflux+MEM_ILL4*fu_mem/MILL4/drug_molar_mass))*MEM_ILL4*fu_mem/MILL4) - (((switchVmax_influx==zero)*CLINT_influx_ILL4*influx_factor_ill4*switch_SFinflux_Lower+switchVmax_influx*ESA*surfaceRatio_ILL4*influx_factor_ill4*switch_SFinflux_Lower/(drug_Km_influx+X_ILL4_DISS/VILL4/drug_molar_mass))*X_ILL4_DISS/VILL4) + (DIFF_ill4*NI_Membrane*switch_SFdiffapi*MEM_ILL4*fu_mem/MILL4)
47	d(MEM_ILL4)/dt = ((DIFF_ill4*NI_ILL4*switch_SFdiffapi*X_ILL4_DISS)/VILL4) - (((switchVmax_efflux==zero)*CLINT_efflux_ILL4*efflux_factor_ill4*switch_SFefflux_Lower+switchVmax_efflux*ESA*surfaceRatio_ILL4*efflux_factor_ill4*switch_SFefflux_Lower/(drug_Km_efflux+MEM_ILL4*fu_mem/MILL4/drug_molar_mass))*MEM_ILL4*fu_mem/MILL4) + (((switchVmax_influx==zero)*CLINT_influx_ILL4*influx_factor_ill4*switch_SFinflux_Lower+switchVmax_influx*ESA*surfaceRatio_ILL4*influx_factor_ill4*switch_SFinflux_Lower/(drug_Km_influx+X_ILL4_DISS/VILL4/drug_molar_mass))*X_ILL4_DISS/VILL4) - ((CLINT_metabolism*metabolism_factor_ill4*switch_SFgutmet*MEM_ILL4*fu_mem)/MILL4) - (DIFF_ill4*NI_Membrane*switch_SFdiffapi*MEM_ILL4*fu_mem/MILL4) + (DIFF_BASO_ill4*NI_Villous*switch_SFdiffbaso*Villous_ILL4*fu_blood/VillousILL4) - (DIFF_BASO_ill4*NI_Membrane*switch_SFdiffbaso*MEM_ILL4*fu_mem/MILL4) + (CLINT_influx_baso_ILL4*influx_factor_ill4_baso*switch_SFinflux_baso*Villous_ILL4/VillousILL4) - (((switchVmax_efflux_baso==zero)*CLINT_efflux_baso_ILL4*switch_SFefflux_baso*baso_efflux_factor_ill4+switchVmax_efflux_baso*baso_efflux_factor_ill4*ESA_baso*basoSurfaceRatio_ILL4*switch_SFefflux_baso/(drug_Km_efflux_baso+MEM_ILL4*fu_mem/MILL4/drug_molar_mass))*MEM_ILL4*fu_mem/MILL4)
48	d(X_CECUM_SOLID)/dt = (X_ILL4_SOLID/TILL4)
49	d(X_CECUM_DISS)/dt = (X_ILL4_DISS/TILL4)
50	d(Villous_DUO)/dt = -(Villous_DUO*Qmuc_DUO/VillousDUO) + (Qmuc_DUO*Artery_drug) + (DIFF_BASO_duo*NI_Membrane*switch_SFdiffbaso*MEM_DUO*fu_mem/MDUO) - (DIFF_BASO_duo*NI_Villous*switch_SFdiffbaso*Villous_DUO*fu_blood/VillousDUO) - (CLINT_influx_baso_DUO*influx_factor_duo_baso*switch_SFinflux_baso*Villous_DUO/VillousDUO) + (((switchVmax_efflux_baso==zero)*CLINT_efflux_baso_DUO*switch_SFefflux_baso*baso_efflux_factor_duo+switchVmax_efflux_baso*baso_efflux_factor_duo*ESA_baso*basoSurfaceRatio_DUO*switch_SFefflux_baso/(drug_Km_efflux_baso+MEM_DUO*fu_mem/MDUO/drug_molar_mass))*MEM_DUO*fu_mem/MDUO)

	ODEs
51	d(Villous_JEJ2)/dt = -(Villous_JEJ2*Qmuc_JEJ2/VillousJEJ2) + (Qmuc_JEJ2*Artery_drug) + (DIFF_BASO_jej2*NI_Membrane*switch_SFdiffbaso*MEM_JEJ2*fu_mem/MJEJ2) - (DIFF_BASO_jej2*NI_Villous*switch_SFdiffbaso*Villous_JEJ2*fu_blood/VillousJEJ2) - (CLINT_influx_baso_JEJ2*influx_factor_jej2_baso*switch_SFinflux_baso*Villous_JEJ2/VillousJEJ2) + (((switchVmax_efflux_baso==zero)*CLINT_efflux_baso_JEJ2*switch_SFefflux_baso*baso_efflux_factor_jej2+switchVmax_efflux_baso*baso_efflux_factor_jej2*ESA_baso*basoSurfaceRatio_JEJ2*switch_SFefflux_baso/(drug_Km_efflux_baso+MEM_JEJ2*fu_mem/MJEJ2/drug_molar_mass))*MEM_JEJ2*fu_mem/MJEJ2)
52	d(Villous_ILL1)/dt = (Qmuc_ILL1*Artery_drug) - (Villous_ILL1*Qmuc_ILL1/VillousILL1) + (DIFF_BASO_ill1*NI_Membrane*switch_SFdiffbaso*MEM_ILL1*fu_mem/MILL1) - (DIFF_BASO_ill1*NI_Villous*switch_SFdiffbaso*Villous_ILL1*fu_blood/VillousILL1) - (CLINT_influx_baso_ILL1*influx_factor_ill1_baso*switch_SFinflux_baso*Villous_ILL1/VillousILL1) + (((switchVmax_efflux_baso==zero)*CLINT_efflux_baso_ILL1*switch_SFefflux_baso*baso_efflux_factor_ill1+switchVmax_efflux_baso*baso_efflux_factor_ill1*ESA_baso*basoSurfaceRatio_ILL1*switch_SFefflux_baso/(drug_Km_efflux_baso+MEM_ILL1*fu_mem/MILL1/drug_molar_mass))*MEM_ILL1*fu_mem/MILL1)
53	d(Villous_ILL2)/dt = -(Villous_ILL2*Qmuc_ILL2/VillousILL2) + (Qmuc_ILL2*Artery_drug) - (DIFF_BASO_ill2*NI_Villous*switch_SFdiffbaso*Villous_ILL2*fu_blood/VillousILL2) + (DIFF_BASO_ill2*NI_Membrane*switch_SFdiffbaso*MEM_ILL2*fu_mem/MILL2) - (CLINT_influx_baso_ILL2*influx_factor_ill2_baso*switch_SFinflux_baso*Villous_ILL2/VillousILL2) + (((switchVmax_efflux_baso==zero)*CLINT_efflux_baso_ILL2*switch_SFefflux_baso*baso_efflux_factor_ill2+switchVmax_efflux_baso*baso_efflux_factor_ill2*ESA_baso*basoSurfaceRatio_ILL2*switch_SFefflux_baso/(drug_Km_efflux_baso+MEM_ILL2*fu_mem/MILL2/drug_molar_mass))*MEM_ILL2*fu_mem/MILL2)
54	d(Villous_ILL3)/dt = -(Villous_ILL3*Qmuc_ILL3/VillousILL3) + (Qmuc_ILL3*Artery_drug) - (DIFF_BASO_ill3*NI_Villous*switch_SFdiffbaso*Villous_ILL3*fu_blood/VillousILL3) + (DIFF_BASO_ill3*NI_Membrane*switch_SFdiffbaso*MEM_ILL3*fu_mem/MILL3) - (CLINT_influx_baso_ILL3*influx_factor_ill3_baso*switch_SFinflux_baso*Villous_ILL3/VillousILL3) + (((switchVmax_efflux_baso==zero)*CLINT_efflux_baso_ILL3*switch_SFefflux_baso*baso_efflux_factor_ill3+switchVmax_efflux_baso*baso_efflux_factor_ill3*ESA_baso*basoSurfaceRatio_ILL3*switch_SFefflux_baso/(drug_Km_efflux_baso+MEM_ILL3*fu_mem/MILL3/drug_molar_mass))*MEM_ILL3*fu_mem/MILL3)
55	d(Villous_ILL4)/dt = -(Villous_ILL4*Qmuc_ILL4/VillousILL4) + (Qmuc_ILL4*Artery_drug) - (DIFF_BASO_ill4*NI_Villous*switch_SFdiffbaso*Villous_ILL4*fu_blood/VillousILL4) + (DIFF_BASO_ill4*NI_Membrane*switch_SFdiffbaso*MEM_ILL4*fu_mem/MILL4) - (CLINT_influx_baso_ILL4*influx_factor_ill4_baso*switch_SFinflux_baso*Villous_ILL4/VillousILL4) + (((switchVmax_efflux_baso==zero)*CLINT_efflux_baso_ILL4*switch_SFefflux_baso*baso_efflux_factor_ill4+switchVmax_efflux_baso*baso_efflux_factor_ill4*ESA_baso*basoSurfaceRatio_ILL4*switch_SFefflux_baso/(drug_Km_efflux_baso+MEM_ILL4*fu_mem/MILL4/drug_molar_mass))*MEM_ILL4*fu_mem/MILL4)
56	d(Villous_JEJ1)/dt = -(Villous_JEJ1*Qmuc_JEJ1/VillousJEJ1) + (Qmuc_JEJ1*Artery_drug) + (DIFF_BASO_jej1*NI_Membrane*switch_SFdiffbaso*MEM_JEJ1*fu_mem/MJEJ1) - (DIFF_BASO_jej1*NI_Villous*switch_SFdiffbaso*Villous_JEJ1*fu_blood/VillousJEJ1) - (CLINT_influx_baso_JEJ1*influx_factor_jej1_baso*switch_SFinflux_baso*Villous_JEJ1/VillousJEJ1) + (((switchVmax_efflux_baso==zero)*CLINT_efflux_baso_JEJ1*switch_SFefflux_baso*baso_efflux_factor_jej1+switchVmax_efflux_baso*baso_efflux_factor_jej1*ESA_baso*basoSurfaceRatio_JEJ1*switch_SFefflux_baso/(drug_Km_efflux_baso+MEM_JEJ1*fu_mem/MJEJ1/drug_molar_mass))*MEM_JEJ1*fu_mem/MJEJ1)
57	d(Liver_drug)/dt = 1/Liver*(-(k_Liver_Venous*Liver_drug) + (k_artery_liver*Artery_drug) + (k_spleen_liver*Spleen_drug) + (Villous_DUO*Qmuc_DUO/VillousDUO) + (Villous_JEJ1*Qmuc_JEJ1/VillousJEJ1) + (Villous_JEJ2*Qmuc_JEJ2/VillousJEJ2) + (Villous_ILL1*Qmuc_ILL1/VillousILL1) + (Villous_ILL2*Qmuc_ILL2/VillousILL2) + (Villous_ILL3*Qmuc_ILL3/VillousILL3) + (Villous_ILL4*Qmuc_ILL4/VillousILL4) - (k_liver_bile*Liver_drug) - (k_liver_metabolites*Liver_drug) + (k_serosa_liver*Serosa_drug))
58	d(Metabolites_liver_drug)/dt = (k_liver_metabolites*Liver_drug)
59	d(Serosa_drug)/dt = 1/Serosa*((k_artery_serosa*Artery_drug) - (k_serosa_liver*Serosa_drug))
60	d(Bile_drug_1)/dt = (k_Liver_IC_S5_Bile_1*Liver_IC_S5_drug_1) + (k_Liver_IC_S4_Bile_1*Liver_IC_S4_drug_1) + (k_Liver_IC_S3_Bile_1*Liver_IC_S3_drug_1) + (k_Liver_IC_S2_Bile_1*Liver_IC_S2_drug_1) + (k_Liver_IC_S1_Bile_1*Liver_IC_S1_drug_1) - (k_transit_1*Bile_drug_1) - (drug_k_bile_deg_1*Bile_drug_1)
61	d(Venous_drug_1)/dt = 1/Venous_1*((k_Liver_EC_S5_Venous_1*Liver_EC_S5_drug_1) + (k_rest_venous_1*Rest_drug_1) + (k_bone_venous_1*Bone_drug_1) + (k_skin_venous_1*Skin_drug_1) + (k_heart_venous_1*Heart_drug_1) + (k_adipos_venous_1*Adipose_drug_1) + (k_muscle_venous_1*Muscle_drug_1) + (k_brain_venous_1*Brain_drug_1) + (k_kidney_venous_1*Kidney_drug_1) - (k_venous_lung_1*Venous_drug_1) - (k_venous_urine_CLR_1*Venous_drug_1) + (k_testes_venous_1*Testes_drug_1) - (k_venous_urine_GFR_1*Venous_drug_1))

	ODEs
62	d(Lung_drug_1)/dt = 1/Lung_1*((k_venous_lung_1*Venous_drug_1) - (k_lung_artery_1*Lung_drug_1))
63	d(Kidney_drug_1)/dt = 1/Kidney_1*(-(k_kidney_venous_1*Kidney_drug_1) + (k_artery_kidney_1*Artery_drug_1))
64	d(Brain_drug_1)/dt = 1/Brain_1*(-(k_brain_venous_1*Brain_drug_1) + (k_artery_brain_1*Artery_drug_1))
65	d(Muscle_drug_1)/dt = 1/Muscle_1*(-(k_muscle_venous_1*Muscle_drug_1) + (k_artery_muscle_1*Artery_drug_1))
66	d(Adipose_drug_1)/dt = 1/Adipose_1*(-(k_adipos_venous_1*Adipose_drug_1) + (k_artery_adipos_1*Artery_drug_1))
67	d(Heart_drug_1)/dt = 1/Heart_1*(-(k_heart_venous_1*Heart_drug_1) + (k_artery_heart_1*Artery_drug_1))
68	d(Skin_drug_1)/dt = 1/Skin_1*(-(k_skin_venous_1*Skin_drug_1) + (k_artery_skin_1*Artery_drug_1))
69	d(Bone_drug_1)/dt = 1/Bone_1*(-(k_bone_venous_1*Bone_drug_1) + (k_artery_bone_1*Artery_drug_1))
70	d(Rest_drug_1)/dt = 1/Rest_1*(-(k_rest_venous_1*Rest_drug_1) + (k_artery_rest_1*Artery_drug_1))
71	d(Artery_drug_1)/dt = 1/Artery_1*(-(k_artery_liver_1*Artery_drug_1) - (k_artery_gut_1*Artery_drug_1) - (k_artery_spleen_1*Artery_drug_1) - (k_artery_rest_1*Artery_drug_1) - (k_artery_bone_1*Artery_drug_1) - (k_artery_skin_1*Artery_drug_1) - (k_artery_heart_1*Artery_drug_1) - (k_artery_adipos_1*Artery_drug_1) - (k_artery_muscle_1*Artery_drug_1) - (k_artery_brain_1*Artery_drug_1) - (k_artery_kidney_1*Artery_drug_1) + (k_lung_artery_1*Lung_drug_1) - (k_artery_testes_1*Artery_drug_1))
72	d(Gut_drug_1)/dt = 1/Gut_1*(-(k_gut_liver_1*Gut_drug_1) + (k_artery_gut_1*Artery_drug_1) + (drug_k_oral_1*drug_fa_1*Gut_Lumen_drug_1))
73	d(Spleen_drug_1)/dt = 1/Spleen_1*(-(k_spleen_liver_1*Spleen_drug_1) + (k_artery_spleen_1*Artery_drug_1))
74	d(Liver_EC_S1_drug_1)/dt = 1/Liver_EC_S1_1*((k_Liver_IC_S1_Liver_EC_S1_1*Liver_IC_S1_drug_1) - (k_Liver_EC_S1_Liver_IC_S1_1*Liver_EC_S1_drug_1) - (k_Liver_EC_S1_Liver_EC_S2_1*Liver_EC_S1_drug_1) + (k_gut_liver_1*Gut_drug_1) + (k_spleen_liver_1*Spleen_drug_1) + (k_artery_liver_1*Artery_drug_1) + (k_Liver_IC_S1_Liver_EC_S1_efflux_1*Liver_IC_S1_drug_1))
75	d(Liver_EC_S2_drug_1)/dt = 1/Liver_EC_S2_1*((k_Liver_IC_S2_Liver_EC_S2_1*Liver_IC_S2_drug_1) - (k_Liver_EC_S2_Liver_IC_S2_1*Liver_EC_S2_drug_1) - (k_Liver_EC_S2_Liver_EC_S3_1*Liver_EC_S2_drug_1) + (k_Liver_EC_S1_Liver_EC_S2_1*Liver_EC_S1_drug_1) + (k_Liver_IC_S2_Liver_EC_S2_efflux_1*Liver_IC_S2_drug_1))
76	d(Liver_EC_S3_drug_1)/dt = 1/Liver_EC_S3_1*((k_Liver_IC_S3_Liver__EC_S3_1*Liver_IC_S3_drug_1) - (k_Liver_EC_S3_Liver_IC_S3_1*Liver_EC_S3_drug_1) - (k_Liver_EC_S3_Liver_EC_S4_1*Liver_EC_S3_drug_1) + (k_Liver_EC_S2_Liver_EC_S3_1*Liver_EC_S2_drug_1) + (k_Liver_IC_S3_Liver_EC_S3_efflux_1*Liver_IC_S3_drug_1))
77	d(Liver_EC_S4_drug_1)/dt = 1/Liver_EC_S4_1*((k_Liver_IC_S4_Liver_EC_S4_1*Liver_IC_S4_drug_1) - (k_Liver_EC_S4_Liver_IC_S4_1*Liver_EC_S4_drug_1) - (k_Liver_EC_S4_Liver_EC_S5_1*Liver_EC_S4_drug_1) + (k_Liver_EC_S3_Liver_EC_S4_1*Liver_EC_S3_drug_1) + (k_Liver_IC_S4_Liver_EC_S4_efflux_1*Liver_IC_S4_drug_1))
78	d(Liver_EC_S5_drug_1)/dt = 1/Liver_EC_S5_1*((k_Liver_IC_S5_Liver_EC_S5_1*Liver_IC_S5_drug_1) - (k_Liver_EC_S5_Liver_IC_S5_1*Liver_EC_S5_drug_1) - (k_Liver_EC_S5_Venous_1*Liver_EC_S5_drug_1) + (k_Liver_EC_S4_Liver_EC_S5_1*Liver_EC_S4_drug_1) + (k_Liver_IC_S5_Liver_EC_S5_efflux_1*Liver_IC_S5_drug_1))
79	d(Liver_IC_S5_drug_1)/dt = 1/Liver_IC_S5_1*(-(k_Liver_IC_S5_Bile_1*Liver_IC_S5_drug_1) - (k_Liver_IC_S5_Metabolites_1*Liver_IC_S5_drug_1) - (k_Liver_IC_S5_Liver_EC_S5_1*Liver_IC_S5_drug_1) + (k_Liver_EC_S5_Liver_IC_S5_1*Liver_EC_S5_drug_1) - (k_Liver_IC_S5_Liver_EC_S5_efflux_1*Liver_IC_S5_drug_1))
80	d(Liver_IC_S3_drug_1)/dt = 1/Liver_IC_S3_1*(-(k_Liver_IC_S3_Metabolites_1*Liver_IC_S3_drug_1) - (k_Liver_IC_S3_Bile_1*Liver_IC_S3_drug_1) - (k_Liver_IC_S3_Liver__EC_S3_1*Liver_IC_S3_drug_1) + (k_Liver_EC_S3_Liver_IC_S3_1*Liver_EC_S3_drug_1) - (k_Liver_IC_S3_Liver_EC_S3_efflux_1*Liver_IC_S3_drug_1))
81	d(Liver_IC_S1_drug_1)/dt = 1/Liver_IC_S1_1*(-(k_Liver_IC_S1_Metabolites_1*Liver_IC_S1_drug_1) - (k_Liver_IC_S1_Bile_1*Liver_IC_S1_drug_1) - (k_Liver_IC_S1_Liver_EC_S1_1*Liver_IC_S1_drug_1) + (k_Liver_EC_S1_Liver_IC_S1_1*Liver_EC_S1_drug_1) - (k_Liver_IC_S1_Liver_EC_S1_efflux_1*Liver_IC_S1_drug_1))
82	d(Liver_IC_S2_drug_1)/dt = 1/Liver_IC_S2_1*(-(k_Liver_IC_S2_Metabolites_1*Liver_IC_S2_drug_1) - (k_Liver_IC_S2_Bile_1*Liver_IC_S2_drug_1) - (k_Liver_IC_S2_Liver_EC_S2_1*Liver_IC_S2_drug_1) + (k_Liver_EC_S2_Liver_IC_S2_1*Liver_EC_S2_drug_1) - (k_Liver_IC_S2_Liver_EC_S2_efflux_1*Liver_IC_S2_drug_1))
83	d(Metabolites_drug_1)/dt = (k_Liver_IC_S5_Metabolites_1*Liver_IC_S5_drug_1) + (k_Liver_IC_S4_Metabolites_1*Liver_IC_S4_drug_1) + (k_Liver_IC_S3_Metabolites_1*Liver_IC_S3_drug_1) + (k_Liver_IC_S2_Metabolites_1*Liver_IC_S2_drug_1) + (k_Liver_IC_S1_Metabolites_1*Liver_IC_S1_drug_1)

	ODEs
84	d(Testes_drug_1)/dt = 1/Testes_1*((k_artery_testes_1*Artery_drug_1) - (k_testes_venous_1*Testes_drug_1))
85	d(Urine_drug_1)/dt = (k_venous_urine_CLR_1*Venous_drug_1) + (k_venous_urine_GFR_1*Venous_drug_1)
86	d(Liver_IC_S4_drug_1)/dt = 1/Liver_IC_S4_1*(-(k_Liver_IC_S4_Metabolites_1*Liver_IC_S4_drug_1) - (k_Liver_IC_S4_Bile_1*Liver_IC_S4_drug_1) - (k_Liver_IC_S4_Liver_EC_S4_1*Liver_IC_S4_drug_1) + (k_Liver_EC_S4_Liver_IC_S4_1*Liver_EC_S4_drug_1) - (k_Liver_IC_S4_Liver_EC_S4_efflux_1*Liver_IC_S4_drug_1))
87	d(Gut_Lumen_drug_1)/dt = -(drug_k_oral_1*drug_fa_1*Gut_Lumen_drug_1) + (k_transit_1*Bile_drug_1) - (drug_k_oral_1*(1-drug_fa_1)*Gut_Lumen_drug_1)

Program Setup

Model Setup

Variants

	Type	Name	Human_phys	Human_physiology_ADAM	Pgp_Ratios_Drozdzik_2019	Talinolol_Caco2Estimate	Talinolol
1	parameter	phys_BW	70				70
2	parameter	phys_Normalized_Q_adipose	4				
3	parameter	phys_Normalized_Q_bone	4				
4	parameter	phys_Normalized_Q_brain	10				
5	parameter	phys_Normalized_Q_gut	17				
6	parameter	phys_Normalized_Q_heart	3				
7	parameter	phys_Normalized_Q_kidney	15				
8	parameter	phys_Normalized_Q_liver	20				
9	parameter	phys_Normalized_Q_lung	80				
10	parameter	phys_Normalized_Q_muscle	14				
11	parameter	phys_Normalized_Q_remainder	1				
12	parameter	phys_Normalized_Q_skin	4				
13	parameter	phys_Normalized_Q_spleen	2				
14	parameter	phys_Normalized_Q_testes	0				
15	parameter	phys_Normalized_weight_adipose	197				

	Type	Name	Human_phys	Human_physiology_ADAM	Pgp_Ratios_Drozdzik_2019	Talinolol_Caco2Estimate	Talinolol
16	parameter	phys_Normalized_weight_artery	25.7				
17	parameter	phys_Normalized_weight_bone	158				
18	parameter	phys_Normalized_weight_brain	21				
19	parameter	phys_Normalized_weight_gut	18				
20	parameter	phys_Normalized_weight_heart	5				
21	parameter	phys_Normalized_weight_kidney	5				
22	parameter	phys_Normalized_weight_liver_blood	4.9				
23	parameter	phys_Normalized_weight_liver_tissue	18				
24	parameter	phys_Normalized_weight_lung	8				
25	parameter	phys_Normalized_weight_muscle	416				
26	parameter	phys_Normalized_weight_remainder	100				
27	parameter	phys_Normalized_weight_skin	41				
28	parameter	phys_Normalized_weight_spleen	3				
29	parameter	phys_HPGL	125				
30	parameter	phys_Normalized_weight_venous	51.4				
31	parameter	LL		680			
32	parameter	LR		1.75			
33	parameter	ESA		120000			
34	parameter	TSTOMACH		16.2			
35	parameter	TDUO		9.384			
36	parameter	TJEJ1		35.292			
37	parameter	TJEJ2		35.292			
38	parameter	TILL1		31.008			
39	parameter	TILL2		31.008			

	Type	Name	Human_phys	Human_physiology_ADAM	Pgp_Ratios_Drozdzik_2019	Talinolol_Caco2Estimate	Talinolol
40	parameter	TILL3		31.008			
41	parameter	TILL4		31.008			
42	parameter	phys_Normalized_weight_stomach		2.1			
43	parameter	pHStomach		1.5			
44	parameter	pHDuo		6.4			
45	parameter	pHJej1		6.5			
46	parameter	pHJej2		6.6			
47	parameter	pHIll1		6.8			
48	parameter	pHIll2		7			
49	parameter	pHIll3		7.7			
50	parameter	pHIll4		7.3			
51	parameter	BW_average		70			
52	parameter	numIntestinalCompartments		7			
53	parameter	Gut_EC_fraction		0.3719			
54	parameter	Gut_IC_fraction		0.6281			
55	parameter	influx_factor_duo		1			
56	parameter	influx_factor_jej1		1			
57	parameter	influx_factor_jej2		1			
58	parameter	influx_factor_ill1		1			
59	parameter	influx_factor_ill2		1			
60	parameter	influx_factor_ill3		1			
61	parameter	influx_factor_ill4		1			
62	parameter	efflux_factor_duo		0.51	0.23		
63	parameter	efflux_factor_jej1		1	1		
64	parameter	efflux_factor_jej2		1.46	1.44		
65	parameter	efflux_factor_ill1		1.5	2.14		
66	parameter	efflux_factor_ill2		1.51	2.14		
67	parameter	efflux_factor_ill3		1.52	2.14		
68	parameter	efflux_factor_ill4		1.51	2.14		
69	parameter	volumeRatio_DUO		0.11534			
70	parameter	volumeRatio_JEJ1		0.22722			
71	parameter	volumeRatio_JEJ2		0.15917			

	Type	Name	Human_phys	Human_physiology_ADAM	Pgp_Ratios_Drozdzik_2019	Talinolol_Caco2Estimate	Talinolol
72	parameter	volumeRatio_ILL1		0.12687			
73	parameter	volumeRatio_ILL2		0.12687			
74	parameter	volumeRatio_ILL3		0.12457			
75	parameter	volumeRatio_ILL4		0.11995			
76	parameter	flowRatio_DUO		0.088			
77	parameter	flowRatio_JEJ1		0.242			
78	parameter	flowRatio_JEJ2		0.242			
79	parameter	flowRatio_ILL1		0.107			
80	parameter	flowRatio_ILL2		0.107			
81	parameter	flowRatio_ILL3		0.107			
82	parameter	flowRatio_ILL4		0.107			
83	parameter	metabolism_factor_duo		1			
84	parameter	metabolism_factor_jej1		1			
85	parameter	metabolism_factor_jej2		1			
86	parameter	metabolism_factor_ill1		1			
87	parameter	metabolism_factor_ill2		1			
88	parameter	metabolism_factor_ill3		1			
89	parameter	metabolism_factor_ill4		1			
90	parameter	phys_Normalized_weight_enterocyte		7.3857			
91	parameter	LumenTotal		126.95			
92	parameter	lumenvolumeRatio_DUO		0.27058			
93	parameter	lumenvolumeRatio_JEJ1		0.16621			
94	parameter	lumenvolumeRatio_JEJ2		0.16621			
95	parameter	lumenvolumeRatio_ILL1		0.099252			
96	parameter	lumenvolumeRatio_ILL2		0.099252			

	Type	Name	Human_phys	Human_physiology_ADAM	Pgp_Ratios_Drozdzik_2019	Talinolol_Caco2Estimate	Talinolol
97	parameter	lumenvolumeRatio_ILL3		0.099252			
98	parameter	lumenvolumeRatio_ILL4		0.099252			
99	parameter	StomachLumenTotal		50			
100	parameter	ESA_baso		6703			
101	parameter	surfaceRatio_DUO		0.115			
102	parameter	surfaceRatio_JEJ1		0.227			
103	parameter	surfaceRatio_JEJ2		0.159			
104	parameter	surfaceRatio_ILL1		0.127			
105	parameter	surfaceRatio_ILL2		0.127			
106	parameter	surfaceRatio_ILL3		0.125			
107	parameter	surfaceRatio_ILL4		0.12			
108	parameter	basoSurfaceRatio_DUO		0.0526			
109	parameter	basoSurfaceRatio_JEJ1		0.2026			
110	parameter	basoSurfaceRatio_JEJ2		0.2026			
111	parameter	basoSurfaceRatio_ILL1		0.1356			
112	parameter	basoSurfaceRatio_ILL2		0.1356			
113	parameter	basoSurfaceRatio_ILL3		0.1356			
114	parameter	basoSurfaceRatio_ILL4		0.1356			
115	parameter	drug_fQ		0.2571			
116	parameter	influx_factor_duo_baso		1			
117	parameter	influx_factor_jej1_baso		1			
118	parameter	influx_factor_jej2_baso		1			
119	parameter	influx_factor_ill1_baso		1			

	Type	Name	Human_phys	Human_physiology_ADAM	Pgp_Ratios_Drozdzik_2019	Talinolol_Caco2Estimate	Talinolol
120	parameter	influx_factor_ill2_baso		1			
121	parameter	influx_factor_ill3_baso		1			
122	parameter	influx_factor_ill4_baso		1			
123	parameter	SFefflux				4.18	
124	parameter	diff_baso				9.44	7
125	parameter	switchVmax_efflux				0.0114	0.00016
126	parameter	drug_Km_efflux				0.567	37
127	parameter	SFinflux				1	
128	parameter	CL_inf_api				0	
129	parameter	CL_inf_baso				0.0715	0
130	parameter	CL_eff_baso				0	
131	parameter	switchVmax_efflux_baso				0.11	
132	parameter	drug_Km_efflux_baso				48.2	
133	parameter	fu_mem					0.0772
134	parameter	LOGP					3.466
135	parameter	MW					363494
136	parameter	REFPHSOL					7.4
137	parameter	PSIZE					0.

	Type	Name	Human_phys	Human_physiology_ADAM	Pgp_Ratios_Drozdzik_2019	Talinolol_Caco2Estimate	Talinolol
							0025
138	parameter	PDENSITY					1200000
139	parameter	pKA					9.43
140	parameter	SOLFASSIF					1234
141	parameter	fu_blood					0.478772
142	parameter	drug_Km_influx					1
143	parameter	switchVmax_influx					0
144	parameter	switch_SFefflux					1
145	parameter	switch_SFgutmet					1
146	parameter	switch_SFdiffapi					1
147	parameter	switch_SFdiffbaso					1
148	parameter	drug_Kp_serosa_raw					5.3838
149	parameter	drug_Kp_liver_raw					8.6052
150	parameter	CLINT_metabolism					0

	Type	Name	Human_phys	Human_physiology_ADAM	Pgp_Ratios_Drozdzik_2019	Talinolol_Caco2Estimate	Talinolol
151	parameter	diff_api					10
152	parameter	CL_eff					1.3614
153	parameter	drug_PSbileg					1.8
154	parameter	drug_fuLiver					0.0772212
155	parameter	drug_fumic					0.497866
156	parameter	drug_funic					0.497866
157	parameter	drug_fuplasma					0.45
158	parameter	drug_CLmetg					0
159	parameter	drug_HLM_CLint					0
160	parameter	drug_Kp_adipose_raw					1.7608
161	parameter	drug_Kp_bone_raw					2.2998

	Type	Name	Human_phys	Human_physiology_ADAM	Pgp_Ratios_Drozdzik_2019	Talinolol_Caco2Estimate	Talinolol
162	parameter	drug_Kp_brain_raw					2.2082
163	parameter	drug_Kp_gut_raw					5.3838
164	parameter	drug_Kp_heart_raw					5.2789
165	parameter	drug_Kp_kidney_raw					4.5849
166	parameter	drug_Kp_lung_raw					8.6052
167	parameter	drug_Kp_muscle_raw					4.7077
168	parameter	drug_Kp_rest_raw					4.2633
169	parameter	switch_SFKp					1
170	parameter	drug_Kp_skin_raw					2.6806
171	parameter	drug_Kp_spleen_raw					5.1

	Type	Name	Human_phys	Human_physiology_ADAM	Pgp_Ratios_Drozdzik_2019	Talinolol_Caco2Estimate	Talinolol
							227
172	parameter	drug_Kp_testes_raw					1
173	parameter	drug_molar_mass					363494
174	parameter	drug_BRP					0.94
175	parameter	switch_SFbile					1
176	parameter	switch_SFmet					1
177	parameter	drug_CLrenal					18.66
178	parameter	drug_FR					0
179	parameter	switch_slow_dist_Kp					1
180	parameter	switch_SFrenal					1
181	parameter	drug_dose_amount_IV					30
182	parameter	drug_dose_rate_IV					15
183	parameter	drug_dose_amount_PO					100
184	parameter	DIFFCOEFF					0.00003918

Data Step

Data Map

Classification	Value
group	Group

Classification	Value
independent	Time_hr_
response	Conc_n_mL_ ~ Plasma_total.Plasma_total_drug
dose from data	Dose_PO_mg -> STOMACH.X_STOMACH_SOLID
	Bolus
dose from data	Dose_IV_mg -> Venous.Venous_drug
	Infusion Data Column: Rate_mg_hr_

Variant and Dose Setup Step

Variant and Dose Setup

Group	Variants1	Variants2	Variants3	Variants4	Variants5	Variants6	Doses1	Doses2	Doses3
	**Baseline**	**Baseline**	**Baseline**	**Baseline**	**Baseline**	**Group Specific**	**Group Specific**	**Data**	**Data**
1	Talinolol_Caco2Estimate	Talinolol	Pgp_Ratios_Drozdzik_2019	Human_physiology_ADAM	Human_phys			Dose_PO_mg	Dose_IV_mg
2	Talinolol_Caco2Estimate	Talinolol	Pgp_Ratios_Drozdzik_2019	Human_physiology_ADAM	Human_phys			Dose_PO_mg	Dose_IV_mg
3	Talinolol_Caco2Estimate	Talinolol	Pgp_Ratios_Drozdzik_2019	Human_physiology_ADAM	Human_phys			Dose_PO_mg	Dose_IV_mg
4	Talinolol_Caco2Estimate	Talinolol	Pgp_Ratios_Drozdzik_2019	Human_physiology_ADAM	Human_phys			Dose_PO_mg	Dose_IV_mg
5	Talinolol_Caco2Estimate	Talinolol	Pgp_Ratios_Drozdzik_2019	Human_physiology_ADAM	Human_phys			Dose_PO_mg	Dose_IV_mg
6	Talinolol_Caco2Estimate	Talinolol	Pgp_Ratios_Drozdzik_2019	Human_physiology_ADAM	Human_phys			Dose_PO_mg	Dose_IV_mg
7	Talinolol_Caco2Estimate	Talinolol	Pgp_Ratios_Drozdzik_2019	Human_physiology_ADAM	Human_phys			Dose_PO_mg	Dose_IV_mg
8	Talinolol_Caco2Estimate	Talinolol	Pgp_Ratios_Drozdzik_2019	Human_physiology_ADAM	Human_phys			Dose_PO_mg	Dose_IV_mg

Fit Step

Estimated Parameters (Pooled Fit)

Name	Transformation	Initial Untransformed Value	Untransformed Bounds
CL_inf_api	log	0.001	[0.0001 1]

Error Model

Use one common error model for all responses: exponential

Algorithm Settings

Property	Value
EstimationFcn	scattersearch

Property	Value
MaxIterations	400
FunctionTolerance	1e-08
MaxStallIterations	50
MaxTime	Inf
NumInitialPoints	300
NumTrialPoints	auto
XTolerance	1e-06
LocalSolver	lsqnonlin

Local Solver Settings

Property	Value
StepTolerance	1e-08
FunctionTolerance	1e-08
OptimalityTolerance	1e-06
MaxIterations	400

Program Results

Fit Step

Pooled Parameter Estimates

Name	Estimate	StandardError
CL_inf_api	0.8375	0.11165

Statistics

Name	Value
AIC	621.7848
BIC	624.6051
LogLikelihood	-309.8924
DFE	123
MSE	8.745
SSE	1075.6382

Pooled Beta

Name	Estimate	StandardError
log(CL_inf_api)	-0.17733	0.13331

Residuals

Group	Time_hr_	Conc_n_mL_
1	0	NaN
1	0.083333	-0.18561
1	0.16667	-0.32581
1	0.25	-0.38312
1	0.33333	-0.43139

Group	Time_hr_	Conc_n_mL_
1	0.5	-0.40365
1	0.66667	-0.30654
1	1	-0.14414
1	1.3333	-0.091877
1	1.6667	0.01135
1	2	0.04041
1	2.5	-0.015784
1	3	0.12644
1	3.5	0.14957
1	4	0.13936
1	5	0.084212
1	6	0.19176
1	8	0.3411
1	10	0.4375
1	12	0.50422
1	24	32.0676
2	0	NaN
2	0.25	0.44179
2	0.5	-0.055967
2	0.75	-0.14919
2	1	-0.083669
2	1.25	-0.1089
2	1.5	-0.1462
2	1.75	-0.1153
2	2	-0.23891
2	2.5	-0.059562
2	3	0.062421
2	3.5	0.076817
2	4	0.0388
2	6	-0.012771
3	0	NaN
3	0.17	-0.36084
3	0.33	-0.37441
3	0.5	-0.38219
3	0.67	-0.24623
3	1	-0.11208
3	1.5	-0.082256
3	2	-0.070085
3	2.5	0.029412
3	3.5	-0.035104
3	4	-0.2869

Group	Time_hr_	Conc_n_mL_
3	4.5	-0.10896
3	6.5	-0.1663
3	8.5	-0.19247
3	12.5	-0.24908
3	16.5	-0.24759
3	24.5	-0.63006
4	0	NaN
4	0.33333	1.3283
4	0.66667	1.8355
4	1	1.9591
4	1.3333	1.2606
4	1.6667	0.71308
4	2	0.36569
4	2.5	-0.33508
4	3	-0.86744
4	3.5	-0.97962
4	4	-0.90693
4	5	-0.84305
4	6	-0.70657
4	8	-0.62703
4	10	-0.61167
4	12	-0.631
4	24	-1.001
5	0	NaN
5	0.33333	0.57757
5	0.66667	0.67684
5	1	0.62148
5	1.3333	0.46543
5	1.6667	0.32175
5	2	0.076343
5	2.5	-0.059786
5	3	-0.42748
5	3.5	-0.70447
5	4	-0.697
5	4.5	-0.7378
5	5	-0.77939
5	6	-0.68165
5	7	-0.53678
5	8	-0.53811
5	10	-0.38042
5	12	-0.38912

Group	Time_hr_	Conc_n_mL_
5	24	-0.60966
6	0	NaN
6	0.25	1.3092
6	0.5	0.85378
6	0.75	0.45775
6	1	0.36892
6	1.25	0.28762
6	1.5	0.32721
6	1.75	0.27376
6	2	0.2576
6	2.5	-0.16241
6	3	-0.61084
6	3.5	-0.80382
6	4	-0.93616
6	6	-0.97455
6	8	-0.74218
6	12	-0.75508
7	0	NaN
7	24	NaN
7	48	NaN
7	72	NaN
7	96	NaN
7	120	NaN
7	144	-1.2191
7	144.5	0.46721
7	145	0.52641
7	145.5	0.27993
7	146	0.10913
7	146.5	-0.28102
7	147	-0.53119
7	147.5	-0.82529
7	148	-0.74294
7	150	-0.79495
7	152	-0.73807
7	156	-0.807
7	160	-0.93579
7	168	-1.2741
8	0	NaN
8	0.25	1.8063
8	0.5	0.8197
8	0.75	0.37231

Group	Time_hr_	Conc_n_mL_
8	1	0.22942
8	1.5	0.40484
8	2	0.34252
8	2.5	-0.092743
8	3	-0.44371
8	4	-0.78933
8	6	-0.74131
8	8	-0.56802
8	12	-0.61461

Covariance Matrix

Name	CL_inf_api
CL_inf_api	0.012466

Error Model

Response	ErrorModel	a
Conc_n_mL_	exponential	2.9453

Report generated by SimBiology v. 25.1 (R2025a) on 16-Sep-2025 16:03:07
